# A novel *TP53* pathway influences the *HGS*-mediated exosome formation in colorectal cancer

**DOI:** 10.1038/srep28083

**Published:** 2016-06-17

**Authors:** Yulin Sun, Weiwei Zheng, Zhengguang Guo, Qiang Ju, Lin Zhu, Jiajia Gao, Lanping Zhou, Fang Liu, Yang Xu, Qimin Zhan, Zhixiang Zhou, Wei Sun, Xiaohang Zhao

**Affiliations:** 1 State Key Laboratory of Molecular Oncology, National Cancer Center/Cancer Hospital, Chinese Academy of Medical Sciences & Peking Union Medical College, Beijing 100021, China; 2 Center of Basic Medical Science, Navy General Hospital, Beijing 100048, China; 3 Core Facility of Instruments, Institute of Basic Medical Sciences, Chinese Academy of Medical Sciences & Peking Union Medical College, Beijing 100005, China; 4 Department of Clinical Biochemistry, Chinese PLA General Hospital, Beijing 100853, China; 5 Department of Colorectal Surgery, National Cancer Center/Cancer Hospital, Chinese Academy of Medical Sciences & Peking Union Medical College, Beijing 100021, China

## Abstract

Tumor-derived exosomes are important for cell-cell communication. However, the role of *TP53* in the control of exosome production in colorectal cancer (CRC) is controversial and unclear. The features of exosomes secreted from HCT116 *TP53*-wild type (WT), *TP53*-knockout (KO) and constructed *TP53* (R273H)-mutant (MT) cells were assessed. The exosomes from the MT and KO cells exhibited significantly reduced sizes compared with the WT cells. A comprehensive proteomic analysis of exosomal proteins was performed using the isobaric tag for relative and absolute quantitation (iTRAQ)-2D-LC-MS/MS strategy. A total of 3437 protein groups with ≥2 matched peptides were identified. Specifically, hepatocyte growth factor-regulated tyrosine kinase substrate (HGS) was consistently down-regulated in the exosomes from the MT and KO cells. Functional studies demonstrated that low HGS levels were responsible for the decreased exosome size. *TP53* regulated *HGS* expression and thus HGS-dependent exosome formation. Furthermore, the HGS expression was gradually increased concomitant with CRC carcinogenesis and was an independent poor prognostic factor. In conclusion, a novel HGS-dependent *TP53* mechanism in exosome formation was identified in CRC. HGS may serve as a novel prognostic biomarker and a candidate target for therapeutic interventions.

Colorectal cancer (CRC) is the third leading prevalent cause of death from cancer in adults^1^. The disease begins as a benign adenomatous polyp, and it subsequently develops into an advanced adenoma and gradually progresses to an invasive cancer. The driving factors behind CRC comprise a series of successive accumulated gene mutations that follow the order “*APC-KRAS-TP53-DCC*”^2^,^3^.

The tumor suppressor gene *TP53* encodes a 393 amino acid phosphoprotein. TP53 acts as a critical regulator of cell cycle arrest, apoptosis and the DNA damage response^4^. Importantly, *TP53* is one of the most commonly mutated genes in human tumors. The frequency of reported TP53 mutations in CRC is approximately 50%, and the mutations primarily affect five ‘hotspot’ codons (175, 245, 248, 273 and 282)^5^,^6^,^7^. According to the International Agency for Research on Cancer (IARC) TP53 database (http://p53.iarc.fr/), these five hotspot mutations occur in CRC with frequencies of 10.5%, 5.5%, 10.6%, 9.7% and 4.8%, respectively. Notably, mutated TP53 also exhibits new oncogenic functions, such as the promotion of proliferation and invasion^4^. Here, we focused on the R273H mutation, which changes the amino acid at codon 273 from arginine to histidine. This mutation has been reported to increase tumor cell proliferation, migration and invasion in breast and lung cancers^9^,^10^.

Exosomes are nano-sized secreted membrane-enclosed vesicles (30–100 nm in diameter) with a “saucer-shape” morphology. The biogenesis mechanisms of exosomes have not been fully elucidated. In general, exosomes are formed from the intraluminal vesicles (ILVs) of multivesicular bodies (MVBs) within the endosomal network. During the maturation of late endosomes, some contents are preferentially sorted into 30–100 nm vesicles that bud into the lumen of late endosomes; these vesicles are referred to as MVBs. The endosomal sorting complex required for transport (ESCRT) pathway comprises five distinct complexes (ESCRTs -0, -I, -II, -III and Vps4) and is a key mediator of MVB biogenesis and the sorting of endosomal cargo proteins into MVBs^11^. An alternative endosomal sorting pathway dependent on CD63 but not ESCRT has also been reported^12^. Some MVBs are fated for degradation, whereas other MVBs are exported following the fusion of the MVB with the plasma membrane^13^. Exosomes contain proteins, lipids, mRNA and miRNA that serve as cargo to deliver messages for cell-cell communication; thus, exosomes play roles in tumor microenvironment remodelling^14^.

Previous studies have demonstrated that the production of exosomes was absent in *TP53*-null cells, whereas wild-type *TP53* enhanced exosome release via the upregulation of *TSAP6* and *CHMP4C*^15^,^16^. Another study indicated that there was no correlation between the tissue TP53 protein expression, TSAP6 (mRNA and protein) and plasma exosome levels in CRC. Furthermore, exosome release was independent of the *TP53* status and *TSAP6* mRNA levels in HCT116 *TP53*-wild type and *TP53*-null cells^17^. These contradictory findings prompted us to perform a comprehensive proteomic analysis of exosomes purified from HCT116 *TP53*-wild type, *TP53*-knockout and *TP53*-mutant cells.

The current findings demonstrated that *TP53*-mutant (R273H) and *TP53*-null cells secreted smaller-sized exosomes compared with the *TP53*-wild type cells. The exosomal protein composition was identified using a chemical label-based isobaric tag for relative and absolute quantitation (iTRAQ) procedure coupled with two-dimensional liquid chromatography and tandem mass spectrometry (MS/MS) analyses. Furthermore, we experimentally confirmed that a differentially expressed protein that was decreased in the exosomes of both the *TP53*-mutant and *TP53*-null cells was responsible, at least in part, for the decreased exosome size. Thus, our study indicated a novel mechanism by which TP53 regulated carcinogenesis by affecting the important component in tumor microenvironment.

## Results

### TP53 mutant (R273H) enhances cell proliferation and migration

HCT116 colon cancer cells express the wild-type *TP53* gene. To construct a cell line that stably expressed the *TP53* R273H mutant, a missense mutant vector of the *TP53* gene was introduced into HCT116 cells [HCT116 *TP53*(R273H)-mutant, MT]. We subsequently investigated the impact of the *TP53* R273H mutant on cell proliferation and migration. We determined that the growth rate of the MT cells was significantly increased compared with the growth of the cells that expressed the empty vector control. The wound-healing assay indicated that the healing speed was faster in the MT cells compared with the controls (Supplementary Fig. S1A,B). Western blotting analysis indicated that the TP53 level in the MT cells was substantially increased compared with the empty vector control, whereas the HCT116-*TP53*^(−/−)^ (KO) cells did not express TP53 (Supplementary Fig. S1C). These findings indicated that the *TP53* mutant (R273H) cell model was successfully constructed.

### Exosomes secreted from *TP53* mutant and knockout cells have a smaller size

**Figure 1:**
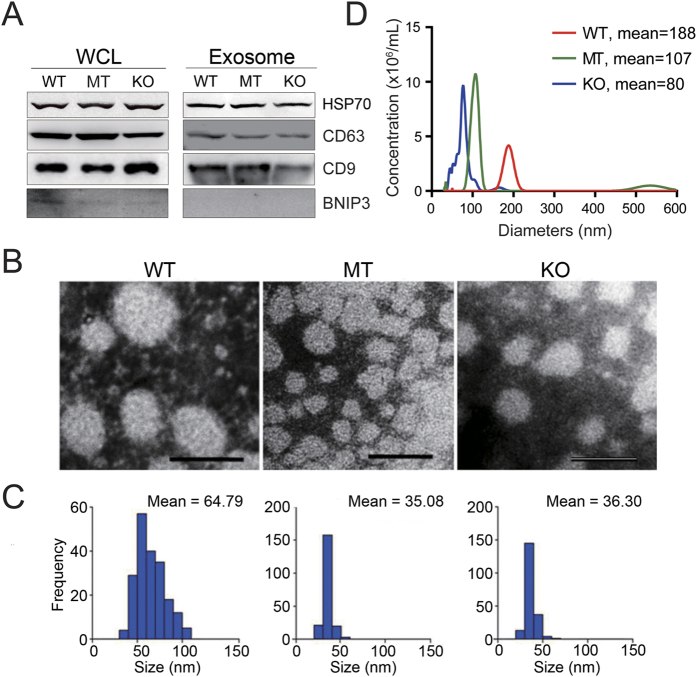
Exosomes purified from HCT116 cells with different *TP53* statuses exhibit distinct sizes. (**A**) Western blot analysis of whole cell lysates (WCL) and vesicles isolated from serum-free conditional media from wild-type *TP53* (WT), *TP53* (R273H) mutant (MT) and *TP53*-null (KO) HCT116 cells. The exosomal markers HSP70, CD63 and CD9 and the mitochondrial protein BNIP3 were detected. The experiments were performed at least in triplicate. (**B**) Electron micrograph of exosomes isolated from WT, MT and KO cells. Negative-staining images indicated that the exosomes exhibited a smooth, saucer-like morphology. Scale bars are 100 nm. (**C**) The mean diameter distribution of 200 exosomes was calculated from electron micrograph images, and the exosome frequency was plotted for the indicated sizes. (**D**) Representative size distribution of exosomes isolated from three groups via NanoSight particle-tracking analysis.

Exosomes secreted from the HCT116 wild-type *TP53* (WT), MT and KO cells were precipitated using traditional ultracentrifugation and nanomaterial methods. Western blotting analysis indicated that the nanoparticles contained the exosome-specific markers HSP70, CD63 and CD9 but not the mitochondrial protein BNIP3 (Fig. 1A). Transmission electron microscopy indicated that the extracted exosomes exhibited a smooth, saucer-like shape (Fig. 1B). Interestingly, the calculation of the diameters of 200 exosomes per group indicated that the WT, MT and KO vesicles had mean diameters of 64.79 ± 15.98, 35.08 ± 5.00 and 36.30 ± 5.33 nm, respectively (Fig. 1C). Statistical analysis demonstrated that the size differences between the WT and MT and the WT and KO groups were significant (*P* < 0.0001). The exosome size alterations of these three cells were further confirmed by Nanosight particle-tracking technology, and similar tendencies were identified (Fig. 1D). Taken together, our findings indicated that the exosomes secreted from the MT and KO cells were significantly smaller than the exosomes secreted from the WT cells.

### Exosomal protein profiling by iTRAQ-2D-LC-MS/MS

To determine whether the altered protein composition corresponded to the reduced size of the exosomes from the MT and KO cells, we performed a comprehensive proteomic analysis (Supplementary Fig. S2A). To minimize the impact of technical variations on true biological differences, we introduced technical repeats and biological duplicates, respectively. For the biological duplicates, two batches of exosomes were isolated from the MT cells; for the technical repeats, each peptide fractionation was split into two identical portions following offline high pH RP-HPLC separation to determine the variation during the subsequent RP-HPLC-MS/MS analysis. As shown in Supplementary Fig. S2B,C, the R-squared of the identified proteins between duplicate experiments was greater than 0.85, which implies an acceptable reproducibility and a well-controlled experimental process.

**Figure 2:**
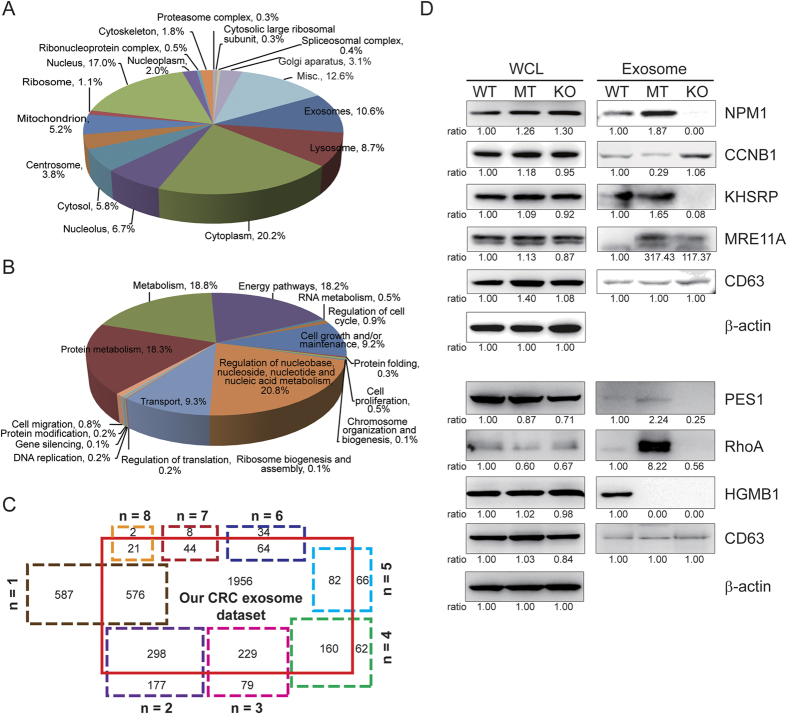
Bioinformatics analysis and Western blotting validation of 3437 quantified exosomal proteins. (**A**) Pie chart of the significantly enriched cellular component analysis based on Gene Ontology annotation. Classifications that contained <0.3% of the total proteins were categorized as miscellaneous (Misc.). (**B**) Pie chart of the biological process analysis based on Gene Ontology annotation. (**C**) Venn diagram indicates the presence, absence, or overlap of proteins identified by our iTRAQ-2D-LC-MS/MS strategy and 2489 previously reported exosomal proteins in CRC cells derived from 8 proteomic studies. These 2489 known proteins were categorized into 8 groups based on their identification number counts, which ranged from n = 1 to n = 8. The higher identified counts represented higher reliability. (**D**) Western blotting validation of the differentially expressed exosomal proteins. The densitometry was performed to quantify each lane, and the ratio of each protein over the loading control β-actin in the whole cell lysate or CD63 in exosome is presented under each blot, with the ratio in the WT cells being the reference value. Based on our quantitative proteomic results, the CD63 expression was relatively consistent among the three types of exosomes, thus it was used as a loading control for exosome fraction in this study. The representative Coomassie blue-stained SDS-PAGE gel was shown in Supplementary Fig. S3. The results indicate equivalent levels of exosomal proteins in the three preparations.

In the iTRAQ experiment, 3437 protein groups were identified with confidence when at least two matched peptides were above the MASCOT significance level. The total FDR was controlled to ≤1% (Supplementary Table S1). Specifically, 3430 protein groups were assigned to a gene. We initially annotated our identified proteins by Gene Ontology (GO). The cellular component classification demonstrated that the proteins from the exosome, lysosome, cytoplasm and nucleolus were highly enriched (*P* < 0.0001, Fig. 2A). These proteins were primarily involved in the biological processes of protein metabolism, energy pathway, RNA metabolism, cell cycle regulation and cell growth and maintenance (*P* < 0.0001, Fig. 2B). We compared our identified protein groups with the previously reported CRC exosome proteins from the Vesiclepedia database (http://www.microvesicles.org/, v3.1). There were 1474 (43.0%) overlapping proteins, whereas 1956 proteins were recognized as exosomal proteins from CRC cells in our study for the first time. A total of 1015 known CRC exosome proteins were not identified in our work (Fig. 2C).

By setting the exosomal proteins from the WT cells as the reference, a total of 3173 proteins were successfully quantified. After removing the proteins with a CV >0.5 in the technical and biological replications, 3003 proteins remained (Supplementary Table S2). The quantification CV was <10.7% for the four technical replicates.

To more stringently screen proteins with biologically significant changes, we applied the criteria fold change ≤0.63 and ≥1.60 and *P* ≤0.05. Finally, 149 differentially expressed proteins were obtained between the WT and MT cell exosomes (MT vs. WT, Supplementary Table S3), 494 proteins between the WT and KO cell exosomes (KO vs. WT, Supplementary Table S4), and 825 proteins between the MT and KO cell exosomes (MT vs. KO, Supplementary Table S5).

### Western blotting validation of differentially expressed exosomal proteins

To confirm the iTRAQ-2D-HPLC-MS/MS results, a western blotting analysis was performed for specific candidate proteins (Fig. 2D). Similar to the proteomics results, NPM1, KHSRP, PES1 and RhoA exhibited increased expression levels in the MT exosomes, whereas CCNB1 had an increased expression level in the KO exosomes. Moreover, the expression of MRE11A was increased in both the MT and KO exosomes compared with the WT exosomes, whereas HMGB1 was more strongly expressed in the WT exosomes. Thus, the changing tendency identified in the western blotting analysis was consistent with our quantitative proteomic results.

### Commonly differentially expressed proteins in the MT and KO exosomes

To investigate the potential mechanisms that underlie the reduced size of the exosomes identified in the MT and KO cells, we analyzed the 41 differentially expressed proteins shared by the MT vs. WT groups and the KO vs. WT groups (Supplementary Table S6). Seventeen of the 41 proteins were down-regulated, and 14 of the 41 proteins were up-regulated in both groups; the remaining proteins exhibited discordant expression trends between the groups.

### Knockdown of HGS reduces the exosome size

**Figure 3:**
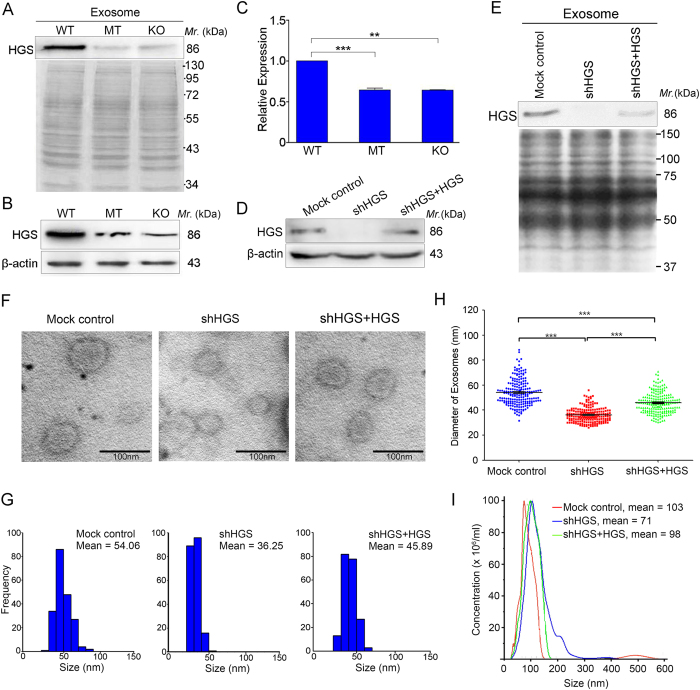
HGS depletion results in smaller exosomes. (**A**) Confirmation of the HGS levels in exosomes from different cell lines using Western blotting. A Coomassie blue-stained SDS-PAGE gel was used as the loading control. (**B**) HGS protein levels detected by Western blotting in the whole cell lysates from wild-type *TP53* (WT), *TP53* (R273H)-mutant (MT) and *TP53*-null (KO) HCT116 cells. β-actin was used as the internal control. (**C**) Real-time PCR detection of the *HGS* mRNA levels in the WT, MT and KO cells. **P < 0.01; ***P < 0.001. (**D**) Knockdown and rescue of HGS in HCT116 cells. The HCT116 cells were stably transfected with pLKO.1-shHGS or the mock control plasmid using lentiviruses. For the rescue experiments, HGS was ectopically expressed in HCT116-shHGS cells via transfection with the pCMV6-Myc-DDK-HGS plasmid. β-actin was used as the internal control. (**E**) Western blotting detection of HGS levels in exosomes secreted from the HGS knockdown (HCT116-shHGS), HGS recovered (HCT116-shHGS-HGS) and mock control cells. A Coomassie blue-stained SDS-PAGE gel was used as the loading control. (**F**) Representative electron micrographs of exosomes isolated from serum-free conditional media collected from the indicated groups. Scale bar, 100 nm; direct magnification, 120,000x. (**G**) The mean diameter distribution of 200 exosomes was calculated from the electron micrograph images, and the exosome frequency was plotted for the indicated size. (H) Statistical analysis of the size distribution for exosomes derived from each group. n = 200 exosomes per group; ***P < 0.0001. (**I**) Representative size distribution of exosomes isolated from the indicated groups via NanoSight particle-tracking analysis.

Of the 31 differentially expressed proteins with consistent alterations in the MT vs. WT and KO vs. WT cells, the expression level of hepatocyte growth factor-regulated tyrosine kinase substrate (HGS) was increased in the exosomes secreted from the WT cells compared with the exosomes secreted from the MT and KO cells (MT vs. WT, ratio 0.63; KO vs. WT, ratio 0.60). HGS has been reported to be involved in exosome biogenesis. Therefore, we subsequently investigated the relationship between HGS and the reduced exosome size. We initially confirmed the expression of HGS in the exosomes from the three cell lines (Fig. 3A). HGS was down-regulated in the MT and KO exosomes compared with the WT exosomes, which is consistent with our proteomic results. Second, HGS expression was detected in the whole cell lysates of the three cells. As shown in Fig. 3B, HGS was also decreased in the MT and KO cells compared with the WT cells. Third, a qRT-PCR assay was performed to determine whether its dysregulation occurred at the transcriptional level. We determined that the *HGS* mRNA was significantly decreased in the MT and KO cells (Fig. 3C), which indicates that *TP53* may regulate the transcription of *HGS*, and the *TP53* (R273H) mutant did not retain this function.

To determine whether the lower HGS levels may reduce the exosome size, we knocked down endogenous *HGS* in HCT116 cells using a stable shRNA lentivirus system (Fig. 3D,E). The results demonstrated that the mean exosome diameter significantly decreased from 54.06 ± 10.34 to 36.25 ± 5.62 nm following HGS depletion (*P* < 0.0001; Fig. 3F,H). Moreover, the HCT116 cells transfected with a specific siRNA that targeted HGS also secreted smaller exosomes compared with the scramble controls (Supplementary Fig. S4). To determine whether HGS was sufficient for the identified effect on the exosome size, a rescue assay was performed by adding back the shRNA-resistant HGS plasmid. Western blotting indicated that the HGS expression in the transfected total cell lysate recovered to the control levels; however, the protein amount in the exosomes was incompletely reinstated (Fig. 3D,E). Electron microscopy analysis demonstrated that the exosome size was partially rescued in the transfected cells compared with the HGS stable knockdown cells (mean diameter: 45.89 ± 8.14 vs. 36.25 ± 5.62 nm, respectively, *P* < 0.0001); however, the exosome size remained smaller than the mock control cells (mean diameter: 45.89 ± 8.14 vs. 54.06 ± 10.34 nm, respectively; *P* < 0.0001; Fig. 3F,H). Furthermore, the alterations in the exosome size identified in the mock control, HGS stable knockdown and rescued expression cells were confirmed through the high-resolution NanoSight system (Fig. 3G,I). Therefore, it appears likely that the lower levels of HGS contributed to the smaller exosome size, and HGS was necessary for the maintenance of the exosome size.

### HGS mRNA expression is associated with TP53 status in human CRC tissues

**Figure 4:**
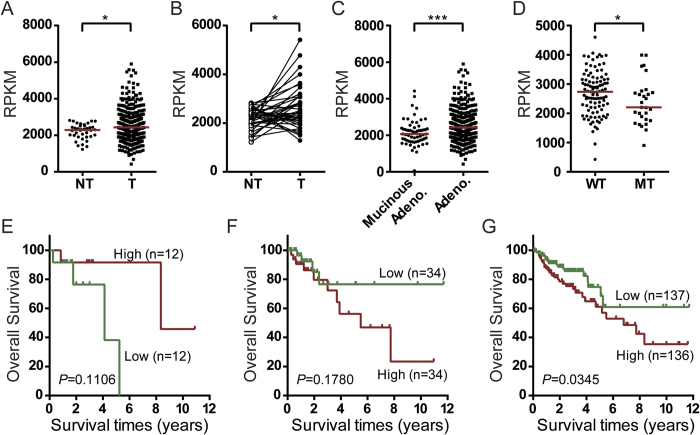
HGS transcript expression was associated with *TP53* status in the RNA-Seq dataset of colon cancer from TCGA. In the University of California, Santa Cruz, genome browser database (UCSC, hg19), the HGS gene has two transcripts, uc002kbg.3 and uc010wus.2. The former transcript comprises the primary transcript, and its average abundance in CRC is more than 350-fold compared with uc010wus.2. In addition, the TP53 gene possesses 13 transcripts. Uc002gij.3 is the dominant full-length variant, whereas the other transcripts are barely expressed in CRC. Therefore, we performed these analyses based on uc002kbg.3 and uc002gij.3. (**A**) HGS mRNA was significantly up-regulated in the tumor tissues of patients with colon adenocarcinoma (n = 392) compared with the adjacent non-tumor tissues (n = 38). *P < 0.05. (**B**) In paired colon adenocarcinoma and non-tumor tissues (n = 38), the same tendency was observed. *P < 0.05. (**C**) For the two pathological subtypes of colon cancer, the HGS mRNA levels were significantly increased in the tumor tissues of adenocarcinoma (n = 392) compared with mucinous adenocarcinoma (n = 62). ***P < 0.0001. (**D**) In colon adenocarcinoma, the somatic mutation of the *TP53* gene was validated in 130 patients. The HGS mRNA levels were significantly lower in the *TP53* mutant group (MT, n = 29) compared with the wild-type group (WT, n = 101). *P < 0.05. (**E**) Kaplan-Meier curve of colon adenocarcinoma patients with TP53 mutation (n = 48). (**F**) Kaplan-Meier curve of colon adenocarcinoma patients with wild-type TP53 (n = 68). (**G**) Kaplan-Meier curve of all patients with colon adenocarcinoma (n = 273). Log-rank test was performed in (**E–G**).

To determine whether *HGS* mRNA was also affected by the TP53 mutant in clinical human samples, we analyzed the normalized gene isoform RNA sequencing dataset of colon cancer patients (n = 459) from The Cancer Genome Atlas (TCGA). We initially compared the abundance of the dominant transcript of *HGS* between tumor and non-tumor tissues. The levels were significantly increased in the tumor tissues (all *P* < 0.05; Fig. 4A,B). Of the different pathological subtypes of colon cancer, we subsequently determined that the HGS level in colon mucinous adenocarcinoma was substantially lower than colon adenocarcinoma (*P* < 0.0001; Fig. 4C). Furthermore, in patients with colon adenocarcinoma, the somatic mutation status of *TP53* was validated in 130 cases. Notably, we determined that the HGS expression was significantly lower in the *TP53* mutant group (Fig. 4D), which is consistent with our findings in the WT and MT cells.

In patients with mutant *TP53*, wild-type *TP53* and all cases with available overall survival, we divided each group into two subgroups, high levels and low levels, based on the median RPKM (Reads per kilobase of transcript per million reads mapped) value of *HGS* in tumor tissues. Importantly, the Kaplan-Meier curves analysis indicated that low levels of *HGS* were slightly associated with a shorter survival time in the *TP53* mutant group (Fig. 4E). However, in the *TP53* wild type group, the patients with high levels of HGS were prone to live for a shorter time (Fig. 4F). When all cases were analyzed, high levels of HGS were significantly correlated with shorter overall survival (*P* = 0.0345, Fig. 4G).

### Overexpression of HGS is associated with poor prognosis in CRC

**Figure 5:**
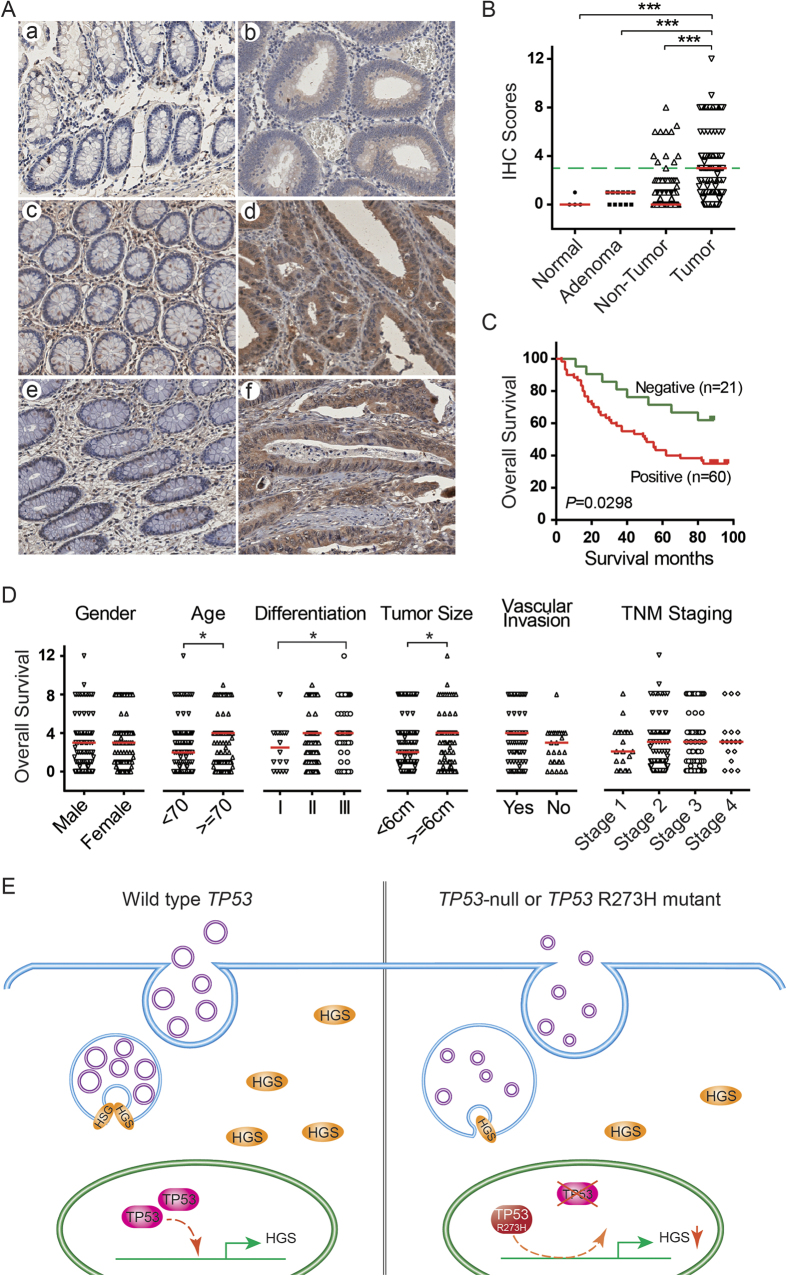
Expression and clinicopathological characteristics of HGS protein in human CRC samples. (**A**) Representative immunohistochemistry images of HGS expression in normal colorectal mucosa (a), tubular adenoma (b), adjacent non-tumor tissues (c,e) and CRC tumors (d,f) (x200 magnification). (**B**) Distribution of HGS levels determined by immunohistochemistry in the normal colorectal mucosa (n = 4), tubular adenoma (n = 11), adjacent non-tumor tissue and matched CRC tumor (n = 235) groups. The short red line represents the median value of each group. The green dashed line represents the threshold of positive staining. (**C**) Kaplan-Meier curve of CRC patients with negative and positive HGS expression. The log-rank test was performed. (**D**) Clinicopathological characteristic analysis of HGS expression in 235 CRC cases. The short red line represents the median value of each group. ***P < 0.0001; *P < 0.05. (**E**) A schematic diagram of the roles of *TP53* in exosome production in CRC. Wild-type *TP53* stimulates the high level expression of HGS. As a key molecule for MVB sorting, HGS recognizes and subsequently directs cargos to the endosomal lumen. With the cooperation of the other ESCRT complexes, the mature MVBs contain several ILVs that form and fuse with the plasma membrane to release their ILVs, which are referred to as exosomes. However, when *TP53* is deficient or mutated at codon 273 (R273H), HGS expression is decreased. In this situation, HGS-dependent ILV formation is inhibited. Enlarged MVBs that contain uniformly small ILVs are present. HGS is necessary to maintain the size and control the components of exosomes.

To confirm the aberrant expression of HGS identified at the transcriptional level, an immunohistochemical staining assay was performed in normal colorectal mucosa (n = 4), tubular adenoma (n = 11) and 235 pairs of malignant tumors and their matched adjacent normal colorectal tissues. HGS was barely detected in the normal colorectal mucosa. The negative expression of HGS remained in the benign tubular adenoma tissues; however, there was a slight increase (Fig. 5A,B). Positive cytoplasmic immunostaining for HGS was observed in 4.7% (11/235) of the adjacent non-cancerous tissues and 45.1% (106/235) of the CRC tumors, respectively. The upregulation of HGS in the CRC tumor tissues was significant compared with the adjacent non-tumor, tubular adenoma and normal colorectal tissues (all *P* < 0.0001).

The subsequent Kaplan-Meier survival analysis indicated a significant correlation between positive cytoplasmic HGS expression levels and a shorter overall survival time (*P* = 0.0298) in the CRC patients (Fig. 5C). The median survival times of the positive and negative expression groups were 50 and 87 months, respectively. These findings were confirmed by both univariate and multivariate Cox regression analyses (Table 1). In the univariate analysis, the HGS-positive patients exhibited a 2.27-fold increase in the RR for overall survival compared with the HGS negative expression group (*P* = 0.0353). The other significant risk factors included vascular invasion, tumor invasive depth, lymph node metastasis, distant metastasis and TNM staging. In the multivariate analysis, only HGS expression (RR = 3.34, *P* = 0.0221), TNM staging (RR = 2.37, *P* = 0.0006) and vascular invasion (RR = 5.50, *P* = 0.0167) were independent prognostic factors for mortality.

The correlations between the clinicopathological characteristics and HGS expression levels were also analyzed (Fig. 5D). The HGS levels were increased in the patients with older age (*P* = 0.0169), poor differentiation (*P* = 0.0340) and larger tumor sizes (*P* = 0.0259). No correlation was observed between HGS and other features, such as gender, vascular invasion or TNM staging.

## Discussion

During the stepwise formation of CRC from normal colonic tissues, the *TP53* mutation is the second key genetic step between the late adenoma and carcinoma stages that closely follow the initial *APC* gene mutation^3^. As a result of the gain-of-function of mutant *TP53*, these mutations were associated with advanced stage tumors and vascular and lymphatic invasions in CRC^18^,^19^. In patients with Dukes’ stage D tumors, *TP53* mutations were also associated with a significantly worse outcome^18^. In this study, the ectopic expression of the *TP53* mutant R273H in HCT116 cells enhanced proliferation and migration, which was consistent with the previous literature^9^,^10^,^20^,^21^,^22^.

To date, 8 proteomic studies have identified exosomal proteins in CRC cells. The numbers of exosomal proteins range from 111 to 1148^23^,^24^,^25^,^26^,^27^,^28^,^29^,^30^. We classified 2489 proteins into 8 groups based on their counts of identification numbers and subsequently overlapped these data with our CRC exosome dataset (n = 3430). With the exception of two groups with identification counts ≥7 that had a >85% coincidence rate, the remaining six groups overlapped with similar proportions to the proteins in our dataset (which ranged from 49.5 to 74.4%) (Fig. 2C). Moreover, our analysis identified 1956 additional proteins, whereas 1015 previously reported CRC exosome proteins were not included in our dataset. The non-identified or newly identified proteins may result from the different CRC cell lines, exosome isolation methods, and proteomic technologies used in different studies. However, there is no doubt that our study produced the largest CRC exosome dataset to date.

Mutated oncogenes may alter exosome secretion. Mutant KRAS dramatically affects the composition of the exosome proteome in CRC: exosomes from mutant KRAS cells contain many tumor-promoting proteins, including KRAS^27^. However, the TP53 protein was not identified in our study or the previously reported CRC exosomal proteins, which suggests that TP53 was not transferred between cells.

Previous studies have demonstrated that the production of exosomes was dependent on the *TP53* status and was stimulated by its target genes *TSAP6* and *CHMP4C* under irradiation stress^15^,^16^. A subsequent study in *Tsap6*-null mice confirmed that exosome production was severely compromised in *Tsap6*-null cells^31^. However, a recent study demonstrated that the TP53/TSAP6 pathway did not regulate the release of exosomes into the plasma of CRC patients^17^. TSAP6 is a glycosylated ferrireductase that participates in the regulation of the endosomal pathway^31^. TSAP6 was detected in the exosomes of lung cancer cells, mouse embryo fibroblasts and mouse spleen tissues^15^,^16^,^31^; however, it has only been identified once in the exosomes from DLD-1 cells in CRC^27^. CHMP4C, which is a component of the ESCRT-III protein complex, is regulated by *TP53* and has been found in the exosomes of lung cancer cells^16^. In CRC, CHMP4C has been recognized as a differentially expressed exosomal protein in metastatic SW620 compared with primary SW480 cells^30^. However, these proteins were not identified in our study. It is possible that the occurrence of these proteins in exosomes is cell type-specific. In this case, exosome formation may also be dependent on the cell/tissue context and external stimuli. Thus, our study suggested that *TP53* altered the composition of exosomes in CRC cells in a manner that was not mediated by the *TSAP6* and *CHMP4C* pathways.

Notably, we determined that the exosome size was significantly reduced in the MT and KO cells compared with the WT cells, which implies that *TP53* regulated the production of exosomes via a novel mechanism. To investigate this mechanism, we focused on shared differentially expressed proteins with consistent alterations in the MT vs. WT and KO vs. WT groups. Of the 31 proteins, HGS was selected for further analysis because of its roles in the endosomal pathway. HGS is a key component of the ESCRT-0 complex and is necessary for MVB sorting. HGS recognizes monoubiquitinated proteins and subsequently directs the cargos to the endosomal lumen and recruits the ESCRT-I complex^32^. Silencing *HGS* in HeLa cells has been reported to induce the generation of enlarged MVBs that frequently contained uniformly small ILVs with a diameter of less than 40 nm^33^. *HGS* silencing also reduced the secretion of vesicles that were 50–100 nm and 100–200 nm in diameter compared with the control cells^34^. In this example, ESCRT-independent and CD63-dependent ILV formations were upregulated^33^. CD63 is required for the ESCRT-independent sorting of some cargo to ILVs^12^,^33^. Moreover, previous studies have demonstrated that the size or formation of ESCRT-dependent ILVs may depend on the amount of ubiquitinated cargo^33^,^35^. It is possible that in the absence of HGS, ESCRT-dependent budding continues; however, the reduced amount of cargo leads to the formation of small ILVs^33^. We analyzed the proportion of ubiquitinated proteins in our differentially expressed proteins based on the annotation of post-translational modification in the Uniprot database (http://www.uniprot.org/). For the MT vs. WT groups, the ratios of ubiquitinated proteins were 32.1% (17/70) and 20.3% (16/79) in the down-regulated and up-regulated proteins, respectively (*P* = 0.5541). However, for the KO vs. WT groups, the ubiquitinated proteins were significantly enriched in the down-regulated proteins [22.6% (61/270) vs. 8.0% (79/224), respectively, *P* < 0.0001]. These findings suggest that the putative ubiquitinated protein cargo is decreased in MT and KO cells. Moreover, HGS depletion causes reduced exosome production, which parallels the decrease in hepatitis C virus (HCV) release in HCV-infected cells^36^. In the plasma of antiretroviral therapy-naive HIV-1-infected patients, the levels of some microRNA contents of exosomes, such as miR-155 and miR-223 but not miR-92, are strong inversely correlated with exosome abundance and size^37^. Therefore, the different ILV sizes indicate distinct generation machineries of ILV subpopulations and their cargoes.

Furthermore, our transmission electron microscopy and NanoSight analysis results demonstrated that the exosome diameters of the MT and KO cells are approximately half the diameters of the WT cells, and their exosome abundance is more than two times the abundance of the WT cells (Fig. 1D). Therefore, the total lipid content of the exosomes from the MT and KO cells remained unchanged compared with the WT cells. However, for individual exosomes, a recent study also demonstrated that HGS depletion causes accumulation of LDL-derived cholesterol in the endocytic pathway in a manner independent of the ESCRT complex^38^. An increased cholesterol content may aid membrane budding and induce small ILV formation^33^,^39^. In addition, ESCRT complexes have been demonstrated to induce lipid phase separation^40^. Thus, it appears that the types of lipid harbored in exosome changed along with the exosome size.

Here, we also discovered that HGS was consistently down-regulated in *TP53* (R273H) mutant and *TP53*-null cells. Furthermore, in clinical specimens of colon cancer, we also determined that the TP53 mutant was associated with low levels of HGS. The knockdown of *HGS* substantially reduced the exosome size, and the restoration of *HGS* expression recovered the exosome size. These findings indicated that *HGS* was a *TP53*-regulated gene that was responsible for the control of exosome size in CRC. HGS is not a known TP53 target gene. There is no significant correlation between the levels of TP53 and HGS mRNA in TP53-wild type clinical samples (R = 0.1509, *P* = 0.1172). It appears that wild-type TP53 regulates HGS expression in an indirect manner. Interestingly, the high levels of HGS mRNA exhibited decreased and increased tendencies of short-term death in the patients with TP53 mutant and wild-type, respectively (Fig. 4E,F). However, in all cases, HGS mRNA is an unfavorable prediction factor in colon cancer (Fig. 4G). These findings suggest that the modulation of HGS mRNA expression is a complex process that is driven by multiple factors in the human body. The regulatory mechanisms of HGS are under exploration. However, these results indicate that *TP53* regulated ESCRT-dependent ILV formation and exosome secretion by *HGS*, whereas *TP53* dysfunction may impair this major machinery and upregulate the alternative CD63-dependent pathway (Fig. 5E).

*HGS* is an essential gene for embryonic development and normal cellular homeostasis. Mice that carry homozygous null mutations of *HGS* exhibit embryonic lethality around day 11 (E11)^41^. The overexpression of HGS reduced EGFR and EGF-mediated STAT3 activation in rat Schwannoma cells^42^. The HGS protein was overexpressed in multiple tumors of different origins (stomach, colon, liver, cervix and melanoma) based on very limited clinical specimens. The silencing of *HGS* attenuated the proliferation, tumorigenesis, and metastatic potential of HeLa cells as a result of an upregulation of E-cadherin and a reduction in beta-catenin signalling^43^. Consistently, we demonstrated that the expression of HGS protein was gradually increased along with CRC tumorigenesis. Moreover, high levels of HGS protein were associated with worse tumor biological behavior, such as worse differentiation, a larger tumor size and poor prognosis. Therefore, HGS may participate in tumor progression via the regulation of the component of tumor microenvironment. Additional investigations are ongoing in our laboratory.

In conclusion, we investigated the influence of *TP53* dysfunction on tumor-derived exosomes in the CRC cell line HCT116. We identified a reduced size of the exosomes secreted from both *TP53* (R273H) mutant and *TP53*-null cells compared with *TP53* wild-type cells. The subsequent quantitative proteomic analysis of exosomal proteins using the iTRAQ-2D-LC-MS/MS strategy indicated that HGS, which is a key component of the ESCRT-0 complex, was down-regulated in the exosomes from the *TP53* (R273H) mutant and null cells. Further validation demonstrated that the *TP53* status regulated HGS expression, and HGS was necessary for the maintenance of larger-sized tumor-derived exosomes. It appears likely that tumor suppressor gene *TP53* remodelling of the tumor microenvironment promotes carcinogenesis via the ESCRT-dependent exosome secretion machinery. HGS may represent as a novel biomarker and target for therapeutic interventions in CRC.

## Materials and Methods

### Cell culture and construction of stable cell lines

The human colon cancer cell HCT116 was obtained from the American Type Culture Collection (Rockville, MD, USA). HCT116-*TP53*^(−/−)^ cells were kindly provided by Dr. Bert Vogelstein (John Hopkins University, Baltimore, MD, USA). The cells were cultured in RPMI 1640 medium supplemented with 10% fetal bovine serum (HyClone, Logan, UT, USA), 100 U/ml penicillin and 100 μg/ml streptomycin.

The pCMV6-Myc-DDK-AC expression plasmid that contained the *TP53* mutant (R273H) (Cat. No. RC400073) and the control plasmid were purchased from Origene (Rockville, MD, USA). The HCT116 cells were transfected with the *TP53* mutant and control vectors using Lipofectamine 2000 (Life Technologies, Carlsbad, CA, USA) according to the manufacturer’s instructions. After 24 hours, the cells were screened via the addition of G418 (Sigma-Aldrich, St. Louis, MO, USA). After selection for 14 days, the stable cell lines (mixed clones) were harvested for use.

To stably knockdown the hepatocyte growth factor-regulated tyrosine kinase substrate (HGS) gene in the HCT116 cells, the specific target sequence (5′-TGTACTCTTCACCTGTGAA-3′)^34^ was synthesized and incorporated into the lentivirus short hairpin (sh) RNA vector pLKO.1. After lentivirus packaging, HCT116 cells were infected and subsequently selected with puromycin.

To recover HGS expression in the HCT116-shHGS stable cells, the pCMV6-Myc-DDK-HGS expression plasmid (Cat. No. RC200609, Origene) was transfected using Lipofectamine 2000.

### Exosomes isolation

The conditioned media from the HCT116 (abbreviated WT), HCT116-*TP53*(R273H) (abbreviated MT) and HCT116-*TP53*^(−/−)^ (abbreviated KO) cells were centrifuged at 3000 g for 15 min at 4 °C to remove cellular debris. The supernatants were subsequently added to Amicon Ultra centrifugal filters with 3000 Daltons MWCO (Millipore, Bedford, MA) and concentrated at 4 °C, 4000 g to a final volume of 500 μL. The concentrated media were mixed with ExoQuick^TM^ Exosome Precipitation Solution (System Biosciences, San Francisco, CA) at a ratio of 1:1 (v/v) and incubated at 4 °C overnight. The next day, the mixtures were centrifuged at 4 °C, 1500 g for 30 min, and the precipitates were re-suspended for further use.

For the rescue experiment, after 24 hours of transfection with pCMV6-Myc-DDK-HGS vector, the HCT116-shHGS cells were washed with PBS three times and subsequently cultured in serum-free medium for an additional 24 hours for exosome isolation.

### Transmission electron microscopy of exosomes

Extracted exosomes were re-suspended in PBS, mounted on copper-mesh formvar grids, and negatively stained using 2% phosphotungstic acid for 10 min. Following the removal of the excess fluid, the grid was air-dried and subsequently assessed using a CM120 transmission electron microscope (Philips, Netherland) operated at 80 kV.

### Size distribution analysis of exosomal particles

The average diameter of the exosomes was measured as previously described^27^. Briefly, 200 exosomes from transmission electron microscopy images of two independent exosome preparations (a total of 200) were determined using the Image J software.

Real-time high-resolution particle detection and sizing were performed on a NanoSight NS500 following the manufacturer’s protocols (Malvern Instruments, Malvern, UK). The Nanoparticle Tracking Analysis system was used to compare the changes in the exosome sizes and abundance in different cell lines.

### Protein extraction

The cells and isolated exosomes were lysed in RIPA buffer [25 mM Tris–HCl (pH 7.5), 150 mM NaCl, 1% NP-40, 1% sodium deoxycholate, and 0.1% SDS] with protease inhibitor cocktail (Roche Applied Science, Germany) on ice for 30 min with occasional shaking. The insoluble components were removed via centrifugation at 12,000 g for 15 minutes at 4 °C. The protein concentration was measured using the RC DC Protein Assay (Bio-Rad, Hercules, CA).

### Proteomic measurements

#### Sample preparation

The exosome proteins were digested via filter-aided sample preparation combined with a microwave-assisted protein preparation method as previously described^44^,^45^. The peptides were dried via vacuum centrifugation and stored at −80 °C.

The digested samples were labelled with iTRAQ technology according to the manufacturer’s protocol (AB Sciex, Framingham, MA), and the samples were then equally pooled for the following analysis.

#### First dimensional high-pH RPLC separation

The pooled mixture of the labelled samples was fractionated using a high-pH RPLC column from Waters (4.6 mm × 250 mm, C18, 3 μm). The samples were loaded onto the column in buffer A1 (0.1% aqueous ammonia in water, pH = 10) and eluted by buffer B1 (0.1% aqueous ammonia in 10% water and 90% ACN; pH = 10, flow rate = 1 mL/min) with a gradient of 5–90% for 60 min. The eluted peptides were collected in 60 fractions and pooled in 20 samples.

#### LC-MS/MS analysis

Each sample was split into two portions and subsequently analyzed on an RP C18 self-packing capillary LC column (75 μm × 100 mm, 3 μm). A Triple TOF 5600 mass spectrometer (AB Sciex) was used to analyze the samples. The elution gradient lasted for 100 min. An elution gradient of 5–30% buffer B2 (0.1% formic acid, 99.9% ACN; flow rate, 0.3 μL/min) was used for the analysis. The MS data were acquired in high sensitivity mode using the following parameters. Thirty data-dependent MS/MS scans were acquired for every full scan. The rolling collision energy was used, and charge state screening (including precursors with a +2 to +4 charge state) and dynamic exclusion (exclusion duration of 15 s) were performed.

#### Database search

The MS/MS spectra were searched against the human subset of the Uniprot database (84,910 entries) (http://www.uniprot.org/) using Mascot software version 2.3.02 (Matrix Science, UK). Trypsin was chosen for cleavage with a maximum number of two allowed missed cleavages. Carbamidomethylation (C) and iTRAQ 4-plex labels were set as fixed modifications. The searches were performed using a peptide and product ion tolerance of 0.05 Da. Scaffold software was used to further filter the database search results using the decoy database method with the following filter: a 1% false-positive rate at the protein level and two unique peptides per protein. After filtering the results as previously described, the peptide abundances in the different reporter ion channels of the MS/MS scan were normalized. The protein abundance ratio was based on unique peptide results. Proteins with a fold change ≥1.6 were considered significantly altered.

### Western blot analysis

Total cell lysates (20 μg) or exosome lysates (15 μg) were separated on 10% SDS-PAGE and transferred to a polyvinylidene difluoride membrane (Millipore). After blocking, the membranes were blotted with the following antibodies: anti-HSP70 (Cat. No. sc33575), anti-CD63 (Cat. No. sc15363), anti-NPM1 (Cat. No. sc4772) (Santa Cruz, Dallas, TX), anti-BNIP3 (Cat. No. ab10433), anti-CD9 (Cat. No. ab92726) (Abcam, Cambridge, UK), anti-β-actin (Cat. No. #4970), anti-CCNB1 (Cat. No. #4135), anti-MRE11 (Cat. No. #4895S), anti-HMGB1 (Cat. No #3935), anti-RhoA (Cat. No. #2177) (Cell Signaling Technology, Danvers, MA, USA) at a dilution of 1:1000, as well as anti-HGS (Cat. No. WH0009146), anti-KHSRP (Cat. No. HPA034739) and anti-PES1 (Cat. No. HPA062439) (Sigma-Aldrich) at a dilution of 1:500. Following intensive washing, the membranes were developed with anti-mouse or rabbit horseradish peroxidase conjugated secondary antibodies (Cell Signaling Technology) at a dilution of 1:5000. The protein bands were visualized using Super Signal West Femto Maximum Sensitivity Substrate (Thermo Fisher Scientific, Waltham, MA, USA) with the ImageQuant LAS4000 system (Fujifilm, Tokyo, Japan).

### Cell proliferation assay

Cell counting kit-8 (CCK-8) (Dojindo, japan) was used to determine the growth of the HCT116 mock control and HCT116-p53 (R273H) cells. Three thousand cells were plated into 96-well plates and subsequently incubated with 10 μL of CCK-8 reagent in the culture medium for 2 h. The absorbance values were detected at 450 nm using a Model 680 microplate reader (Bio-Rad) on days 1, 2, 3, 4, and 5.

### Wound healing assay

Cells were grown to confluence in a 6-well plate. Artificial wound tracks were created by scraping confluent cell monolayers with a pipette tip. Following removal of the detached cells by gently washing with PBS, the cells were incubated with serum-free medium to enable the cells to migrate into the open area. The ability of the cells to migrate into the wound area was assessed at 0 and 24 h after scratching in micrographs of at least three randomly selected wounded areas. The experiments were independently performed three times.

### Quantitative real-time reverse transcriptase-polymerase chain reaction (qRT-PCR)

Total RNA was extracted with TRIzol reagent (Life Technologies) in HCT116, HCT116-p53(R273H) and HCT116-p53^(−/−)^ cells, and complementary DNA was generated using the SuperScript™III First-Strand Synthesis System (Life Technologies). The qRT-PCR assay was performed using a SYBR Premix Dimer Eraser (Perfect Real Time) kit (Takara Bio, Shiga, Japan) on a CFX96 Touch™ Real-Time PCR Detection System (Bio-Rad). The β-actin gene (ACTB) was used as an endogenous control. Each experiment was performed in triplicate. The gene-specific primers for the genes were as follows: HGS: forward 5′-ACTGGAGGGAAAAGGCGGAAG-3′, reverse 5′-GGACTCCCAATCTGTCTCCAAC-3′; ACTB: forward 5′-GGCACCCAGCACAATGAAG-3′, reverse 5′-GCCGATCCACACGGAGTACT-3′. The relative expression was calculated using the ratio of the value of the gene to b-actin and subsequently the controls.

### Immunohistochemistry staining

Formalin-fixed, paraffin-embedded four tissue microarrays that contained 4 normal colorectal mucosa, 11 tubular adenoma and 235 pairs of malignant tumors and their matched adjacent normal colorectal tissues were purchased from Shanghai Outdo Biotech Co., Ltd (Shanghai, China). The samples were stained with anti-HGS antibody (Cat. No. ab56468, Abcam, Cambridge, UK), and images were captured using Aperio ScanScope CS software (Vista, CA, USA). The results were independently evaluated by two independent pathologists. The HGS staining intensity and area were quantified as previously described^46^. A staining index was used, in which < = 3 was considered negative and > = 4 was considered positive expression.

### TCGA RNA sequencing data mining, bioinformatic and statistical analyses

The publically available colon cancer RNA-Sequencing dataset was downloaded from TCGA. The normalized transcript (isoform) expression data from 459 patients were used. Mann-Whitney U tests were used to compare the RPKM (Reads per kilobase of transcript per million reads mapped) between two groups.

Gene Ontology (GO) annotation and novel exosomal protein analysis were performed using the FunRich tool (http://funrich.org/). To assess the differences among the different groups, Kruskal-Wallis one-way analysis of variance or Mann-Whitney rank test were applied. The Kaplan-Meier method and log-rank analysis were performed to compare the survival curves. Univariate and multivariate analyses were performed using the Cox regression model. *P*-values < 0.05 were considered statistically significant. All analyses were performed using SPSS, version 17.0 (IBM software).

## Additional Information

**How to cite this article**: Sun, Y. *et al.* A novel *TP53* pathway influences the *HGS*-mediated exosome formation in colorectal cancer. *Sci. Rep.* **6**, 28083; doi: 10.1038/srep28083 (2016).

## Supplementary Material

Supplementary Information

## Figures and Tables

**Table 1:** Univariate and multivariate survival analyses of HGS expression for overall survival in 235 patients with CRC.

	Relative risk (95% CI)	*P* value
Univariate		
Age (≥70 vs. <70 years old)	1.81 (0.99–3.33)	0.0549
Gender (female vs. male)	1.18 (0.89–1.58)	0.2545
Differentiation (Poorly vs. moderately vs. well)	1.38 (0.87–2.18)	0.1731
Tumor size (≥6 vs. <6 cm)	1.66 (0.93–2.94)	0.0850
Vascular invasion (yes vs. no)	2.27 (1.09–4.75)	0.0291
Tumor invasive depth (T4 vs. T3 vs. T1 + T2)	1.62 (1.12–2.33)	0.0095
Lymph node metastasis (N1b,c+N2 vs. N0 + N1a)	2.41 (1.32–4.40)	0.0042
Distant metastasis (M1 vs. M0)	10.19 (2.45–42.37)	0.0014
TNM staging (IV vs. III vs. II vs. I)	2.17 (1.34–3.50)	0.0016
HGS expression (positive vs. negative)	2.27 (1.06–4.86)	0.0353
Multivariate		
Vascular invasion (yes vs. no)	5.50 (1.73–239.0)	0.0167
TNM staging (III and IV vs. I and II)	2.37 (1.32–4.25)	0.0006
HGS expression (positive vs. negative)	3.34 (1.19–9.41)	0.0221
