# Supplementary material for: A novel TP53 pathway influences the HGS-mediated exosome formation in colorectal cancer
Source: Sci Rep. 2016 Jun 17;6:28083. doi: 10.1038/srep28083 (PMC4911576; doi:10.1038/srep28083)
Supplement: Supplementary Information [file srep28083-s1.pdf]

Supplementary Information

A novel TP53 pathway influences the HGS-mediated exosome formation in colorectal cancer

Yulin Sun[1, §], Weiwei Zheng[2, §], Zhengguang Guo[3, §], Qiang Ju[1], Lin Zhu[4], Jiajia Gao[1], Lanping Zhou[1], Fang Liu[1], Yang Xu[1], Qimin Zhan[1], Zhixiang Zhou[5,*], Wei Sun[3,*], Xiaohang Zhao[1,*]

[1] State Key Laboratory of Molecular Oncology, National Cancer Center/Cancer Hospital, Chinese Academy of Medical Sciences & Peking Union Medical College, Beijing 100021, China

[2] Center of Basic Medical Science, Navy General Hospital, Beijing 100048, China

[3] Core Facility of Instruments, Institute of Basic Medical Sciences, Chinese Academy of Medical Sciences & Peking Union Medical College, Beijing 100005, China

[4] Department of Clinical Biochemistry, Chinese PLA General Hosptital, Beijing 100853, China

[5] Department of Colorectal Surgery, National Cancer Center/Cancer Hospital, Chinese Academy of Medical Sciences & Peking Union Medical College, Beijing 100021, China

Supplementary Figure S1. TP53 R273H mutant promotes cell proliferation and migration in HCT116 cells.

(A) Growth curves of HCT116 *TP53* (R273H) and HCT116 mock control cells. The relative cell numbers are shown as the mean ± SD for each day. ***, *P*<0.0001. (B) Representative images (left panel) and calculated average wound healing rate (right panel) of the wound healing assay in HCT116 *TP53* (R273H) and HCT116 mock control cells. ***, *P*<0.0001. (C) Western blot analysis of TP53 in three stable transfectants. The expression of TP53 in the HCT116*TP53* (R273H) cells was significantly upregulated compared with the empty vector control, whereas TP53 was not expressed in the HCT116-*TP53*(-/-) cells. β-actin was used as a loading control.

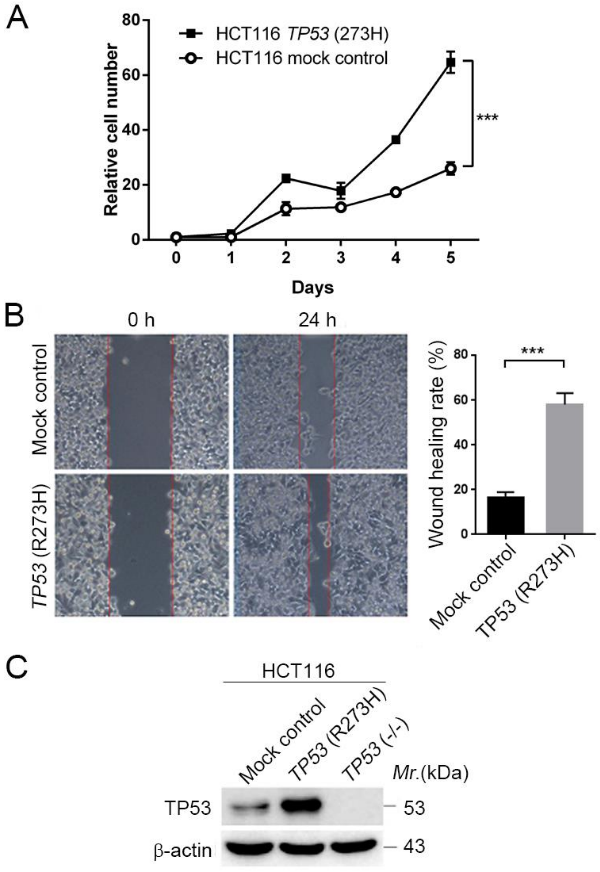

Supplementary Figure S2. Quantitative proteomic analysis strategy of exosomal proteins by iTRAQ-2D-LC-MS/MS.

(A) Experimental workflow of quantitative iTRAQ-2D-LC-MS/MSanalysis. (B) The quantitative results of the proteins derived from the technical replicates. The square of the Pearson correlation coefficient was 0.86. (C) The quantitative results of proteins derived from the biological replicates. The square of the Pearson correlation coefficient was 0.87.

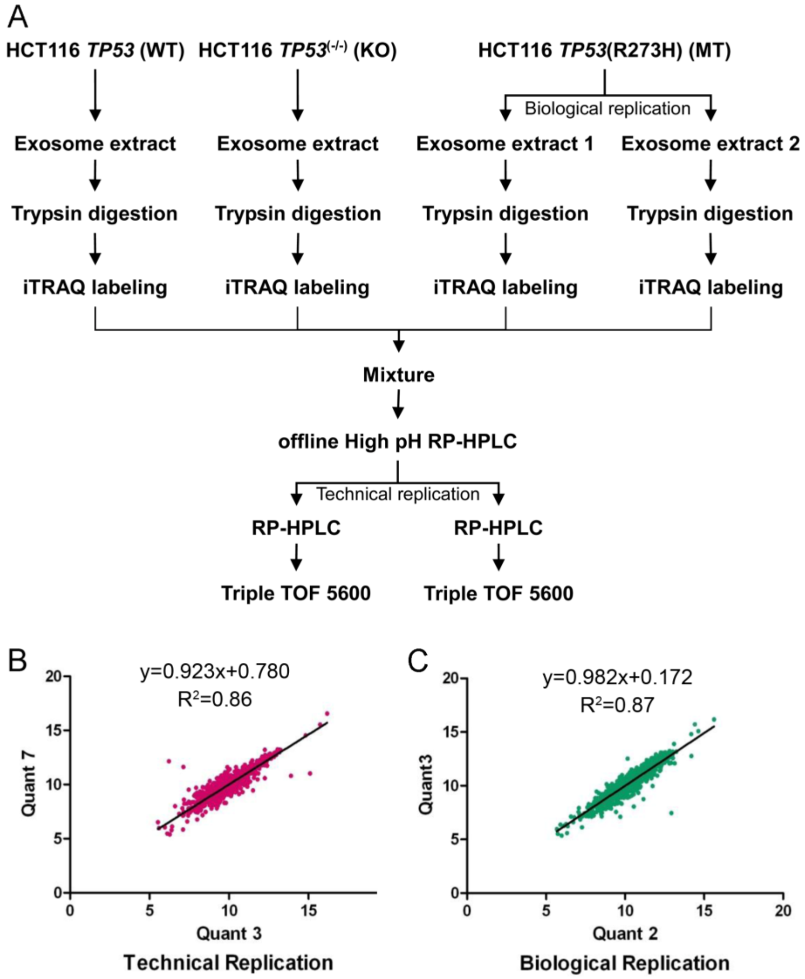

Supplementary Figure S3. Representative Coomassie Blue stained SDS-PAGE gel of exosomal proteins from WT, MT and KO cells.

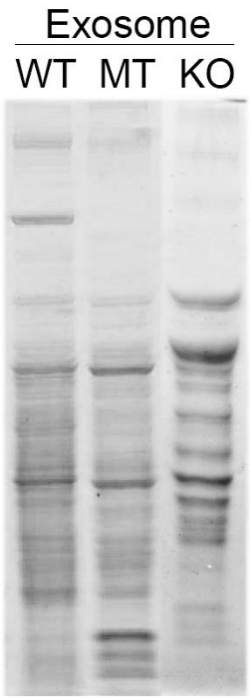

Supplementary Figure S4. Knockdown of HGS using specific siRNA leads to smaller exosomes.

(A) Representative electron micrographs of exosomes isolated from the serum-free conditional medium collected from HCT116 cells transfected with two HGS-targeting siRNA (siHGS-1 and siHGS-2) and negative control siRNA. Scale bar, 100 nm; Direct magnification: 120,000 ×. (B) The mean diameter distribution of 200 observed exosomes was calculated from electron micrograph images, and the exosome frequency was plotted for indicated size. The mean diameter for the exosomes from negative control, siHGS-1 and siHGS-2 was 57.04 nm, 37.18 nm and 36.23 nm, respectively. (C) Statistical analysis of size distribution for exosomes derived from each group. n= 200 exosomes per group; ***P<0.0001.

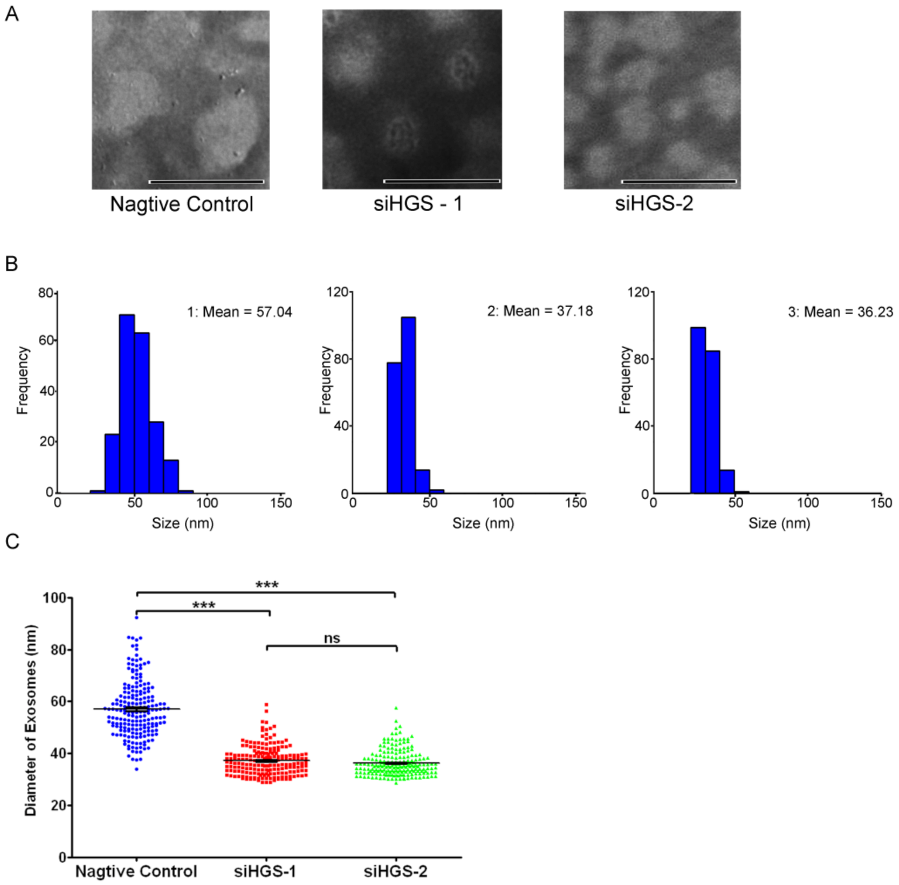

Supplementary Table S1. The identified 3437 protein list by iTRAQ-2D-LC-MS/MS strategy.

Accession Number	Protein Name	Gene Symbol	Molecular Weight	WT-rep 1	WT-rep 2	MT-bio 1-rep 1	MT-bio 1-rep 2	MT-bio 2-rep 1	MT-bio 2-rep 2	KO-rep 1	KO-rep 2	SP	TM
P07900	Cluster of Heat shock protein HSP 90-alpha	HSP90AA1	85 kDa	1	1	1.8	1.8	1.5	1.3	8.8	7.7	0	0
P14618	Cluster of Isoform 3 of Pyruvate kinase isozymes M1/M2	PKM	56 kDa	1	1	0.1	0.1	0.1	0.1	0.1	0.1	0	0
P07195	Cluster of L-lactate dehydrogenase B chain	LDHB	37 kDa	1	1	0.9	0.7	0.6	0.5	5.1	4.7	0	0
O43707	Cluster of Alpha-actinin-4	ACTN4	105 kDa	1	1	1.2	1.2	1	1.1	1.2	1.2	0	0
P11021	78 kDa glucose-regulated protein	HSPA5	72 kDa	1	1	1	1	1.3	1.2	0.7	0.7	3	0
P04406	Glyceraldehyde-3-phosphate dehydrogenase	GAPDH	36 kDa	1	1	1.1	1.1	0.9	0.9	0.7	0.7	0	0
Q15149	Cluster of Isoform 4 of Plectin	PLEC	516 kDa	1	1	0.9	0.9	0.5	0.7	1.7	1.6	0	0
P15311	Cluster of Ezrin	EZR	69 kDa	1	1	1.3	1.3	1.3	1.3	0.9	0.9	0	0
P06733	Cluster of Alpha-enolase	ENO1	47 kDa	1	1	0.7	0.7	1.1	1	1.7	1.6	0	0
P02545	Cluster of Prelamin-A/C	LMNA	74 kDa	1	1.1	1.7	1.7	1.8	1.8	2.2	2.5	0	0
P13639	Elongation factor 2	EEF2	95 kDa	1	1	0.8	0.8	1.2	1.1	0.3	0.2	0	0
P35579	Cluster of Myosin-9	MYH9	227 kDa	1	1	0.7	0.7	0.9	0.9	0.3	0.2	0	0
P08107	Cluster of Heat shock 70 kDa protein 1A/1B	HSPA1A	70 kDa	1	1	0.7	0.7	0.8	0.7	0.3	0.2	0	0
P11142	Cluster of Heat shock cognate 71 kDa protein	HSPA8	71 kDa	1	1	0.5	0.4	0.8	0.7	0.3	0.2	0	0
F8VXB4	Cluster of Keratin, type II cytoskeletal 8	KRT8	57 kDa	1	1	1	1.2	1.6	1.8	0.2	0.2	0	0
P04075	Cluster of Fructose-bisphosphate aldolase A	ALDOA	39 kDa	1	1	1.7	1.6	1.7	1.7	0.2	0.2	1	0
P07355	Cluster of Isoform 2 of Annexin A2	ANXA2	40 kDa	1	1	1	0.9	1.6	1.5	0.2	0.2	0	0
I3L3I4	Cluster of Actin, cytoplasmic 2	ACTG1	42 kDa	1	1	0.9	0.6	0.9	0.9	0.2	0.2	0	0
Q08380	Galectin-3-binding protein	LGALS3BP	65 kDa	0.9	1	0.9	1	0.9	0.9	0.6	0.6	1	0
P11216	Cluster of Glycogen phosphorylase, brain form	PYGB	97 kDa	1	1	0.7	0.6	0.9	0.9	0.2	0.2	0	0
E7EPA7	Transketolase	TKT	69 kDa	1	1	0.7	0.7	0.8	0.7	0.2	0.2	0	0
P49327	Fatty acid synthase	FASN	273 kDa	1	1	0.4	0.5	0.4	0.4	0.2	0.2	0	0
Q00610	Cluster of Clathrin heavy chain 1	CLTC	192 kDa	1	1	1.1	1.2	1.1	1	5.8	5.8	0	0
P19338	Nucleolin	NCL	77 kDa	1	1	0.7	0.7	0.8	0.8	1.3	1.2	0	0
P18206	Cluster of Vinculin	VCL	124 kDa	1	1	0.7	0.8	1.1	0.8	0.5	0.3	0	0
O75369	Cluster of Isoform 8 of Filamin-B	FLNB	282 kDa	1	1	0.8	0.8	1	0.7	0.5	0.3	0	0
P06748	Cluster of Nucleophosmin	NPM1	33 kDa	1	1	1.4	1.3	2.1	2.1	0.4	0.3	0	0
Q13509	Cluster of Tubulin beta-3 chain	TUBB3	50 kDa	1	1	0.8	0.9	1.1	1.2	0.4	0.3	0	1
Q13813	Cluster of Spectrin alpha chain, non-erythrocytic 1	SPTAN1	285 kDa	1	1	1	0.9	0.9	1	0.4	0.3	0	0
P34932	Heat shock 70 kDa protein 4	HSPA4	94 kDa	1	1	0.7	0.6	0.9	0.9	0.4	0.3	0	0
P55072	Transitional endoplasmic reticulum ATPase	VCP	89 kDa	1	1.1	1.2	1.2	1.1	1.1	1.3	1.3	0	0

Q9Y490	Cluster of Talin-1	TLN1	270 kDa	1	1	0.7	0.6	0.9	0.9	0.4	0.3	0	0
P01023	Cluster of Alpha-2-macroglobulin	A2M	163 kDa	1	1	0.9	0.7	1	0.8	0.4	0.3	1	0
P08727	Cluster of Keratin, type I cytoskeletal 19	KRT19	44 kDa	1	1	0.7	0.7	0.9	0.8	0.4	0.3	0	0
Q8NBP7	Cluster of Proprotein convertase subtilisin/kexin type 9	PCSK9	74 kDa	1	1	0.9	0.6	1	0.7	0.4	0.3	1	0
P10809	Cluster of 60 kDa heat shock protein, mitochondrial	HSPD1	61 kDa	1	1	0.8	0.8	0.8	0.7	0.4	0.3	0	0
P12956	X-ray repair cross-complementing protein 6	XRCC6	70 kDa	1	1	1.1	0.9	1.4	1.6	3.5	4.9	0	0
P22314	Cluster of Ubiquitin-like modifier-activating enzyme 1	UBA1	118 kDa	1	1	0.7	0.7	0.8	0.7	0.4	0.3	0	1
D6RGG3	Cluster of Collagen alpha-1(XII) chain	COL12A1	333 kDa	1	1	0.7	0.8	0.6	0.7	0.4	0.3	1	0
Q02790	Cluster of Peptidyl-prolyl cis-trans isomerase FKBP4	FKBP4	52 kDa	1	1	0.7	0.6	0.6	0.6	0.4	0.3	0	0
Q16531	Cluster of DNA damage-binding protein 1	DDB1	127 kDa	1	1	0.6	0.4	0.5	0.6	0.4	0.3	1	0
O94985	Isoform 2 of Calsyntenin-1	CLSTN1	109 kDa	1	1	0.9	0.9	2.1	2.2	0.3	0.3	0	1
Q86VP6	Cluster of Cullin-associated NEDD8-dissociated protein 1	CAND1	136 kDa	1	1	1.8	1.9	1.8	1.8	0.3	0.3	0	0
G3XAD8	Cluster of Stress-induced-phosphoprotein 1	STIP1	68 kDa	1	1	0.7	0.8	1.2	1.5	0.3	0.3	0	0
O75643	U5 small nuclear ribonucleoprotein 200 kDa helicase	SNRNP200	245 kDa	1	1	1	1	1.5	1.4	0.3	0.3	0	0
P21333	Cluster of Filamin-A	FLNA	281 kDa	1	1	1	1	1.4	1.4	0.3	0.3	0	0
P53396	Cluster of ATP-citrate synthase	ACLY	121 kDa	1	1	0.8	0.8	1.4	1.4	0.3	0.3	0	0
Q14980	Cluster of Isoform 2 of Nuclear mitotic apparatus protein 1	NUMA1	237 kDa	1	1	0.9	0.9	1.3	1.4	0.3	0.3	0	0
P38919	Cluster of Eukaryotic initiation factor 4A-III	EIF4A3	47 kDa	1	1	0.9	0.7	1.2	1.3	0.3	0.3	0	0
P62805	Histone H4	HIST1H4A	11 kDa	1	1	1.4	1.3	1.1	1.1	0.6	0.6	0	0
B1AK87	Cluster of Capping protein (Actin filament) muscle Z-line, b	CAPZB	29 kDa	1	1	1	0.9	1.2	1.1	0.3	0.3	0	0
P17655	Cluster of Calpain-2 catalytic subunit	CAPN2	80 kDa	1	1	1	1	1	1.1	0.3	0.3	0	0
Q92598	Cluster of Heat shock protein 105 kDa	HSPH1	97 kDa	1	1	0.9	0.7	0.9	1.1	0.3	0.3	0	0
P07951	Cluster of Isoform 3 of Tropomyosin beta chain	TPM2	29 kDa	1	1	0.9	1	1.1	1	0.3	0.3	0	0
P46940	Cluster of Ras GTPase-activating-like protein IQGAP1	IQGAP1	189 kDa	1	1	1.2	1.1	1	1	0.3	0.3	0	0
P98160	Cluster of Basement membrane-specific heparan sulfate pro	HSPG2	469 kDa	1	1	0.9	0.9	1	1	0.3	0.3	1	0
P14625	Cluster of Endoplasmin	HSP90B1	92 kDa	1	1	0.7	0.7	1	1	0.3	0.3	1	0
P78371	T-complex protein 1 subunit beta	CCT2	57 kDa	1	1	0.7	0.7	1	1	0.3	0.3	0	0
Q01082	Cluster of Spectrin beta chain, non-erythrocytic 1	SPTBN1	275 kDa	1	1	0.8	0.7	0.9	1	0.3	0.3	0	0
P12270	Cluster of Nucleoprotein TPR	TPR	267 kDa	1	1	0.7	0.6	0.9	1	0.3	0.3	0	0
P49368	Cluster of T-complex protein 1 subunit gamma	CCT3	61 kDa	1	1	0.9	1	1.1	0.9	0.3	0.3	0	0
Q14204	Cytoplasmic dynein 1 heavy chain 1	DYNC1H1	532 kDa	1	1	0.8	1.3	0.9	0.9	0.3	0.3	0	0
P04083	Annexin A1	ANXA1	39 kDa	1	1	0.7	0.8	0.9	0.9	0.3	0.3	0	0
Q99988	Growth/differentiation factor 15	GDF15	34 kDa	1	1	0.7	0.8	0.9	0.9	0.3	0.3	1	0
B4DR87	Cluster of Procollagen-lysine,2-oxoglutarate 5-dioxygenase	PLOD1	88 kDa	1	1	0.7	0.7	0.9	0.9	0.3	0.3	1	0

P00558	Cluster of Phosphoglycerate kinase 1	PGK1	45 kDa	1	1	0.7	0.6	0.9	0.9	0.3	0.3	0	0
Q14697	Cluster of Neutral alpha-glucosidase AB	GANAB	107 kDa	1	1	0.7	0.6	0.9	0.9	0.3	0.3	1	0
Q14517	Cluster of Protocadherin Fat 1	FAT1	506 kDa	1	1	0.7	0.6	0.9	0.8	0.3	0.3	1	1
H7C4C8	Cluster of T-complex protein 1 subunit theta (Fragment)	CCT8	35 kDa	No Va	1	No Value	1.6	No Value	0.6	No Val	1.6	0	0
P10586	Cluster of Receptor-type tyrosine-protein phosphatase F	PTPRF	213 kDa	1	1	0.6	0.5	0.9	0.8	0.3	0.3	1	1
P68363	Cluster of Tubulin alpha-1B chain	TUBA1B	50 kDa	1	1	0.5	0.6	0.8	0.8	0.3	0.3	0	0
P09382	Galectin-1	LGALS1	15 kDa	1	1	0.5	0.7	0.6	0.8	0.3	0.3	0	0
Q10567	Cluster of Isoform C of AP-1 complex subunit beta-1	AP1B1	104 kDa	1	1	0.6	0.6	0.8	0.7	0.3	0.3	0	0
E9PNZ4	Cluster of Microtubule-actin cross-linking factor 1, isoform	MACF1	231 kDa	No Va	No Va	No Value	No Value	No Value	No Value	No Val	No Val	0	1
P07602	Isoform Sap-mu-9 of Proactivator polypeptide	PSAP	58 kDa	1	1	0.8	0.9	1.1	1	0.1	0.1	1	0
Q92896	Cluster of Isoform 3 of Golgi apparatus protein 1	GLG1	136 kDa	1	1	0.9	0.8	0.7	0.7	0.3	0.3	1	1
O60568	Procollagen-lysine,2-oxoglutarate 5-dioxygenase 3	PLOD3	85 kDa	1	1	0.7	0.6	0.7	0.7	0.3	0.3	1	0
Q99832	Cluster of T-complex protein 1 subunit eta	CCT7	59 kDa	1	1	0.7	0.7	0.8	0.6	0.3	0.3	0	0
O15230	Laminin subunit alpha-5	LAMA5	400 kDa	1	1	0.6	0.6	0.5	0.5	0.3	0.3	1	0
P26639	Cluster of Threonine--tRNA ligase, cytoplasmic	TARS	83 kDa	1	1	0.7	0.5	0.5	0.4	0.3	0.3	0	0
P15531	Cluster of Nucleoside diphosphate kinase A	NME1	17 kDa	1	1	0.4	0.5	0.4	0.4	0.3	0.3	1	0
O60506	Cluster of Isoform 3 of Heterogeneous nuclear ribonucleopr	SYNCRIP	63 kDa	1	1	1.3	1.2	1.7	1.6	0.2	0.3	0	0
P49589	Cluster of Isoform 3 of Cysteine--tRNA ligase, cytoplasmic	CARS	95 kDa	1	1	1	1	1.1	1.2	0.2	0.3	0	0
P60174	Cluster of Isoform 2 of Triosephosphate isomerase	TPI1	27 kDa	No Va	No Va	No Value	No Value	No Value	No Value	No Val	No Val	0	0
Q99879	Cluster of Histone H2B type 1-M	HIST1H2BM	14 kDa	1	1	0.9	0.9	1.3	1.1	0.2	0.3	0	0
P12109	Collagen alpha-1(VI) chain	COL6A1	109 kDa	1	1	1.2	1.2	1.1	1.1	0.2	0.3	3	0
G5EA52	Cluster of Protein disulfide isomerase family A, member 3,	PDIA3	55 kDa	1	1	0.7	0.7	1	1.1	0.2	0.3	1	0
P06744	Glucose-6-phosphate isomerase	GPI	63 kDa	1	1	0.9	0.9	1	1	0.2	0.3	0	1
P12081	Cluster of Histidine--tRNA ligase, cytoplasmic	HARS	57 kDa	1	1	0.7	0.7	1	1	0.2	0.3	0	0
F5H8J2	Cluster of Protein disulfide-isomerase	P4HB	51 kDa	1	1	0.5	0.6	0.9	1	0.2	0.3	1	0
P11047	Laminin subunit gamma-1	LAMC1	178 kDa	1	1	0.6	0.5	0.9	0.8	0.2	0.3	1	0
P09429	Cluster of High mobility group protein B1	HMGB1	25 kDa	1	1	0.7	0.7	0.6	0.7	0.2	0.3	0	0
P13667	Cluster of Protein disulfide-isomerase A4	PDIA4	73 kDa	1	1	0.5	0.6	0.4	0.6	0.2	0.3	1	0
Q13753	Laminin subunit gamma-2	LAMC2	131 kDa	1	1	1.5	1.5	1.8	1.8	0.9	0.9	1	0
Q08211	ATP-dependent RNA helicase A	DHX9	141 kDa	1	1	1	0.9	0.9	0.9	1.4	1.3	0	0
P50991	T-complex protein 1 subunit delta	CCT4	58 kDa	1	1	1	1	1.1	1.1	0.8	0.8	0	0
E7ET40	Cluster of Urokinase-type plasminogen activator short chair	PLAU	47 kDa	1	1	3	2.7	3.2	2.9	0.3	0.4	1	0
Q8WUM4	Cluster of Programmed cell death 6-interacting protein	PDCD6IP	96 kDa	1	1	1.1	1.1	1.1	1	0.7	0.7	0	0
P10909	Cluster of Isoform 2 of Clusterin	CLU	58 kDa	1	1	0.9	1	0.9	0.9	1.1	1.1	1	0

P23284	Peptidyl-prolyl cis-trans isomerase B	PPIB	24 kDa	1	1	1	1.2	0.8	1.3	0.6	0.4	2	0
P20700	Lamin-B1	LMNB1	66 kDa	1	1	1	0.9	1.2	1	0.6	0.4	0	0
O00391	Cluster of Sulfhydryl oxidase 1	QSOX1	83 kDa	1	1	1.1	0.8	1.1	0.9	0.6	0.4	1	1
P02786	Cluster of Transferrin receptor protein 1	TFRC	85 kDa	1	1	0.9	0.8	0.9	0.8	0.6	0.4	0	1
Q09666	Cluster of Neuroblast differentiation-associated protein AH	AHNAK	629 kDa	1	1	0.9	0.8	0.9	0.8	0.6	0.4	0	0
P13010	X-ray repair cross-complementing protein 5	XRCC5	83 kDa	1	1	0.5	0.6	1	0.7	0.6	0.4	0	0
Q14152	Eukaryotic translation initiation factor 3 subunit A	EIF3A	167 kDa	1	1	0.7	0.8	1.3	1	0.5	0.4	0	0
E9PCA1	T-complex protein 1 subunit epsilon	CCT5	57 kDa	1	1	0.8	1.1	0.9	1	0.5	0.4	0	0
Q14315	Filamin-C	FLNC	291 kDa	1	1	1	0.9	0.7	1	0.5	0.4	0	0
P09972	Cluster of Fructose-bisphosphate aldolase C	ALDOC	39 kDa	1	1	0.8	1	1.2	0.9	0.5	0.4	0	0
H3BM09	Cluster of HLA class I histocompatibility antigen, A-68 alp	HLA-A	34 kDa	1	1	1	0.9	1	0.9	0.5	0.4	1	1
P07814	Bifunctional glutamate/proline--tRNA ligase	EPRS	171 kDa	1	0.9	0.9	0.9	1	0.9	0.5	0.4	0	0
Q14974	Cluster of Importin subunit beta-1	KPNB1	97 kDa	1	1	0.9	0.8	1	0.9	0.5	0.4	0	0
Q9UQ80	Cluster of Proliferation-associated protein 2G4	PA2G4	44 kDa	1	1	0.8	0.7	0.9	0.9	0.5	0.4	0	0
F5H3A1	Cluster of Sodium/potassium-transporting ATPase subunit a	ATP1A1	113 kDa	1	1	0.7	0.6	0.9	0.8	0.5	0.4	0	1
P02768	Cluster of Serum albumin	ALB	69 kDa	1	1	0.8	0.6	0.8	0.8	0.5	0.4	1	0
Q15393	Splicing factor 3B subunit 3	SF3B3	136 kDa	1	1	0.9	0.9	0.9	0.9	1.2	1.2	0	0
Q6YHK3	CD109 antigen	CD109	162 kDa	1	1	0.8	0.9	0.9	0.7	0.5	0.4	0	1
P55060	Exportin-2	CSE1L	110 kDa	1	1	0.8	0.7	0.8	0.7	0.5	0.4	0	0
P55786	Cluster of Puromycin-sensitive aminopeptidase	NPEPPS	103 kDa	1	1	0.9	0.6	1	0.6	0.5	0.4	0	1
P17987	Cluster of T-complex protein 1 subunit alpha	TCP1	60 kDa	1	1	0.8	0.5	0.6	0.6	0.5	0.4	0	0
G3XAI2	Cluster of Laminin subunit beta-1	LAMB1	200 kDa	1	1	1	0.7	0.5	0.5	0.5	0.4	1	0
O14672	Disintegrin and metalloproteinase domain-containing protei	ADAM10	84 kDa	1	1	1	1.1	1.1	1.2	0.7	0.8	1	1
Q01518	Cluster of Adenylyl cyclase-associated protein 1	CAP1	52 kDa	1	1	1.8	1.6	1.7	1.6	0.4	0.4	0	0
B5ME19	Eukaryotic translation initiation factor 3 subunit C	EIF3CL	105 kDa	1	1	1	0.7	1.6	1.3	0.4	0.4	0	0
E9PCI3	Asparagine synthetase	ASNS	62 kDa	1	1	1.3	1.4	1.3	1.3	0.4	0.4	0	0
P01024	Complement C3	C3	187 kDa	1	1	1.2	1.1	1.3	1.2	0.4	0.4	1	0
P11717	Cation-independent mannose-6-phosphate receptor	IGF2R	274 kDa	1	1	1.1	1	1.2	1.2	0.4	0.4	0	1
P09874	Poly [ADP-ribose] polymerase 1	PARP1	113 kDa	1	1	0.9	0.9	1.1	1.1	0.4	0.4	0	0
O75083	Cluster of WD repeat-containing protein 1	WDR1	66 kDa	1	1	1	1	1	1.1	0.4	0.4	0	0
Q9Y4L1	Cluster of Hypoxia up-regulated protein 1	HYOU1	111 kDa	1	1	0.8	0.9	1	1.1	0.4	0.4	1	0
O00468	Cluster of Agrin	AGRN	215 kDa	1	1	0.8	0.9	1	1.1	0.4	0.4	1	0
B4DII8	Cluster of Gamma-secretase C-terminal fragment 57	APP	85 kDa	1	1	0.7	0.8	0.9	1.1	0.4	0.4	0	1
E7EU23	Cluster of Rab GDP dissociation inhibitor beta	GDI2	51 kDa	1	1	0.9	0.8	1.1	1	0.4	0.4	0	0

O43776	Asparagine--tRNA ligase, cytoplasmic	NARS	63 kDa	1	1	0.9	1	1	1	0.4	0.4	0	0
P53621	Coatomer subunit alpha	COPA	138 kDa	1	1	1	0.9	1	1	0.4	0.4	0	0
E9PKG1	Cluster of Protein arginine N-methyltransferase 1	PRMT1	38 kDa	1	1	0.8	0.9	1	1	0.4	0.4	0	0
P40227	Cluster of T-complex protein 1 subunit zeta	CCT6A	58 kDa	1	1	0.7	0.7	1	1	0.4	0.4	0	0
P68104	Elongation factor 1-alpha 1	EEF1A1	50 kDa	1	1	0.9	0.9	0.8	0.9	0.4	0.4	0	0
P30153	Cluster of Serine/threonine-protein phosphatase 2A 65 kDa	PPP2R1A	65 kDa	1	1	0.7	0.6	0.8	0.9	0.4	0.4	0	0
B4DGU4	Cluster of Catenin beta-1	CTNNB1	85 kDa	1	1	0.7	0.7	0.9	0.8	0.4	0.4	0	0
P28066	Cluster of Proteasome subunit alpha type-5	PSMA5	26 kDa	1	1	0.7	0.6	0.9	0.8	0.4	0.4	0	0
P32119	Cluster of Peroxiredoxin-2	PRDX2	22 kDa	1	1	0.6	0.6	0.9	0.8	0.4	0.4	0	0
J3KNT0	Cluster of Fascin	FSCN1	52 kDa	1	1	0.7	0.6	0.8	0.8	0.4	0.4	0	0
P0C0S5	Cluster of Histone H2A.Z	H2AFZ	14 kDa	1	1	0.7	0.6	0.8	0.8	0.4	0.4	0	0
P26583	Cluster of High mobility group protein B2	HMGB2	24 kDa	1	1	0.6	0.5	0.9	0.6	0.4	0.4	0	0
P07996	Cluster of Thrombospondin-1	THBS1	129 kDa	1	1	0.6	0.6	0.7	0.6	0.4	0.4	1	0
E9PFZ5	Cluster of Inactive tyrosine-protein kinase 7	PTK7	119 kDa	No Va	No Va	No Value	No Value	No Value	No Value	No Val	No Val	1	1
Q02818	Cluster of Nucleobindin-1	NUCB1	54 kDa	1	1	0.4	0.4	0.6	0.6	0.4	0.4	1	0
P53618	Coatomer subunit beta	COPB1	107 kDa	1	1	0.5	0.7	0.4	0.4	0.4	0.4	0	0
B4DTG2	Cluster of Elongation factor 1-gamma	EEF1G	56 kDa	1	1	3	2.7	3.2	2.9	0.3	0.4	0	0
Q08945	Cluster of FACT complex subunit SSRP1	SSRP1	81 kDa	1	1	0.7	1	1	1.5	0.3	0.4	0	0
P61978	Cluster of Isoform 3 of Heterogeneous nuclear ribonucleopr	HNRNPK	49 kDa	No Va	1	No Value	2.5	No Value	1.4	No Val	1.4	0	0
P55884	Eukaryotic translation initiation factor 3 subunit B	EIF3B	92 kDa	1	1	1.3	1.5	1.1	1.3	0.3	0.4	0	0
B3KS98	Cluster of Eukaryotic translation initiation factor 3 subunit	EIF3H	42 kDa	1	1	1.1	1.2	0.9	1.3	0.3	0.4	0	0
P06396	Cluster of Isoform 2 of Gelsolin	GSN	81 kDa	1	1	0.8	0.8	1.3	1.2	0.3	0.4	1	0
P24534	Elongation factor 1-beta	EEF1B2	25 kDa	1	1	1	1.1	1.1	1.2	0.3	0.4	0	0
P12429	Annexin A3	ANXA3	36 kDa	1	1	1.1	1.1	1	1	0.3	0.4	0	0
P63104	Cluster of 14-3-3 protein zeta/delta	YWHAZ	28 kDa	1	1	1	1	1	1	0.3	0.4	0	0
P22234	Cluster of Multifunctional protein ADE2	PAICS	47 kDa	1	1	0.6	0.8	1	1	0.3	0.4	0	0
Q06830	Peroxiredoxin-1	PRDX1	22 kDa	1	1	0.7	0.7	0.9	1	0.3	0.4	0	0
Q9Y5B9	FACT complex subunit SPT16	SUPT16H	120 kDa	1	1	0.9	0.8	0.9	0.9	0.3	0.4	0	0
O00231	Cluster of 26S proteasome non-ATPase regulatory subunit	PSMD11	47 kDa	1	1	0.7	0.8	0.9	0.9	0.3	0.4	0	0
P19021	Cluster of Isoform 3 of Peptidyl-glycine alpha-amidating m	PAM	101 kDa	No Va	No Va	No Value	No Value	No Value	No Value	No Val	No Val	1	1
P48444	Cluster of Coatomer subunit delta	ARCN1	57 kDa	1	1	0.7	0.8	0.9	0.9	0.3	0.4	1	0
P38646	Cluster of Stress-70 protein, mitochondrial	HSPA9	74 kDa	1	1	0.6	0.7	0.9	0.9	0.3	0.4	1	0
Q9BXJ9	Cluster of N-alpha-acetyltransferase 15, NatA auxiliary sub	NAA15	101 kDa	1	1	0.6	0.7	0.8	0.9	0.3	0.4	0	0
P05455	Lupus La protein	SSB	47 kDa	1	1	0.8	0.9	0.8	0.8	0.3	0.4	0	0

B4DQJ8	6-phosphogluconate dehydrogenase, decarboxylating	PGD	52 kDa	1	1	0.7	0.6	0.8	0.7	0.3	0.4	0	0
P05783	Cluster of Keratin, type I cytoskeletal 18	KRT18	48 kDa	1	1	0.5	0.6	0.6	0.7	0.3	0.4	0	0
P33176	Cluster of Kinesin-1 heavy chain	KIF5B	110 kDa	1	1	0.9	0.9	0.9	1	0.7	0.8	0	0
P41250	Glycine--tRNA ligase	GARS	83 kDa	1	1	1.2	1.2	1	1	0.9	0.9	1	0
O14980	Exportin-1	XPO1	123 kDa	1	1	0.8	0.8	0.9	0.8	0.6	0.6	0	0
A6NN80	Cluster of Annexin	ANXA6	75 kDa	1	1	1.2	1.2	1.1	1.1	0.2	0.3	0	0
Q7KZF4	Staphylococcal nuclease domain-containing protein 1	SND1	102 kDa	1	1	1.1	1.6	0.9	1.1	0.8	0.5	0	0
J9JID7	Lamin B2, isoform CRA_a	LMNB2	70 kDa	1	1	1.4	1	1.1	0.8	0.8	0.5	0	0
Q15029	116 kDa U5 small nuclear ribonucleoprotein component	EFTUD2	109 kDa	1	1	0.7	0.7	1.1	0.8	0.8	0.5	0	0
Q15365	Cluster of Poly(rC)-binding protein 1	PCBP1	37 kDa	1	1	0.5	0.3	0.6	0.4	0.8	0.5	0	0
B4DUC8	Cluster of Purine nucleoside phosphorylase A	MTAP	33 kDa	1	1	1.1	1	1.6	1.6	0.7	0.5	0	0
P40926	Malate dehydrogenase, mitochondrial	MDH2	36 kDa	1	1	1.4	1.2	1.3	1.2	0.7	0.5	0	0
P11413	Cluster of Isoform 3 of Glucose-6-phosphate 1-dehydrogena	G6PD	62 kDa	1	1	1.1	1.2	0.9	1.1	0.7	0.5	0	0
P12004	Proliferating cell nuclear antigen	PCNA	29 kDa	1	1	1	0.8	1.3	1	0.7	0.5	0	0
Q96KP4	Cluster of Cytosolic non-specific dipeptidase	CNDP2	53 kDa	1	1	0.9	1.1	1.1	1	0.7	0.5	0	0
Q7Z4V5	Cluster of Hepatoma-derived growth factor-related protein 2	HDGFRP2	74 kDa	1	1	1.3	1.1	1	1	0.7	0.5	0	0
Q01105	Cluster of Protein SET	SET	33 kDa	1	1	0.9	0.8	1.1	0.9	0.7	0.5	0	0
P14735	Insulin-degrading enzyme	IDE	118 kDa	1	1	1.2	1	0.9	0.9	0.7	0.5	1	0
Q9Y230	RuvB-like 2	RUVBL2	51 kDa	1	1	1	0.9	0.9	0.9	0.7	0.5	0	0
Q99460	Cluster of 26S proteasome non-ATPase regulatory subunit 1	PSMD1	106 kDa	1	1	0.9	0.9	0.9	0.9	0.7	0.5	1	1
E9PE77	Cluster of Fibronectin	FN1	256 kDa	1	1	0.7	0.7	0.9	0.9	0.7	0.5	1	0
P54577	Tyrosine--tRNA ligase, cytoplasmic	YARS	59 kDa	1	1	0.8	0.7	0.8	0.9	0.7	0.5	0	0
Q06323	Cluster of Proteasome activator complex subunit 1	PSME1	29 kDa	1	1	0.8	0.9	0.7	0.9	0.7	0.5	0	0
G5E9M5	Cluster of Interleukin enhancer binding factor 3, 90kDa, iso	ILF3	96 kDa	1	1	0.8	0.9	1.2	1.1	1.8	1.5	0	0
O14818	Cluster of Proteasome subunit alpha type-7	PSMA7	28 kDa	1	1	0.9	0.7	0.8	0.7	0.7	0.5	0	0
P25789	Cluster of Proteasome subunit alpha type-4	PSMA4	29 kDa	1	1	0.5	0.6	0.4	0.5	0.7	0.5	0	0
P30041	Peroxiredoxin-6	PRDX6	25 kDa	1	1	1.1	1.1	1.8	2	0.6	0.5	0	0
P08581	Cluster of Hepatocyte growth factor receptor	MET	156 kDa	1	1	1	0.9	1.5	1.5	0.6	0.5	1	1
P60228	Cluster of Eukaryotic translation initiation factor 3 subunit E	EIF3E	52 kDa	1	1	1.3	1.1	1.1	1.1	6.9	6.6	0	0
Q8NHW5	Cluster of 60S acidic ribosomal protein P0-like	RPLP0P6	34 kDa	1	1	0.9	0.8	1.4	1.4	0.6	0.5		
P26640	Cluster of Valine--tRNA ligase	VARS	140 kDa	1	1	1.3	1	1.5	1.2	0.6	0.5	0	0
P17174	Cluster of Aspartate aminotransferase, cytoplasmic	GOT1	46 kDa	1	1	1.1	1.1	1.2	1.2	0.6	0.5	0	0
P29317	Cluster of Ephrin type-A receptor 2	EPHA2	108 kDa	1	1	1.1	0.9	1.1	1.2	0.6	0.5	0	1
P49588	Cluster of Alanine--tRNA ligase, cytoplasmic	AARS	107 kDa	1	1	0.9	1.2	1	1.2	0.6	0.5	0	0

P11387	Cluster of DNA topoisomerase 1	TOP1	91 kDa	1	1	1.2	1.1	0.9	1.2	0.6	0.5	0	0
Q9P2E9	Cluster of Ribosome-binding protein 1	RRBP1	152 kDa	1	1	1.2	1.2	1.2	1.1	0.6	0.5	0	1
P78527	DNA-dependent protein kinase catalytic subunit	PRKDC	469 kDa	1	1	1	0.9	1.1	1.1	0.6	0.5	0	0
P45974	Ubiquitin carboxyl-terminal hydrolase 5	USP5	96 kDa	1	1	1	1	1.2	1	0.6	0.5	0	0
P50851	Cluster of Lipopolysaccharide-responsive and beige-like an	LRBA	319 kDa	1	1	0.9	0.8	1.1	1	0.6	0.5	0	1
P61006	Cluster of Ras-related protein Rab-8A	RAB8A	24 kDa	1	1	1.1	1.1	1	1	0.6	0.5	0	0
Q9NS15	Cluster of Latent-transforming growth factor beta-binding p	LTBP3	139 kDa	1	1	1.1	1.1	1	1	0.6	0.5	1	0
P35221	Cluster of Catenin alpha-1	CTNNA1	100 kDa	1	1	0.9	0.9	1	1	0.6	0.5	0	0
P62258	Cluster of 14-3-3 protein epsilon	YWHAE	29 kDa	1	1	1	0.9	0.9	1	0.6	0.5	0	0
B0QY89	Cluster of Eukaryotic translation initiation factor 3 subunit	EIF3L	71 kDa	1	1	0.9	0.7	0.9	1	0.6	0.5	0	1
O15144	Cluster of Actin-related protein 2/3 complex subunit 2	ARPC2	34 kDa	1	1	1	0.8	1.2	0.9	0.6	0.5	0	0
O15355	Protein phosphatase 1G	PPM1G	59 kDa	1	1	1	0.9	1	0.9	0.6	0.5	0	0
O60763	Cluster of General vesicular transport factor p115	USO1	108 kDa	1	1	1.3	1.3	1.1	1	0.8	0.9	0	0
O60841	Eukaryotic translation initiation factor 5B	EIF5B	139 kDa	1	1	1	1.1	0.9	0.9	0.6	0.5	0	0
P62979	Cluster of Ubiquitin-40S ribosomal protein S27a	RPS27A	18 kDa	1	1	1	1.1	0.8	0.9	0.6	0.5	0	0
P63244	Cluster of Guanine nucleotide-binding protein subunit beta-	GNB2L1	35 kDa	1	1	1	0.9	1.1	0.8	0.6	0.5	0	0
Q86UP2	Cluster of Kinectin	KTN1	156 kDa	1	1	0.9	1.2	1	0.8	0.6	0.5	0	1
Q9P2B2	Prostaglandin F2 receptor negative regulator	PTGFRN	99 kDa	1	1	0.9	0.9	0.9	0.8	0.6	0.5	0	1
Q15046	Cluster of Lysine--tRNA ligase	KARS	68 kDa	1	1	1	1	0.8	0.8	0.6	0.5	0	0
P61158	Cluster of Actin-related protein 3	ACTR3	47 kDa	1	1	0.9	0.9	0.8	0.8	0.6	0.5	0	0
O94907	Cluster of Dickkopf-related protein 1	DKK1	29 kDa	1	1	0.8	0.8	0.8	0.8	0.6	0.5	3	0
B4DZI8	Cluster of Coatomer protein complex, subunit beta 2 (Beta p	COPB2	99 kDa	1	1	0.8	0.8	0.8	0.8	0.6	0.5	0	0
P41252	Cluster of Isoleucine--tRNA ligase, cytoplasmic	IARS	145 kDa	1	1	0.9	0.8	1	0.7	0.6	0.5	0	0
P14868	Cluster of Aspartate--tRNA ligase, cytoplasmic	DARS	46 kDa	1	1	0.8	0.8	0.7	0.7	0.6	0.5	0	0
P43034	Cluster of Platelet-activating factor acetylhydrolase IB subu	PAFAH1B1	47 kDa	1	1	0.8	0.7	0.7	0.7	0.6	0.5	0	0
Q6P2Q9	Cluster of Pre-mRNA-processing-splicing factor 8	PRPF8	274 kDa	1	1	0.7	0.7	0.7	0.7	0.6	0.5	0	0
P07686	Cluster of Beta-hexosaminidase subunit beta	HEXB	63 kDa	1	1	0.6	0.6	0.7	0.7	0.6	0.5	1	0
P23246	Splicing factor, proline- and glutamine-rich	SFPQ	76 kDa	1	1	0.6	0.5	0.7	0.7	0.6	0.5	0	0
Q9Y266	Nuclear migration protein nudC	NUDC	38 kDa	1	1	1.1	0.8	0.7	0.6	0.6	0.5	0	1
P07910	Cluster of Heterogeneous nuclear ribonucleoproteins C1/C2	HNRNPC	34 kDa	1	1	0.9	0.9	0.6	0.5	0.6	0.5	0	0
P20618	Proteasome subunit beta type-1	PSMB1	26 kDa	1	1	0.5	0.4	0.6	0.5	0.6	0.5	0	0
Q92945	Far upstream element-binding protein 2	KHSRP	73 kDa	1	1	2.8	2.3	1.8	1.9	0.5	0.5	0	0
P08195	Cluster of Isoform 2 of 4F2 cell-surface antigen heavy chain	SLC3A2	58 kDa	1	1	1.1	1.1	1.5	1.6	0.5	0.5	0	1
P14866	Heterogeneous nuclear ribonucleoprotein L	HNRNPL	64 kDa	1	1	1.2	1.2	1.4	1.5	0.5	0.5	0	0
Q86XP3	ATP-dependent RNA helicase DDX42	DDX42	103 kDa	1	1	0.8	0.9	1.6	1.4	0.5	0.5	0	0
Q92499	ATP-dependent RNA helicase DDX1	DDX1	82 kDa	1	1	1.6	1.7	1.4	1.3	0.5	0.5	0	0

E9PEZ3	Cluster of Protein diaphanous homolog 1	DIAPH1	141 kDa	1	1	1.2	1.2	1.3	1.3	0.5	0.5	0	0
Q5TEC6	Cluster of Histone H3	HIST2H3PS2	15 kDa	1	1	1.1	1.1	1.3	1.3	0.5	0.5	0	0
E9PFM1	Cluster of Eukaryotic translation initiation factor 4 gamma 1	EIF4G1	176 kDa	1	1	1	0.7	1.3	1.3	0.5	0.5	0	0
Q5T4S7	Cluster of Isoform 2 of E3 ubiquitin-protein ligase UBR4	UBR4	576 kDa	1	1	1.2	1	1.4	1.2	0.5	0.5	0	1
H0Y8C6	Importin-5 (Fragment)	IPO5	124 kDa	1	1	1.1	0.8	1.3	1.2	0.5	0.5	0	0
O43175	D-3-phosphoglycerate dehydrogenase	PHGDH	57 kDa	1	1	1	1.1	1.2	1.2	0.5	0.5	0	0
Q06210	Cluster of Glucosamine--fructose-6-phosphate aminotransfe	GFPT1	79 kDa	1	1	1	1	1.2	1.2	0.5	0.5	0	1
Q96QK1	Cluster of Vacuolar protein sorting-associated protein 35	VPS35	92 kDa	1	1	1.2	1.2	1.2	1.1	0.5	0.5	0	0
D6RER5	Cluster of Septin-11	SEPT11	50 kDa	1	1	1.1	1	1.2	1.1	0.5	0.5	0	0
P14314	Glucosidase 2 subunit beta	PRKCSH	59 kDa	1	1	0.9	0.9	1.2	1.1	0.5	0.5	1	0
P46777	60S ribosomal protein L5	RPL5	34 kDa	1	1	0.8	0.9	1.2	1.1	0.5	0.5	0	0
Q14008	Cluster of Cytoskeleton-associated protein 5	CKAP5	226 kDa	1	1	1.2	1.1	1.1	1.1	0.5	0.5	0	0
P11586	Cluster of C-1-tetrahydrofolate synthase, cytoplasmic	MTHFD1	102 kDa	1	1	1.1	1.1	1.1	1.1	0.5	0.5	0	1
P23528	Cofilin-1	CFL1	19 kDa	1	1	0.8	0.7	1.1	1.1	0.5	0.5	0	0
P49915	GMP synthase [glutamine-hydrolyzing]	GMPS	77 kDa	1	1	0.9	1	1	1.1	0.5	0.5	0	0
Q92859	Neogenin	NEO1	160 kDa	1	1	1	0.9	1	1.1	0.5	0.5	0	1
F5H737	Adenosylhomocysteinase	AHCY	45 kDa	1	1	0.9	1	1.1	1	0.5	0.5	0	0
E9PBU3	Cluster of Phosphoribosylaminoimidazolecarboxamide form	ATIC	65 kDa	No Va	No Va	No Value	No Value	No Value	No Value	No Val	No Val	0	0
Q9UGI8	Cluster of Isoform 2 of Testin	TES	47 kDa	1	1	1.1	1.1	1	1	0.5	0.5	0	0
F8W8J4	Myoferlin	MYOF	235 kDa	1	1	0.9	0.9	1	1	0.5	0.5	0	1
O75533	Splicing factor 3B subunit 1	SF3B1	146 kDa	1	1	0.9	0.9	1	1	0.5	0.5	0	1
P16403	Cluster of Histone H1.2	HIST1H1C	21 kDa	1	1	0.9	0.9	1	1	0.5	0.5	0	0
P27348	Cluster of 14-3-3 protein theta	YWHAQ	28 kDa	1	1	0.9	0.9	1	1	0.5	0.5	0	0
Q06481	Cluster of Amyloid-like protein 2	APLP2	87 kDa	1	1	0.8	0.9	1	1	0.5	0.5	1	1
Q9NSD9	Phenylalanine--tRNA ligase beta subunit	FARSB	66 kDa	1	1	0.8	0.7	1	1	0.5	0.5	0	0
P18669	Cluster of Phosphoglycerate mutase 1	PGAM1	29 kDa	1	1	0.9	0.9	0.9	1	0.5	0.5	0	0
Q02952	Cluster of A-kinase anchor protein 12	AKAP12	191 kDa	No Va	No Va	No Value	No Value	No Value	No Value	No Val	No Val	0	0
Q9UNM6	Cluster of 26S proteasome non-ATPase regulatory subunit 1	PSMD13	43 kDa	1	No Va	1.2	No Value	1.9	No Value	0.7	No Val	0	0
P25786	Cluster of Proteasome subunit alpha type-1	PSMA1	30 kDa	1	1	0.9	0.7	0.9	1	0.5	0.5	0	0
P07858	Cathepsin B	CTSB	38 kDa	1	1	0.7	0.8	1.1	0.9	0.5	0.5	1	0
F5H098	Malate dehydrogenase, cytoplasmic	MDH1	39 kDa	1	1	0.9	0.9	1	0.9	0.5	0.5	0	0
P28799	Granulins	GRN	64 kDa	1	1	1.5	1.6	0.9	0.9	0.5	0.5	1	0
E9PGT1	Cluster of Translin	TSN	26 kDa	No Va	No Va	No Value	No Value	No Value	No Value	No Val	No Val	0	1
G3V5Z7	Cluster of Proteasome subunit alpha type	PSMA6	28 kDa	1	1	1.1	1.1	0.9	0.9	0.5	0.5	0	0
Q01813	Cluster of 6-phosphofructokinase type C	PFKP	86 kDa	1	1	0.8	0.8	0.9	0.9	0.5	0.5	1	0
Q01970	1-phosphatidylinositol 4,5-bisphosphate phosphodiesterase	PLCB3	139 kDa	1	1	0.8	0.8	0.9	0.9	0.5	0.5	0	0
Q9HC35	Cluster of Echinoderm microtubule-associated protein-like 4	EML4	109 kDa	No Va	1	No Value	1.4	No Value	2.1	No Val	6.6	0	0

Q16543	Hsp90 co-chaperone Cdc37	CDC37	44 kDa	1	1	1	1	1.1	1	1.1	1.1	0	0
F5GWX5	Cluster of Chromodomain-helicase-DNA-binding protein 4	CHD4	217 kDa	1	1	0.9	0.8	1	0.8	0.5	0.5	0	0
P43490	Nicotinamide phosphoribosyltransferase	NAMPT	56 kDa	1	1	0.9	0.9	0.9	0.8	0.5	0.5	0	0
Q9UBT2	SUMO-activating enzyme subunit 2	UBA2	71 kDa	1	1	0.8	0.9	0.9	0.8	0.5	0.5	0	1
P13797	Cluster of Plastin-3	PLS3	71 kDa	1	1	0.7	0.7	0.9	0.8	0.5	0.5	0	0
Q13435	Cluster of Splicing factor 3B subunit 2	SF3B2	100 kDa	1	1	1	1	0.8	0.8	0.5	0.5	0	0
Q15075	Early endosome antigen 1	EEA1	162 kDa	1	1	0.8	0.9	0.8	0.8	0.5	0.5	0	0
Q14118	Dystroglycan	DAG1	97 kDa	1	1	0.8	0.8	0.8	0.8	0.5	0.5	1	1
Q9UBS4	Cluster of DnaJ homolog subfamily B member 11	DNAJB11	41 kDa	1	1	0.8	0.8	0.8	0.8	0.5	0.5	1	0
Q9Y265	Cluster of RuvB-like 1	RUVBL1	50 kDa	1	1	0.6	0.7	0.8	0.8	0.5	0.5	0	0
E9PK82	Cluster of 40S ribosomal protein S3 (Fragment)	RPS3	14 kDa	No Va	No Va	No Value	No Value	No Value	No Value	No Val	No Val	0	0
P05556	Cluster of Integrin beta-1	ITGB1	88 kDa	1	1	1	0.9	0.7	0.8	0.5	0.5	1	1
P07093	Cluster of Isoform 2 of Glia-derived nexin	SERPINE2	44 kDa	1	1	0.8	0.6	0.8	0.7	0.5	0.5	1	0
Q14CX7	Cluster of N-alpha-acetyltransferase 25, NatB auxiliary sub	NAA25	112 kDa	1	1	0.8	0.8	0.7	0.7	0.5	0.5	0	1
Q9BWD1	Cluster of Acetyl-CoA acetyltransferase, cytosolic	ACAT2	41 kDa	1	1	0.8	0.8	0.7	0.7	0.5	0.5	0	0
O75874	Isocitrate dehydrogenase [NADP] cytoplasmic	IDH1	47 kDa	1	1	1	0.9	0.9	0.6	0.5	0.5	0	0
Q2L7G6	Heterogeneous nuclear ribonucleoprotein R	HNRNPR	67 kDa	1	1	0.8	0.8	0.7	0.5	0.5	0.5	0	0
E7EP74	Cluster of Golgin subfamily B member 1	GOLGB1	377 kDa	1	1	0.5	0.5	0.5	0.5	0.5	0.5	0	1
P52292	Importin subunit alpha-2	KPNA2	58 kDa	1	1	1.1	1	1.5	1.6	0.4	0.5	0	0
P62495	Cluster of Eukaryotic peptide chain release factor subunit 1	ETF1	49 kDa	No Va	No Va	No Value	No Value	No Value	No Value	No Val	No Val	0	0
Q13616	Cullin-1	CUL1	90 kDa	1	1	0.9	0.9	1.5	1.6	0.4	0.5	0	0
P18065	Cluster of Insulin-like growth factor-binding protein 2	IGFBP2	35 kDa	1	1	0.9	0.9	1.6	1.5	0.4	0.5	1	0
P22626	Heterogeneous nuclear ribonucleoproteins A2/B1	HNRNPA2B1	37 kDa	1	1	1	1	1.2	1.2	0.4	0.5	0	0
Q9UHD1	Cysteine and histidine-rich domain-containing protein 1	CHORDC1	37 kDa	1	1	1.2	1.2	1	1.1	0.4	0.5	0	0
O00299	Chloride intracellular channel protein 1	CLIC1	27 kDa	1	1	1.1	1.1	1	1.1	0.4	0.5	0	0
P54136	Arginine--tRNA ligase, cytoplasmic	RARS	75 kDa	1	1	0.9	0.8	0.7	1	0.4	0.5	0	0
P22102	Cluster of Trifunctional purine biosynthetic protein adenosi	GART	108 kDa	1	1	0.9	0.8	0.9	0.9	0.4	0.5	0	0
P25788	Proteasome subunit alpha type-3	PSMA3	28 kDa	1	1	0.8	0.8	0.9	0.9	0.4	0.5	0	0
Q14978	Isoform Beta of Nucleolar and coiled-body phosphoprotein	NOLC1	75 kDa	1	1	0.9	1	0.8	0.9	0.4	0.5	0	0
B1Q2N1	Cluster of ATP-dependent RNA helicase DDX39A	DDX39A	30 kDa	1	1	0.7	0.8	0.8	0.9	0.4	0.5	0	0
P27816	Cluster of Isoform 6 of Microtubule-associated protein 4	MAP4	120 kDa	1	1	0.8	0.7	0.8	0.9	0.4	0.5	0	0
P50454	Cluster of Serpin H1	SERPINH1	46 kDa	1	1	0.7	0.7	0.8	0.9	0.4	0.5	1	0
G3V3M6	Cluster of DNA-(apurinic or apyrimidinic site) lyase (Fragn	APEX1	29 kDa	1	1	0.5	0.7	0.6	0.9	0.4	0.5	0	0
P41091	Eukaryotic translation initiation factor 2 subunit 3	EIF2S3	51 kDa	1	1	1.1	1.1	0.8	0.8	0.4	0.5	0	0
Q9P2J5	Leucine--tRNA ligase, cytoplasmic	LARS	134 kDa	1	1	0.7	0.9	0.8	0.8	0.4	0.5	0	0
Q9Y617	Phosphoserine aminotransferase	PSAT1	40 kDa	1	1	1.1	1	0.7	0.8	0.4	0.5	0	0
O00154	Cluster of Cytosolic acyl coenzyme A thioester hydrolase	ACOT7	42 kDa	1	1	0.9	1	0.7	0.8	0.4	0.5	0	0

P04004	Vitronectin	VTN	54 kDa	1	1	0.5	0.6	0.6	0.8	0.4	0.5	1	0
P37837	Transaldolase	TALDO1	38 kDa	1	1	0.7	0.7	0.8	0.7	0.4	0.5	0	0
P49720	Proteasome subunit beta type-3	PSMB3	23 kDa	1	1	0.6	0.8	0.5	0.6	0.4	0.5	0	0
P50502	Cluster of Hsc70-interacting protein	ST13	41 kDa	1	1	1.3	1.3	1.1	1.1	0.3	0.5	0	0
P62316	Small nuclear ribonucleoprotein Sm D2	SNRPD2	14 kDa	1	1	1.1	1.1	1.1	1.1	1.7	1.8	0	0
Q9P258	Protein RCC2	RCC2	56 kDa	1	1	0.9	0.7	0.7	0.7	0.3	0.5	0	0
O75116	Rho-associated protein kinase 2	ROCK2	161 kDa	1	1	0.5	0.6	0.6	0.7	0.3	0.5	0	0
P15924	Desmoplakin	DSP	332 kDa	1	1	0.8	0.8	0.9	0.9	2.1	2.1	0	0
Q96AE4	Isoform 2 of Far upstream element-binding protein 1	FUBP1	69 kDa	1	1	0.9	1	1	1	1.7	1.8	0	0
O95782	AP-2 complex subunit alpha-1	AP2A1	108 kDa	1	1	0.9	0.9	0.9	0.9	0.9	0.9	0	0
P48163	NADP-dependent malic enzyme	ME1	64 kDa	1	1	1.2	1.1	1.1	1	1.2	1.1	0	0
P35052	Cluster of Glypican-1	GPC1	62 kDa	No Va	No Va	No Value	No Value	No Value	No Value	No Val	No Val	0	1
P16144	Cluster of Integrin beta-4	ITGB4	202 kDa	No Va	No Va	No Value	No Value	No Value	No Value	No Val	No Val	0	1
P35998	Cluster of 26S protease regulatory subunit 7	PSMC2	49 kDa	1	1	0.9	1.2	0.9	0.9	1	0.6	0	0
E7ESI6	Cluster of Thioredoxin reductase 1, cytoplasmic	TXNRD1	64 kDa	1	No Va	1.8	No Value	2.1	No Value	1.2	No Val	0	0
P12277	Cluster of Creatine kinase B-type	CKB	43 kDa	1	1	1.6	1.2	2	1.3	0.9	0.6	0	0
P30048	Thioredoxin-dependent peroxide reductase, mitochondrial	PRDX3	28 kDa	1	1	1.4	1	1.1	1	0.9	0.6	0	0
P42704	Cluster of Leucine-rich PPR motif-containing protein, mito	LRPPRC	158 kDa	1	1	1.1	1	1.1	0.9	0.9	0.6	0	0
P63096	Cluster of Guanine nucleotide-binding protein G(i) subunit	GNAI1	40 kDa	1	1	0.4	0.4	0.8	0.9	0.9	0.6	0	0
B3KXW5	AP-1 complex subunit gamma-1	AP1G1	94 kDa	1	1	0.9	0.9	0.8	0.8	1	0.9	0	0
P23381	Tryptophan--tRNA ligase, cytoplasmic	WARS	53 kDa	1	1	0.9	1	1.2	1.5	0.8	0.6	0	0
B5MCQ5	Protein disulfide-isomerase A6	PDIA6	53 kDa	1	1	1.1	1.1	1.2	1.2	0.8	0.6	1	1
H7BYM6	Cluster of High mobility group protein HMG-I/HMG-Y	HMGA1	34 kDa	1	1	0.9	0.9	0.9	1.2	0.8	0.6	0	0
P00491	Cluster of Purine nucleoside phosphorylase	PNP	32 kDa	1	1	0.8	0.9	1	1.1	0.8	0.6	1	0
Q9NYU2	Cluster of UDP-glucose:glycoprotein glucosyltransferase 1	UGGT1	177 kDa	1	1	1.2	1.1	1	1	0.8	0.6	1	0
P61160	Actin-related protein 2	ACTR2	45 kDa	1	1	0.9	1.1	0.9	1	0.8	0.6	0	0
Q92626	Cluster of Peroxidasin homolog	PXDN	165 kDa	1	1	1.1	1.1	1.1	0.9	0.8	0.6	1	0
B4DNJ6	Serine-threonine kinase receptor-associated protein	STRAP	40 kDa	1	1	1.1	0.8	1.1	0.9	0.8	0.6	0	0
Q15459	Cluster of Splicing factor 3A subunit 1	SF3A1	89 kDa	1	1	0.8	0.8	1	1	1	1.1	0	0
Q7L014	Cluster of Probable ATP-dependent RNA helicase DDX46	DDX46	117 kDa	1	1	0.8	0.8	0.9	0.8	0.8	0.6	0	0
Q9UL46	Proteasome activator complex subunit 2	PSME2	27 kDa	1	1	0.9	0.7	0.9	0.8	0.8	0.6	0	0
P25685	DnaJ homolog subfamily B member 1	DNAJB1	38 kDa	1	1	0.9	0.8	0.8	0.8	0.8	0.6	0	0
P25705	ATP synthase subunit alpha, mitochondrial	ATP5A1	60 kDa	1	1	1.2	0.8	1.1	0.7	0.8	0.6	1	0
P33908	Mannosyl-oligosaccharide 1,2-alpha-mannosidase IA	MAN1A1	73 kDa	1	1	0.7	0.6	0.7	0.7	1.4	1.2	0	1
B5MCA4	Cluster of Epithelial cell adhesion molecule	EPCAM	38 kDa	1	1	0.9	0.6	1.1	0.7	0.8	0.6	1	1
E7EX90	Cluster of Dynactin subunit 1	DCTN1	139 kDa	No Va	No Va	No Value	No Value	No Value	No Value	No Val	No Val	0	0
E9PAV2	Cluster of Proteasome subunit beta type-5	PSMB5	18 kDa	1	1	0.8	0.6	1	0.7	0.8	0.6	0	0

P27694	Cluster of Replication protein A 70 kDa DNA-binding subu	RPA1	68 kDa	1	1	0.9	0.9	0.8	0.5	0.8	0.6	0	0
Q05639	Elongation factor 1-alpha 2	EEF1A2	50 kDa	1	1	0.9	0.6	0.5	0.5	0.8	0.6	0	0
D6RAT0	Cluster of 40S ribosomal protein S3a	RPS3A	26 kDa	No Va	No Va	No Value	No Value	No Value	No Value	No Val	No Val	0	0
G5E9Y2	Cluster of X-prolyl aminopeptidase (Aminopeptidase P) 1, s	XPNPEP1	75 kDa	1	1	2.9	3.3	2	2.3	0.7	0.6	0	0
P35573	Glycogen debranching enzyme	AGL	175 kDa	1	1	1.4	1.5	1.7	1.6	0.7	0.6	0	1
Q9P287	Cluster of BRCA2 and CDKN1A-interacting protein	BCCIP	36 kDa	1	1	1.7	1.5	1.8	1.5	0.7	0.6	0	0
F8VRQ1	Cluster of Heterogeneous nuclear ribonucleoprotein A1	HNRNPA1	33 kDa	1	1	0.9	1.2	1.1	1.3	0.7	0.6	0	0
P16870	Cluster of Carboxypeptidase E	CPE	53 kDa	1	1	1.6	1.6	1.3	1.2	0.7	0.6	1	0
P62277	40S ribosomal protein S13	RPS13	17 kDa	1	1	1.2	1.2	1.3	1.3	2.5	2.3	0	0
Q13618	Cluster of Cullin-3	CUL3	89 kDa	1	1	1.1	0.9	1.2	1.1	0.7	0.6	0	0
Q15181	Inorganic pyrophosphatase	PPA1	33 kDa	1	1	1.1	1	1.1	1.1	0.7	0.6	0	0
O95433	Cluster of Activator of 90 kDa heat shock protein ATPase h	AHSA1	38 kDa	1	1	1	0.8	1.1	1.1	0.7	0.6	0	0
P46060	Cluster of Ran GTPase-activating protein 1	RANGAP1	64 kDa	1	1	1.1	1.1	1	1.1	0.7	0.6	0	0
Q7KZ85	Transcription elongation factor SPT6	SUPT6H	199 kDa	1	1	1	0.8	1	1.1	0.7	0.6	0	0
O00203	Cluster of AP-3 complex subunit beta-1	AP3B1	121 kDa	1	1	1.2	1.1	1.2	1	0.7	0.6	0	0
P07737	Profilin-1	PFN1	15 kDa	1	1	1.2	1.1	1.1	1	0.7	0.6	1	0
P60953	Cluster of Cell division control protein 42 homolog	CDC42	21 kDa	1	1	1.2	1	1.1	1	0.7	0.6	0	0
Q5VYK3	Cluster of Proteasome-associated protein ECM29 homolog	KIAA0368	204 kDa	No Va	No Va	No Value	No Value	No Value	No Value	No Val	No Val	0	0
Q9UQE7	Structural maintenance of chromosomes protein 3	SMC3	142 kDa	1	1	1	1	1.1	1	0.7	0.6	0	0
E7ERJ7	Cluster of Polyadenylate-binding protein 1	PABPC1	67 kDa	No Va	No Va	No Value	No Value	No Value	No Value	No Val	No Val	0	0
P63241	Cluster of Isoform 2 of Eukaryotic translation initiation fact	EIF5A	20 kDa	1	1	1	0.9	1.1	1	0.7	0.6	0	0
Q93034	Cluster of Cullin-5	CUL5	91 kDa	1	1	1.2	1.1	1	1	0.7	0.6	0	0
Q9NTK5	Isoform 3 of Obg-like ATPase 1	OLA1	31 kDa	1	1	1.1	1.1	1	1	0.7	0.6	0	0
B7Z815	Ubiquitin carboxyl-terminal hydrolase	USP7	126 kDa	1	1	1	1	1	1	0.7	0.6	0	0
P05198	Cluster of Eukaryotic translation initiation factor 2 subunit	EIF2S1	36 kDa	1	1	1	1	0.9	1	0.7	0.6	0	0
P18124	Cluster of 60S ribosomal protein L7	RPL7	29 kDa	1	1	1	1	0.9	1	0.7	0.6	0	0
P22059	Cluster of Oxysterol-binding protein 1	OSBP	89 kDa	1	1	0.9	1	0.9	1	0.7	0.6	0	0
P29692	Cluster of Isoform 3 of Elongation factor 1-delta	EEF1D	29 kDa	1	1	0.7	0.9	0.9	1	0.7	0.6	0	0
Q15019	Cluster of Isoform 2 of Septin-2	SEPT2	45 kDa	1	1	0.7	0.8	0.9	1	0.7	0.6	0	0
E9PCS3	Cluster of 26S proteasome non-ATPase regulatory subunit 2	PSMD2	82 kDa	1	1	0.7	0.8	0.8	1	0.7	0.6	0	1
O14745	Cluster of Na(+)/H(+) exchange regulatory cofactor NHE-R	SLC9A3R1	39 kDa	1	1	0.8	0.8	0.7	1	0.7	0.6	0	0
P52294	Cluster of Importin subunit alpha-1	KPNA1	60 kDa	1	1	1.5	1.4	1.2	0.9	0.7	0.6	0	0
P61221	ATP-binding cassette sub-family E member 1	ABCE1	67 kDa	1	1	1	1.1	1.1	0.9	0.7	0.6	0	0
P05387	Cluster of 60S acidic ribosomal protein P2	RPLP2	12 kDa	1	1	1	0.7	1.1	0.9	0.7	0.6	1	0
P31946	Cluster of 14-3-3 protein beta/alpha	YWHAB	28 kDa	1	1	1.1	1.2	0.8	0.9	0.7	0.6	0	0
O15240	Neurosecretory protein VGF	VGF	67 kDa	1	1	0.7	0.8	0.8	0.9	0.7	0.6	1	0
P01033	Cluster of Metalloproteinase inhibitor 1	TIMP1	23 kDa	1	1	1.4	1.6	1.2	0.8	0.7	0.6	1	0

P61604	Cluster of 10 kDa heat shock protein, mitochondrial	HSPE1	11 kDa	1	1	1.4	1.4	1	0.8	0.7	0.6	0	0
Q13620	Cullin-4B	CUL4B	104 kDa	1	1	1.3	1	1	0.8	0.7	0.6	0	0
Q86TI2	Dipeptidyl peptidase 9	DPP9	98 kDa	1	1	1	1	0.9	0.8	0.7	0.6	0	0
Q9BUQ8	Cluster of Probable ATP-dependent RNA helicase DDX23	DDX23	96 kDa	1	1	0.9	0.9	0.9	0.8	0.7	0.6	0	0
E7EPC6	Cluster of CD44 antigen	CD44	79 kDa	1	1	0.8	0.8	0.9	0.8	0.7	0.6	1	1
P28072	Proteasome subunit beta type-6	PSMB6	25 kDa	1	1	0.7	0.7	0.9	0.8	0.7	0.6	0	0
P49736	Cluster of DNA replication licensing factor MCM2	MCM2	102 kDa	1	1	1.3	1	0.8	0.8	0.7	0.6	0	0
P52597	Heterogeneous nuclear ribonucleoprotein F	HNRNPF	46 kDa	1	1	1	0.8	0.8	0.8	0.7	0.6	0	0
Q06124	Cluster of Tyrosine-protein phosphatase non-receptor type 1	PTPN11	68 kDa	1	1	1	0.8	0.8	0.8	0.7	0.6	0	0
Q9UK22	F-box only protein 2	FBXO2	33 kDa	1	1	0.7	0.8	0.8	0.8	0.7	0.6	0	0
H3BLZ8	Cluster of Probable ATP-dependent RNA helicase DDX17	DDX17	80 kDa	1	1	0.9	0.9	0.8	0.7	0.7	0.6	1	0
O14979	Cluster of Isoform 3 of Heterogeneous nuclear ribonucleopr	HNRNPDL	27 kDa	No Va	No Va	No Value	No Value	No Value	No Value	No Val	No Val	0	0
P27708	Cluster of CAD protein	CAD	243 kDa	1	1	0.8	0.7	0.7	0.7	0.7	0.6	0	0
P30740	Cluster of Leukocyte elastase inhibitor	SERPINB1	43 kDa	1	1	0.6	0.6	0.7	0.7	0.7	0.6	0	0
P62937	Cluster of Peptidyl-prolyl cis-trans isomerase A	PPIA	18 kDa	1	1	0.9	0.7	0.8	0.6	0.7	0.6	0	0
Q7L576	Cluster of Cytoplasmic FMR1-interacting protein 1	CYFIP1	145 kDa	1	1	1.3	1.4	1.5	1.9	0.6	0.6	0	0
O00232	26S proteasome non-ATPase regulatory subunit 12	PSMD12	53 kDa	1	1	1.8	1.9	1.8	1.8	0.6	0.6	0	0
Q12905	Interleukin enhancer-binding factor 2	ILF2	43 kDa	1	1	1.7	1.5	1.6	1.5	0.6	0.6	0	0
Q8TEQ6	Gem-associated protein 5	GEMIN5	169 kDa	1	1	1.4	1.2	1.5	1.5	0.6	0.6	0	0
O43242	Cluster of 26S proteasome non-ATPase regulatory subunit 3	PSMD3	61 kDa	1	1	1.5	1.2	1.8	1.4	0.6	0.6	0	0
O95373	Importin-7	IPO7	120 kDa	1	1	1.5	1.5	1.4	1.4	0.6	0.6	0	0
P49591	Serine--tRNA ligase, cytoplasmic	SARS	59 kDa	1	1	1	1.1	1.3	1.4	0.6	0.6	0	0
Q06203	Amidophosphoribosyltransferase	PPAT	57 kDa	1	1	1	1	1.3	1.4	0.6	0.6	0	0
A7MAP0	Cluster of Coronin	CORO1C	54 kDa	1	1	1.2	1.3	1.1	1.4	0.6	0.6	0	0
B4DVE7	Cluster of Annexin	ANXA11	51 kDa	1	1	1.5	1.3	1.3	1.3	0.6	0.6	0	0
P31153	Cluster of S-adenosylmethionine synthase isoform type-2	MAT2A	44 kDa	1	1	1.2	1.3	1.3	1.3	0.6	0.6	0	0
P52888	Thimet oligopeptidase	THOP1	79 kDa	1	1	1	1	1.3	1.3	0.6	0.6	0	0
Q14258	E3 ubiquitin/ISG15 ligase TRIM25	TRIM25	71 kDa	1	1	1	1.1	1	1.3	0.6	0.6	0	0
Q16851	Cluster of UTP--glucose-1-phosphate uridylyltransferase	UGP2	57 kDa	1	1	0.7	1	0.8	1.3	0.6	0.6	0	0
Q92900	Regulator of nonsense transcripts 1	UPF1	124 kDa	1	1	0.9	0.9	1.3	1.2	0.6	0.6	0	0
Q9Y224	Cluster of UPF0568 protein C14orf166	C14orf166	28 kDa	1	1	1.6	1.4	1.2	1.2	0.6	0.6	0	0
P26447	Protein S100-A4	S100A4	12 kDa	1	1	1.2	1.2	1.2	1.2	0.6	0.6	0	0
P60033	Cluster of CD81 antigen	CD81	26 kDa	1	1	1.1	1.1	1.2	1.2	0.6	0.6	0	1
E9PDC5	Receptor-type tyrosine-protein phosphatase S	PTPRS	217 kDa	1	1	1.1	1.1	1.2	1.2	0.6	0.6	1	1
Q6ZMU5	Tripartite motif-containing protein 72	TRIM72	53 kDa	1	1	1	1.1	1.2	1.2	0.6	0.6	0	0
Q9Y678	Cluster of Coatomer subunit gamma-1	COPG1	98 kDa	1	1	0.7	0.9	1.2	1.2	0.6	0.6	0	0
J3KP07	Cluster of Growth arrest-specific protein 6	GAS6	80 kDa	1	1	1.4	1.4	1	1.2	0.6	0.6	1	0

O95834	Isoform 2 of Echinoderm microtubule-associated protein-lik	EML2	86 kDa	1	1	0.6	1	0.9	1.2	0.6	0.6	0	0
P08758	Cluster of Annexin A5	ANXA5	36 kDa	1	1	0.9	1.3	1.2	1.1	0.6	0.6	0	0
P36551	Coproporphyrinogen-III oxidase, mitochondrial	CPOX	50 kDa	1	1	1	1.1	1.2	1.1	0.6	0.6	0	1
P36873	Cluster of Isoform Gamma-2 of Serine/threonine-protein ph	PPP1CC	39 kDa	1	1	1.2	1	1.2	1.1	0.6	0.6	0	0
Q10570	Cleavage and polyadenylation specificity factor subunit 1	CPSF1	161 kDa	1	1	0.8	1	1.2	1.1	0.6	0.6	0	0
Q16181	Cluster of Septin-7	SEPT7	51 kDa	1	1	1.8	1.6	1.1	1.1	0.6	0.6	0	0
Q8N163	Cluster of DBIRD complex subunit KIAA1967	CCAR2	103 kDa	1	1	1.4	1.3	1.1	1.1	0.6	0.6	0	0
Q13867	Cluster of Bleomycin hydrolase	BLMH	53 kDa	1	1	1.4	1.2	1.1	1.1	0.6	0.6	0	0
Q9Y3I0	tRNA-splicing ligase RtcB homolog	RTCB	55 kDa	1	1	1.1	1.1	1.1	1.1	0.6	0.6	0	0
E9PLM6	Midkine	MDK	17 kDa	1	1	1.1	1.1	1.1	1.1	0.6	0.6	1	0
H0Y7A7	Calmodulin (Fragment)	CALM2	21 kDa	1	1	1.1	1.1	1.1	1.1	0.6	0.6	0	0
P49189	4-trimethylaminobutyraldehyde dehydrogenase	ALDH9A1	54 kDa	1	1	1.1	1.1	1.1	1.1	0.6	0.6	1	0
P52907	F-actin-capping protein subunit alpha-1	CAPZA1	33 kDa	1	1	1	1	1.1	1.1	0.6	0.6	0	0
Q16706	Cluster of Alpha-mannosidase 2	MAN2A1	131 kDa	1	1	1	1.1	1	1.1	0.6	0.6	0	1
Q9NZV1	Cysteine-rich motor neuron 1 protein	CRIM1	114 kDa	1	1	1	1.1	0.9	1.1	0.6	0.6	0	1
P10619	Lysosomal protective protein	CTSA	54 kDa	1	1	1.6	1.3	1.4	1.6	5.6	5.7	1	0
P47895	Cluster of Aldehyde dehydrogenase family 1 member A3	ALDH1A3	56 kDa	1	1	0.9	0.9	1.2	1	0.6	0.6	0	0
P52272	Heterogeneous nuclear ribonucleoprotein M	HNRNPM	78 kDa	1	1	1.2	1	1.1	1	0.6	0.6	0	0
P61289	Proteasome activator complex subunit 3	PSME3	30 kDa	1	1	1	0.9	1.1	1	0.6	0.6	0	0
P62333	Cluster of 26S protease regulatory subunit 10B	PSMC6	44 kDa	1	1	1.1	1.5	1	1	0.6	0.6	0	0
Q14563	Semaphorin-3A	SEMA3A	89 kDa	1	1	1.3	1.3	1	1	0.6	0.6	1	0
G8JLD5	Cluster of Dynamin-1-like protein	DNM1L	80 kDa	1	1	1.2	1.3	1	1	0.6	0.6	0	0
Q13347	Eukaryotic translation initiation factor 3 subunit I	EIF3I	37 kDa	1	1	1.2	1.1	1	1	0.6	0.6	0	0
Q9H0D6	5'-3' exoribonuclease 2	XRN2	109 kDa	1	1	0.8	1	1	1	0.6	0.6	0	0
F5H2A7	Prefoldin subunit 3	VBP1	22 kDa	1	1	1	0.9	1	1	0.6	0.6	0	0
F5HFY4	Cluster of Nucleosome assembly protein 1-like 4	NAP1L4	44 kDa	1	1	0.9	0.9	1	1	0.6	0.6	0	0
G5E9U1	Cdc42-interacting protein 4	TRIP10	68 kDa	1	1	0.9	0.9	1	1	0.6	0.6	0	0
Q15020	Cluster of Squamous cell carcinoma antigen recognized by '	SART3	110 kDa	1	1	0.9	0.9	1	1	0.6	0.6	0	0
Q3LXA3	Cluster of Bifunctional ATP-dependent dihydroxyacetone k	DAK	59 kDa	1	1	0.9	0.9	1	1	0.6	0.6	0	0
Q96TA1	Cluster of Isoform 2 of Niban-like protein 1	FAM129B	83 kDa	No Va	No Va	No Value	No Value	No Valu	No Value	No Val	No Val	0	0
Q9HB71	Calcyclin-binding protein	CACYBP	26 kDa	1	1	0.9	1.1	0.9	1	0.6	0.6	0	0
H0Y9J0	Cluster of Rap1 GTPase-GDP dissociation stimulator 1 (Fra	RAP1GDS1	10 kDa	No Va	No Va	No Value	No Value	No Valu	No Value	No Val	No Val	0	0
P26196	Probable ATP-dependent RNA helicase DDX6	DDX6	54 kDa	1	1	0.9	0.9	0.9	1	0.6	0.6	0	0
P62701	Cluster of 40S ribosomal protein S4, X isoform	RPS4X	30 kDa	1	1	0.8	0.7	0.9	1	0.6	0.6	0	0
P26599	Cluster of Polypyrimidine tract-binding protein 1	PTBP1	57 kDa	1	1	1	1	0.8	1	0.6	0.6	0	0
P49792	Cluster of E3 SUMO-protein ligase RanBP2	RANBP2	358 kDa	1	1	0.9	1	0.8	1	0.6	0.6	0	0
Q02878	60S ribosomal protein L6	RPL6	33 kDa	1	1	0.8	0.9	0.8	1	0.6	0.6	0	0

P67809	Cluster of Nuclease-sensitive element-binding protein 1	YBX1	36 kDa	1	1	0.9	1	1.1	0.9	0.6	0.6	0	0
Q92820	Gamma-glutamyl hydrolase	GGH	36 kDa	1	1	0.9	0.7	1.1	0.9	0.6	0.6	3	0
Q9BXP5	Serrate RNA effector molecule homolog	SRRT	101 kDa	1	1	1	1.2	1	0.9	0.6	0.6	0	0
F5H897	Cluster of Heat shock protein 75 kDa, mitochondrial	TRAP1	74 kDa	1	1	1.1	1.1	1	0.9	0.6	0.6	0	0
G3V1S2	Cullin-2	CUL2	89 kDa	1	1	0.8	0.9	1	0.9	0.6	0.6	0	0
P29144	Cluster of Tripeptidyl-peptidase 2	TPP2	138 kDa	1	1	1.2	0.8	1	0.9	0.6	0.6	0	0
Q14232	Cluster of Translation initiation factor eIF-2B subunit alpha	EIF2B1	34 kDa	1	1	1	1.1	0.9	0.9	0.6	0.6	0	0
A6NDW3	Cluster of Lipolysis-stimulated lipoprotein receptor	LSR	64 kDa	1	1	1	1.1	0.9	0.9	0.6	0.6	1	1
E7EVY3	Cluster of Calpastatin	CAST	75 kDa	0.9	1	0.9	1	0.9	0.9	0.6	0.6	0	0
J3KQG6	Cluster of Eukaryotic peptide chain release factor GTP-binc	GSPT1	69 kDa	1	1	0.8	1	0.9	0.9	0.6	0.6	0	0
P00390	Glutathione reductase, mitochondrial	GSR	56 kDa	1	1	0.8	1	0.9	0.9	0.6	0.6	0	0
P24592	Insulin-like growth factor-binding protein 6	IGFBP6	25 kDa	1	1	1	0.9	0.9	0.9	0.6	0.6	1	0
Q14683	Structural maintenance of chromosomes protein 1A	SMC1A	143 kDa	1	1	0.9	0.9	0.9	0.9	0.6	0.6	0	0
Q14444	Cluster of Caprin-1	CAPRIN1	78 kDa	1	1	0.9	0.9	0.9	0.9	0.6	0.6	0	0
Q5SSJ5	Cluster of Heterochromatin protein 1-binding protein 3	HP1BP3	61 kDa	1	1	0.9	0.9	0.9	0.9	0.6	0.6	0	0
B4DVY1	Eukaryotic translation initiation factor 3 subunit D	EIF3D	58 kDa	1	1	0.9	0.9	0.9	0.9	0.6	0.6	0	0
F5H2S7	Dynactin subunit 2	DCTN2	45 kDa	1	1	0.7	0.8	0.9	0.9	0.6	0.6	0	0
O60271	Cluster of C-Jun-amino-terminal kinase-interacting protein	SPAG9	146 kDa	1	1	0.7	0.6	0.9	0.9	0.6	0.6	0	1
P42285	Cluster of Superkiller viralicidic activity 2-like 2	SKIV2L2	118 kDa	1	1	0.5	0.6	0.9	0.9	0.6	0.6	0	0
E7EP00	Protein transport protein Sec24C	SEC24C	107 kDa	1	1	0.9	1	0.8	0.9	0.6	0.6	0	0
J3QRS3	Cluster of Myosin regulatory light chain 12A	MYL12A	20 kDa	1	1	0.9	0.8	0.8	0.9	0.6	0.6	0	0
P07954	Fumarate hydratase, mitochondrial	FH	55 kDa	1	1	0.9	0.9	0.9	0.8	0.6	0.6	0	0
P09525	Cluster of Annexin A4	ANXA4	36 kDa	1	1	0.9	0.8	0.9	0.8	0.6	0.6	0	0
P13497	Cluster of Bone morphogenetic protein 1	BMP1	111 kDa	1	1	0.8	0.8	0.9	0.8	0.6	0.6	1	0
P21589	Cluster of 5'-nucleotidase	NT5E	63 kDa	1	1	0.7	0.7	0.9	0.8	0.6	0.6	1	1
Q13217	DnaJ homolog subfamily C member 3	DNAJC3	58 kDa	1	1	0.5	0.6	0.9	0.8	0.6	0.6	1	0
Q13263	Transcription intermediary factor 1-beta	TRIM28	89 kDa	1	1	1.1	1.1	0.8	0.8	0.6	0.6	1	0
Q96FQ6	Protein S100-A16	S100A16	12 kDa	1	1	0.9	0.8	0.8	0.8	0.6	0.6	0	0
Q9UNH7	Cluster of Sorting nexin-6	SNX6	47 kDa	1	1	0.8	0.8	0.8	0.8	0.6	0.6	0	0
O14776	Cluster of Transcription elongation regulator 1	TCERG1	124 kDa	1	1	0.8	0.8	0.8	0.8	0.6	0.6	0	0
O15143	Actin-related protein 2/3 complex subunit 1B	ARPC1B	41 kDa	1	1	0.8	0.8	0.8	0.8	0.6	0.6	0	0
P31947	14-3-3 protein sigma	SFN	28 kDa	1	1	0.8	0.8	0.8	0.8	0.6	0.6	0	0
Q13740	Isoform 2 of CD166 antigen	ALCAM	64 kDa	1	1	0.7	0.8	0.8	0.8	0.6	0.6	0	1
Q16787	Laminin subunit alpha-3	LAMA3	367 kDa	1	1	0.7	0.8	0.8	0.8	0.6	0.6	1	0
Q92973	Transportin-1	TNPO1	102 kDa	1	1	0.6	0.5	0.8	0.8	0.6	0.6	0	0
A6NE09	Protein RPSAP58	RPSAP58	33 kDa	1	1	1.4	1.4	1.6	1.5	7.2	6.7	0	0
D6RAK8	Cluster of Vitamin D-binding protein	GC	55 kDa	1	1	0.7	0.7	0.8	0.7	0.6	0.6	1	0

F8VQZ7	Cluster of Methionine aminopeptidase	METAP2	53 kDa	1	1	0.7	1	0.7	0.7	0.6	0.6	0	0
J3KN36	Cluster of Nodal modulator 3	NOMO3	139 kDa	1	1	0.7	0.9	0.7	0.7	0.6	0.6	1	1
P62750	Cluster of 60S ribosomal protein L23a	RPL23A	18 kDa	1	1	0.7	0.7	0.7	0.7	0.6	0.6	0	0
Q6UVK1	Chondroitin sulfate proteoglycan 4	CSPG4	251 kDa	1	1	0.6	0.6	0.7	0.7	0.6	0.6	0	1
Q9UBQ5	Eukaryotic translation initiation factor 3 subunit K	EIF3K	25 kDa	1	1	0.8	0.9	0.6	0.6	0.6	0.6	0	0
Q9Y383	Cluster of Putative RNA-binding protein Luc7-like 2	LUC7L2	47 kDa	1	1	1.5	1.9	1.6	1.8	0.5	0.6	0	0
B1AHL2	Cluster of Fibulin 1	FBLN1	78 kDa	1	1	1.7	1.8	1.3	1.4	0.5	0.6	1	0
J3KTL2	Cluster of Serine/arginine-rich-splicing factor 1	SRSF1	28 kDa	1	1	1	1	1.4	1.3	0.5	0.6	0	0
P50914	60S ribosomal protein L14	RPL14	23 kDa	1	1	1.3	1.3	1.3	1.3	0.5	0.6	0	0
P80303	Cluster of Nucleobindin-2	NUCB2	50 kDa	1	1	1.2	1.3	1.2	1.3	0.5	0.6	1	0
Q99623	Cluster of Prohibitin-2	PHB2	33 kDa	1	1	0.8	1.1	1	1.3	0.5	0.6	0	0
Q9HAV4	Exportin-5	XPO5	136 kDa	1	1	0.8	1	1	1.3	0.5	0.6	0	0
E7ETZ4	Basic leucine zipper and W2 domain-containing protein 2 (I	BZW2	47 kDa	1	1	1.3	1.5	1.1	1.2	0.5	0.6	0	0
O60701	UDP-glucose 6-dehydrogenase	UGDH	55 kDa	1	1	0.8	1	1.1	1.2	0.5	0.6	0	0
O75882	Cluster of Isoform 2 of Attractin	ATRN	141 kDa	1	1	0.9	0.9	1.1	1.2	0.5	0.6	0	1
P28482	Cluster of Mitogen-activated protein kinase 1	MAPK1	41 kDa	1	1	0.9	1	0.9	1.2	0.5	0.6	0	0
P31431	Cluster of Syndecan-4	SDC4	22 kDa	1	1	1.1	1	1.2	1.1	0.5	0.6	0	1
P51149	Cluster of Ras-related protein Rab-7a	RAB7A	23 kDa	1	1	0.9	1	1.2	1.1	0.5	0.6	0	0
P78417	Glutathione S-transferase omega-1	GSTO1	28 kDa	1	1	1.3	1.1	1.1	1.1	0.5	0.6	0	0
Q15008	Cluster of 26S proteasome non-ATPase regulatory subunit (	PSMD6	46 kDa	1	1	1.2	1.1	1.1	1.1	0.5	0.6	0	0
Q99436	Proteasome subunit beta type-7	PSMB7	30 kDa	1	1	0.9	0.9	1.1	1.1	0.5	0.6	0	0
O75436	Cluster of Vacuolar protein sorting-associated protein 26A	VPS26A	38 kDa	1	1	0.8	0.9	1	1.1	0.5	0.6	0	0
P50570	Cluster of Dynamin-2	DNM2	98 kDa	1	1	1.4	1.8	0.9	1.1	0.5	0.6	0	0
Q13751	Laminin subunit beta-3	LAMB3	130 kDa	1	1	0.9	0.9	0.9	1.1	0.5	0.6	1	0
Q8N1G4	Leucine-rich repeat-containing protein 47	LRRC47	63 kDa	1	1	0.8	0.7	0.8	1.1	0.5	0.6	0	0
Q96P70	Importin-9	IPO9	116 kDa	1	1	1	1	1	1	0.5	0.6	1	0
Q9BRK5	Cluster of 45 kDa calcium-binding protein	SDF4	42 kDa	1	1	1	1	1	1	0.5	0.6	1	0
A8MZ87	Cluster of Kinesin light chain 2	KLC2	57 kDa	1	1	0.8	0.9	0.9	1	0.5	0.6	0	0
P09960	Leukotriene A-4 hydrolase	LTA4H	69 kDa	1	1	0.8	0.8	0.9	1	0.5	0.6	0	0
P16035	Metalloproteinase inhibitor 2	TIMP2	24 kDa	1	1	1	0.9	0.8	1	0.5	0.6	1	0
Q00653	Cluster of Isoform 4 of Nuclear factor NF-kappa-B p100 su	NFKB2	97 kDa	1	1	0.9	0.8	0.8	1	0.5	0.6	0	0
Q9H4M9	Cluster of EH domain-containing protein 1	EHD1	61 kDa	1	1	1.5	1.4	1	0.9	0.5	0.6	0	0
Q9HCE1	Putative helicase MOV-10	MOV10	114 kDa	1	1	0.7	0.8	1	0.9	0.5	0.6	0	0
Q9NR30	Cluster of Nucleolar RNA helicase 2	DDX21	87 kDa	1	1	1	1.1	0.9	0.9	0.5	0.6	0	0
Q9Y2L1	Exosome complex exonuclease RRP44	DIS3	109 kDa	1	1	0.7	1	0.9	0.9	0.5	0.6	0	0
P08621	U1 small nuclear ribonucleoprotein 70 kDa	SNRNP70	52 kDa	1	1	0.8	0.8	0.9	0.9	0.5	0.6	0	0
P30050	60S ribosomal protein L12	RPL12	18 kDa	1	1	0.9	1	0.8	0.9	0.5	0.6	0	0

P36871	Phosphoglucomutase-1	PGM1	61 kDa	1	1	0.8	0.8	0.8	0.9	0.5	0.6	0	0
P60981	Destrin	DSTN	19 kDa	1	1	1	1.1	0.8	0.8	0.5	0.6	0	0
Q8N392	Rho GTPase-activating protein 18	ARHGAP18	75 kDa	1	1	0.8	0.7	0.8	0.8	0.5	0.6	0	0
Q8NC51	Isoform 2 of Plasminogen activator inhibitor 1 RNA-bindin	SERBP1	44 kDa	1	1	0.8	0.9	0.7	0.8	0.5	0.6	0	0
Q8TAT6	Cluster of Nuclear protein localization protein 4 homolog	NPLOC4	68 kDa	1	1	0.8	1	0.7	0.7	0.5	0.6	0	0
Q9BT78	COP9 signalosome complex subunit 4	COPS4	46 kDa	1	1	0.6	0.7	0.6	0.6	0.5	0.6	0	0
A8K3Z3	Cluster of 26S protease regulatory subunit 8	PSMC5	45 kDa	1	1	0.9	0.9	1	1	0.5	0.5	0	0
O14936	Cluster of Isoform 3 of Peripheral plasma membrane protei	CASK	102 kDa	No Va	No Va	No Value	No Value	No Value	No Value	No Val	No Val	0	1
O75131	Copine-3	CPNE3	60 kDa	1	1	1.2	1.4	1.1	1.3	0.4	0.6	0	0
P13611	Cluster of Isoform Vint of Versican core protein	VCAN	370 kDa	1	1	1	1.1	1.1	1.2	0.3	0.4	1	0
P28070	Proteasome subunit beta type-4	PSMB4	29 kDa	1	1	0.8	1.1	1	1	0.4	0.6	0	0
P39748	Cluster of Flap endonuclease 1	FEN1	43 kDa	1	1	0.9	0.9	1.1	0.9	0.4	0.6	0	0
Q01581	Cluster of Hydroxymethylglutaryl-CoA synthase, cytoplasm	HMGCS1	57 kDa	1	1	1	0.9	0.9	0.9	0.4	0.6	0	0
Q8IUL8	Cartilage intermediate layer protein 2	CILP2	126 kDa	1	1	0.8	0.8	0.8	0.9	0.4	0.6	1	0
Q9NQP4	Prefoldin subunit 4	PFDN4	15 kDa	1	1	0.8	1	0.7	0.9	0.4	0.6	0	0
Q9UM54	Cluster of Isoform 6 of Unconventional myosin-VI	MYO6	149 kDa	1	1	0.9	0.8	0.8	0.8	0.4	0.6	0	0
P04632	Cluster of Calpain small subunit 1	CAPNS1	28 kDa	1	1	0.8	0.7	0.8	0.8	0.4	0.6	0	0
P07339	Cluster of Cathepsin D	CTSD	45 kDa	1	1	0.6	0.6	0.7	0.8	0.1	0.1	1	0
P28838	Cluster of Cytosol aminopeptidase	LAP3	56 kDa	1	1	1.3	1.3	1.2	1.1	1.4	1.3	0	0
P34897	Cluster of Serine hydroxymethyltransferase, mitochondrial	SHMT2	56 kDa	1	1	0.7	0.8	0.8	0.8	0.6	0.6	0	0
P49721	Proteasome subunit beta type-2	PSMB2	23 kDa	1	1	1.2	1.1	1	1	1.2	1.1	0	0
Q09161	Nuclear cap-binding protein subunit 1	NCBP1	92 kDa	1	1	0.9	0.9	0.9	0.9	0.7	0.7	0	0
Q7L1Q6	Cluster of Isoform 4 of Basic leucine zipper and W2 domai	BZW1	49 kDa	1	1	1.2	1.4	1.2	1.3	0.8	0.9	0	0
A0AVT1	Cluster of Ubiquitin-like modifier-activating enzyme 6	UBA6	118 kDa	1	1	1.2	1.4	1.1	1.3	0.4	0.6	0	0
B4DR31	Cluster of Dihydropyrimidinase-related protein 2	DPYSL2	58 kDa	1	1	1.3	1.4	1.1	1.1	1.2	1.2	0	0
B4DWJ2	Cluster of Glutamine--tRNA ligase	QARS	87 kDa	1	1	0.9	1	0.9	1	1	1	1	0
E7EU96	Casein kinase II subunit alpha	CSNK2A1	45 kDa	1	1	1	1.2	1	1.1	0.8	0.9	0	0
H0Y4R1	Inosine-5'-monophosphate dehydrogenase 2 (Fragment)	IMPDH2	51 kDa	1	1	1.1	1.1	1.1	1.1	0.6	0.6	0	0
H3BR01	Cluster of Kunitz-type protease inhibitor 1 (Fragment)	SPINT1	27 kDa	1	1	0.6	0.7	0.9	0.8	0.8	0.7	1	1
P27797	Calreticulin	CALR	48 kDa	1	1	1.3	0.9	1	1	1.1	0.7	1	0
P78504	Cluster of Protein jagged-1	JAG1	134 kDa	1	1	0.9	0.9	0.9	0.9	0.7	0.7	0	1
Q14566	DNA replication licensing factor MCM6	MCM6	93 kDa	1	1	1	0.7	1	0.9	1.1	0.7	0	0
Q8NCA5	Cluster of Protein FAM98A	FAM98A	55 kDa	1	1	1.3	0.8	1.1	0.8	1.1	0.7	0	0
Q9GZM7	Cluster of Tubulointerstitial nephritis antigen-like	TINAGL1	52 kDa	1	1	2	1.8	2	2	1.4	1.4	1	0
A6NEM2	HCF N-terminal chain 2	HCFC1	213 kDa	1	1	0.9	0.9	0.8	0.7	1.1	1.2	0	0
F5H7N9	Cluster of Lactadherin	MFGE8	42 kDa	1	1	1.8	1.4	1.6	1.4	1	0.7	1	0
O14792	Heparan sulfate glucosamine 3-O-sulfotransferase 1	HS3ST1	36 kDa	1	1	1	0.9	1.1	1.2	1	0.7	1	0

O60437	Periplakin	PPL	205 kDa	1	1	1.2	1.1	1.1	1.1	1	0.7	0	0
P10599	Thioredoxin	TXN	12 kDa	1	1	0.8	0.9	0.8	1.1	1	0.7	0	0
P50453	Serpin B9	SERPINB9	42 kDa	1	1	1.2	1.1	1.1	1	1	0.7	0	0
P55010	Cluster of Eukaryotic translation initiation factor 5	EIF5	49 kDa	1	1	1.4	1.1	1	1	1	0.7	0	0
P62829	Cluster of 60S ribosomal protein L23	RPL23	15 kDa	1	1	1	0.9	0.9	0.8	1	0.7	0	0
Q00341	Cluster of Vigilin	HDLBP	141 kDa	1	1	0.7	0.8	0.7	0.8	1	0.7	0	0
Q99598	Cluster of Translin-associated protein X	TSNAX	33 kDa	1	1	1.3	0.8	0.9	0.7	1	0.7	0	0
Q9UNF0	Protein kinase C and casein kinase substrate in neurons prot	PACSIN2	56 kDa	1	1	0.6	0.6	0.5	0.7	1	0.7	0	0
A6NCK0	Cluster of NEDD8-activating enzyme E1 regulatory subunit	NAE1	61 kDa	1	1	1.9	1.3	1.5	1.6	0.9	0.7	0	0
B1AKJ5	Nardilysin	NRD1	139 kDa	1	1	1.4	1.8	1.3	1.6	0.9	0.7	0	0
E7EMI0	Cluster of Argininosuccinate lyase	ASL	50 kDa	1	1	1.3	0.9	1.4	1.4	0.9	0.7	0	0
F5H365	Cluster of Protein transport protein Sec23A	SEC23A	83 kDa	1	1	1	0.9	1.5	1.2	0.9	0.7	0	0
Q14103	Cluster of Isoform 3 of Heterogeneous nuclear ribonucleopr	HNRNPD	33 kDa	1	1	1.2	1	1.4	1.2	0.9	0.7	0	0
Q15833	Syntaxin-binding protein 2	STXBP2	66 kDa	1	1	1.2	1	1.3	1.1	0.9	0.7	0	0
Q8TBC4	Cluster of NEDD8-activating enzyme E1 catalytic subunit	UBA3	52 kDa	1	1	1	0.9	0.7	0.7	0.8	0.8	0	0
Q92696	Cluster of Geranylgeranyl transferase type-2 subunit alpha	RABGGTA	65 kDa	1	1	1.3	0.8	1.6	1	0.9	0.7	0	0
Q9H3S7	Tyrosine-protein phosphatase non-receptor type 23	PTPN23	179 kDa	1	1	0.9	0.8	1.2	1	0.9	0.7	0	0
E7ESC6	Cluster of Exportin-7	XPO7	124 kDa	1	1	1.3	1.2	1.1	1	0.9	0.7	0	1
J3KQB0	THUMP domain-containing protein 1	THUMPD1	49 kDa	1	1	1.1	1	1.1	1	0.9	0.7	0	0
O60749	Cluster of Sorting nexin-2	SNX2	58 kDa	1	1	1	1	1.1	1	0.9	0.7	0	0
P38606	V-type proton ATPase catalytic subunit A	ATP6V1A	68 kDa	1	1	0.8	0.8	1	1	0.9	0.7	0	0
P48147	Prolyl endopeptidase	PREP	81 kDa	1	1	1	0.9	0.9	1	0.9	0.7	0	0
P62906	60S ribosomal protein L10a	RPL10A	25 kDa	1	1	1	0.8	0.8	1	0.9	0.7	0	0
Q08752	Cluster of Peptidyl-prolyl cis-trans isomerase D	PPID	41 kDa	1	1	1.5	1	1.4	0.9	0.9	0.7	0	0
Q24JP5	Cluster of Transmembrane protein 132A	TMEM132A	110 kDa	1	1	1	0.9	1	0.9	0.9	0.7	0	1
Q8WVY7	Ubiquitin-like domain-containing CTD phosphatase 1	UBLCP1	37 kDa	1	1	0.8	0.8	1	0.8	0.9	0.7	0	0
Q92688	Acidic leucine-rich nuclear phosphoprotein 32 family meml	ANP32B	29 kDa	1	1	0.9	0.9	0.9	0.8	0.9	0.7	0	0
Q9Y5L0	Cluster of Transportin-3	TNPO3	104 kDa	1	1	0.7	0.7	0.9	0.8	0.9	0.7	0	0
B5MDF5	GTP-binding nuclear protein Ran	RAN	26 kDa	1	1	1.2	1	0.9	0.7	0.9	0.7	0	0
O14744	Protein arginine N-methyltransferase 5	PRMT5	73 kDa	1	1	0.8	0.7	0.6	0.6	0.9	0.7	0	0
O15031	Plexin-B2	PLXNB2	205 kDa	1	1	1.7	1.6	1.6	1.7	0.8	0.7	1	1
P01031	Complement C5	C5	188 kDa	1	1	1.2	0.9	1.2	1.7	0.8	0.7	3	0
P15880	Cluster of 40S ribosomal protein S2	RPS2	31 kDa	1	1	1.4	1.3	1.5	1.4	0.8	0.7	0	0
P18754	Cluster of Isoform 2 of Regulator of chromosome condensa	RCC1	48 kDa	1	1	1.4	1.3	1.4	1.4	0.8	0.7	0	0
P23921	Ribonucleoside-diphosphate reductase large subunit	RRM1	90 kDa	1	1	1.3	1.2	1.4	1.3	0.8	0.7	0	0
P30622	Cluster of Isoform 3 of CAP-Gly domain-containing linker	CLIP1	157 kDa	No Va	No Va	No Value	No Value	No Value	No Value	No Val	No Val	0	0
Q08378	Golgin subfamily A member 3	GOLGA3	167 kDa	1	1	1	1.1	1.3	1.3	0.8	0.7	0	0

Q5JPT4	SH3 domain-containing kinase-binding protein 1	SH3KBP1	47 kDa	1	1	1.3	1.2	1.2	1.3	0.8	0.7	0	0
Q86X55	Histone-arginine methyltransferase CARM1	CARM1	66 kDa	1	1	1	1.2	1.2	1.2	0.8	0.7	1	0
Q9UBE0	Cluster of SUMO-activating enzyme subunit 1	SAE1	38 kDa	1	1	1.2	1.2	1	1.2	0.8	0.7	0	0
H3BQZ7	HCG2044799	HNRNPUL2-I	85 kDa	1	1	0.9	0.9	1	1.2	0.8	0.7	0	0
P36578	Cluster of 60S ribosomal protein L4	RPL4	48 kDa	1	1	0.9	0.8	1.3	1.1	0.8	0.7	0	0
P37235	Cluster of Hippocalcin-like protein 1	HPCAL1	22 kDa	1	1	1.4	1.1	1.2	1.1	0.8	0.7	0	0
Q04917	Cluster of 14-3-3 protein eta	YWHAH	28 kDa	1	1	1.2	1.1	1	1.1	0.8	0.7	0	0
Q9BY44	Cluster of Eukaryotic translation initiation factor 2A	EIF2A	65 kDa	1	1	1.2	1	1.2	1	0.8	0.7	0	0
Q9H3U1	Isoform 2 of Protein unc-45 homolog A	UNC45A	102 kDa	1	1	1.2	1.2	1.1	1	0.8	0.7	0	0
O43143	Putative pre-mRNA-splicing factor ATP-dependent RNA he	DHX15	91 kDa	1	1	1.1	1.3	1	1	0.8	0.7	0	0
O75326	Semaphorin-7A	SEMA7A	75 kDa	1	1	1.1	1.2	1	1	0.8	0.7	0	1
P05141	Cluster of ADP/ATP translocase 2	SLC25A5	33 kDa	1	1	0.8	0.8	1	1	0.8	0.7	0	1
P22694	Cluster of cAMP-dependent protein kinase catalytic subunit	PRKACB	41 kDa	1	1	0.8	0.8	1	1	0.8	0.7	0	0
P47755	F-actin-capping protein subunit alpha-2	CAPZA2	33 kDa	1	1	0.9	0.9	0.8	1	0.8	0.7	0	0
Q12996	Cleavage stimulation factor subunit 3	CSTF3	83 kDa	1	1	1	0.9	1	0.9	0.8	0.7	0	0
Q92520	Cluster of Protein FAM3C	FAM3C	25 kDa	1	1	1	0.9	1	0.9	0.8	0.7	0	0
Q93008	Cluster of Probable ubiquitin carboxyl-terminal hydrolase F	USP9X	292 kDa	1	1	1	0.9	1	0.9	0.8	0.7	0	0
Q9C0C9	Ubiquitin-conjugating enzyme E2 O	UBE2O	141 kDa	1	1	0.9	0.8	1	0.9	0.8	0.7	0	0
Q9H173	Nucleotide exchange factor SIL1	SIL1	52 kDa	1	1	1	1.3	0.9	0.9	0.8	0.7	1	0
B7Z3X4	Cluster of Glucosamine-6-phosphate isomerase 1	GNPDA1	24 kDa	1	1	1	1	0.9	0.9	0.8	0.7	0	0
E9PHK9	Cluster of Treacle protein	TCOF1	156 kDa	1	1	0.9	1	0.9	0.9	0.8	0.7	0	0
H7C2W3	Cluster of Protein transport protein Sec31A	SEC31A	131 kDa	1	1	1.1	0.9	0.9	0.9	0.8	0.7	1	0
P09622	Cluster of Dihydrolipoyl dehydrogenase, mitochondrial	DLD	54 kDa	1	1	0.9	0.9	0.9	0.9	0.8	0.7	0	0
P55036	Cluster of 26S proteasome non-ATPase regulatory subunit 4	PSMD4	41 kDa	1	1	0.8	0.8	0.8	0.9	0.8	0.7	0	0
Q09028	Cluster of Histone-binding protein RBBP4	RBBP4	48 kDa	1	1	0.9	0.9	0.7	0.9	0.8	0.7	0	0
Q13185	Cluster of Chromobox protein homolog 3	CBX3	21 kDa	1	1	1.1	1	1.3	0.8	0.8	0.7	0	0
Q7Z304	Cluster of MAM domain-containing protein 2	MAMDC2	78 kDa	1	1	1.1	1.1	1	0.8	0.8	0.7	3	0
Q96KR1	Cluster of Zinc finger RNA-binding protein	ZFR	117 kDa	1	1	1	0.9	1	0.8	0.8	0.7	0	0
Q9UHD8	Cluster of Septin-9	SEPT9	65 kDa	No Va	No Va	No Value	No Value	No Value	No Value	No Val	No Val	1	0
Q9Y2T3	Cluster of Guanine deaminase	GDA	51 kDa	1	1	1	0.9	1	0.8	0.8	0.7	0	0
Q9Y376	Calcium-binding protein 39	CAB39	40 kDa	1	1	1	0.8	1	0.8	0.8	0.7	0	0
E7EWL7	Cluster of Disks large homolog 1	DLG1	88 kDa	1	1	0.9	0.8	1	0.8	0.8	0.7	0	0
J3QT22	Cluster of Protein phosphatase methylesterase 1	PPME1	44 kDa	1	1	0.8	1	0.9	0.8	0.8	0.7	0	0
P13489	Cluster of Ribonuclease inhibitor	RNH1	50 kDa	1	1	0.9	0.9	0.9	0.8	0.8	0.7	0	0
P49862	Kallikrein-7	KLK7	28 kDa	1	1	0.6	0.7	0.9	0.8	0.8	0.7	1	0
P53041	Cluster of Serine/threonine-protein phosphatase 5	PPP5C	57 kDa	1	1	0.9	1	0.8	0.8	0.8	0.7	0	0
Q14126	Desmoglein-2	DSG2	122 kDa	1	1	0.6	0.6	0.8	0.8	0.8	0.7	1	1

Q9NTX5	Cluster of Ethylmalonyl-CoA decarboxylase	ECHDC1	34 kDa	1	1	0.9	0.9	0.7	0.8	0.8	0.7	1	0
Q9UKD2	mRNA turnover protein 4 homolog	MRTO4	28 kDa	1	1	0.7	0.7	0.7	0.7	0.8	0.7	0	0
Q9UMS4	Cluster of Pre-mRNA-processing factor 19	PRPF19	55 kDa	1	1	0.6	0.6	0.6	0.7	0.8	0.7	0	0
Q9Y4K0	Cluster of Lysyl oxidase homolog 2	LOXL2	87 kDa	1	1	0.9	0.7	0.8	0.6	0.8	0.7	1	0
O43818	U3 small nucleolar RNA-interacting protein 2	RRP9	52 kDa	1	1	0.7	0.8	0.6	0.6	0.8	0.7	0	0
P19367	Cluster of Isoform 4 of Hexokinase-1	HK1	101 kDa	No Va	No Va	No Value	No Value	No Value	No Value	No Val	No Val	0	0
P25398	40S ribosomal protein S12	RPS12	15 kDa	1	1	1.4	1.3	2.2	2	0.7	0.7	0	0
P62249	40S ribosomal protein S16	RPS16	16 kDa	1	1	1.8	1.5	1.9	1.7	0.7	0.7	0	0
Q05048	Cleavage stimulation factor subunit 1	CSTF1	48 kDa	1	1	1	1.4	1.3	1.7	0.7	0.7	0	0
Q9UNS2	Cluster of Isoform 2 of COP9 signalosome complex subunit	COPS3	46 kDa	1	1	1.4	1.7	1.7	1.6	0.7	0.7	0	0
P62308	Cluster of Small nuclear ribonucleoprotein G	SNRPG	8 kDa	1	1	1.3	1.4	1.2	1.6	0.7	0.7	0	0
G3XAH6	Cluster of Poly(A) polymerase alpha	PAPOLA	81 kDa	1	1	2.1	1.9	1.4	1.5	0.7	0.7	0	1
J3QRZ5	Ubiquitin carboxyl-terminal hydrolase	USP14	52 kDa	1	1	1.5	1.6	1.5	1.4	0.7	0.7	1	0
O75367	Core histone macro-H2A.1	H2AFY	40 kDa	1	1	1.1	1.2	1	1.4	0.7	0.7	0	0
P01008	Antithrombin-III	SERPINC1	53 kDa	1	1	1.3	1.3	1.5	1.3	0.7	0.7	3	0
P04792	Cluster of Heat shock protein beta-1	HSPB1	23 kDa	1	1	1.4	1.3	1.3	1.3	0.7	0.7	0	0
P17980	Cluster of 26S protease regulatory subunit 6A	PSMC3	49 kDa	1	1	1.6	1.6	1.2	1.3	0.7	0.7	0	0
P61201	COP9 signalosome complex subunit 2	COPS2	52 kDa	1	1	1.2	1.1	1.2	1.3	0.7	0.7	0	0
C9JW96	Prohibitin (Fragment)	PHB	27 kDa	1	1	1	1.1	1.2	1.3	0.7	0.7	1	0
H3BP20	Cluster of Beta-hexosaminidase subunit alpha	HEXA	62 kDa	1	1	0.9	1	1.2	1.3	0.7	0.7	1	0
J3QQ67	60S ribosomal protein L18 (Fragment)	RPL18	22 kDa	1	1	1	1.2	1.1	1.3	0.7	0.7	0	0
O95407	Tumor necrosis factor receptor superfamily member 6B	TNFRSF6B	33 kDa	1	1	0.9	1.1	1	1.3	0.7	0.7	3	0
P00367	Cluster of Glutamate dehydrogenase 1, mitochondrial	GLUD1	61 kDa	1	1	1	1	1.3	1.2	0.7	0.7	1	0
P46781	40S ribosomal protein S9	RPS9	23 kDa	1	1	0.8	0.8	1.3	1.2	0.7	0.7	0	0
P54727	Cluster of UV excision repair protein RAD23 homolog B	RAD23B	43 kDa	1	1	1.2	1.2	1.2	1.2	0.7	0.7	0	0
Q00839	Cluster of Heterogeneous nuclear ribonucleoprotein U	HNRNPU	91 kDa	1	1	1.2	1.1	1.2	1.2	0.7	0.7	0	0
Q12874	Splicing factor 3A subunit 3	SF3A3	59 kDa	1	1	0.8	0.8	1.2	1.2	0.7	0.7	0	0
Q99615	DnaJ homolog subfamily C member 7	DNAJC7	56 kDa	1	1	1.1	1.2	1.1	1.2	0.7	0.7	0	0
Q9C0B1	Cluster of Alpha-ketoglutarate-dependent dioxygenase FTO	FTO	58 kDa	1	1	1.1	1.2	1.1	1.2	0.7	0.7	0	0
B1AH89	Tubulin tyrosine ligase-like family, member 12	TTLL12	74 kDa	1	1	1	1.1	1.1	1.2	0.7	0.7	0	0
B4DT77	Annexin	ANXA7	38 kDa	1	1	1	1.2	0.9	1.2	0.7	0.7	0	0
O00629	Cluster of Importin subunit alpha-4	KPNA4	58 kDa	1	1	1.3	1.6	1.2	1.1	0.7	0.7	0	0
O43396	Cluster of Thioredoxin-like protein 1	TXNL1	32 kDa	1	1	1.1	1.1	1.2	1.1	0.7	0.7	0	0
P12110	Cluster of Collagen alpha-2(VI) chain	COL6A2	109 kDa	1	1	1	1	1.2	1.1	0.7	0.7	1	0
P14678	Cluster of Isoform SM-B1 of Small nuclear ribonucleoprote	SNRPB	30 kDa	1	1	1.1	1.3	1.1	1.1	0.7	0.7	0	0
P17931	Cluster of Galectin-3	LGALS3	26 kDa	1	1	1.2	1.2	1.1	1.1	0.7	0.7	0	0
P25787	Proteasome subunit alpha type-2	PSMA2	26 kDa	1	1	1.2	1.1	1.1	1.1	0.7	0.7	0	0

P26006	Cluster of Integrin alpha-3	ITGA3	117 kDa	1	1	1.1	1.1	1.1	1.1	0.7	0.7	1	1
P52788	Cluster of Spermine synthase	SMS	41 kDa	1	0.9	1	1	1.1	1.1	0.7	0.7	0	0
Q5T8U3	Cluster of 60S ribosomal protein L7a (Fragment)	RPL7A	22 kDa	1	1	1	0.8	1.1	1.1	0.7	0.7	0	0
Q7L2H7	Eukaryotic translation initiation factor 3 subunit M	EIF3M	43 kDa	1	1	0.6	0.6	0.5	0.4	0.1	0.1	0	0
Q92876	Kallikrein-6	KLK6	27 kDa	1	1	0.8	0.8	1.1	1.1	0.7	0.7	1	0
Q9H3G5	Cluster of Probable serine carboxypeptidase CPVL	CPVL	54 kDa	1	1	1.1	1.1	1	1.1	0.7	0.7	1	0
Q9P265	Cluster of Disco-interacting protein 2 homolog B	DIP2B	171 kDa	1	1	0.8	0.9	1	1.1	0.7	0.7	0	0
B4DVB8	Cluster of ELAV-like protein 1	ELAVL1	39 kDa	1	1	0.8	0.8	1	1.1	0.7	0.7	0	0
E7EUU1	Cluster of Latent-transforming growth factor beta-binding p	LTBP4	166 kDa	1	1	0.8	0.8	1	1.1	0.7	0.7	1	0
F5H667	Aspartyl/asparaginyl beta-hydroxylase	ASPH	83 kDa	1	1	0.9	0.8	0.9	1.1	0.7	0.7	0	1
O95777	Cluster of N-alpha-acetyltransferase 38, NatC auxiliary sub	NAA38	10 kDa	1	1	0.7	1	1	0.9	1.9	1.6	0	0
P17301	Cluster of Integrin alpha-2	ITGA2	129 kDa	1	1	1.3	1.2	1.2	1	0.7	0.7	1	1
Q9BS26	Endoplasmic reticulum resident protein 44	ERP44	47 kDa	1	1	1.3	1.2	1.2	1	0.7	0.7	3	0
Q9BXS5	AP-1 complex subunit mu-1	AP1M1	49 kDa	1	1	1.3	1	1.2	1	0.7	0.7	0	0
Q9HAV7	GrpE protein homolog 1, mitochondrial	GRPEL1	24 kDa	1	1	1.1	1.1	1.1	1	0.7	0.7	0	0
E9PEP6	Cluster of Neuropilin-1	NRP1	101 kDa	1	1	1.1	1.1	1.1	1	0.7	0.7	1	1
F8VPV9	Cluster of ATP synthase subunit beta	ATP5B	55 kDa	1	1	1	1.1	1.1	1	0.7	0.7	0	0
O15067	Cluster of Phosphoribosylformylglycinamidine synthase	PFAS	145 kDa	1	1	1.1	1	1.1	1	0.7	0.7	0	0
O60664	Cluster of Perilipin-3	PLIN3	47 kDa	1	1	0.9	1	1.1	1	0.7	0.7	0	0
O76094	Cluster of Signal recognition particle 72 kDa protein	SRP72	75 kDa	1	1	1.2	1.3	1	1	0.7	0.7	0	0
P00492	Hypoxanthine-guanine phosphoribosyltransferase	HPRT1	25 kDa	1	1	1.2	1.2	1	1	0.7	0.7	0	0
Q13177	Cluster of Serine/threonine-protein kinase PAK 2	PAK2	58 kDa	1	1	1.1	1.2	1	1	0.7	0.7	0	0
Q13363	Cluster of C-terminal-binding protein 1	CTBP1	48 kDa	1	1	1.2	1.1	1	1	0.7	0.7	0	0
Q92616	Translational activator GCN1	GCN1L1	293 kDa	1	1	1.1	1.1	1	1	0.7	0.7	0	0
Q9NPH2	Inositol-3-phosphate synthase 1	ISYNA1	61 kDa	1	1	1.1	1.1	1	1	0.7	0.7	0	0
Q9NZL9	Cluster of Methionine adenosyltransferase 2 subunit beta	MAT2B	38 kDa	1	1	1	1.1	1	1	0.7	0.7	0	0
Q9Y295	Cluster of Developmentally-regulated GTP-binding protein	DRG1	41 kDa	1	1	1	1.1	1	1	0.7	0.7	0	0
E7EM64	Cluster of COP9 signalosome complex subunit 6	COPS6	36 kDa	1	1	1.1	0.9	1	1	0.7	0.7	1	0
H3BVG0	Cluster of Nuclear pore complex protein Nup93	NUP93	100 kDa	1.1	1	1	0.9	1	1	0.7	0.7	0	1
O00469	Isoform 2 of Procollagen-lysine,2-oxoglutarate 5-dioxygena	PLOD2	87 kDa	1	1	0.9	0.9	1	1	0.7	0.7	1	1
P00750	Cluster of Tissue-type plasminogen activator	PLAT	63 kDa	1	1	1	0.8	1	1	0.7	0.7	1	0
P49458	Cluster of Signal recognition particle 9 kDa protein	SRP9	10 kDa	1	1	1.1	1.2	0.9	1	0.7	0.7	0	0
P51991	Cluster of Heterogeneous nuclear ribonucleoprotein A3	HNRNPA3	40 kDa	1	1	0.5	0.8	0.9	1	0.7	0.7	0	0
P78539	Sushi repeat-containing protein SRPX	SRPX	52 kDa	1	1	0.8	1.1	0.8	1	0.7	0.7	1	0
P05386	60S acidic ribosomal protein P1	RPLP1	12 kDa	1	1	0.9	0.9	0.8	1	0.7	0.7	0	0
B4DLW8	Cluster of Probable ATP-dependent RNA helicase DDX5	DDX5	61 kDa	1	1	0.5	0.4	0.6	1	0.7	0.7	0	0
E7ES43	Heat shock 70 kDa protein 4L	HSPA4L	98 kDa	1	1	1.2	1.2	1	0.9	0.7	0.7	0	0

H3BPE7	RNA-binding protein FUS	FUS	53 kDa	1	1	1.1	1	1	0.9	0.7	0.7	0	0
O00560	Syntenin-1	SDCBP	32 kDa	1	1	1	1	1	0.9	0.7	0.7	0	0
Q14764	Major vault protein	MVP	99 kDa	1	1	1	1	1	0.9	0.7	0.7	0	0
Q9NUJ1	Abhydrolase domain-containing protein 10, mitochondrial	ABHD10	34 kDa	1	1	0.9	0.9	1	0.9	0.7	0.7	1	0
P06703	Protein S100-A6	S100A6	10 kDa	1	1	1.1	1.2	0.9	0.9	0.7	0.7	0	0
E7EWS7	Ubiquitin-conjugating enzyme E2 L3	UBE2L3	24 kDa	1	1	1.1	1.1	0.9	0.9	0.7	0.7	0	0
H0Y5F5	Cluster of Polyadenylate-binding protein 4 (Fragment)	PABPC4	60 kDa	1	1	1	1	0.9	0.9	0.7	0.7	0	0
P00734	Prothrombin	F2	70 kDa	1	1	1	1	0.9	0.9	0.7	0.7	1	0
P15927	Isoform 3 of Replication protein A 32 kDa subunit	RPA2	39 kDa	1	1	1	1	0.9	0.9	0.7	0.7	0	0
Q6UXN9	WD repeat-containing protein 82	WDR82	35 kDa	1	1	0.9	1	0.9	0.9	0.7	0.7	0	0
Q99729	Isoform 3 of Heterogeneous nuclear ribonucleoprotein A/B	HNRNPAB	31 kDa	1	1	1.2	0.9	0.9	0.9	0.7	0.7	0	0
Q9BTE3	Cluster of Mini-chromosome maintenance complex-binding	MCMBP	73 kDa	1	1	1	0.9	0.9	0.9	0.7	0.7	0	0
Q9UBB4	Cluster of Ataxin-10	ATXN10	53 kDa	1	1	1	0.9	0.9	0.9	0.7	0.7	0	0
Q9UHB9	Signal recognition particle 68 kDa protein	SRP68	71 kDa	1	1	0.9	0.9	0.9	0.9	0.7	0.7	0	0
Q9Y4E8	Cluster of Ubiquitin carboxyl-terminal hydrolase 15	USP15	112 kDa	1	1	0.9	0.9	0.9	0.9	0.7	0.7	0	0
C9JFE4	Cluster of COP9 signalosome complex subunit 1	GPS1	53 kDa	1	1	0.9	0.9	0.9	0.9	0.7	0.7	0	0
E9PHY5	Cluster of Band 4.1-like protein 2	EPB41L2	104 kDa	1	1	0.9	0.9	0.9	0.9	0.7	0.7	0	0
H0Y930	Extracellular matrix protein FRAS1 (Fragment)	FRAS1	250 kDa	1	1	0.9	0.9	0.9	0.9	0.7	0.7	1	1
H0YLA4	Sorbitol dehydrogenase	SORD	36 kDa	1	1	0.9	0.9	0.9	0.9	0.7	0.7	0	0
Q07021	Complement component 1 Q subcomponent-binding proteir	C1QBP	31 kDa	1	1	0.9	0.9	0.9	0.9	0.7	0.7	0	0
Q96C19	Cluster of EF-hand domain-containing protein D2	EFHD2	27 kDa	1	1	0.8	0.9	0.9	0.9	0.7	0.7	0	0
Q9BUJ2	Heterogeneous nuclear ribonucleoprotein U-like protein 1	HNRNPUL1	96 kDa	1	1	0.9	0.8	0.9	0.9	0.7	0.7	0	0
G8JLH6	CD9 antigen (Fragment)	CD9	25 kDa	1	1	0.9	0.8	0.9	0.9	0.7	0.7	0	1
H0YMB3	Cluster of GMP reductase 2	GMPR2	34 kDa	1	1	0.7	0.8	0.9	0.9	0.7	0.7	0	0
J3QK90	NSFL1 cofactor p47	NSFL1C	41 kDa	1	1	0.7	0.7	0.9	0.9	0.7	0.7	0	0
P10644	Cluster of cAMP-dependent protein kinase type I-alpha regı	PRKAR1A	43 kDa	1	1	0.6	0.7	0.9	0.9	0.7	0.7	0	0
P11274	Cluster of Breakpoint cluster region protein	BCR	143 kDa	1	No Va	0.7	No Value	0.9	No Value	0.8	No Val	0	0
P12259	Coagulation factor V	F5	252 kDa	1	1	0.9	0.8	0.8	0.9	0.7	0.7	1	0
P21399	Cytoplasmic aconitate hydratase	ACO1	98 kDa	1	1	0.8	0.8	0.8	0.9	0.7	0.7	0	0
P55145	Mesencephalic astrocyte-derived neurotrophic factor	MANF	21 kDa	1	1	1.1	1.2	0.7	0.9	0.7	0.7	1	0
P78406	Cluster of mRNA export factor	RAE1	41 kDa	1	1	0.8	0.9	0.7	0.9	0.7	0.7	0	0
Q00325	Phosphate carrier protein, mitochondrial	SLC25A3	40 kDa	1	1	1.4	1.2	0.9	0.8	0.7	0.7	0	1
Q13451	Cluster of Peptidyl-prolyl cis-trans isomerase FKBP5	FKBP5	51 kDa	1	1	1.2	1.1	0.9	0.8	0.7	0.7	0	0
Q86SQ4	Isoform 3 of G-protein coupled receptor 126	GPR126	140 kDa	1	1	1	0.9	0.9	0.8	0.7	0.7	1	1
Q8TAQ2	Cluster of SWI/SNF complex subunit SMARCC2	SMARCC2	133 kDa	1	1	0.9	0.9	0.9	0.8	0.7	0.7	0	0
Q99497	Protein DJ-1	PARK7	20 kDa	1	1	0.9	0.8	0.9	0.8	0.7	0.7	0	0
Q9Y2A7	Nck-associated protein 1	NCKAP1	129 kDa	1	1	0.9	1.1	0.8	0.8	0.7	0.7	0	1

A5YKK6	Cluster of Isoform 4 of CCR4-NOT transcription complex s	CNOT1	174 kDa	1	1	0.9	1	0.8	0.8	0.7	0.7	0	1
G5E9E7	Cluster of Tight junction protein 1 (Zona occludens 1), isof	TJP1	190 kDa	No Va	No Va	No Value	No Value	No Value	No Value	No Val	No Val	0	0
H0YN26	Cluster of Acidic leucine-rich nuclear phosphoprotein 32 fa	ANP32A	20 kDa	1	1	0.8	0.9	0.8	0.8	0.7	0.7	0	0
O00567	Cluster of Nucleolar protein 56	NOP56	66 kDa	1	1	0.9	0.8	0.8	0.8	0.7	0.7	0	0
O76061	Cluster of Stanniocalcin-2	STC2	33 kDa	1	1	0.9	0.8	0.8	0.8	0.7	0.7	1	0
P01034	Cystatin-C	CST3	16 kDa	1	1	0.8	0.8	0.8	0.8	0.7	0.7	1	0
P13521	Secretogranin-2	SCG2	71 kDa	1	1	0.8	0.8	0.8	0.8	0.7	0.7	1	0
P43686	26S protease regulatory subunit 6B	PSMC4	47 kDa	1	1	0.7	0.7	0.7	0.8	0.7	0.7	0	0
P62269	40S ribosomal protein S18	RPS18	18 kDa	1	1	0.8	0.8	0.8	0.7	0.7	0.7	0	0
Q8NBJ4	Cluster of Golgi membrane protein 1	GOLM1	45 kDa	1	1	0.8	0.8	0.7	0.7	0.7	0.7	1	0
Q9Y263	Cluster of Phospholipase A-2-activating protein	PLAA	87 kDa	1	1	0.8	0.8	0.7	0.7	0.7	0.7	0	0
Q9Y3A5	Ribosome maturation protein SBDS	SBDS	29 kDa	1	1	0.9	1	0.6	0.7	0.7	0.7	0	0
Q9Y4X5	E3 ubiquitin-protein ligase ARIH1	ARIH1	64 kDa	1	1	0.8	1	0.6	0.7	0.7	0.7	0	0
P02765	Alpha-2-HS-glycoprotein	AHSG	39 kDa	1	1	0.7	0.8	0.6	0.7	0.7	0.7	1	0
B0QYK1	Ewing sarcoma breakpoint region 1	EWSR1	63 kDa	1	1	0.7	0.6	0.8	0.6	0.7	0.7	0	0
F5H0L8	SEC23-interacting protein	SEC23IP	90 kDa	1	1	0.5	0.5	0.6	0.6	0.7	0.7	0	0
H7C463	Cluster of Mitochondrial inner membrane protein (Fragmen	IMMT	68 kDa	1	1	0.4	0.4	0.6	0.6	0.7	0.7	0	1
O75822	Cluster of Eukaryotic translation initiation factor 3 subunit .	EIF3J	29 kDa	1	1	0.3	0.4	0.3	0.4	0.7	0.7	0	0
P49321	Cluster of Nuclear autoantigenic sperm protein	NASP	85 kDa	1	1	1.5	1.4	1.4	1.9	0.6	0.7	1	0
Q86UY0	TXNDC5 protein	TXNDC5	40 kDa	1	1	1.5	1.5	1.4	1.7	0.6	0.7	1	0
Q8IZ83	Cluster of Aldehyde dehydrogenase family 16 member A1	ALDH16A1	85 kDa	1	1	1.4	1.4	1.5	1.5	0.6	0.7	1	1
C9JK10	Cluster of Integrin alpha-6 (Fragment)	ITGA6	122 kDa	1	1	1.3	1.3	1.5	1.5	0.6	0.7	0	1
J3KR97	Cluster of Tubulin-specific chaperone D	TBCD	137 kDa	1	1	1.1	1.1	1.5	1.5	0.6	0.7	0	1
O00267	Transcription elongation factor SPT5	SUPT5H	121 kDa	1	1	0.9	0.9	1.3	1.5	0.6	0.7	0	0
O75390	Cluster of Citrate synthase, mitochondrial	CS	52 kDa	1	1	1.4	1.4	1.2	1.5	0.6	0.7	0	1
P10253	Lysosomal alpha-glucosidase	GAA	105 kDa	1	1	1.1	1.2	1.1	1.5	0.6	0.7	0	0
P12830	Cluster of Cadherin-1	CDH1	97 kDa	1	1	1.2	1.4	1.3	1.4	0.6	0.7	1	1
P17812	CTP synthase 1	CTPS1	67 kDa	1	1	1.2	1.2	1	1.4	0.6	0.7	0	0
P43243	Cluster of Matrin-3	MATR3	95 kDa	1	1	1.2	1.2	1.3	1.3	0.6	0.7	1	0
P53602	Diphosphomevalonate decarboxylase	MVD	43 kDa	1	1	1	1.2	1	1.2	0.6	0.7	0	1
Q96BP3	Peptidylprolyl isomerase domain and WD repeat-containing	PPWD1	74 kDa	1	1	1	1.1	1.2	1.1	0.6	0.7	0	0
Q9NRR5	Cluster of Ubiquilin-4	UBQLN4	64 kDa	1	1	1.1	1.1	1.1	1.1	0.6	0.7	0	0
D6R9T0	Cluster of Heterogeneous nuclear ribonucleoprotein H, N-te	HNRNPH1	18 kDa	1	1	1	1.1	1.1	1.1	0.6	0.7	0	0
E7ETB3	Cluster of Aspartyl aminopeptidase	DNPEP	55 kDa	1	1	1	0.9	1.1	1.1	0.6	0.7	0	1
F5H1Z7	Semaphorin-3C	SEMA3C	87 kDa	1	1	0.7	0.8	1.1	1.1	0.6	0.7	1	0
O14929	Cluster of Histone acetyltransferase type B catalytic subuni	HAT1	50 kDa	1	1	1	0.9	1	1.1	0.6	0.7	0	0
P30520	Adenylosuccinate synthetase isozyme 2	ADSS	50 kDa	1	1	0.9	1.2	0.9	1.1	0.6	0.7	0	0

P46063	ATP-dependent DNA helicase Q1	RECQL	73 kDa	1	1	0.9	0.9	0.9	1.1	0.6	0.7	0	0
Q15404	Cluster of Ras suppressor protein 1	RSU1	32 kDa	1	1	0.9	1	0.8	1.1	0.6	0.7	0	0
Q15691	Microtubule-associated protein RP/EB family member 1	MAPRE1	30 kDa	1	1	0.8	0.9	0.7	1.1	0.6	0.7	0	0
Q6P3W7	SCY1-like protein 2	SCYL2	104 kDa	1	1	1.3	0.9	1.5	1	0.6	0.7	0	0
Q9NTZ6	RNA-binding protein 12	RBM12	97 kDa	1	1	1.1	0.8	1.4	1	0.6	0.7	0	0
E7ESY4	Cluster of Metastasis-associated protein MTA1	MTA1	79 kDa	1	1	1	1.3	1	1	0.6	0.7	1	0
E9PFW3	AP-2 complex subunit mu	AP2M1	52 kDa	1	1	1.1	1.1	1	1	0.6	0.7	0	0
O14579	Coatomer subunit epsilon	COPE	34 kDa	1	1	1	1.1	1	1	0.6	0.7	0	0
O43684	Mitotic checkpoint protein BUB3	BUB3	37 kDa	1	1	1	1	1	1	0.6	0.7	0	0
O76003	Glutaredoxin-3	GLRX3	37 kDa	1	1	1	1	1	1	0.6	0.7	0	0
P23193	Cluster of Transcription elongation factor A protein 1	TCEA1	34 kDa	1	1	1	1	1	1	0.6	0.7	0	0
P30086	Phosphatidylethanolamine-binding protein 1	PEBP1	21 kDa	1	1	1.1	1.1	0.9	1	0.6	0.7	0	0
P56537	Cluster of Eukaryotic translation initiation factor 6	EIF6	27 kDa	1	1	1	1.1	0.9	1	0.6	0.7	0	0
Q00688	Peptidyl-prolyl cis-trans isomerase FKBP3	FKBP3	25 kDa	1	1	1.2	1	0.9	1	0.6	0.7	0	0
Q03405	Urokinase plasminogen activator surface receptor	PLAUR	37 kDa	1	1	1	0.9	0.9	1	0.6	0.7	1	1
Q14512	Fibroblast growth factor-binding protein 1	FGFBP1	26 kDa	1	1	1.4	1.6	0.8	1	0.6	0.7	1	0
Q14694	Cluster of Isoform 2 of Ubiquitin carboxyl-terminal hydrola	USP10	93 kDa	1	1	0.8	1	0.8	1	0.6	0.7	0	0
Q14C86	Cluster of GTPase-activating protein and VPS9 domain-cor	GAPVD1	165 kDa	1	1	0.8	0.7	0.8	1	0.6	0.7	0	0
Q96HE7	Cluster of ERO1-like protein alpha	ERO1L	54 kDa	1	1	0.8	0.8	1	0.9	0.6	0.7	1	0
Q9Y4B6	Protein VPRBP	VPRBP	169 kDa	1	1	1.2	1.3	0.9	0.9	0.6	0.7	0	0
E9PGF9	Protein O-GlcNAcase	MGEA5	97 kDa	1	1	0.8	1	0.9	0.9	0.6	0.7	0	0
J3QQX2	Cluster of Rho GDP-dissociation inhibitor 1	ARHGDIA	26 kDa	1	1	0.8	0.9	0.9	0.9	0.6	0.7	0	0
O43813	Cluster of LanC-like protein 1	LANCL1	45 kDa	1	1	0.8	0.9	0.7	0.9	0.6	0.7	0	0
P40429	Cluster of 60S ribosomal protein L13a	RPL13A	24 kDa	1	1	0.6	0.9	0.7	0.9	0.6	0.7	0	0
P61011	Signal recognition particle 54 kDa protein	SRP54	56 kDa	1	1	0.9	0.9	0.8	0.8	0.6	0.7	0	0
Q7Z6Z7	Cluster of E3 ubiquitin-protein ligase HUWE1	HUWE1	482 kDa	1	1	1	0.8	0.8	0.8	0.6	0.7	0	1
H0Y360	Cluster of AMP deaminase 2 (Fragment)	AMPD2	99 kDa	1	1	0.8	0.9	0.7	0.8	0.6	0.7	0	0
I3L0H8	Cluster of ATP-dependent RNA helicase DDX19A	DDX19A	51 kDa	1	1	0.9	1	1	0.7	0.6	0.7	0	0
O14964	Hepatocyte growth factor-regulated tyrosine kinase substrat	HGS	86 kDa	1	1	0.6	0.5	0.7	0.7	0.6	0.6	0	0
O60341	Cluster of Lysine-specific histone demethylase 1A	KDM1A	93 kDa	1	1	0.8	0.6	0.6	0.7	0.6	0.7	1	0
P00505	Aspartate aminotransferase, mitochondrial	GOT2	48 kDa	1	1	0.6	0.6	0.5	0.6	0.6	0.7	1	0
P09012	U1 small nuclear ribonucleoprotein A	SNRPA	31 kDa	1	1	0.9	0.9	1.1	1	1.9	1.9	0	0
P20042	Eukaryotic translation initiation factor 2 subunit 2	EIF2S2	38 kDa	1	1	1.6	1.6	0.9	1.4	0.5	0.7	0	0
P33992	Cluster of DNA replication licensing factor MCM5	MCM5	82 kDa	1	1	1.6	1.5	1.5	1.3	0.5	0.7	0	0
P61353	60S ribosomal protein L27	RPL27	16 kDa	1	1	1.4	1.3	1.3	1.3	0.5	0.7	0	0
P61764	Cluster of Syntaxin-binding protein 1	STXBP1	68 kDa	No Va	1	No Value	0.8	No Value	0.9	No Val	0.9	0	0
Q04721	Cluster of Neurogenic locus notch homolog protein 2	NOTCH2	265 kDa	1	1	1	1	1.3	1.3	0.5	0.7	0	1

Q96AG4	Leucine-rich repeat-containing protein 59	LRRC59	35 kDa	1	1	1.1	0.9	1.1	1.2	0.5	0.7	0	1
Q96C86	m7GpppX diphosphatase	DCPS	39 kDa	1	1	0.9	0.8	1.4	1.1	0.5	0.7	0	0
Q9NQX3	Cluster of Gephyrin	GPHN	80 kDa	1	1	1	1.3	1	1	0.5	0.7	0	0
A0MZ66	Cluster of Shootin-1	KIAA1598	72 kDa	1	1	0.9	0.9	1	1	0.5	0.7	0	0
E7ENU4	Double-stranded RNA-specific adenosine deaminase	ADAR	141 kDa	1	1	1.1	1	0.9	1	0.5	0.7	0	0
G8JLD3	Cluster of ELKS/Rab6-interacting/CAST family member 1	ERC1	125 kDa	1	1	0.8	0.9	0.8	0.9	0.5	0.7	0	0
P43487	Cluster of Ran-specific GTPase-activating protein	RANBP1	23 kDa	1	1	0.8	0.8	0.8	0.9	0.5	0.7	0	0
P49903	Selenide, water dikinase 1	SEPHS1	43 kDa	1	1	0.7	1	0.7	0.9	0.5	0.7	0	0
P63279	Cluster of SUMO-conjugating enzyme UBC9	UBE2I	18 kDa	1	1	0.8	0.9	0.7	0.9	0.5	0.7	0	0
Q01085	Cluster of Isoform 2 of Nucleolysin TIAR	TIAL1	43 kDa	1	1	0.7	0.7	0.7	0.9	0.5	0.7	0	0
Q15262	Receptor-type tyrosine-protein phosphatase kappa	PTPRK	162 kDa	1	1	0.8	0.8	0.9	0.8	0.5	0.7	1	1
Q16204	Coiled-coil domain-containing protein 6	CCDC6	53 kDa	1	1	0.7	0.8	0.6	0.8	0.5	0.7	0	0
Q9UH65	Cluster of Switch-associated protein 70	SWAP70	69 kDa	1	1	0.9	0.9	0.9	0.9	0.7	0.7	0	0
B3KSH1	Eukaryotic translation initiation factor 3 subunit F	EIF3F	39 kDa	1	1	1.2	1.1	1.2	1.1	0.8	0.8	1	0
O60832	H/ACA ribonucleoprotein complex subunit 4	DKC1	58 kDa	1	1	0.8	0.9	0.9	0.9	1.3	1.4	0	0
O75821	Eukaryotic translation initiation factor 3 subunit G	EIF3G	36 kDa	1	1	1	1.1	0.9	0.9	0.6	0.6	0	0
P00747	Plasminogen	PLG	91 kDa	1	1	0.7	0.7	0.8	0.7	0.3	0.2	1	0
P29279	Connective tissue growth factor	CTGF	38 kDa	1	1	1.9	1.8	1.6	1.6	0.8	0.9	3	0
P42166	Cluster of Lamina-associated polypeptide 2, isoform alpha	TMPO	75 kDa	1	1	1	0.9	0.9	0.8	1	1.1	0	1
P42785	Cluster of Lysosomal Pro-X carboxypeptidase	PRCP	56 kDa	1	1	0.9	0.9	1.1	1.1	2.1	2	1	0
P61081	NEDD8-conjugating enzyme Ubc12	UBE2M	21 kDa	1	1	1.4	1.4	1.2	1.2	1.6	1.5	0	0
Q01469	Cluster of Fatty acid-binding protein, epidermal	FABP5	15 kDa	1	1	0.5	0.6	0.7	0.8	1	1.1	0	0
Q9NXH9	tRNA (guanine(26)-N(2))-dimethyltransferase	TRMT1	72 kDa	1	1	1.3	1.5	1.3	1	1.3	0.8	0	0
E7EQB2	Lactoferroxin-B (Fragment)	LTF	77 kDa	1	1	0.9	0.7	0.8	1	1.3	0.8	1	0
B4DR80	Cluster of Serine/threonine-protein kinase 24	STK24	46 kDa	1	1	1.2	0.9	1.4	1.4	1.2	0.8	0	0
E7ERF4	Adenylosuccinate lyase	ADSL	56 kDa	1	1	1.2	0.8	1.5	1	1.2	0.8	0	0
E7ERL6	Cluster of Microtubule-associated serine/threonine-protein l	MAST2	176 kDa	1	1	1	0.9	0.9	0.9	0.4	0.6	0	1
E9PGT6	COP9 signalosome complex subunit 8	COPS8	19 kDa	1	1	0.9	0.9	0.9	0.9	1.2	0.8	0	0
O43809	Cleavage and polyadenylation specificity factor subunit 5	NUDT21	26 kDa	1	1	1	1.1	1.2	0.8	1.2	0.8	0	0
O94826	Mitochondrial import receptor subunit TOM70	TOMM70A	67 kDa	1	1	1	0.6	0.8	0.8	1.2	0.8	0	1
P30040	Cluster of Endoplasmic reticulum resident protein 29	ERP29	29 kDa	1	1	0.8	0.7	0.7	0.7	1.2	0.8	1	0
P32929	Cystathionine gamma-lyase	CTH	45 kDa	1	1	1.1	1.1	1.2	1.2	1	0.9	0	0
P62993	Cluster of Growth factor receptor-bound protein 2	GRB2	25 kDa	1	1	1.2	1.6	1.2	1.7	1.1	0.8	0	0
Q13283	Ras GTPase-activating protein-binding protein 1	G3BP1	52 kDa	1	1	1	1.3	1.2	1.3	1.1	0.8	0	0
Q15437	Protein transport protein Sec23B	SEC23B	86 kDa	1	1	1	1	0.9	0.9	0.8	0.9	0	0
Q86SR1	Cluster of Isoform 2 of Polypeptide N-acetylgalactosaminyl	GALNT10	62 kDa	1	1	0.8	1.3	1.1	1.1	1.1	0.8	1	0
Q8WVQ1	Soluble calcium-activated nucleotidase 1	CANT1	45 kDa	1	1	1.2	1.2	1.1	1.2	1.5	1.4	0	1

Q92979	Ribosomal RNA small subunit methyltransferase NEP1	EMG1	27 kDa	1	1	0.7	0.9	0.9	1	1.1	0.8	0	0
Q96QD8	Sodium-coupled neutral amino acid transporter 2	SLC38A2	56 kDa	1	1	0.6	0.6	0.9	0.8	1.1	0.8	0	1
Q9NUP9	Cluster of Protein lin-7 homolog C	LIN7C	22 kDa	1	1	1.1	1	0.8	0.7	1.1	0.8	0	0
Q9UQB8	Cluster of Brain-specific angiogenesis inhibitor 1-associate	BAIAP2	61 kDa	1	1	0.8	0.6	0.8	0.6	1.1	0.8	0	0
E9PAV3	Nascent polypeptide-associated complex subunit alpha	NACA	205 kDa	1	1	1	1.1	1	1	1.1	1.2	0	0
E9PDM8	Cluster of Protein transport protein Sec24D	SEC24D	75 kDa	1	1	2	2.9	2.2	1.5	1	0.8	0	0
F5H241	Glucosylceramidase	GBA	48 kDa	1	1	1.3	1.3	1.3	1.2	1	0.8	1	0
O14974	Cluster of Isoform 4 of Protein phosphatase 1 regulatory sul	PPP1R12A	109 kDa	1	1	1.1	1.3	1.2	1.2	1	0.8	0	0
P39023	Cluster of 60S ribosomal protein L3	RPL3	46 kDa	1	1	1.7	1.4	1.2	1.1	1	0.8	1	0
P50895	Basal cell adhesion molecule	BCAM	67 kDa	1	1	1.3	1.2	1.1	1.1	1	0.8	1	1
P53999	Activated RNA polymerase II transcriptional coactivator p1	SUB1	14 kDa	1	1	1.2	1.2	1	1.1	1	0.8	0	0
Q969P0	Immunoglobulin superfamily member 8	IGSF8	65 kDa	1	1	0.7	1.1	0.9	1	1	0.8	1	1
Q96PZ0	Cluster of Pseudouridylate synthase 7 homolog	PUS7	75 kDa	1	1	1	0.8	0.7	1	1	0.8	0	0
E9PGZ0	Cold shock domain-containing protein E1	CSDE1	91 kDa	1	1	1.1	1	1	0.9	1	0.8	0	0
F5GY50	Prostaglandin reductase 1	PTGR1	33 kDa	1	1	0.9	0.9	0.9	0.9	1	0.8	0	0
O43405	Cluster of Cochlin	COCH	59 kDa	1	1	0.8	0.8	0.9	0.9	1	0.8	1	0
P00966	Argininosuccinate synthase	ASS1	47 kDa	1	1	1.1	1.2	0.9	0.9	0.7	0.7	0	0
P04080	Cystatin-B	CSTB	11 kDa	1	1	0.9	0.9	1	0.8	1	0.8	0	0
P09661	U2 small nuclear ribonucleoprotein A'	SNRPA1	28 kDa	1	1	0.8	0.7	0.8	0.8	1	0.8	0	0
P20290	Cluster of Isoform 2 of Transcription factor BTF3	BTF3	18 kDa	1	1	0.8	1	0.6	0.8	1	0.8	0	0
P49419	Cluster of Alpha-aminoadipic semialdehyde dehydrogenase	ALDH7A1	58 kDa	1	1	0.8	0.7	1	0.7	1	0.8	0	0
P56192	Cluster of Methionine--tRNA ligase, cytoplasmic	MARS	101 kDa	1	1	1.1	1.1	1.1	1.2	0.8	0.9	0	1
P60903	Protein S100-A10	S100A10	11 kDa	1	1	2.4	2	1.9	1.9	0.8	0.9	0	1
Q2TAY7	Cluster of WD40 repeat-containing protein SMU1	SMU1	58 kDa	1	1	1.6	1.6	1.6	1.6	0.9	0.8	0	0
Q32MZ4	Cluster of Isoform 3 of Leucine-rich repeat flightless-intera	LRRFIP1	83 kDa	No Va	No Va	No Value	No Value	No Value	No Value	No Val	No Val	0	0
Q5H9R7	Cluster of Isoform 5 of Serine/threonine-protein phosphatas	PPP6R3	98 kDa	1	1	1.5	1.4	1.4	1.4	0.9	0.8	0	0
Q8IYB7	Cluster of DIS3-like exonuclease 2	DIS3L2	99 kDa	1	1	1.4	1.2	1.4	1.3	0.9	0.8	0	0
Q9UN86	Isoform B of Ras GTPase-activating protein-binding protein	G3BP2	51 kDa	1	1	1.2	1.1	1.4	1.3	0.9	0.8	0	0
Q9UPU5	Cluster of Ubiquitin carboxyl-terminal hydrolase 24	USP24	294 kDa	No Va	No Va	No Value	No Value	No Value	No Value	No Val	No Val	0	0
B7ZKL3	Cluster of EPS8L2 protein	EPS8L2	82 kDa	1	1	1.7	1.7	1.1	1.3	0.9	0.8	0	0
O43240	Kallikrein-10	KLK10	30 kDa	1	1	1.5	1.4	1.2	1.2	0.9	0.8	1	0
O43252	Bifunctional 3'-phosphoadenosine 5'-phosphosulfate syntha	PAPSS1	71 kDa	1	1	1.1	1	1.2	1.2	0.9	0.8	0	0
P01137	Transforming growth factor beta-1	TGFB1	44 kDa	1	1	1	1	1.2	1.2	0.9	0.8	1	0
P84090	Enhancer of rudimentary homolog	ERH	12 kDa	1	1	1	1	0.9	1.2	0.9	0.8	0	0
Q09328	Alpha-1,6-mannosylglycoprotein 6-beta-N-acetylglucosami	MGAT5	85 kDa	1	1	1	0.9	0.8	1.2	0.9	0.8	0	1
Q8WXX5	DnaJ homolog subfamily C member 9	DNAJC9	30 kDa	1	1	0.8	0.9	1.4	1.1	0.9	0.8	0	0
Q92922	SWI/SNF complex subunit SMARCC1	SMARCC1	123 kDa	1	1	0.9	1.2	1.3	1.1	0.9	0.8	0	0

Q99584	Protein S100-A13	S100A13	11 kDa	1	1	1.1	1.1	1.2	1.1	0.9	0.8	0	0
A8MX94	Glutathione S-transferase P	GSTP1	19 kDa	1	1	1.1	1.2	1.1	1.1	0.9	0.8	0	0
B4DHT4	Cluster of U4/U6.U5 tri-snRNP-associated protein 2	USP39	54 kDa	1	1	1.4	1.1	1	1.1	0.9	0.8	0	0
E7EUG6	Cluster of General transcription factor IIF subunit 1	GTF2F1	48 kDa	1	1	1	1.1	1	1.1	0.9	0.8	0	0
H0YB16	Cluster of Focal adhesion kinase 1 (Fragment)	PTK2	81 kDa	No Va	No Va	No Value	No Value	No Value	No Value	No Val	No Val	0	0
P04843	Dolichyl-diphosphooligosaccharide--protein glycosyltransfe	RPN1	69 kDa	1	1	0.9	1	1	1.1	0.9	0.8	1	1
P11766	Alcohol dehydrogenase class-3	ADH5	40 kDa	1	1	1.1	0.9	1.4	1	0.9	0.8	0	0
P37108	Cluster of Signal recognition particle 14 kDa protein	SRP14	15 kDa	1	1	1.1	0.8	1.2	1	0.9	0.8	1	0
P55268	Laminin subunit beta-2	LAMB2	196 kDa	1	1	0.9	0.8	1.2	1	0.9	0.8	1	0
Q13045	Cluster of Protein flightless-1 homolog	FLII	145 kDa	1	1	1.1	1.8	1.1	1	0.9	0.8	0	0
Q68CQ4	Digestive organ expansion factor homolog	DIEXF	87 kDa	1	1	1.4	1.4	1.1	1	0.9	0.8	0	0
Q8WUJ3	Cluster of Protein KIAA1199	CEMIP	153 kDa	1	1	1	1.1	1.1	1	0.9	0.8	1	0
Q92747	Actin-related protein 2/3 complex subunit 1A	ARPC1A	42 kDa	1	1	1.1	1	1.1	1	0.9	0.8	0	0
Q9UJJ9	N-acetylglucosamine-1-phosphotransferase subunit gamma	GNPTG	34 kDa	1	1	1	1.2	1	1	0.9	0.8	1	0
Q9ULC4	Cluster of Malignant T-cell-amplified sequence 1	MCTS1	21 kDa	1	1	1.1	1.1	1	1	0.9	0.8	0	0
Q9Y5S2	Cluster of Serine/threonine-protein kinase MRCK beta	CDC42BPB	194 kDa	1	1	1	1.1	1	1	0.9	0.8	0	0
B4DGP8	Cluster of Calnexin	CANX	72 kDa	1	1	1	1	1	1	0.9	0.8	1	1
B4DM74	60S ribosomal protein L18a	RPL18A	18 kDa	1	1	0.9	0.9	1	1	0.9	0.8	0	0
C9JIF9	Acylamino-acid-releasing enzyme	APEH	82 kDa	1	1	0.7	0.8	0.9	1	0.9	0.8	0	0
J3KQA0	Cluster of Synaptotagmin I, isoform CRA_b	SYT1	47 kDa	1	1	1.1	1.3	0.8	1	0.9	0.8	0	1
J3KTE4	Ribosomal protein L19	RPL19	23 kDa	1	1	0.9	0.9	0.8	1	0.9	0.8	0	0
O75340	Cluster of Programmed cell death protein 6	PDCD6	22 kDa	1	1	1.1	0.8	0.7	1	0.9	0.8	0	0
O75487	Glypican-4	GPC4	62 kDa	1	1	1.2	1.3	1.1	0.9	0.9	0.8	0	1
O95831	Apoptosis-inducing factor 1, mitochondrial	AIFM1	67 kDa	1	1	1.4	1.1	1.1	0.9	0.9	0.8	0	1
P04040	Catalase	CAT	60 kDa	1	1	1.1	1.1	1	0.9	0.9	0.8	0	0
P04114	Apolipoprotein B-100	APOB	516 kDa	1	1	1	0.9	1	0.9	0.9	0.8	1	0
P34896	Cluster of Serine hydroxymethyltransferase, cytosolic	SHMT1	53 kDa	1	1	1	0.8	1	0.9	0.9	0.8	0	0
P54819	Cluster of Adenylate kinase 2, mitochondrial	AK2	26 kDa	1	1	1	1.2	0.9	0.9	0.9	0.8	0	0
Q08257	Quinone oxidoreductase	CRYZ	35 kDa	1	1	0.9	0.9	0.7	0.9	0.9	0.8	0	0
Q12904	Aminoacyl tRNA synthase complex-interacting multifuncti	AIMP1	34 kDa	1	1	0.6	0.6	1	0.8	0.9	0.8	0	0
Q96RS6	NudC domain-containing protein 1	NUDCD1	67 kDa	1	1	1.3	1.3	0.9	0.8	0.9	0.8	0	0
Q9H5V8	CUB domain-containing protein 1	CDCP1	93 kDa	1	1	1.2	1.1	0.9	0.8	0.9	0.8	1	1
Q9Y2X7	Cluster of ARF GTPase-activating protein GIT1	GIT1	84 kDa	1	1	0.7	0.6	0.9	0.8	0.9	0.8	0	0
Q9Y5S9	Cluster of RNA-binding protein 8A	RBM8A	20 kDa	1	1	1.1	0.9	0.8	0.8	0.9	0.8	0	0
E9PJF4	Cluster of Methylosome subunit pICln	CLNS1A	20 kDa	1	1	0.9	0.9	0.8	0.8	0.9	0.8	0	0
O95816	BAG family molecular chaperone regulator 2	BAG2	24 kDa	1	1	0.8	0.7	0.8	0.8	0.9	0.8	0	0
P13861	Cluster of cAMP-dependent protein kinase type II-alpha reg	PRKAR2A	46 kDa	1	1	0.7	0.7	0.8	0.8	0.9	0.8	0	0

P21281	Cluster of V-type proton ATPase subunit B, brain isoform	ATP6V1B2	57 kDa	1	1	0.7	0.7	0.8	0.8	0.9	0.8	0	0
P26358	Isoform 2 of DNA (cytosine-5)-methyltransferase 1	DNMT1	185 kDa	1	1	0.9	1	0.7	0.8	0.9	0.8	0	0
P62191	26S protease regulatory subunit 4	PSMC1	49 kDa	1	1	0.7	0.7	0.7	0.8	0.9	0.8	0	0
P62280	40S ribosomal protein S11	RPS11	18 kDa	1	1	0.7	0.7	0.8	0.7	0.9	0.8	0	0
Q07666	Cluster of KH domain-containing, RNA-binding, signal trar	KHDRBS1	48 kDa	1	1	0.7	0.6	0.8	0.7	0.9	0.8	0	0
Q13421	Cluster of Isoform 4 of Mesothelin	MSLN	68 kDa	1	1	0.7	0.9	0.7	0.7	0.9	0.8	1	1
Q14651	Plastin-1	PLS1	70 kDa	1	1	0.7	0.8	0.7	0.7	0.9	0.8	0	0
Q14677	Cluster of Clathrin interactor 1	CLINT1	68 kDa	1	1	0.9	0.8	1	0.6	0.9	0.8	0	0
Q14703	Membrane-bound transcription factor site-1 protease	MBTPS1	118 kDa	1	1	1	1.1	0.9	0.6	0.9	0.8	0	1
Q6IBS0	Cluster of Twinfilin-2	TWF2	40 kDa	1	1	0.7	0.6	0.5	0.4	0.9	0.8	0	0
Q96B26	Cluster of Exosome complex component RRP43	EXOSC8	30 kDa	1	1	0.8	0.9	0.8	0.9	1	1.1	0	0
Q99536	Synaptic vesicle membrane protein VAT-1 homolog	VAT1	42 kDa	1	1	1.2	1.6	1.6	1.8	0.8	0.8	0	0
Q9BQA1	Methylosome protein 50	WDR77	37 kDa	1	1	1.5	1.3	1.5	1.6	0.8	0.8	0	0
Q9H2G2	Cluster of Isoform 2 of STE20-like serine/threonine-protein	SLK	139 kDa	1	1	1.3	1.4	1.4	1.6	0.8	0.8	0	0
Q9UBW8	COP9 signalosome complex subunit 7a	COPS7A	30 kDa	1	1	1.3	1.2	1.5	1.4	0.8	0.8	0	0
Q9UKV8	Protein argonaute-2	AGO2	97 kDa	1	1	0.9	0.9	0.9	0.8	0.9	0.9	0	0
Q9Y2Z0	Suppressor of G2 allele of SKP1 homolog	SUGT1	41 kDa	1	1	1.3	1.3	1.3	1.3	0.8	0.8	0	0
E9PDY5	Cluster of Heparan-sulfate 6-O-sulfotransferase 2	HS6ST2	73 kDa	1	1	1.2	1.3	1.2	1.3	0.8	0.8	1	1
H3BPF6	Cluster of Prefoldin subunit 5 (Fragment)	PFDN5	17 kDa	1	1	1.2	1.2	1.2	1.3	0.8	0.8	0	0
H3BRL3	Ubiquitin domain-containing protein UBFD1	UBFD1	33 kDa	1	1	1.1	1.1	1.2	1.3	0.8	0.8	0	0
J3KNX3	Cluster of 6-phosphofructokinase	PFKM	93 kDa	1	1	1.1	1.1	1.1	1.3	0.8	0.8	0	0
O43765	Small glutamine-rich tetratricopeptide repeat-containing prc	SGTA	34 kDa	1	1	1.1	1.1	1.2	1.2	0.8	0.8	0	0
O75153	Cluster of Clustered mitochondria protein homolog	CLUH	147 kDa	1	1	1.2	1.2	1.1	1.2	0.8	0.8	0	0
O94776	Metastasis-associated protein MTA2	MTA2	75 kDa	1	1	1.1	1.2	1.1	1.2	0.8	0.8	0	0
P00441	Cluster of Superoxide dismutase [Cu-Zn]	SOD1	16 kDa	1	1	1.1	1	1.1	1.2	0.8	0.8	0	0
P09497	Isoform Non-brain of Clathrin light chain B	CLTB	23 kDa	1	1	1	1.1	1	1.2	0.8	0.8	0	0
P16278	Cluster of Beta-galactosidase	GLB1	76 kDa	1	1	0.9	1.1	1	1.2	0.8	0.8	1	0
P17900	Ganglioside GM2 activator	GM2A	21 kDa	1	1	1	1	1.5	1.1	0.8	0.8	1	0
P48047	Cluster of ATP synthase subunit O, mitochondrial	ATP5O	23 kDa	1	1	1.2	1.1	1.3	1.1	0.8	0.8	0	0
P61163	Cluster of Alpha-centractin	ACTR1A	43 kDa	No Va	1	No Value	1.1	No Value	1	No Val	0.9	0	0
P62491	Cluster of Ras-related protein Rab-11A	RAB11A	24 kDa	1	1	1.2	1.1	1.3	1.1	0.8	0.8	0	0
Q06265	Cluster of Exosome complex component RRP45	EXOSC9	49 kDa	1	1	1.1	1.2	1.2	1.1	0.8	0.8	0	0
Q08209	Cluster of Isoform 2 of Serine/threonine-protein phosphatas	PPP3CA	58 kDa	1	1	1.2	1.1	1.2	1.1	0.8	0.8	0	0
Q15233	Non-POU domain-containing octamer-binding protein	NONO	54 kDa	1	1	1.3	1.2	1.1	1.1	0.8	0.8	0	0
Q8WVM8	Cluster of Sec1 family domain-containing protein 1	SCFD1	72 kDa	1	1	1.2	1.2	1.1	1.1	0.8	0.8	1	1
Q9GZL7	Ribosome biogenesis protein WDR12	WDR12	48 kDa	1	1	1.1	1	1.1	1.1	0.8	0.8	0	0
Q9UHV9	Prefoldin subunit 2	PFDN2	17 kDa	1	1	1	1	1.1	1.1	0.8	0.8	0	0

Q9Y333	U6 snRNA-associated Sm-like protein LSm2	LSM2	11 kDa	1	1	1	1	1.1	1.1	0.8	0.8	0	0
Q9Y6G9	Cytoplasmic dynein 1 light intermediate chain 1	DYNC1LI1	57 kDa	1	1	0.8	1	1.1	1.1	0.8	0.8	0	0
B7ZKJ8	Cluster of ITIH4 protein	ITIH4	104 kDa	1	1	1	1.2	1	1.1	0.8	0.8	1	0
A8MV58	Cluster of Drebrin	DBN1	76 kDa	1	1	1	1.2	1	1.1	0.8	0.8	0	0
B4E1Z4	Cluster of Complement factor B Ba fragment	CFB	141 kDa	1	1	1.1	1.1	1	1.1	0.8	0.8	1	0
H0YJ34	Fermitin family homolog 2 (Fragment)	FERMT2	73 kDa	1	1	1	1.1	1	1.1	0.8	0.8	0	0
J3KNL3	Cluster of Chitinase domain-containing protein 1	CHID1	48 kDa	1	1	1.1	1	1	1.1	0.8	0.8	1	0
O00499	Cluster of Isoform IIB of Myc box-dependent-interacting pr	BIN1	56 kDa	1	1	1	1	1	1.1	0.8	0.8	0	0
O75937	DnaJ homolog subfamily C member 8	DNAJC8	30 kDa	1	1	1	1	1	1.1	0.8	0.8	0	0
O96019	Cluster of Actin-like protein 6A	ACTL6A	47 kDa	1	1	1	0.9	1	1.1	0.8	0.8	0	0
P24752	Cluster of Acetyl-CoA acetyltransferase, mitochondrial	ACAT1	45 kDa	1	1	0.9	0.9	1	1.1	0.8	0.8	0	0
Q07065	Cytoskeleton-associated protein 4	CKAP4	66 kDa	1	1	1	1.1	0.9	1.1	0.8	0.8	0	1
Q13557	Cluster of Isoform Delta 12 of Calcium/calmodulin-depend	CAMK2D	54 kDa	1	1	0.9	1.1	1.2	1	0.8	0.8		
Q9H9G7	Cluster of Protein argonaute-3	AGO3	97 kDa	1	1	1.2	1.2	1.1	1	0.8	0.8	0	0
Q9NX58	Cell growth-regulating nucleolar protein	LYAR	44 kDa	1	1	1	1	1.1	1	0.8	0.8	0	0
Q9NYL9	Cluster of Tropomodulin-3	TMOD3	40 kDa	1	1	0.9	0.9	1.1	1	0.8	0.8	0	0
Q9UKK9	Cluster of ADP-sugar pyrophosphatase	NUDT5	24 kDa	1	1	0.9	0.9	1.1	1	0.8	0.8	0	0
Q9Y2X3	Nucleolar protein 58	NOP58	60 kDa	1	1	1.3	1.4	1	1	0.8	0.8	0	0
P02749	Beta-2-glycoprotein 1	APOH	38 kDa	1	1	1.4	1.3	1	1	0.8	0.8	1	0
H0YLU8	Cluster of Low-density lipoprotein receptor	LDLR	95 kDa	1	1	1.3	1.2	1	1	0.8	0.8	1	1
I3NI00	Cluster of Basigin	BSG	46 kDa	1	1	1.2	1.2	1	1	0.8	0.8	1	1
O00571	Cluster of ATP-dependent RNA helicase DDX3X	DDX3X	73 kDa	1	1	1	1.1	1	1	0.8	0.8	0	0
O60925	Prefoldin subunit 1	PFDN1	14 kDa	1	1	1.1	1	1	1	0.8	0.8	0	1
O75146	Huntingtin-interacting protein 1-related protein	HIP1R	119 kDa	1	1	0.9	1	1	1	0.8	0.8	0	0
O95456	Cluster of Proteasome assembly chaperone 1	PSMG1	33 kDa	1	1	0.9	1	1	1	0.8	0.8	0	0
P09496	Cluster of Clathrin light chain A	CLTA	27 kDa	1	1	1	0.9	1	1	0.8	0.8	0	0
P10155	Cluster of 60 kDa SS-A/Ro ribonucleoprotein	TROVE2	61 kDa	1	1	1	0.9	1	1	0.8	0.8	0	0
P21283	Cluster of V-type proton ATPase subunit C 1	ATP6V1C1	44 kDa	1	1	0.9	0.9	1	1	0.8	0.8	0	0
P33993	DNA replication licensing factor MCM7	MCM7	81 kDa	1	1	0.9	0.8	1	1	0.8	0.8	0	0
P35237	Cluster of Serpin B6	SERPINB6	43 kDa	1	1	0.7	0.8	1	1	0.8	0.8	0	0
P78347	Cluster of General transcription factor II-I	GTF2I	112 kDa	1	1	0.8	0.7	1	1	0.8	0.8	0	0
Q15637	Cluster of Isoform 3 of Splicing factor 1	SF1	67 kDa	1	1	1.1	1.1	0.9	1	0.8	0.8	0	0
Q96DI7	U5 small nuclear ribonucleoprotein 40 kDa protein	SNRNP40	39 kDa	1	1	1	1	0.9	1	0.8	0.8	0	1
Q9NQC3	Cluster of Reticulon-4	RTN4	130 kDa	No Va	No Va	No Value	No Value	No Value	No Value	No Val	No Val	0	1
Q9NRW7	Cluster of Vacuolar protein sorting-associated protein 45	VPS45	65 kDa	1	1	1.1	0.9	0.9	1	0.8	0.8	0	0
A6NM98	Cluster of ARD1 homolog A, N-acetyltransferase (S. cerevi	NAA10	25 kDa	1	1	0.9	0.9	0.9	1	0.8	0.8	0	0
E5RJR5	S-phase kinase-associated protein 1	SKP1	19 kDa	1	1	0.8	0.8	0.9	1	0.8	0.8	0	0

H0YCP8	Cluster of Poly(U)-binding-splicing factor PUF60 (Fragmer	PUF60	28 kDa	No Va	No Va	No Value	No Value	No Valu	No Value	No Val	No Val	0	0
H7BY58	Cluster of Protein-L-isoaspartate O-methyltransferase	PCMT1	30 kDa	1	1	0.8	0.8	0.9	1	0.8	0.8	0	1
O00487	26S proteasome non-ATPase regulatory subunit 14	PSMD14	35 kDa	1	1	1	1	0.8	1	0.8	0.8	0	0
P22223	Cluster of Isoform 2 of Cadherin-3	CDH3	87 kDa	1	1	0.9	1.2	0.7	1	0.8	0.8	1	1
P46782	40S ribosomal protein S5	RPS5	23 kDa	1	1	0.8	0.7	0.6	1	0.8	0.8	0	0
P49756	Cluster of RNA-binding protein 25	RBM25	100 kDa	1	1	1	0.9	1	0.9	0.8	0.8	0	0
P61964	WD repeat-containing protein 5	WDR5	37 kDa	1	1	0.9	0.9	1	0.9	0.8	0.8	0	0
P67775	Cluster of Serine/threonine-protein phosphatase 2A catalyti	PPP2CA	36 kDa	1	1	0.9	0.9	1	0.9	0.8	0.8	0	0
Q13547	Cluster of Histone deacetylase 1	HDAC1	55 kDa	1	1	0.9	0.8	1	0.9	0.8	0.8	0	0
Q13596	Sorting nexin-1	SNX1	59 kDa	1	1	0.7	0.7	1	0.9	0.8	0.8	0	0
Q15061	WD repeat-containing protein 43	WDR43	75 kDa	1	1	1	1.2	0.9	0.9	0.8	0.8	0	0
Q8NBF2	NHL repeat-containing protein 2	NHLRC2	79 kDa	1	1	1	1.1	0.9	0.9	0.8	0.8	0	0
Q8NG11	Tetraspanin-14	TSPAN14	31 kDa	1	1	1.1	1	0.9	0.9	0.8	0.8	0	1
Q8WXF1	Paraspeckle component 1	PSPC1	59 kDa	1	1	1	1	0.9	0.9	0.8	0.8	0	0
Q9GZT8	NIF3-like protein 1	NIF3L1	42 kDa	1	1	1	1	0.9	0.9	0.8	0.8	0	1
P62318	Cluster of Small nuclear ribonucleoprotein Sm D3	SNRPD3	14 kDa	1	1	1	1	0.9	0.9	0.8	0.8	0	0
B7Z6D9	Cluster of Rho guanine nucleotide exchange factor 7	ARHGEF7	20 kDa	1	1	1	1	0.9	0.9	0.8	0.8	0	0
C9J502	Cluster of Prostate leucine zipper variant 2	TPD52	26 kDa	No Va	No Va	No Value	No Value	No Valu	No Value	No Val	No Val	1	0
P19823	Inter-alpha-trypsin inhibitor heavy chain H2	ITIH2	106 kDa	1	1	1.1	0.9	0.9	0.9	0.8	0.8	1	0
P21796	Voltage-dependent anion-selective channel protein 1	VDAC1	31 kDa	1	1	1.1	0.9	0.9	0.9	0.8	0.8	0	0
P25205	Cluster of DNA replication licensing factor MCM3	MCM3	91 kDa	1	1	1	0.9	0.9	0.9	0.8	0.8	0	0
P36915	Guanine nucleotide-binding protein-like 1	GNL1	69 kDa	1	1	0.9	0.9	0.9	0.9	0.8	0.8	0	0
P48556	26S proteasome non-ATPase regulatory subunit 8	PSMD8	40 kDa	1	1	0.8	0.9	0.9	0.9	0.8	0.8	1	1
P49411	Cluster of Elongation factor Tu, mitochondrial	TUFM	50 kDa	1	1	0.7	0.9	0.9	0.9	0.8	0.8	0	0
P61326	Cluster of Protein mago nashi homolog	MAGOH	17 kDa	1	1	0.8	0.8	0.9	0.9	0.8	0.8	0	0
Q02241	Cluster of Kinesin-like protein KIF23	KIF23	110 kDa	1	1	0.8	0.8	0.9	0.9	0.8	0.8	0	0
Q12792	Isoform 4 of Twinfilin-1	TWF1	29 kDa	1	1	0.9	1	0.8	0.9	0.8	0.8	0	0
Q13085	Cluster of Acetyl-CoA carboxylase 1	ACACA	266 kDa	1	1	0.9	0.9	0.8	0.9	0.8	0.8	0	0
Q15007	Pre-mRNA-splicing regulator WTAP	WTAP	44 kDa	1	1	0.8	0.9	0.8	0.9	0.8	0.8	0	0
Q16610	Cluster of Extracellular matrix protein 1	ECM1	61 kDa	1	1	0.7	0.7	0.8	0.9	0.8	0.8	1	0
Q6FI81	Anamorsin	CIAPIN1	34 kDa	1	1	0.7	0.7	0.7	0.9	0.8	0.8	0	0
Q92925	Cluster of Isoform 3 of SWI/SNF-related matrix-associated	SMARCD2	55 kDa	1	1	0.7	0.8	0.6	0.9	0.8	0.8	0	0
Q96HC4	Cluster of PDZ and LIM domain protein 5	PDLIM5	64 kDa	1	1	0.9	1	1.2	0.8	0.8	0.8	0	1
Q9NR45	Cluster of Sialic acid synthase	NANS	40 kDa	1	1	1.1	1.1	1	0.8	0.8	0.8	0	0
Q9UBR2	Cathepsin Z	CTSZ	34 kDa	1	1	1.4	1.2	0.9	0.8	0.8	0.8	3	0
Q9Y285	Phenylalanine--tRNA ligase alpha subunit	FARSA	58 kDa	1	1	1.2	1	0.9	0.8	0.8	0.8	0	0
B0QZ18	Cluster of Copine I	CPNE1	60 kDa	1	1	0.9	1	0.9	0.8	0.8	0.8	0	0

J3KPT2	Cluster of Exostoses (Multiple) 2, isoform CRA_d	EXT2	86 kDa	No Va	No Va	No Value	No Value	No Valu	No Value	No Val	No Val	0	1
P35659	Cluster of Protein DEK	DEK	43 kDa	1	1	0.8	1	0.9	0.8	0.8	0.8	0	0
P39019	40S ribosomal protein S19	RPS19	16 kDa	1	1	1	0.9	0.9	0.8	0.8	0.8	0	0
P47813	Cluster of Eukaryotic translation initiation factor 1A, X-chr	EIF1AX	16 kDa	1	1	0.8	0.9	0.9	0.8	0.8	0.8	0	0
P49902	Cluster of Cytosolic purine 5'-nucleotidase	NT5C2	65 kDa	1	1	0.7	0.6	0.9	0.8	0.8	0.8	0	0
P55263	Isoform 3 of Adenosine kinase	ADK	34 kDa	1	1	0.9	1	0.8	0.8	0.8	0.8	0	0
Q13464	Rho-associated protein kinase 1	ROCK1	158 kDa	1	1	0.9	1	0.8	0.8	0.8	0.8	0	0
Q15291	Retinoblastoma-binding protein 5	RBBP5	59 kDa	1	1	1	0.9	0.8	0.8	0.8	0.8	0	0
Q6IN85	Cluster of Serine/threonine-protein phosphatase 4 regulator	SMEK1	95 kDa	1	1	0.9	0.9	0.8	0.8	0.8	0.8	0	0
Q6PD62	RNA polymerase-associated protein CTR9 homolog	CTR9	134 kDa	0.9	1	0.9	0.9	0.8	0.8	0.8	0.8	0	0
Q92466	DNA damage-binding protein 2	DDB2	48 kDa	1	1	0.7	0.9	0.8	0.8	0.8	0.8	0	0
Q92888	Cluster of Rho guanine nucleotide exchange factor 1	ARHGEF1	102 kDa	1	1	0.7	0.7	0.8	0.8	0.8	0.8	0	0
Q9HB40	Retinoid-inducible serine carboxypeptidase	SCPEP1	51 kDa	1	1	0.6	0.7	0.8	0.8	0.8	0.8	1	0
Q9UI26	Importin-11	IPO11	113 kDa	1	1	0.7	0.6	0.8	0.8	0.8	0.8	1	0
Q9UK41	Vacuolar protein sorting-associated protein 28 homolog	VPS28	25 kDa	1	1	1	1	0.7	0.8	0.8	0.8	0	0
Q9ULT8	E3 ubiquitin-protein ligase HECTD1	HECTD1	289 kDa	1	1	0.9	0.9	0.7	0.8	0.8	0.8	0	0
Q9Y5Y6	Suppressor of tumorigenicity 14 protein	ST14	95 kDa	1	1	0.7	0.8	0.7	0.8	0.8	0.8	0	1
B4DP17	Cluster of Cellular nucleic acid-binding protein	CNBP	18 kDa	1	1	0.7	0.8	0.7	0.8	0.8	0.8	0	0
F8W1H5	Ras-related protein Rab-5C	RAB5C	27 kDa	1	1	0.8	0.7	0.7	0.8	0.8	0.8	0	0
O43172	Cluster of Isoform 2 of U4/U6 small nuclear ribonucleoprot	PRPF4	58 kDa	No Va	No Va	No Value	No Value	No Valu	No Value	No Val	No Val	0	0
O94874	E3 UFM1-protein ligase 1	UFL1	90 kDa	1	1	0.7	0.7	0.7	0.8	0.8	0.8	0	0
O95793	Cluster of Double-stranded RNA-binding protein Staufen h	STAU1	63 kDa	1	1	0.7	0.7	0.6	0.8	0.8	0.8	0	0
P07225	Cluster of Vitamin K-dependent protein S	PROS1	75 kDa	1	1	0.7	0.7	0.8	0.7	0.8	0.8	1	1
P23368	Cluster of NAD-dependent malic enzyme, mitochondrial	ME2	65 kDa	1	1	1	0.9	0.7	0.7	0.8	0.8	0	0
P31689	DnaJ homolog subfamily A member 1	DNAJA1	45 kDa	1	1	0.7	0.7	0.7	0.7	0.8	0.8	0	0
P36952	Cluster of Serpin B5	SERPINB5	42 kDa	1	1	0.8	0.7	0.7	0.6	0.8	0.8	0	0
P45973	Chromobox protein homolog 5	CBX5	22 kDa	1	1	0.5	0.6	0.7	0.6	0.8	0.8	0	0
P49366	Deoxyhypusine synthase	DHPS	41 kDa	1	1	0.4	0.5	0.5	0.6	0.8	0.8	0	0
P62917	60S ribosomal protein L8	RPL8	28 kDa	1	1	1.3	1.3	1.2	1.3	1.3	1.3	0	0
Q69YN2	CWF19-like protein 1	CWF19L1	61 kDa	1	1	0.5	0.5	0.4	0.4	0.8	0.8	0	0
Q8N7H5	RNA polymerase II-associated factor 1 homolog	PAF1	60 kDa	1	1	1.5	1.6	1.3	1.6	0.7	0.8	0	0
Q8TD19	Serine/threonine-protein kinase Nek9	NEK9	107 kDa	1	1	1.7	1.2	1.3	1.6	0.7	0.8	0	0
Q92905	COP9 signalosome complex subunit 5	COPS5	38 kDa	1	1	1.2	1.2	1.5	1.4	0.7	0.8	0	0
Q9NY97	UDP-GlcNAc:betaGal beta-1,3-N-acetylglucosaminyltransf	B3GNT2	46 kDa	1	1	1.4	1.3	1.3	1.4	0.7	0.8	0	1
Q9P0L0	Vesicle-associated membrane protein-associated protein A	VAPA	28 kDa	1	1	1.2	1.2	1.2	1.4	0.7	0.8	0	1
P30419	Cluster of Glycylpeptide N-tetradecanoyltransferase 1	NMT1	57 kDa	1	1	1.1	1	1.1	1.3	0.7	0.8	0	0
B5MC59	Cluster of Replication protein A 14 kDa subunit	RPA3	9 kDa	1	1	1.1	1	1	1.3	0.7	0.8	0	0

C9JPM4	ADP-ribosylation factor 4 (Fragment)	ARF4	15 kDa	1	1	1.3	1.2	1.3	1.2	0.7	0.8	0	1
B7Z9I3	Cluster of Renin receptor	ATP6AP2	36 kDa	1	1	1.1	1.2	1.1	1.2	0.7	0.8	1	1
E7EQ69	Cluster of N-alpha-acetyltransferase 50	NAA50	19 kDa	1	1	1	1.2	1.1	1.2	0.7	0.8	0	0
H3BRM0	Cluster of Phosphomannomutase 2	PMM2	18 kDa	1	1	1	1.1	1.1	1.2	0.7	0.8	0	0
J3KNW4	Four and a half LIM domains protein 2	FHL2	44 kDa	1	1	1.1	1.1	1.1	1.1	0.7	0.8	0	0
J3KPV4	Cluster of DNA replication licensing factor MCM4	MCM4	95 kDa	1	1	1	1	1.1	1.1	0.7	0.8	0	0
O75462	Cytokine receptor-like factor 1	CRLF1	46 kDa	1	1	1.1	1.2	1	1.1	0.7	0.8	1	0
P05362	Intercellular adhesion molecule 1	ICAM1	58 kDa	1	1	0.9	1	1	1.1	0.7	0.8	1	1
P31949	Protein S100-A11	S100A11	12 kDa	1	1	1	0.9	1	1.1	0.7	0.8	0	0
P62753	Cluster of 40S ribosomal protein S6	RPS6	29 kDa	1	1	1.1	1.2	0.9	1.1	0.7	0.8	0	0
Q08J23	tRNA (cytosine(34)-C(5))-methyltransferase	NSUN2	86 kDa	1	1	1	1	0.9	1.1	0.7	0.8	0	0
Q10471	Cluster of Polypeptide N-acetylgalactosaminyltransferase 2	GALNT2	65 kDa	1	1	0.9	1	0.9	1.1	0.7	0.8	1	0
Q14558	Cluster of Phosphoribosyl pyrophosphate synthase-associat	PRPSAP1	39 kDa	1	1	1.6	1.2	1.1	1	0.7	0.8	0	0
Q15424	Cluster of Scaffold attachment factor B1	SAFB	103 kDa	1	1	0.9	1.1	1.1	1	0.7	0.8	0	0
Q15428	Splicing factor 3A subunit 2	SF3A2	49 kDa	1	1	1	0.9	1.1	1	0.7	0.8	0	0
Q15942	Cluster of Zyxin	ZYX	61 kDa	1	1	0.9	0.9	1.1	1	0.7	0.8	0	0
Q9UBC2	Isoform 2 of Epidermal growth factor receptor substrate 15-	EPS15L1	100 kDa	1	1	1.2	1.3	1	1	0.7	0.8	0	0
Q9UHL4	Dipeptidyl peptidase 2	DPP7	54 kDa	1	1	0.9	1.3	1	1	0.7	0.8	1	0
P69905	Hemoglobin subunit alpha	HBA1	15 kDa	1	1	0.9	1	1	1	0.7	0.8	0	0
A8MTH6	Sorcin	SRI	20 kDa	1	1	0.9	0.9	1	1	0.7	0.8	0	0
O15551	Cluster of Claudin-3	CLDN3	23 kDa	1	1	0.9	0.9	1	1	0.7	0.8	0	1
O43399	Cluster of Tumor protein D54	TPD52L2	22 kDa	1	1	1.1	1.2	0.9	1	0.7	0.8	0	0
P00568	Cluster of Adenylate kinase isoenzyme 1	AK1	22 kDa	1	1	0.9	0.9	0.9	1	0.7	0.8	0	0
P00918	Cluster of Carbonic anhydrase 2	CA2	29 kDa	1	1	0.9	0.9	0.9	1	0.7	0.8	0	0
P09341	Growth-regulated alpha protein	CXCL1	11 kDa	1	1	1.1	1.3	0.8	1	0.7	0.8	3	0
P16615	Cluster of Sarcoplasmic/endoplasmic reticulum calcium AT	ATP2A2	115 kDa	1	1	1	0.9	0.8	1	0.7	0.8	0	1
P16885	1-phosphatidylinositol 4,5-bisphosphate phosphodiesterase	PLCG2	148 kDa	1	1	1.8	1.4	1.5	0.9	0.7	0.8	0	0
P37802	Transgelin-2	TAGLN2	22 kDa	1	1	0.9	1	1.2	0.9	0.7	0.8	1	0
P50897	Palmitoyl-protein thioesterase 1	PPT1	34 kDa	1	1	1.1	1.4	1	0.9	0.7	0.8	1	0
P61088	Cluster of Ubiquitin-conjugating enzyme E2 N	UBE2N	17 kDa	1	1	1	0.9	1	0.9	0.7	0.8	0	0
Q13619	Cullin-4A	CUL4A	88 kDa	1	1	0.9	0.7	1	0.9	0.7	0.8	0	0
Q8IW45	Cluster of Isoform 2 of ATP-dependent (S)-NAD(P)H-hydr	CARKD	41 kDa	1	1	1.2	1.2	0.9	0.9	0.7	0.8	1	0
Q92797	Symplekin	SYMPK	141 kDa	1	1	0.9	0.9	0.9	0.9	0.7	0.8	0	0
Q9UNN5	Cluster of FAS-associated factor 1	FAF1	74 kDa	1	1	0.8	0.9	0.9	0.9	0.7	0.8	0	1
Q13510	Isoform 2 of Acid ceramidase	ASAH1	47 kDa	1	1	0.8	0.8	0.9	0.9	0.7	0.8	1	0
O75531	Barrier-to-autointegration factor	BANF1	10 kDa	1	1	0.8	0.9	0.8	0.9	0.7	0.8	0	0
A6NKB8	Cluster of Aminopeptidase B	RNPEP	68 kDa	1	1	0.9	1	0.7	0.9	0.7	0.8	1	0

B3KSI9	Cluster of Leucine zipper transcription factor-like 1, isoforr	LZTFL1	33 kDa	1	1	1	1	0.6	0.9	0.7	0.8	0	0
B4DLN1	Cluster of Mitochondrial dicarboxylate carrier	SLC25A10	48 kDa	1	1	0.8	0.7	0.9	0.8	0.7	0.8	0	1
B4DMU0	Cluster of Pyrroline-5-carboxylate reductase	PYCR1	36 kDa	1	1	1	1.1	0.8	0.8	0.7	0.8	0	0
B7Z6Z4	Cluster of Myosin light polypeptide 6	MYL6	27 kDa	No Va	No Va	No Value	No Value	No Value	No Value	No Val	No Val	0	0
E9PCI9	Farnesyl pyrophosphate synthase	FDPS	41 kDa	1	1	0.9	0.9	0.8	0.8	0.7	0.8	0	0
E9PFR3	Cluster of Serine/threonine-protein phosphatase 2A 56 kDa	PPP2R5D	69 kDa	1	1	0.8	0.9	0.8	0.8	0.7	0.8	0	0
O43324	Cluster of Eukaryotic translation elongation factor 1 epsilor	EEF1E1	20 kDa	1	1	0.9	0.8	0.8	0.8	0.7	0.8	0	1
O60264	SWI/SNF-related matrix-associated actin-dependent regulat	SMARCA5	122 kDa	1	1	0.7	0.7	0.8	0.8	0.7	0.8	0	0
P13693	Cluster of Translationally-controlled tumor protein	TPT1	20 kDa	1	1	0.9	0.9	0.7	0.8	0.7	0.8	0	0
P15104	Glutamine synthetase	GLUL	42 kDa	1	1	0.8	0.8	0.7	0.8	0.7	0.8	0	0
P21291	Cluster of Cysteine and glycine-rich protein 1	CSRP1	21 kDa	1	1	0.7	0.7	0.7	0.8	0.7	0.8	0	0
P45880	Voltage-dependent anion-selective channel protein 2	VDAC2	32 kDa	1	1	1	0.8	0.9	0.7	0.7	0.8	0	0
P46013	Antigen KI-67	MKI67	359 kDa	1	1	0.7	0.7	0.9	0.7	0.7	0.8	0	0
P63220	40S ribosomal protein S21	RPS21	9 kDa	1	1	0.9	1	0.7	0.7	0.7	0.8	0	0
Q05086	Ubiquitin-protein ligase E3A	UBE3A	101 kDa	1	1	0.8	0.7	0.7	0.7	0.7	0.8	0	1
Q12907	Cluster of Vesicular integral-membrane protein VIP36	LMAN2	40 kDa	1	1	0.8	0.5	0.7	0.7	0.7	0.8	1	1
Q13011	Delta(3,5)-Delta(2,4)-dienoyl-CoA isomerase, mitochondria	ECH1	36 kDa	1	1	0.8	0.8	0.9	0.9	0.8	0.8	0	0
Q14847	LIM and SH3 domain protein 1	LASP1	30 kDa	1	1	1	1	1.1	1.3	0.6	0.8	0	0
Q15057	Cluster of Arf-GAP with coiled-coil, ANK repeat and PH d	ACAP2	88 kDa	1	1	1.3	1.3	1.2	1.1	0.6	0.8	0	0
Q15758	Neutral amino acid transporter B(0)	SLC1A5	57 kDa	1	1	1.4	1	1.2	1.1	0.6	0.8	0	1
Q9H074	Polyadenylate-binding protein-interacting protein 1	PAIP1	54 kDa	1	1	0.9	1	1	1.1	0.6	0.8	0	0
Q9H3P7	Golgi resident protein GCP60	ACBD3	61 kDa	1	1	0.7	0.9	1	1.1	0.6	0.8	0	0
Q9Y281	Cofilin-2	CFL2	19 kDa	1	1	0.9	1.1	0.8	1.1	0.6	0.8	0	0
B4DYH8	Cluster of N-acetylglucosamine-6-sulfatase	GNS	60 kDa	1	1	1.4	1.3	1.2	1	0.6	0.8	1	0
B4DZM8	26S proteasome non-ATPase regulatory subunit 5	PSMD5	51 kDa	1	1	0.9	1.3	1.1	1	0.6	0.8	0	0
F5H4C2	Ran-binding protein 3	RANBP3	47 kDa	1	1	1	1	1	1	0.6	0.8	0	0
H3BLV8	Vacuolar protein sorting-associated protein 29 (Fragment)	VPS29	22 kDa	1	1	1	0.9	0.9	1	0.6	0.8	0	0
J3KQT7	Cluster of Inositol monophosphatase 1	IMPA1	37 kDa	1	1	0.8	1	0.8	1	0.6	0.8	0	0
O14617	AP-3 complex subunit delta-1	AP3D1	130 kDa	1	1	0.8	0.8	0.8	0.9	0.6	0.8	0	0
O43148	mRNA cap guanine-N7 methyltransferase	RNMT	55 kDa	1	1	0.6	0.6	0.8	0.9	0.6	0.8	0	0
O60884	DnaJ homolog subfamily A member 2	DNAJA2	46 kDa	1	1	0.9	0.8	0.7	0.9	0.6	0.8	0	0
P10606	Cytochrome c oxidase subunit 5B, mitochondrial	COX5B	14 kDa	1	1	0.6	0.7	0.8	0.8	0.5	0.5	0	0
P33240	Cluster of Cleavage stimulation factor subunit 2	CSTF2	61 kDa	1	1	0.7	1	0.8	0.7	0.6	0.8	0	0
P46087	Putative ribosomal RNA methyltransferase NOP2	NOP2	89 kDa	1	1	0.7	0.8	0.8	0.7	0.6	0.8	0	0
P46108	Cluster of Adapter molecule crk	CRK	34 kDa	1	1	0.7	0.9	0.6	0.7	0.6	0.8	0	0
P62314	Small nuclear ribonucleoprotein Sm D1	SNRPD1	13 kDa	1	1	0.4	0.5	0.4	0.5	0.6	0.8	0	0
Q08AM6	Cluster of Protein VAC14 homolog	VAC14	88 kDa	1	1	1.1	1.4	1.1	1.5	0.5	0.8	0	0

Q14019	Coactosin-like protein	COTL1	16 kDa	1	1	1	1.5	1.7	1.3	0.5	0.8	0	0
Q14139	Ubiquitin conjugation factor E4 A	UBE4A	123 kDa	1	1	1.1	1.3	1.1	1.2	0.5	0.8	0	0
Q15293	Cluster of Reticulocalbin-1	RCN1	39 kDa	1	1	0.9	1.4	0.8	1.1	0.5	0.8	1	0
Q5LJB0	Cluster of Ubiquitin carboxyl-terminal hydrolase L5 (Fragm	UCHL5	29 kDa	1	1	1	1	1.1	1	0.5	0.8	0	0
Q8NBJ5	Procollagen galactosyltransferase 1	COLGALT1	72 kDa	1	1	0.7	0.9	0.9	0.9	0.5	0.8	1	0
Q9BQG0	Cluster of Myb-binding protein 1A	MYBBP1A	149 kDa	1	1	1.2	1.4	1	0.8	0.5	0.8	0	0
Q9Y6Q5	AP-1 complex subunit mu-2	AP1M2	48 kDa	1	1	0.8	1.2	0.6	0.8	0.5	0.8	0	0
C9J4T6	IL-8(8-77)	IL8	11 kDa	Value	1	100	18.6	100	28.9	100	9.1	1	0
E7EQV9	Ribosomal protein L15 (Fragment)	RPL15	21 kDa	1	1	1	1.1	0.9	1	1	1.1	0	0
F2Z2X4	Exportin-4	XPO4	130 kDa	1	1	0.9	1	0.8	0.8	0.7	0.7	0	0
F8VVL1	Density-regulated protein	DENR	18 kDa	1	1	0.9	0.8	0.9	0.8	1.4	1.3	0	0
G3V3D1	Cluster of Epididymal secretory protein E1 (Fragment)	NPC2	24 kDa	1	1	1	1.1	1	1.1	1.4	1.6	1	0
H0YN88	Cluster of 40S ribosomal protein S17	RPS17L	16 kDa	1	1	1.3	1.2	1	0.9	1.5	1.4	0	0
O14497	Cluster of AT-rich interactive domain-containing protein 1A	ARID1A	242 kDa	1	1	0.6	0.8	0.7	0.7	1.2	1.1	0	0
O15212	Prefoldin subunit 6	PFDN6	15 kDa	1	1	0.8	0.8	0.8	0.9	1.5	0.9	0	0
O60911	Cathepsin L2	CTSV	37 kDa	1	1	2.3	1.9	2.2	2.3	1.4	0.9	1	0
P06730	Cluster of Isoform 2 of Eukaryotic translation initiation fact	EIF4E	29 kDa	No Va	No Va	No Value	No Value	No Valu	No Value	No Val	No Val	0	0
P10768	Cluster of S-formylglutathione hydrolase	ESD	31 kDa	1	1	1	0.9	0.9	1	1.4	0.9	0	0
P35527	Keratin, type I cytoskeletal 9	KRT9	62 kDa	1	1	1	0.9	1	0.8	1.4	0.9	0	0
P40222	Alpha-taxilin	TXLNA	62 kDa	1	1	1	1	1	1	0.9	0.8	0	0
P51659	Peroxisomal multifunctional enzyme type 2	HSD17B4	80 kDa	1	1	1.7	1.3	1.7	1.3	1.3	0.9	0	0
P62873	Cluster of Guanine nucleotide-binding protein G(I)/G(S)/G(	GNB1	37 kDa	1	1	0.8	1.1	1.3	1.2	1.3	0.9	0	0
P78536	Disintegrin and metalloproteinase domain-containing protei	ADAM17	93 kDa	1	1	0.9	0.9	0.9	0.8	1.3	0.9	1	1
Q14554	Protein disulfide-isomerase A5	PDIA5	60 kDa	1	1	0.9	0.9	0.9	0.9	0.8	0.8	1	0
Q16630	Cluster of Cleavage and polyadenylation specificity factor s	CPSF6	59 kDa	1	1	1.2	1.9	1.2	1.1	1.2	0.9	0	0
Q5QPH3	Cluster of Acetyl-coenzyme A synthetase, cytoplasmic	ACSS2	80 kDa	1	1	0.9	0.8	0.9	1	1.2	0.9	0	1
Q86X76	Cluster of Nitrilase homolog 1	NIT1	36 kDa	1	1	1.1	1.1	1.1	0.9	1.2	0.9	0	0
Q8NCL4	Cluster of Polypeptide N-acetylgalactosaminyltransferase 6	GALNT6	71 kDa	1	1	0.8	0.8	0.8	0.8	1.2	0.9	1	0
Q969U7	Proteasome assembly chaperone 2	PSMG2	29 kDa	1	1	1	1.2	0.9	0.9	0.8	0.8	0	0
Q9BYT8	Neurolysin, mitochondrial	NLN	81 kDa	1	1	1.6	1.4	1.4	1.6	1.1	0.9	0	0
P16949	Cluster of Stathmin	STMN1	17 kDa	1	1	1.3	1	1.4	1.4	1.1	0.9	0	0
B3KW71	Cluster of Dihydropteridine reductase	QDPR	22 kDa	1	1	1.3	1.5	1.4	1.3	1.1	0.9	0	0
B7WPD3	Transcription elongation factor B (SIII), polypeptide 2 (18k	TCEB2	18 kDa	1	1	1.3	1.4	1.3	1.2	1.1	0.9	0	0
E7EWZ6	Integrin alpha-V light chain	ITGAV	111 kDa	1	1	1.1	1.2	1.2	1.2	1.1	0.9	0	1
E9PD53	Structural maintenance of chromosomes protein	SMC4	144 kDa	1	1	1.1	0.9	1.2	1.1	1.1	0.9	0	0
H0YB56	Cluster of Protein LYRIC (Fragment)	MTDH	25 kDa	1	1	1.2	1.3	0.9	1.1	1.1	0.9	0	1
J3KNM0	Cluster of Protein phosphatase 1A	PPM1A	51 kDa	1	1	1.1	1.2	1.3	1	1.1	0.9	0	0

O15042	Cluster of U2 snRNP-associated SURP motif-containing pr	U2SURP	118 kDa	1	1	1.1	1	1.1	1	1.1	0.9	0	0
O15427	Cluster of Monocarboxylate transporter 4	SLC16A3	49 kDa	1	1	1.1	1	1.1	1	1.1	0.9	0	1
O60313	Dynamin-like 120 kDa protein, mitochondrial	OPA1	112 kDa	1	1	0.9	0.8	1	1	1.1	0.9	0	0
O75312	Cluster of Zinc finger protein ZPR1	ZNF259	51 kDa	1	1	1	0.9	1.3	0.9	1.1	0.9	0	0
O75663	TIP41-like protein	TIPRL	31 kDa	1	1	1.2	1.1	1.1	0.9	1.1	0.9	0	0
P13984	General transcription factor IIF subunit 2	GTF2F2	28 kDa	1	1	1.1	1.1	1	0.9	1.1	0.9	0	0
Q14696	LDLR chaperone MESD	MESDC2	26 kDa	1	1	0.7	0.9	1	0.9	1.1	0.9	1	0
Q69YN4	Protein virilizer homolog	KIAA1429	202 kDa	1	1	0.9	0.8	1	0.9	1.1	0.9	0	0
Q6DKJ4	Cluster of Nucleoredoxin	NXN	48 kDa	1	1	1.1	1.6	0.9	0.9	1.1	0.9	0	0
Q8IYS1	Peptidase M20 domain-containing protein 2	PM20D2	48 kDa	1	1	0.9	1	0.8	0.9	1.1	0.9	0	0
Q8NCW5	NAD(P)H-hydrate epimerase	APOA1BP	32 kDa	1	1	1.1	1	0.9	0.8	1.1	0.9	1	0
Q96EP5	DAZ-associated protein 1	DAZAP1	43 kDa	1	1	1.1	0.9	0.8	0.8	1.1	0.9	0	0
Q96GG9	Cluster of DCN1-like protein 1	DCUN1D1	30 kDa	1	1	1.2	0.8	1.1	0.7	1.1	0.9	0	0
Q96I24	Far upstream element-binding protein 3	FUBP3	62 kDa	1	1	0.9	0.8	0.8	0.7	1.1	0.9	0	0
Q9BR76	Cluster of Coronin-1B	CORO1B	54 kDa	1	1	1.2	1.3	1.5	1.8	1	0.9	0	0
Q9HCD5	Nuclear receptor coactivator 5	NCOA5	66 kDa	1	1	2.6	2.5	1.9	1.7	1	0.9	0	0
Q9UHJ6	Sedoheptulokinase	SHPK	51 kDa	1	1	1.4	1.3	1.4	1.5	1	0.9	0	0
Q96FJ2	Cluster of Dynein light chain 2, cytoplasmic	DYNLL2	10 kDa	1	1	1.4	1.2	1.4	1.4	1	0.9	0	0
O60518	Ran-binding protein 6	RANBP6	125 kDa	1	1	1.6	1.4	1.4	1.3	1	0.9	0	0
B3KXH8	Sorting nexin-9	SNX9	46 kDa	1	1	1.1	1	1.4	1.3	1	0.9	0	0
E9PB90	Hexokinase-2	HK2	99 kDa	1	1	1.6	1.6	1.4	1.2	1	0.9	0	0
F5H6I0	Beta-2-microglobulin form pI 5.3	B2M	14 kDa	1	1	1.3	1.3	1.3	1.2	1	0.9	1	0
O60476	Mannosyl-oligosaccharide 1,2-alpha-mannosidase IB	MAN1A2	73 kDa	1	1	1.1	1.2	1.3	1.2	1	0.9	0	1
O94973	Cluster of AP-2 complex subunit alpha-2	AP2A2	104 kDa	1	1	1.5	1	1.3	1.2	1	0.9	0	0
P12532	Cluster of Creatine kinase U-type, mitochondrial	CKMT1A	47 kDa	1	1	1.3	1.3	1.2	1.2	1	0.9	1	0
P13807	Cluster of Glycogen [starch] synthase, muscle	GYS1	84 kDa	1	1	1.2	1.2	1.2	1.2	1	0.9	0	0
P15514	Amphiregulin	AREG	28 kDa	1	1	1.1	1.1	1.2	1.2	1	0.9	0	1
P19876	Cluster of C-X-C motif chemokine 3	CXCL3	11 kDa	1	1	1.4	1.3	1.2	1.1	1	0.9	3	0
P31040	Succinate dehydrogenase [ubiquinone] flavoprotein subunit	SDHA	73 kDa	1	1	1.2	1.2	1.2	1.1	1	0.9	0	0
P48506	Glutamate--cysteine ligase catalytic subunit	GCLC	73 kDa	1	1	1.4	1.2	1.1	1.1	1	0.9	0	1
P53367	Isoform A of Arfaptin-1	ARFIP1	39 kDa	1	1	1.3	1.2	1.1	1.1	1	0.9	0	0
P61086	Cluster of Ubiquitin-conjugating enzyme E2 K	UBE2K	22 kDa	1	1	0.8	0.8	1.1	1.1	1	0.9	0	0
P63151	Cluster of Serine/threonine-protein phosphatase 2A 55 kDa	PPP2R2A	52 kDa	1	1	1.2	1.3	1.4	1	1	0.9	0	0
Q13162	Cluster of Peroxiredoxin-4	PRDX4	31 kDa	1	1	1.9	1.5	1.2	1	1	0.9	1	0
Q5VU97	VWFA and cache domain-containing protein 1	CACHD1	142 kDa	1	1	1.7	1.3	1.1	1	1	0.9	0	1
Q8N5I2	Arrestin domain-containing protein 1	ARRDC1	46 kDa	1	1	1	1.2	1.1	1	1	0.9	0	0
Q96DG6	Carboxymethylenebutenolidase homolog	CMBL	28 kDa	1	1	1.1	1.1	1.1	1	1	0.9	0	0

C9J5C3	Programmed cell death protein 10 (Fragment)	PDCD10	24 kDa	1	1	0.8	0.7	1.1	1	1	0.9	0	0
C9JQD4	Cluster of Peptidyl-prolyl cis-trans isomerase (Fragment)	PPIH	16 kDa	1	1	1	1.1	1	1	1	0.9	0	0
D3DQV9	Cluster of Eukaryotic translation initiation factor 4 gamma :	EIF4G2	102 kDa	1	1	1	1.1	1	1	1	0.9	0	0
E7EP65	Cluster of Abl interactor 2 (Fragment)	ABI2	53 kDa	1	1	1.2	1	1	1	1	0.9	0	0
H7C0P6	Cluster of Mitogen-activated protein kinase kinase kinase k	MAP4K4	121 kDa	1	1	1	1	1	1	1	0.9	1	0
O43719	HIV Tat-specific factor 1	HTATSF1	86 kDa	1	1	1	0.9	1	1	1	0.9	0	0
O43852	Cluster of Calumenin	CALU	37 kDa	1	1	0.9	0.9	1	1	1	0.9	1	0
O75935	Dynactin subunit 3	DCTN3	21 kDa	1	1	1.2	1.3	0.9	1	1	0.9	0	0
P19784	Casein kinase II subunit alpha'	CSNK2A2	41 kDa	1	1	1	1	1	0.9	1	0.9	0	0
P22695	Cytochrome b-c1 complex subunit 2, mitochondrial	UQCRC2	48 kDa	1	1	1	0.9	1	0.9	1	0.9	0	0
P30405	Peptidyl-prolyl cis-trans isomerase F, mitochondrial	PPIF	22 kDa	1	1	0.9	0.9	1	0.9	1	0.9	0	0
P48637	Cluster of Glutathione synthetase	GSS	52 kDa	1	1	0.9	0.9	1	0.9	1	0.9	0	0
P51532	Cluster of Transcription activator BRG1	SMARCA4	185 kDa	1	1	1.3	1.1	0.9	0.9	1	0.9	0	0
P54709	Cluster of Sodium/potassium-transporting ATPase subunit l	ATP1B3	32 kDa	1	1	1	1	0.9	0.9	1	0.9	0	1
P58546	Myotrophin	MTPN	13 kDa	1	1	1.1	0.9	0.9	0.9	1	0.9	0	0
Q13287	N-myc-interactor	NMI	35 kDa	1	1	0.9	0.9	0.9	0.9	1	0.9	0	0
Q8NHP8	Cluster of Putative phospholipase B-like 2	PLBD2	65 kDa	1	1	1	1	0.8	0.9	1	0.9	1	0
Q8WWM7	Cluster of Ataxin-2-like protein	ATXN2L	113 kDa	1	1	0.5	0.5	1.2	0.8	1	0.9	0	1
Q9BRX5	DNA replication complex GINS protein PSF3	GINS3	25 kDa	1	1	0.9	0.9	1	0.8	1	0.9	0	0
Q9BVG4	Cluster of UPF0368 protein Cxorf26	PBDC1	26 kDa	1	1	0.9	0.9	0.9	0.8	1	0.9	0	0
Q9BZF9	Uveal autoantigen with coiled-coil domains and ankyrin rep	UACA	163 kDa	1	1	0.9	0.9	0.9	0.8	1	0.9	0	0
Q9H2M9	Cluster of Rab3 GTPase-activating protein non-catalytic sul	RAB3GAP2	156 kDa	1	1	1	0.8	0.9	0.8	1	0.9	0	0
Q9H6T3	RNA polymerase II-associated protein 3	RPAP3	76 kDa	1	1	0.8	0.8	0.9	0.8	1	0.9	0	0
Q9P2I0	Cluster of Cleavage and polyadenylation specificity factor s	CPSF2	88 kDa	1	1	0.8	0.8	0.9	0.8	1	0.9	0	0
Q9UBL3	Cluster of Set1/Ash2 histone methyltransferase complex sul	ASH2L	69 kDa	1	1	0.9	0.9	0.8	0.8	1	0.9	0	0
Q9UBQ7	Cluster of Glyoxylate reductase/hydroxypyruvate reductase	GRHPR	36 kDa	1	1	0.9	0.9	0.8	0.8	1	0.9	0	0
Q9UDT6	Cluster of CAP-Gly domain-containing linker protein 2	CLIP2	116 kDa	1	1	0.9	0.9	0.8	0.8	1	0.9	0	0
P18077	60S ribosomal protein L35a	RPL35A	13 kDa	1	1	0.9	0.8	0.8	0.8	1	0.9	0	0
Q86UD1	Out at first protein homolog	OAF	31 kDa	1	1	0.9	0.9	0.8	0.7	1	0.9	1	0
Q9BVM2	Cluster of Protein DPCD	DPCD	23 kDa	1	1	0.8	0.8	0.8	0.7	1	0.9	0	0
A2A274	Aconitase 2, mitochondrial	ACO2	88 kDa	1	1	0.6	0.8	0.8	0.7	1	0.9	0	0
A2A2V4	Cluster of Vascular endothelial growth factor	VEGFA	19 kDa	1	1	0.7	0.6	0.8	0.7	1	0.9	1	0
E7EP23	Cluster of Elongator complex protein 2	ELP2	100 kDa	1	1	0.7	0.7	0.7	0.7	1	0.9	1	0
F5GZ78	Cluster of Paxillin	PXN	64 kDa	No Va	No Va	No Value	No Value	No Valu	No Value	No Val	No Val	0	0
F5H025	Cluster of Neural cell adhesion molecule L1	L1CAM	140 kDa	1	1	0.6	0.6	0.6	0.7	1	0.9	1	1
F5H8B1	Cluster of Ethanolamine-phosphate cytidylyltransferase	PCYT2	46 kDa	1	1	1.2	1.1	1	1	1.2	1.3	0	0
G5E9W3	Cleavage and polyadenylation specific factor 3, 73kDa, isof	CPSF3	73 kDa	1	1	1.5	1.5	1.8	1.8	0.9	0.9	0	0

J3KPM9	Signal transducer and activator of transcription 1-alpha/beta	STAT1	83 kDa	1	1	1.7	1.3	1.6	1.8	0.9	0.9	0	0
O00170	AH receptor-interacting protein	AIP	38 kDa	1	1	1.2	1.3	1.6	1.7	0.9	0.9	0	0
O95630	STAM-binding protein	STAMBP	48 kDa	1	1	1	1	0.9	0.9	0.8	0.8	0	0
P18084	Integrin beta-5	ITGB5	88 kDa	1	1	1.4	1.5	1.5	1.6	0.9	0.9	0	1
P51665	Cluster of 26S proteasome non-ATPase regulatory subunit 7	PSMD7	37 kDa	1	1	2	1.5	1.5	1.5	0.9	0.9	0	0
P62263	40S ribosomal protein S14	RPS14	16 kDa	1	1	1	1	1.6	1.4	0.9	0.9	0	0
P62312	U6 snRNA-associated Sm-like protein LSm6	LSM6	9 kDa	1	1	1.4	1.3	1.5	1.4	0.9	0.9	0	0
Q01650	Large neutral amino acids transporter small subunit 1	SLC7A5	55 kDa	1	1	1.6	1.6	1.4	1.4	0.9	0.9	0	1
Q14997	Proteasome activator complex subunit 4	PSME4	211 kDa	1	1	1.5	1.6	1.4	1.4	0.9	0.9	0	0
Q16831	Cluster of Uridine phosphorylase 1	UPP1	34 kDa	1	1	1.5	1.6	1.3	1.4	0.9	0.9	0	0
Q7RTV0	PHD finger-like domain-containing protein 5A	PHF5A	12 kDa	1	1	1.3	1.3	1.3	1.4	0.9	0.9	0	0
Q8WX92	Negative elongation factor B	NELFB	66 kDa	1	1	1.7	1.4	1.1	1.4	0.9	0.9	0	0
Q92614	Unconventional myosin-XVIIIa	MYO18A	233 kDa	1	1	1.6	1.4	1.5	1.3	0.9	0.9	0	0
Q96T51	Cluster of RUN and FYVE domain-containing protein 1	RUFY1	80 kDa	1	1	1.6	1.4	1.4	1.3	0.9	0.9	0	0
Q9BTY2	Plasma alpha-L-fucosidase	FUCA2	54 kDa	1	1	1.4	1.3	1.4	1.3	0.9	0.9	1	0
Q9Y6K5	Cluster of 2'-5'-oligoadenylate synthase 3	OAS3	121 kDa	1	1	1	1.2	1.4	1.3	0.9	0.9	0	0
P62851	40S ribosomal protein S25	RPS25	14 kDa	1	1	1.3	1.3	1.3	1.3	0.9	0.9	0	0
E9PL57	Cluster of Protein NEDD8-MDP1 (Fragment)	NEDD8-MDP	20 kDa	1	1	1.1	1.1	1.1	1.3	0.9	0.9	0	0
G3V1C4	Cluster of Cell division cycle 27, isoform CRA_b	CDC27	93 kDa	1	1	1.3	1	1.7	1.2	0.9	0.9	0	1
H0YGL6	Cluster of Ras-related protein Rab-6A (Fragment)	RAB6A	23 kDa	1	1	1.6	1.3	1.3	1.2	0.9	0.9	0	0
O75976	Carboxypeptidase D	CPD	153 kDa	1	1	1.3	1.3	1.3	1.2	0.9	0.9	0	1
O95347	Cluster of Structural maintenance of chromosomes protein 2	SMC2	136 kDa	1	1	1.6	1.4	1.2	1.2	0.9	0.9	0	0
P06132	Cluster of Uroporphyrinogen decarboxylase	UROD	41 kDa	1	1	1.4	1.4	1.2	1.2	0.9	0.9	0	0
P17480	Cluster of Nucleolar transcription factor 1	UBTF	89 kDa	1	1	1.2	1.2	1.2	1.2	0.9	0.9	0	0
P39060	Collagen alpha-1(XVIII) chain	COL18A1	178 kDa	1	1	1.1	1.2	1.2	1.2	0.9	0.9	1	0
P49755	Transmembrane emp24 domain-containing protein 10	TMED10	25 kDa	1	1	1.1	1.1	1.2	1.2	0.9	0.9	1	1
Q13155	Aminoacyl tRNA synthase complex-interacting multifunctio	AIMP2	35 kDa	1	1	1.2	1.6	1.1	1.2	0.9	0.9	0	0
Q6NXG1	Cluster of Epithelial splicing regulatory protein 1	ESRP1	76 kDa	1	1	1.1	1.1	1.1	1.2	0.9	0.9	1	0
Q8NEZ5	F-box only protein 22	FBXO22	45 kDa	1	1	1.2	0.9	1.1	1.2	0.9	0.9	0	0
Q99614	Tetratricopeptide repeat protein 1	TTC1	34 kDa	1	1	1.1	1.1	1	1.2	0.9	0.9	0	0
Q9H2J4	Phosducin-like protein 3	PDCL3	28 kDa	1	1	1.1	1.1	1	1.2	0.9	0.9	0	0
Q9NUQ9	Cluster of Protein FAM49B	FAM49B	37 kDa	1	1	0.8	0.9	0.9	1.2	0.9	0.9	0	0
Q9NWT1	p21-activated protein kinase-interacting protein 1	PAK1IP1	44 kDa	1	1	1.4	1.4	1.3	1.1	0.9	0.9	0	0
Q9Y5X3	Sorting nexin-5	SNX5	47 kDa	1	1	1.3	1.3	1.2	1.1	0.9	0.9	0	0
O15145	Actin-related protein 2/3 complex subunit 3	ARPC3	21 kDa	1	1	1.1	1	1.2	1.1	0.9	0.9	0	0
A8MZB2	N-acetyltransferase 5	NAA20	19 kDa	1	1	1	1.1	1.1	1.1	0.9	0.9	0	0
B4DL14	ATP synthase gamma chain	ATP5C1	28 kDa	1	1	1.1	1	1.1	1.1	0.9	0.9	0	0

C9JG97	Angio-associated migratory cell protein	AAMP	45 kDa	1	1	1.3	1.3	1	1.1	0.9	0.9	0	0
E7EX17	Cluster of Uncharacterized protein	EIF4B	70 kDa	1	1	1	1.1	1	1.1	0.9	0.9	0	0
F6SBX2	Isoleucine--tRNA ligase, mitochondrial	IARS2	106 kDa	1	1	0.9	1.1	1	1.1	0.9	0.9	0	0
F8WCF6	Cluster of Actin-related protein 2/3 complex subunit 4	ARPC4	21 kDa	1	1	1.1	1	1	1.1	0.9	0.9	0	0
H3BLU7	Cluster of Aflatoxin B1 aldehyde reductase member 2 (Frag	AKR7A2	35 kDa	1	1	0.9	1	1	1.1	0.9	0.9	0	0
H3BUQ2	Cluster of 2-oxoglutarate and iron-dependent oxygenase do	OGFOD1	58 kDa	1	1	0.8	0.9	1	1.1	0.9	0.9	0	0
O95817	BAG family molecular chaperone regulator 3	BAG3	62 kDa	1	1	1	0.9	0.9	1.1	0.9	0.9	0	0
O95861	Cluster of Isoform 2 of 3'(2'),5'-bisphosphate nucleotidase 1	BPNT1	36 kDa	1	1	1.2	1	0.8	1.1	0.9	0.9	0	0
P00736	Cluster of Complement C1r subcomponent	C1R	80 kDa	1	1	0.7	0.6	0.8	0.6	0.7	0.7	1	0
P20020	Cluster of Plasma membrane calcium-transporting ATPase	ATP2B1	139 kDa	1	1	1.1	1	1.3	1	0.9	0.9	0	1
P46779	Cluster of 60S ribosomal protein L28	RPL28	16 kDa	1	1	1.2	1.2	1.2	1	0.9	0.9	0	0
P61254	Cluster of 60S ribosomal protein L26	RPL26	17 kDa	1	1	0.8	0.8	1.1	1	0.9	0.9	0	0
Q13438	Protein OS-9	OS9	76 kDa	1	1	1.2	1.2	1	1	0.9	0.9	1	0
Q29RF7	Cluster of Sister chromatid cohesion protein PDS5 homolog	PDS5A	151 kDa	1	1	1.1	1.2	1	1	0.9	0.9	0	0
Q5VW32	BRO1 domain-containing protein BROX	BROX	46 kDa	1	1	1.1	1.1	1	1	0.9	0.9	0	0
Q8IUI8	Cytokine receptor-like factor 3	CRLF3	50 kDa	1	1	1.1	1.1	1	1	0.9	0.9	0	1
Q99961	Cluster of Endophilin-A2	SH3GL1	41 kDa	1	1	1	1	1	1	0.9	0.9	0	0
Q9BWJ5	Splicing factor 3B subunit 5	SF3B5	10 kDa	1	1	1	1	1	1	0.9	0.9	0	0
Q9HAB8	Phosphopantothenate--cysteine ligase	PPCS	34 kDa	1	1	0.9	1	1	1	0.9	0.9	0	0
Q9NR28	Cluster of Diablo homolog, mitochondrial	DIABLO	27 kDa	1	1	0.9	1	1	1	0.9	0.9	1	1
Q9NWH9	SAFB-like transcription modulator	SLTM	117 kDa	1	1	0.9	0.9	1	1	0.9	0.9	1	0
Q9NZB2	Cluster of Isoform F of Constitutive coactivator of PPAR-ga	FAM120A	125 kDa	1	1	1.1	1.1	0.9	1	0.9	0.9	0	0
Q9ULF5	Zinc transporter ZIP10	SLC39A10	94 kDa	1	1	1.1	1.1	0.9	1	0.9	0.9	1	1
P62079	Tetraspanin-5	TSPAN5	30 kDa	1	1	0.8	0.9	0.9	1	0.9	0.9	0	1
Q8TEA8	D-tyrosyl-tRNA(Tyr) deacylase 1	DTD1	23 kDa	1	1	1	1.2	0.8	1	0.9	0.9	0	0
E9PEB9	Cluster of Dystonin	DST	881 kDa	1	1	0	0.6	No Value	0.5	0	1	1	0
B4E1D7	Pyridoxine-5'-phosphate oxidase	PNPO	28 kDa	1	1	0.8	1.2	0.8	1	0.9	0.9	0	0
C9J2Y9	DNA-directed RNA polymerase	POLR2B	133 kDa	1	1	1	1.2	1.1	0.9	0.9	0.9	0	0
D6RF12	Cluster of Phosphoacetylglucosamine mutase	PGM3	62 kDa	1	1	1	0.6	1.1	0.9	0.9	0.9	0	0
F5H2M7	Cluster of Serine/threonine-protein kinase WNK1	WNK1	305 kDa	1	1	2	1.2	1	0.9	0.9	0.9	1	0
G3V180	Cluster of Dipeptidyl peptidase 3	DPP3	84 kDa	1	1	1.2	1.2	1	0.9	0.9	0.9	0	0
H3BP91	Cluster of Enoyl-CoA delta isomerase 1, mitochondrial (Fra	ECI1	32 kDa	No Va	No Va	No Value	No Value	No Value	No Value	No Val	No Val	0	0
O43592	Exportin-T	XPOT	110 kDa	1	1	1.3	1.1	1	0.9	0.9	0.9	0	0
O60678	Protein arginine N-methyltransferase 3	PRMT3	60 kDa	1	1	1.1	1.1	1	0.9	0.9	0.9	0	1
O60869	Endothelial differentiation-related factor 1	EDF1	16 kDa	1	1	0.9	1.1	1	0.9	0.9	0.9	0	0
O95602	DNA-directed RNA polymerase I subunit RPA1	POLR1A	195 kDa	1	1	1.1	0.9	1	0.9	0.9	0.9	0	0
P29353	Cluster of SHC-transforming protein 1	SHC1	63 kDa	1	1	1	0.9	1	0.9	0.9	0.9	0	0

P33316	Cluster of Isoform 2 of Deoxyuridine 5'-triphosphate nucleo	DUT	18 kDa	1	1	1.2	1.3	0.9	0.9	0.9	0.9	0	0
P48739	Phosphatidylinositol transfer protein beta isoform	PITPNB	32 kDa	1	1	0.9	1.1	0.9	0.9	0.9	0.9	0	0
P51114	Cluster of Fragile X mental retardation syndrome-related pr	FXR1	70 kDa	No Va	No Va	No Value	No Value	No Value	No Value	No Val	No Val	1	0
Q13242	Serine/arginine-rich splicing factor 9	SRSF9	26 kDa	1	1	1.5	1	0.9	0.9	0.9	0.9	0	0
Q15435	Cluster of Protein phosphatase 1 regulatory subunit 7	PPP1R7	42 kDa	1	1	1.1	1	0.9	0.9	0.9	0.9	0	0
Q6NZI2	Polymerase I and transcript release factor	PTRF	43 kDa	1	1	1.1	1	0.9	0.9	0.9	0.9	0	0
Q6P9A2	Putative polypeptide N-acetylgalactosaminyltransferase-like	GALNT18	70 kDa	1	1	1	1	0.9	0.9	0.9	0.9	0	1
Q96G03	Cluster of Phosphoglucomutase-2	PGM2	68 kDa	1	1	0.9	1	0.9	0.9	0.9	0.9	0	0
Q96MX6	Cluster of WD repeat-containing protein 92	WDR92	40 kDa	1	1	0.9	1	0.9	0.9	0.9	0.9	0	0
Q9BZK7	Cluster of F-box-like/WD repeat-containing protein TBL1X	TBL1XR1	56 kDa	1	1	0.9	0.9	0.9	0.9	0.9	0.9	0	0
Q9H1E3	Nuclear ubiquitous casein and cyclin-dependent kinase subs	NUCKS1	27 kDa	1	1	0.9	0.9	0.9	0.9	0.9	0.9	0	0
Q9ULE6	Paladin	PALD1	97 kDa	1	1	0.9	0.9	0.9	0.9	0.9	0.9	0	1
Q9Y2W1	Thyroid hormone receptor-associated protein 3	THRAP3	109 kDa	1	1	0.9	0.9	0.9	0.9	0.9	0.9	0	0
Q9Y3U8	Cluster of 60S ribosomal protein L36	RPL36	12 kDa	1	1	0.8	0.6	0.9	0.9	0.9	0.9	0	0
A6NMA8	Cluster of Quinone oxidoreductase-like protein 1	CRYZL1	37 kDa	1	1	1	1.2	0.8	0.9	0.9	0.9	0	0
B0UX83	Cluster of HLA-B associated transcript 3	BAT3	119 kDa	1	1	0.9	0.9	0.8	0.9	0.9	0.9		
B0UZ83	Cluster of Complement C4-A alpha chain	C4A	193 kDa	1	1	0.9	0.9	0.8	0.9	0.9	0.9	1	0
B7Z5W1	Junctional adhesion molecule A	F11R	33 kDa	1	1	0.8	0.8	0.8	0.9	0.9	0.9	0	1
E5RJD8	Cluster of Tubulin-specific chaperone A	TBCA	14 kDa	1	1	0.8	0.7	0.8	0.9	0.9	0.9	0	0
E9PGZ1	Cluster of Caldesmon	CALD1	62 kDa	No Va	No Va	No Value	No Value	No Value	No Value	No Val	No Val	0	1
F8VX04	Sodium-coupled neutral amino acid transporter 1	SLC38A1	56 kDa	1	1	1.2	0.9	0.7	0.9	0.9	0.9	0	1
H7C2Q3	26S proteasome non-ATPase regulatory subunit 2 (Fragmen	PSMD2	16 kDa	1	1	0.7	0.8	0.7	0.9	0.9	0.9	0	1
H7C2Q8	EBNA1 binding protein 2, isoform CRA_d	EBNA1BP2	41 kDa	1	1	1.2	0.8	1.2	0.8	0.9	0.9	0	0
J3QS36	Cluster of L-xylulose reductase (Fragment)	DCXR	24 kDa	1	1	0.9	0.9	1	0.8	0.9	0.9	0	0
O14773	Tripeptidyl-peptidase 1	TPP1	61 kDa	1	1	1.2	1.3	0.9	0.8	0.9	0.9	1	0
O43290	U4/U6.U5 tri-snRNP-associated protein 1	SART1	90 kDa	1	1	0.9	0.9	0.9	0.8	0.9	0.9	0	0
O75054	Immunoglobulin superfamily member 3	IGSF3	135 kDa	1	1	0.9	0.9	0.9	0.8	0.9	0.9	0	1
O75150	Cluster of E3 ubiquitin-protein ligase BRE1B	RNF40	114 kDa	1	1	0.7	0.9	0.9	0.8	0.9	0.9	0	0
P11908	Cluster of Isoform 2 of Ribose-phosphate pyrophosphokina	PRPS2	35 kDa	1	1	0.7	0.6	0.9	0.8	0.9	0.9	0	0
P29373	Cellular retinoic acid-binding protein 2	CRABP2	16 kDa	1	1	0.9	0.9	0.8	0.8	0.9	0.9	0	0
P29590	Cluster of Protein PML	PML	98 kDa	No Va	No Va	No Value	No Value	No Value	No Value	No Val	No Val	0	0
Q13033	Striatin-3	STRN3	87 kDa	1	1	0.8	0.9	0.8	0.8	0.9	0.9	0	0
Q13404	Ubiquitin-conjugating enzyme E2 variant 1	UBE2V1	16 kDa	1	1	0.8	0.8	0.8	0.8	0.9	0.9	0	0
Q13443	Disintegrin and metalloproteinase domain-containing protei	ADAM9	91 kDa	1	1	0.7	0.7	0.8	0.8	0.9	0.9	1	1
Q15643	Cluster of Thyroid receptor-interacting protein 11	TRIP11	228 kDa	1	1	0.7	0.7	0.8	0.8	0.9	0.9	0	0
Q53FA7	Quinone oxidoreductase PIG3	TP53I3	36 kDa	1	1	0.7	0.7	0.7	0.8	0.9	0.9	0	0
Q92817	Envoplakin	EVPL	232 kDa	1	1	0.7	0.7	0.7	0.8	0.9	0.9	0	0

Q96EK5	KIF1-binding protein	KIAA1279	72 kDa	1	1	1.1	0.8	1	0.7	0.9	0.9	0	0
Q9BQ52	Cluster of Zinc phosphodiesterase ELAC protein 2	ELAC2	92 kDa	1	1	0.9	0.8	0.8	0.7	0.9	0.9	0	0
Q9BUH6	Uncharacterized protein C9orf142	C9orf142	22 kDa	1	1	0.8	0.7	0.8	0.7	0.9	0.9	0	0
Q9NVM9	Cluster of Protein asunder homolog	Asun	80 kDa	1	1	0.7	0.6	0.8	0.7	0.9	0.9	0	0
Q9UJW0	Cluster of Dynactin subunit 4	DCTN4	52 kDa	1	1	0.8	0.8	0.7	0.7	0.9	0.9	0	0
Q9UPT5	Exocyst complex component 7	EXOC7	83 kDa	1	1	0.8	0.8	0.7	0.7	0.9	0.9	0	0
Q15904	Cluster of V-type proton ATPase subunit S1	ATP6AP1	52 kDa	1	1	0.7	0.8	0.8	0.6	0.9	0.9	1	1
A8MV37	Protein SEC13 homolog	SEC13	34 kDa	1	1	0.8	0.7	0.7	0.6	0.9	0.9	0	0
B4DU58	Macrophage-capping protein	CAPG	36 kDa	1	1	0.8	0.8	0.9	0.8	3.6	2.8	0	0
B5MCF9	Cluster of Pescadillo homolog	PES1	66 kDa	1	1	2.4	2	1.9	1.9	0.8	0.9	0	0
E7EQT4	Cluster of Apoptotic chromatin condensation inducer in the	ACIN1	147 kDa	1	No Va	0.9	No Value	1.3	No Value	0.8	No Val	0	0
E9PHK0	Tetranectin	CLEC3B	18 kDa	1	1	1.9	1.8	1.6	1.6	0.8	0.9	1	0
F8W8Q7	Golgin subfamily A member 4	GOLGA4	262 kDa	1	1	1.4	1.4	1.4	1.4	0.8	0.9	0	0
H0YDW2	Cluster of Liprin-alpha-1 (Fragment)	PPFIA1	42 kDa	1	1	1.2	1.4	1.2	1.3	0.8	0.9	0	1
O00462	Beta-mannosidase	MANBA	101 kDa	1	1	1.2	1.3	1.2	1.3	0.8	0.9	1	0
O43865	Cluster of Putative adenosylhomocysteinase 2	AHCYL1	59 kDa	1	No Va	1.3	No Value	1.4	No Value	1	No Val	0	0
O75717	WD repeat and HMG-box DNA-binding protein 1	WDHD1	126 kDa	1	1	1.5	1.2	1.5	1.2	0.8	0.9	0	1
O95218	Zinc finger Ran-binding domain-containing protein 2	ZRANB2	37 kDa	1	1	1.4	1.1	1.4	1.2	0.8	0.9	0	0
O95352	Cluster of Ubiquitin-like modifier-activating enzyme ATG7	ATG7	78 kDa	1	1	1.2	1.1	1.3	1.2	0.8	0.9	0	0
P08579	U2 small nuclear ribonucleoprotein B"	SNRPB2	25 kDa	1	1	1.1	1.1	1.2	1.2	0.8	0.9	0	0
P19525	Interferon-induced, double-stranded RNA-activated protein	EIF2AK2	62 kDa	1	1	1	1	1.2	1.2	0.8	0.9	0	0
P22087	rRNA 2'-O-methyltransferase fibrillarin	FBL	34 kDa	1	1	1.1	1.1	1.1	1.2	0.8	0.9	0	0
P36955	Cluster of Pigment epithelium-derived factor	SERPINF1	46 kDa	1	1	1.4	1.4	1.2	1.1	0.8	0.9	1	1
P46939	Utrophin	UTRN	394 kDa	1	1	1	1	1.2	1.1	0.8	0.9	0	0
P49773	Cluster of Histidine triad nucleotide-binding protein 1	HINT1	14 kDa	1	1	1.2	1.2	1.1	1.1	0.8	0.9	0	0
P49840	Cluster of Glycogen synthase kinase-3 alpha	GSK3A	51 kDa	1	1	1	1.2	1.1	1.1	0.8	0.9	0	0
P50552	Vasodilator-stimulated phosphoprotein	VASP	40 kDa	1	1	1.1	1	1.1	1.1	0.8	0.9	0	0
P53990	Cluster of Isoform 2 of IST1 homolog	IST1	37 kDa	1	1	1	1	1.1	1.1	0.8	0.9	0	0
Q16222	UDP-N-acetylhexosamine pyrophosphorylase	UAP1	59 kDa	1	1	1	1.2	1	1.1	0.8	0.9	0	0
Q86TU7	Cluster of Histone-lysine N-methyltransferase setd3	SETD3	67 kDa	1	1	1.2	1.1	1	1.1	0.8	0.9	0	0
Q8TEX9	Cluster of Isoform 2 of Importin-4	IPO4	119 kDa	1	1	1.1	1.1	1	1.1	0.8	0.9	0	0
Q9H832	Cluster of Ubiquitin-conjugating enzyme E2 Z	UBE2Z	38 kDa	1	1	1	1.1	1	1.1	0.8	0.9	0	0
Q9HCY8	Protein S100-A14	S100A14	12 kDa	1	1	1	1.1	1	1.1	0.8	0.9	0	0
Q9NP79	Vacuolar protein sorting-associated protein VTA1 homolog	VTA1	34 kDa	1	1	0.9	1.1	1	1.1	0.8	0.9	0	0
Q9NQR4	Cluster of Omega-amidase NIT2	NIT2	31 kDa	1	1	0.9	1	1	1.1	0.8	0.9	1	0
Q9NQT4	Exosome complex component RRP46	EXOSC5	25 kDa	1	1	1.1	1.1	0.9	1.1	0.8	0.9	0	0
Q9NTM9	Copper homeostasis protein cutC homolog	CUTC	29 kDa	1	1	1	1.1	0.9	1.1	0.8	0.9	0	0

Q9UI42	Carboxypeptidase A4	CPA4	47 kDa	1	1	1.3	1.4	1.2	1	0.8	0.9	1	0
Q9UKX7	Isoform 2 of Nuclear pore complex protein Nup50	NUP50	47 kDa	1	1	1.3	1.3	1.1	1	0.8	0.9	0	0
F5GZK1	Cluster of Exostosin-like 2	EXTL2	36 kDa	1	1	0.9	1.2	1	1	0.8	0.9	1	0
F8W7C6	Cluster of 60S ribosomal protein L10	RPL10	19 kDa	1	1	1.1	1.1	1	1	0.8	0.9	0	0
H0YEB7	Cluster of N-acetyl-D-glucosamine kinase (Fragment)	NAGK	20 kDa	No Va	No Va	No Value	No Value	No Value	No Value	No Val	No Val	0	0
I3L3Q4	Glyoxalase domain-containing protein 4 (Fragment)	GLOD4	26 kDa	1	1	1	0.8	0.8	0.8	4	3.6	1	0
O00743	Serine/threonine-protein phosphatase 6 catalytic subunit	PPP6C	35 kDa	1	1	0.9	0.9	1	1	0.8	0.9	0	0
O15511	Actin-related protein 2/3 complex subunit 5	ARPC5	16 kDa	1	1	0.9	1.1	0.9	1	0.8	0.9	0	0
O60216	Double-strand-break repair protein rad21 homolog	RAD21	72 kDa	1	1	0.9	0.8	0.9	1	0.8	0.9	0	0
O60343	Cluster of TBC1 domain family member 4	TBC1D4	147 kDa	1	1	1	0.9	1	0.9	0.8	0.9	0	0
O75934	Pre-mRNA-splicing factor SPF27	BCAS2	26 kDa	1	1	0.8	0.8	1	0.9	0.8	0.9	0	0
O76021	Ribosomal L1 domain-containing protein 1	RSL1D1	55 kDa	1	1	1	1	0.9	0.9	0.8	0.9	0	0
O94992	Cluster of Protein HEXIM1	HEXIM1	41 kDa	1	1	1	1	0.9	0.9	0.8	0.9	0	0
O95453	Cluster of Poly(A)-specific ribonuclease PARN	PARN	73 kDa	1	1	1	1	0.9	0.9	0.8	0.9	0	0
P09455	Retinol-binding protein 1	RBP1	16 kDa	1	1	1	1	0.9	0.9	0.8	0.9	0	0
P12955	Xaa-Pro dipeptidase	PEPD	55 kDa	1	1	0.8	0.8	0.9	0.9	0.8	0.9	0	0
P14550	Alcohol dehydrogenase [NADP(+)]	AKR1A1	37 kDa	1	1	0.8	0.8	0.9	0.9	0.8	0.9	0	0
P36957	Cluster of Dihydrolipoyllysine-residue succinyltransferase c	DLST	49 kDa	1	1	0.6	0.6	0.9	0.9	0.8	0.9	0	0
P42766	60S ribosomal protein L35	RPL35	15 kDa	1	1	1	1.1	0.8	0.9	0.8	0.9	0	0
P52735	Isoform 3 of Guanine nucleotide exchange factor VAV2	VAV2	97 kDa	1	1	0.9	1	0.8	0.9	0.8	0.9	0	0
Q08170	Cluster of Serine/arginine-rich splicing factor 4	SRSF4	57 kDa	1	1	0.9	1	0.8	0.9	0.8	0.9	0	0
Q13426	DNA repair protein XRCC4	XRCC4	38 kDa	1	1	1.1	1.3	0.9	0.8	0.8	0.9	0	0
Q15102	Platelet-activating factor acetylhydrolase IB subunit gamma	PAFAH1B3	26 kDa	1	1	1.1	1.1	0.8	0.8	0.8	0.9	0	0
Q5QPL9	Cluster of RNA binding protein, autoantigenic (HnRNP-ass	RALY	25 kDa	1	1	1.2	0.9	0.8	0.8	0.8	0.9	0	0
Q641Q3	Meteorin-like protein	METRNL	34 kDa	1	1	0.8	0.8	0.8	0.8	0.8	0.9	1	0
Q8N3U4	Isoform 2 of Cohesin subunit SA-2	STAG2	146 kDa	1	1	0.8	0.8	0.8	0.8	0.8	0.9	0	0
Q96SI9	Spermatid perinuclear RNA-binding protein	STRBP	74 kDa	1	1	0.7	0.7	0.8	0.8	0.8	0.9	1	0
Q99417	C-Myc-binding protein	MYCBP	12 kDa	1	1	1.1	1.2	0.7	0.8	0.8	0.9	0	0
Q9H8H0	Cluster of Nucleolar protein 11	NOL11	81 kDa	1	1	1	0.8	0.7	0.8	0.8	0.9	0	0
Q9P2R3	Cluster of Isoform 4 of Ankyrin repeat and FYVE domain-c	ANKFY1	133 kDa	No Va	No Va	No Value	No Value	No Value	No Value	No Val	No Val	0	0
Q9UM47	Neurogenic locus notch homolog protein 3	NOTCH3	244 kDa	1	1	0.6	0.8	0.7	0.8	0.8	0.9	1	1
P67870	Casein kinase II subunit beta	CSNK2B	25 kDa	1	1	0.7	0.9	1	0.7	0.8	0.9	0	0
Q9NQG5	Regulation of nuclear pre-mRNA domain-containing protei	RPRD1B	37 kDa	1	1	0.9	0.9	0.8	0.7	0.8	0.9	0	0
Q9NZZ3	Charged multivesicular body protein 5	CHMP5	25 kDa	1	1	0.7	0.7	0.8	0.7	0.8	0.9	0	0
B5ME91	Programmed cell death protein 4	PDCD4	51 kDa	1	1	0.9	1	0.7	0.7	0.8	0.9	0	0
C9J0D9	Complement receptor type 2	CR2	110 kDa	1	1	0.7	0.6	0.6	0.7	0.8	0.9	0	1
C9JJP5	Protein TFG (Fragment)	TFG	20 kDa	1	1	0.6	0.7	0.5	0.6	0.8	0.9	0	0

F8VPG7	Phosphatidylinositol-binding clathrin assembly protein	PICALM	70 kDa	1	1	0.7	0.6	0.4	0.3	0.8	0.9	0	0
H0YGR4	Oligoribonuclease, mitochondrial (Fragment)	REXO2	22 kDa	1	1	1.3	1.6	1.7	1.9	0.7	0.9	0	0
J3KQ34	COP9 signalosome complex subunit 7b	COPS7B	26 kDa	1	1	1.5	1.5	1.5	1.4	0.7	0.9	0	0
J3QRU1	Cluster of Tyrosine-protein kinase Yes	YES1	61 kDa	1	1	1.1	0.9	1.4	1.4	0.7	0.9	0	0
O75688	Cluster of Isoform 4 of Protein phosphatase 1B	PPM1B	42 kDa	1	1	1	1.6	1.1	1.4	0.7	0.9	0	0
O94903	Cluster of Proline synthase co-transcribed bacterial homolo;	PROSC	30 kDa	1	1	0.9	1.1	0.8	1.2	0.7	0.9	0	0
O95486	Protein transport protein Sec24A	SEC24A	120 kDa	1	1	1.1	1.3	1.1	1	0.7	0.9	0	0
P11441	Ubiquitin-like protein 4A	UBL4A	18 kDa	1	1	1	1.5	0.8	1	0.7	0.9	0	0
P15559	NAD(P)H dehydrogenase [quinone] 1	NQO1	31 kDa	1	1	1.1	0.8	0.8	1	0.7	0.9	0	0
P25445	Tumor necrosis factor receptor superfamily member 6	FAS	38 kDa	1	1	1	1.1	0.7	1	0.7	0.9	1	1
P29992	Cluster of Guanine nucleotide-binding protein subunit alpha	GNA11	42 kDa	1	1	1	0.7	1.4	0.9	0.7	0.9	0	0
P36959	GMP reductase 1	GMPR	37 kDa	1	1	0.8	0.7	1	0.9	0.7	0.9	0	0
P49207	60S ribosomal protein L34	RPL34	13 kDa	1	1	0.6	0.7	0.6	0.7	0.7	0.9	0	0
P55809	Cluster of Succinyl-CoA:3-ketoacid coenzyme A transferas	OXCT1	56 kDa	1	1	0.5	0.7	0.4	0.6	0.7	0.9	1	0
P61586	Cluster of Transforming protein RhoA	RHOA	22 kDa	1	1	1.5	2	1.4	1.5	0.6	0.9	0	0
Q02880	Cluster of DNA topoisomerase 2-beta	TOP2B	183 kDa	1	1	0.7	1.1	0.9	1.5	0.6	0.9	0	0
Q13232	Nucleoside diphosphate kinase 3	NME3	19 kDa	1	1	1	1	1.2	1.3	0.6	0.9	1	0
Q5JR95	40S ribosomal protein S8	RPS8	22 kDa	1	1	0.8	0.9	0.8	1	0.6	0.9	0	0
Q6IA69	Cluster of Glutamine-dependent NAD(+) synthetase	NADSYN1	79 kDa	1	1	0.7	1	0.7	1	0.6	0.9	0	0
Q6PKG0	Cluster of La-related protein 1	LARP1	124 kDa	No Va	No Va	No Value	No Value	No Value	No Value	No Val	No Val	0	0
Q8TAF3	Cluster of WD repeat-containing protein 48	WDR48	76 kDa	1	1	0.9	0.9	1.2	0.9	0.6	0.9	0	0
Q99575	Ribonucleases P/MRP protein subunit POP1	POP1	115 kDa	1	1	1	0.9	1.1	0.9	0.6	0.9	0	0
Q9BQT9	Calsyntenin-3	CLSTN3	106 kDa	1	1	1.1	1.3	0.9	0.9	0.6	0.9	1	1
Q9BTE6	Cluster of Isoform 3 of Alanyl-tRNA editing protein Aarsd1	AARSD1	66 kDa	1	1	0.9	0.9	0.6	0.9	0.6	0.9	0	0
Q9H0C8	Integrin-linked kinase-associated serine/threonine phosphata	ILKAP	43 kDa	1	1	1.6	1.5	1.3	1.2	1.6	1.2	0	0
Q9NPD3	Exosome complex component RRP41	EXOSC4	26 kDa	1	1	0.8	0.9	0.8	0.8	0.9	0.9	0	0
Q9NZL4	Hsp70-binding protein 1	HSPBP1	39 kDa	1	1	1	1.1	1	1	1	0.9	0	0
Q9UI12	V-type proton ATPase subunit H	ATP6V1H	56 kDa	1	1	0.8	0.8	0.9	0.9	0.5	0.5	0	0
Q9Y608	Cluster of Leucine-rich repeat flightless-interacting protein	LRRFIP2	82 kDa	No Va	No Va	No Value	No Value	No Value	No Value	No Val	No Val	0	0
B3KUK2	Cluster of Superoxide dismutase	SOD2	20 kDa	1	1	1.2	1.1	2.1	2.2	1	1	0	0
Q9NWB6	Cluster of Arginine and glutamate-rich protein 1	ARGLU1	33 kDa	1	1	1	0.8	1.4	1.1	1.5	1	0	0
A6NM69	Cluster of Non-specific lipid-transfer protein	SCP2	54 kDa	1	1	1.4	0.9	1.2	0.9	1.5	1	0	0
B7Z6H3	Cluster of Peptidyl-prolyl cis-trans isomerase FKBP9	FKBP9	69 kDa	1	1	0.8	0.8	0.9	1	1.6	1.6	1	0
C0IMW5	SWI/SNF-related matrix-associated actin-dependent regulat	SMARCE1	38 kDa	1	1	0.7	0.9	1.2	1.3	1.4	1	0	0
E9PNW4	CD59 glycoprotein	CD59	12 kDa	1	1	1.6	1.3	1.8	1.1	1.4	1	1	1
F5GX23	26S proteasome non-ATPase regulatory subunit 9	PSMD9	20 kDa	1	1	1.4	1.2	1.1	1.1	1.4	1	0	0
G3V3G9	Cluster of DDB1- and CUL4-associated factor 8	DCAF8	85 kDa	1	1	1.1	1.1	0.8	0.9	1.4	1	0	0

G3V5T9	Cluster of Cyclin-dependent kinase 2	CDK2	39 kDa	1	1	1.2	1	1.2	1	1.1	1	0	0
H0Y512	Cluster of Adipocyte plasma membrane-associated protein (	APMAP	45 kDa	No Va	No Va	No Value	No Value	No Valu	No Value	No Val	No Val	0	1
H0YKF0	Cluster of Electron transfer flavoprotein subunit alpha, mitc	ETFA	30 kDa	1	1	1.1	1	1.5	1.3	1.3	1	0	0
J3KN08	Cluster of Matrilin-2	MATN2	107 kDa	No Va	1	No Value	0.5	No Valu	0.6	No Val	0.7	1	0
J3KNP4	Cluster of Semaphorin-4B	SEMA4B	93 kDa	1	1	1.4	1.1	1.7	1.1	1.3	1	1	1
O00291	Cluster of Huntingtin-interacting protein 1	HIP1	116 kDa	1	1	1.4	1.3	1	1.1	1.3	1	0	0
O43657	Tetraspanin-6	TSPAN6	28 kDa	1	1	0.8	0.7	1.1	1	1.3	1	0	1
O75351	Vacuolar protein sorting-associated protein 4B	VPS4B	49 kDa	1	1	1.4	1	1.1	0.9	1.3	1	0	0
P03950	Angiogenin	ANG	17 kDa	1	1	1.1	1.1	0.9	0.9	1.3	1	1	0
P04181	Ornithine aminotransferase, mitochondrial	OAT	49 kDa	1	1	1	0.9	1	0.8	1.3	1	0	0
P05026	Sodium/potassium-transporting ATPase subunit beta-1	ATP1B1	35 kDa	1	1	1.1	1	0.9	0.8	1.3	1	0	1
P13674	Isoform 2 of Prolyl 4-hydroxylase subunit alpha-1	P4HA1	61 kDa	1	1	0.9	1	0.9	0.7	1.3	1	1	0
P24928	DNA-directed RNA polymerase II subunit RPB1	POLR2A	217 kDa	1	1	1	0.6	0.7	0.6	1.3	1	0	0
P31930	Cytochrome b-c1 complex subunit 1, mitochondrial	UQCRC1	53 kDa	1	1	1.5	1.3	1.7	1.5	1.2	1	0	0
P78345	Ribonuclease P protein subunit p38	RPP38	32 kDa	1	1	1.3	1.4	1.4	1.4	1.2	1	0	0
Q01780	Cluster of Exosome component 10	EXOSC10	101 kDa	1	1	1.2	1.1	1.1	1.2	1.2	1	0	0
Q02750	Dual specificity mitogen-activated protein kinase kinase 1	MAP2K1	43 kDa	1	1	1	0.9	1.3	1.1	1.2	1	0	0
Q12841	Cluster of Follistatin-related protein 1	FSTL1	35 kDa	1	1	1.3	1.2	1.2	1.1	1.2	1	1	0
Q13409	Cluster of Cytoplasmic dynein 1 intermediate chain 2	DYNC1I2	71 kDa	No Va	No Va	No Value	No Value	No Valu	No Value	No Val	No Val	0	0
Q5T8P6	Cluster of RNA-binding protein 26	RBM26	114 kDa	1	1	1.4	1.5	1.1	1.1	1.2	1	0	0
Q5VTR2	E3 ubiquitin-protein ligase BRE1A	RNF20	114 kDa	1	1	1.2	1.1	1.1	1.1	1.2	1	0	0
Q68E01	Isoform 2 of Integrator complex subunit 3	INTS3	118 kDa	1	1	1.2	1.1	1	1	1.2	1	1	0
Q99538	Cluster of Legumain	LGMN	49 kDa	1	1	0.7	0.9	1	0.9	1.2	1	1	0
Q9H444	Charged multivesicular body protein 4b	CHMP4B	25 kDa	1	1	1	1.2	0.9	0.9	1.2	1	0	0
Q9UQ35	Serine/arginine repetitive matrix protein 2	SRRM2	300 kDa	1	1	1.1	1.1	0.9	0.9	1.2	1	0	0
Q9Y287	Integral membrane protein 2B	ITM2B	30 kDa	1	1	1.1	1	0.9	0.9	1.2	1	0	1
Q9Y2T2	Cluster of AP-3 complex subunit mu-1	AP3M1	47 kDa	1	1	1	1	0.9	0.9	1.2	1	0	0
Q9Y3B4	Pre-mRNA branch site protein p14	SF3B14	15 kDa	1	1	1	0.9	0.9	0.9	1.2	1	0	0
Q9Y5P6	Mannose-1-phosphate guanyltransferase beta	GMPPB	40 kDa	1	1	1.5	1.1	0.8	0.9	1.2	1	0	0
Q5TFE4	Cluster of 5'-nucleotidase domain-containing protein 1	NT5DC1	52 kDa	1	1	1	0.9	0.8	0.9	1.2	1	0	0
Q7LBR1	Charged multivesicular body protein 1b	CHMP1B	22 kDa	1	1	0.7	0.7	1.1	0.8	1.2	1	0	0
H9KV70	Neutrophil gelatinase-associated lipocalin	LCN2	23 kDa	1	1	1.1	0.7	1	0.6	1.2	1	1	0
B4E1K7	Cluster of Stomatin-like protein 2	STOML2	33 kDa	1	1	0.6	0.5	0.6	0.4	1.2	1	1	0
C9JA08	60S ribosomal export protein NMD3	NMD3	60 kDa	1	1	1.5	1.6	1.7	1.4	1.1	1	0	0
E7EM93	Peroxisomal NADH pyrophosphatase NUDT12	NUDT12	50 kDa	1	1	1.4	1.4	1.3	1.4	1.1	1	0	0
E9PB02	Ribonuclease P protein subunit p30	RPP30	36 kDa	1	1	1.7	1.5	1.7	1.3	1.1	1	0	0
F8VQE1	Cluster of LIM domain and actin-binding protein 1	LIMA1	67 kDa	No Va	No Va	No Value	No Value	No Valu	No Value	No Val	No Val	0	0

H3BQH0	Cluster of Calponin-2 (Fragment)	CNN2	23 kDa	1	1	1.4	1.2	1.3	1.3	1.1	1	0	0
O15498	Synaptobrevin homolog YKT6	YKT6	22 kDa	1	1	1.1	1	1.3	1.3	1.1	1	0	0
P15121	Aldose reductase	AKR1B1	36 kDa	1	1	1.3	1.2	1.2	1.3	1.1	1	0	0
P19838	Cluster of Nuclear factor NF-kappa-B p105 subunit	NFKB1	105 kDa	1	1	1.5	1.3	1.3	1.2	1.1	1	0	0
P24347	Cluster of Stromelysin-3	MMP11	55 kDa	1	1	1.1	1.2	1.3	1.2	1.1	1	1	0
P26368	Splicing factor U2AF 65 kDa subunit	U2AF2	54 kDa	1	1	0.9	0.8	1.3	1.2	1.1	1	0	0
P30533	Alpha-2-macroglobulin receptor-associated protein	LRPAP1	41 kDa	1	1	1.3	1.3	1	1.2	1.1	1	1	0
P40939	Trifunctional enzyme subunit alpha, mitochondrial	HADHA	83 kDa	1	1	1	1	1.3	1.1	1.1	1	0	0
P46531	Neurogenic locus notch homolog protein 1	NOTCH1	272 kDa	1	1	1.2	1.1	1.1	1.1	1.1	1	0	1
P48059	Cluster of Isoform 5 of LIM and senescent cell antigen-like-	LIMS1	42 kDa	1	1	1.1	1.1	1.1	1.1	1.1	1	0	0
P54920	Alpha-soluble NSF attachment protein	NAPA	33 kDa	1	1	1.1	1.1	1.1	1.1	1.1	1	0	0
P98175	RNA-binding protein 10	RBM10	104 kDa	1	1	1	1	1.1	1.1	1.1	1	0	0
Q14691	DNA replication complex GINS protein PSF1	GINS1	23 kDa	1	1	1	1	1.1	1.1	1.1	1	0	0
Q15042	Rab3 GTPase-activating protein catalytic subunit	RAB3GAP1	111 kDa	1	1	1	1	0.8	1.1	1.1	1	0	0
Q15819	Ubiquitin-conjugating enzyme E2 variant 2	UBE2V2	16 kDa	1	1	0.9	0.6	1.3	1	1.1	1	0	0
Q6WCQ1	Cluster of Isoform 2 of Myosin phosphatase Rho-interacting	MPRIP	118 kDa	1	1	1.1	1.1	1.2	1	1.1	1	0	0
Q8N335	Glycerol-3-phosphate dehydrogenase 1-like protein	GPD1L	38 kDa	1	1	1.2	1	1.2	1	1.1	1	0	0
Q92522	Histone H1x	H1FX	22 kDa	1	1	1.2	1.2	1.1	1	1.1	1	0	0
Q96C36	Cluster of Pyrroline-5-carboxylate reductase 2	PYCR2	34 kDa	1	1	1	1	1.1	1	1.1	1	0	0
Q9BYC5	Cluster of Alpha-(1,6)-fucosyltransferase	FUT8	67 kDa	1	1	0.7	0.8	1.1	1	1.1	1	0	1
Q9UBV4	Protein Wnt-16	WNT16	41 kDa	1	1	1.1	1.7	1	1	1.1	1	1	0
Q9UEW8	Cluster of STE20/SPS1-related proline-alanine-rich protein	STK39	59 kDa	1	1	1.2	1.1	1	1	1.1	1	0	0
Q9UKN8	General transcription factor 3C polypeptide 4	GTF3C4	92 kDa	1	1	1	1	1	1	1.1	1	0	0
Q9UKS6	Protein kinase C and casein kinase substrate in neurons prot	PACSIN3	48 kDa	1	1	1	1	0.9	1	1.1	1	0	0
Q9Y696	Chloride intracellular channel protein 4	CLIC4	29 kDa	1	1	0.8	0.9	0.9	1	1.1	1	0	0
A6NDG6	Phosphoglycolate phosphatase	PGP	34 kDa	1	1	1.1	0.8	0.9	1	1.1	1	1	0
Q02487	Desmocollin-2	DSC2	100 kDa	1	1	1.3	0.9	0.7	1	1.1	1	0	1
P46776	Cluster of 60S ribosomal protein L27a	RPL27A	17 kDa	1	1	1	0.9	1.1	0.9	1.1	1	0	0
P62244	Cluster of 40S ribosomal protein S15a	RPS15A	15 kDa	1	1	1	1	1	0.9	1.1	1	0	1
C9JV86	CD63 antigen	CD63	25 kDa	1	1	0.9	0.9	1	0.9	1.1	1	0	1
P47914	60S ribosomal protein L29	RPL29	18 kDa	1	1	0.8	0.8	1	0.9	1.1	1	0	0
Q2KHT3	Isoform 2 of Protein CLEC16A	CLEC16A	104 kDa	1	1	1	1.3	0.9	0.9	1.1	1	1	0
B4DT35	Cluster of Nucleoporin p54	NUP54	51 kDa	1	1	1.2	1	0.9	0.9	1.1	1	0	0
B4E241	Serine/arginine-rich-splicing factor 3	SRSF3	14 kDa	1	1	1.2	0.9	0.9	0.9	1.1	1	0	0
B7ZKM8	Protein transport protein Sec24B	SEC24B	140 kDa	1	1	0.9	0.9	0.9	0.9	1.1	1	0	0
C9J0J7	Cluster of Profilin-2	PFN2	10 kDa	1	1	0.9	0.8	0.9	0.9	1.1	1	0	1
C9JPG0	Cluster of Secernin-1	SCRN1	49 kDa	1	1	0.9	0.8	1	0.8	1.1	1	0	0

D3YTA9	Cluster of Calcineurin subunit B type 1	PPP3R1	21 kDa	1	1	0.8	0.9	0.8	0.8	1.1	1	0	0
E9PGM4	1,4-alpha-glucan-branching enzyme	GBE1	76 kDa	1	1	0.9	0.8	0.8	0.8	1.1	1	0	0
F5H2A4	High mobility group protein HMGI-C	HMGA2	13 kDa	1	1	0.9	0.8	0.8	0.8	1.1	1	0	0
F5H6B8	Splicing factor, suppressor of white-apricot homolog	SFSWAP	110 kDa	1	1	0.9	0.8	0.8	0.8	1.1	1	0	0
H0Y3M3	Cluster of Echinoderm microtubule-associated protein-like :	EML3	98 kDa	1	1	0.8	0.8	0.8	0.8	1.1	1	0	0
H0YMA9	Cluster of Protein CASP	CUX1	78 kDa	No Va	No Va	No Value	No Value	No Value	No Value	No Val	No Val	0	1
O15160	Cluster of DNA-directed RNA polymerases I and III subuni	POLR1C	39 kDa	1	1	0.8	0.7	0.8	0.8	1.1	1	0	0
O15264	Cluster of Mitogen-activated protein kinase 13	MAPK13	42 kDa	1	1	1.2	0.9	0.7	0.8	1.1	1	0	0
O75179	Cluster of Ankyrin repeat domain-containing protein 17	ANKRD17	274 kDa	No Va	1	No Value	0.9	No Value	1	No Val	0.5	0	0
O94766	Cluster of Galactosylgalactosylxylosylprotein 3-beta-glucur	B3GAT3	37 kDa	No Va	No Va	No Value	No Value	No Value	No Value	No Val	No Val	1	0
P04844	Cluster of Isoform 2 of Dolichyl-diphosphooligosaccharide-	RPN2	68 kDa	1	1	0.8	0.9	0.9	0.7	1.1	1	1	1
P08582	Cluster of Melanotransferrin	MFI2	80 kDa	1	1	0.8	0.7	0.6	0.7	1.1	1	1	0
P30044	Peroxiredoxin-5, mitochondrial	PRDX5	22 kDa	1	1	1	1.2	1.1	1.2	1.7	1.9	0	0
P46736	Isoform 1 of Lys-63-specific deubiquitinase BRCC36	BRCC3	33 kDa	1	1	0.7	0.6	0.7	0.6	1.1	1	0	0
P48200	Iron-responsive element-binding protein 2	IREB2	105 kDa	1	1	0.5	0.5	0.5	0.5	1.1	1	0	0
P62834	Cluster of Ras-related protein Rap-1A	RAP1A	21 kDa	1	1	0.4	0.4	0.4	0.4	1.1	1	0	0
Q07960	Rho GTPase-activating protein 1	ARHGAP1	50 kDa	1	1	0.3	0.3	0.3	0.3	1.1	1	0	1
Q13257	Mitotic spindle assembly checkpoint protein MAD2A	MAD2L1	24 kDa	1	1	1.2	1.1	2.1	2.2	1	1	0	0
Q5JVF3	Isoform 4 of PCI domain-containing protein 2	PCID2	52 kDa	1	1	1.4	1.5	1.7	1.7	1	1	0	0
Q6P2E9	Enhancer of mRNA-decapping protein 4	EDC4	152 kDa	1	1	1.3	1.6	1.6	1.5	1	1	0	0
Q8IWV7	Cluster of E3 ubiquitin-protein ligase UBR1	UBR1	200 kDa	1	1	1.7	1.8	1.5	1.4	1	1	0	0
Q969Q0	Cluster of 60S ribosomal protein L36a-like	RPL36AL	12 kDa	1	1	1.6	1.6	1.4	1.4	1	1	0	0
Q99816	Tumor susceptibility gene 101 protein	TSG101	44 kDa	1	1	1.3	1.2	1.2	1.4	1	1	0	0
Q9H9B4	Cluster of Sideroflexin-1	SFXN1	36 kDa	1	1	1.6	1.5	1.5	1.3	1	1	0	1
Q9HD42	Charged multivesicular body protein 1a	CHMP1A	22 kDa	1	1	1.2	1.1	1.4	1.3	1	1	0	0
Q9NPF4	Probable tRNA threonylcarbamoyladenosine biosynthesis p	OSGEP	36 kDa	1	1	1.8	1.9	1.3	1.3	1	1	0	0
Q9NS86	LanC-like protein 2	LANCL2	51 kDa	1	1	1.4	1.5	0.9	1.3	1	1	0	0
Q9NZ08	Endoplasmic reticulum aminopeptidase 1	ERAP1	107 kDa	1	1	1.4	1.4	1.2	1.2	1	1	1	0
Q9P273	Teneurin-3	TENM3	301 kDa	1	1	1.2	1.3	1.2	1.2	1	1	0	1
Q9UBF2	Coatomer subunit gamma-2	COPG2	98 kDa	1	1	1.2	1.2	1.2	1.2	1	1	0	0
Q9UDY2	Isoform C1 of Tight junction protein ZO-2	TJP2	131 kDa	1	1	1.1	1.2	1.2	1.2	1	1	0	0
Q8WUA8	Tsukushin	TSKU	38 kDa	1	1	1	1	1.1	1.2	1	1	1	0
O75607	Nucleoplasmin-3	NPM3	19 kDa	1	1	1.2	1.2	1	1.2	1	1	0	0
P35268	60S ribosomal protein L22	RPL22	15 kDa	1	1	0.9	0.9	0.9	1.2	1	1	0	0
Q16394	Cluster of Exostosin-1	EXT1	86 kDa	1	1	0.9	0.8	0.8	1.2	1	1	1	1
E7ETH0	Complement factor I light chain	CFI	67 kDa	1	1	1.4	1.3	1.5	1.1	1	1	1	0
O14646	Cluster of Chromodomain-helicase-DNA-binding protein 1	CHD1	197 kDa	1	1	1.1	1.2	1.3	1.1	1	1	0	0

H0YED9	Wilms tumor protein (Fragment)	WT1	20 kDa	1	1	1.4	1.3	1.2	1.1	1	1	0	0
A8MUW5	Protein FAM98B	FAM98B	46 kDa	1	1	1	1	1.2	1.1	1	1	0	1
B4DKT0	Golgi reassembly-stacking protein 2	GORASP2	49 kDa	1	1	0.8	0.8	1.2	1.1	1	1	0	0
F5H012	E3 ubiquitin-protein ligase TRIM21	TRIM21	54 kDa	1	1	1.5	1.5	1.1	1.1	1	1	0	0
G5E975	SWI/SNF related, matrix associated, actin dependent regula	SMARCB1	45 kDa	1	1	1.2	1.2	1.1	1.1	1	1	0	0
J3KQG9	UPF0696 protein C11orf68	C11orf68	32 kDa	1	1	1.2	1.2	1.1	1.1	1	1	0	0
O00273	DNA fragmentation factor subunit alpha	DFFA	37 kDa	1	1	1	1.1	1.1	1.1	1	1	0	0
O43395	Cluster of U4/U6 small nuclear ribonucleoprotein Prp3	PRPF3	78 kDa	1	1	1	0.9	1.1	1.1	1	1	0	0
O43681	ATPase ASNA1	ASNA1	39 kDa	1	1	1.1	0.9	1	1.1	1	1	0	0
P00533	Cluster of Epidermal growth factor receptor	EGFR	134 kDa	1	1	1	1	0.9	1.1	1	1	1	1
P05121	Cluster of Plasminogen activator inhibitor 1	SERPINE1	45 kDa	1	1	1.1	1.1	1.3	1.4	1.3	1.3	1	0
P09543	2',3'-cyclic-nucleotide 3'-phosphodiesterase	CNP	48 kDa	1	1	1.3	1.4	1.2	1	1	1	0	0
P09958	Cluster of Furin	FURIN	87 kDa	0.9	0.9	1.1	1.1	1.1	1	1	1	1	1
P13073	Cytochrome c oxidase subunit 4 isoform 1, mitochondrial	COX4I1	20 kDa	1	1	1	1.1	1.1	1	1	1	0	1
P36543	Cluster of Isoform 2 of V-type proton ATPase subunit E 1	ATP6V1E1	24 kDa	1	1	1.1	1	1.1	1	1	1	0	0
P41567	Eukaryotic translation initiation factor 1	EIF1	13 kDa	1	1	1.2	1.2	1	1	1	1	0	0
P46976	Cluster of Glycogenin-1	GYG1	39 kDa	1	1	1.1	1.2	1	1	1	1	0	0
P49006	MARCKS-related protein	MARCKSL1	20 kDa	1	1	1.1	1.1	1	1	1	1	0	0
P52948	Cluster of Nuclear pore complex protein Nup98-Nup96	NUP98	198 kDa	No Va	No Va	No Value	No Value	No Valu	No Value	No Val	No Val	0	0
P53004	Biliverdin reductase A	BLVRA	33 kDa	1	1	1.1	1.1	1	1	1	1	0	0
Q12860	Contactin-1	CNTN1	113 kDa	1	1	1.1	1.1	1	1	1	1	1	0
Q5MNZ6	Cluster of WD repeat domain phosphoinositide-interacting	WDR45B	38 kDa	1	1	1.2	1	1	1	1	1	0	0
Q5VZK9	Leucine-rich repeat-containing protein 16A	LRRC16A	152 kDa	1	1	1.1	1	1	1	1	1	0	0
Q7Z2Z2	Elongation factor Tu GTP-binding domain-containing prote	EFTUD1	125 kDa	1	1	1	1	1	1	1	1	0	0
Q8IUF8	Cluster of Isoform 4 of MYC-induced nuclear antigen	MINA	53 kDa	1	1	0.9	0.9	1	1	1	1	0	0
Q8NFW8	N-acylneuraminate cytidylyltransferase	CMAS	48 kDa	1	1	0.8	0.8	1	1	1	1	0	0
Q8WUM0	Nuclear pore complex protein Nup133	NUP133	129 kDa	1	1	1	1.2	0.9	1	1	1	0	0
Q92541	Cluster of RNA polymerase-associated protein RTF1 homo	RTF1	80 kDa	1	1	1.1	1.1	0.9	1	1	1	0	0
Q9BT09	Protein canopy homolog 3	CNPY3	31 kDa	1	1	1	1.1	0.9	1	1	1	1	0
Q9H2D6	Cluster of TRIO and F-actin-binding protein	TRIOBP	261 kDa	No Va	No Va	No Value	No Value	No Valu	No Value	No Val	No Val	0	0
Q9NUQ3	Gamma-taxilin	TXLNG	61 kDa	1	1	1	1.1	0.9	1	1	1	0	0
Q9NVD7	Cluster of Alpha-parvin	PARVA	42 kDa	1	1	0.9	1	0.9	1	1	1	0	1
Q9Y3A3	MOB-like protein phocein	MOB4	26 kDa	1	1	1.4	1	1.5	0.9	1	1	0	0
Q9Y3C6	Peptidyl-prolyl cis-trans isomerase-like 1	PPIL1	18 kDa	1	1	1.1	1.1	1.1	0.9	1	1	0	0
Q9Y446	Plakophilin-3	PKP3	87 kDa	1	1	0.9	1	1.1	0.9	1	1	0	0
O43633	Charged multivesicular body protein 2a	CHMP2A	25 kDa	1	1	1	0.8	1.1	0.9	1	1	0	0
Q01658	Protein Dr1	DR1	19 kDa	1	1	1.3	1.3	1	0.9	1	1	0	0

Q9NY12	H/ACA ribonucleoprotein complex subunit 1	GAR1	22 kDa	1	1	1	1.1	1	0.9	1	1	0	0
B1B0M1	Cluster of GRIP1-associated protein 1	GRIPAP1	91 kDa	1	1	1.1	1	1	0.9	1	1	0	0
B4DMQ3	Cluster of Nuclear pore complex protein Nup85	NUP85	70 kDa	1	1	1	0.9	1	0.9	1	1	1	0
D3YTB1	60S ribosomal protein L32 (Fragment)	RPL32	16 kDa	1	1	0.9	0.9	1	0.9	1	1	0	0
D6RAR4	Hepatocyte growth factor activator short chain	HGFAC	71 kDa	1	1	0.9	0.9	1	0.9	1	1	1	0
E9PRZ1	Cluster of Protein SAAL1	SAAL1	54 kDa	1	1	0.8	0.9	1	0.9	1	1	0	0
F6T1Q0	2',5'-phosphodiesterase 12	PDE12	52 kDa	1	1	1	0.8	1	0.9	1	1	0	0
H0YLI6	Cluster of Isocitrate dehydrogenase [NAD] subunit alpha, n	IDH3A	15 kDa	1	1	1.2	1.1	0.9	0.9	1	1	0	0
H3BSW6	Cluster of Cytoplasmic tRNA 2-thiolation protein 2	CTU2	64 kDa	1	1	1.1	1.1	0.9	0.9	1	1	0	0
H7BYF2	Nucleoporin p58/p45 (Fragment)	NUPL1	53 kDa	1	1	1	1.1	0.9	0.9	1	1	0	0
J3KPP4	Cisplatin resistance-associated overexpressed protein, isofo	LUC7L3	58 kDa	1	1	1	1	0.9	0.9	1	1	0	0
O00159	Cluster of Isoform 3 of Unconventional myosin-Ic	MYO1C	120 kDa	1	1	1	1	0.9	0.9	1	1	0	0
O00622	Protein CYR61	CYR61	42 kDa	1	1	1	1	0.9	0.9	1	1	3	0
O14802	DNA-directed RNA polymerase III subunit RPC1	POLR3A	156 kDa	1	1	0.9	1	0.9	0.9	1	1	0	0
O14908	PDZ domain-containing protein GIPC1	GIPC1	36 kDa	1	1	0.9	1	0.9	0.9	1	1	0	0
O43815	Striatin	STRN	86 kDa	1	1	0.8	1	0.9	0.9	1	1	0	0
O95292	Vesicle-associated membrane protein-associated protein B/(	VAPB	27 kDa	1	1	0.8	1	0.9	0.9	1	1	0	1
P11166	Solute carrier family 2, facilitated glucose transporter meml	SLC2A1	54 kDa	1	1	0.9	0.9	0.9	0.9	1	1	0	1
P11172	Cluster of Uridine 5'-monophosphate synthase	UMPS	52 kDa	1	1	0.9	0.9	0.9	0.9	1	1	0	0
P17858	Cluster of 6-phosphofructokinase, liver type	PFKL	85 kDa	1	1	0.9	0.9	0.9	0.9	1	1	0	0
P19174	1-phosphatidylinositol 4,5-bisphosphate phosphodiesterase	PLCG1	149 kDa	1	1	0.9	0.9	0.9	0.9	1	1	0	0
P31942	Heterogeneous nuclear ribonucleoprotein H3	HNRNPH3	37 kDa	1	1	0.8	0.9	0.9	0.9	1	1	0	0
P40818	Cluster of Ubiquitin carboxyl-terminal hydrolase 8	USP8	128 kDa	1	1	0.6	0.9	0.9	0.9	1	1	0	0
P43121	Cluster of Cell surface glycoprotein MUC18	MCAM	72 kDa	1	1	0.9	0.8	0.9	0.9	1	1	0	1
P46459	Cluster of Vesicle-fusing ATPase	NSF	83 kDa	1	1	0.9	0.8	0.9	0.9	1	1	0	0
P53609	Geranylgeranyl transferase type-1 subunit beta	PGGT1B	42 kDa	1	1	0.8	0.8	0.9	0.9	1	1	0	0
Q15257	Cluster of Serine/threonine-protein phosphatase 2A activatc	PPP2R4	41 kDa	1	1	0.8	0.8	0.9	0.9	1	1	0	0
Q16763	Ubiquitin-conjugating enzyme E2 S	UBE2S	24 kDa	1	1	0.8	0.8	0.9	0.9	1	1	0	0
Q5RKV6	Exosome complex component MTR3	EXOSC6	28 kDa	1	1	0.8	0.8	0.9	0.9	1	1	0	0
Q6NVY1	Cluster of 3-hydroxyisobutyryl-CoA hydrolase, mitochondr	HIBCH	43 kDa	1	1	0.7	0.7	0.9	0.9	1	1	0	0
Q7Z478	ATP-dependent RNA helicase DHX29	DHX29	155 kDa	1	1	0.6	0.7	0.9	0.9	1	1	1	0
Q86VN1	Vacuolar protein-sorting-associated protein 36	VPS36	44 kDa	1	1	1	1	0.8	0.9	1	1	0	0
Q8N9N7	Leucine-rich repeat-containing protein 57	LRRC57	27 kDa	1	1	1	1	0.8	0.9	1	1	0	0
Q8WWY3	Cluster of U4/U6 small nuclear ribonucleoprotein Prp31	PRPF31	55 kDa	1	1	0.9	0.8	0.8	0.9	1	1	0	0
Q96ST3	Paired amphipathic helix protein Sin3a	SIN3A	145 kDa	1	1	0.8	0.8	0.8	0.9	1	1	0	0
Q9C0C4	Semaphorin-4C	SEMA4C	93 kDa	1	1	0.8	0.8	0.8	0.9	1	1	1	1
Q9C0H2	Protein tweety homolog 3	TTYH3	58 kDa	1	1	0.7	0.7	0.8	0.9	1	1	0	1

Q9GZS1	Isoform 2 of DNA-directed RNA polymerase I subunit RPA	POLR1E	47 kDa	1	1	0.7	0.6	0.8	0.9	1	1	0	1
Q9H1B5	Xylosyltransferase 2	XYLT2	97 kDa	1	1	0.9	1	0.7	0.9	1	1	1	0
Q9H773	Cluster of dCTP pyrophosphatase 1	DCTPP1	19 kDa	1	1	0.9	0.9	1	0.8	1	1	0	0
Q9P016	Thymocyte nuclear protein 1	THYN1	26 kDa	1	1	0.9	0.8	1	0.8	1	1	0	0
Q9P0M6	Core histone macro-H2A.2	H2AFY2	40 kDa	1	1	1	1	0.9	0.8	1	1	0	0
Q9UBB6	Cluster of Isoform 3 of Neurochondrin	NCDN	79 kDa	1	1	0.9	1	0.9	0.8	1	1	0	0
O95749	Cluster of Geranylgeranyl pyrophosphate synthase	GGPS1	35 kDa	1	1	0.8	0.8	0.9	0.8	1	1	0	0
P24539	ATP synthase subunit b, mitochondrial	ATP5F1	29 kDa	1	1	0.8	0.8	0.9	0.8	1	1	1	1
Q14966	Isoform 3 of Zinc finger protein 638	ZNF638	218 kDa	1	1	1	1.1	0.8	0.8	1	1	0	0
Q9UMF0	Intercellular adhesion molecule 5	ICAM5	97 kDa	1	1	1	1	0.8	0.8	1	1	0	1
H7C2W9	60S ribosomal protein L31 (Fragment)	RPL31	13 kDa	1	1	0.9	1	0.8	0.8	1	1	0	0
O00566	U3 small nucleolar ribonucleoprotein protein MPP10	MPHOSPH10	79 kDa	1	1	0.9	0.9	0.8	0.8	1	1	0	1
Q04323	Isoform 2 of UBX domain-containing protein 1	UBXN1	35 kDa	1	1	0.9	0.8	0.8	0.8	1	1	0	0
P48507	Glutamate--cysteine ligase regulatory subunit	GCLM	31 kDa	1	1	0.8	0.8	0.8	0.8	1	1	0	0
Q9H3K6	Cluster of BolA-like protein 2	BOLA2	10 kDa	1	1	0.8	0.8	0.8	0.8	1	1	0	0
Q5TDH0	Cluster of Protein DDI1 homolog 2	DDI2	45 kDa	1	1	0.8	0.8	0.8	0.8	1	1	0	0
Q99805	Transmembrane 9 superfamily member 2	TM9SF2	76 kDa	1	1	0.7	0.7	0.8	0.8	1	1	0	1
B4DE64	Cluster of Programmed cell death protein 5	PDCD5	15 kDa	1	1	0.8	1	0.7	0.8	1	1	0	0
P38117	Cluster of Electron transfer flavoprotein subunit beta	ETFB	28 kDa	1	1	1.1	0.9	0.7	0.8	1	1	0	0
Q13042	Cell division cycle protein 16 homolog	CDC16	72 kDa	1	1	1	1	0.8	0.7	1	1	0	0
B5MD17	Chromobox protein homolog 1 (Fragment)	CBX1	19 kDa	1	1	0.8	0.8	0.8	0.7	1	1	0	0
G3V117	Enhancer of yellow 2 homolog (Drosophila), isoform CRA_	ENY2	11 kDa	1	1	0.9	0.6	0.8	0.7	1	1	0	0
B4DP11	Prostaglandin E synthase 3	PTGES3	16 kDa	1	1	0.7	0.8	0.7	0.7	1	1	0	0
B4E2W0	Trifunctional enzyme subunit beta, mitochondrial	HADHB	49 kDa	1	1	0.7	0.7	0.7	0.7	1	1	1	0
E9PCD7	Epididymis-specific alpha-mannosidase	MAN2B2	108 kDa	1	1	0.6	0.7	0.7	0.7	1	1	1	0
E9PK91	Bcl-2-associated transcription factor 1	BCLAF1	100 kDa	1	1	0.8	0.8	0.7	0.6	1	1	0	0
F5H2U2	Cluster of Serine/threonine-protein kinase PRP4 homolog	PRPF4B	115 kDa	1	1	0.5	0.5	0.5	0.5	1	1	0	0
G3V0F8	Cluster of Protein tyrosine phosphatase, non-receptor type 6	PTPN6	70 kDa	1	1	1.4	2	1.8	2.1	0.9	1	0	0
H0YC27	Cluster of Regulator of microtubule dynamics protein 1 (Fra	RMDN1	13 kDa	No Va	No Va	No Value	No Value	No Value	No Value	No Val	No Val	1	0
J3KPP7	Beta-arrestin-1	ARRB1	47 kDa	1	1	1.7	2.1	1.7	2	0.9	1	0	0
O15397	Cluster of Importin-8	IPO8	120 kDa	1	1	1.4	1.4	1.4	1.7	0.9	1	0	0
O43237	Cluster of Cytoplasmic dynein 1 light intermediate chain 2	DYNC1LI2	54 kDa	1	1	1.4	1.4	1.4	1.5	0.9	1	0	0
P06280	Alpha-galactosidase A	GLA	49 kDa	1	1	1.7	1.7	1.6	1.4	0.9	1	1	0
P10646	Cluster of Tissue factor pathway inhibitor	TFPI	35 kDa	1	1	1.4	1.3	1.5	1.4	0.9	1	1	0
P40763	Signal transducer and activator of transcription 3	STAT3	88 kDa	1	1	1.5	1.5	1.4	1.3	0.9	1	0	0
P54886	Delta-1-pyrroline-5-carboxylate synthase	ALDH18A1	87 kDa	1	1	1.3	1.2	1.4	1.3	0.9	1	0	0
Q15311	RalA-binding protein 1	RALBP1	76 kDa	1	1	1.1	1.5	1.2	1.3	0.9	1	0	0

Q4G0J3	La-related protein 7	LARP7	67 kDa	1	1	1.3	1.4	1.2	1.3	0.9	1	0	0
Q7Z460	Cluster of CLIP-associating protein 1	CLASP1	169 kDa	No Va	No Va	No Value	No Value	No Value	No Value	No Val	No Val	0	0
Q8N441	Fibroblast growth factor receptor-like 1	FGFRL1	55 kDa	1	1	1	1.1	1.1	1.3	0.9	1	1	1
Q8NBZ7	Cluster of UDP-glucuronic acid decarboxylase 1	UXS1	48 kDa	1	1	0.9	1.3	0.8	1.3	0.9	1	1	0
Q8NFH4	Nucleoporin Nup37	NUP37	37 kDa	1	1	1.4	1.3	1.2	1.2	0.9	1	0	0
Q96BW5	Phosphotriesterase-related protein	PTER	39 kDa	1	1	1.1	1.1	1.1	1.2	0.9	1	0	0
Q96QC0	Serine/threonine-protein phosphatase 1 regulatory subunit 1	PPP1R10	99 kDa	1	1	1	1	1.1	1.2	0.9	1	0	0
Q96T76	Cluster of MMS19 nucleotide excision repair protein homo	MMS19	113 kDa	1	1	1	0.9	1.1	1.2	0.9	1	1	0
Q99426	Tubulin-folding cofactor B	TBCB	27 kDa	1	1	0.9	1	0.9	0.9	1	1	0	0
Q99519	Sialidase-1	NEU1	45 kDa	1	1	1.1	1.1	1.3	1.1	0.9	1	0	0
Q9BZQ8	Protein Niban	FAM129A	103 kDa	1	1	0.9	0.9	1.3	1.1	0.9	1	0	0
Q9GZP4	PITH domain-containing protein 1	PITHD1	24 kDa	1	1	1.2	1.2	1.1	1.1	0.9	1	0	0
Q9H2P0	Activity-dependent neuroprotector homeobox protein	ADNP	124 kDa	1	1	0.9	1.1	1.1	1.1	0.9	1	0	0
Q9HAU5	Regulator of nonsense transcripts 2	UPF2	148 kDa	1	1	1.3	1	1	1.1	0.9	1	0	0
Q9NQ88	Fructose-2,6-bisphosphatase TIGAR	TIGAR	30 kDa	1	1	0.8	0.9	1	1.1	0.9	1		
Q9NR12	PDZ and LIM domain protein 7	PDLIM7	50 kDa	1	1	0.9	0.8	0.8	1.1	0.9	1	0	0
Q9NR50	Translation initiation factor eIF-2B subunit gamma	EIF2B3	50 kDa	1	1	1.1	0.8	1.4	1	0.9	1	0	0
Q9NZ32	Cluster of Actin-related protein 10	ACTR10	46 kDa	1	1	1.6	1.4	1.2	1	0.9	1	0	0
Q9UL25	Ras-related protein Rab-21	RAB21	24 kDa	1	1	1	1	1.1	1	0.9	1	0	0
Q9Y5A9	YTH domain family protein 2	YTHDF2	62 kDa	1	1	0.9	1	1	1	0.9	1	0	0
O14944	Proepiregulin	EREG	19 kDa	1	1	1	0.9	1	1	0.9	1	0	1
P53634	Cluster of Dipeptidyl peptidase 1	CTSC	52 kDa	1	1	0.9	0.9	1	1	0.9	1	1	0
G3V0J0	Cluster of Fragile X mental retardation 1, isoform CRA_e	FMR1	67 kDa	1	1	0.8	0.8	1	1	0.9	1	0	0
H6UMI1	Cluster of GABARAP-a	GABARAP	12 kDa	1	1	1.4	1.4	0.9	1	0.9	1	0	0
H0YAV1	Ribonucleoside-diphosphate reductase subunit M2 B (Fragr	RRM2B	47 kDa	1	1	1.1	1.2	0.9	1	0.9	1	0	1
Q96CN7	Isochorismatase domain-containing protein 1	ISOC1	32 kDa	1	1	0.9	1.1	0.9	1	0.9	1	0	0
B4DTE8	Phospholipid scramblase 1	PLSCR1	27 kDa	1	1	0.9	1.1	0.9	1	0.9	1	1	0
Q9UNW1	Cluster of Multiple inositol polyphosphate phosphatase 1	MINPP1	55 kDa	1	1	1.1	1	0.9	1	0.9	1	1	0
A4D0Z6	Inosine-5'-monophosphate dehydrogenase C	IMPDH1	64 kDa	1	1	0.9	1	0.9	1	0.9	1	0	0
P20645	Cluster of Cation-dependent mannose-6-phosphate receptor	M6PR	31 kDa	1	1	0.7	0.8	0.9	1	0.9	1	1	1
C9JFR7	Cytochrome c (Fragment)	CYCS	11 kDa	1	1	1.4	1.2	0.8	1	0.9	1	0	0
Q99996	Cluster of Isoform 6 of A-kinase anchor protein 9	AKAP9	455 kDa	1	1	0.7	0.7	0.8	1	0.9	1	0	1
F8VVA7	Cluster of Coatomer subunit zeta-1	COPZ1	22 kDa	1	1	0.9	1.1	0.9	0.9	0.9	1	0	1
P63173	60S ribosomal protein L38	RPL38	8 kDa	1	1	1	1	0.9	0.9	0.9	1	0	0
E9PRJ3	Cluster of CD151 antigen (Fragment)	CD151	20 kDa	1	1	0.9	0.9	0.9	0.9	0.9	1	0	1
P04066	Tissue alpha-L-fucosidase	FUCA1	54 kDa	1	1	0.9	1	0.8	0.9	0.9	1	3	0
Q13485	Mothers against decapentaplegic homolog 4	SMAD4	60 kDa	1	1	0.7	0.9	0.8	0.9	0.9	1	0	0

A6NJ11	Ubiquitin fusion degradation protein 1 homolog	UFD1L	33 kDa	1	1	0.9	1	0.9	0.8	0.9	1	0	0
B7Z3P1	Tubulin-specific chaperone E	TBCE	65 kDa	1	1	1	0.9	0.9	0.8	0.9	1	0	0
C9K0K7	Cluster of Phosphoribosyl pyrophosphate synthase-associat	PRPSAP2	27 kDa	No Va	No Va	No Value	No Value	No Value	No Value	No Val	No Val	0	0
D6RFW1	Small glutamine-rich tetratricopeptide repeat-containing pro	SGTB	22 kDa	1	1	0.8	0.9	0.8	0.8	0.9	1	0	0
E7EWM9	Extracellular matrix protein FRAS1	FRAS1	79 kDa	1	1	0.7	0.8	0.8	0.8	0.9	1	1	1
F5GY99	Cluster of Polypeptide N-acetylgalactosaminyltransferase 1	GALNT1	57 kDa	1	1	1	0.9	0.9	1	0.9	0.7	0	1
F5H4Z4	Cluster of F-box/LRR-repeat protein 18	FBXL18	88 kDa	1	1	0.8	1	0.7	0.8	0.9	1	0	0
G5E928	Lysosomal alpha-mannosidase D peptide	MAN2B1	114 kDa	1	1	0.8	0.8	0.7	0.8	0.9	1	1	0
H7C5L1	Cluster of Prostaglandin E synthase 2 (Fragment)	PTGES2	34 kDa	1	1	0.9	1.2	0.9	0.7	0.9	1	0	0
J3KP43	Ribonucleoside-diphosphate reductase subunit M2	RRM2	51 kDa	1	1	0.6	0.7	0.6	0.7	0.9	1	0	1
J3QSS4	Formin-binding protein 1-like	FNBP1L	70 kDa	1	1	0.8	0.7	0.6	0.5	0.9	1	0	0
O60306	Intron-binding protein aquarius	AQR	171 kDa	1	1	0.9	1.1	1	1.6	0.8	1	0	1
O60513	Beta-1,4-galactosyltransferase 4	B4GALT4	40 kDa	1	1	1.3	0.8	1.2	1.3	0.8	1	1	0
O75223	Cluster of Gamma-glutamylcyclotransferase	GGCT	21 kDa	1	1	1.2	1.2	1.1	1.2	0.8	1	0	0
O75400	Pre-mRNA-processing factor 40 homolog A	PRPF40A	109 kDa	1	1	1.1	1.1	1.1	1.2	0.8	1	0	0
O75694	Nuclear pore complex protein Nup155	NUP155	155 kDa	1	1	1.2	0.9	1.1	1.2	0.8	1	1	0
O95202	LETM1 and EF-hand domain-containing protein 1, mitocho	LETM1	83 kDa	1	1	0.9	0.9	0.8	1.2	0.8	1	0	1
P05161	Ubiquitin-like protein ISG15	ISG15	18 kDa	1	1	1.2	1.1	1.3	1.1	0.8	1	0	0
P18031	Tyrosine-protein phosphatase non-receptor type 1	PTPN1	50 kDa	1	1	1	1.1	1	1.1	0.8	1	0	1
P28331	Isoform 2 of NADH-ubiquinone oxidoreductase 75 kDa sub	NDUFS1	81 kDa	1	1	1.2	1	1	1.1	0.8	1	0	0
P30084	Enoyl-CoA hydratase, mitochondrial	ECHS1	31 kDa	1	1	0.8	0.9	1	1	0.8	1	0	0
P43304	Glycerol-3-phosphate dehydrogenase, mitochondrial	GPD2	81 kDa	1	1	1	1.2	0.8	1	0.8	1	1	0
P46783	Cluster of 40S ribosomal protein S10	RPS10	19 kDa	1	1	0.8	0.9	0.8	1	0.8	1	0	0
P48723	Heat shock 70 kDa protein 13	HSPA13	52 kDa	1	1	0.8	0.8	0.8	1	0.8	1	1	0
P51572	B-cell receptor-associated protein 31	BCAP31	28 kDa	1	1	0.8	1	0.9	0.9	0.8	1	0	1
P57740	Cluster of Nuclear pore complex protein Nup107	NUP107	106 kDa	1	1	0.7	0.9	0.9	0.9	0.8	1	0	1
P62166	Neuronal calcium sensor 1	NCS1	22 kDa	1	1	0.9	1	0.7	0.9	0.8	1	0	0
Q13275	Cluster of Semaphorin-3F	SEMA3F	88 kDa	1	1	0.8	0.8	0.7	0.9	0.8	1	1	0
Q13425	Cluster of Beta-2-syntrophin	SNTB2	58 kDa	1	1	0.8	0.8	0.7	0.9	0.8	1	1	0
Q13610	Periodic tryptophan protein 1 homolog	PWP1	56 kDa	1	1	0.8	0.8	0.6	0.9	0.8	1	0	0
Q14137	Ribosome biogenesis protein BOP1	BOP1	84 kDa	1	1	0.9	0.7	0.6	0.9	0.8	1	0	0
Q16512	Cluster of Serine/threonine-protein kinase N1	PKN1	104 kDa	1	1	0.7	0.8	0.8	0.8	0.8	1	0	0
Q32P28	Cluster of Prolyl 3-hydroxylase 1	LEPRE1	83 kDa	1	1	0.8	0.8	0.7	0.8	0.8	1	1	0
Q504X1	ARHGAP12 protein	ARHGAP12	96 kDa	1	1	0.4	0.5	0.7	0.8	0.8	1	0	0
Q5T2E6	UPF0668 protein C10orf76	C10orf76	79 kDa	1	1	0.6	0.7	0.6	0.8	0.8	1	0	0
Q5VTL8	Pre-mRNA-splicing factor 38B	PRPF38B	64 kDa	1	1	0.5	0.5	0.6	0.7	0.8	1	0	0
Q6ZRP7	Cluster of Sulfhydryl oxidase 2	QSOX2	78 kDa	1	1	0.9	1.1	1	1.4	0.7	1	0	1

Q86SF2	N-acetylgalactosaminyltransferase 7	GALNT7	75 kDa	1	1	0.9	1.1	0.9	1.2	0.7	1	0	0
Q8IXH7	Negative elongation factor C/D	NELFCD	66 kDa	1	1	1.4	1.5	1.1	1.1	0.7	1	0	0
Q8TCS8	Cluster of Polyribonucleotide nucleotidyltransferase 1, mitc	PNPT1	86 kDa	1	1	0.9	1	0.9	1.1	0.7	1	0	0
Q8WU90	Zinc finger CCCH domain-containing protein 15	ZC3H15	49 kDa	1	1	0.9	0.9	0.9	1	0.7	1	0	0
Q92673	Sortilin-related receptor	SORL1	248 kDa	1	1	0.6	0.8	0.9	1	0.7	1	0	1
Q92783	Cluster of Signal transducing adapter molecule 1	STAM	59 kDa	1	1	1	0.9	1	0.9	0.7	1	0	0
Q96H20	Cluster of Vacuolar-sorting protein SNF8	SNF8	29 kDa	1	1	1	0.9	0.8	0.8	0.7	1	0	0
Q96P11	Cluster of Isoform 2 of Putative methyltransferase NSUN5	NSUN5	50 kDa	1	1	0.8	0.9	0.8	0.8	0.7	1	0	0
Q96Q11	CCA tRNA nucleotidyltransferase 1, mitochondrial	TRNT1	50 kDa	1	1	1.3	1.3	0.7	0.8	0.7	1	0	0
Q96SB4	Cluster of Isoform 1 of SRSF protein kinase 1	SRPK1	92 kDa	1	1	0.7	0.7	0.7	0.7	0.7	1	0	0
Q9HD45	Cluster of Transmembrane 9 superfamily member 3	TM9SF3	68 kDa	1	1	1	1.4	0.9	1.3	0.6	1	0	1
Q9NW13	Cluster of RNA-binding protein 28	RBM28	86 kDa	1	1	1.5	1.4	1.2	1.2	0.6	1	0	0
Q9UHR4	Brain-specific angiogenesis inhibitor 1-associated protein 2-	BAIAP2L1	57 kDa	1	1	0.9	1	1	1.2	0.6	1	0	0
B4DE16	Beta-catenin-like protein 1	CTNNBL1	62 kDa	1	1	1.4	1	1.3	1.1	1.7	2.2	0	0
Q969H8	UPF0556 protein C19orf10	C19orf10	19 kDa	1	1	0.8	0.8	0.8	0.9	0.4	0.6	1	0
J3QL51	Protein RPL17-C18ORF32	RPL17-C18OI	26 kDa	1	1	1.2	1.6	0.8	0.9	1.2	1.3	0	0
P62487	DNA-directed RNA polymerase II subunit RPB7	POLR2G	19 kDa	1	1	0.7	0.8	0.8	0.9	2	1.6	0	0
P34059	Cluster of N-acetylgalactosamine-6-sulfatase	GALNS	58 kDa	1	1	1.1	1	1.1	1	1.6	1.7	1	0
Q8WUD1	Cluster of Ras-related protein Rab-2B	RAB2B	24 kDa	No Va	1	No Value	1	No Value	0.8	No Val	1	0	0
Q9NVC6	Cluster of Mediator of RNA polymerase II transcription sub	MED17	73 kDa	1	1	1	1	1	1	1.1	1	0	0
P39656	Dolichyl-diphosphooligosaccharide--protein glycosyltransfe	DDOST	51 kDa	1	1	0.6	0.6	0.9	0.8	0.4	0.4	1	1
Q13952	Cluster of Nuclear transcription factor Y subunit gamma	NFYC	50 kDa	1	1	1	1.1	1.1	1.3	1.8	1.1	0	0
Q15198	Platelet-derived growth factor receptor-like protein	PDGFRL	42 kDa	1	1	1.3	1	1	0.9	1.6	1.1	1	0
Q06033	Inter-alpha-trypsin inhibitor heavy chain H3	ITIH3	100 kDa	1	1	1	1	1.2	1.1	1.5	1.1	1	0
P07305	Histone H1.0	H1F0	21 kDa	1	1	1.6	1.7	1.4	1.3	0.5	0.5	0	0
Q15828	Cystatin-M	CST6	17 kDa	1	1	0.9	0.8	1.1	1	1.5	1.1	3	0
A8MW61	Cluster of Pleiotropic regulator 1	PLRG1	57 kDa	1	1	0.9	0.9	1	1	1.5	1.1	0	1
E7EN22	Ubiquitin carboxyl-terminal hydrolase	USP19	151 kDa	1	1	0.7	0.7	0.9	0.8	1.5	1.1	0	1
E9PE68	Oxysterol-binding protein	OSBPL8	97 kDa	1	1	1.2	0.9	1.3	1	1.4	1.1	0	1
E9PGR1	Heparanase 8 kDa subunit	HPSE	55 kDa	1	1	0.9	0.9	1	1	1.4	1.1	1	0
F5GZ27	Cluster of Lon protease homolog	LONP1	86 kDa	1	1	1.2	1	0.8	0.9	1.4	1.1	0	0
F5H3L5	Protocadherin-1	PCDH1	113 kDa	1	1	0.9	0.9	0.7	0.9	1.4	1.1	1	1
F5H6P0	Protein-glutamine gamma-glutamyltransferase 2	TGM2	70 kDa	1	1	1	1	1.1	0.8	1.4	1.1	0	0
H0YFA7	Cluster of N-acetylmannosamine kinase (Fragment)	GNE	76 kDa	No Va	No Va	No Value	No Value	No Value	No Value	No Val	No Val	0	1
J3KNP2	Cluster of Transducin beta-like protein 3 (Fragment)	TBL3	77 kDa	1	1	0.9	0.9	0.9	0.8	1.4	1.1	0	0
O00264	Cluster of Membrane-associated progesterone receptor com	PGRMC1	22 kDa	1	1	0.9	1	0.8	0.8	1.4	1.1	0	1
O15294	UDP-N-acetylglucosamine--peptide N-acetylglucosaminyltı	OGT	117 kDa	1	1	1.2	1.2	1.5	1.5	1.3	1.1	0	0

O75396	Vesicle-trafficking protein SEC22b	SEC22B	25 kDa	1	1	1.1	1.1	1.2	1.5	1.3	1.1		
O94888	UBX domain-containing protein 7	UBXN7	55 kDa	1	1	1	1.4	1.7	1.1	1.3	1.1	0	0
O95671	N-acetylserotonin O-methyltransferase-like protein	ASMTL	69 kDa	1	1	1.1	0.8	1.4	1.1	1.3	1.1	0	0
P07203	Glutathione peroxidase 1	GPX1	22 kDa	1	1	1.2	1.1	1.2	1.1	1.3	1.1	0	0
P49137	Cluster of MAP kinase-activated protein kinase 2	MAPKAPK2	46 kDa	1	1	1.1	1.2	1.1	1.1	1.3	1.1	0	1
P49750	Isoform 4 of YLP motif-containing protein 1	YLPM1	242 kDa	1	1	1.2	1.2	1.2	1	1.3	1.1	0	0
Q14320	Cluster of Protein FAM50A	FAM50A	40 kDa	1	1	1.2	1.1	1.2	1	1.3	1.1	0	0
Q3MHD2	Protein LSM12 homolog	LSM12	22 kDa	1	1	1.1	1	1.2	1	1.3	1.1	0	0
Q68EM7	Isoform 5 of Rho GTPase-activating protein 17	ARHGAP17	94 kDa	1	1	1.2	0.9	1.1	1	1.3	1.1	0	0
Q8IV36	Cluster of Protein hid-1 homolog	HID1	89 kDa	1	1	1.1	1.1	1	1	1.3	1.1	0	0
Q8IWV8	Cluster of E3 ubiquitin-protein ligase UBR2	UBR2	201 kDa	1	1	1.1	1.1	0.9	0.9	1.3	1.1	0	1
Q8IX12	Cluster of Cell division cycle and apoptosis regulator protei	CCAR1	133 kDa	1	1	1	1	0.9	0.9	1.3	1.1	0	0
Q8N8S7	Protein enabled homolog	ENAH	67 kDa	1	1	0.9	0.9	0.9	0.9	1.3	1.1	0	0
Q8NCH0	Carbohydrate sulfotransferase 14	CHST14	43 kDa	1	1	1.2	1	1	0.8	1.3	1.1	0	1
Q8NDZ4	Deleted in autism protein 1	C3orf58	49 kDa	1	1	0.8	0.8	0.8	0.8	1.3	1.1	1	0
Q92485	Cluster of Acid sphingomyelinase-like phosphodiesterase 3l	SMPDL3B	51 kDa	1	1	0.9	0.8	0.8	0.7	1.3	1.1	1	0
Q96GD0	Pyridoxal phosphate phosphatase	PDXP	32 kDa	1	1	0.7	0.8	0.8	0.7	1.3	1.1	0	0
Q96IJ6	Mannose-1-phosphate guanyltransferase alpha	GMPPA	46 kDa	1	1	0.7	0.6	0.8	0.7	1.3	1.1	0	0
Q96LD4	Tripartite motif-containing protein 47	TRIM47	70 kDa	1	1	0.6	0.6	0.6	0.6	1.3	1.1	0	0
Q96SZ5	2-aminoethanethiol dioxygenase	ADO	30 kDa	1	1	2	1.8	1.7	1.8	1.2	1.1	0	0
Q9NVP1	Cluster of ATP-dependent RNA helicase DDX18	DDX18	75 kDa	1	1	1.6	1.6	1.6	1.6	1.2	1.1	0	0
Q9NW82	WD repeat-containing protein 70	WDR70	73 kDa	1	1	2.3	2.2	1.8	1.5	1.2	1.1	0	0
Q9UIQ6	Leucyl-cystinyl aminopeptidase	LNPEP	117 kDa	1	1	1.8	1.3	1.8	1.4	1.2	1.1	0	1
Q9UKG1	DCC-interacting protein 13-alpha	APPL1	80 kDa	1	1	1.5	1.4	1.3	1.4	1.2	1.1	0	0
Q9UNX4	WD repeat-containing protein 3	WDR3	106 kDa	1	1	1.1	1	1.4	1.3	1.2	1.1	0	0
Q9Y5K6	CD2-associated protein	CD2AP	71 kDa	1	1	1.4	1.3	1.3	1.3	1.2	1.1	0	0
Q9Y6D9	Cluster of Mitotic spindle assembly checkpoint protein MA	MAD1L1	83 kDa	1	1	1.4	1.4	1.2	1.3	1.2	1.1	1	0
Q9Y6W5	Wiskott-Aldrich syndrome protein family member 2	WASF2	54 kDa	1	1	1.1	1.3	1.1	1.3	1.2	1.1	0	0
E5RI99	60S ribosomal protein L30 (Fragment)	RPL30	13 kDa	1	1	1.2	1.4	1.1	1.2	1.3	1.4	0	0
Q8IV08	Phospholipase D3	PLD3	55 kDa	1	1	1.3	1.4	1.3	1.2	1.2	1.1	0	1
E9PQK6	Apoptosis inhibitor 5	API5	14 kDa	1	1	1.4	1.3	1.3	1.2	1.2	1.1	0	0
Q9Y244	Proteasome maturation protein	POMP	16 kDa	1	1	1.4	1.1	1.3	1.2	1.2	1.1	0	0
Q15847	Adipose most abundant gene transcript 2 protein	ADIRF	8 kDa	1	1	1.4	1.4	1.2	1.2	1.2	1.1	0	0
Q92692	Isoform Alpha of Poliovirus receptor-related protein 2	PVRL2	51 kDa	1	1	1.3	1.3	1.2	1.2	1.2	1.1	0	1
P06727	Apolipoprotein A-IV	APOA4	45 kDa	1	1	1	1	1.1	1.2	1.2	1.1	3	0
Q9NWX6	Probable tRNA(His) guanylyltransferase	THG1L	35 kDa	1	1	1.1	1.2	1.4	1.1	1.2	1.1	0	0
C9JEJ2	Cluster of Choline-phosphate cytidylyltransferase A	PCYT1A	43 kDa	1	1	1.2	1	1.3	1.1	1.2	1.1	0	0

Q9UK45	U6 snRNA-associated Sm-like protein LSm7	LSM7	12 kDa	1	1	1.6	1.4	1.1	1.1	1.2	1.1	0	0
Q12955	Ankyrin-3	ANK3	480 kDa	1	1	1.3	1.2	1.1	1.1	1.2	1.1	0	0
Q9BSJ8	Cluster of Extended synaptotagmin-1	ESYT1	123 kDa	1	1	0.8	0.8	1.1	1.1	1.2	1.1	0	1
P62854	Cluster of 40S ribosomal protein S26	RPS26	13 kDa	1	1	1.5	1.2	1	1.1	1.2	1.1	0	0
P05546	Heparin cofactor 2	SERPIND1	57 kDa	1	1	1.2	1.2	1	1.1	1.2	1.1	1	0
B7ZKS7	Cluster of USP48 protein	USP48	121 kDa	1	1	1.1	1.1	1	1.1	1.2	1.1	0	0
A3KFL2	Exosome complex component RRP4	EXOSC2	32 kDa	1	1	0.9	0.8	0.9	1.1	1.2	1.1	0	0
B4DUV6	Cluster of cDNA FLJ51319, highly similar to tRNA (adenir	TRMT6	36 kDa	No Va	No Va	No Value	No Value	No Value	No Value	No Val	No Val	0	0
E5RHG8	Transcription elongation factor B polypeptide 1 (Fragment)	TCEB1	10 kDa	1	1	1.2	1.1	1.1	1	1.2	1.1	0	0
E9PCJ7	Cluster of Putative E3 ubiquitin-protein ligase UBR7	UBR7	40 kDa	1	1	1.1	1	1.1	1	1.2	1.1	0	0
E9PCK7	RRP12-like protein	RRP12	137 kDa	1	1	0.9	0.9	1.1	1	1.2	1.1	0	1
E9PDL6	Protein NDRG1	NDRG1	35 kDa	1	1	0.9	0.9	1.1	1	1.2	1.1	0	0
F2Z2Y4	Pyridoxal kinase	PDXK	31 kDa	1	1	0.8	0.9	1.1	1	1.2	1.1	0	0
J3KRG1	Cluster of Tether-containing UBX domain for GLUT4 (Fra	ASPSCR1	21 kDa	No Va	No Va	No Value	No Value	No Value	No Value	No Val	No Val	0	0
J3QQJ0	Cluster of SAP30-binding protein (Fragment)	SAP30BP	36 kDa	1	1	1.2	1.1	1	1	1.2	1.1	0	0
O14841	5-oxoprolinase	OPLAH	137 kDa	1	1	1.2	1.1	1	1	1.2	1.1	0	0
O95750	Fibroblast growth factor 19	FGF19	24 kDa	1	1	0.9	1	1	1	1.2	1.1	3	0
P06746	Cluster of DNA polymerase beta	POLB	38 kDa	1	1	1.1	0.9	1	1	1.2	1.1	0	0
P13995	Cluster of Bifunctional methylenetetrahydrofolate dehydrog	MTHFD2	38 kDa	1	1	0.8	0.9	0.9	1	1.2	1.1	1	0
P15907	Cluster of Beta-galactoside alpha-2,6-sialyltransferase 1	ST6GAL1	47 kDa	1	1	0.9	1.1	1.1	0.9	1.2	1.1	1	0
P26373	60S ribosomal protein L13	RPL13	24 kDa	1	1	1.1	0.9	1	0.9	1.2	1.1	0	0
P28370	Probable global transcription activator SNF2L1	SMARCA1	123 kDa	1	1	1	0.9	1	0.9	1.2	1.1	0	0
P48729	Cluster of Casein kinase I isoform alpha	CSNK1A1	39 kDa	1	1	1.1	1.1	0.9	0.9	1.2	1.1	0	0
P83436	Conserved oligomeric Golgi complex subunit 7	COG7	86 kDa	1	1	0.8	1	0.9	0.9	1.2	1.1	0	0
Q04206	Cluster of Transcription factor p65	RELA	60 kDa	1	1	0.9	0.9	0.9	0.9	1.2	1.1	0	0
Q14676	Cluster of Mediator of DNA damage checkpoint protein 1	MDC1	227 kDa	1	1	0.9	0.9	0.9	0.9	1.2	1.1	0	0
Q15269	Cluster of Periodic tryptophan protein 2 homolog	PWP2	102 kDa	1	1	0.9	0.9	0.9	0.9	1.2	1.1	0	0
Q15417	Cluster of Calponin-3	CNN3	36 kDa	1	1	0.7	0.9	0.9	0.9	1.2	1.1	0	0
Q52LW3	Rho GTPase-activating protein 29	ARHGAP29	142 kDa	1	1	0.9	0.8	0.9	0.9	1.2	1.1	0	0
Q5JRX3	Cluster of Presequence protease, mitochondrial	PITRM1	117 kDa	No Va	No Va	No Value	No Value	No Value	No Value	No Val	No Val	0	0
Q5T2T1	MAGUK p55 subfamily member 7	MPP7	66 kDa	1	1	0.8	0.8	0.9	0.9	1.2	1.1	0	0
Q7Z7M9	Polypeptide N-acetylgalactosaminyltransferase 5	GALNT5	106 kDa	1	1	0.7	0.7	0.8	0.9	1.2	1.1	0	0
Q92542	Nicastrin	NCSTN	78 kDa	1	1	1	0.9	0.9	0.8	1.2	1.1	1	1
Q9BTC0	Cluster of Death-inducer obliterator 1	DIDO1	244 kDa	1	1	1	1.1	0.8	0.8	1.2	1.1	0	0
Q9BW91	Isoform 2 of ADP-ribose pyrophosphatase, mitochondrial	NUDT9	34 kDa	1	1	0.8	0.9	0.8	0.8	1.2	1.1	1	0
Q9H0A0	Cluster of N-acetyltransferase 10	NAT10	116 kDa	1	1	0.8	0.8	0.8	0.8	1.2	1.1	0	0
Q9H267	Vacuolar protein sorting-associated protein 33B	VPS33B	71 kDa	1	1	0.8	0.7	0.8	0.8	1.2	1.1	0	0

Q9H307	Pinin	PNN	82 kDa	1	1	0.7	0.7	0.8	0.8	1.2	1.1	0	0
Q9H497	Cluster of Torsin-3A	TOR3A	46 kDa	1	1	0.8	0.7	0.8	0.7	1.2	1.1	1	0
Q9HB07	Cluster of UPF0160 protein MYG1, mitochondrial	C12orf10	42 kDa	1	1	0.6	0.6	0.8	0.7	1.2	1.1	1	0
Q9NVT9	Armadillo repeat-containing protein 1	ARMC1	31 kDa	1	1	0.9	0.8	0.7	0.7	1.2	1.1	0	0
Q9Y520	Cluster of Isoform 3 of Protein PRRC2C	PRRC2C	296 kDa	No Va	No Va	No Value	No Value	No Value	No Value	No Val	No Val	0	0
F5H4E4	Ketosamine-3-kinase	FN3KRP	29 kDa	1	1	0.6	0.8	0.7	0.7	1.2	1.1	0	0
H7BXH8	Cluster of Heterogeneous nuclear ribonucleoprotein L-like (	HNRNPLL	24 kDa	No Va	No Va	No Value	No Value	No Value	No Value	No Val	No Val	0	0
P46109	Crk-like protein	CRKL	34 kDa	1	1	1.2	1.4	1.6	1.8	1.1	1.1	0	0
P60891	Cluster of Ribose-phosphate pyrophosphokinase 1	PRPS1	35 kDa	1	1	1.1	1.2	1.6	1.8	1.1	1.1	0	0
Q9Y5J7	Mitochondrial import inner membrane translocase subunit T	TIMM9	10 kDa	1	1	1.3	1.4	1.5	1.8	1.1	1.1	0	0
Q99543	DnaJ homolog subfamily C member 2	DNAJC2	72 kDa	1	1	1.6	1.9	1.6	1.7	1.1	1.1	0	0
E7EPK6	40S ribosomal protein S24	RPS24	32 kDa	1	1	1.7	1.7	1.5	1.5	1.1	1.1	0	0
Q96M27	Cluster of Protein PRRC1	PRRC1	47 kDa	1	1	1.3	1.2	1.4	1.4	1.1	1.1	0	1
Q9NVJ2	Cluster of ADP-ribosylation factor-like protein 8B	ARL8B	22 kDa	1	1	1.4	1.5	1.5	1.3	1.1	1.1	1	0
Q4J6C6	Cluster of Prolyl endopeptidase-like	PREPL	84 kDa	No Va	No Va	No Value	No Value	No Value	No Value	No Val	No Val	0	0
O95571	Protein ETHE1, mitochondrial	ETHE1	28 kDa	1	1	1.2	1.2	1.5	1.3	1.1	1.1	0	0
Q92854	Cluster of Semaphorin-4D	SEMA4D	96 kDa	1	1	1.4	1.5	1.3	1.3	1.1	1.1	1	1
Q00059	Cluster of Transcription factor A, mitochondrial	TFAM	29 kDa	1	1	1.2	1.2	1.3	1.3	1.1	1.1	1	0
P61970	Nuclear transport factor 2	NUTF2	14 kDa	1	1	1	1.4	1.2	1.3	1.1	1.1	0	0
P62857	40S ribosomal protein S28	RPS28	8 kDa	1	1	1.1	1.3	1.1	1.3	1.1	1.1	0	0
Q9Y2A9	UDP-GlcNAc:betaGal beta-1,3-N-acetylglucosaminyltransf	B3GNT3	43 kDa	1	1	1.3	1.4	1.3	1.2	1.1	1.1	1	0
Q96HS1	Cluster of Serine/threonine-protein phosphatase PGAM5, m	PGAM5	32 kDa	1	1	1.2	1.3	1.2	1.2	1.1	1.1	1	0
P21583	Kit ligand	KITLG	31 kDa	1	1	1.2	1.2	1.2	1.2	1.1	1.1	0	1
B4DH53	Microtubule-associated protein 1S	MAP1S	110 kDa	1	1	1	1.2	1.2	1.2	1.1	1.1	0	0
E7ES19	Cluster of Thrombospondin-4	THBS4	96 kDa	1	1	1.2	1.2	1.2	1.1	1.1	1.1	1	0
F5GYN4	Cluster of Ubiquitin thioesterase OTUB1	OTUB1	28 kDa	1	1	1.1	1.2	1.2	1.1	1.1	1.1	0	0
F8W930	Insulin-like growth factor 2 mRNA-binding protein 2	IGF2BP2	67 kDa	1	1	1.3	1.1	1.2	1.1	1.1	1.1	0	0
G3XAD6	Cluster of FIP1 like 1 (S. cerevisiae), isoform CRA_d	FIP1L1	66 kDa	No Va	No Va	No Value	No Value	No Value	No Value	No Val	No Val	0	0
H0Y612	Cluster of E3 ubiquitin-protein ligase TRIM33 (Fragment)	TRIM33	99 kDa	1	1	1.3	1.2	1.1	1.1	1.1	1.1	0	0
H0YHG0	Uncharacterized protein (Fragment)		59 kDa	1	1	1.2	1.2	1.1	1.1	1.1	1.1		
H3BPK3	Cluster of Hydroxyacylglutathione hydrolase, mitochondria	HAGH	26 kDa	No Va	No Va	No Value	No Value	No Value	No Value	No Val	No Val	0	0
H7C0V4	Reticulon-4 receptor (Fragment)	RTN4R	53 kDa	1	1	1.1	1.2	1.1	1.1	1.1	1.1	0	1
J3QKR5	Cluster of Cyclin-dependent kinase 11B	CDK11B	90 kDa	No Va	No Va	No Value	No Value	No Value	No Value	No Val	No Val	0	0
O60508	Pre-mRNA-processing factor 17	CDC40	66 kDa	1	1	1.1	1	1.1	1.1	1.1	1.1	1	0
P22681	Cluster of E3 ubiquitin-protein ligase CBL	CBL	100 kDa	1	1	1	1	1.1	1.1	1.1	1.1	0	0
P35270	Sepiapterin reductase	SPR	28 kDa	1	1	0.9	0.9	1.1	1.1	1.1	1.1	0	0
P35658	Isoform 5 of Nuclear pore complex protein Nup214	NUP214	215 kDa	1	1	1.2	1.3	1	1.1	1.1	1.1	1	0

P50452	Cluster of Serpin B8	SERPINB8	43 kDa	1	1	1.1	1.2	1	1.1	1.1	1.1	0	0
P53365	Cluster of Arfaptin-2	ARFIP2	38 kDa	1	1	1.1	1.1	1	1.1	1.1	1.1	1	0
P53611	Geranylgeranyl transferase type-2 subunit beta	RABGGTB	37 kDa	1	1	1.1	1.1	1	1.1	1.1	1.1	0	0
P53985	Monocarboxylate transporter 1	SLC16A1	54 kDa	1	1	1	1.1	1	1.1	1.1	1.1	0	1
P54802	Alpha-N-acetylglucosaminidase	NAGLU	82 kDa	1	1	0.9	1	1	1.1	1.1	1.1	1	0
P55001	Microfibrillar-associated protein 2	MFAP2	21 kDa	1	1	1	1.1	0.9	1.1	1.1	1.1	1	0
P61513	Cluster of 60S ribosomal protein L37a	RPL37A	10 kDa	1	1	1	0.9	1.2	1	1.1	1.1	0	0
P78356	Cluster of Phosphatidylinositol 5-phosphate 4-kinase type-2	PIP4K2B	47 kDa	1	1	1.3	1.3	1.1	1	1.1	1.1	0	0
Q12805	Cluster of EGF-containing fibulin-like extracellular matrix	EFEMP1	55 kDa	1	1	1.1	1.1	1.1	1	1.1	1.1	1	0
Q13630	GDP-L-fucose synthase	TSTA3	36 kDa	1	1	1.1	1	1.1	1	1.1	1.1	0	0
Q13895	Bystin	BYSL	50 kDa	1	1	1	1	1.1	1	1.1	1.1	0	0
Q7L266	Cluster of L-asparaginase	ASRGL1	32 kDa	1	1	1	1	1.1	1	1.1	1.1	0	1
Q7Z739	Cluster of YTH domain family protein 3	YTHDF3	64 kDa	1	1	0.9	1	1.1	1	1.1	1.1	0	0
Q8WWI5	Choline transporter-like protein 1	SLC44A1	73 kDa	1	1	0.8	0.9	1.1	1	1.1	1.1	0	1
Q92621	Nuclear pore complex protein Nup205	NUP205	228 kDa	1	1	1.3	1.3	1	1	1.1	1.1	0	1
Q96EU7	C1GALT1-specific chaperone 1	C1GALT1C1	36 kDa	1	1	1.1	1.2	1	1	1.1	1.1	0	0
Q96K76	Isoform 2 of Ubiquitin carboxyl-terminal hydrolase 47	USP47	147 kDa	1	1	1.1	1.1	1	1	1.1	1.1	0	0
Q96PU5	Cluster of Isoform 6 of E3 ubiquitin-protein ligase NEDD4-	NEDD4L	109 kDa	1	1	1	1.1	1	1	1.1	1.1	0	0
Q9BRT9	DNA replication complex GINS protein SLD5	GINS4	26 kDa	1	1	1	0.9	1	1	1.1	1.1	0	0
Q9H223	EH domain-containing protein 4	EHD4	61 kDa	1	1	0.9	0.9	1	1	1.1	1.1	0	0
Q9HCP0	Cluster of Casein kinase I isoform gamma-1	CSNK1G1	49 kDa	1	1	0.9	0.9	1	1	1.1	1.1	0	0
Q9NVX2	Notchless protein homolog 1	NLE1	53 kDa	1	1	0.9	0.9	1	1	1.1	1.1	0	0
Q9NWT6	Hypoxia-inducible factor 1-alpha inhibitor	HIF1AN	40 kDa	1	1	0.9	0.9	1	1	1.1	1.1	0	0
Q9P2M7	Cluster of Cingulin	CGN	136 kDa	1	1	0.7	0.8	1	1	1.1	1.1	0	0
Q9UIG0	Tyrosine-protein kinase BAZ1B	BAZ1B	171 kDa	1	1	1.1	1.2	0.9	1	1.1	1.1	0	0
E5RJA0	Acyl-protein thioesterase 1	LYPLA1	15 kDa	1	1	1.1	1.1	0.9	1	1.1	1.1	0	1
P36507	Dual specificity mitogen-activated protein kinase kinase 2	MAP2K2	44 kDa	1	1	0.9	0.9	0.9	1	1.1	1.1	0	0
Q8WW12	PEST proteolytic signal-containing nuclear protein	PCNP	19 kDa	1	1	0.9	0.9	0.9	1	1.1	1.1	0	0
F5H1H6	Cluster of Cytospin-A	SPECC1L	120 kDa	1	1	1	0.8	0.9	1	1.1	1.1	0	0
P61019	Isoform 2 of Ras-related protein Rab-2A	RAB2A	21 kDa	1	1	0.8	0.8	0.9	1	1.1	1.1	0	0
Q86VS8	Cluster of Protein Hook homolog 3	HOOK3	83 kDa	1	1	1	1	0.8	1	1.1	1.1	0	0
P20827	Ephrin-A1	EFNA1	24 kDa	1	1	0.8	0.8	0.8	1	1.1	1.1	1	0
Q9Y221	Isoform 2 of 60S ribosome subunit biogenesis protein NIP7	NIP7	15 kDa	1	1	0.9	0.9	1.1	0.9	1.1	1.1	0	0
Q96L92	Sorting nexin-27	SNX27	61 kDa	1	1	1.3	1.1	1	0.9	1.1	1.1	0	0
Q8NI36	Cluster of WD repeat-containing protein 36	WDR36	105 kDa	1	1	1	1	1	0.9	1.1	1.1	0	0
P51452	Cluster of Dual specificity protein phosphatase 3	DUSP3	20 kDa	1	1	0.9	0.9	1	0.9	1.1	1.1	0	0
P00450	Cluster of Ceruloplasmin	CP	122 kDa	1	1	0.6	0.7	1	0.9	1.1	1.1	1	0

H0Y4Z8	Cluster of Rab-like protein 6 (Fragment)	RABL6	26 kDa	1	1	1.1	1.1	0.9	0.9	1.1	1.1	0	0
C9JC60	Cluster of Nicotinate phosphoribosyltransferase	NAPRT1	60 kDa	1	1	1	1	0.9	0.9	1.1	1.1	0	0
P52298	Cluster of Isoform 2 of Nuclear cap-binding protein subunit	NCBP2	16 kDa	1	1	1	1	0.9	0.9	1.1	1.1	0	0
P62913	Cluster of Isoform 2 of 60S ribosomal protein L11	RPL11	20 kDa	No Va	No Va	No Value	No Value	No Value	No Value	No Val	No Val	0	0
P10643	Complement component C7	C7	94 kDa	1	1	1	1	0.9	0.9	1.1	1.1	3	0
P42677	40S ribosomal protein S27	RPS27	9 kDa	1	1	0.9	1	0.9	0.9	1.1	1.1	0	0
P29034	Cluster of Protein S100-A2	S100A2	11 kDa	1	1	0.9	1	0.9	0.9	1.1	1.1	0	0
Q99986	Cluster of Serine/threonine-protein kinase VRK1	VRK1	45 kDa	1	1	0.9	1	0.9	0.9	1.1	1.1	0	1
Q13243	Serine/arginine-rich splicing factor 5	SRSF5	31 kDa	1	1	0.8	1	0.9	0.9	1.1	1.1	0	0
B4DDT7	5'-AMP-activated protein kinase subunit gamma-1	PRKAG1	34 kDa	1	1	1	0.9	0.9	0.9	1.1	1.1	1	0
B4DI46	Testican-3	SPOCK3	36 kDa	1	1	1	0.9	0.9	0.9	1.1	1.1	1	0
B7Z1E5	Cell differentiation protein RCD1 homolog	RQCD1	37 kDa	1	1	0.9	0.9	0.9	0.9	1.1	1.1	1	0
C9JGI3	Thymidine phosphorylase (Fragment)	TYMP	46 kDa	1	1	0.9	0.9	0.9	0.9	1.1	1.1	0	0
D6RHG7	Serine incorporator 5	SERINC5	51 kDa	0.9	1	0.9	0.9	0.9	0.9	1.1	1.1	0	1
J3KMY7	Cluster of SRA stem-loop-interacting RNA-binding protein	SLIRP	12 kDa	1	1	0.8	0.9	0.9	0.9	1.1	1.1	0	0
J3KNW0	Cluster of FCH domain only protein 2	FCHO2	89 kDa	1	1	0.9	0.8	0.9	0.9	1.1	1.1	0	0
O43795	Unconventional myosin-Ib	MYO1B	132 kDa	1	1	0.8	0.8	0.9	0.9	1.1	1.1	0	0
O94763	Unconventional prefoldin RPB5 interactor 1	URI1	60 kDa	1	1	0.7	0.8	0.9	0.9	1.1	1.1	0	0
P05154	Plasma serine protease inhibitor	SERPINA5	46 kDa	1	1	0.9	0.7	0.9	0.9	1.1	1.1	1	0
P07711	Cluster of Cathepsin L1	CTSL	38 kDa	1	1	1	1	0.8	0.9	1.1	1.1	1	0
P15529	Membrane cofactor protein	CD46	44 kDa	1	1	0.7	0.8	0.8	0.9	1.1	1.1	0	1
P49770	Translation initiation factor eIF-2B subunit beta	EIF2B2	39 kDa	1	1	0.6	0.8	0.8	0.9	1.1	1.1	0	0
P52848	Cluster of Bifunctional heparan sulfate N-deacetylase/N-sul	NDST1	101 kDa	1	1	1	1.1	0.9	0.8	1.1	1.1	0	1
P60866	40S ribosomal protein S20	RPS20	13 kDa	1	1	1.1	1	0.9	0.8	1.1	1.1	0	0
Q00765	Receptor expression-enhancing protein 5	REEP5	21 kDa	1	1	1	1	0.9	0.8	1.1	1.1	0	1
Q03135	Caveolin-1	CAV1	20 kDa	1	1	1	0.9	0.9	0.8	1.1	1.1	0	1
Q12972	Nuclear inhibitor of protein phosphatase 1	PPP1R8	38 kDa	1	1	0.8	0.8	0.9	0.8	1.1	1.1	0	0
Q14157	Cluster of Isoform 4 of Ubiquitin-associated protein 2-like	UBAP2L	103 kDa	No Va	No Va	No Value	No Value	No Value	No Value	No Val	No Val	0	0
Q5T200	Cluster of Zinc finger CCCH domain-containing protein 13	ZC3H13	197 kDa	1	1	0.8	0.8	0.9	0.8	1.1	1.1	0	0
Q6PCE3	Glucose 1,6-bisphosphate synthase	PGM2L1	70 kDa	1	1	1	1.1	0.8	0.8	1.1	1.1	0	0
Q7Z6M1	Cluster of Rab9 effector protein with kelch motifs	RABEPK	41 kDa	1	1	0.9	0.9	0.8	0.8	1.1	1.1	0	0
Q86UN3	Reticulon-4 receptor-like 2	RTN4RL2	46 kDa	1	1	0.8	0.9	0.8	0.8	1.1	1.1	1	0
Q8IV38	Ankyrin repeat and MYND domain-containing protein 2	ANKMY2	49 kDa	1	1	0.8	0.9	0.8	0.8	1.1	1.1	0	0
Q8IXB1	DnaJ homolog subfamily C member 10	DNAJC10	91 kDa	1	1	0.9	0.8	0.8	0.8	1.1	1.1	0	1
Q92572	AP-3 complex subunit sigma-1	AP3S1	22 kDa	1	1	0.7	0.8	0.8	0.8	1.1	1.1	0	0
Q96AX1	Vacuolar protein sorting-associated protein 33A	VPS33A	68 kDa	1	1	0.8	0.7	0.8	0.8	1.1	1.1	0	0
Q96AY3	Cluster of Peptidyl-prolyl cis-trans isomerase FKBP10	FKBP10	64 kDa	1	1	0.7	0.7	0.8	0.8	1.1	1.1	1	0

Q96FK6	WD repeat-containing protein 89	WDR89	43 kDa	1	1	0.9	0.8	0.7	0.8	1.1	1.1	0	0
Q96GA7	Cluster of Serine dehydratase-like	SDSL	35 kDa	1	1	0.8	0.8	0.8	0.7	1.1	1.1	0	1
Q96I15	Cluster of Selenocysteine lyase	SCLY	48 kDa	1	1	0.8	0.7	0.8	0.7	1.1	1.1	0	1
Q99959	Plakophilin-2	PKP2	97 kDa	1	1	1.1	0.9	0.6	0.7	1.1	1.1	0	0
Q9BUI4	DNA-directed RNA polymerase III subunit RPC3	POLR3C	61 kDa	1	1	0.7	0.8	0.6	0.7	1.1	1.1	0	0
Q9BV38	WD repeat-containing protein 18	WDR18	47 kDa	1	1	0.5	0.6	0.6	0.7	1.1	1.1	0	0
Q9BWU0	Kanadaptin	SLC4A1AP	89 kDa	1	1	1.4	1.5	0.7	0.6	1.1	1.1	0	0
Q9BZH6	WD repeat-containing protein 11	WDR11	137 kDa	1	1	1.7	1.6	1.5	2.3	1	1.1	0	1
Q9H939	Proline-serine-threonine phosphatase-interacting protein 2	PSTPIP2	39 kDa	1	1	1.5	1.5	1.7	1.7	1	1.1	0	0
Q9UIC8	Cluster of Leucine carboxyl methyltransferase 1	LCMT1	38 kDa	1	1	1.5	1.3	1.6	1.7	1	1.1	0	0
Q9Y2S2	Lambda-crystallin homolog	CRYL1	35 kDa	1	1	1.7	1.9	1.5	1.6	1	1.1	0	0
Q9Y2V7	Cluster of Conserved oligomeric Golgi complex subunit 6	COG6	73 kDa	1	1	1.3	1.4	1.5	1.5	1	1.1	0	0
D6RJA0	Histone-lysine N-methyltransferase SETD7	SETD7	40 kDa	1	1	1.4	1.5	1.2	1.5	1	1.1	0	0
E1P660	Uncharacterized protein	ZNF207	53 kDa	1	1	1.5	1.5	1.4	1.3	1	1.1	1	0
J3KN32	REST corepressor 1	RCOR1	53 kDa	1	1	1.2	1	1.4	1.2	1	1.1	1	0
Q9GZN4	Brain-specific serine protease 4	PRSS22	34 kDa	1	1	1.2	1	1.3	1.2	1	1.1	1	1
Q9NT62	Ubiquitin-like-conjugating enzyme ATG3	ATG3	36 kDa	1	1	1	1.1	1.2	1.2	1	1.1	0	0
P51884	Lumican	LUM	38 kDa	1	1	0.9	1	1.1	1.2	1	1.1	3	0
Q9BRP4	Cluster of Proteasomal ATPase-associated factor 1	PAAF1	42 kDa	1	1	0.9	0.9	1.1	1.2	1	1.1	0	1
Q14669	Isoform 3 of E3 ubiquitin-protein ligase TRIP12	TRIP12	226 kDa	1	1	0.9	1.2	1	1.2	1	1.1	0	0
Q9ULX6	A-kinase anchor protein 8-like	AKAP8L	72 kDa	1	1	1.2	1.2	0.9	1.2	1	1.1	0	0
P21912	Succinate dehydrogenase [ubiquinone] iron-sulfur subunit,	SDHB	32 kDa	1	1	1.2	1.3	1.2	1.1	1	1.1	2	0
H7C3P7	Ras-related protein Ral-A (Fragment)	RALA	18 kDa	1	1	1.4	1.5	1.1	1.1	1	1.1	0	0
P23919	Cluster of Thymidylate kinase	DTYMK	24 kDa	1	1	1.2	1.2	1.1	1.1	1	1.1	0	0
F5H740	Voltage-dependent anion-selective channel protein 3	VDAC3	31 kDa	1	1	1.1	1.1	1.1	1.1	1	1.1	0	0
H3BV69	Cluster of Cytochrome c oxidase subunit 5A, mitochondrial	COX5A	12 kDa	1	1	0.9	1.1	1.1	1.1	1	1.1	0	0
P08651	Cluster of Nuclear factor 1 C-type	NFIC	56 kDa	1	1	1.1	0.9	1.1	1.1	1	1.1	0	0
B4DHE8	Cluster of RNA-binding protein Musashi homolog 2	MSI2	35 kDa	1	1	1.1	0.8	1.1	1.1	1	1.1	0	0
Q9UJH8	Cluster of Meteorin	METRN	31 kDa	1	1	1	0.8	1.1	1.1	1	1.1	1	0
Q01130	Cluster of Serine/arginine-rich splicing factor 2	SRSF2	25 kDa	1	1	1.4	1.6	1	1.1	1	1.1	0	0
C9JP00	Cluster of Muscleblind-like protein 1	MBNL1	38 kDa	No Va	No Va	No Value	No Value	No Value	No Value	No Val	No Val	1	0
J3KQ42	Tetraspanin-4	TSPAN4	28 kDa	1	1	1.2	1.2	1	1.1	1	1.1	0	1
G3V5I3	Alpha-1-antichymotrypsin His-Pro-less	SERPINA3	51 kDa	1	No Va	0.9	No Value	1.1	No Value	0.8	No Val	1	0
E7ENY0	Cluster of Alpha-adducin	ADD1	73 kDa	No Va	No Va	No Value	No Value	No Value	No Value	No Val	No Val	0	0
B1AJY7	26S proteasome non-ATPase regulatory subunit 10	PSMD10	21 kDa	1	1	1.1	1.1	1	1.1	1	1.1	0	0
B7ZKQ9	SCARB1 protein	SCARB1	54 kDa	1	1	0.9	1.1	1	1.1	1	1.1	0	1
F5H2H7	Semaphorin-3B	SEMA3B	83 kDa	1	1	1.2	1.4	0.7	1.1	1	1.1		

I3L1G1	Cluster of TOM1-like protein 1 (Fragment)	TOM1L1	28 kDa	1	1	1.1	1.1	1.2	1	1	1.1	0	0
O00151	PDZ and LIM domain protein 1	PDLIM1	36 kDa	1	1	1.1	1.1	1.1	1	1	1.1	0	0
O15479	Melanoma-associated antigen B2	MAGEB2	35 kDa	1	1	1.1	1.1	1.1	1	1	1.1	0	0
O43291	Kunitz-type protease inhibitor 2	SPINT2	28 kDa	1	1	1	1.1	1	1	1	1.1	1	1
O75175	Cluster of CCR4-NOT transcription complex subunit 3	CNOT3	82 kDa	1	1	1.1	1	1	1	1	1.1	0	0
O76071	Probable cytosolic iron-sulfur protein assembly protein CIA	CIAO1	38 kDa	1	1	1	1	1	1	1	1.1	0	0
O94906	Pre-mRNA-processing factor 6	PRPF6	107 kDa	1	1	1	1	1	1	1	1.1	0	0
O95400	CD2 antigen cytoplasmic tail-binding protein 2	CD2BP2	38 kDa	1	1	0.8	0.8	1	1	1	1.1	0	0
P13284	Gamma-interferon-inducible lysosomal thiol reductase	IFI30	28 kDa	1	1	1.1	1.2	0.9	1	1	1.1	1	1
P50281	Matrix metalloproteinase-14	MMP14	66 kDa	1	1	1.1	1.1	0.9	1	1	1.1	1	1
P53007	Tricarboxylate transport protein, mitochondrial	SLC25A1	34 kDa	1	1	1	1.1	0.9	1	1	1.1	0	0
P53582	Methionine aminopeptidase 1	METAP1	43 kDa	1	1	1	1.1	0.9	1	1	1.1	0	0
Q15397	Pumilio domain-containing protein KIAA0020	KIAA0020	74 kDa	1	1	1	1.1	0.9	1	1	1.1	0	0
Q6IC75	Cluster of ADP-ribosylation factor-binding protein GGA1	GGA1	72 kDa	1	1	1.1	1.2	0.8	1	1	1.1	0	0
Q709C8	Vacuolar protein sorting-associated protein 13C	VPS13C	422 kDa	1	1	1.1	1.1	0.9	0.9	1	1.1	0	0
Q8TBA6	Golgin subfamily A member 5	GOLGA5	83 kDa	1	1	1	1	0.9	0.9	1	1.1	0	1
Q96CV9	Cluster of Optineurin	OPTN	66 kDa	1	1	1	1	0.9	0.9	1	1.1	0	0
Q96HR9	Receptor expression-enhancing protein 6	REEP6	21 kDa	1	1	0.9	1	0.9	0.9	1	1.1	0	1
Q96JB2	Conserved oligomeric Golgi complex subunit 3	COG3	94 kDa	1	1	0.9	1	0.9	0.9	1	1.1	2	0
Q96QR8	Cluster of Transcriptional activator protein Pur-beta	PURB	33 kDa	1	1	0.9	0.9	0.9	0.9	1	1.1	0	0
Q9H6F5	Coiled-coil domain-containing protein 86	CCDC86	40 kDa	1	1	0.9	0.9	0.9	0.9	1	1.1	0	0
Q9NXR7	Cluster of Isoform 1 of BRCA1-A complex subunit BRE	BRE	47 kDa	1	1	0.8	0.9	0.9	0.9	1	1.1	0	0
Q9NY27	Serine/threonine-protein phosphatase 4 regulatory subunit 2	PPP4R2	47 kDa	1	1	1.2	1.1	0.8	0.9	1	1.1	0	0
Q9UKM7	Cluster of Endoplasmic reticulum mannosyl-oligosaccharid	MAN1B1	80 kDa	1	1	0.9	1	0.8	0.9	1	1.1	0	1
P61457	Pterin-4-alpha-carbinolamine dehydratase	PCBD1	12 kDa	1	1	0.9	0.9	0.8	0.9	1	1.1	0	0
P61962	DDB1- and CUL4-associated factor 7	DCAF7	39 kDa	1	1	0.8	0.9	0.8	0.9	1	1.1	0	0
F8W9X7	Coiled-coil domain-containing protein 93	CCDC93	73 kDa	1	1	0.9	0.7	0.8	0.9	1	1.1	0	0
B4DRL9	Cluster of Rab proteins geranylgeranyltransferase compone	CHM	57 kDa	1	1	0.7	0.7	0.8	0.9	1	1.1	0	0
J3KNF4	Cluster of Copper chaperone for superoxide dismutase	CCS	27 kDa	1	1	0.9	0.9	1	0.8	1	1.1	0	0
Q86V48	Cluster of Leucine zipper protein 1	LUZP1	120 kDa	1	1	1	0.9	0.9	0.8	1	1.1	0	0
H0Y7W6	Cluster of Formin-binding protein 1 (Fragment)	FNBP1	66 kDa	1	1	1	0.9	0.9	0.8	1	1.1	0	0
P36969	Phospholipid hydroperoxide glutathione peroxidase, mitoch	GPX4	22 kDa	1	1	0.8	0.9	0.9	0.8	1	1.1	1	0
Q14344	Cluster of Guanine nucleotide-binding protein subunit alpha	GNA13	44 kDa	1	1	1.2	1.2	0.8	0.8	1	1.1	0	0
H0Y435	Cluster of Microtubule-associated protein tau	MAPT	28 kDa	1	No Va	0.3	No Value	0.3	No Value	0.6	No Val	0	0
Q969T9	Cluster of WW domain-binding protein 2	WBP2	28 kDa	1	1	0.8	1	0.8	0.8	1	1.1	0	0
H0Y400	Cluster of Spliceosome RNA helicase DDX39B (Fragment)	DDX39B	22 kDa	1	1	0.9	0.9	0.8	0.8	1	1.1	0	0
E9PNU4	Cluster of Syntaxin-5	STX5	28 kDa	1	1	0.9	0.9	0.8	0.8	1	1.1	0	1

Q6GMV2	SET and MYND domain-containing protein 5	SMYD5	47 kDa	1	1	0.8	0.9	0.8	0.8	1	1.1	0	0
Q15427	Splicing factor 3B subunit 4	SF3B4	44 kDa	1	1	1.3	1.1	0.7	0.8	1	1.1	0	0
O60220	Mitochondrial import inner membrane translocase subunit T	TIMM8A	11 kDa	1	1	0.8	0.9	0.7	0.8	1	1.1	0	0
P20962	Parathymosin	PTMS	12 kDa	1	1	0.7	0.9	0.7	0.8	1	1.1	0	0
P83731	60S ribosomal protein L24	RPL24	18 kDa	1	1	0.7	0.8	0.7	0.8	1	1.1	0	0
P52657	Transcription initiation factor IIA subunit 2	GTF2A2	12 kDa	1	1	0.5	0.6	0.7	0.8	1	1.1	0	0
H7BZT4	Cluster of Uncharacterized protein		11 kDa	1	1	1.4	0.9	0.7	0.7	1	1.1		
F8W1L0	Cluster of MHC class I polypeptide-related sequence A	MICA	38 kDa	1	1	0.9	0.9	0.7	0.7	1	1.1	0	1
B4DTU4	Cluster of DNA ligase	LIG1	98 kDa	1	1	0.6	0.6	0.7	0.6	1	1.1	0	0
Q9H9S4	Calcium-binding protein 39-like	CAB39L	39 kDa	1	1	1.7	1.4	1.9	1.7	0.9	1.1	0	0
Q9Y2I1	Nischarin	NISCH	167 kDa	1	1	0.9	0.6	0.7	1	0.9	1.1	0	0
Q9Y5K8	V-type proton ATPase subunit D	ATP6V1D	28 kDa	1	1	1.2	1.2	1.1	1.5	0.9	1.1	0	0
Q96RN5	Mediator of RNA polymerase II transcription subunit 15	MED15	87 kDa	1	1	1.2	1.5	1.1	1.4	0.9	1.1	0	0
P02452	Collagen alpha-1(I) chain	COL1A1	139 kDa	1	1	1.2	1.2	1	1.2	0.9	1.1	1	0
Q658Y4	Cluster of Protein FAM91A1	FAM91A1	94 kDa	1	1	1	1.2	1	1.2	0.9	1.1	0	0
B4DJ85	Glomulin	GLMN	67 kDa	1	1	1.1	1	0.9	1.2	0.9	1.1	0	0
B4DR61	Cluster of Protein transport protein Sec61 subunit alpha iso	SEC61A1	53 kDa	1	1	1.2	1.1	1	1.1	0.9	1.1	0	1
C9J2K4	V-type proton ATPase subunit F	ATP6V1F	16 kDa	1	1	1.1	1.1	1	1.1	0.9	1.1	0	0
E7ESX1	Chordin	CHRD	102 kDa	1	1	1	1.1	1	1.1	0.9	1.1	1	0
F5GYQ1	V-type proton ATPase subunit d 1	ATP6V0D1	45 kDa	1	1	0.7	0.9	0.8	1.1	0.9	1.1	0	0
F5GZA8	SH3 domain-binding protein 1	SH3BP1	55 kDa	1	1	0.9	1.2	1	1	0.9	1.1	1	0
F5GZS0	Probable ATP-dependent RNA helicase DHX36	DHX36	113 kDa	1	1	1.3	1.4	0.9	1	0.9	1.1	0	1
F5H7J9	Cluster of Low-density lipoprotein receptor-related protein	LRP6	175 kDa	1	1	0.9	0.7	0.9	1	0.9	1.1	0	1
H0Y5T9	Bromodomain-containing protein 2 (Fragment)	BRD2	88 kDa	1	1	1.1	1.1	0.8	1	0.9	1.1	0	0
H0Y9X1	Translation machinery-associated protein 16 (Fragment)	TMA16	28 kDa	1	1	1	1	0.7	1	0.9	1.1	0	0
J3KN82	Cluster of Probable methylthioribulose-1-phosphate dehydr	APIP	29 kDa	1	1	0.9	0.6	0.7	1	0.9	1.1	0	0
O14828	Secretory carrier-associated membrane protein 3	SCAMP3	38 kDa	1	1	1	1	0.9	0.9	0.9	1.1	0	1
O43823	A-kinase anchor protein 8	AKAP8	76 kDa	1	1	0.9	0.9	0.9	0.9	0.9	1.1	0	0
O60524	Nuclear export mediator factor NEMF	NEMF	123 kDa	1	1	0.8	0.9	0.9	0.9	0.9	1.1	0	0
O95340	Bifunctional 3'-phosphoadenosine 5'-phosphosulfate syntha	PAPSS2	70 kDa	1	1	1	0.6	0.9	0.9	0.9	1.1	0	0
O95633	Follistatin-related protein 3	FSTL3	28 kDa	1	1	0.8	0.9	0.8	0.9	0.9	1.1	1	0
O95865	Cluster of N(G),N(G)-dimethylarginine dimethylaminohydr	DDAH2	30 kDa	1	1	1.1	0.8	1.1	0.8	0.9	1.1	0	0
P29966	Myristoylated alanine-rich C-kinase substrate	MARCKS	32 kDa	1	1	0.7	0.8	0.7	0.8	0.9	1.1	0	0
P47985	Cluster of Cytochrome b-c1 complex subunit Rieske, mitoc	UQCRFS1	30 kDa	No V	No Va	No Value	No Valu	No Valu	No Valu	No Val	No Val	0	0
Q00403	Transcription initiation factor IIB	GTF2B	35 kDa	1	1	0.6	0.7	0.7	0.8	0.9	1.1	0	0
Q14185	Dedicator of cytokinesis protein 1	DOCK1	215 kDa	1	1	1.2	1.1	1	1.1	0.8	0.9	0	0
Q14766	Cluster of Latent-transforming growth factor beta-binding p	LTBP1	187 kDa	1	1	1	1.6	1	1.5	0.8	1.1	1	0

Q14919	Isoform 2 of Dr1-associated corepressor	DRAP1	23 kDa	1	1	0.8	0.9	0.9	1	1.2	1.1	0	0
Q8TD16	Protein bicaudal D homolog 2	BICD2	94 kDa	1	1	1.1	1.2	0.9	1.2	0.8	1.1	0	0
Q96J01	THO complex subunit 3	THOC3	39 kDa	1	1	1	1.1	0.8	1.2	0.8	1.1	1	1
Q9BRG1	Vacuolar protein-sorting-associated protein 25	VPS25	21 kDa	1	1	1	1.3	1	1.1	0.8	1.1	0	0
Q9BTV6	WD repeat-containing protein 85	DPH7	51 kDa	1	1	1	1	0.9	1.1	0.8	1.1	0	0
Q9H1Y0	Autophagy protein 5	ATG5	32 kDa	1	1	1.1	1.1	0.9	1	0.8	1.1	1	0
Q9H2C0	Gigaxonin	GAN	68 kDa	1	1	0.8	1	0.9	0.9	0.8	1.1	0	0
Q9UIJ7	GTP:AMP phosphotransferase, mitochondrial	AK3	26 kDa	1	1	0.7	0.7	0.9	0.8	0.7	0.6	0	0
Q9Y606	tRNA pseudouridine synthase A, mitochondrial	PUS1	47 kDa	1	1	0.9	0.8	1	0.8	0.8	0.7	0	0
C9J384	Uncharacterized protein C3orf26 (Fragment)	CMSS1	26 kDa	1	1	0.9	0.8	1	0.8	1	1	0	0
C9JLU1	DNA-directed RNA polymerases I, II, and III subunit RPAE	POLR2H	17 kDa	1	1	1.3	0.9	1.4	0.9	2	1.2	0	0
Q6P1N0	Coiled-coil and C2 domain-containing protein 1A	CC2D1A	104 kDa	1	1	0.7	1.1	0.8	1.2	1	1.5	0	0
Q99459	Cell division cycle 5-like protein	CDC5L	92 kDa	1	1	1.7	1.1	1.8	1.1	1.7	1.2	0	0
Q9BQ67	Glutamate-rich WD repeat-containing protein 1	GRWD1	49 kDa	1	1	1.1	0.9	1.1	0.9	1.7	1.2	0	0
Q9Y294	Cluster of Histone chaperone ASF1A	ASF1A	23 kDa	1	1	1.3	1.1	0.9	0.8	1.7	1.2	0	0
O95376	E3 ubiquitin-protein ligase ARIH2	ARIH2	58 kDa	1	1	1.6	1.6	1.5	1.6	1.6	1.2	0	0
Q8IWJ2	Cluster of GRIP and coiled-coil domain-containing protein	GCC2	196 kDa	1	1	1.6	1.5	1.3	1.2	1.6	1.2	0	0
Q8WXI4	Cluster of Isoform 2 of Acyl-coenzyme A thioesterase 11	ACOT11	67 kDa	1	1	1.8	1.3	1.5	1.1	1.6	1.2	0	0
P22670	MHC class II regulatory factor RFX1	RFX1	105 kDa	1	1	1	0.8	1	0.9	1.6	1.2	0	0
D6REX5	Selenoprotein P (Fragment)	SEPP1	35 kDa	1	1	0.7	0.9	0.9	0.9	1.6	1.2	1	1
O43617	Trafficking protein particle complex subunit 3	TRAPPC3	20 kDa	1	1	1.4	1.4	1.3	0.8	1.6	1.2	0	0
P30043	Flavin reductase (NADPH)	BLVRB	22 kDa	1	1	1.2	1.1	1.1	1.2	1.5	1.2	1	0
E9PCT1	Cluster of Serine/arginine repetitive matrix protein 1	SRRM1	104 kDa	No Va	No Va	No Value	No Value	No Value	No Value	No Val	No Val	0	0
J3KNF8	Cytochrome b5 type B	CYB5B	17 kDa	1	1	1.4	1.2	1	1.2	1.5	1.2	0	1
O00471	Exocyst complex component 5	EXOC5	82 kDa	1	1	1	1	1.2	1	1.5	1.2	0	0
O95163	Cluster of Elongator complex protein 1	IKBKAP	150 kDa	1	1	0.8	0.9	0.8	0.8	1.5	1.2	0	0
P60983	Cluster of Glia maturation factor beta	GMFB	17 kDa	1	1	0.9	0.6	0.8	0.6	1.5	1.2	0	0
P49821	NADH dehydrogenase [ubiquinone] flavoprotein 1, mitocho	NDUFV1	51 kDa	1	1	2.1	1.7	1.9	1.9	1.4	1.2	1	0
Q9Y2S0	DNA-directed RNA polymerases I and III subunit RPAC2	POLR1D	15 kDa	1	1	2	1.7	1.7	1.5	1.4	1.2	0	0
Q9NQY0	Bridging integrator 3	BIN3	30 kDa	1	1	1.6	1.4	1.5	1.4	1.4	1.2	0	0
B8ZZC8	Cluster of Methyltransferase-like protein 5	METTL5	28 kDa	1	1	1	0.8	1.3	1.2	1.4	1.2	0	0
P02794	Cluster of Ferritin heavy chain	FTH1	21 kDa	1	1	1.2	1.1	1.2	1	1.4	1.2	0	1
Q9Y3B3	Cluster of Transmembrane emp24 domain-containing protei	TMED7	25 kDa	1	1	1.1	1	1	1	1.4	1.2	1	1
P0DJI8	Cluster of Serum amyloid A-1 protein	SAA1	14 kDa	1	1	1	1	1	1	1.4	1.2	1	0
Q9BQ61	Uncharacterized protein C19orf43	C19orf43	18 kDa	1	1	0.9	0.9	1	1	1.4	1.2	0	0
F5GX77	tRNA methyltransferase 112 homolog	TRMT112	12 kDa	1	1	0.9	0.8	1	1	1.4	1.2	0	0
E9PL10	Transcription factor BTF3 homolog 4	BTF3L4	16 kDa	1	1	1.1	1	1	0.9	1.4	1.2	0	0

Q9UBU8	Cluster of Mortality factor 4-like protein 1	MORF4L1	41 kDa	1	1	0.8	0.8	1	0.9	1.4	1.2	0	0
P19827	Inter-alpha-trypsin inhibitor heavy chain H1	ITIH1	101 kDa	1	1	0.9	0.8	1	0.8	1.4	1.2	1	0
P49257	Protein ERGIC-53	LMAN1	58 kDa	1	1	0.9	0.8	0.8	0.8	1.4	1.2	0	1
Q8NC96	Cluster of Adaptin ear-binding coat-associated protein 1	NECAP1	30 kDa	1	1	0.7	0.7	0.7	0.8	1.4	1.2	0	0
Q9UII2	ATPase inhibitor, mitochondrial	ATPIF1	12 kDa	1	1	0.9	0.7	0.9	0.7	1.4	1.2	0	0
Q04760	Lactoylglutathione lyase	GLO1	21 kDa	1	1	0.8	0.9	0.8	0.7	1.4	1.2	0	0
Q4VXU2	Cluster of Polyadenylate-binding protein 1-like	PABPC1L	68 kDa	No Va	No Va	No Value	No Value	No Value	No Value	No Val	No Val	0	0
O94923	D-glucuronyl C5-epimerase	GLCE	70 kDa	No Va	1	No Value	1.1	No Value	1	No Val	1.3	1	0
H0Y6E7	Cluster of RNA-binding motif protein, X chromosome (Fra	RBMX	32 kDa	1	1	0.7	0.6	0.7	0.7	1.4	1.2	0	0
P27986	Cluster of Phosphatidylinositol 3-kinase regulatory subunit	PIK3R1	84 kDa	1	1	1	0.6	1	0.6	1.4	1.2	0	0
Q8N1G2	Cap-specific mRNA (nucleoside-2'-O-)-methyltransferase 1	CMTR1	95 kDa	1	1	3.1	4.6	3.4	3.9	1.3	1.2	0	0
Q9Y3E7	Cluster of Isoform 4 of Charged multivesicular body protei	CHMP3	21 kDa	1	1	1.7	1.6	1.4	1.5	1.3	1.2	0	0
O95881	Thioredoxin domain-containing protein 12	TXNDC12	19 kDa	1	1	1.4	1.5	1.4	1.4	1.3	1.2	1	0
F8VSZ4	Cluster of Plexin-A1	PLXNA1	209 kDa	1	1	1.2	1.3	1.4	1.4	1.3	1.2	0	1
P35250	Cluster of Replication factor C subunit 2	RFC2	39 kDa	1	1	1.7	1.7	1.3	1.4	1.3	1.2	0	0
G5E9J2	Cluster of Adenine phosphoribosyltransferase	APRT	15 kDa	No Va	No Va	No Value	No Value	No Value	No Value	No Val	No Val	0	0
C9JZR2	Cluster of Catenin delta-1	CTNND1	105 kDa	No Va	No Va	No Value	No Value	No Value	No Value	No Val	No Val	0	0
Q9H1B7	Cluster of Interferon regulatory factor 2-binding protein-lik	IRF2BPL	83 kDa	1	1	1.5	1.5	1.3	1.4	1.3	1.2	0	0
Q6ZRH9	Uncharacterized protein FLJ46347		54 kDa	1	1	1.2	1.1	1.4	1.3	1.3	1.2		
P50238	Cysteine-rich protein 1	CRIP1	9 kDa	1	1	1.5	1.4	1.3	1.3	1.3	1.2	0	0
B5MBX2	Transcobalamin-2	TCN2	47 kDa	1	1	1.4	1.3	1.3	1.3	1.3	1.2	1	0
B9EGP5	MON2 protein	MON2	190 kDa	1	1	1.1	1	1.3	1.3	1.3	1.2	0	0
D6RHI9	Cluster of Ribonuclease T2 (Fragment)	RNASET2	29 kDa	1	1	1.5	1.3	1.3	1.2	1.3	1.2	1	0
E9PED3	Ankycorbin	RAI14	107 kDa	1	1	1.2	1.2	1.3	1.2	1.3	1.2	0	0
F5GXF7	Cluster of Zinc finger protein 185	ZNF185	77 kDa	1	1	1.3	1.2	1.2	1.2	1.3	1.2	0	0
H0Y5J4	Cluster of Drebrin-like protein (Fragment)	DBNL	40 kDa	1	1	1.1	0.8	1.3	1.1	1.3	1.2	1	0
H7C1F6	Cluster of ES1 protein homolog, mitochondrial (Fragment)	C21orf33	27 kDa	1	1	1.8	1.4	1.2	1.1	1.3	1.2	1	0
O60885	Cluster of Bromodomain-containing protein 4	BRD4	152 kDa	1	1	1.3	1.4	1.2	1.1	1.3	1.2	0	0
O94760	Cluster of N(G),N(G)-dimethylarginine dimethylaminohydr	DDAH1	31 kDa	1	1	1.2	1.2	1.2	1.1	1.3	1.2	0	0
O94910	Latrophilin-1	LPHN1	163 kDa	1	1	1	1	1.2	1.1	1.3	1.2	0	1
O95786	Probable ATP-dependent RNA helicase DDX58	DDX58	107 kDa	1	1	1.3	1.2	1.1	1.1	1.3	1.2	0	0
P32321	Deoxycytidylate deaminase	DCTD	20 kDa	1	1	1.2	1.2	1.1	1.1	1.3	1.2	0	0
P37198	Nuclear pore glycoprotein p62	NUP62	53 kDa	1	1	0.9	0.9	1.1	1.1	1.3	1.2	0	0
P49247	Ribose-5-phosphate isomerase	RPIA	33 kDa	1	1	0.8	1.2	0.9	1.1	1.3	1.2	0	0
P54725	UV excision repair protein RAD23 homolog A	RAD23A	40 kDa	1	1	1.2	1.1	1.3	1	1.3	1.2	0	0
P81605	Dermcidin	DCD	11 kDa	1	1	1.1	1.1	1.1	1	1.3	1.2	1	0
Q03169	Cluster of Tumor necrosis factor alpha-induced protein 2	TNFAIP2	73 kDa	1	1	1	1	1.1	1	1.3	1.2	0	0

Q13442	28 kDa heat- and acid-stable phosphoprotein	PDAP1	21 kDa	1	1	1.1	0.9	1.1	1	1.3	1.2	0	0
Q14498	RNA-binding protein 39	RBM39	59 kDa	1	1	1.1	1.1	1	1	1.3	1.2	0	0
Q15276	Rab GTPase-binding effector protein 1	RABEP1	99 kDa	1	1	1.2	1	1	1	1.3	1.2	0	0
Q5VW36	Focadhesin	FOCAD	200 kDa	1	1	0.6	0.9	1	1	1.3	1.2	1	1
Q8NBF6	Cluster of Late secretory pathway protein AVL9 homolog	AVL9	72 kDa	1	1	0.7	0.8	1	1	1.3	1.2	0	1
Q969S3	Zinc finger protein 622	ZNF622	54 kDa	1	1	0.7	0.7	1	1	1.3	1.2	0	0
Q96I25	Splicing factor 45	RBM17	45 kDa	1	1	1.1	1	0.9	0.9	1.3	1.2	0	0
Q99523	Sortilin	SORT1	92 kDa	1	1	1	1	0.9	0.9	1.3	1.2	0	1
Q9H9E3	Cluster of Conserved oligomeric Golgi complex subunit 4	COG4	89 kDa	1	1	0.9	0.9	0.9	0.9	1.3	1.2	0	0
Q9UNE7	Cluster of E3 ubiquitin-protein ligase CHIP	STUB1	35 kDa	1	1	0.9	0.8	0.9	0.9	1.3	1.2	0	0
Q9UP83	Conserved oligomeric Golgi complex subunit 5	COG5	93 kDa	1	1	0.6	0.7	0.9	0.9	1.3	1.2	0	0
D6RFG8	Deoxycytidine kinase	DCK	37 kDa	1	1	1	0.9	0.8	0.9	1.3	1.2	0	0
P19623	Spermidine synthase	SRM	34 kDa	1	1	1	0.9	1	0.8	1.3	1.2	0	0
Q01081	Cluster of Splicing factor U2AF 35 kDa subunit	U2AF1	28 kDa	1	1	0.8	0.7	1	0.8	1.3	1.2	0	0
Q13112	Chromatin assembly factor 1 subunit B	CHAF1B	61 kDa	1	1	1	0.9	0.9	0.8	1.3	1.2	0	0
Q15654	Cluster of Thyroid receptor-interacting protein 6	TRIP6	50 kDa	1	1	0.7	0.7	0.9	0.8	1.3	1.2	0	0
Q86WR0	Coiled-coil domain-containing protein 25	CCDC25	24 kDa	1	1	0.6	0.7	0.9	0.8	1.3	1.2	0	0
Q8IY81	pre-rRNA processing protein FTSJ3	FTSJ3	97 kDa	1	1	0.7	0.6	0.9	0.8	1.3	1.2	0	0
Q9UKZ9	Procollagen C-endopeptidase enhancer 2	PCOLCE2	46 kDa	1	1	0.7	0.8	0.8	0.8	1.3	1.2	1	0
Q14746	Cluster of Conserved oligomeric Golgi complex subunit 2	COG2	83 kDa	1	1	0.7	0.7	0.8	0.8	1.3	1.2	0	0
A2A2V1	Major prion protein (Fragment)	PRNP	27 kDa	1	1	0.9	0.9	0.7	0.8	1.3	1.2	1	1
P53680	AP-2 complex subunit sigma	AP2S1	17 kDa	1	1	0.8	0.6	0.9	0.7	1.3	1.2	0	0
Q8TB72	Cluster of Pumilio homolog 2	PUM2	114 kDa	1	1	0.9	0.9	0.8	0.7	1.3	1.2	0	0
Q9NPF2	Carbohydrate sulfotransferase 11	CHST11	42 kDa	1	1	0.8	0.8	0.8	0.6	1.3	1.2	1	0
P78318	Immunoglobulin-binding protein 1	IGBP1	39 kDa	1	1	1.9	1.5	1.5	1.5	1.2	1.2	0	0
Q8NCN5	Pyruvate dehydrogenase phosphatase regulatory subunit, mi	PDPR	99 kDa	1	1	1.4	1.5	1.4	1.5	1.2	1.2	0	0
Q5TH30	NDRG family member 3	NDRG3	43 kDa	1	1	1.5	1.4	1.5	1.4	1.2	1.2	0	1
H7BYY3	Mediator of RNA polymerase II transcription subunit 23	MED23	157 kDa	1	1	1.3	1.4	1.4	1.4	1.2	1.2	0	1
Q9HAT2	Sialate O-acetylesterase	SIAE	58 kDa	1	1	1.3	1.3	1.4	1.4	1.2	1.2	1	0
Q5VWZ2	Lysophospholipase-like protein 1	LYPLAL1	26 kDa	1	1	1.2	1.2	1.4	1.4	1.2	1.2	0	0
O43414	ERI1 exoribonuclease 3	ERI3	37 kDa	1	1	1	1	1.3	1.4	1.2	1.2	0	0
Q9NR09	Baculoviral IAP repeat-containing protein 6	BIRC6	530 kDa	1	1	1.6	1.5	1.2	1.4	1.2	1.2	0	0
Q13907	Cluster of Isopentenyl-diphosphate Delta-isomerase 1	IDI1	26 kDa	1	1	1.5	1.4	1.5	1.3	1.2	1.2	0	0
G3XAK1	Cluster of Hepatocyte growth factor-like protein beta chain	MST1	82 kDa	1	1	1.4	1.6	1.4	1.3	1.2	1.2	1	0
B4DK25	Cluster of Arginyl-tRNA--protein transferase 1	ATE1	46 kDa	1	1	1.7	1.8	1.3	1.3	1.2	1.2	0	0
F5H2L4	Acyl-coenzyme A thioesterase 13	ACOT13	12 kDa	1	1	1.5	1.6	1.3	1.3	1.2	1.2	0	0
B4DFL2	Isocitrate dehydrogenase [NADP]	IDH2	45 kDa	1	1	1.2	1.2	1.3	1.3	1.2	1.2	0	0

Q5T0D2	Cluster of Cytidine monophosphate (UMP-CMP) kinase 1, c	CMPK1	19 kDa	1	1	1	1.1	1.2	1.3	1.2	1.2	1	0
P00387	Cluster of Isoform 3 of NADH-cytochrome b5 reductase 3	CYB5R3	38 kDa	1	1	1.2	1.2	1.2	1.2	1.2	1.2	0	1
F5GXP4	ADP-ribosylation factor-like protein 6-interacting protein 1	ARL6IP1	20 kDa	1	1	1.1	1.2	1.1	1.2	1.2	1.2	0	1
Q9UBF6	RING-box protein 2	RNF7	13 kDa	1	1	0.9	0.9	1.1	1.2	1.2	1.2	0	0
A2ACR1	Proteasome subunit beta type	PSMB9	21 kDa	1	1	0.8	0.9	0.9	1.2	1.2	1.2	0	0
G3V529	ATP-dependent RNA helicase DDX24	DDX24	91 kDa	1	1	1.3	1.4	1.1	1.1	1.2	1.2	0	0
P45984	Cluster of Isoform Beta-1 of Mitogen-activated protein kina	MAPK9	44 kDa	1	1	1.1	1.1	1.1	1.1	1.2	1.2	0	0
F8W8S3	Cluster of Transducin-like enhancer protein 3	TLE3	83 kDa	1	1	1.1	1.1	1.1	1.1	1.2	1.2	0	0
Q9C0J8	Cluster of pre-mRNA 3' end processing protein WDR33	WDR33	146 kDa	1	1	1.1	1	1.1	1.1	1.2	1.2	0	1
B8ZZN6	Small ubiquitin-related modifier 1	SUMO1	17 kDa	1	1	1	1	1.1	1.1	1.2	1.2	0	0
C9JP16	Cartilage-associated protein	CRTAP	41 kDa	1	1	1	1	1.1	1.1	1.2	1.2	1	0
E9PB93	DNA-directed RNA polymerase II subunit RPB4	POLR2D	12 kDa	1	1	1	1	1.1	1.1	1.2	1.2	0	0
H3BTH8	Hyaluronan and proteoglycan link protein 3	HAPLN3	48 kDa	1	1	0.9	1	1.1	1.1	1.2	1.2	1	1
P68402	Cluster of Platelet-activating factor acetylhydrolase IB subu	PAFAH1B2	26 kDa	1	1	0.8	0.6	1.1	1.1	1.2	1.2	0	0
Q9H0R4	Haloacid dehalogenase-like hydrolase domain-containing pr	HDHD2	29 kDa	1	1	1.2	1.2	1	1.1	1.2	1.2	0	0
B4DQU7	Cluster of Caspase-7 subunit p20	CASP7	32 kDa	1	1	1.2	1.2	1	1.1	1.2	1.2	0	0
Q9ULV0	Cluster of Unconventional myosin-Vb	MYO5B	214 kDa	1	1	0.9	1	1	1.1	1.2	1.2	0	0
J3KNL6	Cluster of Protein transport protein Sec16A	SEC16A	252 kDa	1	1	1.2	1.2	1.5	1	1.2	1.2	1	1
H0Y2W2	Cluster of ATPase family AAA domain-containing protein 3	ATAD3A	64 kDa	1	1	0.9	1.1	1.2	1	1.2	1.2	0	0
C1IDX9	ATG12 autophagy related 12 homolog (S. cerevisiae), isofo	ATG12	21 kDa	1	1	1.4	1.4	1.1	1	1.2	1.2	0	0
O95372	Acyl-protein thioesterase 2	LYPLA2	25 kDa	1	1	1	1	1.1	1	1.2	1.2	0	0
B4DQI4	Abhydrolase domain-containing protein 14B	ABHD14B	20 kDa	1	1	0.9	0.9	1.1	1	1.2	1.2	0	0
B8ZZG1	MAGUK p55 subfamily member 6	MPP6	49 kDa	1	1	1.7	1.7	1	1	1.2	1.2	0	0
J3QK89	Calcium homeostasis endoplasmic reticulum protein	CHERP	105 kDa	1	1	1	1.1	1	1	1.2	1.2	0	0
J3QTA2	BAG family molecular chaperone regulator 1	BAG1	39 kDa	1	1	1.1	1	1	1	1.2	1.2	0	0
O75439	Cluster of Mitochondrial-processing peptidase subunit beta	PMPCB	54 kDa	1	1	0.9	1	1	1	1.2	1.2	0	0
O95983	Isoform 2 of Methyl-CpG-binding domain protein 3	MBD3	29 kDa	1	1	0.9	0.9	1	1	1.2	1.2	0	0
P06493	Cyclin-dependent kinase 1	CDK1	34 kDa	1	1	0.9	0.9	1	1	1.2	1.2	0	0
P19388	DNA-directed RNA polymerases I, II, and III subunit RPAB	POLR2E	25 kDa	1	1	1.2	1	1	0.9	1.2	1.2	0	0
Q14247	Cluster of Isoform 2 of Src substrate cortactin	CTTN	71 kDa	No Va	No Va	No Value	No Value	No Value	No Value	No Val	No Val	1	0
Q5T1M5	FK506-binding protein 15	FKBP15	134 kDa	1	1	0.9	0.9	1	0.9	1.2	1.2	0	0
Q676U5	Isoform 2 of Autophagy-related protein 16-1	ATG16L1	66 kDa	1	1	0.9	0.9	1	0.9	1.2	1.2	0	0
Q6P1N9	Isoform 2 of Putative deoxyribonuclease TATDN1	TATDN1	28 kDa	1	1	1.3	1.3	0.9	0.9	1.2	1.2	0	1
Q75MJ1	ATP-binding cassette sub-family F member 2	ABCF2	72 kDa	1	1	1.1	1.1	0.9	0.9	1.2	1.2	0	0
Q7Z7M0	Multiple epidermal growth factor-like domains protein 8	MEGF8	303 kDa	1	1	1	1	0.9	0.9	1.2	1.2	0	1
Q86U44	N6-adenosine-methyltransferase 70 kDa subunit	METTL3	64 kDa	1	1	1	1	0.9	0.9	1.2	1.2	0	0
Q8TED0	Cluster of U3 small nucleolar RNA-associated protein 15 ho	UTP15	58 kDa	1	1	0.9	1	0.9	0.9	1.2	1.2	0	0

Q8WVC0	RNA polymerase-associated protein LEO1	LEO1	75 kDa	1	1	1	0.9	0.9	0.9	1.2	1.2	0	0
Q9BRA2	Cluster of Thioredoxin domain-containing protein 17	TXNDC17	14 kDa	1	1	0.9	0.9	0.9	0.9	1.2	1.2	0	0
Q9H2U2	Cluster of Isoform 4 of Inorganic pyrophosphatase 2, mitocl	PPA2	19 kDa	No Va	No Va	No Value	No Value	No Value	No Value	No Val	No Val	0	1
Q9H3N1	Thioredoxin-related transmembrane protein 1	TMX1	32 kDa	1	1	0.9	0.9	0.9	0.9	1.2	1.2	1	1
Q9H410	Kinetochore-associated protein DSN1 homolog	DSN1	40 kDa	1	1	0.9	0.9	0.9	0.9	1.2	1.2	0	0
Q9H6Y2	WD repeat-containing protein 55	WDR55	42 kDa	1	1	0.8	0.8	0.9	0.9	1.2	1.2	0	0
Q9NRF8	CTP synthase 2	CTPS2	66 kDa	1	1	0.8	0.8	0.9	0.9	1.2	1.2	0	0
Q9UBL6	Copine-7	CPNE7	70 kDa	1	1	0.8	0.8	0.9	0.9	1.2	1.2	0	0
Q9UJX3	Cluster of Anaphase-promoting complex subunit 7	ANAPC7	67 kDa	1	1	0.9	0.9	0.8	0.9	1.2	1.2	0	0
Q9UL15	BAG family molecular chaperone regulator 5	BAG5	51 kDa	1	1	0.6	0.7	0.8	0.9	1.2	1.2	0	0
Q9ULP9	TBC1 domain family member 24	TBC1D24	63 kDa	1	1	1.4	1.8	0.9	1.1	0.5	0.6	0	0
Q9Y5L4	Mitochondrial import inner membrane translocase subunit T	TIMM13	11 kDa	1	1	1	1	0.9	0.8	1.2	1.2	0	0
G5EA58	Cluster of Hydroxymethylbilane synthase, isoform CRA_d	HMBS	35 kDa	1	1	1	1	0.9	0.8	1.2	1.2	0	0
P30530	Tyrosine-protein kinase receptor UFO	AXL	98 kDa	1	1	1	1	0.8	0.8	1.2	1.2	0	1
Q10713	Cluster of Mitochondrial-processing peptidase subunit alpha	PMPCA	58 kDa	1	1	0.9	0.9	0.8	0.8	1.2	1.2	0	0
Q6P597	Kinesin light chain 3	KLC3	55 kDa	1	1	0.8	0.9	0.8	0.8	1.2	1.2	0	0
Q7LGC8	Carbohydrate sulfotransferase 3	CHST3	55 kDa	1	1	0.8	0.8	0.8	0.8	1.2	1.2	0	1
Q6ZMI0	Cluster of Protein phosphatase 1 regulatory subunit 21	PPP1R21	88 kDa	1	1	0.8	0.8	0.8	0.8	1.2	1.2	0	0
Q7LBC6	Cluster of Lysine-specific demethylase 3B	KDM3B	192 kDa	1	1	1.2	1.3	0.7	0.8	1.2	1.2	1	0
Q8IYB1	Protein MB21D2	MB21D2	56 kDa	1	1	1.1	1	0.9	0.7	1.2	1.2	0	0
Q9H1A4	Cluster of Anaphase-promoting complex subunit 1	ANAPC1	217 kDa	1	1	0.8	0.8	0.8	0.7	1.2	1.2	0	1
G3V141	Collagen triple helix repeat containing 1, isoform CRA_c	CTHRC1	25 kDa	1	1	0.8	0.8	0.7	0.7	1.2	1.2	1	0
P34096	Ribonuclease 4	RNASE4	17 kDa	1	1	0.7	0.7	0.7	0.7	1.2	1.2	1	0
Q15785	Mitochondrial import receptor subunit TOM34	TOMM34	35 kDa	1	1	0.6	0.5	0.5	0.5	1.2	1.2	0	0
Q9H814	Phosphorylated adapter RNA export protein	PHAX	44 kDa	1	1	1.3	1.4	1.6	1.6	1.1	1.2	0	0
Q9NV06	DDB1- and CUL4-associated factor 13	DCAF13	51 kDa	1	1	1.6	1.6	1.4	1.5	1.1	1.2	0	0
Q8N684	Cluster of Isoform 3 of Cleavage and polyadenylation speci	CPSF7	56 kDa	1	1	1.2	1.3	1.4	1.5	1.1	1.2	0	0
Q5TCM7	Protein tyrosine phosphatase type IVA 1	PTP4A1	17 kDa	1	1	1.4	1.4	1.2	1.4	1.1	1.2	0	0
P55769	Cluster of NHP2-like protein 1	NHP2L1	14 kDa	1	1	1	1.4	0.9	1.4	1.1	1.2	0	0
A6NMQ1	DNA polymerase	POLA1	166 kDa	1	1	1.5	1.6	1.2	1.3	1.1	1.2	0	0
Q9UEY8	Isoform 1 of Gamma-adducin	ADD3	76 kDa	1	1	1.2	1.3	1.2	1.3	1.1	1.2	0	0
Q96ST2	Protein IWS1 homolog	IWS1	92 kDa	1	1	1.1	1.1	1.2	1.3	1.1	1.2	0	0
P07099	Cluster of Epoxide hydrolase 1	EPHX1	53 kDa	1	1	1.3	1.1	1.4	1.2	1.1	1.2	0	0
Q9NYB0	Cluster of Telomeric repeat-binding factor 2-interacting pro	TERF2IP	44 kDa	1	1	1.3	1.3	1.3	1.2	1.1	1.2	0	0
Q9BXR0	Cluster of Queuine tRNA-ribosyltransferase	QTRT1	44 kDa	1	1	1.2	1.3	1.2	1.2	1.1	1.2	0	0
Q9BTL3	RNMT-activating mini protein	FAM103A1	14 kDa	1	1	1.4	1.4	1.1	1.2	1.1	1.2	0	0
O75844	CAAX prenyl protease 1 homolog	ZMPSTE24	55 kDa	1	1	1.2	1.4	1.1	1.2	1.1	1.2	0	1

Q00577	Transcriptional activator protein Pur-alpha	PURA	35 kDa	1	1	1	1.3	1.1	1.2	1.1	1.2	0	0
F5GWK3	Pre-mRNA-splicing factor RBM22	RBM22	41 kDa	1	1	1.4	1.5	1.1	1.1	1.1	1.2	0	0
H0Y9D8	Inorganic pyrophosphatase 2, mitochondrial (Fragment)	PPA2	26 kDa	1	1	1.3	1.2	1.1	1.1	1.1	1.2	0	1
Q96N67	Cluster of Dedicator of cytokinesis protein 7	DOCK7	243 kDa	1	1	1.1	1.1	1.1	1.1	1.1	1.2	0	0
F5GYQ8	Acyl-coenzyme A oxidase	ACOX1	70 kDa	1	1	1.1	1	1.1	1.1	1.1	1.2	0	0
B5MCP9	40S ribosomal protein S7	RPS7	21 kDa	1	1	1	1	1.1	1.1	1.1	1.2	0	0
Q92793	Cluster of CREB-binding protein	CREBBP	265 kDa	1	1	1	1.1	1	1.1	1.1	1.2	0	0
P41208	Cluster of Centrin-2	CETN2	20 kDa	1	1	0.9	1.3	1	1	1.1	1.2	0	0
Q9Y6I3	Isoform 2 of Epsin-1	EPN1	69 kDa	1	1	1.1	1.1	1	1	1.1	1.2	0	0
O43760	Synaptogyrin-2	SYNGR2	25 kDa	1	1	1	1.1	1	1	1.1	1.2	0	1
Q12929	Cluster of Epidermal growth factor receptor kinase substrat	EPS8	92 kDa	1	1	1.1	1	1	1	1.1	1.2	0	0
P49711	Cluster of Transcriptional repressor CTCF	CTCF	83 kDa	1	1	1	1	1	1	1.1	1.2	0	0
Q9BPU6	Dihydropyrimidinase-related protein 5	DPYSL5	61 kDa	1	1	0.9	0.9	1	1	1.1	1.2	0	0
P46778	60S ribosomal protein L21	RPL21	19 kDa	1	1	0.7	0.7	1	1	1.1	1.2	0	0
E7EN89	Cluster of Toll interacting protein, isoform CRA_b	TOLLIP	27 kDa	1	1	1.2	1.1	0.9	1	1.1	1.2	0	0
Q9H078	Cluster of Caseinolytic peptidase B protein homolog	CLPB	79 kDa	1	1	1	1.1	0.9	1	1.1	1.2	0	0
Q96PD2	Cluster of Discoidin, CUB and LCCL domain-containing pr	DCBLD2	85 kDa	1	1	0.7	1.1	0.9	1	1.1	1.2	0	1
Q08345	Cluster of Epithelial discoidin domain-containing receptor 1	DDR1	101 kDa	1	1	1	1.3	0.8	1	1.1	1.2	1	1
Q5R372	Cluster of Isoform 8 of Rab GTPase-activating protein 1-lik	RABGAP1L	43 kDa	1	1	0.8	0.9	1	0.9	1.1	1.2	0	1
O14556	Glyceraldehyde-3-phosphate dehydrogenase, testis-specific	GAPDHS	45 kDa	No Va	1	No Value	1	No Valu	0.7	No Val	0.4	0	0
F8WAF2	Cluster of CCR4-NOT transcription complex subunit 10	CNOT10	89 kDa	1	1	1	1	0.9	0.9	1.1	1.2	0	0
G3XAP6	Cartilage oligomeric matrix protein	COMP	80 kDa	1	1	1	1	0.9	0.9	1.1	1.2	1	0
O14787	Transportin-2	TNPO2	101 kDa	1	1	0.7	0.8	0.9	0.9	1.1	1.2	0	0
O14949	Cytochrome b-c1 complex subunit 8	UQCRQ	10 kDa	1	1	0.7	0.8	0.9	0.9	1.1	1.2	0	0
O15446	Isoform 2 of DNA-directed RNA polymerase I subunit RPA	CD3EAP	55 kDa	1	1	0.9	0.7	0.9	0.9	1.1	1.2	0	0
P11171	Protein 4.1	EPB41	97 kDa	1	1	0.8	0.9	0.8	0.9	1.1	1.2	0	0
P38432	Coilin	COIL	63 kDa	1	1	0.8	0.8	0.8	0.9	1.1	1.2	0	0
P46977	Dolichyl-diphosphooligosaccharide--protein glycosyltransfe	STT3A	81 kDa	1	1	0.6	1	0.7	0.9	1.1	1.2	0	1
P51116	Fragile X mental retardation syndrome-related protein 2	FXR2	74 kDa	1	1	0.9	0.9	1	0.8	1.1	1.2	0	0
P57081	Cluster of tRNA (guanine-N(7)-)-methyltransferase subunit	WDR4	45 kDa	1	1	1.1	1.1	0.9	0.8	1.1	1.2	0	0
Q06330	Isoform 7 of Recombining binding protein suppressor of ha	RBPJ	54 kDa	1	1	0.9	0.8	0.9	0.8	1.1	1.2	0	0
Q13573	SNW domain-containing protein 1	SNW1	61 kDa	1	1	0.9	1	0.8	0.8	1.1	1.2	0	0
Q6QNY0	Biogenesis of lysosome-related organelles complex 1 subun	BLOC1S3	21 kDa	1	1	0.9	1	0.8	0.8	1.1	1.2	0	0
Q6YP21	Kynurenine--oxoglutarate transaminase 3	CCBL2	51 kDa	1	1	0.8	1	0.8	0.8	1.1	1.2	0	0
Q7L2J0	7SK snRNA methylphosphate capping enzyme	MEPCE	74 kDa	1	1	0.8	0.9	0.8	0.8	1.1	1.2	0	0
Q8IXL6	Cluster of Extracellular serine/threonine protein kinase Fam	FAM20C	66 kDa	1	1	0.8	0.9	0.8	0.8	1.1	1.2	2	0
Q8N8R5	UPF0565 protein C2orf69	C2orf69	43 kDa	1	1	0.9	0.8	0.8	0.8	1.1	1.2	3	0

A6NJZ9	Nucleolar complex protein 3 homolog	NOC3L	93 kDa	1	1	0.8	0.7	0.8	0.8	1.1	1.2	0	0
D6RD47	40S ribosomal protein S23	RPS23	15 kDa	1	1	0.8	1	0.7	0.8	1.1	1.2	0	0
F5H1Y4	Golgi-associated PDZ and coiled-coil motif-containing prot	GOPC	51 kDa	1	1	0.8	0.9	0.7	0.8	1.1	1.2	0	0
P11177	Pyruvate dehydrogenase E1 component subunit beta, mitocl	PDHB	39 kDa	1	1	0.8	0.8	0.7	0.8	1.1	1.2	0	0
Q13643	Four and a half LIM domains protein 3	FHL3	31 kDa	1	1	0.7	0.7	0.7	0.8	1.1	1.2	0	0
Q14562	ATP-dependent RNA helicase DHX8	DHX8	139 kDa	1	1	0.9	0.9	0.8	0.7	1.1	1.2	1	0
Q6P988	Protein notum homolog	NOTUM	56 kDa	1	1	0.7	0.8	0.7	0.7	1.1	1.2	1	0
Q96MW5	Conserved oligomeric Golgi complex subunit 8	COG8	68 kDa	1	1	1.1	0.7	0.7	0.7	1.1	1.2	0	0
Q9Y6A5	Transforming acidic coiled-coil-containing protein 3	TACC3	90 kDa	1	1	0.8	0.7	0.7	0.7	1.1	1.2	0	0
H0Y4R2	Cluster of NADPH--cytochrome P450 reductase (Fragment)	POR	82 kDa	1	1	0.8	0.6	0.9	0.8	1.7	1.6	0	1
Q9BY76	Angiopoietin-related protein 4	ANGPTL4	45 kDa	1	1	1.6	1.4	1.4	1.5	1	1.2	1	0
Q9NXH8	Torsin-4A	TOR4A	47 kDa	1	1	1.4	1.4	1.3	1.4	1	1.2	0	0
I3L1Q2	Cluster of B-cell CLL/lymphoma 7 protein family member (	BCL7C	33 kDa	1	1	0.8	1.1	1.2	1.4	1	1.2	0	0
Q6DKK2	Tetratricopeptide repeat protein 19, mitochondrial	TTC19	42 kDa	1	1	1.2	1.1	1.4	1.2	1	1.2	1	0
Q8TCA0	Cluster of Leucine-rich repeat-containing protein 20	LRRC20	21 kDa	1	1	1.3	1	1.5	1.1	1	1.2	0	0
Q96GQ7	Probable ATP-dependent RNA helicase DDX27	DDX27	90 kDa	1	1	1.1	1.4	1.2	1.1	1	1.2	0	0
O96008	Mitochondrial import receptor subunit TOM40 homolog	TOMM40	38 kDa	1	1	1.1	1.2	1.1	1.1	1	1.2	0	0
H7C525	Cluster of Coiled-coil domain-containing protein 58 (Fragm	CCDC58	16 kDa	1	1	1	1	1.1	1.1	1	1.2	0	0
Q8NFI3	Cluster of Cytosolic endo-beta-N-acetylglucosaminidase	ENGASE	84 kDa	1	1	1.2	1.4	1	1.1	1	1.2	0	0
Q7Z7H5	Isoform 3 of Transmembrane emp24 domain-containing pro	TMED4	21 kDa	1	1	1.1	1.3	1	1.1	1	1.2	1	1
I3L430	Cluster of Heme oxygenase 2	HMOX2	33 kDa	1	1	0.9	1	0.9	1.1	1	1.2	0	1
P50613	Cluster of Cyclin-dependent kinase 7	CDK7	39 kDa	1	1	1	1	0.8	1.1	1	1.2	0	0
Q9H936	Cluster of Mitochondrial glutamate carrier 1	SLC25A22	34 kDa	1	1	1.1	1.1	0.9	1	1	1.2	0	1
Q15024	Exosome complex component RRP42	EXOSC7	32 kDa	1	1	1	0.9	0.9	1	1	1.2	0	0
Q9NP72	Ras-related protein Rab-18	RAB18	23 kDa	1	1	0.8	0.9	0.8	1	1	1.2	0	1
C9JYQ9	60S ribosomal protein L22-like 1	RPL22L1	14 kDa	1	1	0.8	0.8	0.8	0.9	1	1.2	0	0
Q86Y82	Syntaxin-12	STX12	32 kDa	1	1	0.7	0.7	0.7	0.8	1	1.2	0	1
E9PEI9	Lanosterol synthase	LSS	82 kDa	1	1	0.3	0.4	0.4	0.4	1	1.2	0	0
P14854	Cytochrome c oxidase subunit 6B1	COX6B1	10 kDa	1	1	1.2	1.8	1	1.6	0.9	1.2	0	0
Q9UKK3	Poly [ADP-ribose] polymerase 4	PARP4	193 kDa	1	1	1.2	1.6	1.2	1.4	0.9	1.2	0	0
Q5VYB9	Cluster of Low density lipoprotein receptor-related protein	LRP11	35 kDa	1	1	1.3	1.3	1.2	1.3	0.9	1.2	1	1
B7Z642	Neuronal membrane glycoprotein M6-a	GPM6A	30 kDa	1	1	1.5	1.9	1.1	1.1	0.9	1.2	0	1
Q9Y5P4	Isoform 3 of Collagen type IV alpha-3-binding protein	COL4A3BP	84 kDa	1	1	1	1	1.1	1.1	0.9	1.2	0	0
Q5GLZ8	Cluster of Isoform 6 of Probable E3 ubiquitin-protein ligase	HERC4	107 kDa	No Va	No Va	No Value	No Value	No Value	No Value	No Val	No Val	0	1
Q9BRJ7	Cluster of Protein syndesmos	NUDT16L1	23 kDa	1	1	1	0.9	0.9	1	0.9	1.2	0	0
P26572	Cluster of Alpha-1,3-mannosyl-glycoprotein 2-beta-N-acety	MGAT1	51 kDa	1	1	0.8	0.9	0.7	1	0.9	1.2	0	1
B4DPY8	Cluster of TOX high mobility group box family member 4	TOX4	64 kDa	1	1	0.7	0.7	1	0.8	0.9	1.2	0	0

O60512	Beta-1,4-galactosyltransferase 3	B4GALT3	44 kDa	1	1	0.7	0.7	0.9	0.8	0.9	1.2	0	0
P17050	Alpha-N-acetylgalactosaminidase	NAGA	47 kDa	1	1	0.8	1.1	1.1	0.7	0.9	1.2	1	0
P30049	ATP synthase subunit delta, mitochondrial	ATP5D	17 kDa	1	1	0.8	0.7	0.8	0.7	0.9	1.2	0	0
Q9Y248	DNA replication complex GINS protein PSF2	GINS2	21 kDa	1	1	0.4	0.5	0.4	0.6	0.9	1.2	0	0
P49356	Protein farnesyltransferase subunit beta	FNTB	49 kDa	1	1	1.2	1.1	0.8	0.9	1	1.1	0	0
Q13444	Cluster of Isoform 6 of Disintegrin and metalloproteinase d	ADAM15	89 kDa	1	1	1	1.1	0.9	1.1	0.8	1.2	1	1
Q86SQ0	Cluster of Pleckstrin homology-like domain family B memb	PHLDB2	142 kDa	1	1	1.1	1.2	1.2	1	0.8	1.2	0	1
H0Y9V9	Cluster of 60S ribosomal protein L9 (Fragment)	RPL9	22 kDa	1	1	1.4	1.2	1.3	1.3	1.1	1	0	0
Q96J02	Cluster of E3 ubiquitin-protein ligase Itchy homolog	ITCH	103 kDa	1	1	1.2	1.4	1	1.1	1	1.2	0	0
O75354	Cluster of Ectonucleoside triphosphate diphosphohydrolase	ENTPD6	53 kDa	1	1	0.9	1.1	1	1.1	0.8	0.9	0	1
Q9H0H5	Cluster of Rac GTPase-activating protein 1	RACGAP1	71 kDa	1	1	0.9	1	0.9	1.4	1.5	2.2	0	0
Q13642	Cluster of Isoform 4 of Four and a half LIM domains protei	FHL1	35 kDa	1	1	1.1	1.5	1.2	1.3	0.9	1	1	0
Q96DM3	Uncharacterized protein C18orf8	C18orf8	75 kDa	1	1	1.1	1.1	1.1	1	0.7	0.7	0	0
B4DX68	Lymphokine-activated killer T-cell-originated protein kinas	PBK	37 kDa	1	1	1.5	1.2	1.5	1.2	1.6	1.5	0	0
Q96P16	Regulation of nuclear pre-mRNA domain-containing protei	RPRD1A	36 kDa	1	1	0.9	1.1	1	1	2	1.3	0	0
Q86W92	Cluster of Isoform 2 of Liprin-beta-1	PPFIBP1	113 kDa	1	1	1	0.8	0.9	0.9	2	1.3	0	0
B7ZC38	Endophilin-B2	SH3GLB2	44 kDa	1	1	2	1.3	1.9	1.2	1.9	1.3	0	0
Q14739	Lamin-B receptor	LBR	71 kDa	1	1	1.2	1.3	1.4	1.1	1.8	1.3	0	1
P80217	Interferon-induced 35 kDa protein	IFI35	32 kDa	1	1	1.2	1.3	1.4	1.5	1.3	1.3	0	0
Q5ZPR3	Cluster of CD276 antigen	CD276	57 kDa	1	1	1.1	1.2	1	0.8	1.7	1.3	1	1
J3QL81	Cluster of Mitochondrial enolase superfamily member 1	ENOSF1	41 kDa	1	1	1.5	1.2	1.3	1.2	1.6	1.3	0	0
B1AP13	Cluster of CD55 molecule, decay accelerating factor for co	CD55	49 kDa	No V	No Va	No Value	No Value	No Value	No Value	No Val	No Val	1	1
Q9Y6N5	Cluster of Sulfide:quinone oxidoreductase, mitochondrial	SQRDL	50 kDa	1	1	0.8	0.8	1.3	1	1.6	1.3	0	0
J3QR68	Haptoglobin beta chain (Fragment)	HP	45 kDa	1	1	1.2	1.1	1.2	1	1.6	1.3	1	0
P06732	Creatine kinase M-type	CKM	43 kDa	1	1	1.1	1	1.2	0.9	1.6	1.3	0	0
Q6P1J9	Parafibromin	CDC73	61 kDa	1	1	1	0.9	1.1	0.9	1.6	1.3	0	0
Q8TEB1	Cluster of DDB1- and CUL4-associated factor 11	DCAF11	62 kDa	1	1	0.9	0.7	0.9	0.9	1.6	1.3	0	0
O75503	Ceroid-lipofuscinosis neuronal protein 5	CLN5	41 kDa	1	1	0.5	0.7	0.8	1	2.1	3.1	0	1
P62304	Small nuclear ribonucleoprotein E	SNRPE	11 kDa	1	1	1	0.9	0.8	0.8	1.6	1.3	0	0
Q9NX08	COMM domain-containing protein 8	COMMD8	21 kDa	1	1	2.2	1.8	2.4	1.8	1.5	1.3	0	0
Q86UA1	Pre-mRNA-processing factor 39	PRPF39	78 kDa	1	1	1.6	1.3	1.4	1.5	1.5	1.3	0	0
B4E2N0	Protein LZIC	LZIC	24 kDa	1	1	1.4	1.3	1.4	1.4	1.5	1.3	0	0
Q9H583	HEAT repeat-containing protein 1	HEATR1	242 kDa	1	1	1.3	1.6	1.3	1.4	1.5	1.3	0	0
Q9BTU6	Cluster of Phosphatidylinositol 4-kinase type 2-alpha	PI4K2A	54 kDa	1	1	1.3	1.2	1.6	1.3	1.5	1.3	0	0
Q9NUM4	Transmembrane protein 106B	TMEM106B	31 kDa	1	1	1.2	1	1.3	1.2	1.5	1.3	0	1
Q96E11	Cluster of Ribosome-recycling factor, mitochondrial	MRRF	29 kDa	1	1	1.2	1.2	1.2	1.2	1.5	1.3	0	0
E9PHA2	Cluster of Condensin complex subunit 2	NCAPH	82 kDa	No V	No Va	No Value	No Value	No Value	No Value	No Val	No Val	0	0

Q5TZA2	Cluster of Rootletin	CROCC	229 kDa	No Va	1	No Value	1.2	No Value	0.9	No Val	0.8	0	0
B4DRY3	MOB kinase activator 1B	MOB1B	25 kDa	1	1	1.2	1.1	1.1	1.2	1.5	1.3	0	0
C9JRZ6	Coiled-coil-helix-coiled-coil-helix domain-containing prote	CHCHD3	27 kDa	1	1	1.2	1.2	1.3	1.1	1.5	1.3	0	1
E5RFP0	NudC domain-containing protein 2	NUDCD2	15 kDa	1	1	1.1	1	1	1	1.5	1.3	0	0
P02795	Cluster of Metallothionein-2	MT2A	6 kDa	1	1	0.8	0.7	1	1	1.5	1.3	0	0
P36405	ADP-ribosylation factor-like protein 3	ARL3	20 kDa	1	1	1	1.1	0.9	1	1.5	1.3	0	0
P46821	Microtubule-associated protein 1B	MAP1B	271 kDa	1	1	1	1	1.2	0.9	1.5	1.3	0	0
Q00534	Cyclin-dependent kinase 6	CDK6	37 kDa	1	1	1.1	1.2	0.9	0.9	1.5	1.3	0	0
Q06587	Isoform 2 of E3 ubiquitin-protein ligase RING1	RING1	39 kDa	1	1	1.6	1.1	1.2	0.8	1.5	1.3	0	0
Q2M2I8	Isoform 2 of AP2-associated protein kinase 1	AAK1	94 kDa	1	1	0.9	0.9	0.9	0.8	1.5	1.3	0	0
Q92785	Zinc finger protein ubi-d4	DPF2	44 kDa	1	1	0.9	0.9	0.9	0.8	1.5	1.3	0	0
Q96IZ0	PRKC apoptosis WT1 regulator protein	PAWR	37 kDa	1	1	0.8	0.9	0.7	0.8	1.5	1.3	0	0
Q96QG7	Myotubularin-related protein 9	MTMR9	63 kDa	1	1	0.7	0.7	0.7	0.7	1.5	1.3	0	0
Q9BSG5	Isoform 2 of Retbindin	RTBDN	28 kDa	1	1	2	1.7	2	1.9	1.4	1.3	1	1
Q9BUP3	Oxidoreductase HTATIP2	HTATIP2	27 kDa	1	1	1.6	1.7	1.5	1.5	1.4	1.3	1	1
Q9H008	Phospholysine phosphohistidine inorganic pyrophosphate p	LHPP	29 kDa	1	1	1.3	1.2	1.4	1.4	1.4	1.3	0	0
Q9H4G4	Golgi-associated plant pathogenesis-related protein 1	GLIPR2	17 kDa	1	1	1.2	1.1	1.4	1.4	1.4	1.3	0	0
E9PC74	Translation initiation factor eIF-2B subunit epsilon	EIF2B5	78 kDa	1	1	1.5	1	1.3	1.1	1.4	1.3	0	0
H3BSH7	Cirhin (Fragment)	CIRH1A	78 kDa	1	1	1.3	1.3	1.2	1.1	1.4	1.3	1	0
O00401	Neural Wiskott-Aldrich syndrome protein	WASL	55 kDa	1	1	1.2	1.3	1.2	1.1	1.4	1.3	0	0
O60826	Coiled-coil domain-containing protein 22	CCDC22	71 kDa	1	1	1.3	1.2	1.1	1.1	1.4	1.3	0	0
O75792	Ribonuclease H2 subunit A	RNASEH2A	33 kDa	1	1	1.1	1.1	1.1	1.1	1.4	1.3	0	0
Q96G46	tRNA-dihydrouridine(47) synthase [NAD(P)(+)]-like	DUS3L	73 kDa	1	1	1	1	1.1	1.1	1.4	1.3	0	0
H7BYW6	Platelet-derived growth factor subunit A (Fragment)	PDGFA	23 kDa	1	1	1.2	1.1	1.1	1	1.4	1.3	1	0
P82094	TATA element modulatory factor	TMF1	123 kDa	1	1	1	1	1.1	1	1.4	1.3	0	0
Q9NWS0	PIH1 domain-containing protein 1	PIH1D1	32 kDa	1	1	1	1.1	1	1	1.4	1.3	0	0
O94832	Unconventional myosin-Id	MYO1D	116 kDa	1	1	1	0.9	1	1	1.4	1.3	0	0
Q9UBI6	Guanine nucleotide-binding protein G(I)/G(S)/G(O) subunit	GNG12	8 kDa	1	1	0.8	0.9	0.8	1	1.4	1.3	0	0
Q8IXK2	Polypeptide N-acetylgalactosaminyltransferase 12	GALNT12	67 kDa	1	1	0.8	1.2	0.7	1	1.4	1.3	2	0
Q9NSI2	Protein FAM207A	FAM207A	25 kDa	1	1	1	1	1	0.9	1.4	1.3	0	0
Q9BWF3	Cluster of RNA-binding protein 4	RBM4	40 kDa	1	1	1.4	1.3	0.9	0.9	1.4	1.3	0	0
Q05209	Tyrosine-protein phosphatase non-receptor type 12	PTPN12	88 kDa	1	1	1	1	0.9	0.9	1.4	1.3	0	0
Q96FX7	Cluster of tRNA (adenine(58)-N(1))-methyltransferase cata	TRMT61A	31 kDa	1	1	1	0.9	0.9	0.9	1.4	1.3	0	0
Q96QZ7	Membrane-associated guanylate kinase, WW and PDZ dom	MAGI1	165 kDa	1	1	0.9	0.9	0.9	0.9	1.4	1.3	0	0
O75947	ATP synthase subunit d, mitochondrial	ATP5H	18 kDa	1	1	0.9	0.9	0.9	0.9	1.4	1.3	0	0
Q9Y3B2	Exosome complex component CSL4	EXOSC1	21 kDa	1	1	0.8	0.7	0.9	0.9	1.4	1.3	0	0
C9J2P0	Cluster of Ubiquitin-conjugating enzyme E2 E1 (Fragment)	UBE2E1	16 kDa	1	1	0.8	0.7	0.9	0.9	1.4	1.3	0	0

Q9GZX3	Cluster of Carbohydrate sulfotransferase 6	CHST6	44 kDa	1	1	0.7	0.9	0.8	0.9	1.4	1.3	1	0
P10301	Ras-related protein R-Ras	RRAS	23 kDa	1	1	0.9	0.8	0.9	0.8	1.4	1.3	0	0
Q9H7D7	WD repeat-containing protein 26	WDR26	72 kDa	1	1	1.1	0.9	0.8	0.8	1.4	1.3	0	0
Q14264	HERV-R_7q21.2 provirus ancestral Env polyprotein	ERV3-1	68 kDa	1	1	0.9	0.9	0.8	0.8	1.4	1.3	3	0
Q8N5K1	CDGSH iron-sulfur domain-containing protein 2	CISD2	15 kDa	1	1	0.8	0.8	0.8	0.8	1.4	1.3	0	1
O43665	Regulator of G-protein signaling 10	RGS10	20 kDa	1	1	0.8	0.8	0.8	0.8	1.4	1.3	0	0
B4E0G4	PHD finger protein 6	PHF6	38 kDa	1	1	0.7	0.8	0.8	0.8	1.4	1.3	0	0
Q08722	Isoform OA3-312 of Leukocyte surface antigen CD47	CD47	34 kDa	1	1	0.6	0.7	0.7	0.7	1.4	1.3	0	1
O75909	Isoform 4 of Cyclin-K	CCNK	66 kDa	1	1	1.5	1.6	0.7	0.6	1.4	1.3	0	0
Q6PCB0	von Willebrand factor A domain-containing protein 1	VWA1	47 kDa	1	1	1.5	1.6	1.5	1.8	1.3	1.3	1	0
E7EMK3	Flotillin-2	FLOT2	53 kDa	1	1	1.6	1.5	1.5	1.5	1.3	1.3	0	0
Q70IA6	MOB kinase activator 2	MOB2	27 kDa	1	1	1.2	1.3	1.4	1.5	1.3	1.3	0	0
B8ZZZ1	AP-1 complex subunit sigma-3	AP1S3	16 kDa	1	1	1.7	1.7	1.4	1.4	1.3	1.3	0	0
Q9H788	Cluster of SH2 domain-containing protein 4A	SH2D4A	53 kDa	1	1	1.7	1.6	1.3	1.4	1.3	1.3	0	0
Q96JB5	Isoform 3 of CDK5 regulatory subunit-associated protein 3	CDK5RAP3	31 kDa	1	1	1.1	1.1	1.3	1.4	1.3	1.3	0	0
G3V4W4	Glucosamine 6-phosphate N-acetyltransferase (Fragment)	GNPNAT1	18 kDa	1	1	1.8	1.7	1.4	1.3	1.3	1.3	0	0
Q9NQ55	Cluster of Suppressor of SWI4 1 homolog	PPAN	53 kDa	1	1	1.3	1.2	1.4	1.3	1.3	1.3	0	1
Q5R2V8	C2orf4 protein	MEMO1	34 kDa	1	1	1.3	1.3	1.2	1.3	1.3	1.3	0	1
Q13595	Isoform Short of Transformer-2 protein homolog alpha	TRA2A	13 kDa	1	1	1.3	1.2	1.2	1.3	1.3	1.3	0	0
Q9UQ53	Cluster of Alpha-1,3-mannosyl-glycoprotein 4-beta-N-acety	MGAT4B	63 kDa	1	1	1.1	1.1	1	1.3	1.3	1.3	1	0
H3BN51	Dynactin subunit 5	DCTN5	17 kDa	1	1	1.7	1.4	1.3	1.2	1.3	1.3	0	0
Q8TEW0	Cluster of Isoform 2 of Partitioning defective 3 homolog	PARD3	151 kDa	No Va	No Va	No Value	No Value	No Value	No Value	No Val	No Val	0	0
E5RJP2	Cluster of Vinexin (Fragment)	SORBS3	20 kDa	1	1	1.5	1.4	1.3	1.2	1.3	1.3	0	0
B7Z4K6	Deoxyribonuclease-2-alpha	DNASE2	34 kDa	1	1	1.2	1.3	1.2	1.2	1.3	1.3	1	0
B7Z2R2	Cytochrome b-c1 complex subunit 7	UQCRB	19 kDa	1	1	1.3	1.2	1.2	1.2	1.3	1.3	0	0
C9JME2	FERM, RhoGEF and pleckstrin domain-containing protein	FARP1	122 kDa	1	1	1.1	1.1	1.2	1.2	1.3	1.3	0	0
P61927	60S ribosomal protein L37	RPL37	11 kDa	1	1	1.1	1.1	1.2	1.2	1.3	1.3	0	0
P17706	Tyrosine-protein phosphatase non-receptor type 2	PTPN2	48 kDa	1	1	1	1.3	1.1	1.2	1.3	1.3	0	1
Q92538	Golgi-specific brefeldin A-resistance guanine nucleotide ex	GBF1	206 kDa	1	1	1	1	1.1	1.2	1.3	1.3	0	0
D6RBN5	OCIA domain-containing protein 1	OCIAD1	24 kDa	1	1	1	0.9	1.1	1.2	1.3	1.3	0	1
P31751	Cluster of RAC-beta serine/threonine-protein kinase	AKT2	56 kDa	1	1	1	1.2	1	1.2	1.3	1.3	0	0
Q9UN37	Cluster of Vacuolar protein sorting-associated protein 4A	VPS4A	49 kDa	1	1	1.4	1.2	1.2	1.1	1.3	1.3	0	0
E9PK59	N-terminal kinase-like protein	SCYL1	86 kDa	1	1	1.3	1.2	1.2	1.1	1.3	1.3	0	0
Q15056	Eukaryotic translation initiation factor 4H	EIF4H	27 kDa	1	1	1.8	1.7	1.1	1.1	1.3	1.3	0	0
F8WCA0	Vesicle-associated membrane protein 2	VAMP2	13 kDa	1	1	1.4	1.5	1.1	1.1	1.3	1.3	0	1
P49642	Cluster of DNA primase small subunit	PRIM1	50 kDa	1	1	1.4	1.4	1.1	1.1	1.3	1.3	0	0
A8MU44	Protein Hook homolog 1	HOOK1	80 kDa	1	1.1	1.2	1.2	1.1	1.1	1.3	1.3	0	0

C9J2Q2	ATP synthase mitochondrial F1 complex assembly factor 2	ATPAF2	23 kDa	1	1	1.1	1.1	1.1	1.1	1.3	1.3	0	0
Q92954	Cluster of Proteoglycan 4	PRG4	151 kDa	1	1	1	1.1	1.1	1.1	1.3	1.3	1	0
B5ME97	Cluster of Septin 10, isoform CRA_c	SEPT10	63 kDa	No Va	No Va	No Value	No Value	No Valu	No Value	No Val	No Values		
P46934	Cluster of E3 ubiquitin-protein ligase NEDD4	NEDD4	149 kDa	No Va	No Va	No Value	No Value	No Valu	No Value	No Val	No Val	0	0
O75165	DnaJ homolog subfamily C member 13	DNAJC13	254 kDa	No Va	1	No Value	0.7	No Valu	0.8	No Val	0.6	0	1
Q92917	G patch domain and KOW motifs-containing protein	GPKOW	52 kDa	1	1	0.7	0.7	1.1	1.1	1.3	1.3	0	0
Q9UJX6	Isoform 2 of Anaphase-promoting complex subunit 2	ANAPC2	93 kDa	1	1	1.3	1.2	1	1.1	1.3	1.3	0	0
Q68BL7	Cluster of Olfactomedin-like protein 2A	OLFML2A	73 kDa	1	1	1.2	1.1	1	1.1	1.3	1.3	1	0
Q0VDF9	Cluster of Heat shock 70 kDa protein 14	HSPA14	55 kDa	1	1	1	1.1	1	1.1	1.3	1.3	0	0
Q9Y4E6	WD repeat-containing protein 7	WDR7	164 kDa	1	1	1.4	1.3	1.1	1	1.3	1.3	0	0
H0YH84	Cluster of CCR4-NOT transcription complex subunit 2 (Fra	CNOT2	20 kDa	No Va	No Va	No Value	No Value	No Valu	No Value	No Val	No Val	0	0
Q9HBD1	Cluster of RING finger and CCCH-type zinc finger domain-	RC3H2	132 kDa	1	1	1	0.9	1.1	1	1.3	1.3	0	0
C9JMV9	Cluster of Protein ABHD14A-ACY1	ABHD14A-A(	56 kDa	1	1	1.2	1.2	1	1	1.3	1.3	0	0
Q9NYC9	Dynein heavy chain 9, axonemal	DNAH9	512 kDa	1	1	1.1	0.9	0.9	1	1.3	1.3	0	0
B5MC40	Transcriptional repressor p66-alpha	GATAD2A	70 kDa	1	1	0.9	0.9	0.9	1	1.3	1.3	0	0
E9PF19	Transducin beta-like protein 2	TBL2	46 kDa	1	1	0.9	1	0.7	1	1.3	1.3	1	0
F8VY86	Tumor suppressor p53-binding protein 1	TP53BP1	214 kDa	1	1	1.1	1.1	1	0.9	1.3	1.3	0	0
O95297	Myelin protein zero-like protein 1	MPZL1	29 kDa	1	1	0.9	0.9	1	0.9	1.3	1.3	1	1
Q14195	Isoform LCRMP-4 of Dihydropyrimidinase-related protein	DPYSL3	74 kDa	1	1	0.9	0.8	1	0.9	1.3	1.3	0	0
Q6P275	Cluster of Cohesin subunit SA-1	STAG1	140 kDa	1	1	1	1	0.9	0.9	1.3	1.3	0	0
Q8WX93	Cluster of Palladin	PALLD	151 kDa	No Va	No Va	No Value	No Value	No Valu	No Value	No Val	No Val	0	0
Q96EE3	Nucleoporin SEH1	SEH1L	40 kDa	1	1	1	1	0.9	0.9	1.3	1.3	0	0
Q99714	3-hydroxyacyl-CoA dehydrogenase type-2	HSD17B10	27 kDa	1	1	0.9	0.9	0.9	0.9	1.3	1.3	0	0
Q9BPX3	Cluster of Condensin complex subunit 3	NCAPG	114 kDa	1	1	0.9	0.9	0.8	0.9	1.3	1.3	0	0
Q9UH62	Armadillo repeat-containing X-linked protein 3	ARMCX3	43 kDa	1	1	0.8	0.8	0.8	0.9	1.3	1.3	0	1
E9PC67	ATP-binding cassette sub-family F member 1	ABCF1	60 kDa	1	1	0.7	0.7	0.8	0.9	1.3	1.3	0	0
H0YE76	Activating signal cointegrator 1 complex subunit 1 (Fragme	ASCC1	21 kDa	1	1	1.2	0.9	1	0.8	1.3	1.3	0	0
O15344	Midline-1	MID1	75 kDa	1	1	0.9	0.8	0.9	0.8	1.3	1.3	0	0
Q9NX24	H/ACA ribonucleoprotein complex subunit 2	NHP2	17 kDa	1	1	0.9	0.7	0.9	0.8	1.3	1.3	0	0
Q9NZ52	Cluster of ADP-ribosylation factor-binding protein GGA3	GGA3	78 kDa	No Va	1	No Value	1	No Valu	0.9	No Val	0.7	0	0
A6NHR9	Structural maintenance of chromosomes flexible hinge dom	SMCHD1	226 kDa	1	No Va	0.8	No Value	1	No Value	1.1	No Val	0	0
P48745	Protein NOV homolog	NOV	39 kDa	1	1	0.8	0.8	0.8	0.8	1.3	1.3	3	0
Q15386	Ubiquitin-protein ligase E3C	UBE3C	124 kDa	1	1	0.8	0.8	0.8	0.8	1.3	1.3	0	0
Q6P3X3	Tetratricopeptide repeat protein 27	TTC27	97 kDa	1	1	0.7	0.7	0.8	0.8	1.3	1.3	0	0
O00161	Cluster of Synaptosomal-associated protein 23	SNAP23	23 kDa	1	1	0.9	1	0.7	0.8	1.3	1.3	0	1
O00483	NADH dehydrogenase [ubiquinone] 1 alpha subcomplex su	NDUFA4	9 kDa	1	1	0.5	0.6	0.7	0.8	1.3	1.3	0	1
Q9NXF7	DDB1- and CUL4-associated factor 16	DCAF16	24 kDa	1	1	0.6	0.8	0.9	0.7	1.3	1.3	0	0

Q15738	Sterol-4-alpha-carboxylate 3-dehydrogenase, decarboxylati	NSDHL	42 kDa	1	1	0.5	0.6	0.6	0.7	1.3	1.3	0	1
Q9ULC5	Long-chain-fatty-acid--CoA ligase 5	ACSL5	76 kDa	1	1	1	0.8	0.6	0.6	1.3	1.3	0	1
D6RH31	Nephronectin (Fragment)	NPNT	67 kDa	1	1	0.8	0.8	0.6	0.6	1.3	1.3	1	0
Q8IWZ8	SURP and G-patch domain-containing protein 1	SUGP1	72 kDa	1	1	0.6	0.6	0.6	0.6	1.3	1.3	0	0
Q96HW7	Cluster of Integrator complex subunit 4	INTS4	108 kDa	1	1	1.6	1.6	1.7	1.8	1.2	1.3	0	0
Q9HA77	Cluster of Probable cysteine--tRNA ligase, mitochondrial	CARS2	62 kDa	1	1	1.4	1.3	1.4	1.4	1.2	1.3	0	0
Q8TCT9	Minor histocompatibility antigen H13	HM13	41 kDa	1	1	1.5	1.8	1.3	1.4	1.2	1.3	0	1
F8W031	Uncharacterized protein (Fragment)		29 kDa	1	1	1.2	1.2	1.1	1.1	1.1	1.1		
Q53H96	Pyrroline-5-carboxylate reductase 3	PYCRL	29 kDa	1	1	1.3	1.3	1.2	1.3	1.2	1.3	0	0
O60243	Heparan-sulfate 6-O-sulfotransferase 1	HS6ST1	48 kDa	1	1	1.2	1.3	1.2	1.3	1.2	1.3	2	0
P09668	Pro-cathepsin H	CTSH	37 kDa	1	1	1.3	1.3	0.9	1.3	1.2	1.3	1	0
Q9BVJ7	Dual specificity protein phosphatase 23	DUSP23	17 kDa	1	1	1.4	1.4	1.1	1.2	1.2	1.3	0	0
Q99941	Cyclic AMP-dependent transcription factor ATF-6 beta	ATF6B	77 kDa	1	1	1.5	1.5	1.1	1.1	1.2	1.3	0	1
Q9BW85	Coiled-coil domain-containing protein 94	CCDC94	37 kDa	1	1	1.2	1.3	1.1	1.1	1.2	1.3	0	0
P51511	Matrix metalloproteinase-15	MMP15	76 kDa	1	1	1.1	1.2	1.1	1.1	1.2	1.3	0	1
Q6P9B6	Cluster of TLD domain-containing protein KIAA1609	TLDC1	51 kDa	1	1	1	1	1.1	1.1	1.2	1.3	0	0
Q9Y2P8	RNA 3'-terminal phosphate cyclase-like protein	RCL1	41 kDa	1	1	0.9	1	1	1.1	1.2	1.3	0	0
Q9NVH2	Integrator complex subunit 7	INTS7	107 kDa	1	1	0.8	0.9	0.9	1.1	1.2	1.3	0	0
E9PN47	Cluster of Probable aminopeptidase NPEPL1	NPEPL1	53 kDa	1	No Va	1.1	No Value	0.9	No Value	1	No Val	0	0
Q9BZI7	Regulator of nonsense transcripts 3B	UPF3B	58 kDa	1	1	0.9	1.1	0.7	1.1	1.2	1.3	0	0
Q9BY77	Isoform 2 of Polymerase delta-interacting protein 3	POLDIP3	43 kDa	1	1	1.2	1.3	1.1	1	1.2	1.3	0	0
Q8TBB5	Kelch domain-containing protein 4	KLHDC4	58 kDa	1	1	1.2	1.1	1	1	1.2	1.3	0	0
P11279	Lysosome-associated membrane glycoprotein 1	LAMP1	45 kDa	1	1	1.1	1.1	1	1	1.2	1.3	0	1
E7ETV8	Importin-5 (Fragment)	IPO5	14 kDa	1	1	1.1	0.8	1.3	1.2	0.5	0.5	0	0
P12644	Bone morphogenetic protein 4	BMP4	47 kDa	1	1	0.9	1	0.9	1	1.2	1.3	1	0
Q8IUR7	Cluster of Isoform 2 of Armadillo repeat-containing protein	ARMC8	74 kDa	1	1	1	0.9	0.9	1	1.2	1.3	0	0
H0YBT8	Cluster of Transforming acidic coiled-coil-containing protei	TACC1	51 kDa	1	1	1	0.8	0.9	1	1.2	1.3	0	0
Q9NV88	Cluster of Integrator complex subunit 9	INTS9	74 kDa	1	1	1	0.9	0.8	1	1.2	1.3	0	0
Q9UK59	Lariat debranching enzyme	DBR1	62 kDa	1	1	0.9	0.9	0.9	0.9	1.2	1.3	0	0
Q86SZ2	Trafficking protein particle complex subunit 6B	TRAPPC6B	18 kDa	1	1	0.9	0.9	0.9	0.9	1.2	1.3	0	0
C9JAB2	Cluster of Serine/arginine-rich-splicing factor 7	SRSF7	27 kDa	1	1	0.9	0.9	0.9	0.9	1.2	1.3	0	0
Q9Y4W6	AFG3-like protein 2	AFG3L2	89 kDa	1	1	0.8	0.9	0.9	0.9	1.2	1.3	0	1
Q9H270	Vacuolar protein sorting-associated protein 11 homolog	VPS11	108 kDa	1	1	1.2	1.6	0.8	0.9	1.2	1.3	0	0
O95155	Ubiquitin conjugation factor E4 B	UBE4B	146 kDa	1	1	1	1	0.9	0.8	1.2	1.3	0	0
G3XAN8	Mitochondrial import inner membrane translocase subunit T	TIMM8B	11 kDa	1	1	0.8	0.9	0.9	0.8	1.2	1.3	0	0
P01266	Thyroglobulin	TG	305 kDa	1	1	1	1	0.8	0.8	1.2	1.3	1	0
Q14160	Cluster of Isoform 3 of Protein scribble homolog	SCRIB	178 kDa	1	1	0.9	0.9	0.8	0.8	1.2	1.3	0	0

O00625	Pirin	PIR	32 kDa	1	1	0.8	0.8	0.8	0.8	1.2	1.3	0	0
O43909	Exostosin-like 3	EXTL3	105 kDa	1	1	0.8	0.8	0.8	0.8	1.2	1.3	0	1
Q96ES7	SAGA-associated factor 29 homolog	CCDC101	33 kDa	1	1	0.8	0.7	0.8	0.8	1.2	1.3	0	0
Q13433	Zinc transporter ZIP6	SLC39A6	85 kDa	1	1	0.8	0.5	0.9	0.7	1.2	1.3	0	1
Q12913	Receptor-type tyrosine-protein phosphatase eta	PTPRJ	146 kDa	1	1	1	1	0.7	1	0.9	1.1	1	1
P51784	Ubiquitin carboxyl-terminal hydrolase 11	USP11	110 kDa	1	1	1.8	1.4	2.4	1.6	1.1	1.3	1	0
Q14114	Cluster of Low-density lipoprotein receptor-related protein	LRP8	106 kDa	1	1	1.2	1.3	1.1	1.3	1.1	1.3	1	1
O43708	Maleylacetoacetate isomerase	GSTZ1	24 kDa	1	1	1	1.3	1.2	1.2	1.1	1.3	0	0
Q9Y3B9	RRP15-like protein	RRP15	31 kDa	1	1	1	1.2	1	1.2	1.1	1.3	0	0
F5H4L0	Cluster of Gamma-tubulin complex component 2	TUBGCP2	106 kDa	1	1	0.9	1.2	1	1.2	1.1	1.3	0	0
B4DIS3	Dpy-30-like protein, isoform CRA_b	LOC84661	14 kDa	1	1	0.9	1.1	1.2	1.1	1.1	1.3		
F5H8D7	DNA repair protein XRCC1	XRCC1	66 kDa	1	1	1.1	0.9	1	1.1	1.1	1.3	0	0
Q9BRP1	Programmed cell death protein 2-like	PDCD2L	39 kDa	1	1	1	1.2	0.9	1.1	1.1	1.3	0	0
Q9UDY8	Mucosa-associated lymphoid tissue lymphoma translocatior	MALT1	92 kDa	1	1	0.7	1.1	0.9	1.1	1.1	1.3	0	0
B1AK64	Chromatin modification-related protein MEAF6	MEAF6	22 kDa	1	1	0.6	0.6	1	1	1.1	1.3	0	0
B5MD23	Tetraspanin-9	TSPAN9	30 kDa	1	1	0.9	1	0.9	1	1.1	1.3	0	1
P56385	ATP synthase subunit e, mitochondrial	ATP5I	8 kDa	1	1	0.9	1.1	0.8	1	1.1	1.3	0	0
Q6H3X3	Cluster of Retinoic acid early transcript 1G protein	RAET1G	37 kDa	1	1	0.9	1.2	1.1	0.9	1.1	1.3	1	1
Q15388	Mitochondrial import receptor subunit TOM20 homolog	TOMM20	16 kDa	1	1	0.7	0.9	0.9	0.9	1.1	1.3	0	1
O95273	Cluster of Cyclin-D1-binding protein 1	CCNDBP1	40 kDa	1	1	1.1	1	0.7	0.9	1.1	1.3	1	0
P61225	Cluster of Ras-related protein Rap-2b	RAP2B	21 kDa	1	1	1.1	1.2	1.7	1.5	1	1.3	0	0
Q5XPI4	Cluster of E3 ubiquitin-protein ligase RNF123	RNF123	149 kDa	1	1	1.6	1.4	1.2	1.5	1	1.3	0	0
P53597	Cluster of Succinyl-CoA ligase [ADP/GDP-forming] subun	SUCLG1	36 kDa	1	1	1.6	1.7	1.4	1.3	1	1.3	1	0
Q8NFD5	AT-rich interactive domain-containing protein 1B	ARID1B	236 kDa	1	1	1	1.5	0.8	1.1	1	1.3	0	0
P78324	Cluster of Isoform 4 of Tyrosine-protein phosphatase non-re	SIRPA	55 kDa	No Va	1	No Value	0.7	No Value	0.7	No Val	0.5	0	1
Q9H0E9	Cluster of Isoform 3 of Bromodomain-containing protein 8	BRD8	81 kDa	No Va	1	No Value	1	No Value	0.9	No Val	1	0	0
Q13724	Cluster of Mannosyl-oligosaccharide glucosidase	MOGS	92 kDa	1	1	1.7	1.1	1.5	0.9	1	1.3	0	1
O00422	Cluster of Histone deacetylase complex subunit SAP18	SAP18	18 kDa	1	1	0.6	0.7	0.8	0.9	1	1.3	0	0
Q13951	Core-binding factor subunit beta	CBFB	22 kDa	1	1	0.7	0.9	0.8	0.8	1	1.3	0	0
Q9NX62	Inositol monophosphatase 3	IMPAD1	39 kDa	1	1	0.8	0.8	1.1	1.1	0.9	1.3	0	1
Q9NZR2	Low-density lipoprotein receptor-related protein 1B	LRP1B	515 kDa	1	1	0.7	1.1	0.9	1	0.9	1.3	1	1
E9PI88	GDP-mannose 4,6 dehydratase	GMDS	39 kDa	1	1	1	1.1	0.8	1	0.9	1.3	0	0
Q7L5D6	Cluster of Golgi to ER traffic protein 4 homolog	GET4	37 kDa	1	No Va	1.2	No Value	1.3	No Value	0.8	No Val	0	0
Q9UK61	Cluster of Protein FAM208A	FAM208A	189 kDa	1	No Va	0.5	No Value	0.9	No Value	1.3	No Val	0	0
H3BRR0	Cluster of Maspardin	SPG21	17 kDa	1	1	0.7	0.8	0.7	0.9	0.9	0.9	0	0
Q5TA95	Tetratricopeptide repeat domain 4	TTC4	46 kDa	1	1	0.7	0.8	0.8	0.8	0.9	1.3	0	0
Q5VV41	Rho guanine nucleotide exchange factor 16	ARHGEF16	80 kDa	No Va	1	No Value	1.3	No Value	1.3	No Val	1.3	0	0

Q96S44	TP53-regulating kinase	TP53RK	28 kDa	1	1	1.2	1.3	1.1	0.7	0.9	1.3	0	0
E9PH42	Receptor-type tyrosine-protein phosphatase U	PTPRU	162 kDa	1	1	0.7	0.6	0.5	0.7	0.9	1.3	0	1
G3V1L6	Chromosome 20 open reading frame 42, isoform CRA_c	FERMT1	48 kDa	1	1	1.2	1.8	1.5	1.5	0.8	1.3	0	0
P60468	Protein transport protein Sec61 subunit beta	SEC61B	10 kDa	1	1	1.1	1.8	1	1.3	0.8	1.3	0	1
Q96GM5	SWI/SNF-related matrix-associated actin-dependent regulat	SMARCD1	58 kDa	1	1	0.8	0.8	1	1.2	0.8	1.3	0	0
Q9Y508	RING finger protein 114	RNF114	26 kDa	1	1	0.9	0.9	1.1	1.1	0.8	1.3	0	0
Q6XZF7	Dynamin-binding protein	DNMBP	177 kDa	1	1	0.9	0.9	0.9	1	2.2	1.4	0	0
J3KPU0	[Pyruvate dehydrogenase [acetyl-transferring]]-phosphatase	PDP1	64 kDa	1	1	0.9	0.8	0.8	0.6	2	1.4	0	0
J3KPV7	Sulfurtransferase	MPST	35 kDa	1	1	0.6	1	0.7	0.7	1.9	1.4	0	0
O75695	Protein XRP2	RP2	40 kDa	1	1	1.3	1.2	1.3	1.3	1.8	1.4	0	0
Q8WUA2	Peptidyl-prolyl cis-trans isomerase-like 4	PPIL4	57 kDa	1	1	0.9	0.9	1	1	1.8	1.4	0	0
Q6RW13	Cluster of Type-1 angiotensin II receptor-associated protein	AGTRAP	17 kDa	1	1	1.1	1.1	1.3	0.9	1.8	1.4	1	1
E9PSI1	Cluster of Transmembrane 9 superfamily member 1	TM9SF1	92 kDa	1	1	1	1	0.9	0.7	1.8	1.4	1	1
P32320	Cytidine deaminase	CDA	16 kDa	1	1	1.2	1.1	1.5	1.3	1.7	1.4	0	0
O00220	Tumor necrosis factor receptor superfamily member 10A	TNFRSF10A	50 kDa	1	1	0.7	1.1	1.4	1	1.7	1.4	0	1
P23560	Brain-derived neurotrophic factor	BDNF	28 kDa	1	1	1	0.9	1.1	1	1.7	1.4	1	1
H0YD97	Cluster of Pyruvate dehydrogenase protein X component, m	PDHX	21 kDa	No Va	No Va	No Value	No Value	No Value	No Value	No Val	No Val	0	0
Q9Y5B0	Cluster of RNA polymerase II subunit A C-terminal domain	CTDP1	104 kDa	No Va	No Va	No Value	No Value	No Value	No Value	No Val	No Val	0	0
J3QS62	CDK5 regulatory subunit-associated protein 3 (Fragment)	CDK5RAP3	23 kDa	1	1	1	0.9	0.9	0.9	1.7	1.4	0	0
G5E955	HCG27698, isoform CRA_c	DDX47	45 kDa	1	1	0.8	0.9	0.8	0.8	1.7	1.4	0	0
Q9Y3C8	Ubiquitin-fold modifier-conjugating enzyme 1	UFC1	19 kDa	1	1	1.9	1.6	1.7	1.4	1.6	1.4	0	0
Q13093	Platelet-activating factor acetylhydrolase	PLA2G7	50 kDa	1	1	1.4	1.3	1.3	1.3	1.6	1.4	1	0
C9JP52	TBC1 domain family member 5	TBC1D5	91 kDa	1	1	0.9	0.9	1.2	1	1.6	1.4	0	1
B7WP27	Pre-mRNA-splicing factor CWC22 homolog	CWC22	106 kDa	1	1	0.7	0.8	0.8	0.9	1.6	1.4	0	0
Q7L3B6	Hsp90 co-chaperone Cdc37-like 1	CDC37L1	39 kDa	1	1	0.9	0.9	0.9	0.8	1.6	1.4	0	0
F5H293	Seizure 6-like protein 2	SEZ6L2	94 kDa	1	1	0.9	0.7	0.9	0.8	1.6	1.4	1	1
Q5TA45	Cluster of Integrator complex subunit 11	CPSF3L	68 kDa	1	1	1.6	1.7	1.3	1.5	1.5	1.4	0	0
Q8N9R8	Cluster of Protein SCAI	SCAI	70 kDa	1	1	1.5	1.5	1.4	1.3	1.5	1.4	0	0
F8W6G1	Nuclear receptor-binding protein	NRBP1	61 kDa	1	1	1.2	1.2	1.1	1.2	1.5	1.4	0	0
B4DJA5	Ras-related protein Rab-5A	RAB5A	22 kDa	1	1	1.2	1.2	1.1	1.2	1.5	1.4	0	0
H7C4F1	Cluster of Arf-GAP with GTPase, ANK repeat and PH dom	AGAP3	21 kDa	1	1	1.1	1	1.1	1.1	1.5	1.4	0	0
B8ZZY2	Arf-GAP domain and FG repeat-containing protein 1	AGFG1	56 kDa	1	1	1	1	1	1.1	1.5	1.4	0	0
P15151	Poliovirus receptor	PVR	45 kDa	1	1	1.3	1.2	1.1	1	1.5	1.4	1	1
J3KQV0	Cyclin-dependent kinase inhibitor 1	CDKN1A	22 kDa	1	1	1.3	1.2	1	1	1.5	1.4	0	0
O00442	RNA 3'-terminal phosphate cyclase	RTCA	39 kDa	1	1	0.9	0.9	1	1	1.5	1.4	0	0
P03973	Antileukoproteinase	SLPI	14 kDa	1	1	0.9	0.9	1	1	1.5	1.4	3	0
Q15043	Zinc transporter ZIP14	SLC39A14	54 kDa	1	1	0.8	0.8	0.9	1	1.5	1.4	1	1

Q9H2A7	C-X-C motif chemokine 16	CXCL16	28 kDa	1	1	1	0.7	1.2	0.9	1.5	1.4	0	1
O14976	Cyclin-G-associated kinase	GAK	143 kDa	1	1	1.1	1.1	1.1	0.9	1.5	1.4	0	0
Q9NRV9	Cluster of Heme-binding protein 1	HEBP1	21 kDa	1	1	1.3	1.2	1	0.9	1.5	1.4	0	0
H0YN81	Cluster of WD repeat-containing protein 61 (Fragment)	WDR61	32 kDa	1	1	1.2	1.1	0.9	0.9	1.5	1.4	0	0
C9JNV2	Cluster of Protein BUD31 homolog	BUD31	14 kDa	1	1	1	1	0.9	0.9	1.5	1.4	0	0
Q92804	TATA-binding protein-associated factor 2N	TAF15	62 kDa	1	1	1	0.9	0.9	0.9	1.5	1.4	0	1
O00116	Alkyldihydroxyacetonephosphate synthase, peroxisomal	AGPS	73 kDa	1	1	0.9	0.9	0.9	0.9	1.5	1.4	1	0
Q6VY07	Cluster of Phosphofurin acidic cluster sorting protein 1	PACS1	105 kDa	1	1	0.8	0.7	0.9	0.9	1.5	1.4	0	0
Q9NPJ6	Mediator of RNA polymerase II transcription subunit 4	MED4	30 kDa	1	1	1	0.8	1	0.8	1.5	1.4	0	0
Q92882	Osteoclast-stimulating factor 1	OSTF1	24 kDa	1	1	0.9	0.9	0.9	0.8	1.5	1.4	0	0
H0Y626	Uncharacterized protein		108 kDa	1	1	0.8	0.7	0.9	0.8	1.5	1.4		
Q01995	Cluster of Transgelin	TAGLN	23 kDa	1	1	0.7	0.7	0.9	0.8	1.5	1.4	0	0
Q9BYN0	Sulfiredoxin-1	SRXN1	14 kDa	1	1	0.9	0.8	0.8	0.7	1.5	1.4	0	0
J3KMX3	Alpha-fetoprotein	AFP	70 kDa	1	1	0.6	0.5	0.8	0.7	1.5	1.4	1	0
E7EQY1	Cluster of Protein FAM136A	FAM136A	27 kDa	1	1	0.7	0.6	0.6	0.7	1.5	1.4	0	0
Q8NEB9	Phosphatidylinositol 3-kinase catalytic subunit type 3	PIK3C3	102 kDa	1	1	2	1.8	2	2	1.4	1.4	0	0
J3KR58	Selenide, water dikinase 2	SEPHS2	51 kDa	1	1	1.4	1.4	1.4	1.4	1.4	1.4	0	0
O75044	Cluster of SLIT-ROBO Rho GTPase-activating protein 2	SRGAP2	121 kDa	1	1	1.2	1.2	1.4	1.4	1.4	1.4	0	0
Q13418	Cluster of Integrin-linked protein kinase	ILK	51 kDa	1	1	1.6	1.4	1.5	1.3	1.4	1.4	0	0
Q86VX2	Isoform 2 of COMM domain-containing protein 7	COMMD7	22 kDa	1	1	1.1	1.2	1.3	1.3	1.4	1.4	0	1
P41273	Tumor necrosis factor ligand superfamily member 9	TNFSF9	27 kDa	1	1	1.2	1.3	1.6	1.2	1.4	1.4	0	0
F5GZJ1	Condensin complex subunit 1	NCAPD2	152 kDa	1	No Va	1.3	No Value	1.1	No Value	0.9	No Val	0	0
O60231	Putative pre-mRNA-splicing factor ATP-dependent RNA he	DHX16	119 kDa	1	1	1.2	1.2	1.3	1.2	1.4	1.4	0	0
P52758	Cluster of Ribonuclease UK114	HRSP12	14 kDa	1	1	1.4	1.4	1	1.2	1.4	1.4	1	0
H7C5G1	Isoamyl acetate-hydrolyzing esterase 1 homolog (Fragment)	IAH1	25 kDa	1	1	1.2	1	1.3	1.1	1.4	1.4	0	0
Q16539	Mitogen-activated protein kinase 14	MAPK14	41 kDa	1	1	1.3	1.3	1.1	1.1	1.4	1.4	0	0
E9PBL8	Conserved oligomeric Golgi complex subunit 1 (Fragment)	COG1	107 kDa	1	1	1.2	1.3	1.1	1.1	1.4	1.4	0	0
Q9H4A6	Golgi phosphoprotein 3	GOLPH3	34 kDa	1	1	1.3	1.2	1.1	1.1	1.4	1.4	0	0
Q58WW2	Isoform 3 of DDB1- and CUL4-associated factor 6	DCAF6	107 kDa	1	1	1.2	1.2	1.1	1.1	1.4	1.4	0	0
O15118	Cluster of Niemann-Pick C1 protein	NPC1	142 kDa	1	No Va	1.2	No Value	0.7	No Value	1.2	No Val	0	1
Q9NZ01	Trans-2,3-enoyl-CoA reductase	TECR	36 kDa	No V	1	No Value	1	No Value	1	No Val	0.6	0	1
Q9UGP8	Translocation protein SEC63 homolog	SEC63	88 kDa	1	1	1.5	1.3	1.5	1.6	0.8	0.8	0	1
P15169	Carboxypeptidase N catalytic chain	CPN1	52 kDa	No V	1	No Value	1	No Value	1.4	No Val	0.9	1	0
Q14692	Ribosome biogenesis protein BMS1 homolog	BMS1	146 kDa	1	1	1	0.9	1	1.1	1.4	1.4	0	0
Q15047	Histone-lysine N-methyltransferase SETDB1	SETDB1	143 kDa	1	1	0.8	0.8	0.8	1.1	1.4	1.4	0	1
Q14690	Protein RRP5 homolog	PDCD11	209 kDa	1	1	1	1	1	1	1.4	1.4	0	0
E9PL17	Polyribonucleotide 5'-hydroxyl-kinase Clp1	CLP1	49 kDa	1	1	0.9	0.8	1	1	1.4	1.4	0	0

Q9UBV8	Peflin	PEF1	30 kDa	1	1	1.1	1.1	0.9	1	1.4	1.4	0	0
P42574	Cluster of Caspase-3	CASP3	32 kDa	1	1	1.1	1.1	0.9	1	1.4	1.4	0	0
Q9Y237	Cluster of Isoform 2 of Peptidyl-prolyl cis-trans isomerase N	PIN4	17 kDa	1	1	1	0.9	0.9	1	1.4	1.4	0	0
Q9Y371	Endophilin-B1	SH3GLB1	41 kDa	1	1	1	0.9	0.9	1	1.4	1.4	0	0
E7ESJ7	Protein FAM114A2	FAM114A2	48 kDa	1	1	1.1	0.9	1	0.9	1.4	1.4	0	0
J3KPZ4	Nuclear nucleic acid-binding protein C1D	C1D	20 kDa	1	1	0.9	0.8	1	0.9	1.4	1.4	0	0
P00742	Coagulation factor X	F10	55 kDa	1	1	1	1	0.9	0.9	1.4	1.4	1	0
Q14165	Cluster of Malectin	MLEC	32 kDa	1	1	0.9	1	0.9	0.9	1.4	1.4	1	1
C9JYY7	Cluster of SEC14-like protein 2 (Fragment)	SEC14L2	10 kDa	1	1	1	1.1	0.7	0.8	1.4	1.4	0	0
O60244	Mediator of RNA polymerase II transcription subunit 14	MED14	161 kDa	1	1	0.8	0.8	0.7	0.8	1.4	1.4	0	0
P07357	Complement component C8 alpha chain	C8A	65 kDa	1	1	0.7	0.7	0.6	0.7	1.4	1.4	3	0
P41271	Isoform 2 of Neuroblastoma suppressor of tumorigenicity 1	NBL1	23 kDa	1	1	1.7	1.5	1.7	1.5	1.3	1.4	1	0
Q9NZM3	Cluster of Isoform 3 of Intersectin-2	ITSN2	142 kDa	No Va	No Va	No Value	No Value	No Value	No Value	No Val	No Val	0	0
P31937	Cluster of 3-hydroxyisobutyrate dehydrogenase, mitochond	HIBADH	35 kDa	1	1	1.2	1.5	1.1	1.4	1.3	1.4	1	0
Q05519	Cluster of Isoform 2 of Serine/arginine-rich splicing factor 1	SRSF11	53 kDa	1	1	1.1	1.2	0.9	1.4	1.3	1.4	0	0
P02649	Cluster of Apolipoprotein E	APOE	36 kDa	1	1	1.6	1.4	1.4	1.3	1.3	1.4	1	0
P23469	Cluster of Receptor-type tyrosine-protein phosphatase epsil	PTPRE	81 kDa	1	1	1.4	1.4	1.3	1.3	1.3	1.4	0	1
Q5TBA9	Protein furry homolog	FRY	339 kDa	1	1	1.3	1.4	1.2	1.3	1.3	1.4	0	0
Q9ULR0	Cluster of Pre-mRNA-splicing factor ISY1 homolog	ISY1	33 kDa	1	1	0.9	1	1	1.3	1.3	1.4	0	0
J3KRC4	Cluster of 5'(3')-deoxyribonucleotidase, cytosolic type	NT5C	20 kDa	1	1	1.1	1.2	1.3	1.2	1.3	1.4	0	0
J3KNI9	TBC1 domain family member 15	TBC1D15	79 kDa	1	1	1.2	1.4	1.1	1.2	1.3	1.4	0	0
Q06546	GA-binding protein alpha chain	GABPA	51 kDa	1	1	1.3	1.3	1.1	1.2	1.3	1.4	0	0
Q07812	Apoptosis regulator BAX	BAX	21 kDa	1	1	1.1	1.1	1.1	1.1	1.3	1.4	0	0
Q7Z7A3	Cytoplasmic tRNA 2-thiolation protein 1	CTU1	36 kDa	1	1	1.1	1.1	1.1	1.1	1.3	1.4	0	0
Q9C0C2	182 kDa tankyrase-1-binding protein	TNKS1BP1	182 kDa	1	1	1	1.1	1.1	1.1	1.3	1.4	0	0
Q9H9K5	HERV-MER_4q12 provirus ancestral Env polyprotein	ERVMER34-1	64 kDa	1	1	1.1	1	1	1.1	1.3	1.4	0	1
Q9UI10	Translation initiation factor eIF-2B subunit delta	EIF2B4	58 kDa	1	1	1	0.9	1.3	1	1.3	1.4	0	0
Q9Y3T9	Nucleolar complex protein 2 homolog	NOC2L	85 kDa	1	1	1.4	1.5	1	1	1.3	1.4	0	0
Q9H3T3	Semaphorin-6B	SEMA6B	95 kDa	1	1	0.9	1	1	1	1.3	1.4	0	1
Q9NZ45	CDGSH iron-sulfur domain-containing protein 1	CISD1	12 kDa	1	1	0.7	0.7	1	1	1.3	1.4	0	1
G3V1P3	Loss of heterozygosity 12 chromosomal region 1 protein	LOH12CR1	20 kDa	1	1	0.9	0.9	0.9	1	1.3	1.4	0	0
Q96CT7	Coiled-coil domain-containing protein 124	CCDC124	26 kDa	1	1	0.8	0.9	1	0.9	1.3	1.4	0	0
Q99574	Neuroserpin	SERPINI1	46 kDa	1	1	0.8	0.9	0.9	0.9	1.3	1.4	1	0
Q9H446	RWD domain-containing protein 1	RWDD1	28 kDa	1	1	1	1.1	0.8	0.9	1.3	1.4	0	0
Q13501	Sequestosome-1	SQSTM1	48 kDa	1	1	0.9	0.9	0.8	0.9	1.3	1.4	0	0
E5RFU2	Bifunctional epoxide hydrolase 2	EPHX2	59 kDa	1	1	0.9	0.9	0.8	0.8	1.3	1.4	0	0
Q9UBG0	C-type mannose receptor 2	MRC2	167 kDa	1	1	0.7	0.7	0.7	0.8	1.3	1.4	0	1

Q9NXV6	CDKN2A-interacting protein	CDKN2AIP	61 kDa	1	1	0.6	0.6	0.6	0.6	1.3	1.4	0	0
P13716	Isoform 2 of Delta-aminolevulinic acid dehydratase	ALAD	39 kDa	1	1	1.5	1.5	1.4	1.5	1.2	1.4	0	0
Q9UHD2	Serine/threonine-protein kinase TBK1	TBK1	84 kDa	1	1	1.4	1.4	1.4	1.5	1.2	1.4	0	0
Q8NBL1	Protein O-glucosyltransferase 1	POGLUT1	46 kDa	1	1	1.3	1.3	1.3	1.3	1.2	1.4	1	0
Q9Y657	Spindlin-1	SPIN1	30 kDa	1	1	1.1	1.1	1.2	1.3	1.2	1.4	0	0
Q0PNE2	Elongator complex protein 6	ELP6	30 kDa	1	1	1	1.3	1	1.2	1.2	1.4	0	1
Q9NRA1	Cluster of Platelet-derived growth factor C	PDGFC	39 kDa	1	1	0.9	1.1	1	1.4	0.7	1	1	0
Q15018	BRISC complex subunit Abro1	FAM175B	47 kDa	1	1	1	0.9	1.6	1.1	1.2	1.4	0	0
J3KMX1	Cluster of Nuclear pore complex protein Nup88 (Fragment)	NUP88	79 kDa	1	1	1.1	0.9	1.1	1.1	1.2	1.4	0	0
Q9P253	Vacuolar protein sorting-associated protein 18 homolog	VPS18	110 kDa	1	1	0.8	0.9	1.1	1.1	1.2	1.4	0	0
Q9C0I1	Myotubularin-related protein 12	MTMR12	86 kDa	1	1	1	1.2	0.9	1.1	1.2	1.4	0	0
O14763	Tumor necrosis factor receptor superfamily member 10B	TNFRSF10B	48 kDa	1	1	0.9	1.2	1.2	1	1.2	1.4	0	1
Q9H944	Cluster of Mediator of RNA polymerase II transcription sub	MED20	23 kDa	1	1	0.9	1	0.9	0.9	1.2	1.4	1	0
Q9NQT5	Exosome complex component RRP40	EXOSC3	30 kDa	1	1	0.7	0.8	0.8	0.9	1.2	1.4	0	0
Q9Y314	Nitric oxide synthase-interacting protein	NOSIP	33 kDa	1	1	1.5	1.6	1.1	1.5	1.1	1.4	0	0
B4DFL0	Nucleolar protein 3	NOL3	29 kDa	1	1	1.2	0.8	1.1	0.7	1.1	0.9	0	0
E9PDI2	Adenylyl cyclase-associated protein	CAP2	50 kDa	1	1	0.9	1	0.9	1.1	1.1	1.4	0	0
O95336	6-phosphogluconolactonase	PGLS	28 kDa	1	1	0.8	0.8	0.9	1	1.1	1.4	0	0
H3BPJ9	Cluster of NADH dehydrogenase [ubiquinone] 1 beta subco	NDUFB10	19 kDa	1	1	0.9	0.7	0.8	0.8	1.1	1.4	0	0
O95295	SNARE-associated protein Snapin	SNAPIN	15 kDa	1	1	0.6	0.9	0.6	0.6	1.1	1.4	0	0
Q8NFH3	Nucleoporin Nup43	NUP43	42 kDa	1	1	0.9	1.1	1.1	1	0.7	0.8	0	0
Q8WXD5	Gem-associated protein 6	GEMIN6	19 kDa	1	1	0.9	1.3	1.3	1.2	1	1.4	0	0
D6REB5	Alpha-L-iduronidase	IDUA	58 kDa	1	1	0.7	0.7	0.7	0.9	1	1.4	1	0
O75494	Cluster of Serine/arginine-rich splicing factor 10	SRSF10	31 kDa	1	1	0.9	0.9	0.9	1.1	0.9	1.4	0	0
F5GY80	Complement component C8 beta chain	C8B	60 kDa	1	1	1.2	1.3	1	1	0.9	1.4	1	0
H0Y8V2	Extracellular matrix protein FRAS1 (Fragment)	FRAS1	47 kDa	1	1	0.9	0.9	0.9	0.9	0.7	0.7	1	1
Q12846	Syntaxin-4	STX4	34 kDa	1	1	1.2	1.2	1.1	1.5	0.9	1.1	0	1
Q6EMK4	Vasorin	VASN	72 kDa	1	1	1.2	0.7	0.8	0.8	1.4	1	0	1
H7BZ14	Cluster of Peptidyl-prolyl cis-trans isomerase (Fragment)	PPIL3	20 kDa	1	1	1	1	1.1	0.8	1.4	1.1	0	1
Q5JSH3	Cluster of WD repeat-containing protein 44	WDR44	101 kDa	1	1	1.1	1.2	0.9	1.1	0.7	0.8	0	0
O76074	Isoform PDE5A2 of cGMP-specific 3',5'-cyclic phosphodie:	PDE5A	95 kDa	1	1	0.6	0.6	0.8	0.8	0.8	0.7	0	0
Q9UBB5	Methyl-CpG-binding domain protein 2	MBD2	43 kDa	1	1	1	1	1	0.9	2	1.5	0	0
Q9Y315	Putative deoxyribose-phosphate aldolase	DERA	35 kDa	1	1	1	1	1.4	1	1.9	1.5	0	0
O43353	Cluster of Receptor-interacting serine/threonine-protein kin	RIPK2	61 kDa	1	1	1	0.9	0.9	0.8	1.9	1.5	0	0
O95628	Cluster of Isoform 6 of CCR4-NOT transcription complex s	CNOT4	85 kDa	1	1	1.3	1.2	1.5	1.4	1.8	1.5	0	0
Q16186	Proteasomal ubiquitin receptor ADRM1	ADRM1	42 kDa	1	1	1.9	1.7	1.2	1.3	1.8	1.5	0	0
Q9Y6R7	IgGFc-binding protein	FCGBP	572 kDa	1	1	0.8	0.9	1.2	1.1	1.8	1.5	1	0

Q12974	Cluster of Protein tyrosine phosphatase type IVA 2	PTP4A2	19 kDa	1	1	1	0.9	1.1	1.1	1.8	1.5	0	0
Q14108	Lysosome membrane protein 2	SCARB2	54 kDa	1	1	0.8	0.8	1	0.9	1.8	1.5	1	1
Q53GQ0	Estradiol 17-beta-dehydrogenase 12	HSD17B12	34 kDa	1	1	0.7	0.7	0.9	0.6	1.8	1.5	0	1
Q14331	Protein FRG1	FRG1	29 kDa	1	1	0.8	0.9	1.2	1.3	1.7	1.5	0	0
P05114	Cluster of Non-histone chromosomal protein HMG-14	HMGN1	11 kDa	1	1	1.3	1.3	1.4	1.2	1.7	1.5	0	1
P33947	ER lumen protein retaining receptor 2	KDELR2	24 kDa	1	1	1.1	1	1.2	1	1.7	1.5	0	1
O60671	Cell cycle checkpoint protein RAD1	RAD1	32 kDa	1	1	1.1	1.1	1.1	1	1.7	1.5	0	0
G8JLA8	Cluster of Transforming growth factor-beta-induced protein	TGFBI	75 kDa	1	1	1	1	1	1	1.7	1.5	1	0
Q96CW5	Gamma-tubulin complex component 3	TUBGCP3	104 kDa	1	1	1	0.9	1	1	1.7	1.5	0	0
Q13362	Isoform 4 of Serine/threonine-protein phosphatase 2A 56 kI	PPP2R5C	63 kDa	1	1	0.9	0.9	1	1	1.7	1.5	0	0
Q9NWY4	UPF0609 protein C4orf27	C4orf27	39 kDa	1	1	1.1	0.9	1	0.9	1.7	1.5	0	0
Q9Y2H0	Cluster of Isoform 2 of Disks large-associated protein 4	DLGAP4	108 kDa	1	1	1	0.9	0.9	0.9	1.7	1.5	0	0
Q66K14	TBC1 domain family member 9B	TBC1D9B	141 kDa	1	1	0.7	0.7	0.7	0.7	1.7	1.5	0	0
Q7L775	EPM2A-interacting protein 1	EPM2AIP1	70 kDa	1	1	1.3	1.3	1.3	1.4	1.6	1.5	0	0
O95502	Neuronal pentraxin receptor	NPTXR	53 kDa	1	No Va	1.2	No Value	1.1	No Value	1.8	No Val	2	0
O75348	V-type proton ATPase subunit G 1	ATP6V1G1	14 kDa	1	1	1.4	1.5	1.4	1.3	1.6	1.5	0	0
B4DJL6	UPF0464 protein C15orf44	VWA9	61 kDa	1	1	1.5	1.2	1.5	1.2	1.6	1.5	0	1
E9PEQ6	Peptidyl-prolyl cis-trans isomerase	PPIE	28 kDa	1	1	1.4	1.4	1.2	1.2	1.6	1.5	0	0
Q8N1Q1	Carbonic anhydrase 13	CA13	29 kDa	1	1	1.1	1.1	1.1	1.2	1.6	1.5	0	0
Q13131	Isoform 2 of 5'-AMP-activated protein kinase catalytic subu	PRKAA1	66 kDa	1	1	1	1.1	1	1.2	1.6	1.5	0	0
O43286	Beta-1,4-galactosyltransferase 5	B4GALT5	45 kDa	1	1	1	1	1.1	1.1	1.6	1.5	2	0
Q8IYI6	Exocyst complex component 8	EXOC8	82 kDa	1	1	0.8	0.8	1	1.1	1.6	1.5	0	0
Q5T123	SH3 domain binding glutamic acid-rich protein like 3	SH3BGRL3	9 kDa	1	1	1.2	1.3	1.1	1	1.6	1.5	0	0
Q6I9Y2	THO complex subunit 7 homolog	THOC7	24 kDa	1	1	1.2	1.1	1.1	1	1.6	1.5	0	0
Q15363	Transmembrane emp24 domain-containing protein 2	TMED2	23 kDa	1	1	1	1	0.9	1	1.6	1.5	1	1
C9JMP5	Cluster of SOSS complex subunit B1 (Fragment)	NABP2	16 kDa	1	1	1.2	0.9	1	0.9	1.6	1.5	0	0
Q12769	Cluster of Nuclear pore complex protein Nup160	NUP160	162 kDa	1	1	0.8	0.8	0.9	0.9	1.6	1.5	0	0
Q92974	Rho guanine nucleotide exchange factor 2	ARHGEF2	112 kDa	1	1	0.8	0.8	0.9	0.9	1.6	1.5	0	0
Q9NXR1	Cluster of Isoform 2 of Nuclear distribution protein nudE h	NDE1	38 kDa	1	1	0.7	0.8	0.9	0.9	1.6	1.5	0	0
Q99590	Protein SCAF11	SCAF11	165 kDa	1	1	0.7	0.7	0.8	0.9	1.6	1.5	0	0
Q86X83	COMM domain-containing protein 2	COMMD2	23 kDa	1	1	0.9	0.9	0.9	0.8	1.6	1.5	0	0
C9J6P4	Cluster of Zinc finger CCCH-type antiviral protein 1	ZC3HAV1	114 kDa	No Va	No Va	No Value	No Value	No Value	No Value	No Val	No Val	0	0
Q16762	Thiosulfate sulfurtransferase	TST	33 kDa	1	1	0.8	0.8	0.8	0.8	1.6	1.5	0	0
E2QRD5	Protein C15orf38-AP3S2	C15orf38-AP3	44 kDa	1	1	1.2	1	0.7	0.7	1.6	1.5	0	0
P19404	NADH dehydrogenase [ubiquinone] flavoprotein 2, mitoch	NDUFV2	27 kDa	1	1	0.6	0.6	0.4	0.5	1.6	1.5	0	0
A8CTX8	Intersectin 1 long form variant 2	ITSN1	195 kDa	1	1	1.7	1.7	1.5	1.6	1.5	1.5	0	1
Q8WUF5	RelA-associated inhibitor	PPP1R13L	89 kDa	1	1	1.1	1.2	1.4	1.3	1.5	1.5	0	0

Q9P2K2	Thioredoxin domain-containing protein 16	TXNDC16	94 kDa	1	1	1.1	1.2	1.1	1.3	1.5	1.5	0	1
Q9UFC0	Leucine-rich repeat and WD repeat-containing protein 1	LRWD1	71 kDa	1	1	1.2	0.6	0.8	1	1	4.2	0	0
Q9NUW8	Cluster of Tyrosyl-DNA phosphodiesterase 1	TDP1	68 kDa	1	1	1.4	1.4	1.1	1.2	1.5	1.5	0	0
G3XAE6	Extracellular sulfatase Sulf-2	SULF2	98 kDa	1	1	1.1	1.2	1.1	1.1	1.5	1.5	1	0
Q32P41	Cluster of tRNA (guanine(37)-N1)-methyltransferase	TRMT5	58 kDa	1	1	1.2	1.1	1.1	1.1	1.5	1.5	0	0
Q13111	Cluster of Chromatin assembly factor 1 subunit A	CHAF1A	107 kDa	1	1	1	1	1.1	1.1	1.5	1.5	0	0
Q9UHP3	Isoform USP25m of Ubiquitin carboxyl-terminal hydrolase	USP25	130 kDa	1	1	0.8	1.2	1	1.1	1.5	1.5	0	0
E9PHA6	Cluster of DNA mismatch repair protein Msh2	MSH2	103 kDa	1	No Va	1.2	No Value	0.5	No Value	1	No Val	0	0
O75323	Cluster of Protein NipSnap homolog 2	GBAS	34 kDa	1	1	1.3	1.2	0.9	1.1	1.5	1.5	0	0
Q6UW63	KDEL motif-containing protein 1	KDELC1	58 kDa	1	1	1	1	1.1	1	1.5	1.5	3	0
B7Z7B0	Cluster of Cleavage and polyadenylation-specificity factor s	CPSF4	22 kDa	1	1	1.1	1.1	1	1	1.5	1.5	0	0
Q07954	Prolow-density lipoprotein receptor-related protein 1	LRP1	505 kDa	1	1	0.9	1.1	1	1	1.5	1.5	1	1
Q5K651	Sterile alpha motif domain-containing protein 9	SAMD9	184 kDa	1	No Va	0.7	No Value	0.7	No Value	1	No Val	0	0
P18583	Cluster of Isoform A of Protein SON	SON	232 kDa	1	1	0.9	0.9	1	1	1.5	1.5	0	0
H0YH01	A-kinase anchor protein 7 isoform gamma (Fragment)	AKAP7	6 kDa	1	1	0.9	0.9	1	1	1.5	1.5	0	0
Q9H9T3	Elongator complex protein 3	ELP3	62 kDa	1	1	0.8	0.9	1	1	1.5	1.5	0	0
A6NML8	Cluster of Diaphanous homolog 2 (Drosophila), isoform CR	DIAPH2	125 kDa	No Va	No Va	No Value	No Value	No Value	No Value	No Val	No Val	0	0
Q9UJX2	Cluster of Cell division cycle protein 23 homolog	CDC23	69 kDa	No Va	No Va	No Value	No Value	No Value	No Value	No Val	No Val	1	0
Q9Y3C1	Cluster of Nucleolar protein 16	NOP16	21 kDa	1	1	0.9	0.8	1	1	1.5	1.5	0	0
Q8N108	Cluster of Mesoderm induction early response protein 1	MIER1	58 kDa	1	1	0.9	0.9	0.9	1	1.5	1.5	1	0
P29728	2'-5'-oligoadenylate synthase 2	OAS2	82 kDa	1	1	0.9	0.9	0.9	1	1.5	1.5	0	0
B7ZB78	Ras-related protein Rap-1b	RAP1B	14 kDa	1	1	1.3	1	1	0.9	1.5	1.5	0	0
A0FGR8	Cluster of Isoform 2 of Extended synaptotagmin-2	ESYT2	99 kDa	1	1	0.8	0.9	0.9	0.9	1.5	1.5	0	1
P50225	Cluster of Sulfotransferase 1A1	SULT1A1	34 kDa	1	1	0.8	0.8	0.9	0.9	1.5	1.5	0	0
F5H5U7	Cluster of Coiled-coil domain-containing protein 132	CCDC132	108 kDa	1	1	0.7	0.6	0.9	0.9	1.5	1.5	0	0
Q9BZ29	Cluster of Dedicator of cytokinesis protein 9	DOCK9	236 kDa	No Va	No Va	No Value	No Value	No Value	No Value	No Val	No Val	0	0
Q13277	Cluster of Syntaxin-3	STX3	33 kDa	No Va	1	No Value	0.5	No Value	0.6	No Val	1.3	0	1
Q16822	Cluster of Phosphoenolpyruvate carboxykinase [GTP], mitc	PCK2	71 kDa	No Va	1	No Value	0.8	No Value	1	No Val	0.5	0	0
Q14532	Cluster of Keratin, type I cuticular Ha2	KRT32	50 kDa	No Va	1	No Value	1	No Value	0.8	No Val	0.4	0	0
B3KQV7	Equilibrative nucleoside transporter 1	SLC29A1	59 kDa	1	1	0.8	0.8	0.8	0.8	1.5	1.5	0	1
P46937	Yorkie homolog	YAP1	54 kDa	1	1	0.7	0.8	0.7	0.8	1.5	1.5	0	0
Q93052	Lipoma-preferred partner	LPP	66 kDa	1	1	0.6	0.5	0.7	0.6	1.5	1.5	0	0
Q9NX70	Mediator of RNA polymerase II transcription subunit 29	MED29	21 kDa	1	1	0.5	0.5	0.5	0.5	1.5	1.5	0	0
C9JBI3	Phosphoserine phosphatase (Fragment)	PSPH	21 kDa	1	1	1.1	1.1	1.3	1.2	1.4	1.5	0	0
J3KQS6	BRISC and BRCA1-A complex member 1	BABAM1	36 kDa	1	1	1.7	1.3	1.1	1.2	1.4	1.5	0	0
G3V1L6	Isoform 4 of Fermitin family homolog 1	FERMT1	57 kDa	1	1	1	1	1.1	1.2	1.4	1.5	0	0
Q9GZX9	Twisted gastrulation protein homolog 1	TWSG1	25 kDa	1	1	1.1	1.2	1.1	1.1	1.4	1.5	1	0

Q03111	Protein ENL	MLLT1	62 kDa	1	1	1.2	1.1	1.1	1.1	1.4	1.5	0	0
F5GXG0	E3 ubiquitin-protein ligase UHRF1	UHRF1	90 kDa	1	1	1	1	1.1	1.1	1.4	1.5		
P14222	Perforin-1	PRF1	61 kDa	1	1	1	1.1	0.9	1.1	1.4	1.5	1	0
Q96B54	Zinc finger protein 428	ZNF428	20 kDa	1	1	0.9	1	1	1	1.4	1.5	0	0
Q9UBV2	Protein sel-1 homolog 1	SEL1L	89 kDa	1	1	0.8	0.7	1	1	1.4	1.5	1	1
F8VW96	Cysteine and glycine-rich protein 2	CSRP2	27 kDa	1	1	0.9	1	0.9	0.9	1.4	1.5	0	0
Q4G0F5	Vacuolar protein sorting-associated protein 26B	VPS26B	39 kDa	1	1	0.8	1	0.9	0.9	1.4	1.5	0	0
Q04837	Single-stranded DNA-binding protein, mitochondrial	SSBP1	17 kDa	1	1	0.8	0.9	0.9	0.9	1.4	1.5	0	0
G5E934	Cluster of Cancer susceptibility candidate 4, isoform CRA_	CASC4	49 kDa	1	1	0.9	1	0.9	0.8	1.4	1.5	0	0
G3V1U8	CDK-activating kinase assembly factor MAT1	MNAT1	31 kDa	1	1	0.8	0.8	0.8	0.8	1.4	1.5	0	0
Q15172	Serine/threonine-protein phosphatase 2A 56 kDa regulatory	PPP2R5A	56 kDa	1	1	1.6	1.5	1.5	1.4	1.3	1.5	0	0
G3V3F1	Neural cell expressed, developmentally down-regulated 1, i	NEDD1	73 kDa	1	1	1.4	1.4	1.2	1.4	1.3	1.5	0	0
O75818	Ribonuclease P protein subunit p40	RPP40	42 kDa	1	1	0.9	1	1.2	1.4	1.3	1.5	0	0
Q8NFZ4	Cluster of Neuroligin-2	NLGN2	91 kDa	1	1	0.9	0.9	1.1	1.1	1.3	1.5	0	1
P25490	Transcriptional repressor protein YY1	YY1	45 kDa	1	1	1	1.3	1	1.1	1.3	1.5	0	0
Q96S59	Ran-binding protein 9	RANBP9	78 kDa	1	1	1.2	1.1	1	1	1.3	1.5	0	0
O43464	Cluster of Serine protease HTRA2, mitochondrial	HTRA2	49 kDa	1	1	1.2	0.8	1	1	1.3	1.5	0	1
P51693	Amyloid-like protein 1	APLP1	72 kDa	1	1	0.8	0.8	1	0.9	1.3	1.5	1	1
Q9NRD1	F-box only protein 6	FBXO6	34 kDa	1	1	1	1.2	0.9	0.9	1.3	1.5	0	0
B7Z5Z2	Ras-related protein R-Ras2	RRAS2	24 kDa	1	1	1	0.8	0.8	0.9	1.3	1.5	0	0
P50750	Isoform 2 of Cyclin-dependent kinase 9	CDK9	53 kDa	1	1	0.8	0.8	0.9	0.8	1.3	1.5	0	0
Q8NEF9	Serum response factor-binding protein 1	SRFBP1	49 kDa	1	1	1.1	1.3	1	1.2	1.2	1.5	0	0
A8MX29	Kinesin light chain 2	KLC2	37 kDa	1	1	1.1	1.1	0.9	1.1	1.2	1.5	0	0
Q13641	Trophoblast glycoprotein	TPBG	46 kDa	1	1	0.9	1.3	0.9	0.8	1.2	1.5	0	1
O75368	SH3 domain-binding glutamic acid-rich-like protein	SH3BGRL	13 kDa	1	1	0.9	0.7	0.7	0.7	1.2	1.5	0	0
E5RJF8	Centrin-3	CETN3	22 kDa	1	1	1	1	1.1	1	1.1	1.5	0	0
Q9Y3D6	Mitochondrial fission 1 protein	FIS1	17 kDa	1	1	0.6	0.5	0.7	0.5	1.1	1.5	0	1
A6NGP5	Cluster of Hematological and neurological-expressed 1-like	HN1L	19 kDa	1	1	1.6	2.3	1.5	2.4	1	1.5	0	0
I3L291	Uncharacterized protein		59 kDa	1	1	0.7	1.1	0.8	1.2	1	1.5		
P33527	Multidrug resistance-associated protein 1	ABCC1	172 kDa	1	1	0.9	1.1	0.8	1.1	1	1.5	0	1
C9J1Z8	ADP-ribosylation factor 5 (Fragment)	ARF5	17 kDa	1	1	0.6	1	0.7	0.9	1	1.5	0	0
O15127	Cluster of Secretory carrier-associated membrane protein 2	SCAMP2	37 kDa	1	1	1	0.8	1.2	0.9	0.6	0.5	0	1
P48740	Cluster of Mannan-binding lectin serine protease 1	MASP1	79 kDa	No Va	No Va	No Value	No Value	No Value	No Value	No Val	No Val	1	0
Q13371	Phosducin-like protein	PDCL	34 kDa	1	1	1.2	1.7	1	1.7	0.9	0.9	0	0
Q9P2R7	Isoform 2 of Succinyl-CoA ligase [ADP-forming] subunit b	SUCLA2	48 kDa	1	1	1.2	0.8	1.4	1.1	2.6	1.6	0	0
E7EQ61	Ubiquitin-like modifier-activating enzyme 5	UBA5	42 kDa	1	1	1.1	0.8	1.2	1	2	1.6	0	1
A8MUM1	Cluster of Protein TSSC1	TSSC1	46 kDa	1	1	0.7	0.8	0.8	0.9	2	1.6	0	0

Q15208	Cluster of Serine/threonine-protein kinase 38	STK38	54 kDa	1	1	1.3	1	1.3	1.2	1.9	1.6	0	0
Q8NEJ9	Cluster of Neuroguidin	NGDN	36 kDa	1	1	1.1	1	1.2	1.1	1.9	1.6	0	0
Q15554	Telomeric repeat-binding factor 2	TERF2	56 kDa	1	1	0.7	1	1	0.9	1.9	1.6	0	1
J3KR78	Queuine tRNA-ribosyltransferase subunit QTRTD1	QTRTD1	48 kDa	1	1	0.8	0.7	0.9	0.8	1.9	1.6	0	0
P06400	Retinoblastoma-associated protein	RB1	106 kDa	1	1	1.7	1.4	1.8	1.7	1.8	1.6	0	0
Q9H7B2	Ribosome production factor 2 homolog	RPF2	36 kDa	1	1	1.2	1.2	1.2	1.3	1.8	1.6	0	0
H0Y8R1	Cluster of G-rich sequence factor 1 (Fragment)	GRSF1	47 kDa	No Va	No Va	No Value	No Value	No Value	No Value	No Val	No Val	0	0
B7Z3Y2	Prenylcysteine oxidase 1	PCYOX1	48 kDa	1	1	1	1.3	1	1	1.8	1.6	1	0
Q96F24	Nuclear receptor-binding factor 2	NRBF2	32 kDa	1	1	0.8	0.7	1	0.9	1.8	1.6	0	0
Q9HCU4	Cadherin EGF LAG seven-pass G-type receptor 2	CELSR2	317 kDa	1	1	0.8	0.8	0.8	0.8	1.8	1.6	0	1
Q9Y6X9	MORC family CW-type zinc finger protein 2	MORC2	118 kDa	1	1	1.4	1.5	1.6	1.5	1.7	1.6	0	0
H3BRS6	ADP-dependent glucokinase (Fragment)	ADPGK	22 kDa	1	1	1.3	1.3	1.6	1.5	1.7	1.6	1	1
O75683	Surfeit locus protein 6	SURF6	41 kDa	1	1	1.2	1.2	1.4	1.3	1.7	1.6	0	0
P08574	Cytochrome c1, heme protein, mitochondrial	CYC1	35 kDa	1	1	1.1	1.2	1.4	1.3	1.7	1.6	0	1
Q9H5N1	Rab GTPase-binding effector protein 2	RABEP2	64 kDa	1	1	0.8	1	1	1.3	1.7	1.6	0	0
B7Z7A3	Nucleolar GTP-binding protein 1	GTPBP4	68 kDa	No Va	1	No Value	0.9	No Value	1.1	No Val	1.1	0	0
B7Z3D5	Cluster of Myotubularin related protein 1, isoform CRA_a	MTMR1	65 kDa	1	1	1.2	1.3	1.2	1.1	1.7	1.6	0	0
P41240	Tyrosine-protein kinase CSK	CSK	51 kDa	1	1	1.1	1.1	1.1	1.1	1.7	1.6	0	0
Q96G25	Mediator of RNA polymerase II transcription subunit 8	MED8	29 kDa	1	1	0.8	0.8	1.1	1.1	1.7	1.6	0	0
A6NGV6	Aryl hydrocarbon receptor nuclear translocator	ARNT	46 kDa	1	1	0.7	0.7	1.1	1	1.7	1.6	0	0
Q7Z7E8	Ubiquitin-conjugating enzyme E2 Q1	UBE2Q1	46 kDa	1	1	0.9	0.9	1	1	1.7	1.6	0	0
Q99747	Gamma-soluble NSF attachment protein	NAPG	35 kDa	1	1	0.9	0.9	1	0.9	1.7	1.6	0	0
Q96SB3	Neurabin-2	PPP1R9B	89 kDa	1	1	0.9	1.1	0.9	0.9	1.7	1.6	0	0
Q9Y6A4	UPF0468 protein C16orf80	C16orf80	23 kDa	1	1	1.1	0.9	0.9	0.9	1.7	1.6	0	0
Q5NDL2	EGF domain-specific O-linked N-acetylglucosamine transfe	EOGT	62 kDa	1	1	0.9	0.9	0.9	0.9	1.7	1.6	1	0
D6RDX8	Tetraspanin-17	TSPAN17	34 kDa	1	1	0.8	0.6	0.9	0.8	1.7	1.6	0	1
Q9Y5B8	Nucleoside diphosphate kinase 7	NME7	42 kDa	1	1	0.5	0.7	0.8	0.8	1.7	1.6	0	0
P00488	Coagulation factor XIII A chain	F13A1	83 kDa	1	1	0.9	0.9	0.5	0.7	1.7	1.6	0	0
Q96T23	Remodeling and spacing factor 1	RSF1	164 kDa	1	1	1.6	1.6	1.7	1.6	1.6	1.6	1	0
O15400	Syntaxin-7	STX7	30 kDa	1	1	1.5	1.4	1.3	1.4	1.6	1.6	0	1
F5H2A9	Cluster of Very long-chain-specific acyl-CoA dehydrogenas	ACADVL	73 kDa	1	1	0.9	0.9	0.8	0.7	0.7	0.6	0	0
Q5VST3	Cluster of Protein O-linked mannose beta1,2-N-acetylgluco	POMGNT1	85 kDa	1	1	1.1	1	1.3	1.2	1.6	1.6	0	1
Q96B45	UPF0693 protein C10orf32	C10orf32	12 kDa	1	1	1.2	0.9	1.1	1.1	1.6	1.6	0	0
Q9NP77	Cluster of RNA polymerase II subunit A C-terminal domain	SSU72	23 kDa	1	1	1	0.9	1.1	1.1	1.6	1.6	0	0
P49441	Inositol polyphosphate 1-phosphatase	INPP1	44 kDa	1	1	0.9	1.1	1	1.1	1.6	1.6	0	0
Q9NRN7	L-aminoadipate-semialdehyde dehydrogenase-phosphopant	AASDHPPT	36 kDa	1	1	1	1	0.9	1	1.6	1.6	0	0
D6RF48	Syntaxin-18	STX18	35 kDa	1	1	0.9	0.9	0.9	1	1.6	1.6	0	1

Q96A49	Synapse-associated protein 1	SYAP1	40 kDa	1	1	0.8	0.8	0.9	1	1.6	1.6	2	0
E9PRZ9	Protein C11orf58 (Fragment)	C11orf58	10 kDa	1	1	0.9	0.9	1	0.9	1.6	1.6	0	0
Q9BX68	Histidine triad nucleotide-binding protein 2, mitochondrial	HINT2	17 kDa	1	1	0.9	0.8	0.9	0.9	1.6	1.6	2	0
B1AUU8	Cluster of Epidermal growth factor receptor pathway substr	EPS15	84 kDa	1	1	0.8	0.8	0.8	0.9	1.6	1.6	1	0
Q9ULH1	Arf-GAP with SH3 domain, ANK repeat and PH domain-cc	ASAP1	126 kDa	1	1	0.7	0.8	0.7	0.8	1.6	1.6	0	0
G8JLQ3	Biogenesis of lysosome-related organelles complex 1 subun	BLOC1S1	9 kDa	1	1	1.1	0.9	0.8	0.7	1.6	1.6	0	0
Q9H081	Protein MIS12 homolog	MIS12	24 kDa	1	1	0.6	0.7	0.7	0.7	1.6	1.6	0	0
A8MZF9	Cluster of Developmentally-regulated GTP-binding protein	DRG2	38 kDa	1	1	1.5	1.7	2	2.1	1.5	1.6	0	0
Q14376	Cluster of UDP-glucose 4-epimerase	GALE	38 kDa	1	1	1.5	1.6	1.3	1.4	1.5	1.6	0	0
Q9H4E7	Cluster of Differentially expressed in FDCP 6 homolog	DEF6	74 kDa	1	1	1.2	1.2	1.2	1.3	1.5	1.6	0	0
D6RC11	Cluster of Claudin domain-containing protein 1	CLDND1	24 kDa	No Va	No Va	No Value	No Value	No Value	No Value	No Val	No Val	1	1
P01009	Alpha-1-antitrypsin	SERPINA1	47 kDa	1	1	1.1	1.2	1.2	1.2	1.5	1.6	1	0
P21709	Ephrin type-A receptor 1	EPHA1	108 kDa	No Va	1	No Value	0.9	No Value	1	No Val	0.7	0	1
O75554	WW domain-binding protein 4	WBP4	43 kDa	1	1	1.2	1.2	1.1	1.2	1.5	1.6	0	0
J3KQ83	ATP synthase-coupling factor 6, mitochondrial	ATP5J	13 kDa	1	1	0.9	0.9	0.8	1.1	1.5	1.6	0	0
P56381	ATP synthase subunit epsilon, mitochondrial	ATP5E	6 kDa	1	1	0.9	1	1.1	1	1.5	1.6	0	0
Q86Y38	Xylosyltransferase 1	XYLT1	108 kDa	1	1	1	1	0.9	1	1.5	1.6	3	0
E7ER20	Cluster of Proteasome inhibitor PI31 subunit	PSMF1	22 kDa	1	1	0.9	1	0.9	1	1.5	1.6	0	0
Q9NZJ9	Cluster of Diphosphoinositol polyphosphate phosphohydrol	NUDT4	20 kDa	1	1	0.9	0.9	0.9	1	1.5	1.6	0	0
Q9Y217	Myotubularin-related protein 6	MTMR6	72 kDa	1	1	1.1	1.1	0.9	0.9	1.5	1.6	0	0
B1AK40	Cluster of Acylpyruvase FAHD1, mitochondrial	FAHD1	27 kDa	No Va	No Va	No Value	No Value	No Value	No Value	No Val	No Val	0	0
P55196	Cluster of Isoform 2 of Afadin	MLLT4	206 kDa	1	1	0.9	1	0.8	0.9	1.5	1.6	0	0
P48960	CD97 antigen	CD97	92 kDa	1	1	0.5	0.7	0.8	0.8	1.5	1.6	1	1
J3KR54	Isovaleryl Coenzyme A dehydrogenase, isoform CRA_a	IVD	47 kDa	1	1	0.7	0.6	0.8	0.8	1.5	1.6	0	0
F5H7J5	Regulatory-associated protein of mTOR	RPTOR	132 kDa	1	1	0.9	1.1	1.2	1.2	1.4	1.6	0	1
F8WBX1	Rho GTPase-activating protein 27	ARHGAP27	95 kDa	1	1	1	1.1	1	1.1	1.4	1.6	0	0
O95470	Cluster of Sphingosine-1-phosphate lyase 1	SGPL1	64 kDa	1	1	0.9	1.2	1.1	0.9	1.4	1.6	0	1
Q6P4I2	WD repeat-containing protein 73	WDR73	42 kDa	1	1	0.8	0.8	0.8	0.9	1.4	1.6	0	0
P36404	ADP-ribosylation factor-like protein 2	ARL2	21 kDa	1	1	0.9	0.7	0.8	0.8	1.4	1.6	0	0
Q9NV70	Isoform 2 of Exocyst complex component 1	EXOC1	100 kDa	1	1	0.7	0.9	0.7	0.8	1.4	1.6	0	0
P42345	Serine/threonine-protein kinase mTOR	MTOR	289 kDa	1	1	0.8	0.9	1.3	1.3	1.3	1.6	0	0
Q7Z5K2	Cluster of Wings apart-like protein homolog	WAPAL	133 kDa	1	1	0.9	1.1	0.8	1.1	1.3	1.6	0	0
Q53H82	Beta-lactamase-like protein 2	LACTB2	33 kDa	1	1	1	0.9	0.9	1	1.3	1.6	0	0
B4DZH6	Histone deacetylase 6	HDAC6	133 kDa	1	1	0.8	0.9	0.7	0.9	1.3	1.6	0	1
Q9UKI9	Cluster of Isoform 2 of POU domain, class 2, transcription f	POU2F3	48 kDa	1	1	0.7	0.9	0.7	0.9	1.3	1.6	0	0
Q13505	Cluster of Metaxin-1	MTX1	51 kDa	1	1	0.7	1	0.7	0.7	1.3	1.6	0	1
Q8NES3	Cluster of Beta-1,3-N-acetylglucosaminyltransferase lunatic	LFNG	42 kDa	1	1	0.5	0.6	0.6	0.7	1.3	1.3	1	0

F5H1E6	DNA-directed RNA polymerase	POLR3B	121 kDa	1	1	1.4	1.5	1	1.4	1.2	1.6	0	0
Q9UBK9	Cluster of Protein UXT	UXT	18 kDa	1	1	1.2	1.2	1.2	1.3	1.2	1.6	0	0
P40937	Cluster of Replication factor C subunit 5	RFC5	38 kDa	1	1	1.2	1.3	0.8	0.9	1.2	1.6	0	0
O14561	Cluster of Acyl carrier protein, mitochondrial	NDUFAB1	17 kDa	1	1	0.9	0.9	0.8	0.9	1.2	1.6	0	0
Q5SGD2	Protein phosphatase 1L	PPM1L	41 kDa	1	1	0.6	0.6	0.7	0.7	1.2	1.6	0	1
Q8N5M4	Cluster of Tetratricopeptide repeat protein 9C	TTC9C	20 kDa	1	1	1.4	1.5	1.4	1.3	1.6	1.5	0	0
Q96A65	Cluster of Exocyst complex component 4	EXOC4	111 kDa	1	1	1	1.2	0.9	1.2	0.7	0.7	0	0
F6S928	Cluster of Mitochondrial import receptor subunit TOM5 ho	TOMM5	10 kDa	1	1	0.9	1.3	0.9	1.3	2.4	1.7	0	0
E7EUF1	Cluster of Ectonucleotide pyrophosphatase/phosphodiestera	ENPP2	102 kDa	1	1	1.6	1.5	1.7	1.5	1.9	1.7	0	1
J3KR72	Cluster of Transcription initiation factor TFIID subunit 6	TAF6	79 kDa	1	1	1.6	1.5	1.5	1.3	1.9	1.7	0	0
F5H5N1	Cluster of NADH dehydrogenase [ubiquinone] iron-sulfur p	NDUFS7	20 kDa	1	1	1.3	1.4	1.2	1.2	1.9	1.7	0	0
H0Y4W0	Cluster of COMM domain-containing protein 3 (Fragment)	COMMD3	18 kDa	No Va	1	No Value	1.2	No Value	1.3	No Val	1	0	0
H0Y6W0	Cluster of DNA topoisomerase (Fragment)	TOP3B	59 kDa	1	No Va	1.4	No Value	1.2	No Value	1.2	No Val	0	0
Q8NI27	Cluster of THO complex subunit 2	THOC2	183 kDa	No Va	1	No Value	0.6	No Value	0.8	No Val	2.6	0	0
O75530	Polycomb protein EED	EED	50 kDa	1	No Va	1.1	No Value	1	No Value	1.1	No Val	0	0
P61020	Cluster of Ras-related protein Rab-5B	RAB5B	24 kDa	1	1	1.1	0.9	1.2	1.2	1.9	1.7	0	0
Q9Y6W3	Calpain-7	CAPN7	93 kDa	1	1	1.3	1.2	1.1	1.1	1.9	1.7	0	0
F8VYA6	Striatin-4	STRN4	81 kDa	1	No Va	1.4	No Value	1.4	No Value	1.2	No Val	0	1
E9PLI6	Probable RNA-binding protein EIF1AD (Fragment)	EIF1AD	13 kDa	1	1	1.1	1	1.2	1	1.9	1.7	0	0
O15126	Secretory carrier-associated membrane protein 1	SCAMP1	38 kDa	1	1	1.2	1.2	1	0.9	1.9	1.7	0	1
Q15853	Upstream stimulatory factor 2	USF2	37 kDa	1	1	0.5	0.7	0.6	0.6	1.9	1.7	0	0
Q9H1D9	DNA-directed RNA polymerase III subunit RPC6	POLR3F	36 kDa	1	1	1.1	1.5	1.1	1.4	1.8	1.7	0	0
Q96I99	Isoform 2 of Succinyl-CoA ligase [GDP-forming] subunit b	SUCLG2	48 kDa	1	1	1.3	1.3	1.5	1.3	1.8	1.7	0	0
B4E040	Ras-related protein Ral-B	RALB	26 kDa	1	1	1	1	1.2	1.2	1.8	1.7	0	0
Q96NB3	Zinc finger protein 830	ZNF830	42 kDa	1	1	0.9	0.9	1.1	1	1.8	1.7	0	0
O00425	Insulin-like growth factor 2 mRNA-binding protein 3	IGF2BP3	64 kDa	1	1	0.9	1	1	1	1.8	1.7	0	0
Q969V3	Nicalin	NCLN	63 kDa	1	1	0.9	0.9	0.9	1	1.8	1.7	0	1
Q8WXI9	Transcriptional repressor p66-beta	GATAD2B	65 kDa	1	1	0.9	0.9	0.9	0.9	1.8	1.7	0	0
Q9C0B0	RING finger protein unkempt homolog	UNK	88 kDa	1	1	0.9	0.9	1	0.8	1.8	1.7	0	0
Q86UN2	Reticulon-4 receptor-like 1	RTN4RL1	49 kDa	1	1	0.8	0.9	0.8	0.8	1.8	1.7	3	0
Q9BWM7	Sideroflexin-3	SFXN3	36 kDa	1	1	0.9	0.9	0.8	0.7	1.8	1.7	0	1
Q9NZT2	Opioid growth factor receptor	OGFR	73 kDa	1	1	0.7	0.9	0.7	0.7	1.8	1.7	0	0
I3L4H1	Cluster of Phosphatidylinositol transfer protein alpha isofor	PITPNA	25 kDa	No Va	No Va	No Value	No Value	No Value	No Value	No Val	No Val	0	0
Q9Y303	Isoform 3 of Putative N-acetylglucosamine-6-phosphate dea	AMDHD2	64 kDa	1	1	0.8	0.9	0.6	0.7	1.8	1.7	0	0
J3KPM7	NADH dehydrogenase [ubiquinone] iron-sulfur protein 2, m	NDUFS2	52 kDa	1	1	1.4	1.4	1.7	1.8	1.7	1.7	0	0
Q9NX46	Poly(ADP-ribose) glycohydrolase ARH3	ADPRHL2	39 kDa	1	1	1.5	1.5	1.6	1.6	1.7	1.7	2	0
Q8IZ73	RNA pseudouridylate synthase domain-containing protein 2	RPUSD2	61 kDa	1	1	1.3	1.4	1.5	1.4	1.7	1.7	0	0

C9JQV0	Cluster of Uncharacterized protein C7orf50 (Fragment)	C7orf50	22 kDa	1	1	1.5	1.5	1.1	1.4	1.7	1.7	0	0
Q9UHW5	Isoform 2 of GPN-loop GTPase 3	GPN3	34 kDa	1	1	1.3	1.1	1.6	1.3	1.7	1.7	0	0
D6W5A2	Metastasis associated 1 family, member 3, isoform CRA_a	MTA3	61 kDa	1	1	1.4	1.5	1.3	1.3	1.7	1.7	0	0
H3BQQ4	FAD-linked sulfhydryl oxidase ALR	GFER	15 kDa	1	1	0.9	1	1.3	1.3	1.7	1.7	0	0
J3KNX7	NADH dehydrogenase (Ubiquinone) flavoprotein 3, 10kDa	NDUFV3	51 kDa	1	1	1.6	1.5	1.4	1.2	1.7	1.7	0	0
Q8IZH2	5'-3' exoribonuclease 1	XRN1	194 kDa	1	1	0.8	0.8	1	1.2	1.7	1.7	0	0
O15431	High affinity copper uptake protein 1	SLC31A1	21 kDa	1	1	0.9	1.2	1.1	1.1	1.7	1.7	0	1
F8WBS8	26S proteasome non-ATPase regulatory subunit 2	PSMD2	7 kDa	1	1	1.1	1	1.1	1.1	1.7	1.7	0	1
Q9UDY4	DnaJ homolog subfamily B member 4	DNAJB4	38 kDa	1	1	0.9	1	1	1.1	1.7	1.7	0	0
Q8TAA9	Vang-like protein 1	VANGL1	60 kDa	1	1	0.9	0.9	1	1	1.7	1.7	0	1
E9PDI4	Ladinin-1	LAD1	59 kDa	1	1	0.9	0.8	1	1	1.7	1.7	0	0
Q01432	AMP deaminase 3	AMPD3	89 kDa	1	1	0.9	0.8	0.9	1	1.7	1.7	0	0
F5GWY5	Podocalyxin	PODXL	59 kDa	1	1	1.6	2.1	0.9	0.9	1.7	1.7	1	1
Q8IYB8	ATP-dependent RNA helicase SUPV3L1, mitochondrial	SUPV3L1	88 kDa	1	1	0.8	0.7	0.9	0.8	1.7	1.7	0	0
H3BRY3	Coronin	CORO1A	43 kDa	1	1	0.9	1	0.7	0.7	1.7	1.7	0	0
E7ETA6	Pericentriolar material 1 protein	PCM1	228 kDa	1	1	2.7	2.7	2	2	1.6	1.7	0	0
Q15014	Mortality factor 4-like protein 2	MORF4L2	32 kDa	No Va	1	No Value	0.9	No Value	0.8	No Val	1.3	0	0
Q5T6F2	Cluster of Ubiquitin-associated protein 2	UBAP2	117 kDa	1	1	1	1.3	1.7	1.3	1.6	1.7	1	0
E5RK00	Dynactin subunit 6	DCTN6	18 kDa	1	1	1.2	1.2	1.2	1.2	1.6	1.7	0	0
F5H7V7	NADH dehydrogenase [ubiquinone] 1 alpha subcomplex su	NDUFA8	15 kDa	1	1	1	1.1	1.1	1.2	1.6	1.7	0	0
H3BMS0	RNA-binding protein with serine-rich domain 1	RNPS1	27 kDa	1	1	1	1	1.1	1.2	1.6	1.7	0	0
E9PFN5	Glutathione S-transferase kappa 1	GSTK1	22 kDa	1	1	1.4	1.2	1	1.2	1.6	1.7	0	0
O43505	N-acetyllactosaminide beta-1,3-N-acetylglucosaminyltransf	B3GNT1	47 kDa	1	1	1.1	1	1.1	1	1.6	1.7	2	0
Q9BXS4	Cluster of Transmembrane protein 59	TMEM59	36 kDa	1	1	0.8	0.8	1.1	1	1.6	1.7	1	1
F5GX65	Armadillo repeat-containing protein 10	ARMC10	25 kDa	1	1	0.8	0.9	0.9	1	1.6	1.7	0	1
Q9HCE5	Methyltransferase-like protein 14	METTL14	52 kDa	1	1	1.3	1.5	1.1	0.9	1.6	1.7	0	0
P07108	Cluster of Isoform 4 of Acyl-CoA-binding protein	DBI	14 kDa	1	1	0.8	0.9	0.9	0.9	1.6	1.7	0	0
P29372	Isoform 2 of DNA-3-methyladenine glycosylase	MPG	32 kDa	1	1	0.8	0.8	0.9	0.9	1.6	1.7	0	0
Q96QU8	Exportin-6	XPO6	129 kDa	1	1	0.8	0.9	0.8	0.8	1.6	1.7	0	0
Q9HD47	Cluster of Ran guanine nucleotide release factor	RANGRF	20 kDa	1	1	0.7	0.7	0.8	0.8	1.6	1.7	0	0
Q9H7P6	Cluster of Multivesicular body subunit 12B	MVB12B	36 kDa	1	1	0.7	0.6	0.7	0.7	1.6	1.7	0	0
P28340	DNA polymerase delta catalytic subunit	POLD1	124 kDa	1	1	1.7	1.7	1.2	1.5	1.5	1.7	0	0
Q5R372	Rab GTPase-activating protein 1-like	RABGAP1L	93 kDa	1	1	0.8	1.1	1	1.1	1.5	1.7	0	1
J3KRD0	Archaemetzincin-2 (Fragment)	AMZ2	28 kDa	1	1	1	1	1	1.1	1.5	1.7	0	0
G8JLK7	Nucleolar protein 6	NOL6	122 kDa	1	1	0.7	0.8	0.9	0.9	1.5	1.7	0	1
Q6GMV3	Putative peptidyl-tRNA hydrolase PTRHD1	PTRHD1	16 kDa	1	1	0.7	1	0.8	0.9	1.5	1.7	0	0
Q6MZV4	Protein NOXP20	FAM114A1	40 kDa	1	1	0.9	1.2	1	1.2	1.4	1.7	0	0

Q9UMX5	Neudesin	NENF	19 kDa	1	1	1.1	1.2	0.9	1.1	1.4	1.7	3	0
H0YG22	DNA-binding protein A (Fragment)	YBX3	19 kDa	1	1	0.8	1.1	0.9	1	1.4	1.7	0	0
Q96DA6	Mitochondrial import inner membrane translocase subunit T	DNAJC19	12 kDa	1	1	0.8	0.7	0.9	0.8	1.4	1.7	1	0
B7Z6B8	Cluster of 2,4-dienoyl-CoA reductase, mitochondrial	DECR1	35 kDa	1	1	1.2	0.9	1.6	1.7	1.2	1.7	0	0
Q99496	E3 ubiquitin-protein ligase RING2	RNF2	38 kDa	1	1	1	1	1	1.1	1.2	1.7	0	0
F5GZX4	DNA-directed RNA polymerase	POLR1B	132 kDa	1	1	1.1	1.2	0.9	1	1.2	1.7	0	0
E9PMQ6	Heat shock factor protein 1	HSF1	47 kDa	No Va	1	No Value	1.1	No Valu	1.1	No Val	0.7	0	0
J3KQ24	Golgin subfamily A member 7	GOLGA7	16 kDa	1	1	1	0.9	0.9	0.6	0.5	0.5	0	0
Q5VIR6	Cluster of Isoform 4 of Vacuolar protein sorting-associated	VPS53	94 kDa	1	1	1.1	1.2	1.3	1.2	2.6	1.8	0	0
O75143	Cluster of Isoform 3 of Autophagy-related protein 13	ATG13	44 kDa	1	0.9	1.9	1.7	1.7	1.5	2.3	1.8	0	0
Q15063	Periostin	POSTN	93 kDa	1	1	1	0.8	1.2	1	2.3	1.8	1	0
E9PGP2	Coagulation factor XIa heavy chain	F11	64 kDa	1	1	1.7	1.7	1.5	1.4	2.1	1.8	1	0
O95619	YEATS domain-containing protein 4	YEATS4	27 kDa	1	1	0.8	0.6	0.7	0.7	2.1	1.8	0	0
Q92995	Ubiquitin carboxyl-terminal hydrolase 13	USP13	97 kDa	1	No Va	0.9	No Value	1.2	No Value	1.3	No Val	0	0
Q9UKY7	Protein CDV3 homolog	CDV3	27 kDa	1	1	1.3	1.2	1.2	1.2	2	1.8	0	0
H3BNK9	Protein FAM192A (Fragment)	FAM192A	16 kDa	1	1	1	1	0.9	0.9	2	1.8	0	0
H3BTI0	Cysteine-rich secretory protein LCCL domain-containing 2	CRISPLD2	56 kDa	1	1	1.2	1.2	1.3	1.4	1.9	1.8	1	0
O95707	Ribonuclease P protein subunit p29	POP4	25 kDa	1	1	0.8	0.8	1.2	1.1	1.9	1.8	0	0
Q9BQI0	Allograft inflammatory factor 1-like	AIF1L	17 kDa	1	1	0.8	0.9	1.1	1.1	1.9	1.8	0	0
Q92575	UBX domain-containing protein 4	UBXN4	57 kDa	1	1	1	0.9	1.1	1	1.9	1.8	0	1
A2RUC4	Cluster of tRNA wybutosine-synthesizing protein 5	TYW5	37 kDa	1	1	0.9	0.8	0.9	1	1.9	1.8	0	0
H3BUQ9	Cluster of Na(+)/H(+) exchange regulatory cofactor NHE-R	SLC9A3R2	18 kDa	No Va	1	No Value	1.6	No Valu	1.2	No Val	1.7	0	0
O75063	Glycosaminoglycan xylosylkinase	FAM20B	46 kDa	1	1	1.1	1.4	1	1.3	1.8	1.8	0	0
O60645	Exocyst complex component 3	EXOC3	87 kDa	1	1	1.1	1.1	1.2	1.1	1.8	1.8	0	0
P08240	Signal recognition particle receptor subunit alpha	SRPR	70 kDa	1	1	1.2	1.3	1.1	1.1	1.8	1.8	0	0
H3BM74	NEDD8 ultimate buster 1	NUB1	73 kDa	1	1	1	0.9	1.1	1.1	1.8	1.8	0	0
E7ETQ9	Cluster of Dyslexia-associated protein KIAA0319-like prot	KIAA0319L	46 kDa	No Va	No Va	No Value	No Value	No Valu	No Value	No Val	No Val	0	1
H7C4X9	Cluster of Protein kinase C-binding protein 1 (Fragment)	ZMYND8	121 kDa	No Va	No Va	No Value	No Value	No Valu	No Value	No Val	No Val	0	0
B4DKD1	Protein tweety homolog 2	TTYH2	56 kDa	1	1	0.8	0.9	0.9	1.1	1.8	1.8	0	1
P45985	Dual specificity mitogen-activated protein kinase kinase 4	MAP2K4	44 kDa	1	1	1.1	1	1	1	1.8	1.8	0	0
Q5VZE5	N-alpha-acetyltransferase 35, NatC auxiliary subunit	NAA35	84 kDa	1	1	1	0.9	0.8	1	1.8	1.8	0	0
O95999	B-cell lymphoma/leukemia 10	BCL10	26 kDa	1	1	1	1	0.9	0.9	1.8	1.8	0	0
Q9H9A6	Leucine-rich repeat-containing protein 40	LRRC40	68 kDa	1	1	1	0.9	0.9	0.9	1.8	1.8	0	0
Q5JTV8	Torsin-1A-interacting protein 1	TOR1AIP1	66 kDa	1	1	0.9	0.9	0.9	0.9	1.8	1.8	0	1
Q5W0V3	Protein FAM160B1	FAM160B1	87 kDa	1	No Va	0.7	No Value	0.8	No Value	0.7	No Val	0	0
O60487	Myelin protein zero-like protein 2	MPZL2	24 kDa	1	1	0.6	0.7	0.8	0.9	1.8	1.8	0	1
Q15542	Transcription initiation factor TFIID subunit 5	TAF5	87 kDa	1	1	0.8	1.1	1	0.8	1.8	1.8	0	0

P49959	Cluster of Isoform 2 of Double-strand break repair protein M	MRE11A	78 kDa	1	1	1.9	1.7	1.5	1.4	1.7	1.8	0	0
B5MC72	Endoplasmic reticulum lectin 1	ERLEC1	52 kDa	1	1	1	1.1	1.3	1.3	1.7	1.8	0	0
Q9NQZ2	Something about silencing protein 10	UTP3	55 kDa	1	1	1.1	1.1	1.1	1.1	1.7	1.8	0	0
P61218	Cluster of DNA-directed RNA polymerases I, II, and III sub	POLR2F	14 kDa	1	1	1.2	1.1	1.1	1	1.7	1.8	0	0
P0CB43	Protein FAM203B	HGH1	42 kDa	1	No Va	1.5	No Value	1.4	No Value	1	No Val	0	0
Q96IY1	Cluster of Kinetochore-associated protein NSL1 homolog	NSL1	32 kDa	1	1	0.9	1	1	1	1.7	1.8	0	0
H0YJ66	Dehydrogenase/reductase SDR family member 7 (Fragment	DHRS7	45 kDa	1	1	0.9	0.8	1	0.9	1.7	1.8	1	0
B4E1F0	Plasma protease C1 inhibitor	SERPING1	56 kDa	1	1	0.7	0.6	0.9	0.9	1.7	1.8	1	0
O94813	Slit homolog 2 protein	SLIT2	170 kDa	1	1	0.9	0.9	0.8	0.8	1.7	1.8	1	1
O75449	Cluster of Katanin p60 ATPase-containing subunit A1	KATNA1	56 kDa	1	1	0.7	0.9	0.8	0.8	1.7	1.8	0	0
Q9Y5H3	Cluster of Protocadherin gamma-A10	PCDHGA10	101 kDa	1	1	1.1	1.2	1.3	1.1	1.6	1.8	0	1
F6QR24	Nuclear pore complex protein Nup153	NUP153	157 kDa	1	1	1	1	1.1	1	1.6	1.8	0	0
Q96FZ7	Cluster of Charged multivesicular body protein 6	CHMP6	23 kDa	1	1	0.8	0.9	0.9	1	1.6	1.8	0	0
E9PQE8	Cluster of Trafficking protein particle complex subunit 4	TRAPPC4	19 kDa	1	1	1	1	0.9	0.9	1.6	1.8	0	0
Q86U86	Protein polybromo-1	PBRM1	193 kDa	1	1	0.9	0.8	1.1	0.8	1.6	1.8	0	0
Q7Z3E2	Cluster of Uncharacterized protein C10orf118	CCDC186	104 kDa	1	1	1.6	1.2	1.5	1.6	1.5	1.8	0	0
P51398	Cluster of 28S ribosomal protein S29, mitochondrial	DAP3	46 kDa	1	1	0.6	1.1	0.7	1	0.3	0.5	0	0
Q14574	Isoform 3B of Desmocollin-3	DSC3	93 kDa	1	1	1.2	0.9	1.2	1.2	1.5	1.8	0	1
Q7Z4H8	Cluster of KDEL motif-containing protein 2	KDELC2	59 kDa	1	1	1	1.2	1.1	1.2	1.5	1.8	1	0
H0YH87	Cluster of Ataxin-2 (Fragment)	ATXN2	98 kDa	1	1	1	1	1.1	1.2	1.5	1.8	0	0
O76070	Cluster of Gamma-synuclein	SNCG	13 kDa	1	1	2.2	1.8	0.7	0.8	0.7	0.5	0	0
H0YIM9	Cluster of Protein CHURC1-FNTB (Fragment)	CHURC1-FNT	10 kDa	1	1	0.9	1.2	1	1.2	1.4	1.8	0	0
Q96F86	Cluster of Enhancer of mRNA-decapping protein 3	EDC3	56 kDa	1	1	1	0.8	1	0.8	1.4	1.8	0	0
Q9NPQ8	Cluster of Isoform 2 of Synembryn-A	RIC8A	59 kDa	1	1	1.4	1.6	1.2	1.4	1.3	1.8	0	0
Q9Y4Z0	U6 snRNA-associated Sm-like protein LSm4	LSM4	15 kDa	1	1	1.4	0.9	0.7	0.7	1	1.1	0	0
Q4G176	Cluster of Acyl-CoA synthetase family member 3, mitochor	ACSF3	64 kDa	1	1	1.1	1	0.7	0.9	1.3	1.8	0	1
Q16836	Cluster of Isoform 2 of Hydroxyacyl-coenzyme A dehydrog	HADH	42 kDa	No Va	No Va	No Value	No Value	No Value	No Value	No Val	No Val	0	0
H0YCJ8	Blood group Rh(CE) polypeptide (Fragment)	RHCE	8 kDa	1	1	1.9	2	1.6	1.6	1.2	1.8	0	1
H0Y4D4	Cluster of 3-ketoacyl-CoA thiolase, peroxisomal (Fragment	ACAA1	25 kDa	No Va	No Va	No Value	No Value	No Value	No Value	No Val	No Val	0	0
Q8WW22	DnaJ homolog subfamily A member 4	DNAJA4	45 kDa	1	1	1	1.1	1.3	1.3	1.2	1.8	0	0
Q6PIJ6	Cluster of Isoform 2 of F-box only protein 38	FBXO38	126 kDa	1	1	1.1	1.2	1	1.4	1.1	1.8	0	0
Q8NEL9	Cluster of Phospholipase DDHD1	DDHD1	100 kDa	1	1	0.6	0.8	0.9	0.7	1.3	1.3	0	0
Q96A33	Cluster of Coiled-coil domain-containing protein 47	CCDC47	56 kDa	No Va	No Va	No Value	No Value	No Value	No Value	No Val	No Val	0	1
J3KQJ1	Cluster of Sulfatase-modifying factor 2	SUMF2	36 kDa	1	1	0.7	0.7	0.9	0.9	2.5	1.9	1	0
J3QT38	Cluster of Exocyst complex component 6B	EXOC6B	79 kDa	1	1	1	1.5	1.3	1.1	2.4	1.9	0	0
P51946	Cluster of Cyclin-H	CCNH	38 kDa	1	1	1.2	1.3	1.4	1.4	2.3	1.9	0	0
Q8TDZ2	Cluster of Isoform 4 of Protein-methionine sulfoxide oxidas	MICAL1	120 kDa	1	No Va	1.9	No Value	1.2	No Value	1.5	No Val	0	0

Q9NUQ6	Cluster of Isoform 2 of SPATS2-like protein	SPATS2L	54 kDa	No Va	1	No Value	1.1	No Value	1.3	No Val	1.3	0	0
H0YHT2	Cluster of Kinesin-like protein KIF21A (Fragment)	KIF21A	109 kDa	No Va	1	No Value	0.9	No Value	0.8	No Val	1.2	0	0
H3BSA4	Na(+)/H(+) exchange regulatory cofactor NHE-RF2 (Fragm	SLC9A3R2	19 kDa	1	1	1.3	1.2	1.2	1	2.3	1.9	0	0
P14649	Myosin light chain 6B	MYL6B	23 kDa	No Va	1	No Value	1	No Value	0.9	No Val	0.9	0	0
P49643	DNA primase large subunit	PRIM2	59 kDa	No Va	1	No Value	1	No Value	0.7	No Val	1.1	0	0
O43688	Isoform 2 of Lipid phosphate phosphohydrolase 2	PPAP2C	34 kDa	1	1	3.2	2.9	2.8	2.5	2.2	1.9	0	1
Q8N8N7	Prostaglandin reductase 2	PTGR2	38 kDa	1	1	1.4	1	1.4	1.5	2.2	1.9	0	0
Q8TEM1	Nuclear pore membrane glycoprotein 210	NUP210	205 kDa	1	1	1.1	0.9	1.2	1	2.2	1.9	0	1
O95084	Serine protease 23	PRSS23	43 kDa	1	1	1	1	1.3	1.2	2.1	1.9	1	0
Q8WZA9	Immunity-related GTPase family Q protein	IRGQ	63 kDa	1	1	1.4	1.4	1.1	1.1	2.1	1.9	0	0
Q8IZL8	Isoform 2 of Proline-, glutamic acid- and leucine-rich protei	PELP1	135 kDa	No Va	1	No Value	0.8	No Value	1	No Val	0.7	0	1
Q15648	Mediator of RNA polymerase II transcription subunit 1	MED1	168 kDa	1	1	1.1	1.1	1	1.1	2.1	1.9	0	0
Q15334	Lethal(2) giant larvae protein homolog 1	LLGL1	115 kDa	1	1	1.2	1.2	1.1	1	2.1	1.9	0	0
Q6DKI1	60S ribosomal protein L7-like 1	RPL7L1	29 kDa	1	1	1	1	1	1	2.1	1.9	0	0
Q7Z417	Nuclear fragile X mental retardation-interacting protein 2	NUFIP2	76 kDa	1	1	0.8	0.9	0.9	0.9	2.1	1.9	0	0
O95479	GDH/6PGL endoplasmic bifunctional protein	H6PD	89 kDa	1	1	0.8	0.7	1	0.8	2.1	1.9	1	0
Q9H5X1	MIP18 family protein FAM96A	FAM96A	18 kDa	1	1	0.7	0.8	0.7	0.8	2.1	1.9	1	0
H0YE28	Protein C11orf31 (Fragment)	C11orf31	10 kDa	1	1	1.4	1.3	1.1	1.1	2	1.9	0	0
O15084	Serine/threonine-protein phosphatase 6 regulatory ankyrin r	ANKRD28	113 kDa	1	1	0.8	0.7	0.8	0.8	2	1.9	0	0
Q12962	Transcription initiation factor TFIID subunit 10	TAF10	22 kDa	1	1	1.5	1.5	1.4	1.4	1.9	1.9	0	0
Q8IWB7	WD repeat and FYVE domain-containing protein 1	WDFY1	46 kDa	1	1	1.2	1.3	1.4	1.4	1.9	1.9	0	0
Q9UBS3	DnaJ homolog subfamily B member 9	DNAJB9	26 kDa	1	1	0.9	0.9	1.1	1	1.9	1.9	2	0
Q8N0X4	Citrate lyase subunit beta-like protein, mitochondrial	CLYBL	37 kDa	1	1	0.9	1	0.8	0.9	1.9	1.9	0	1
Q9Y2S7	Polymerase delta-interacting protein 2	POLDIP2	42 kDa	1	1	0.8	0.8	0.7	0.7	1.9	1.9	0	0
P55011	Solute carrier family 12 member 2	SLC12A2	131 kDa	1	1	0.7	0.6	0.7	0.6	1.9	1.9	0	1
Q9UKZ1	UPF0760 protein C2orf29	CNOT11	55 kDa	1	1	1.2	1.1	1.3	1.4	1.8	1.9	0	0
O75752	UDP-GalNAc:beta-1,3-N-acetylgalactosaminyltransferase 1	B3GALNT1	40 kDa	1	1	1.3	1.2	1.2	1.2	1.8	1.9	1	0
H3BM91	COMM domain-containing protein 4 (Fragment)	COMMD4	23 kDa	1	1	1	1	1.2	1.2	1.8	1.9	0	0
P21964	Catechol O-methyltransferase	COMT	30 kDa	1	1	1.3	1.3	1.1	1.2	1.8	1.9	0	1
Q6NUK1	Calcium-binding mitochondrial carrier protein SCaMC-1	SLC25A24	53 kDa	1	1	1.2	1.2	1.2	1	1.8	1.9	0	0
E9PJM3	Cluster of F-box only protein 3	FBXO3	49 kDa	1	1	0.9	0.9	1	1	1.8	1.9	0	0
H0YBQ0	Thioredoxin reductase 3 (Fragment)	TXNRD3	76 kDa	1	1	0.8	1	0.9	1	1.8	1.9	0	0
E7EWW0	UPF0505 protein C16orf62	C16orf62	119 kDa	No Va	1	No Value	1.3	No Value	1.4	No Val	0.6	0	1
B3KPE7	Bcl-2-like protein 13	BCL2L13	35 kDa	1	1	0.7	0.7	0.9	0.9	1.8	1.9	0	1
Q9P2X0	Dolichol-phosphate mannosyltransferase subunit 3	DPM3	10 kDa	1	1	0.7	0.8	0.7	0.9	1.8	1.9	0	1
E7EWE9	Kinetochore protein Spc24	SPC24	17 kDa	1	1	1	1	1	0.8	1.8	1.9	0	0
Q49AR2	UPF0489 protein C5orf22	C5orf22	50 kDa	1	1	0.8	0.8	0.9	0.7	1.8	1.9	0	0

O75955	Flotillin-1	FLOT1	47 kDa	1	1	0.8	0.6	0.7	0.7	1.8	1.9	0	0
Q9NWU2	Glucose-induced degradation protein 8 homolog	GID8	27 kDa	1	1	0.6	0.6	0.6	0.5	1.8	1.9	0	0
Q9UBP0	Spastin	SPAST	67 kDa	1	1	0.7	0.7	0.8	0.6	0.6	0.3	0	1
B4DKB2	Endothelin-converting enzyme 1	ECE1	84 kDa	1	1	1	1.2	1.1	1.2	1.7	1.9	0	1
Q9NVU7	Protein SDA1 homolog	SDAD1	80 kDa	1	1	1.2	1	1.1	1.1	1.7	1.9	0	0
Q9BV44	THUMP domain-containing protein 3	THUMPD3	57 kDa	No Va	1	No Value	1.1	No Valu	0.8	No Val	1.1	0	0
Q5F1R6	Isoform 2 of DnaJ homolog subfamily C member 21	DNAJC21	67 kDa	1	1	0.7	0.9	0.9	1	1.7	1.9	0	0
C9JFV8	Deoxyguanosine kinase, mitochondrial	DGUOK	30 kDa	1	1	1	0.9	0.9	0.9	1.7	1.9	0	0
B7ZKK9	Cluster of PPP2R5E protein	PPP2R5E	54 kDa	1	1	1	0.9	0.9	0.9	1.7	1.9	0	0
Q9NVE7	Pantothenate kinase 4	PANK4	86 kDa	1	1	1	1.1	1.1	1.2	1.6	1.9	0	0
A6NHG4	D-dopachrome decarboxylase-like protein	DDTL	14 kDa	1	1	0.9	1.2	0.9	1.1	1.6	1.9	0	0
J3QRV5	Lethal(2) giant larvae protein homolog 2	LLGL2	113 kDa	1	1	0.7	0.7	0.9	1	1.6	1.9	1	0
Q8TF42	Ubiquitin-associated and SH3 domain-containing protein B	UBASH3B	73 kDa	1	1	0.7	0.7	0.5	0.6	1.6	1.9	0	0
Q9H8M7	Protein FAM188A	FAM188A	50 kDa	1	1	1	1	0.8	0.9	1	0.9	0	0
Q9Y5Z4	Heme-binding protein 2	HEBP2	23 kDa	1	1	1.9	1.6	1.9	1.7	1.5	1.9	0	0
P51178	1-phosphatidylinositol 4,5-bisphosphate phosphodiesterase	PLCD1	86 kDa	1	1	0.8	1	0.9	1.3	1.5	1.9	0	0
Q9NX02	Cluster of NACHT, LRR and PYD domains-containing prot	NLRP2	121 kDa	1	1	1.1	1.6	1.2	1.2	1.5	1.9	0	0
Q9C0B5	Palmitoyltransferase ZDHHC5	ZDHHC5	78 kDa	1	1	0.8	1.1	0.7	0.8	1.5	1.9	0	1
J3KMX4	Ragulator complex protein LAMTOR3	LAMTOR3	13 kDa	1	1	1	1.1	0.9	1.1	1.4	1.9	0	0
Q9NRX5	Serine incorporator 1	SERINC1	50 kDa	1	1	1	0.9	0.7	1	1.4	1.9	0	1
Q96KP1	Exocyst complex component 2	EXOC2	104 kDa	No Va	1	No Value	1.1	No Valu	1.1	No Val	1	0	0
P54289	Voltage-dependent calcium channel subunit alpha-2/delta-1	CACNA2D1	125 kDa	1	1	1.1	1	1	1	1.2	1.9	1	1
P78316	Nucleolar protein 14	NOP14	98 kDa	No Va	1	No Value	0.6	No Valu	1	No Val	1.2	0	0
P24385	G1/S-specific cyclin-D1	CCND1	34 kDa	1	1	1.2	1.3	1.5	1.8	1	0.9	0	0
Q9BW61	DET1- and DDB1-associated protein 1	DDA1	12 kDa	1	1	1.3	1.4	1.5	1.4	2.6	2	0	0
Q5SRE5	Nucleoporin NUP188 homolog	NUP188	196 kDa	No Va	1	No Value	0.8	No Valu	1	No Val	0.6	0	1
Q9ULJ6	Cluster of Zinc finger MIZ domain-containing protein 1	ZMIZ1	115 kDa	1	1	1.4	1.1	1.3	1.2	2.5	2	0	0
P82909	28S ribosomal protein S36, mitochondrial	MRPS36	11 kDa	1	1	1	1	1.5	1.4	2.4	2	0	0
O15357	Phosphatidylinositol 3,4,5-trisphosphate 5-phosphatase 2	INPPL1	139 kDa	1	1	1.2	1.2	1.1	1	2.4	2	0	0
Q9H6R7	WD repeat-containing protein C2orf44	C2orf44	79 kDa	1	1	1.2	1	1.1	1	2.4	2	0	0
Q9BV19	Uncharacterized protein C1orf50	C1orf50	22 kDa	1	1	0.9	0.8	1.1	1	2.4	2	0	1
Q7Z4H3	HD domain-containing protein 2	HDDC2	23 kDa	1	1	2	1.9	1.6	1.4	2.3	2	0	0
P24666	Low molecular weight phosphotyrosine protein phosphatase	ACP1	18 kDa	1	1	1.5	1.3	1.4	1.2	2.3	2	0	0
Q9BQC3	Diphthamide biosynthesis protein 2	DPH2	52 kDa	1	1	0.9	1	1.1	1.1	2.3	2	0	0
Q9Y305	Acyl-coenzyme A thioesterase 9, mitochondrial	ACOT9	50 kDa	1	1	1.3	1.1	1.4	1.2	2.2	2	0	0
Q3ZCQ8	Isoform 2 of Mitochondrial import inner membrane transloc	TIMM50	50 kDa	1	1	1.3	1.1	1.3	1.1	2.2	2	0	1
Q14116	Interleukin-18	IL18	22 kDa	1	1	0.7	1.3	0.7	1.2	1.5	1.8	0	0

B4DRF9	Cyclic AMP-dependent transcription factor ATF-1	ATF1	15 kDa	1	1	1.5	1.6	1.2	1.1	2.1	2	0	0
P13726	Tissue factor	F3	33 kDa	1	1	0.9	0.9	1.1	1.1	2.1	2	0	1
P06213	Insulin receptor	INSR	156 kDa	1	1	1	0.8	1.1	0.8	2.1	2	0	1
O43805	Sjoegren syndrome nuclear autoantigen 1	SSNA1	14 kDa	1	1	0.8	0.9	0.6	0.6	2.1	2	0	0
Q9NQZ5	StAR-related lipid transfer protein 7, mitochondrial	STARD7	43 kDa	1	1	3.3	3	5.2	4.8	2	2	1	0
Q9BPX5	Actin-related protein 2/3 complex subunit 5-like protein	ARPC5L	17 kDa	1	No Va	1	No Value	0.8	No Value	0.8	No Val	0	0
Q9C0D3	Protein zyg-11 homolog B	ZYG11B	84 kDa	1	1	1	1	1.1	1.3	2	2	0	0
P24821	Isoform 6 of Tenascin	TNC	171 kDa	1	1	1.1	1.2	1.2	1.1	2	2	1	0
O75521	Enoyl-CoA delta isomerase 2, mitochondrial	ECI2	44 kDa	1	1	1	1.1	1.1	1.1	2	2	0	0
Q9P2B4	CTTNBP2 N-terminal-like protein	CTTNBP2NL	70 kDa	No Va	1	No Value	1.5	No Value	1.8	No Val	1.4	0	0
P09234	Cluster of U1 small nuclear ribonucleoprotein C	SNRPC	17 kDa	1	1	1	1	1.1	1	2	2	0	0
Q8NFU3	Thiosulfate sulfurtransferase/rhodanese-like domain-contai	TSTD1	13 kDa	1	1	0.9	0.9	1	1	2	2	0	0
B4DP36	Calcium-binding and coiled-coil domain-containing protein	CALCOCO2	55 kDa	1	1	0.7	0.7	0.7	0.7	2	2	0	0
E9PC90	Cluster of G2/mitotic-specific cyclin-B1 (Fragment)	CCNB1	45 kDa	1	1	0.6	0.6	0.6	0.6	2	2	1	0
B2RAK2	MHC class I polypeptide-related sequence B	MICB	43 kDa	1	1	1.2	1.2	1.4	1.3	1.9	2	1	1
G3V4K3	Spermatogenesis-defective protein 39 homolog	VIPAS39	60 kDa	1	1	0.8	0.8	0.9	0.9	1.9	2	0	0
C9JWR9	3-phosphoinositide-dependent protein kinase 1	PDPK1	61 kDa	1	1	0.8	0.7	0.9	0.8	1.9	2	0	0
P15407	Fos-related antigen 1	FOSL1	29 kDa	1	1	1.3	1.3	1.2	1.3	1.8	2	0	0
Q96T37	Cluster of Isoform 3 of Putative RNA-binding protein 15	RBM15	106 kDa	1	1	1.9	1.7	1.9	1.6	1.7	2	0	0
Q7L985	Cluster of Leucine-rich repeat and immunoglobulin-like do	LINGO2	68 kDa	1	1	1	1.1	1.2	1.2	1.6	2	0	1
O00217	NADH dehydrogenase [ubiquinone] iron-sulfur protein 8, m	NDUFS8	24 kDa	1	1	0.9	1.1	0.8	1.1	1.5	2	1	0
Q86VP3	Isoform 3 of Phosphofurin acidic cluster sorting protein 2	PACS2	98 kDa	1	No Va	1.1	No Value	0.9	No Value	1.2	No Val	0	0
Q8WUW1	Protein BRICK1	BRK1	9 kDa	1	1	0.7	0.8	0.6	0.8	1.4	2	0	0
A8MSH5	UPF0549 protein C20orf43	RTFDC1	38 kDa	1	1	1.4	1	1.1	1.1	1.3	0.7		
O43318	Mitogen-activated protein kinase kinase kinase 7	MAP3K7	67 kDa	1	1	0.6	0.9	1	1	1.3	1.2	0	0
B8ZZD4	Tax1-binding protein 1	TAX1BP1	94 kDa	No Va	1	No Value	1.2	No Value	0.9	No Val	0.8	0	1
Q9NWV4	Cluster of UPF0587 protein C1orf123	C1orf123	18 kDa	1	1	3	2.4	1.3	1.1	3	2.1	0	0
Q8TDN6	Ribosome biogenesis protein BRX1 homolog	BRIX1	41 kDa	1	1	1	1	1.3	1.1	2.6	2.1	0	0
B4DME2	Protein phosphatase 1 regulatory subunit 12C	PPP1R12C	77 kDa	1	1	1.3	1.2	1.3	1.1	2.5	2.1	0	0
F5H6J9	Activating signal cointegrator 1 complex subunit 2	ASCC2	77 kDa	1	1	1	0.9	1.1	1.1	2.4	2.1	0	0
Q8NBN3	Transmembrane protein 87A	TMEM87A	63 kDa	1	1	1.2	1.2	1	1.1	2.3	2.1	1	1
A9UHW6	MIF4G domain-containing protein	MIF4GD	25 kDa	1	1	1.5	1.4	1.2	1.2	2.2	2.1	0	0
Q9HA65	TBC1 domain family member 17	TBC1D17	73 kDa	1	1	0.7	0.7	1	1	2.2	2.1	0	0
O75843	Cluster of AP-1 complex subunit gamma-like 2	AP1G2	87 kDa	1	1	1.2	1.1	1.2	1.3	2.1	2.1	0	0
H7C2N1	Cluster of Prothymosin alpha (Fragment)	PTMA	16 kDa	No Va	No Va	No Value	No Value	No Value	No Value	No Val	No Val	0	0
Q9BUT1	Cluster of 3-hydroxybutyrate dehydrogenase type 2	BDH2	27 kDa	1	1	1	1.1	1.1	1.2	2.1	2.1	1	0
Q96CB8	Cluster of Integrator complex subunit 12	INTS12	49 kDa	1	1	1.4	1.4	1.1	1.1	2.1	2.1	0	0

P00403	Cytochrome c oxidase subunit 2	MT-CO2	26 kDa	No V	1	No Value	0.7	No Valu	1	No Val	0.2	0	1
O95104	Splicing factor, arginine/serine-rich 15	SCAF4	126 kDa	1	1	0.8	0.8	0.9	0.9	2.1	2.1	0	0
Q9ULD2	Cluster of Isoform 2 of Microtubule-associated tumor suppr	MTUS1	136 kDa	No V	No Va	No Value	No Value	No Valu	No Value	No Val	No Val	0	1
Q9NX55	Cluster of Huntingtin-interacting protein K	HYPK	15 kDa	1	1	1.4	1.5	1.5	1.4	2	2.1	0	0
H3BLY1	Cluster of Contactin-associated protein-like 3B (Fragment)	CNTNAP3B	129 kDa	1	1	1.1	1.2	1.3	1.4	2	2.1	1	1
H7BZW1	Cluster of Uncharacterized protein (Fragment)		34 kDa	1	1	1.2	1.2	1.2	1.2	2	2.1		
Q96TC7	Cluster of Regulator of microtubule dynamics protein 3	RMDN3	52 kDa	1	1	1.1	1	1.1	1.2	2	2.1	1	1
Q9H254	Spectrin beta chain, non-erythrocytic 4	SPTBN4	289 kDa	No V	No Va	No Value	No Value	No Valu	No Value	No Val	No Val	0	0
F5H801	Cluster of 2-oxoglutarate dehydrogenase, mitochondrial	OGDH	111 kDa	1	No Va	1.3	No Value	0.7	No Value	0.6	No Val	0	0
E9PMG1	Cluster of RalBP1-associated Eps domain-containing protei	REPS1	80 kDa	1	1	1	1.1	1.1	1	2	2.1	0	0
Q9UPY3	Cluster of Endoribonuclease Dicer	DICER1	219 kDa	No V	1	No Value	1.2	No Valu	1.3	No Val	0.9	0	0
E9PIL6	Cluster of Lysine-specific demethylase 2A	KDM2A	82 kDa	1	1	0.6	0.8	0.9	0.9	2	2.1	0	0
Q13043	Cluster of Serine/threonine-protein kinase 4	STK4	56 kDa	1	1	0.7	0.7	0.6	0.8	2	2.1	0	0
Q6PGP7	Cluster of Tetratricopeptide repeat protein 37	TTC37	175 kDa	1	No Va	1	No Value	1.1	No Value	0.4	No Val	0	0
E9PBC6	Cluster of Transforming acidic coiled-coil-containing protei	TACC2	303 kDa	No V	No Va	No Value	No Value	No Valu	No Value	No Val	No Val	0	0
O00214	Isoform 2 of Galectin-8	LGALS8	40 kDa	1	No Va	0.9	No Value	1.1	No Value	1.3	No Val	0	0
Q5BKZ1	DBIRD complex subunit ZNF326	ZNF326	66 kDa	1	1	0.9	0.9	1.2	1.4	1.9	2.1	0	0
E9PCR4	Anaphase-promoting complex subunit 4	ANAPC4	92 kDa	1	1	0.9	0.9	0.9	0.9	1.9	2.1	0	0
P41236	Protein phosphatase inhibitor 2	PPP1R2	23 kDa	No V	1	No Value	1.1	No Valu	1	No Val	0.9	0	0
Q9BRZ2	E3 ubiquitin-protein ligase TRIM56	TRIM56	81 kDa	1	1	1	0.7	0.8	0.8	1.9	2.1	0	0
D3YTF8	Cluster of Thioredoxin reductase 2, mitochondrial	TXNRD2	54 kDa	1	1	1	1.2	1.2	1.2	1.8	2.1	0	0
A3KN83	Protein strawberry notch homolog 1	SBNO1	154 kDa	1	1	0.7	0.6	0.7	0.7	1.7	2.1	0	0
P16930	Fumarylacetoacetase	FAH	46 kDa	1	1	1	0.9	0.9	1.2	1.6	2.1	0	0
Q96BH1	E3 ubiquitin-protein ligase RNF25	RNF25	51 kDa	1	1	1.4	1.5	1.3	1.4	2.6	2.2	0	0
Q9Y2Q5	Ragulator complex protein LAMTOR2	LAMTOR2	14 kDa	1.1	1	1.1	1.2	1.2	1.3	2.4	2.2	0	0
I3L3S0	Trafficking protein particle complex subunit 1 (Fragment)	TRAPPC1	11 kDa	1	1	1.4	1.3	1.5	1.4	2.3	2.2	0	0
Q2NL82	Pre-rRNA-processing protein TSR1 homolog	TSR1	92 kDa	1	1	1.2	1.3	1.2	1.2	2.3	2.2	0	0
Q9Y5X2	Sorting nexin-8	SNX8	53 kDa	1	1	0.8	0.7	1.1	1.1	2.3	2.2	0	0
H0YFI1	Ragulator complex protein LAMTOR1 (Fragment)	LAMTOR1	9 kDa	1	1	1.1	1.1	1.5	1.5	2.2	2.2	0	0
O60493	Sorting nexin-3	SNX3	19 kDa	1	1	0.9	0.9	1.2	1.2	2.2	2.2	0	0
B1AMW1	CD58 antigen, (Lymphocyte function-associated antigen 3),	CD58	27 kDa	1	1	0.9	0.8	1	1.2	2.2	2.2	0	1
Q56VL3	OCIA domain-containing protein 2	OCIAD2	17 kDa	1	1	1.4	1.4	1.1	1.1	2.2	2.2	0	0
Q9BYG3	MKI67 FHA domain-interacting nucleolar phosphoprotein	NIFK	34 kDa	1	1	0.9	1	1	1	2.2	2.2	0	0
Q14181	DNA polymerase alpha subunit B	POLA2	66 kDa	1	1	0.9	0.9	0.8	0.9	2.2	2.2	0	0
Q9BQP7	Uncharacterized protein C20orf72	MGME1	39 kDa	1	1	1	1	1.1	1.2	2.1	2.2	0	0
F5GWN1	Protein SSXT	SS18	4 kDa	1	1	0.8	0.9	0.9	0.9	2.1	2.2	0	0
Q9BYB4	Guanine nucleotide-binding protein subunit beta-like protei	GNB1L	36 kDa	1	No Va	1	No Value	0.8	No Value	0.9	No Val	0	0

P57735	Ras-related protein Rab-25	RAB25	23 kDa	1	1	1	1.1	0.8	0.8	2.1	2.2	0	0
A4D1W8	Ependymin related protein 1 (Zebrafish), isoform CRA_b	EPDR1	38 kDa	1	1	1.2	1.2	1.2	1.4	2	2.2	1	1
A6NFM0	Cluster of DNA-directed RNA polymerase II subunit RPB1	POLR2J2	17 kDa	1	1	1.2	1.3	1.1	1.2	2	2.2	0	0
O60333	Kinesin-like protein KIF1B	KIF1B	204 kDa	1	No Va	1.3	No Value	1	No Value	0.9	No Val	0	0
O75493	Carbonic anhydrase-related protein 11	CA11	36 kDa	1	1	1.3	1.5	1.3	1.4	1.7	2.2	1	0
B9ZVT1	RNA-binding protein 12B	RBM12B	103 kDa	1	1	1.4	1	1.3	1.1	1.7	2.2	0	0
Q86W42	THO complex subunit 6 homolog	THOC6	38 kDa	1	1	1.2	1.4	1.1	1.5	1.6	2.2	0	0
Q9H6U6	Isoform 4 of Breast carcinoma-amplified sequence 3	BCAS3	104 kDa	1	1	0.9	1	0.9	1.4	1.5	2.2	0	0
Q96H79	Zinc finger CCCH-type antiviral protein 1-like	ZC3HAV1L	33 kDa	1	1	0.7	0.7	0.8	0.9	1.5	2.2	0	0
C9J8Q5	Succinate-semialdehyde dehydrogenase, mitochondrial	ALDH5A1	54 kDa	1	1	1.6	0.7	1.5	0.7	1.4	0.6	0	0
Q9H2P9	Diphthine synthase	DPH5	32 kDa	0.9	1	1.2	1.1	1.2	1	3.3	2.3	0	0
Q8NEW0	Zinc transporter 7	SLC30A7	42 kDa	1	1	1.3	1.2	1.5	1.5	2.9	2.3	0	1
P86790	Vacuolar fusion protein CCZ1 homolog B	CCZ1B	56 kDa	1	No Va	1.1	No Value	1.1	No Value	0.6	No Val	0	0
Q969E8	Pre-rRNA-processing protein TSR2 homolog	TSR2	21 kDa	1	1	1.4	1.4	1.4	1.3	2.7	2.3	0	0
Q92636	Protein FAN	NSMAF	104 kDa	1	1	1.4	1.5	2	1.9	2.5	2.3	0	0
C9JJV1	Protein TSSC4 (Fragment)	TSSC4	22 kDa	1	1	1.2	1.2	1.3	1.3	2.5	2.3	0	0
A6NN98	Myotubularin related protein 2, isoform CRA_a	MTMR2	66 kDa	1	1	1.5	1.4	1.1	1.3	2.5	2.3	0	0
H3BP13	Trafficking protein particle complex subunit 2-like protein	TRAPPC2L	27 kDa	1	1	1.6	1.4	1.8	1.5	2.3	2.3	0	0
G8JLI5	WD repeat domain phosphoinositide-interacting protein 4	WDR45	38 kDa	1	1	1	1	1	1	2.3	2.3	0	1
Q9Y3Z3	SAM domain and HD domain-containing protein 1	SAMHD1	72 kDa	No Va	1	No Value	1.1	No Value	1.2	No Val	1	0	0
Q96ED9	Protein Hook homolog 2	HOOK2	83 kDa	1	No Va	1.7	No Value	1.4	No Value	1.5	No Val	0	0
Q96FJ0	AMSH-like protease	STAMBPL1	50 kDa	1	1	0.9	0.9	1	1.1	2.2	2.3	0	0
B4DDV1	Interferon-induced protein with tetratricopeptide repeats 5	IFIT5	51 kDa	1	1	1.3	1.7	1.3	1.7	2	2.3	0	0
Q9NV96	Cell cycle control protein 50A	TMEM30A	41 kDa	1	No Va	1.7	No Value	1.3	No Value	0.8	No Val	0	1
Q9NRX1	RNA-binding protein PNO1	PNO1	28 kDa	1	No Va	1.8	No Value	1.2	No Value	1.8	No Val	0	0
Q6ZMP0	Thrombospondin type-1 domain-containing protein 4	THSD4	112 kDa	1	1	2.1	2.1	1.4	1.5	2	2.3	1	0
Q15714	TSC22 domain family protein 1	TSC22D1	110 kDa	1	1	1.1	1.1	1.2	1.3	2	2.3	0	0
B8X2Z3	CARMIL2b	RLTPR	148 kDa	1	1	0.8	0.8	0.9	1	2	2.3	0	0
C9JJ19	28S ribosomal protein S34, mitochondrial	MRPS34	26 kDa	1	1	1.4	1.5	1.1	1.5	1.9	2.3	0	0
Q9BSL1	Ubiquitin-associated domain-containing protein 1	UBAC1	45 kDa	No Va	1	No Value	1	No Value	1	No Val	1.2	0	0
G5E9W7	28S ribosomal protein S22, mitochondrial	MRPS22	37 kDa	1	1	0.9	0.9	0.9	0.9	1.8	2.3	1	0
Q9NVZ3	Adaptin ear-binding coat-associated protein 2	NECAP2	28 kDa	1	1	1.1	1.3	0.8	0.9	1.8	2.3	0	0
P23434	Glycine cleavage system H protein, mitochondrial	GCSH	19 kDa	1	1	0.7	1	0.8	0.8	1.8	2.3	1	0
Q6P1K2	Isoform 4 of Polyamine-modulated factor 1	PMF1	19 kDa	1	1	1.6	1.6	1.2	1.2	2.7	2.4	0	0
P82932	28S ribosomal protein S6, mitochondrial	MRPS6	14 kDa	1	1	2.1	1.9	2.6	2.6	2.5	2.4	0	0
Q92889	DNA repair endonuclease XPF	ERCC4	104 kDa	1	1	1.3	1.2	1.4	1.2	2.5	2.4	0	0
Q9NZN4	Cluster of EH domain-containing protein 2	EHD2	61 kDa	1	1	1.1	1.1	1.3	1.2	2.3	2.4	0	0

B4DRN8	Cluster of Probable palmitoyltransferase ZDHHC20	ZDHHC20	34 kDa	1	1	0.9	1.2	1.4	1.7	2.2	2.4	0	1
C9J1R1	NF-kappa-B essential modulator	IKBKG	48 kDa	No Va	1	No Value	0.7	No Value	0.7	No Val	0.7	0	0
Q4G0N4	NAD kinase domain-containing protein 1	NADK2	49 kDa	1	No Va	0.8	No Value	1.3	No Value	0.5	No Val	0	0
P49757	Protein numb homolog	NUMB	71 kDa	1	1	0.5	0.4	0.5	0.5	2.1	2.4	0	0
Q7L7X3	Cluster of Serine/threonine-protein kinase TAO1	TAOK1	116 kDa	1	1	0.9	1	1.1	1.3	2	2.4	0	0
Q6P158	Putative ATP-dependent RNA helicase DHX57	DHX57	156 kDa	1	1	0.5	0.4	0.4	0.5	2.9	2.5	0	0
B1AHA8	Heme oxygenase (Decycling) 1 (Fragment)	HMOX1	22 kDa	1	No Va	0.9	No Value	1	No Value	1.8	No Val	0	1
Q12933	TNF receptor-associated factor 2	TRAF2	56 kDa	No Va	1	No Value	0.8	No Value	0.7	No Val	0.7	0	0
Q8NFQ8	Torsin-1A-interacting protein 2	TOR1AIP2	51 kDa	1	1	0.8	0.9	0.9	1	2.5	2.5	0	1
Q9BRS2	Serine/threonine-protein kinase RIO1	RIOK1	66 kDa	1	1	1	1.1	1.2	1.2	2.4	2.5	0	0
Q9Y3C7	Mediator of RNA polymerase II transcription subunit 31	MED31	16 kDa	1	1.1	1.7	1.7	1.8	1.8	2.2	2.5	0	0
Q99828	Calcium and integrin-binding protein 1	CIB1	22 kDa	1	1	1.2	1.3	1.3	1.4	2.2	2.5	0	0
Q08AD1	Calmodulin-regulated spectrin-associated protein 2	CAMSAP2	168 kDa	1	1	1	1.1	0.7	1.1	0.6	1.4	0	0
Q7Z6J9	tRNA-splicing endonuclease subunit Sen54	TSEN54	59 kDa	No Va	1	No Value	1	No Value	1	No Val	0.9	0	0
Q15382	GTP-binding protein Rheb	RHEB	20 kDa	1	1	1.6	1.5	1.5	1.3	0.5	0.7	0	0
O43837	Isocitrate dehydrogenase [NAD] subunit beta, mitochondria	IDH3B	42 kDa	1	1	1.2	1	1.3	1	3.4	2.6	0	0
Q8N9V3	WD repeat, SAM and U-box domain-containing protein 1	WDSUB1	53 kDa	1	1	1.3	1.2	1.4	1.3	3.2	2.6	0	0
Q9UPY8	Microtubule-associated protein RP/EB family member 3	MAPRE3	32 kDa	1	1	1.1	1	1.1	1	3.1	2.6	0	0
Q712K3	Ubiquitin-conjugating enzyme E2 R2	UBE2R2	27 kDa	No Va	1	No Value	0.9	No Value	0.9	No Val	1.4	0	0
G5EA48	SLIT-ROBO Rho GTPase activating protein 1, isoform CR	SRGAP1	117 kDa	No Va	1	No Value	1.1	No Value	1.5	No Val	1.5	0	0
Q9H993	UPF0364 protein C6orf211	C6orf211	51 kDa	1	1	0.8	0.9	0.7	0.8	2.8	2.6	0	0
P02675	Fibrinogen beta chain	FGB	56 kDa	1	No Va	0.8	No Value	0.9	No Value	0.4	No Val	1	0
B4DQH9	Ran-binding protein 10	RANBP10	65 kDa	1	1	1.4	1.4	1.6	1.9	2.7	2.6	0	0
O96005	Cleft lip and palate transmembrane protein 1	CLPTM1	76 kDa	1	1	1.4	1.3	1.3	1.3	2.7	2.6	0	1
O60921	Cluster of Checkpoint protein HUS1	HUS1	32 kDa	1	1	1.2	1.3	1.1	1.3	2.7	2.6	0	0
Q9BSB4	Autophagy-related protein 101	ATG101	25 kDa	1	1	1.1	1.1	1.2	1.1	2.6	2.6		
P46100	Transcriptional regulator ATRX	ATRX	283 kDa	1	1	0.5	0.6	0.7	0.7	2.5	2.6	0	0
B4DQ19	GTP-binding protein SAR1a	SAR1A	18 kDa	No Va	1	No Value	1.1	No Value	0.8	No Val	0.7	1	0
P07902	Cluster of Galactose-1-phosphate uridylyltransferase	GALT	43 kDa	1	1	1.7	1.7	1.6	1.7	2.4	2.6	0	0
Q96SU4	Isoform 7 of Oxysterol-binding protein-related protein 9	OSBPL9	81 kDa	1	1	0.7	0.7	0.7	0.8	2.3	2.6	0	0
B7Z3R9	Cluster of Acid phosphatase-like protein 2	ACPL2	53 kDa	1	1	1.1	1.5	1.3	1.8	1.9	2.6	1	0
Q86W50	Methyltransferase-like protein 16	METTL16	64 kDa	No Va	1	No Value	1	No Value	0.8	No Val	0.8	0	0
P56377	AP-1 complex subunit sigma-2	AP1S2	19 kDa	1	1	1.4	1.5	1.4	1.3	1.9	2.6	0	0
B4DLZ9	Cluster of E3 ubiquitin-protein ligase RNF220	RNF220	40 kDa	No Va	No Va	No Value	No Value	No Value	No Value	No Val	No Val	1	0
A1A4S6	Rho GTPase-activating protein 10	ARHGAP10	89 kDa	1	1	1	1.2	1.4	1.3	3.4	2.7	0	0
Q8NC60	Nitric oxide-associated protein 1	NOA1	78 kDa	1	1	1.3	1.2	1.2	1.3	2.6	2.7	2	0
Q9HBU6	Cluster of Ethanolamine kinase 1	ETNK1	51 kDa	1	1	1.5	1.3	1.2	1.2	2.3	2.7	0	1

Q9Y639	Cluster of Neuroplastin	NPTN	44 kDa	1	1	1.6	1.5	1.8	1.5	1.7	2.7	1	1
Q13405	Cluster of 39S ribosomal protein L49, mitochondrial	MRPL49	19 kDa	1	1	0.8	0.8	0.9	0.8	3.6	2.8	0	0
P84022	Cluster of Isoform 2 of Mothers against decapentaplegic ho	SMAD3	43 kDa	1	1	0.9	0.9	1.1	0.9	3.2	2.8	0	0
Q9BRT6	Protein LLP homolog	LLPH	15 kDa	1	1	1.4	1.4	1.2	1.1	3.1	2.8	0	0
P52564	Cluster of Dual specificity mitogen-activated protein kinase	MAP2K6	37 kDa	1	1	1.7	1.8	1.2	1.3	3	2.8	0	0
Q9Y697	Cluster of Isoform Cytoplasmic of Cysteine desulfurase, mi	NFS1	44 kDa	1	1	1.4	1.5	1.4	1.4	2.7	2.8	0	0
E7EPD0	Cluster of Target of Myb protein 1	TOM1	50 kDa	1	1	1.1	1	1.2	1.2	2.6	2.8	0	0
Q8NFF5	Cluster of Isoform 2 of FAD synthase	FLAD1	54 kDa	1	1	1	1	0.8	1	2.3	2.8	0	0
Q9H4L7	Cluster of SWI/SNF-related matrix-associated actin-depend	SMARCAD1	117 kDa	1	1	2.5	3.3	3.2	3.6	2.2	2.8	0	0
O95573	Cluster of Long-chain-fatty-acid--CoA ligase 3	ACSL3	80 kDa	1	1	0.8	0.6	1.3	1	3.5	2.9	0	1
H0Y621	Cluster of Endoplasmic reticulum-Golgi intermediate comp	ERGIC3	37 kDa	1	No Va	0.8	No Value	0.8	No Value	0.5	No Val	0	1
P62760	Cluster of Visinin-like protein 1	VSNL1	22 kDa	1	No Va	1.2	No Value	1	No Value	2.9	No Val	0	0
Q9NZD2	Glycolipid transfer protein	GLTP	24 kDa	1	No Va	0.7	No Value	0.8	No Value	1	No Val	0	0
Q5JTD0	Tight junction-associated protein 1	TJAP1	62 kDa	1	1	1.3	1.3	1.6	1.5	2.9	2.9	0	0
B4DW92	Nuclear receptor 2C2-associated protein	NR2C2AP	19 kDa	1	1	0.9	0.8	1.1	1	2.9	2.9	0	0
C9JKL2	MLN64 N-terminal domain homolog	STARD3NL	25 kDa	1	1	1.6	1.6	1.3	1.3	3.1	3	0	1
O14732	Inositol monophosphatase 2	IMPA2	31 kDa	1	1	1.1	1.2	1.2	1.3	3	3	0	0
P08123	Collagen alpha-2(I) chain	COL1A2	129 kDa	1	1	1.1	1.3	1.2	1.3	2.9	3	3	0
Q8IWE4	DCN1-like protein 3	DCUN1D3	34 kDa	1	1	2.1	2	1.8	1.5	2.7	3	0	0
Q9UN36	Protein NDRG2	NDRG2	41 kDa	1	1	1.1	1.2	1.2	1.5	2.5	3	0	1
Q15006	ER membrane protein complex subunit 2	EMC2	35 kDa	1	1	1.4	1.3	1.3	1.3	2.5	3	0	0
Q9NS98	Semaphorin-3G	SEMA3G	87 kDa	1	1	0.6	0.8	0.9	0.5	0.9	1.1	1	0
F5H6C8	Mitochondrial antiviral-signaling protein	MAVS	40 kDa	1	1	0.8	0.8	1	0.9	3.6	3.1	0	1
Q7Z4Q2	HEAT repeat-containing protein 3	HEATR3	75 kDa	No Va	1	No Value	1.4	No Value	1.2	No Val	0.9	0	0
Q96JM3	Chromosome alignment-maintaining phosphoprotein 1	CHAMP1	89 kDa	1	No Va	1.2	No Value	1	No Value	1.5	No Val	0	0
C9JXZ5	Vesicle-associated membrane protein 8	VAMP8	9 kDa	No Va	1	No Value	0.9	No Value	0.8	No Val	0.5	0	1
Q15031	Probable leucine--tRNA ligase, mitochondrial	LARS2	102 kDa	1	1	1.4	1.6	1.4	1.5	3.4	3.1	0	0
F8W785	Golgi integral membrane protein 4	GOLIM4	79 kDa	1	1	1.1	0.9	1	0.9	3	3.1	0	1
Q9Y2R0	Cytochrome C oxidase assembly factor 3 homolog, mitocho	COA3	12 kDa	No Va	1	No Value	0.7	No Value	1	No Val	0.5	0	1
Q9H5V9	UPF0428 protein CXorf56	CXorf56	26 kDa	1	No Va	1	No Value	1.2	No Value	1.1	No Val	0	0
Q8TF74	WAS/WASL-interacting protein family member 2	WIPF2	46 kDa	1	1	0.5	0.7	0.8	1	2.1	3.1	0	0
O15382	Branched-chain-amino-acid aminotransferase, mitochondria	BCAT2	44 kDa	1	1	1.4	0.9	0.9	1.3	0.7	1.2	0	0
Q9HBH0	Rho-related GTP-binding protein RhoF	RHOF	24 kDa	1	1	0.9	0.8	1.1	0.9	0.7	0.5	0	0
Q8N4J0	UPF0586 protein C9orf41	C9orf41	47 kDa	1	No Va	0.8	No Value	0.8	No Value	1	No Val	0	0
J3KQQ3	Zinc finger and BTB domain-containing protein 7B	ZBTB7B	62 kDa	1	1	0.3	0.3	0.6	0.5	3.1	3.2	0	0
Q99622	Protein C10	C12orf57	13 kDa	1	1	0.8	0.8	0.7	0.8	2.9	3.2	0	0
Q9UIV1	CCR4-NOT transcription complex subunit 7	CNOT7	33 kDa	1	1	1.2	1.4	1.4	1.3	2.8	3.2	0	0

G3XAG8	Platelet-derived growth factor beta polypeptide (Simian sar	PDGFB	26 kDa	1	1	0.7	0.7	0.7	0.9	0.5	0.7	1	0
C9J0I9	Nuclear-interacting partner of ALK	ZC3HC1	51 kDa	No Va	1	No Value	1.3	No Valu	2	No Val	1.9	0	0
Q8N0Z6	Tetratricopeptide repeat protein 5	TTC5	49 kDa	1	No Va	1.3	No Value	1.1	No Value	1	No Val	0	0
P53384	Cytosolic Fe-S cluster assembly factor NUBP1	NUBP1	35 kDa	1	1	1.4	1.8	1.3	1.6	0.9	0.7	0	0
P40938	Replication factor C subunit 3	RFC3	41 kDa	No Va	1	No Value	1.2	No Valu	1.3	No Val	0.6	0	0
O00186	Syntaxin-binding protein 3	STXBP3	68 kDa	1	1	1.3	1	1.6	1.1	3.5	3.3	0	0
E9PBB0	PERQ amino acid-rich with GYF domain-containing protei	GIGYF2	149 kDa	1.1	1	1.4	1.5	1.5	1.4	4.2	3.4	0	0
H0YES5	HBS1-like protein (Fragment)	HBS1L	19 kDa	1	1	2.1	1.8	2.1	1.7	4.1	3.4	0	1
P62699	Protein yippee-like 5	YPEL5	14 kDa	1	1	1.1	1.1	1.2	1.2	3.8	3.4	0	0
Q8N5A5	Zinc finger CCCH-type with G patch domain-containing pr	ZGPAT	57 kDa	No Va	1	No Value	1.3	No Valu	1.6	No Val	1	0	0
I3L2Y3	E3 ubiquitin-protein ligase RNF167 (Fragment)	RNF167	23 kDa	1	No Va	0.9	No Value	1.1	No Value	1.3	No Val	1	1
Q8N3E9	1-phosphatidylinositol 4,5-bisphosphate phosphodiesterase	PLCD3	89 kDa	1	1	1.3	1.6	1.1	1.7	3.7	3.4	0	0
Q9NRB3	Carbohydrate sulfotransferase 12	CHST12	48 kDa	1	1	1.4	1.4	1.3	1.3	3.6	3.4	0	1
Q06418	Tyrosine-protein kinase receptor TYRO3	TYRO3	97 kDa	1	No Va	0.9	No Value	0.6	No Value	1	No Val	0	1
P51687	Sulfite oxidase, mitochondrial	SUOX	60 kDa	No Va	1	No Value	0.9	No Valu	0.9	No Val	1.4	0	0
P19387	DNA-directed RNA polymerase II subunit RPB3	POLR2C	31 kDa	1	1	0.8	0.9	0.8	0.9	3.4	3.4	0	0
O00178	GTP-binding protein 1	GTPBP1	72 kDa	1	1	0.9	0.9	0.9	1	2.7	3.4	0	0
E9PN76	RING finger protein 214	RNF214	58 kDa	1	1	1.2	1.1	0.9	0.9	4.1	3.5	0	0
Q13535	Serine/threonine-protein kinase ATR	ATR	301 kDa	No Va	1	No Value	1.2	No Valu	1.2	No Val	0.6	0	1
C9J180	Ubiquitin-conjugating enzyme E2 E2 (Fragment)	UBE2E2	18 kDa	1	1	5.1	4	5	4	3.9	3.5	0	0
Q8IZ21	Phosphatase and actin regulator 4	PHACTR4	78 kDa	1	No Va	1.1	No Value	1	No Value	1.2	No Val	0	0
E9PB30	Anoctamin	ANO6	104 kDa	1	1	3	3.2	5.4	5.9	3.3	3.5	0	1
H7C2J9	26S proteasome non-ATPase regulatory subunit 4 (Fragmer	PSMD4	4 kDa	No Va	1	No Value	1	No Valu	1.1	No Val	0.4	0	0
O95965	Integrin beta-like protein 1	ITGBL1	54 kDa	No Va	1	No Value	0.7	No Valu	0.9	No Val	0.8	1	0
Q9H029	GTP-binding protein SAR1b	DKFZp434B2	15 kDa	1	No Va	1.2	No Value	1.3	No Value	0.6	No Values		
Q9H1K1	Iron-sulfur cluster assembly enzyme ISCU, mitochondrial	ISCU	18 kDa	1	1	2.1	2.2	2	2.3	3.3	3.5	0	0
Q9H269	Cluster of Vacuolar protein sorting-associated protein 16 hc	VPS16	95 kDa	1	1	1.2	1.3	1	1.1	3	3.5	0	0
I3L1I0	Glyoxalase domain-containing protein 4 (Fragment)	GLOD4	13 kDa	1	1	1	0.8	0.8	0.8	4	3.6	1	0
H7C2C7	Cluster of Negative elongation factor A (Fragment)	NELFA	27 kDa	1	1	1.1	1	1.3	1.2	3.7	3.6	0	0
H0Y368	Cluster of Dolichol-phosphate mannosyltransferase (Fragm	DPM1	33 kDa	1	1	1.5	1.2	1.1	1.1	2.2	3.6	0	0
Q5R3F8	Protein phosphatase 1 regulatory subunit 29	ELFN2	90 kDa	1	1	1.3	1.2	1.1	1.1	3.7	3.8	0	1
J3KR35	Coiled-coil domain containing 12, isoform CRA_a	CCDC12	21 kDa	1	1	1.2	1.3	1.5	1.3	3.6	3.8	0	0
Q16254	Transcription factor E2F4	E2F4	44 kDa	1	1	1.4	1.3	2	1.7	4.3	3.9	0	0
A8MVZ6	BICD1 protein	BICD1	95 kDa	No Va	1	No Value	2.9	No Valu	2.2	No Val	1.5	0	0
B1AKU4	Cluster of Kinesin-associated protein 3	KIFAP3	87 kDa	No Va	No Va	No Value	No Value	No Valu	No Value	No Val	No Val	0	0
Q15050	Ribosome biogenesis regulatory protein homolog	RRS1	41 kDa	No Va	1	No Value	0.9	No Valu	1.2	No Val	1	0	0
E9PQG7	MLL cleavage product N320	KMT2A	432 kDa	No Va	1	No Value	0.8	No Valu	0.9	No Val	0.4	0	0

H0YDV2	Coiled-coil domain-containing protein 73 (Fragment)	CCDC73	13 kDa	No Va	1	No Value	1	No Value	0.7	No Val	0.9	0	0
P12036	Neurofilament heavy polypeptide	NEFH	112 kDa	1	No Va	0.5	No Value	0.8	No Value	0.7	No Val	0	0
J3QQZ1	Sodium channel protein type 4 subunit alpha	SCN4A	208 kDa	1	1	0.9	0.8	1.2	1.1	4.2	3.9	0	1
Q9H7D0	Dedicator of cytokinesis protein 5	DOCK5	215 kDa	1	No Va	1.1	No Value	1	No Value	0.8	No Val	0	0
Q7Z4H7	Isoform 3 of HAUS augmin-like complex subunit 6	HAUS6	104 kDa	No Va	1	No Value	1.2	No Value	0.9	No Val	0.7	0	0
F8VUJ3	Cluster of Protein POC1B-GALNT4	POC1B-GALN	66 kDa	1	1	1	1.2	1.8	1.2	4.6	4	0	0
O14893	Gem-associated protein 2	GEMIN2	32 kDa	No Va	1	No Value	1.1	No Value	1	No Val	1.4	0	0
Q9NRL2	Bromodomain adjacent to zinc finger domain protein 1A	BAZ1A	179 kDa	1	1	1.1	1.1	1	1.1	4.5	4	0	0
P61966	AP-1 complex subunit sigma-1A	AP1S1	19 kDa	1	No Va	0.5	No Value	1.3	No Value	1.1	No Val	0	0
Q9H4L5	Oxysterol-binding protein-related protein 3	OSBPL3	101 kDa	1	No Va	0.7	No Value	1.1	No Value	0.3	No Val	0	0
F5GX82	Cluster of Protein furry homolog-like	FRYL	340 kDa	No Va	1	No Value	1	No Value	0.9	No Val	0.5	0	0
H7C5I8	Cluster of Down syndrome critical region protein 3 (Fragme	DSCR3	9 kDa	1	1	0.8	0.8	1.1	1	3.7	4	0	0
B1AL05	Cluster of 39S ribosomal protein L43, mitochondrial	MRPL43	21 kDa	1	1	0.7	0.5	0.8	0.4	0.5	0.4	0	0
C9J973	Cluster of NHL repeat-containing protein 3	NHLRC3	16 kDa	1	No Va	0.9	No Value	1.1	No Value	1.2	No Val	1	0
Q08379	Cluster of Golgin subfamily A member 2	GOLGA2	113 kDa	No Va	1	No Value	1	No Value	1	No Val	1.6	0	0
F8VNP5	Cluster of Retinoic acid receptor RXR-beta	RXRB	43 kDa	No Va	1	No Value	1.3	No Value	0.9	No Val	0.9	0	0
Q9Y232	Cluster of Chromodomain Y-like protein	CDYL	66 kDa	No Va	No Va	No Value	No Value	No Value	No Value	No Val	No Val	0	0
H0YKH6	Cluster of Ceramide synthase 2 (Fragment)	CERS2	27 kDa	1	No Va	1	No Value	0.9	No Value	0.5	No Val	0	1
Q8TDW7	Protocadherin Fat 3	FAT3	506 kDa	1	1	0.9	0.9	1.3	1.7	2.8	4.1	1	1
B4DLT4	Ribosomal protein S6 kinase beta-1	RPS6KB1	53 kDa	1	No Va	1.3	No Value	0.9	No Value	0.9	No Val	0	0
Q9P0J7	E3 ubiquitin-protein ligase KCMF1	KCMF1	42 kDa	No Va	1	No Value	1.3	No Value	1.1	No Val	1.4	0	0
Q9H8W4	Pleckstrin homology domain-containing family F member 2	PLEKHF2	28 kDa	1	No Va	1	No Value	0.6	No Value	0.4	No Val	0	0
Q96JC9	ELL-associated factor 1	EAF1	29 kDa	No Va	1	No Value	0.6	No Value	0.6	No Val	1	0	0
Q8IYS2	Uncharacterized protein KIAA2013	KIAA2013	69 kDa	No Va	1	No Value	1.1	No Value	1.1	No Val	0.7	0	1
Q8TDD1	ATP-dependent RNA helicase DDX54	DDX54	99 kDa	No Va	1	No Value	1.1	No Value	1	No Val	0.8	0	0
F5H774	Reticulon-3	RTN3	101 kDa	1	1	1.2	1.9	1.2	1.1	1.2	0.9	0	1
P83876	Thioredoxin-like protein 4A	TXNL4A	17 kDa	1	No Va	1.4	No Value	0.8	No Value	1.2	No Val	0	0
Q9NXX6	Non-structural maintenance of chromosomes element 4 hon	NSMCE4A	44 kDa	No Va	1	No Value	1	No Value	0.9	No Val	1.6	0	0
B4DUE9	Serine incorporator 3	SERINC3	47 kDa	No Va	1	No Value	1	No Value	1.1	No Val	0.7	0	1
Q7L1V2	Vacuolar fusion protein MON1 homolog B	MON1B	59 kDa	No Va	1	No Value	1.3	No Value	1.5	No Val	1.1	0	0
Q9Y2J4	Angiomotin-like protein 2	AMOTL2	86 kDa	1	No Va	0.9	No Value	1.1	No Value	0.9	No Val	0	0
Q5JVZ5	Engulfment and cell motility 2	ELMO2	82 kDa	No Va	1	No Value	1.2	No Value	1.2	No Val	0.5	0	1
Q96RE7	Nucleus accumbens-associated protein 1	NACC1	57 kDa	No Va	1	No Value	1.1	No Value	1	No Val	1.2	0	0
H0YFP2	WD repeat-containing protein 74 (Fragment)	WDR74	4 kDa	No Va	1	No Value	0.8	No Value	1	No Val	0.3	0	0
P21730	C5a anaphylatoxin chemotactic receptor	C5AR1	39 kDa	No Va	1	No Value	0.9	No Value	1.2	No Val	1.4	0	1
Q9P2K3	REST corepressor 3	RCOR3	56 kDa	1	No Va	0.6	No Value	1	No Value	2	No Val	0	0
P17535	Transcription factor jun-D	JUND	35 kDa	1	No Va	1.8	No Value	2.9	No Value	1.2	No Val	0	0

P35555	Fibrillin-1	FBN1	312 kDa	1	No Va	1.3	No Value	1.4	No Value	0.9	No Val	1	0
E7ESZ7	Cluster of NADH dehydrogenase [ubiquinone] 1 alpha subc	NDUFA10	45 kDa	1	No Va	1	No Value	0.9	No Value	0.5	No Val	1	0
Q5VSL9	Cluster of Protein FAM40A	STRIP1	96 kDa	1	No Va	1.2	No Value	1	No Value	1.1	No Val	0	0
Q8NB90	Cluster of Spermatogenesis-associated protein 5	SPATA5	98 kDa	No V	1	No Value	1.2	No Valu	1	No Val	0.7	0	0
E9PF84	Cluster of Isocitrate dehydrogenase [NAD] subunit gamma,	IDH3G	31 kDa	No V	1	No Value	1	No Valu	1	No Val	1.1	0	0
P62995	Transformer-2 protein homolog beta	TRA2B	34 kDa	1	1	0.7	0.7	0.8	0.8	0.7	0.8	0	0
Q9Y463	Cluster of Dual specificity tyrosine-phosphorylation-regulat	DYRK1B	69 kDa	No V	No Va	No Value	No Value	No Valu	No Value	No Val	No Val	0	0
O95429	BAG family molecular chaperone regulator 4	BAG4	50 kDa	1	1	0.9	0.8	0.9	1	5.7	4.4	0	0
G5EA42	Cluster of Tropomodulin 2 (Neuronal), isoform CRA_a	TMOD2	34 kDa	1	1	1	1	0.9	0.8	4.4	4.4	0	0
P17275	Transcription factor jun-B	JUNB	36 kDa	1	No Va	1.5	No Value	0.9	No Value	0.9	No Val	0	0
Q9UMY4	Sorting nexin-12	SNX12	20 kDa	No V	1	No Value	0.9	No Valu	0.9	No Val	1.1	0	0
H0YHB8	6-phosphofructokinase, muscle type (Fragment)	PFKM	5 kDa	1	No Va	0.8	No Value	1.6	No Value	0.3	No Val	0	0
J3KPQ0	Fibroblast growth factor receptor	FGFR4	80 kDa	No V	1	No Value	1	No Valu	1.4	No Val	1.2	1	1
G3V1Q0	Chromosome 20 open reading frame 31, isoform CRA_c	EDEM2	41 kDa	1	No Va	1.7	No Value	1.9	No Value	1.9	No Val	1	0
Q9ULD0	2-oxoglutarate dehydrogenase-like, mitochondrial	OGDHL	114 kDa	No V	No Va	No Value	No Value	No Valu	No Value	No Val	No Val	0	0
B4DUX6	Calponin-1	CNN1	31 kDa	No V	1	No Value	0.8	No Valu	0.8	No Val	0.2	0	0
Q0IIM8	Isoform 2 of TBC1 domain family member 8B	TBC1D8B	72 kDa	1	No Va	0.9	No Value	1	No Value	0.6	No Val	0	0
H0YHH6	Plasma membrane calcium-transporting ATPase 1 (Fragmen	ATP2B1	19 kDa	No V	1	No Value	1.4	No Valu	1.7	No Val	1.9	0	1

Supplementary Table S2. The quantitative 3103 protein list by iTRAQ-2D-HPLC-MS/MS strategy.

Accession Number	Protein subgroup name	Gene symbol	Molecular Weight	FC (MT/WT)	P-value (MT/WT)	FC (KO/WT)	P-value (KO/WT)	FC (MT/KO)	P-value (MT/KO)
O75909	Isoform 4 of Cyclin-K	CCNK	66 kDa	1.10	0.73	1.35	0.09	0.81	0.56
O94985	Isoform 2 of Calsyntenin-1	CLSTN1	109 kDa	1.53	0.24	0.30	0.00	5.08	0.04
Q9NWV4	Cluster of UPF0587 protein C1orf123	C1orf123	18 kDa	1.95	0.13	2.55	0.18	0.76	0.46
Q9BWU0	Kanadaptin	SLC4A1AP	89 kDa	1.05	0.84	1.10	0.00	0.95	0.84
Q8WWM7	Cluster of Ataxin-2-like protein	ATXN2L	113 kDa	0.75	0.23	0.95	0.50	0.79	0.47
F5GWY5	Podocalyxin	PODXL	59 kDa	1.38	0.29	1.70	0.00	0.81	0.35
P63096	Cluster of Guanine nucleotide-binding protein G(i) subunit a	GNAI1	40 kDa	0.63	0.07	0.75	0.34	0.83	0.60
B4DLW8	Cluster of Probable ATP-dependent RNA helicase DDX5	DDX5	61 kDa	0.63	0.07	0.70	0.00	0.89	0.61
F5H2M7	Cluster of Serine/threonine-protein kinase WNK1	WNK1	305 kDa	1.28	0.35	0.90	0.00	1.42	0.23
F8VPG7	Phosphatidylinositol-binding clathrin assembly protein	PICALM	70 kDa	0.50	0.01	0.85	0.20	0.59	0.07
H7BZT4	Cluster of Uncharacterized protein		11 kDa	0.93	0.68	1.05	0.50	0.88	0.64
J3KQQ3	Zinc finger and BTB domain-containing protein 7B	ZBTB7B	62 kDa	0.43	0.00	3.15	0.01	0.13	0.00
G3XAD8	Cluster of Stress-induced-phosphoprotein 1	STIP1	68 kDa	1.05	0.80	0.30	0.00	3.50	0.03
Q13257	Mitotic spindle assembly checkpoint protein MAD2A	MAD2L1	24 kDa	1.65	0.11	1.00	1.00	1.65	0.11
G3XAI2	Cluster of Laminin subunit beta-1	LAMB1	200 kDa	0.68	0.07	0.45	0.06	1.50	0.28
E9PB30	Anoctamin	ANO6	104 kDa	4.38	0.02	3.40	0.03	1.29	0.43
B5ME19	Eukaryotic translation initiation factor 3 subunit C	EIF3CL	105 kDa	1.15	0.50	0.40	0.00	2.88	0.03
Q86XP3	ATP-dependent RNA helicase DDX42	DDX42	103 kDa	1.18	0.43	0.50	0.00	2.35	0.04
Q02880	Cluster of DNA topoisomerase 2-beta	TOP2B	183 kDa	1.05	0.79	0.75	0.34	1.40	0.33
O95573	Cluster of Long-chain-fatty-acid--CoA ligase 3	ACSL3	80 kDa	0.93	0.65	3.20	0.09	0.29	0.00
Q9H270	Vacuolar protein sorting-associated protein 11 homolog	VPS11	108 kDa	1.13	0.54	1.25	0.13	0.90	0.67
Q8TDW7	Protocadherin Fat 3	FAT3	506 kDa	1.20	0.37	3.45	0.17	0.35	0.01
F5H8J2	Cluster of Protein disulfide-isomerase	P4HB	51 kDa	0.75	0.13	0.25	0.04	3.00	0.05
Q08945	Cluster of FACT complex subunit SSRP1	SSRP1	81 kDa	1.05	0.78	0.35	0.05	3.00	0.05
P53396	Cluster of ATP-citrate synthase	ACLY	121 kDa	1.10	0.60	0.30	0.00	3.67	0.02
P30041	Peroxiredoxin-6	PRDX6	25 kDa	1.50	0.12	0.55	0.07	2.73	0.05
Q96Q11	CCA tRNA nucleotidyltransferase 1, mitochondrial	TRNT1	50 kDa	1.03	0.89	0.85	0.50	1.21	0.53
P13010	X-ray repair cross-complementing protein 5	XRCC5	83 kDa	0.70	0.07	0.50	0.13	1.40	0.31
P28799	Granulins	GRN	64 kDa	1.23	0.32	0.50	0.00	2.45	0.03
Q13616	Cullin-1	CUL1	90 kDa	1.23	0.32	0.45	0.06	2.72	0.05
P18065	Cluster of Insulin-like growth factor-binding protein 2	IGFBP2	35 kDa	1.23	0.32	0.45	0.06	2.72	0.05
Q14512	Fibroblast growth factor-binding protein 1	FGFBP1	26 kDa	1.20	0.35	0.65	0.09	1.85	0.12
P11142	Cluster of Heat shock cognate 71 kDa protein	HSPA8	71 kDa	0.60	0.02	0.25	0.04	2.40	0.07
Q504X1	ARHGAP12 protein	ARHGAP12	96 kDa	0.60	0.02	0.90	0.50	0.67	0.12

Q15819	Ubiquitin-conjugating enzyme E2 variant 2	UBE2V2	16 kDa	0.95	0.75	1.05	0.50	0.90	0.67
Q05639	Elongation factor 1-alpha 2	EEF1A2	50 kDa	0.63	0.03	0.70	0.20	0.89	0.65
P50570	Cluster of Dynamin-2	DNM2	98 kDa	1.30	0.22	0.55	0.07	2.36	0.06
B8ZZG1	MAGUK p55 subfamily member 6	MPP6	49 kDa	1.35	0.18	1.20	0.00	1.13	0.51
Q92696	Cluster of Geranylgeranyl transferase type-2 subunit alpha	RABGGTA	65 kDa	1.18	0.39	0.80	0.30	1.47	0.24
Q9Y6Q5	AP-1 complex subunit mu-2	AP1M2	48 kDa	0.85	0.32	0.65	0.26	1.31	0.39
Q13045	Cluster of Protein flightless-1 homolog	FLII	145 kDa	1.25	0.27	0.85	0.20	1.47	0.22
P29992	Cluster of Guanine nucleotide-binding protein subunit alpha-	GNA11	42 kDa	1.00	1.00	0.80	0.30	1.25	0.43
Q7LBC6	Cluster of Lysine-specific demethylase 3B	KDM3B	192 kDa	1.00	1.00	1.20	0.00	0.83	0.27
Q6DKJ4	Cluster of Nucleoredoxin	NXN	48 kDa	1.13	0.50	1.00	1.00	1.13	0.65
B1AK64	Chromatin modification-related protein MEAF6	MEAF6	22 kDa	0.80	0.18	1.20	0.30	0.67	0.10
P27986	Cluster of Phosphatidylinositol 3-kinase regulatory subunit a	PIK3R1	84 kDa	0.80	0.18	1.30	0.20	0.62	0.05
Q9Y5P6	Mannose-1-phosphate guanyltransferase beta	GMPPB	40 kDa	1.08	0.66	1.10	0.50	0.98	0.92
Q15365	Cluster of Poly(rC)-binding protein 1	PCBP1	37 kDa	0.45	0.00	0.65	0.26	0.69	0.21
Q8NHW5	Cluster of 60S acidic ribosomal protein P0-like	RPLP0P6	34 kDa	1.13	0.49	0.55	0.07	2.05	0.08
P07910	Cluster of Heterogeneous nuclear ribonucleoproteins C1/C2	HNRNPC	34 kDa	0.73	0.08	0.55	0.07	1.32	0.33
P42285	Cluster of Superkiller viralicidic activity 2-like 2	SKIV2L2	118 kDa	0.73	0.08	0.60	0.00	1.21	0.31
Q99598	Cluster of Translin-associated protein X	TSNAX	33 kDa	0.93	0.61	0.85	0.50	1.09	0.75
P53618	Coatomer subunit beta	COPB1	107 kDa	0.50	0.01	0.40	0.00	1.25	0.25
Q15427	Splicing factor 3B subunit 4	SF3B4	44 kDa	0.98	0.87	1.05	0.50	0.93	0.74
Q06587	Isoform 2 of E3 ubiquitin-protein ligase RING1	RING1	39 kDa	1.18	0.37	1.40	0.16	0.84	0.43
P07355	Cluster of Isoform 2 of Annexin A2	ANXA2	40 kDa	1.25	0.25	0.20	0.00	6.25	0.01
Q13724	Cluster of Mannosyl-oligosaccharide glucosidase	MOGS	92 kDa	1.30	0.20	1.15	0.50	1.13	0.63
Q9UBV4	Protein Wnt-16	WNT16	41 kDa	1.20	0.32	1.05	0.50	1.14	0.59
H9KV70	Neutrophil gelatinase-associated lipocalin	LCN2	23 kDa	0.85	0.30	1.10	0.50	0.77	0.26
Q13162	Cluster of Peroxiredoxin-4	PRDX4	31 kDa	1.40	0.13	0.95	0.50	1.47	0.20
Q14152	Eukaryotic translation initiation factor 3 subunit A	EIF3A	167 kDa	0.95	0.73	0.45	0.06	2.11	0.07
Q16851	Cluster of UTP--glucose-1-phosphate uridylyltransferase	UGP2	57 kDa	0.95	0.73	0.60	0.00	1.58	0.08
P11441	Ubiquitin-like protein 4A	UBL4A	18 kDa	1.08	0.65	0.80	0.30	1.34	0.30
Q8TF74	WAS/WASL-interacting protein family member 2	WIPF2	46 kDa	0.75	0.10	2.60	0.19	0.29	0.01
O00220	Tumor necrosis factor receptor superfamily member 10A	TNFRSF10A	50 kDa	1.05	0.75	1.55	0.17	0.68	0.10
B7Z6B8	Cluster of 2,4-dienoyl-CoA reductase, mitochondrial	DECR1	35 kDa	1.35	0.15	1.45	0.32	0.93	0.77
Q16630	Cluster of Cleavage and polyadenylation specificity factor su	CPSF6	59 kDa	1.35	0.15	1.05	0.80	1.29	0.36
P60468	Protein transport protein Sec61 subunit beta	SEC61B	10 kDa	1.30	0.19	1.05	0.87	1.24	0.46
B7Z642	Neuronal membrane glycoprotein M6-a	GPM6A	30 kDa	1.40	0.13	1.05	0.80	1.33	0.31
P01033	Cluster of Metalloproteinase inhibitor 1	TIMP1	23 kDa	1.25	0.24	0.65	0.09	1.92	0.08
P68402	Cluster of Platelet-activating factor acetylhydrolase IB subur	PAFAH1B2	26 kDa	0.90	0.47	1.20	0.00	0.75	0.09

E2QRD5	Protein C15orf38-AP3S2	C15orf38-AP3S	44 kDa	0.90	0.47	1.55	0.06	0.58	0.03
O60306	Intron-binding protein aquarius	AQR	171 kDa	1.15	0.41	0.90	0.50	1.28	0.36
Q15018	BRISC complex subunit Abro1	FAM175B	47 kDa	1.15	0.41	1.30	0.20	0.88	0.57
O95834	Isoform 2 of Echinoderm microtubule-associated protein-like	EML2	86 kDa	0.93	0.59	0.60	0.00	1.54	0.08
Q9Y266	Nuclear migration protein nudC	NUDC	38 kDa	0.80	0.16	0.55	0.07	1.45	0.20
P51991	Cluster of Heterogeneous nuclear ribonucleoprotein A3	HNRNPA3	40 kDa	0.80	0.16	0.70	0.00	1.14	0.42
E9PDM8	Cluster of Protein transport protein Sec24D	SEC24D	75 kDa	2.15	0.03	0.90	0.50	2.39	0.05
Q99959	Plakophilin-2	PKP2	97 kDa	0.83	0.21	1.10	0.00	0.75	0.09
B5MCA4	Cluster of Epithelial cell adhesion molecule	EPCAM	38 kDa	0.83	0.21	0.70	0.20	1.18	0.52
C0IMW5	SWI/SNF-related matrix-associated actin-dependent regulato	SMARCE1	38 kDa	1.03	0.87	1.20	0.50	0.85	0.51
P38919	Cluster of Eukaryotic initiation factor 4A-III	EIF4A3	47 kDa	1.03	0.87	0.30	0.00	3.42	0.01
Q8NFD5	AT-rich interactive domain-containing protein 1B	ARID1B	236 kDa	1.10	0.55	1.15	0.50	0.96	0.84
F5H2H7	Semaphorin-3B	SEMA3B	83 kDa	1.10	0.55	1.05	0.50	1.05	0.83
P16885	1-phosphatidylinositol 4,5-bisphosphate phosphodiesterase g	PLCG2	148 kDa	1.40	0.12	0.75	0.13	1.87	0.08
Q13242	Serine/arginine-rich splicing factor 9	SRSF9	26 kDa	1.08	0.64	0.90	0.00	1.19	0.31
E9PFM1	Cluster of Eukaryotic translation initiation factor 4 gamma 1	EIF4G1	176 kDa	1.08	0.64	0.50	0.00	2.15	0.03
Q9NQZ5	StAR-related lipid transfer protein 7, mitochondrial	STARD7	43 kDa	4.08	0.01	2.00	0.00	2.04	0.03
F8VUJ3	Cluster of Protein POC1B-GALNT4	POC1B-GALN	66 kDa	1.30	0.18	4.30	0.06	0.30	0.00
P26583	Cluster of High mobility group protein B2	HMGB2	24 kDa	0.65	0.03	0.40	0.00	1.63	0.06
P55786	Cluster of Puromycin-sensitive aminopeptidase	NPEPPS	103 kDa	0.78	0.12	0.45	0.06	1.72	0.11
E7ERF4	Adenylosuccinate lyase	ADSL	56 kDa	1.13	0.46	1.00	1.00	1.13	0.65
P01031	Complement C5	C5	188 kDa	1.25	0.23	0.75	0.13	1.67	0.12
Q99459	Cell division cycle 5-like protein	CDC5L	92 kDa	1.43	0.11	1.45	0.32	0.98	0.94
Q15056	Eukaryotic translation initiation factor 4H	EIF4H	27 kDa	1.43	0.11	1.30	0.00	1.10	0.56
P08581	Cluster of Hepatocyte growth factor receptor	MET	156 kDa	1.23	0.25	0.55	0.07	2.23	0.05
P24928	DNA-directed RNA polymerase II subunit RPB1	POLR2A	217 kDa	0.73	0.06	1.15	0.50	0.63	0.07
P61604	Cluster of 10 kDa heat shock protein, mitochondrial	HSPE1	11 kDa	1.15	0.39	0.65	0.09	1.77	0.09
O00267	Transcription elongation factor SPT5	SUPT5H	121 kDa	1.15	0.39	0.65	0.09	1.77	0.09
P11047	Laminin subunit gamma-1	LAMC1	178 kDa	0.70	0.05	0.25	0.04	2.80	0.03
P14854	Cytochrome c oxidase subunit 6B1	COX6B1	10 kDa	1.40	0.12	1.05	0.80	1.33	0.29
P10586	Cluster of Receptor-type tyrosine-protein phosphatase F	PTPRF	213 kDa	0.70	0.05	0.30	0.00	2.33	0.02
Q13217	DnaJ homolog subfamily C member 3	DNAJC3	58 kDa	0.70	0.05	0.60	0.00	1.17	0.35
F8VXB4	Cluster of Keratin, type II cytoskeletal 8	KRT8	57 kDa	1.40	0.12	0.20	0.00	7.00	0.01
B4DRN8	Cluster of Probable palmitoyltransferase ZDHHC20	ZDHHC20	34 kDa	1.30	0.17	2.30	0.05	0.57	0.02
Q92917	G patch domain and KOW motifs-containing protein	GPKOW	52 kDa	0.90	0.45	1.30	0.00	0.69	0.04
P06396	Cluster of Isoform 2 of Gelsolin	GSN	81 kDa	1.03	0.86	0.35	0.05	2.93	0.03
P46781	40S ribosomal protein S9	RPS9	23 kDa	1.03	0.86	0.70	0.00	1.46	0.09

P25398	40S ribosomal protein S12	RPS12	15 kDa	1.73	0.05	0.70	0.00	2.46	0.02
Q02487	Desmocollin-2	DSC2	100 kDa	0.98	0.85	1.05	0.50	0.93	0.71
Q00325	Phosphate carrier protein, mitochondrial	SLC25A3	40 kDa	1.08	0.62	0.70	0.00	1.54	0.07
Q9UBR2	Cathepsin Z	CTSZ	34 kDa	1.08	0.62	0.80	0.00	1.34	0.14
Q12904	Aminoacyl tRNA synthase complex-interacting multifunctio	AIMP1	34 kDa	0.75	0.08	0.85	0.20	0.88	0.53
P00488	Coagulation factor XIII A chain	F13A1	83 kDa	0.75	0.08	1.65	0.05	0.45	0.00
Q9ULC5	Long-chain-fatty-acid--CoA ligase 5	ACSL5	76 kDa	0.75	0.08	1.30	0.00	0.58	0.01
B7ZC38	Endophilin-B2	SH3GLB2	44 kDa	1.60	0.06	1.60	0.30	1.00	1.00
Q16181	Cluster of Septin-7	SEPT7	51 kDa	1.40	0.11	0.60	0.00	2.33	0.02
Q7KZF4	Staphylococcal nuclease domain-containing protein 1	SND1	102 kDa	1.18	0.33	0.65	0.26	1.81	0.10
G3V3M6	Cluster of DNA-(apurinic or apyrimidinic site) lyase (Fragm	APEX1	29 kDa	0.68	0.03	0.45	0.06	1.50	0.16
P07195	Cluster of L-lactate dehydrogenase B chain	LDHB	37 kDa	0.68	0.03	4.90	0.03	0.14	0.00
P06748	Cluster of Nucleophosmin	NPM1	33 kDa	1.73	0.04	0.35	0.05	4.93	0.01
Q15293	Cluster of Reticulocalbin-1	RCN1	39 kDa	1.05	0.73	0.65	0.26	1.62	0.14
Q96C86	m7GpppX diphosphatase	DCPS	39 kDa	1.05	0.73	0.60	0.16	1.75	0.10
Q8WXX5	DnaJ homolog subfamily C member 9	DNAJC9	30 kDa	1.05	0.73	0.85	0.20	1.24	0.38
Q14766	Cluster of Latent-transforming growth factor beta-binding pr	LTBP1	187 kDa	1.28	0.18	0.95	0.80	1.34	0.27
I3L291	Uncharacterized protein		59 kDa	0.95	0.70	1.25	0.50	0.76	0.27
Q99417	C-Myc-binding protein	MYCBP	12 kDa	0.95	0.70	0.85	0.20	1.12	0.61
P25705	ATP synthase subunit alpha, mitochondrial	ATP5A1	60 kDa	0.95	0.70	0.70	0.20	1.36	0.26
Q96GG9	Cluster of DCN1-like protein 1	DCUN1D1	30 kDa	0.95	0.70	1.00	1.00	0.95	0.80
Q96MW5	Conserved oligomeric Golgi complex subunit 8	COG8	68 kDa	0.80	0.14	1.15	0.20	0.70	0.08
P12956	X-ray repair cross-complementing protein 6	XRCC6	70 kDa	1.25	0.21	4.20	0.14	0.30	0.00
Q08752	Cluster of Peptidyl-prolyl cis-trans isomerase D	PPID	41 kDa	1.20	0.27	0.80	0.30	1.50	0.16
Q9H4M9	Cluster of EH domain-containing protein 1	EHD1	61 kDa	1.20	0.27	0.55	0.07	2.18	0.04
Q9Y3A3	MOB-like protein phocein	MOB4	26 kDa	1.20	0.27	1.00	1.00	1.20	0.27
Q8NCA5	Cluster of Protein FAM98A	FAM98A	55 kDa	1.00	1.00	0.90	0.70	1.11	0.67
Q9Y678	Cluster of Coatomer subunit gamma-1	COPG1	98 kDa	1.00	1.00	0.60	0.00	1.67	0.05
P42345	Serine/threonine-protein kinase mTOR	MTOR	289 kDa	1.08	0.61	1.45	0.20	0.74	0.16
Q96S44	TP53-regulating kinase	TP53RK	28 kDa	1.08	0.61	1.10	0.70	0.98	0.92
Q96RS6	NudC domain-containing protein 1	NUDCD1	67 kDa	1.08	0.61	0.85	0.20	1.26	0.32
Q8NBZ7	Cluster of UDP-glucuronic acid decarboxylase 1	UXS1	48 kDa	1.08	0.61	0.95	0.50	1.13	0.57
P27694	Cluster of Replication protein A 70 kDa DNA-binding subur	RPA1	68 kDa	0.78	0.10	0.70	0.20	1.11	0.65
O94888	UBX domain-containing protein 7	UBXN7	55 kDa	1.30	0.15	1.20	0.30	1.08	0.70
Q5VU97	VWFA and cache domain-containing protein 1	CACHD1	142 kDa	1.28	0.17	0.95	0.50	1.34	0.24
P49736	Cluster of DNA replication licensing factor MCM2	MCM2	102 kDa	0.98	0.85	0.65	0.09	1.50	0.15
Q9Y6N5	Cluster of Sulfide:quinone oxidoreductase, mitochondrial	SQRDL	50 kDa	0.98	0.85	1.45	0.20	0.67	0.08

B4DUC8	Cluster of Purine nucleoside phosphorylase A	MTAP	33 kDa	1.33	0.14	0.60	0.16	2.21	0.04
P20042	Eukaryotic translation initiation factor 2 subunit 2	EIF2S2	38 kDa	1.38	0.11	0.60	0.16	2.29	0.04
P51784	Ubiquitin carboxyl-terminal hydrolase 11	USP11	110 kDa	1.80	0.03	1.20	0.30	1.50	0.14
O15264	Cluster of Mitogen-activated protein kinase 13	MAPK13	42 kDa	0.90	0.42	1.05	0.50	0.86	0.41
D6RF12	Cluster of Phosphoacetylglucosamine mutase	PGM3	62 kDa	0.90	0.42	0.90	0.00	1.00	1.00
Q14703	Membrane-bound transcription factor site-1 protease	MBTPS1	118 kDa	0.90	0.42	0.85	0.20	1.06	0.78
P62263	40S ribosomal protein S14	RPS14	16 kDa	1.25	0.19	0.90	0.00	1.39	0.10
Q8IXK2	Polypeptide N-acetylgalactosaminyltransferase 12	GALNT12	67 kDa	0.93	0.55	1.35	0.09	0.69	0.07
P26639	Cluster of Threonine--tRNA ligase, cytoplasmic	TARS	83 kDa	0.53	0.00	0.30	0.00	1.75	0.04
A6NGP5	Cluster of Hematological and neurological-expressed 1-like		HN1L	19 kDa	1.95	0.03	1.25	0.50	1.56	0.14
P12277	Cluster of Creatine kinase B-type	CKB	43 kDa	1.53	0.06	0.75	0.34	2.03	0.05
G5EA52	Cluster of Protein disulfide isomerase family A, member 3, i	PDIA3	55 kDa	0.88	0.31	0.25	0.04	3.50	0.02
A6NGV6	Aryl hydrocarbon receptor nuclear translocator	ARNT	46 kDa	0.88	0.31	1.65	0.05	0.53	0.01
Q13433	Zinc transporter ZIP6	SLC39A6	85 kDa	0.73	0.05	1.25	0.13	0.58	0.02
Q9BQG0	Cluster of Myb-binding protein 1A	MYBBP1A	149 kDa	1.10	0.50	0.65	0.26	1.69	0.10
Q6IBS0	Cluster of Twinfilin-2	TWF2	40 kDa	0.55	0.01	0.85	0.20	0.65	0.04
C9JFR7	Cytochrome c (Fragment)	CYCS	11 kDa	1.10	0.50	0.95	0.50	1.16	0.49
P55809	Cluster of Succinyl-CoA:3-ketoacid coenzyme A transferase	OXCT1	56 kDa	0.55	0.01	0.80	0.30	0.69	0.09
O43617	Trafficking protein particle complex subunit 3	TRAPPC3	20 kDa	1.23	0.22	1.40	0.30	0.88	0.52
Q6P3W7	SCY1-like protein 2	SCYL2	104 kDa	1.18	0.29	0.65	0.09	1.81	0.07
Q14980	Cluster of Isoform 2 of Nuclear mitotic apparatus protein 1	NUMA1	237 kDa	1.13	0.41	0.30	0.00	3.75	0.01
C9JLU1	DNA-directed RNA polymerases I, II, and III subunit RPAB	POLR2H	17 kDa	1.13	0.41	1.60	0.37	0.70	0.21
Q9BWF3	Cluster of RNA-binding protein 4	RBM4	40 kDa	1.13	0.41	1.35	0.09	0.83	0.32
J9JID7	Lamin B2, isoform CRA_a	LMNB2	70 kDa	1.08	0.59	0.65	0.26	1.65	0.11
Q9NTZ6	RNA-binding protein 12	RBM12	97 kDa	1.08	0.59	0.65	0.09	1.65	0.09
Q9NR50	Translation initiation factor eIF-2B subunit gamma	EIF2B3	50 kDa	1.08	0.59	0.95	0.50	1.13	0.55
Q9NWB6	Cluster of Arginine and glutamate-rich protein 1	ARGLU1	33 kDa	1.08	0.59	1.25	0.50	0.86	0.51
P60891	Cluster of Ribose-phosphate pyrophosphokinase 1	PRPS1	35 kDa	1.43	0.08	1.10	0.00	1.30	0.14
Q14344	Cluster of Guanine nucleotide-binding protein subunit alpha-	GNA13	44 kDa	1.00	1.00	1.05	0.50	0.95	0.79
H7C2Q8	EBNA1 binding protein 2, isoform CRA_d	EBNA1BP2	41 kDa	1.00	1.00	0.90	0.00	1.11	0.45
Q02818	Cluster of Nucleobindin-1	NUCB1	54 kDa	0.50	0.00	0.40	0.00	1.25	0.18
Q12874	Splicing factor 3A subunit 3	SF3A3	59 kDa	1.00	1.00	0.70	0.00	1.43	0.08
H7C463	Cluster of Mitochondrial inner membrane protein (Fragment	IMMT	68 kDa	0.50	0.00	0.70	0.00	0.71	0.04
J3KPV7	Sulfurtransferase	MPST	35 kDa	0.75	0.06	1.65	0.23	0.45	0.01
P36957	Cluster of Dihydrolipoyllysine-residue succinyltransferase c	DLST	49 kDa	0.75	0.06	0.85	0.20	0.88	0.50
P23381	Tryptophan--tRNA ligase, cytoplasmic	WARS	53 kDa	1.15	0.34	0.70	0.20	1.64	0.10
F5H365	Cluster of Protein transport protein Sec23A	SEC23A	83 kDa	1.15	0.34	0.80	0.30	1.44	0.17

Q15029	116 kDa U5 small nuclear ribonucleoprotein component	EFTUD2	109 kDa	0.83	0.16	0.65	0.26	1.27	0.36
Q7LBR1	Charged multivesicular body protein 1b	CHMP1B	22 kDa	0.83	0.16	1.10	0.50	0.75	0.15
C9J1Z8	ADP-ribosylation factor 5 (Fragment)	ARF5	17 kDa	0.80	0.12	1.25	0.50	0.64	0.09
P46977	Dolichyl-diphosphooligosaccharide--protein glycosyltransfer	STT3A	81 kDa	0.80	0.12	1.15	0.20	0.70	0.07
P12270	Cluster of Nucleoprotein TPR	TPR	267 kDa	0.80	0.12	0.30	0.00	2.67	0.01
P00450	Cluster of Ceruloplasmin	CP	122 kDa	0.80	0.12	1.10	0.00	0.73	0.05
J3KN82	Cluster of Probable methylthioribulose-1-phosphate dehydra	APIP	29 kDa	0.80	0.12	1.00	1.00	0.80	0.25
Q8NBP7	Cluster of Proprotein convertase subtilisin/kexin type 9	PCSK9	74 kDa	0.80	0.12	0.35	0.05	2.29	0.03
Q9Y3A5	Ribosome maturation protein SBDS	SBDS	29 kDa	0.80	0.12	0.70	0.00	1.14	0.35
P55145	Mesencephalic astrocyte-derived neurotrophic factor	MANF	21 kDa	0.98	0.84	0.70	0.00	1.39	0.09
Q14204	Cytoplasmic dynein 1 heavy chain 1	DYNC1H1	532 kDa	0.98	0.84	0.30	0.00	3.25	0.01
Q13641	Trophoblast glycoprotein	TPBG	46 kDa	0.98	0.84	1.35	0.26	0.72	0.12
Q9H6U6	Isoform 4 of Breast carcinoma-amplified sequence 3	BCAS3	104 kDa	1.05	0.70	1.85	0.25	0.57	0.05
P40937	Cluster of Replication factor C subunit 5	RFC5	38 kDa	1.05	0.70	1.40	0.30	0.75	0.18
Q14331	Protein FRG1	FRG1	29 kDa	1.05	0.70	1.60	0.11	0.66	0.04
P26368	Splicing factor U2AF 65 kDa subunit	U2AF2	54 kDa	1.05	0.70	1.05	0.50	1.00	1.00
O14773	Tripeptidyl-peptidase 1	TPP1	61 kDa	1.05	0.70	0.90	0.00	1.17	0.30
P52292	Importin subunit alpha-2	KPNA2	58 kDa	1.30	0.13	0.45	0.06	2.89	0.02
G3V1C4	Cluster of Cell division cycle 27, isoform CRA_b	CDC27	93 kDa	1.30	0.13	0.90	0.00	1.44	0.07
P22234	Cluster of Multifunctional protein ADE2	PAICS	47 kDa	0.85	0.22	0.35	0.05	2.43	0.03
H7C1F6	Cluster of ES1 protein homolog, mitochondrial (Fragment)	C21orf33	27 kDa	1.38	0.09	1.25	0.13	1.10	0.62
H6UMI1	Cluster of GABARAP-a	GABARAP	12 kDa	1.18	0.28	0.95	0.50	1.24	0.32
P55769	Cluster of NHP2-like protein 1	NHP2L1	14 kDa	1.18	0.28	1.15	0.20	1.02	0.91
G5E9Y2	Cluster of X-prolyl aminopeptidase (Aminopeptidase P) 1, so	XPNPEP1	75 kDa	2.63	0.01	0.65	0.09	4.04	0.01
F8VX04	Sodium-coupled neutral amino acid transporter 1	SLC38A1	56 kDa	0.93	0.52	0.90	0.00	1.03	0.82
F5H1Z7	Semaphorin-3C	SEMA3C	87 kDa	0.93	0.52	0.65	0.09	1.42	0.16
Q9Y5X2	Sorting nexin-8	SNX8	53 kDa	0.93	0.52	2.25	0.03	0.41	0.00
P23528	Cofilin-1	CFL1	19 kDa	0.93	0.52	0.50	0.00	1.85	0.03
P17050	Alpha-N-acetylgalactosaminidase	NAGA	47 kDa	0.93	0.52	1.05	0.80	0.88	0.53
H7C5L1	Cluster of Prostaglandin E synthase 2 (Fragment)	PTGES2	34 kDa	0.93	0.52	0.95	0.50	0.97	0.88
O95671	N-acetylserotonin O-methyltransferase-like protein	ASMTL	69 kDa	1.10	0.47	1.20	0.30	0.92	0.63
A6NM69	Cluster of Non-specific lipid-transfer protein	SCP2	54 kDa	1.10	0.47	1.25	0.50	0.88	0.56
Q5BKZ1	DBIRD complex subunit ZNF326	ZNF326	66 kDa	1.10	0.47	2.00	0.06	0.55	0.01
Q9P2R7	Isoform 2 of Succinyl-CoA ligase [ADP-forming] subunit be	SUCLA2	48 kDa	1.13	0.39	2.10	0.27	0.54	0.05
I3L1Q2	Cluster of B-cell CLL/lymphoma 7 protein family member C	BCL7C	33 kDa	1.13	0.39	1.10	0.50	1.02	0.91
P68363	Cluster of Tubulin alpha-1B chain	TUBA1B	50 kDa	0.68	0.02	0.30	0.00	2.25	0.02
Q92973	Transportin-1	TNPO1	102 kDa	0.68	0.02	0.60	0.00	1.13	0.39

O95295	SNARE-associated protein Snapin	SNAPIN	15 kDa	0.68	0.02	1.25	0.34	0.54	0.02
E9PAV2	Cluster of Proteasome subunit beta type-5	PSMB5	18 kDa	0.78	0.08	0.70	0.20	1.11	0.63
Q9Y4X5	E3 ubiquitin-protein ligase ARIH1	ARIH1	64 kDa	0.78	0.08	0.70	0.00	1.11	0.44
P46782	40S ribosomal protein S5	RPS5	23 kDa	0.78	0.08	0.80	0.00	0.97	0.79
B5MDF5	GTP-binding nuclear protein Ran	RAN	26 kDa	0.95	0.66	0.80	0.30	1.19	0.42
Q9H2A7	C-X-C motif chemokine 16	CXCL16	28 kDa	0.95	0.66	1.45	0.07	0.66	0.03
P22223	Cluster of Isoform 2 of Cadherin-3	CDH3	87 kDa	0.95	0.66	0.80	0.00	1.19	0.25
Q14019	Coactosin-like protein	COTL1	16 kDa	1.38	0.09	0.65	0.26	2.12	0.04
Q5T6F2	Cluster of Ubiquitin-associated protein 2	UBAP2	117 kDa	1.33	0.11	1.65	0.05	0.80	0.21
J3KNP4	Cluster of Semaphorin-4B	SEMA4B	93 kDa	1.33	0.11	1.15	0.50	1.15	0.50
Q16186	Proteasomal ubiquitin receptor ADRM1	ADRM1	42 kDa	1.53	0.05	1.65	0.14	0.92	0.66
B3KSI9	Cluster of Leucine zipper transcription factor-like 1, isoform	LZTFL1	33 kDa	0.88	0.28	0.75	0.13	1.17	0.44
Q5VW36	Focadhesin	FOCAD	200 kDa	0.88	0.28	1.25	0.13	0.70	0.06
P36578	Cluster of 60S ribosomal protein L4	RPL4	48 kDa	1.03	0.84	0.75	0.13	1.37	0.18
Q9Y294	Cluster of Histone chaperone ASF1A	ASF1A	23 kDa	1.03	0.84	1.45	0.32	0.71	0.13
Q9NVZ3	Adaptin ear-binding coat-associated protein 2	NECAP2	28 kDa	1.03	0.84	2.05	0.15	0.50	0.01
Q13426	DNA repair protein XRCC4	XRCC4	38 kDa	1.03	0.84	0.85	0.20	1.21	0.36
P51178	1-phosphatidylinositol 4,5-bisphosphate phosphodiesterase d	PLCD1	86 kDa	1.00	1.00	1.70	0.18	0.59	0.03
O75688	Cluster of Isoform 4 of Protein phosphatase 1B	PPM1B	42 kDa	1.28	0.14	0.80	0.30	1.59	0.09
Q01130	Cluster of Serine/arginine-rich splicing factor 2	SRSF2	25 kDa	1.28	0.14	1.05	0.50	1.21	0.34
Q9HCE5	Methyltransferase-like protein 14	METTL14	52 kDa	1.20	0.22	1.65	0.05	0.73	0.08
P82909	28S ribosomal protein S36, mitochondrial	MRPS36	11 kDa	1.23	0.19	2.20	0.11	0.56	0.01
Q14558	Cluster of Phosphoribosyl pyrophosphate synthase-associate	PRPSAP1	39 kDa	1.23	0.19	0.75	0.13	1.63	0.08
Q9Y3T9	Nucleolar complex protein 2 homolog	NOC2L	85 kDa	1.23	0.19	1.35	0.09	0.91	0.57
O75643	U5 small nuclear ribonucleoprotein 200 kDa helicase	SNRNP200	245 kDa	1.23	0.19	0.30	0.00	4.08	0.01
E9PNW4	CD59 glycoprotein	CD59	12 kDa	1.45	0.06	1.20	0.50	1.21	0.40
Q05048	Cleavage stimulation factor subunit 1	CSTF1	48 kDa	1.35	0.09	0.70	0.00	1.93	0.02
Q6ZMP0	Thrombospondin type-1 domain-containing protein 4	THSD4	112 kDa	1.78	0.03	2.15	0.08	0.83	0.28
P52294	Cluster of Importin subunit alpha-1	KPNA1	60 kDa	1.25	0.16	0.65	0.09	1.92	0.04
O00186	Syntaxin-binding protein 3	STXBP3	68 kDa	1.25	0.16	3.40	0.03	0.37	0.00
P13639	Elongation factor 2	EEF2	95 kDa	0.98	0.82	0.25	0.04	3.90	0.01
O95707	Ribonuclease P protein subunit p29	POP4	25 kDa	0.98	0.82	1.85	0.04	0.53	0.01
B4DKT0	Golgi reassembly-stacking protein 2	GORASP2	49 kDa	0.98	0.82	1.00	1.00	0.98	0.82
F6S928	Cluster of Mitochondrial import receptor subunit TOM5 hon	TOMM5	10 kDa	1.10	0.45	2.05	0.20	0.54	0.03
Q6P1N9	Isoform 2 of Putative deoxyribonuclease TATDN1	TATDN1	28 kDa	1.10	0.45	1.20	0.00	0.92	0.45
Q8WXI4	Cluster of Isoform 2 of Acyl-coenzyme A thioesterase 11	ACOT11	67 kDa	1.43	0.07	1.40	0.30	1.02	0.93
B7Z3R9	Cluster of Acid phosphatase-like protein 2	ACPL2	53 kDa	1.43	0.07	2.25	0.17	0.63	0.06

Q92673	Sortilin-related receptor	SORL1	248 kDa	0.83	0.13	0.85	0.50	0.97	0.88
Q14677	Cluster of Clathrin interactor 1	CLINT1	68 kDa	0.83	0.13	0.85	0.20	0.97	0.86
Q9HD45	Cluster of Transmembrane 9 superfamily member 3	TM9SF3	68 kDa	1.15	0.30	0.80	0.50	1.44	0.18
O60513	Beta-1,4-galactosyltransferase 4	B4GALT4	40 kDa	1.15	0.30	0.90	0.50	1.28	0.26
F5GZS0	Probable ATP-dependent RNA helicase DHX36	DHX36	113 kDa	1.15	0.30	1.00	1.00	1.15	0.47
P17900	Ganglioside GM2 activator	GM2A	21 kDa	1.15	0.30	0.80	0.00	1.44	0.06
P62333	Cluster of 26S protease regulatory subunit 10B	PSMC6	44 kDa	1.15	0.30	0.60	0.00	1.92	0.02
O43148	mRNA cap guanine-N7 methyltransferase	RNMT	55 kDa	0.73	0.04	0.70	0.20	1.04	0.85
P60983	Cluster of Glia maturation factor beta	GMFB	17 kDa	0.73	0.04	1.35	0.26	0.54	0.01
B7ZKL3	Cluster of EPS8L2 protein	EPS8L2	82 kDa	1.45	0.06	0.85	0.20	1.71	0.06
Q96QD8	Sodium-coupled neutral amino acid transporter 2	SLC38A2	56 kDa	0.73	0.04	0.95	0.80	0.76	0.20
O43805	Sjoegren syndrome nuclear autoantigen 1	SSNA1	14 kDa	0.73	0.04	2.05	0.03	0.35	0.00
P32119	Cluster of Peroxiredoxin-2	PRDX2	22 kDa	0.73	0.04	0.40	0.00	1.81	0.02
Q9UBQ5	Eukaryotic translation initiation factor 3 subunit K	EIF3K	25 kDa	0.73	0.04	0.60	0.00	1.21	0.19
Q92945	Far upstream element-binding protein 2	KHSRP	73 kDa	2.20	0.01	0.50	0.00	4.40	0.00
Q9NS86	LanC-like protein 2	LANCL2	51 kDa	1.28	0.13	1.00	1.00	1.28	0.13
P23284	Peptidyl-prolyl cis-trans isomerase B	PPIB	24 kDa	1.08	0.55	0.50	0.13	2.15	0.03
B8ZZC8	Cluster of Methyltransferase-like protein 5	METTL5	28 kDa	1.08	0.55	1.30	0.20	0.83	0.27
Q5QPL9	Cluster of RNA binding protein, autoantigenic (HnRNP-asso	RALY	25 kDa	0.93	0.49	0.85	0.20	1.09	0.63
Q16394	Cluster of Exostosin-1	EXT1	86 kDa	0.93	0.49	1.00	1.00	0.93	0.49
J3QRU1	Cluster of Tyrosine-protein kinase Yes	YES1	61 kDa	1.20	0.20	0.80	0.30	1.50	0.11
O94826	Mitochondrial import receptor subunit TOM70	TOMM70A	67 kDa	0.80	0.09	1.00	1.00	0.80	0.31
P20290	Cluster of Isoform 2 of Transcription factor BTF3	BTF3	18 kDa	0.80	0.09	0.90	0.50	0.89	0.51
Q8N1G4	Leucine-rich repeat-containing protein 47	LRRC47	63 kDa	0.85	0.18	0.55	0.07	1.55	0.09
Q6IA69	Cluster of Glutamine-dependent NAD(+) synthetase	NADSYN1	79 kDa	0.85	0.18	0.75	0.34	1.13	0.56
P12081	Cluster of Histidine--tRNA ligase, cytoplasmic	HARS	57 kDa	0.85	0.18	0.25	0.04	3.40	0.01
O95340	Bifunctional 3'-phosphoadenosine 5'-phosphosulfate synthas	PAPSS2	70 kDa	0.85	0.18	1.00	1.00	0.85	0.36
P46778	60S ribosomal protein L21	RPL21	19 kDa	0.85	0.18	1.15	0.20	0.74	0.09
Q969S3	Zinc finger protein 622	ZNF622	54 kDa	0.85	0.18	1.25	0.13	0.68	0.04
Q9NZ45	CDGSH iron-sulfur domain-containing protein 1	CISD1	12 kDa	0.85	0.18	1.35	0.09	0.63	0.02
Q9HA65	TBC1 domain family member 17	TBC1D17	73 kDa	0.85	0.18	2.15	0.03	0.40	0.00
O75874	Isocitrate dehydrogenase [NADP] cytoplasmic	IDH1	47 kDa	0.85	0.18	0.50	0.00	1.70	0.03
P14625	Cluster of Endoplasmin	HSP90B1	92 kDa	0.85	0.18	0.30	0.00	2.83	0.01
P78371	T-complex protein 1 subunit beta	CCT2	57 kDa	0.85	0.18	0.30	0.00	2.83	0.01
P40227	Cluster of T-complex protein 1 subunit zeta	CCT6A	58 kDa	0.85	0.18	0.40	0.00	2.13	0.01
P18206	Cluster of Vinculin	VCL	124 kDa	0.85	0.18	0.40	0.11	2.13	0.04
Q9C0B5	Palmitoyltransferase ZDHHC5	ZDHHC5	78 kDa	0.85	0.18	1.70	0.18	0.50	0.01

Q9HCD5	Nuclear receptor coactivator 5	NCOA5	66 kDa	2.18	0.01	0.95	0.50	2.29	0.02
Q9NPF4	Probable tRNA threonylcarbamoyladenosine biosynthesis pr	OSGEP	36 kDa	1.58	0.04	1.00	1.00	1.58	0.04
Q9Y617	Phosphoserine aminotransferase	PSAT1	40 kDa	0.90	0.35	0.45	0.06	2.00	0.03
C9JYY7	Cluster of SEC14-like protein 2 (Fragment)	SEC14L2	10 kDa	0.90	0.35	1.40	0.00	0.64	0.01
O43657	Tetraspanin-6	TSPAN6	28 kDa	0.90	0.35	1.15	0.50	0.78	0.20
O75340	Cluster of Programmed cell death protein 6	PDCD6	22 kDa	0.90	0.35	0.85	0.20	1.06	0.74
C9J5C3	Programmed cell death protein 10 (Fragment)	PDCD10	24 kDa	0.90	0.35	0.95	0.50	0.95	0.74
Q9BYC5	Cluster of Alpha-(1,6)-fucosyltransferase	FUT8	67 kDa	0.90	0.35	1.05	0.50	0.86	0.35
Q96EK5	KIF1-binding protein	KIAA1279	72 kDa	0.90	0.35	0.90	0.00	1.00	1.00
Q9NUP9	Cluster of Protein lin-7 homolog C	LIN7C	22 kDa	0.90	0.35	0.95	0.80	0.95	0.78
Q9BZH6	WD repeat-containing protein 11	WDR11	137 kDa	1.78	0.02	1.05	0.50	1.69	0.06
P48960	CD97 antigen	CD97	92 kDa	0.70	0.02	1.55	0.06	0.45	0.00
Q9Y5B8	Nucleoside diphosphate kinase 7	NME7	42 kDa	0.70	0.02	1.65	0.05	0.42	0.00
Q2L7G6	Heterogeneous nuclear ribonucleoprotein R	HNRNPR	67 kDa	0.70	0.02	0.50	0.00	1.40	0.07
Q96GM5	SWI/SNF-related matrix-associated actin-dependent regulatc	SMARCD1	58 kDa	0.95	0.64	1.05	0.87	0.90	0.66
Q9Y248	DNA replication complex GINS protein PSF2	GINS2	21 kDa	0.48	0.00	1.05	0.80	0.45	0.01
B4E1D7	Pyridoxine-5'-phosphate oxidase	PNPO	28 kDa	0.95	0.64	0.90	0.00	1.06	0.64
Q8IZH2	5'-3' exoribonuclease 1	XRN1	194 kDa	0.95	0.64	1.70	0.00	0.56	0.00
Q9UDY8	Mucosa-associated lymphoid tissue lymphoma translocation	MALT1	92 kDa	0.95	0.64	1.20	0.30	0.79	0.18
Q9BZI7	Regulator of nonsense transcripts 3B	UPF3B	58 kDa	0.95	0.64	1.25	0.13	0.76	0.11
P04004	Vitronectin	VTN	54 kDa	0.63	0.01	0.45	0.06	1.39	0.15
P49720	Proteasome subunit beta type-3	PSMB3	23 kDa	0.63	0.01	0.45	0.06	1.39	0.15
P17987	Cluster of T-complex protein 1 subunit alpha	TCP1	60 kDa	0.63	0.01	0.45	0.06	1.39	0.15
P12004	Proliferating cell nuclear antigen	PCNA	29 kDa	1.03	0.82	0.60	0.16	1.71	0.06
Q9HAV4	Exportin-5	XPO5	136 kDa	1.03	0.82	0.55	0.07	1.86	0.04
Q13620	Cullin-4B	CUL4B	104 kDa	1.03	0.82	0.65	0.09	1.58	0.08
Q08345	Cluster of Epithelial discoidin domain-containing receptor 1	DDR1	101 kDa	1.03	0.82	1.15	0.20	0.89	0.47
Q9H5N1	Rab GTPase-binding effector protein 2	RABEP2	64 kDa	1.03	0.82	1.65	0.05	0.62	0.02
P61225	Cluster of Ras-related protein Rap-2b	RAP2B	21 kDa	1.38	0.07	1.15	0.50	1.20	0.38
P09382	Galectin-1	LGALS1	15 kDa	0.65	0.01	0.30	0.00	2.17	0.01
Q9NZ32	Cluster of Actin-related protein 10	ACTR10	46 kDa	1.30	0.10	0.95	0.50	1.37	0.15
J3QSS4	Formin-binding protein 1-like	FNBP1L	70 kDa	0.65	0.01	0.95	0.50	0.68	0.04
P52657	Transcription initiation factor IIA subunit 2	GTF2A2	12 kDa	0.65	0.01	1.05	0.50	0.62	0.02
J3KMX3	Alpha-fetoprotein	AFP	70 kDa	0.65	0.01	1.45	0.07	0.45	0.00
O00483	NADH dehydrogenase [ubiquinone] 1 alpha subcomplex sub	NDUFA4	9 kDa	0.65	0.01	1.30	0.00	0.50	0.00
J3KQS6	BRISC and BRCA1-A complex member 1	BABAM1	36 kDa	1.33	0.09	1.45	0.07	0.91	0.57
P08195	Cluster of Isoform 2 of 4F2 cell-surface antigen heavy chain	SLC3A2	58 kDa	1.33	0.09	0.50	0.00	2.65	0.01

Q99623	Cluster of Prohibitin-2	PHB2	33 kDa	1.05	0.66	0.55	0.07	1.91	0.03
Q13185	Cluster of Chromobox protein homolog 3	CBX3	21 kDa	1.05	0.66	0.75	0.13	1.40	0.13
P09341	Growth-regulated alpha protein	CXCL1	11 kDa	1.05	0.66	0.75	0.13	1.40	0.13
J3KQA0	Cluster of Synaptotagmin I, isoform CRA_b	SYT1	47 kDa	1.05	0.66	0.85	0.20	1.24	0.28
Q16254	Transcription factor E2F4	E2F4	44 kDa	1.60	0.03	4.10	0.04	0.39	0.00
O75818	Ribonuclease P protein subunit p40	RPP40	42 kDa	1.13	0.34	1.40	0.16	0.80	0.20
P62873	Cluster of Guanine nucleotide-binding protein G(I)/G(S)/G(	GNB1	37 kDa	1.10	0.42	1.10	0.70	1.00	1.00
Q6ZRP7	Cluster of Sulfhydryl oxidase 2	QSOX2	78 kDa	1.10	0.42	0.85	0.50	1.29	0.25
O75351	Vacuolar protein sorting-associated protein 4B	VPS4B	49 kDa	1.10	0.42	1.15	0.50	0.96	0.80
P50897	Palmitoyl-protein thioesterase 1	PPT1	34 kDa	1.10	0.42	0.75	0.13	1.47	0.10
P11766	Alcohol dehydrogenase class-3	ADH5	40 kDa	1.10	0.42	0.85	0.20	1.29	0.20
H0Y8C6	Importin-5 (Fragment)	IPO5	124 kDa	1.10	0.42	0.50	0.00	2.20	0.01
P52564	Cluster of Dual specificity mitogen-activated protein kinase	MAP2K6	37 kDa	1.50	0.04	2.90	0.03	0.52	0.00
P39023	Cluster of 60S ribosomal protein L3	RPL3	46 kDa	1.35	0.08	0.90	0.50	1.50	0.10
B4DII8	Cluster of Gamma-secretase C-terminal fragment 57	APP	85 kDa	0.88	0.24	0.40	0.00	2.19	0.01
P07858	Cathepsin B	CTSB	38 kDa	0.88	0.24	0.50	0.00	1.75	0.02
P38117	Cluster of Electron transfer flavoprotein subunit beta	ETFB	28 kDa	0.88	0.24	1.00	1.00	0.88	0.24
G8JLQ3	Biogenesis of lysosome-related organelles complex 1 subuni	BLOC1S1	9 kDa	0.88	0.24	1.60	0.00	0.55	0.00
F5GYQ1	V-type proton ATPase subunit d 1	ATP6V0D1	45 kDa	0.88	0.24	1.00	1.00	0.88	0.43
Q15691	Microtubule-associated protein RP/EB family member 1	MAPRE1	30 kDa	0.88	0.24	0.65	0.09	1.35	0.16
P34932	Heat shock 70 kDa protein 4	HSPA4	94 kDa	0.78	0.06	0.35	0.05	2.21	0.02
Q9Y490	Cluster of Talin-1	TLN1	270 kDa	0.78	0.06	0.35	0.05	2.21	0.02
P38646	Cluster of Stress-70 protein, mitochondrial	HSPA9	74 kDa	0.78	0.06	0.35	0.05	2.21	0.02
Q13505	Cluster of Metaxin-1	MTX1	51 kDa	0.78	0.06	1.45	0.20	0.53	0.01
Q9UP83	Conserved oligomeric Golgi complex subunit 5	COG5	93 kDa	0.78	0.06	1.25	0.13	0.62	0.02
B4E1F0	Plasma protease C1 inhibitor	SERPING1	56 kDa	0.78	0.06	1.75	0.04	0.44	0.00
F8VQZ7	Cluster of Methionine aminopeptidase	METAP2	53 kDa	0.78	0.06	0.60	0.00	1.29	0.10
P11216	Cluster of Glycogen phosphorylase, brain form	PYGB	97 kDa	0.78	0.06	0.20	0.00	3.88	0.00
P00558	Cluster of Phosphoglycerate kinase 1	PGK1	45 kDa	0.78	0.06	0.30	0.00	2.58	0.01
Q14697	Cluster of Neutral alpha-glucosidase AB	GANAB	107 kDa	0.78	0.06	0.30	0.00	2.58	0.01
O60271	Cluster of C-Jun-amino-terminal kinase-interacting protein 4	SPAG9	146 kDa	0.78	0.06	0.60	0.00	1.29	0.10
P10644	Cluster of cAMP-dependent protein kinase type I-alpha regu	PRKAR1A	43 kDa	0.78	0.06	0.70	0.00	1.11	0.39
Q7Z478	ATP-dependent RNA helicase DHX29	DHX29	155 kDa	0.78	0.06	1.00	1.00	0.78	0.06
F5H5U7	Cluster of Coiled-coil domain-containing protein 132	CCDC132	108 kDa	0.78	0.06	1.50	0.00	0.52	0.00
Q14137	Ribosome biogenesis protein BOP1	BOP1	84 kDa	0.78	0.06	0.90	0.50	0.86	0.38
P40429	Cluster of 60S ribosomal protein L13a	RPL13A	24 kDa	0.78	0.06	0.65	0.09	1.19	0.34
Q8N3E9	1-phosphatidylinositol 4,5-bisphosphate phosphodiesterase d	PLCD3	89 kDa	1.43	0.05	3.55	0.04	0.40	0.00

B4DR80	Cluster of Serine/threonine-protein kinase 24	STK24	46 kDa	1.23	0.15	1.00	1.00	1.23	0.36
P21333	Cluster of Filamin-A	FLNA	281 kDa	1.20	0.18	0.30	0.00	4.00	0.00
Q9Y4B6	Protein VPRBP	VPRBP	169 kDa	1.08	0.52	0.65	0.09	1.65	0.05
Q92900	Regulator of nonsense transcripts 1	UPF1	124 kDa	1.08	0.52	0.60	0.00	1.79	0.02
P33316	Cluster of Isoform 2 of Deoxyuridine 5'-triphosphate nucleot	DUT	18 kDa	1.08	0.52	0.90	0.00	1.19	0.19
H0Y5J4	Cluster of Drebrin-like protein (Fragment)	DBNL	40 kDa	1.08	0.52	1.25	0.13	0.86	0.33
Q86SR1	Cluster of Isoform 2 of Polypeptide N-acetylgalactosaminylt	GALNT10	62 kDa	1.08	0.52	0.95	0.80	1.13	0.53
G3XAH6	Cluster of Poly(A) polymerase alpha	PAPOLA	81 kDa	1.73	0.02	0.70	0.00	2.46	0.01
E7EMI0	Cluster of Argininosuccinate lyase	ASL	50 kDa	1.25	0.13	0.80	0.30	1.56	0.08
Q9NW82	WD repeat-containing protein 70	WDR70	73 kDa	1.95	0.01	1.15	0.20	1.70	0.05
Q9UK45	U6 snRNA-associated Sm-like protein LSm7	LSM7	12 kDa	1.30	0.09	1.15	0.20	1.13	0.47
Q12907	Cluster of Vesicular integral-membrane protein VIP36	LMAN2	40 kDa	0.68	0.01	0.75	0.13	0.90	0.49
Q9H173	Nucleotide exchange factor SIL1	SIL1	52 kDa	1.03	0.81	0.75	0.13	1.37	0.13
Q2KHT3	Isoform 2 of Protein CLEC16A	CLEC16A	104 kDa	1.03	0.81	1.05	0.50	0.98	0.87
O75312	Cluster of Zinc finger protein ZPR1	ZNF259	51 kDa	1.03	0.81	1.00	1.00	1.03	0.88
P05387	Cluster of 60S acidic ribosomal protein P2	RPLP2	12 kDa	0.93	0.44	0.65	0.09	1.42	0.11
Q4G176	Cluster of Acyl-CoA synthetase family member 3, mitochon	ACSF3	64 kDa	0.93	0.44	1.55	0.27	0.60	0.04
O95273	Cluster of Cyclin-D1-binding protein 1	CCNDBP1	40 kDa	0.93	0.44	1.20	0.30	0.77	0.12
Q9NZR2	Low-density lipoprotein receptor-related protein 1B	LRP1B	515 kDa	0.93	0.44	1.10	0.70	0.84	0.38
Q969P0	Immunoglobulin superfamily member 8	IGSF8	65 kDa	0.93	0.44	0.90	0.50	1.03	0.87
Q96PD2	Cluster of Discoidin, CUB and LCCL domain-containing pro	DCBLD2	85 kDa	0.93	0.44	1.15	0.20	0.80	0.16
Q9H3P7	Golgi resident protein GCP60	ACBD3	61 kDa	0.93	0.44	0.70	0.20	1.32	0.19
Q8IYB1	Protein MB21D2	MB21D2	56 kDa	0.93	0.44	1.20	0.00	0.77	0.05
P62993	Cluster of Growth factor receptor-bound protein 2	GRB2	25 kDa	1.43	0.05	0.95	0.80	1.50	0.09
Q01658	Protein Dr1	DR1	19 kDa	1.13	0.31	1.00	1.00	1.13	0.31
H3BQQ4	FAD-linked sulfhydryl oxidase ALR	GFER	15 kDa	1.13	0.31	1.70	0.00	0.66	0.01
O95831	Apoptosis-inducing factor 1, mitochondrial	AIFM1	67 kDa	1.13	0.31	0.85	0.20	1.32	0.16
P31946	Cluster of 14-3-3 protein beta/alpha	YWHAB	28 kDa	1.00	1.00	0.65	0.09	1.54	0.07
Q9NY27	Serine/threonine-protein phosphatase 4 regulatory subunit 2	PPP4R2	47 kDa	1.00	1.00	1.05	0.50	0.95	0.74
O94903	Cluster of Proline synthase co-transcribed bacterial homolog	PROSC	30 kDa	1.00	1.00	0.80	0.30	1.25	0.25
Q9Y6R7	IgGFc-binding protein	FCGBP	572 kDa	1.00	1.00	1.65	0.14	0.61	0.02
Q9H5V8	CUB domain-containing protein 1	CDCP1	93 kDa	1.00	1.00	0.85	0.20	1.18	0.35
P49247	Ribose-5-phosphate isomerase	RPIA	33 kDa	1.00	1.00	1.25	0.13	0.80	0.15
J3QQZ1	Sodium channel protein type 4 subunit alpha	SCN4A	208 kDa	1.00	1.00	4.05	0.03	0.25	0.00
Q13451	Cluster of Peptidyl-prolyl cis-trans isomerase FKBP5	FKBP5	51 kDa	1.00	1.00	0.70	0.00	1.43	0.05
P46777	60S ribosomal protein L5	RPL5	34 kDa	1.00	1.00	0.50	0.00	2.00	0.01
Q13509	Cluster of Tubulin beta-3 chain	TUBB3	50 kDa	1.00	1.00	0.35	0.05	2.86	0.01

Q9BR76	Cluster of Coronin-1B	CORO1B	54 kDa	1.45	0.04	0.95	0.50	1.53	0.07
Q9BQT9	Calsyntenin-3	CLSTN3	106 kDa	1.05	0.64	0.75	0.34	1.40	0.15
Q99879	Cluster of Histone H2B type 1-M	HIST1H2BM	14 kDa	1.05	0.64	0.25	0.04	4.20	0.01
Q9BZQ8	Protein Niban	FAM129A	103 kDa	1.05	0.64	0.95	0.50	1.11	0.53
P51532	Cluster of Transcription activator BRG1	SMARCA4	185 kDa	1.05	0.64	0.95	0.50	1.11	0.53
P13667	Cluster of Protein disulfide-isomerase A4	PDIA4	73 kDa	0.53	0.00	0.25	0.04	2.10	0.02
Q16531	Cluster of DNA damage-binding protein 1	DDB1	127 kDa	0.53	0.00	0.35	0.05	1.50	0.09
B4E1K7	Cluster of Stomatin-like protein 2	STOML2	33 kDa	0.53	0.00	1.10	0.50	0.48	0.00
P19404	NADH dehydrogenase [ubiquinone] flavoprotein 2, mitocho	NDUFV2	27 kDa	0.53	0.00	1.55	0.06	0.34	0.00
Q7L2H7	Eukaryotic translation initiation factor 3 subunit M	EIF3M	43 kDa	0.53	0.00	0.10	0.00	5.25	0.00
P41091	Eukaryotic translation initiation factor 2 subunit 3	EIF2S3	51 kDa	0.95	0.60	0.45	0.06	2.11	0.02
O95865	Cluster of N(G),N(G)-dimethylarginine dimethylaminohydro	DDAH2	30 kDa	0.95	0.60	1.00	1.00	0.95	0.75
Q15102	Platelet-activating factor acetylhydrolase IB subunit gamma	PAFAH1B3	26 kDa	0.95	0.60	0.85	0.20	1.12	0.50
Q13263	Transcription intermediary factor 1-beta	TRIM28	89 kDa	0.95	0.60	0.60	0.00	1.58	0.03
O95202	LETM1 and EF-hand domain-containing protein 1, mitochor	LETM1	83 kDa	0.95	0.60	0.90	0.50	1.06	0.75
Q9NUQ9	Cluster of Protein FAM49B	FAM49B	37 kDa	0.95	0.60	0.90	0.00	1.06	0.60
A2ACR1	Proteasome subunit beta type	PSMB9	21 kDa	0.95	0.60	1.20	0.00	0.79	0.06
Q9NX62	Inositol monophosphatase 3	IMPAD1	39 kDa	0.95	0.60	1.10	0.70	0.86	0.45
P25445	Tumor necrosis factor receptor superfamily member 6	FAS	38 kDa	0.95	0.60	0.80	0.30	1.19	0.36
P61086	Cluster of Ubiquitin-conjugating enzyme E2 K	UBE2K	22 kDa	0.95	0.60	0.95	0.50	1.00	1.00
Q9BSJ8	Cluster of Extended synaptotagmin-1	ESYT1	123 kDa	0.95	0.60	1.15	0.20	0.83	0.21
Q96G25	Mediator of RNA polymerase II transcription subunit 8	MED8	29 kDa	0.95	0.60	1.65	0.05	0.58	0.01
Q92876	Kallikrein-6	KLK6	27 kDa	0.95	0.60	0.70	0.00	1.36	0.06
Q9BTE6	Cluster of Isoform 3 of Alanyl-tRNA editing protein Aarsd1	AARSD1	66 kDa	0.83	0.10	0.75	0.34	1.10	0.63
P49903	Selenide, water dikinase 1	SEPHS1	43 kDa	0.83	0.10	0.60	0.16	1.38	0.15
Q06830	Peroxiredoxin-1	PRDX1	22 kDa	0.83	0.10	0.35	0.05	2.36	0.02
J3QRV5	Lethal(2) giant larvae protein homolog 2	LLGL2	113 kDa	0.83	0.10	1.75	0.13	0.47	0.00
Q13596	Sorting nexin-1	SNX1	59 kDa	0.83	0.10	0.80	0.00	1.03	0.76
I3L3I4	Cluster of Actin, cytoplasmic 2	ACTG1	42 kDa	0.83	0.10	0.20	0.00	4.13	0.00
P40818	Cluster of Ubiquitin carboxyl-terminal hydrolase 8	USP8	128 kDa	0.83	0.10	1.00	1.00	0.83	0.10
P63220	40S ribosomal protein S21	RPS21	9 kDa	0.83	0.10	0.75	0.13	1.10	0.56
P67870	Casein kinase II subunit beta	CSNK2B	25 kDa	0.83	0.10	0.85	0.20	0.97	0.84
B5ME91	Programmed cell death protein 4	PDCD4	51 kDa	0.83	0.10	0.85	0.20	0.97	0.84
P23368	Cluster of NAD-dependent malic enzyme, mitochondrial	ME2	65 kDa	0.83	0.10	0.80	0.00	1.03	0.76
H3BRY3	Coronin	CORO1A	43 kDa	0.83	0.10	1.70	0.00	0.49	0.00
Q9Y315	Putative deoxyribose-phosphate aldolase	DERA	35 kDa	1.10	0.39	1.70	0.18	0.65	0.04
Q92598	Cluster of Heat shock protein 105 kDa	HSPH1	97 kDa	0.90	0.31	0.30	0.00	3.00	0.01

Q92820	Gamma-glutamyl hydrolase	GGH	36 kDa	0.90	0.31	0.60	0.00	1.50	0.03
Q05519	Cluster of Isoform 2 of Serine/arginine-rich splicing factor 1	SRSF11	53 kDa	1.15	0.25	1.35	0.09	0.85	0.28
H0YKF0	Cluster of Electron transfer flavoprotein subunit alpha, mitoc	ETFA	30 kDa	1.23	0.14	1.15	0.50	1.07	0.71
J3QT38	Cluster of Exocyst complex component 6B	EXOC6B	79 kDa	1.23	0.14	2.15	0.14	0.57	0.01
Q8TCA0	Cluster of Leucine-rich repeat-containing protein 20	LRRC20	21 kDa	1.23	0.14	1.10	0.50	1.11	0.52
E9PC74	Translation initiation factor eIF-2B subunit epsilon	EIF2B5	78 kDa	1.23	0.14	1.35	0.09	0.91	0.50
P62854	Cluster of 40S ribosomal protein S26	RPS26	13 kDa	1.20	0.16	1.15	0.20	1.04	0.78
Q99941	Cyclic AMP-dependent transcription factor ATF-6 beta	ATF6B	77 kDa	1.30	0.08	1.25	0.13	1.04	0.79
F5H012	E3 ubiquitin-protein ligase TRIM21	TRIM21	54 kDa	1.30	0.08	1.00	1.00	1.30	0.08
J3KR97	Cluster of Tubulin-specific chaperone D	TBCD	137 kDa	1.30	0.08	0.65	0.09	2.00	0.02
H0YFI1	Ragulator complex protein LAMTOR1 (Fragment)	LAMTOR1	9 kDa	1.30	0.08	2.20	0.00	0.59	0.00
E9PIL6	Cluster of Lysine-specific demethylase 2A	KDM2A	82 kDa	0.80	0.07	2.05	0.03	0.39	0.00
Q9Y3U8	Cluster of 60S ribosomal protein L36	RPL36	12 kDa	0.80	0.07	0.90	0.00	0.89	0.25
B4DPY8	Cluster of TOX high mobility group box family member 4	TOX4	64 kDa	0.80	0.07	1.05	0.80	0.76	0.15
Q99996	Cluster of Isoform 6 of A-kinase anchor protein 9	AKAP9	455 kDa	0.80	0.07	0.95	0.50	0.84	0.25
P33240	Cluster of Cleavage stimulation factor subunit 2	CSTF2	61 kDa	0.80	0.07	0.70	0.20	1.14	0.46
P49419	Cluster of Alpha-aminoadipic semialdehyde dehydrogenase	ALDH7A1	58 kDa	0.80	0.07	0.90	0.50	0.89	0.46
Q8TAT6	Cluster of Nuclear protein localization protein 4 homolog	NPLOC4	68 kDa	0.80	0.07	0.55	0.07	1.45	0.09
B4DRF9	Cyclic AMP-dependent transcription factor ATF-1	ATF1	15 kDa	1.35	0.06	2.05	0.03	0.66	0.02
Q06203	Amidophosphoribosyltransferase	PPAT	57 kDa	1.18	0.19	0.60	0.00	1.96	0.01
O43414	ERI1 exoribonuclease 3	ERI3	37 kDa	1.18	0.19	1.20	0.00	0.98	0.82
J3KTL2	Cluster of Serine/arginine-rich-splicing factor 1	SRSF1	28 kDa	1.18	0.19	0.55	0.07	2.14	0.02
Q9Y2X3	Nucleolar protein 58	NOP58	60 kDa	1.18	0.19	0.80	0.00	1.47	0.04
P02749	Beta-2-glycoprotein 1	APOH	38 kDa	1.18	0.19	0.80	0.00	1.47	0.04
Q96HC4	Cluster of PDZ and LIM domain protein 5	PDLIM5	64 kDa	0.98	0.79	0.80	0.00	1.22	0.13
F5GZ27	Cluster of Lon protease homolog	LONP1	86 kDa	0.98	0.79	1.25	0.34	0.78	0.16
P09972	Cluster of Fructose-bisphosphate aldolase C	ALDOC	39 kDa	0.98	0.79	0.45	0.06	2.17	0.02
O15144	Cluster of Actin-related protein 2/3 complex subunit 2	ARPC2	34 kDa	0.98	0.79	0.55	0.07	1.77	0.03
A6NMA8	Cluster of Quinone oxidoreductase-like protein 1	CRYZL1	37 kDa	0.98	0.79	0.90	0.00	1.08	0.44
P29144	Cluster of Tripeptidyl-peptidase 2	TPP2	138 kDa	0.98	0.79	0.60	0.00	1.63	0.02
Q9H3S7	Tyrosine-protein phosphatase non-receptor type 23	PTPN23	179 kDa	0.98	0.79	0.80	0.30	1.22	0.29
Q86UP2	Cluster of Kinectin	KTN1	156 kDa	0.98	0.79	0.55	0.07	1.77	0.03
Q09328	Alpha-1,6-mannosylglycoprotein 6-beta-N-acetylglucosamin	MGAT5	85 kDa	0.98	0.79	0.85	0.20	1.15	0.40
P55268	Laminin subunit beta-2	LAMB2	196 kDa	0.98	0.79	0.85	0.20	1.15	0.40
H0YE76	Activating signal cointegrator 1 complex subunit 1 (Fragmer	ASCC1	21 kDa	0.98	0.79	1.30	0.00	0.75	0.03
B1AMW1	CD58 antigen, (Lymphocyte function-associated antigen 3),	CD58	27 kDa	0.98	0.79	2.20	0.00	0.44	0.00
Q9Y285	Phenylalanine--tRNA ligase alpha subunit	FARSA	58 kDa	0.98	0.79	0.80	0.00	1.22	0.13

Q8WX92	Negative elongation factor B	NELFB	66 kDa	1.40	0.05	0.90	0.00	1.56	0.03
Q8N1G2	Cap-specific mRNA (nucleoside-2'-O-)-methyltransferase 1	CMTR1	95 kDa	3.75	0.00	1.25	0.13	3.00	0.01
Q13155	Aminoacyl tRNA synthase complex-interacting multifunctio	AIMP2	35 kDa	1.28	0.09	0.90	0.00	1.42	0.04
Q9NX02	Cluster of NACHT, LRR and PYD domains-containing prote	NLRP2	121 kDa	1.28	0.09	1.70	0.18	0.75	0.11
Q53GQ0	Estradiol 17-beta-dehydrogenase 12	HSD17B12	34 kDa	0.73	0.02	1.65	0.14	0.44	0.00
P46108	Cluster of Adapter molecule crk	CRK	34 kDa	0.73	0.02	0.70	0.20	1.04	0.84
Q92636	Protein FAN	NSMAF	104 kDa	1.70	0.02	2.40	0.05	0.71	0.04
Q7L576	Cluster of Cytoplasmic FMR1-interacting protein 1	CYFIP1	145 kDa	1.53	0.03	0.60	0.00	2.54	0.01
B4DK25	Cluster of Arginyl-tRNA--protein transferase 1	ATE1	46 kDa	1.53	0.03	1.20	0.00	1.27	0.09
Q9BXJ9	Cluster of N-alpha-acetyltransferase 15, NatA auxiliary subu	NAA15	101 kDa	0.75	0.03	0.35	0.05	2.14	0.02
O00422	Cluster of Histone deacetylase complex subunit SAP18	SAP18	18 kDa	0.75	0.03	1.15	0.50	0.65	0.04
P30153	Cluster of Serine/threonine-protein phosphatase 2A 65 kDa r	PPP2R1A	65 kDa	0.75	0.03	0.40	0.00	1.88	0.01
Q9GZS1	Isoform 2 of DNA-directed RNA polymerase I subunit RPA4	POLR1E	47 kDa	0.75	0.03	1.00	1.00	0.75	0.03
P46109	Crk-like protein	CRKL	34 kDa	1.50	0.03	1.10	0.00	1.36	0.05
Q9UL15	BAG family molecular chaperone regulator 5	BAG5	51 kDa	0.75	0.03	1.20	0.00	0.63	0.01
O60487	Myelin protein zero-like protein 2	MPZL2	24 kDa	0.75	0.03	1.80	0.00	0.42	0.00
Q92925	Cluster of Isoform 3 of SWI/SNF-related matrix-associated a	SMARCD2	55 kDa	0.75	0.03	0.80	0.00	0.94	0.50
F5H3A1	Cluster of Sodium/potassium-transporting ATPase subunit al	ATP1A1	113 kDa	0.75	0.03	0.45	0.06	1.67	0.04
P49862	Kallikrein-7	KLK7	28 kDa	0.75	0.03	0.75	0.13	1.00	1.00
Q9Y2X7	Cluster of ARF GTPase-activating protein GIT1	GIT1	84 kDa	0.75	0.03	0.85	0.20	0.88	0.38
P53680	AP-2 complex subunit sigma	AP2S1	17 kDa	0.75	0.03	1.25	0.13	0.60	0.01
Q86WR0	Coiled-coil domain-containing protein 25	CCDC25	24 kDa	0.75	0.03	1.25	0.13	0.60	0.01
Q8IY81	pre-rRNA processing protein FTSJ3	FTSJ3	97 kDa	0.75	0.03	1.25	0.13	0.60	0.01
Q9Y303	Isoform 3 of Putative N-acetylglucosamine-6-phosphate deac	AMDHD2	64 kDa	0.75	0.03	1.75	0.04	0.43	0.00
Q9NXF7	DDB1- and CUL4-associated factor 16	DCAF16	24 kDa	0.75	0.03	1.30	0.00	0.58	0.00
G3V117	Enhancer of yellow 2 homolog (Drosophila), isoform CRA_c	ENY2	11 kDa	0.75	0.03	1.00	1.00	0.75	0.03
Q14517	Cluster of Protocadherin Fat 1	FAT1	506 kDa	0.75	0.03	0.30	0.00	2.50	0.01
P28066	Cluster of Proteasome subunit alpha type-5	PSMA5	26 kDa	0.75	0.03	0.40	0.00	1.88	0.01
P49902	Cluster of Cytosolic purine 5'-nucleotidase	NT5C2	65 kDa	0.75	0.03	0.80	0.00	0.94	0.50
P11908	Cluster of Isoform 2 of Ribose-phosphate pyrophosphokinase	PRPS2	35 kDa	0.75	0.03	0.90	0.00	0.83	0.10
P62937	Cluster of Peptidyl-prolyl cis-trans isomerase A	PPIA	18 kDa	0.75	0.03	0.65	0.09	1.15	0.38
Q9Y4K0	Cluster of Lysyl oxidase homolog 2	LOXL2	87 kDa	0.75	0.03	0.75	0.13	1.00	1.00
E7ETA6	Pericentriolar material 1 protein	PCM1	228 kDa	2.35	0.01	1.65	0.05	1.42	0.08
Q96PZ0	Cluster of Pseudouridylate synthase 7 homolog	PUS7	75 kDa	0.88	0.19	0.90	0.50	0.97	0.85
P02795	Cluster of Metallothionein-2	MT2A	6 kDa	0.88	0.19	1.40	0.16	0.63	0.01
Q8NBF6	Cluster of Late secretory pathway protein AVL9 homolog	AVL9	72 kDa	0.88	0.19	1.25	0.13	0.70	0.03
Q9UBV2	Protein sel-1 homolog 1	SEL1L	89 kDa	0.88	0.19	1.45	0.07	0.60	0.01

Q9UK41	Vacuolar protein sorting-associated protein 28 homolog	VPS28	25 kDa	0.88	0.19	0.80	0.00	1.09	0.39
Q13042	Cell division cycle protein 16 homolog	CDC16	72 kDa	0.88	0.19	1.00	1.00	0.88	0.19
Q9NSD9	Phenylalanine--tRNA ligase beta subunit	FARSB	66 kDa	0.88	0.19	0.50	0.00	1.75	0.02
P35237	Cluster of Serpin B6	SERPINB6	43 kDa	0.88	0.19	0.80	0.00	1.09	0.39
P78347	Cluster of General transcription factor II-I	GTF2I	112 kDa	0.88	0.19	0.80	0.00	1.09	0.39
Q9P2M7	Cluster of Cingulin	CGN	136 kDa	0.88	0.19	1.10	0.00	0.80	0.06
Q15047	Histone-lysine N-methyltransferase SETDB1	SETDB1	143 kDa	0.88	0.19	1.40	0.00	0.63	0.01
G3V0F8	Cluster of Protein tyrosine phosphatase, non-receptor type 6,	PTPN6	70 kDa	1.83	0.01	0.95	0.50	1.92	0.02
O43242	Cluster of 26S proteasome non-ATPase regulatory subunit 3	PSMD3	61 kDa	1.48	0.03	0.60	0.00	2.46	0.01
P61586	Cluster of Transforming protein RhoA	RHOA	22 kDa	1.60	0.02	0.75	0.34	2.13	0.02
Q68CQ4	Digestive organ expansion factor homolog	DIEXF	87 kDa	1.23	0.12	0.85	0.20	1.44	0.08
J3KNL6	Cluster of Protein transport protein Sec16A	SEC16A	252 kDa	1.23	0.12	1.20	0.00	1.02	0.82
C1IDX9	ATG12 autophagy related 12 homolog (S. cerevisiae), isofor	ATG12	21 kDa	1.23	0.12	1.20	0.00	1.02	0.82
P30048	Thioredoxin-dependent peroxide reductase, mitochondrial	PRDX3	28 kDa	1.13	0.28	0.75	0.34	1.50	0.09
P55010	Cluster of Eukaryotic translation initiation factor 5	EIF5	49 kDa	1.13	0.28	0.85	0.50	1.32	0.18
F5H1E6	DNA-directed RNA polymerase	POLR3B	121 kDa	1.33	0.06	1.40	0.30	0.95	0.73
Q8N8N7	Prostaglandin reductase 2	PTGR2	38 kDa	1.33	0.06	2.05	0.09	0.65	0.02
Q9UIQ6	Leucyl-cystinyl aminopeptidase	LNPEP	117 kDa	1.58	0.02	1.15	0.20	1.37	0.10
P09668	Pro-cathepsin H	CTSH	37 kDa	1.20	0.14	1.25	0.13	0.96	0.76
Q5ZPR3	Cluster of CD276 antigen	CD276	57 kDa	1.03	0.79	1.50	0.24	0.68	0.06
Q6IC75	Cluster of ADP-ribosylation factor-binding protein GGA1	GGA1	72 kDa	1.03	0.79	1.05	0.50	0.98	0.86
O43809	Cleavage and polyadenylation specificity factor subunit 5	NUDT21	26 kDa	1.03	0.79	1.00	1.00	1.03	0.89
E7EQ61	Ubiquitin-like modifier-activating enzyme 5	UBA5	42 kDa	1.03	0.79	1.80	0.16	0.57	0.01
P37108	Cluster of Signal recognition particle 14 kDa protein	SRP14	15 kDa	1.03	0.79	0.85	0.20	1.21	0.25
O95861	Cluster of Isoform 2 of 3'(2'),5'-bisphosphate nucleotidase 1	BPNT1	36 kDa	1.03	0.79	0.90	0.00	1.14	0.24
Q9UHP3	Isoform USP25m of Ubiquitin carboxyl-terminal hydrolase 2	USP25	130 kDa	1.03	0.79	1.50	0.00	0.68	0.01
Q10570	Cleavage and polyadenylation specificity factor subunit 1	CPSF1	161 kDa	1.03	0.79	0.60	0.00	1.71	0.02
O60701	UDP-glucose 6-dehydrogenase	UGDH	55 kDa	1.03	0.79	0.55	0.07	1.86	0.02
Q96J01	THO complex subunit 3	THOC3	39 kDa	1.03	0.79	0.95	0.80	1.08	0.66
P26640	Cluster of Valine--tRNA ligase	VARS	140 kDa	1.25	0.10	0.55	0.07	2.27	0.01
O94973	Cluster of AP-2 complex subunit alpha-2	AP2A2	104 kDa	1.25	0.10	0.95	0.50	1.32	0.13
Q9Y3D6	Mitochondrial fission 1 protein	FIS1	17 kDa	0.58	0.00	1.30	0.37	0.44	0.01
Q5VTL8	Pre-mRNA-splicing factor 38B	PRPF38B	64 kDa	0.58	0.00	0.90	0.50	0.64	0.03
O00629	Cluster of Importin subunit alpha-4	KPNA4	58 kDa	1.30	0.07	0.70	0.00	1.86	0.01
H3BP20	Cluster of Beta-hexosaminidase subunit alpha	HEXA	62 kDa	1.10	0.35	0.70	0.00	1.57	0.02
E9PE68	Oxysterol-binding protein	OSBPL8	97 kDa	1.10	0.35	1.25	0.34	0.88	0.41
O75935	Dynactin subunit 3	DCTN3	21 kDa	1.10	0.35	0.95	0.50	1.16	0.35

Q9NRV9	Cluster of Heme-binding protein 1	HEBP1	21 kDa	1.10	0.35	1.45	0.07	0.76	0.07
Q14126	Desmoglein-2	DSG2	122 kDa	0.70	0.01	0.75	0.13	0.93	0.62
O75822	Cluster of Eukaryotic translation initiation factor 3 subunit J	EIF3J	29 kDa	0.35	0.00	0.70	0.00	0.50	0.00
P27797	Calreticulin	CALR	48 kDa	1.05	0.60	0.90	0.70	1.17	0.45
Q15198	Platelet-derived growth factor receptor-like protein	PDGFRL	42 kDa	1.05	0.60	1.35	0.39	0.78	0.21
Q9UHL4	Dipeptidyl peptidase 2	DPP7	54 kDa	1.05	0.60	0.75	0.13	1.40	0.09
Q8IW45	Cluster of Isoform 2 of ATP-dependent (S)-NAD(P)H-hydra	CARKD	41 kDa	1.05	0.60	0.75	0.13	1.40	0.09
P41208	Cluster of Centrin-2	CETN2	20 kDa	1.05	0.60	1.15	0.20	0.91	0.50
Q9ULR0	Cluster of Pre-mRNA-splicing factor ISY1 homolog	ISY1	33 kDa	1.05	0.60	1.35	0.09	0.78	0.09
Q9UI10	Translation initiation factor eIF-2B subunit delta	EIF2B4	58 kDa	1.05	0.60	1.35	0.09	0.78	0.09
B7ZB78	Ras-related protein Rap-1b	RAP1B	14 kDa	1.05	0.60	1.50	0.00	0.70	0.01
O60493	Sorting nexin-3	SNX3	19 kDa	1.05	0.60	2.20	0.00	0.48	0.00
Q9UQB8	Cluster of Brain-specific angiogenesis inhibitor 1-associated	BAIAP2	61 kDa	0.70	0.01	0.95	0.80	0.74	0.12
Q6P1K2	Isoform 4 of Polyamine-modulated factor 1	PMF1	19 kDa	1.40	0.04	2.55	0.06	0.55	0.00
D6RH31	Nephronectin (Fragment)	NPNT	67 kDa	0.70	0.01	1.30	0.00	0.54	0.00
O00170	AH receptor-interacting protein	AIP	38 kDa	1.45	0.03	0.90	0.00	1.61	0.02
Q8TD19	Serine/threonine-protein kinase Nek9	NEK9	107 kDa	1.45	0.03	0.75	0.13	1.93	0.02
O60506	Cluster of Isoform 3 of Heterogeneous nuclear ribonucleopro	SYNCRIP	63 kDa	1.45	0.03	0.25	0.04	5.80	0.00
P25789	Cluster of Proteasome subunit alpha type-4	PSMA4	29 kDa	0.50	0.00	0.60	0.16	0.83	0.31
Q15063	Periostin	POSTN	93 kDa	1.00	1.00	2.05	0.15	0.49	0.01
P43304	Glycerol-3-phosphate dehydrogenase, mitochondrial	GPD2	81 kDa	1.00	1.00	0.90	0.50	1.11	0.51
P20618	Proteasome subunit beta type-1	PSMB1	26 kDa	0.50	0.00	0.55	0.07	0.91	0.51
Q8NCH0	Carbohydrate sulfotransferase 14	CHST14	43 kDa	1.00	1.00	1.20	0.30	0.83	0.22
O43464	Cluster of Serine protease HTRA2, mitochondrial	HTRA2	49 kDa	1.00	1.00	1.40	0.16	0.71	0.04
Q8TEA8	D-tyrosyl-tRNA(Tyr) deacylase 1	DTD1	23 kDa	1.00	1.00	0.90	0.00	1.11	0.31
P49366	Deoxyhypusine synthase	DHPS	41 kDa	0.50	0.00	0.80	0.00	0.63	0.01
G3V1L6	Chromosome 20 open reading frame 42, isoform CRA_c	FERMT1	48 kDa	1.50	0.03	1.05	0.87	1.43	0.13
D6RDX8	Tetraspanin-17	TSPAN17	34 kDa	0.78	0.04	1.65	0.05	0.47	0.00
J3KPU0	[Pyruvate dehydrogenase [acetyl-transferring]]-phosphatase	PDP1	64 kDa	0.78	0.04	1.70	0.26	0.46	0.01
Q13610	Periodic tryptophan protein 1 homolog	PWP1	56 kDa	0.78	0.04	0.90	0.50	0.86	0.33
Q99536	Synaptic vesicle membrane protein VAT-1 homolog	VAT1	42 kDa	1.55	0.02	0.80	0.00	1.94	0.01
P49770	Translation initiation factor eIF-2B subunit beta	EIF2B2	39 kDa	0.78	0.04	1.10	0.00	0.70	0.01
P06213	Insulin receptor	INSR	156 kDa	0.93	0.39	2.05	0.03	0.45	0.00
Q15542	Transcription initiation factor TFIID subunit 5	TAF5	87 kDa	0.93	0.39	1.80	0.00	0.51	0.00
P15559	NAD(P)H dehydrogenase [quinone] 1	NQO1	31 kDa	0.93	0.39	0.80	0.30	1.16	0.38
P60981	Destrin	DSTN	19 kDa	0.93	0.39	0.55	0.07	1.68	0.03
B4DMU0	Cluster of Pyrroline-5-carboxylate reductase	PYCR1	36 kDa	0.93	0.39	0.75	0.13	1.23	0.21

Q9BTC0	Cluster of Death-inducer obliterator 1	DIDO1	244 kDa	0.93	0.39	1.15	0.20	0.80	0.13
H7C5I8	Cluster of Down syndrome critical region protein 3 (Fragme	DSCR3	9 kDa	0.93	0.39	3.85	0.03	0.24	0.00
Q8IYI6	Exocyst complex component 8	EXOC8	82 kDa	0.93	0.39	1.55	0.06	0.60	0.01
Q9BXS4	Cluster of Transmembrane protein 59	TMEM59	36 kDa	0.93	0.39	1.65	0.05	0.56	0.00
P57735	Ras-related protein Rab-25	RAB25	23 kDa	0.93	0.39	2.15	0.03	0.43	0.00
P78539	Sushi repeat-containing protein SRPX	SRPX	52 kDa	0.93	0.39	0.70	0.00	1.32	0.06
B4DVB8	Cluster of ELAV-like protein 1	ELAVL1	39 kDa	0.93	0.39	0.70	0.00	1.32	0.06
E7EUU1	Cluster of Latent-transforming growth factor beta-binding pr	LTBP4	166 kDa	0.93	0.39	0.70	0.00	1.32	0.06
P61254	Cluster of 60S ribosomal protein L26	RPL26	17 kDa	0.93	0.39	0.90	0.00	1.03	0.76
Q14966	Isoform 3 of Zinc finger protein 638	ZNF638	218 kDa	0.93	0.39	1.00	1.00	0.93	0.39
Q6PCE3	Glucose 1,6-bisphosphate synthase	PGM2L1	70 kDa	0.93	0.39	1.10	0.00	0.84	0.10
H0Y9X1	Translation machinery-associated protein 16 (Fragment)	TMA16	28 kDa	0.93	0.39	1.00	1.00	0.93	0.59
Q9NXH9	tRNA (guanine(26)-N(2))-dimethyltransferase	TRMT1	72 kDa	1.28	0.08	1.05	0.87	1.21	0.36
Q08AM6	Cluster of Protein VAC14 homolog	VAC14	88 kDa	1.28	0.08	0.65	0.26	1.96	0.03
Q8IXH7	Negative elongation factor C/D	NELFCD	66 kDa	1.28	0.08	0.85	0.50	1.50	0.08
Q5T8P6	Cluster of RNA-binding protein 26	RBM26	114 kDa	1.28	0.08	1.10	0.50	1.16	0.35
H7C3P7	Ras-related protein Ral-A (Fragment)	RALA	18 kDa	1.28	0.08	1.05	0.50	1.21	0.23
F5GWK3	Pre-mRNA-splicing factor RBM22	RBM22	41 kDa	1.28	0.08	1.15	0.20	1.11	0.47
Q9H1D9	DNA-directed RNA polymerase III subunit RPC6	POLR3F	36 kDa	1.28	0.08	1.75	0.04	0.73	0.04
F8WCA0	Vesicle-associated membrane protein 2	VAMP2	13 kDa	1.28	0.08	1.30	0.00	0.98	0.82
Q8WXD5	Gem-associated protein 6	GEMIN6	19 kDa	1.18	0.16	1.20	0.50	0.98	0.90
Q7Z4H3	HD domain-containing protein 2	HDDC2	23 kDa	1.73	0.01	2.15	0.08	0.80	0.13
Q7L7X3	Cluster of Serine/threonine-protein kinase TAO1	TAOK1	116 kDa	1.08	0.44	2.20	0.11	0.49	0.00
O95407	Tumor necrosis factor receptor superfamily member 6B	TNFRSF6B	33 kDa	1.08	0.44	0.70	0.00	1.54	0.02
O43592	Exportin-T	XPOT	110 kDa	1.08	0.44	0.90	0.00	1.19	0.13
Q96L92	Sorting nexin-27	SNX27	61 kDa	1.08	0.44	1.10	0.00	0.98	0.79
B4DZM8	26S proteasome non-ATPase regulatory subunit 5	PSMD5	51 kDa	1.08	0.44	0.70	0.20	1.54	0.06
Q02750	Dual specificity mitogen-activated protein kinase kinase 1	MAP2K1	43 kDa	1.08	0.44	1.10	0.50	0.98	0.87
A6NCK0	Cluster of NEDD8-activating enzyme E1 regulatory subunit	NAE1	61 kDa	1.58	0.02	0.80	0.30	1.97	0.02
Q96EP5	DAZ-associated protein 1	DAZAP1	43 kDa	0.90	0.25	1.00	1.00	0.90	0.46
Q9Y5S9	Cluster of RNA-binding protein 8A	RBM8A	20 kDa	0.90	0.25	0.85	0.20	1.06	0.67
Q9H7D7	WD repeat-containing protein 26	WDR26	72 kDa	0.90	0.25	1.35	0.09	0.67	0.02
Q9Y2A7	Nck-associated protein 1	NCKAP1	129 kDa	0.90	0.25	0.70	0.00	1.29	0.07
P10599	Thioredoxin	TXN	12 kDa	0.90	0.25	0.85	0.50	1.06	0.74
Q9NRX5	Serine incorporator 1	SERINC1	50 kDa	0.90	0.25	1.65	0.23	0.55	0.02
E9PSI1	Cluster of Transmembrane 9 superfamily member 1	TM9SF1	92 kDa	0.90	0.25	1.60	0.20	0.56	0.01
Q14315	Filamin-C	FLNC	291 kDa	0.90	0.25	0.45	0.06	2.00	0.02

I3L0H8	Cluster of ATP-dependent RNA helicase DDX19A	DDX19A	51 kDa	0.90	0.25	0.65	0.09	1.38	0.09
Q9NR12	PDZ and LIM domain protein 7	PDLIM7	50 kDa	0.90	0.25	0.95	0.50	0.95	0.67
E9PF19	Transducin beta-like protein 2	TBL2	46 kDa	0.90	0.25	1.30	0.00	0.69	0.01
Q14566	DNA replication licensing factor MCM6	MCM6	93 kDa	0.90	0.25	0.90	0.70	1.00	1.00
Q15554	Telomeric repeat-binding factor 2	TERF2	56 kDa	0.90	0.25	1.75	0.13	0.51	0.00
Q86U86	Protein polybromo-1	PBRM1	193 kDa	0.90	0.25	1.70	0.09	0.53	0.00
Q9Y314	Nitric oxide synthase-interacting protein	NOSIP	33 kDa	1.43	0.03	1.25	0.34	1.14	0.41
Q9UHW5	Isoform 2 of GPN-loop GTPase 3	GPN3	34 kDa	1.33	0.05	1.70	0.00	0.78	0.04
P28340	DNA polymerase delta catalytic subunit	POLD1	124 kDa	1.53	0.02	1.60	0.11	0.95	0.71
P82932	28S ribosomal protein S6, mitochondrial	MRPS6	14 kDa	2.30	0.01	2.45	0.02	0.94	0.61
H0Y368	Cluster of Dolichol-phosphate mannosyltransferase (Fragme	DPM1	33 kDa	1.23	0.10	2.90	0.22	0.42	0.25
O75396	Vesicle-trafficking protein SEC22b	SEC22B	25 kDa	1.23	0.10	1.20	0.30	1.02	0.88
P10253	Lysosomal alpha-glucosidase	GAA	105 kDa	1.23	0.10	0.65	0.09	1.88	0.02
H3BN51	Dynactin subunit 5	DCTN5	17 kDa	1.40	0.03	1.30	0.00	1.08	0.42
P51659	Peroxisomal multifunctional enzyme type 2	HSD17B4	80 kDa	1.50	0.02	1.10	0.70	1.36	0.13
B4DDV1	Interferon-induced protein with tetratricopeptide repeats 5	IFIT5	51 kDa	1.50	0.02	2.15	0.08	0.70	0.03
P33527	Multidrug resistance-associated protein 1	ABCC1	172 kDa	0.98	0.76	1.25	0.50	0.78	0.22
Q9Y281	Cofilin-2	CFL2	19 kDa	0.98	0.76	0.70	0.20	1.39	0.10
O00217	NADH dehydrogenase [ubiquinone] iron-sulfur protein 8, mi	NDUFS8	24 kDa	0.98	0.76	1.75	0.20	0.56	0.02
Q7Z5K2	Cluster of Wings apart-like protein homolog	WAPAL	133 kDa	0.98	0.76	1.45	0.20	0.67	0.03
O00391	Cluster of Sulfhydryl oxidase 1	QSOX1	83 kDa	0.98	0.76	0.50	0.13	1.95	0.02
G3V3G9	Cluster of DDB1- and CUL4-associated factor 8	DCAF8	85 kDa	0.98	0.76	1.20	0.50	0.81	0.25
B4DNJ6	Serine-threonine kinase receptor-associated protein	STRAP	40 kDa	0.98	0.76	0.70	0.20	1.39	0.10
P35998	Cluster of 26S protease regulatory subunit 7	PSMC2	49 kDa	0.98	0.76	0.80	0.50	1.22	0.35
Q8TAF3	Cluster of WD repeat-containing protein 48	WDR48	76 kDa	0.98	0.76	0.75	0.34	1.30	0.20
B4E241	Serine/arginine-rich-splicing factor 3	SRSF3	14 kDa	0.98	0.76	1.05	0.50	0.93	0.56
Q99729	Isoform 3 of Heterogeneous nuclear ribonucleoprotein A/B	HNRNPAB	31 kDa	0.98	0.76	0.70	0.00	1.39	0.04
H0YGR4	Oligoribonuclease, mitochondrial (Fragment)	REXO2	22 kDa	1.63	0.02	0.80	0.30	2.03	0.01
P51665	Cluster of 26S proteasome non-ATPase regulatory subunit 7	PSMD7	37 kDa	1.63	0.02	0.90	0.00	1.81	0.01
P57081	Cluster of tRNA (guanine-N(7)-)-methyltransferase subunit \	WDR4	45 kDa	0.98	0.76	1.15	0.20	0.85	0.21
H7BYM6	Cluster of High mobility group protein HMG-I/HMG-Y	HMGA1	34 kDa	0.98	0.76	0.70	0.20	1.39	0.10
Q9P253	Vacuolar protein sorting-associated protein 18 homolog	VPS18	110 kDa	0.98	0.76	1.30	0.20	0.75	0.06
Q9BQI0	Allograft inflammatory factor 1-like	AIF1L	17 kDa	0.98	0.76	1.85	0.04	0.53	0.00
P35268	60S ribosomal protein L22	RPL22	15 kDa	0.98	0.76	1.00	1.00	0.98	0.76
B1AHL2	Cluster of Fibulin 1	FBLN1	78 kDa	1.55	0.02	0.55	0.07	2.82	0.01
P49321	Cluster of Nuclear autoantigenic sperm protein	NASP	85 kDa	1.55	0.02	0.65	0.09	2.38	0.01
G3V4W4	Glucosamine 6-phosphate N-acetyltransferase (Fragment)	GNPNAT1	18 kDa	1.55	0.02	1.30	0.00	1.19	0.13

E9PH42	Receptor-type tyrosine-protein phosphatase U	PTPRU	162 kDa	0.63	0.00	1.10	0.70	0.57	0.03
P23246	Splicing factor, proline- and glutamine-rich	SFPQ	76 kDa	0.63	0.00	0.55	0.07	1.14	0.39
Q8TF42	Ubiquitin-associated and SH3 domain-containing protein B	UBASH3B	73 kDa	0.63	0.00	1.75	0.13	0.36	0.00
O14964	Hepatocyte growth factor-regulated tyrosine kinase substrate	HGS	86 kDa	0.63	0.00	0.60	0.05	1.05	0.76
P46100	Transcriptional regulator ATRX	ATRX	283 kDa	0.63	0.00	2.55	0.02	0.25	0.00
J3KP07	Cluster of Growth arrest-specific protein 6	GAS6	80 kDa	1.25	0.08	0.60	0.00	2.08	0.01
P52758	Cluster of Ribonuclease UK114	HRSP12	14 kDa	1.25	0.08	1.40	0.00	0.89	0.22
P07900	Cluster of Heat shock protein HSP 90-alpha	HSP90AA1	85 kDa	1.60	0.02	8.25	0.05	0.19	0.00
O14745	Cluster of Na(+)/H(+) exchange regulatory cofactor NHE-RI	SLC9A3R1	39 kDa	0.83	0.07	0.65	0.09	1.27	0.15
E9PCS3	Cluster of 26S proteasome non-ATPase regulatory subunit 2	PSMD2	82 kDa	0.83	0.07	0.65	0.09	1.27	0.15
Q14C86	Cluster of GTPase-activating protein and VPS9 domain-cont	GAPVD1	165 kDa	0.83	0.07	0.65	0.09	1.27	0.15
O75369	Cluster of Isoform 8 of Filamin-B	FLNB	282 kDa	0.83	0.07	0.40	0.11	2.06	0.02
P23434	Glycine cleavage system H protein, mitochondrial	GCSH	19 kDa	0.83	0.07	2.05	0.15	0.40	0.00
Q9H8H0	Cluster of Nucleolar protein 11	NOL11	81 kDa	0.83	0.07	0.85	0.20	0.97	0.81
F5H4Z4	Cluster of F-box/LRR-repeat protein 18	FBXL18	88 kDa	0.83	0.07	0.95	0.50	0.87	0.28
O95479	GDH/6PGL endoplasmic bifunctional protein	H6PD	89 kDa	0.83	0.07	2.00	0.06	0.41	0.00
Q9BRZ2	E3 ubiquitin-protein ligase TRIM56	TRIM56	81 kDa	0.83	0.07	2.00	0.06	0.41	0.00
D6RD47	40S ribosomal protein S23	RPS23	15 kDa	0.83	0.07	1.15	0.20	0.72	0.03
Q01081	Cluster of Splicing factor U2AF 35 kDa subunit	U2AF1	28 kDa	0.83	0.07	1.25	0.13	0.66	0.01
B4DE64	Cluster of Programmed cell death protein 5	PDCD5	15 kDa	0.83	0.07	1.00	1.00	0.83	0.07
B3KXH8	Sorting nexin-9	SNX9	46 kDa	1.20	0.12	0.95	0.50	1.26	0.15
Q9UNX4	WD repeat-containing protein 3	WDR3	106 kDa	1.20	0.12	1.15	0.20	1.04	0.74
O75063	Glycosaminoglycan xylosylkinase	FAM20B	46 kDa	1.20	0.12	1.80	0.00	0.67	0.01
O00291	Cluster of Huntingtin-interacting protein 1	HIP1	116 kDa	1.20	0.12	1.15	0.50	1.04	0.78
B9ZVT1	RNA-binding protein 12B	RBM12B	103 kDa	1.20	0.12	1.95	0.16	0.62	0.02
Q9Y4E6	WD repeat-containing protein 7	WDR7	164 kDa	1.20	0.12	1.30	0.00	0.92	0.35
P49591	Serine--tRNA ligase, cytoplasmic	SARS	59 kDa	1.20	0.12	0.60	0.00	2.00	0.01
O00154	Cluster of Cytosolic acyl coenzyme A thioester hydrolase	ACOT7	42 kDa	0.85	0.10	0.45	0.06	1.89	0.02
P26358	Isoform 2 of DNA (cytosine-5)-methyltransferase 1	DNMT1	185 kDa	0.85	0.10	0.85	0.20	1.00	1.00
P05556	Cluster of Integrin beta-1	ITGB1	88 kDa	0.85	0.10	0.50	0.00	1.70	0.01
O00161	Cluster of Synaptosomal-associated protein 23	SNAP23	23 kDa	0.85	0.10	1.30	0.00	0.65	0.01
Q15019	Cluster of Isoform 2 of Septin-2	SEPT2	45 kDa	0.85	0.10	0.65	0.09	1.31	0.12
C9JIF9	Acylamino-acid-releasing enzyme	APEH	82 kDa	0.85	0.10	0.85	0.20	1.00	1.00
P20645	Cluster of Cation-dependent mannose-6-phosphate receptor	M6PR	31 kDa	0.85	0.10	0.95	0.50	0.89	0.38
Q01082	Cluster of Spectrin beta chain, non-erythrocytic 1	SPTBN1	275 kDa	0.85	0.10	0.30	0.00	2.83	0.00
P62701	Cluster of 40S ribosomal protein S4, X isoform	RPS4X	30 kDa	0.85	0.10	0.60	0.00	1.42	0.03
Q9NQP4	Prefoldin subunit 4	PFDN4	15 kDa	0.85	0.10	0.50	0.13	1.70	0.04

P36959	GMP reductase 1	GMPR	37 kDa	0.85	0.10	0.80	0.30	1.06	0.69
Q9HCE1	Putative helicase MOV-10	MOV10	114 kDa	0.85	0.10	0.55	0.07	1.55	0.04
Q6GMV3	Putative peptidyl-tRNA hydrolase PTRHD1	PTRHD1	16 kDa	0.85	0.10	1.60	0.11	0.53	0.00
Q96F24	Nuclear receptor-binding factor 2	NRBF2	32 kDa	0.85	0.10	1.70	0.09	0.50	0.00
E7EQB2	Lactoferroxin-B (Fragment)	LTF	77 kDa	0.85	0.10	1.05	0.87	0.81	0.33
P26572	Cluster of Alpha-1,3-mannosyl-glycoprotein 2-beta-N-acetyl	MGAT1	51 kDa	0.85	0.10	1.05	0.80	0.81	0.21
P54136	Arginine--tRNA ligase, cytoplasmic	RARS	75 kDa	0.85	0.10	0.45	0.06	1.89	0.02
P01023	Cluster of Alpha-2-macroglobulin	A2M	163 kDa	0.85	0.10	0.35	0.05	2.43	0.01
P41252	Cluster of Isoleucine--tRNA ligase, cytoplasmic	IARS	145 kDa	0.85	0.10	0.55	0.07	1.55	0.04
P45880	Voltage-dependent anion-selective channel protein 2	VDAC2	32 kDa	0.85	0.10	0.75	0.13	1.13	0.38
B3KS98	Cluster of Eukaryotic translation initiation factor 3 subunit H	EIF3H	42 kDa	1.13	0.24	0.35	0.05	3.21	0.00
H0YB56	Cluster of Protein LYRIC (Fragment)	MTDH	25 kDa	1.13	0.24	1.00	1.00	1.13	0.43
F8VRQ1	Cluster of Heterogeneous nuclear ribonucleoprotein A1	HNRNPA1	33 kDa	1.13	0.24	0.65	0.09	1.73	0.02
O75487	Glypican-4	GPC4	62 kDa	1.13	0.24	0.85	0.20	1.32	0.11
Q92922	SWI/SNF complex subunit SMARCC1	SMARCC1	123 kDa	1.13	0.24	0.85	0.20	1.32	0.11
O75323	Cluster of Protein NipSnap homolog 2	GBAS	34 kDa	1.13	0.24	1.50	0.00	0.75	0.02
P08758	Cluster of Annexin A5	ANXA5	36 kDa	1.13	0.24	0.60	0.00	1.88	0.01
A8MZF9	Cluster of Developmentally-regulated GTP-binding protein 2	DRG2	38 kDa	1.83	0.01	1.55	0.06	1.18	0.26
Q04721	Cluster of Neurogenic locus notch homolog protein 2	NOTCH2	265 kDa	1.15	0.18	0.60	0.16	1.92	0.02
P52888	Thimet oligopeptidase	THOP1	79 kDa	1.15	0.18	0.60	0.00	1.92	0.01
Q14563	Semaphorin-3A	SEMA3A	89 kDa	1.15	0.18	0.60	0.00	1.92	0.01
Q92621	Nuclear pore complex protein Nup205	NUP205	228 kDa	1.15	0.18	1.10	0.00	1.05	0.60
B4DHT4	Cluster of U4/U6.U5 tri-snRNP-associated protein 2	USP39	54 kDa	1.15	0.18	0.85	0.20	1.35	0.09
B4DQH9	Ran-binding protein 10	RANBP10	65 kDa	1.58	0.02	2.65	0.02	0.59	0.00
P16949	Cluster of Stathmin	STMN1	17 kDa	1.28	0.06	1.00	1.00	1.28	0.15
Q6RW13	Cluster of Type-1 angiotensin II receptor-associated protein	AGTRAP	17 kDa	1.10	0.31	1.60	0.20	0.69	0.04
Q9H4L7	Cluster of SWI/SNF-related matrix-associated actin-depende	SMARCAD1	117 kDa	3.15	0.00	2.50	0.13	1.26	0.17
Q86SF2	N-acetylgalactosaminyltransferase 7	GALNT7	75 kDa	1.03	0.76	0.85	0.50	1.21	0.29
A6NHG4	D-dopachrome decarboxylase-like protein	DDTL	14 kDa	1.03	0.76	1.75	0.13	0.59	0.01
E9PN76	RING finger protein 214	RNF214	58 kDa	1.03	0.76	3.80	0.07	0.27	0.00
P30520	Adenylosuccinate synthetase isozyme 2	ADSS	50 kDa	1.03	0.76	0.65	0.09	1.58	0.03
Q00534	Cyclin-dependent kinase 6	CDK6	37 kDa	1.03	0.76	1.40	0.16	0.73	0.04
B4DHE8	Cluster of RNA-binding protein Musashi homolog 2	MSI2	35 kDa	1.03	0.76	1.05	0.50	0.98	0.84
H0YN81	Cluster of WD repeat-containing protein 61 (Fragment)	WDR61	32 kDa	1.03	0.76	1.45	0.07	0.71	0.02
P14314	Glucosidase 2 subunit beta	PRKCSH	59 kDa	1.03	0.76	0.50	0.00	2.05	0.01
P06703	Protein S100-A6	S100A6	10 kDa	1.03	0.76	0.70	0.00	1.46	0.02
H0YLI6	Cluster of Isocitrate dehydrogenase [NAD] subunit alpha, mi	IDH3A	15 kDa	1.03	0.76	1.00	1.00	1.03	0.76

Q9NX08	COMM domain-containing protein 8	COMMD8	21 kDa	2.05	0.01	1.40	0.16	1.46	0.05
O75882	Cluster of Isoform 2 of Attractin	ATRN	141 kDa	1.03	0.76	0.55	0.07	1.86	0.02
Q6H3X3	Cluster of Retinoic acid early transcript 1G protein	RAET1G	37 kDa	1.03	0.76	1.20	0.30	0.85	0.24
O95470	Cluster of Sphingosine-1-phosphate lyase 1	SGPL1	64 kDa	1.03	0.76	1.50	0.13	0.68	0.02
Q9BRP4	Cluster of Proteasomal ATPase-associated factor 1	PAAF1	42 kDa	1.03	0.76	1.05	0.50	0.98	0.84
Q9UBF6	RING-box protein 2	RNF7	13 kDa	1.03	0.76	1.20	0.00	0.85	0.10
B1AKJ5	Nardilysin	NRD1	139 kDa	1.53	0.02	0.80	0.30	1.91	0.01
Q6PIJ6	Cluster of Isoform 2 of F-box only protein 38	FBXO38	126 kDa	1.18	0.13	1.45	0.42	0.81	0.33
Q15758	Neutral amino acid transporter B(0)	SLC1A5	57 kDa	1.18	0.13	0.70	0.20	1.68	0.03
O75367	Core histone macro-H2A.1	H2AFY	40 kDa	1.18	0.13	0.70	0.00	1.68	0.01
Q8NFI3	Cluster of Cytosolic endo-beta-N-acetylglucosaminidase	ENGASE	84 kDa	1.18	0.13	1.10	0.50	1.07	0.63
P16870	Cluster of Carboxypeptidase E	CPE	53 kDa	1.43	0.03	0.65	0.09	2.19	0.01
P17980	Cluster of 26S protease regulatory subunit 6A	PSMC3	49 kDa	1.43	0.03	0.70	0.00	2.04	0.01
P35579	Cluster of Myosin-9	MYH9	227 kDa	0.80	0.04	0.25	0.04	3.20	0.00
E9PE77	Cluster of Fibronectin	FN1	256 kDa	0.80	0.04	0.60	0.16	1.33	0.13
J3KQJ1	Cluster of Sulfatase-modifying factor 2	SUMF2	36 kDa	0.80	0.04	2.20	0.16	0.36	0.00
Q9UKI9	Cluster of Isoform 2 of POU domain, class 2, transcription fa	POU2F3	48 kDa	0.80	0.04	1.45	0.20	0.55	0.01
B3KPE7	Bcl-2-like protein 13	BCL2L13	35 kDa	0.80	0.04	1.85	0.04	0.43	0.00
B4DR87	Cluster of Procollagen-lysine,2-oxoglutarate 5-dioxygenase 1	PLOD1	88 kDa	0.80	0.04	0.30	0.00	2.67	0.00
J3QK90	NSFL1 cofactor p47	NSFL1C	41 kDa	0.80	0.04	0.70	0.00	1.14	0.18
Q6NVY1	Cluster of 3-hydroxyisobutyryl-CoA hydrolase, mitochondria	HIBCH	43 kDa	0.80	0.04	1.00	1.00	0.80	0.04
Q9UII2	ATPase inhibitor, mitochondrial	ATPIF1	12 kDa	0.80	0.04	1.30	0.20	0.62	0.01
F8W1L0	Cluster of MHC class I polypeptide-related sequence A	MICA	38 kDa	0.80	0.04	1.05	0.50	0.76	0.05
Q8TCT9	Minor histocompatibility antigen H13	HM13	41 kDa	1.50	0.02	1.25	0.13	1.20	0.20
Q9Y5J7	Mitochondrial import inner membrane translocase subunit T	TIMM9	10 kDa	1.50	0.02	1.10	0.00	1.36	0.03
P62166	Neuronal calcium sensor 1	NCS1	22 kDa	0.88	0.14	0.90	0.50	0.97	0.84
A6NKB8	Cluster of Aminopeptidase B	RNPEP	68 kDa	0.88	0.14	0.75	0.13	1.17	0.28
Q9H1B5	Xylosyltransferase 2	XYLT2	97 kDa	0.88	0.14	1.00	1.00	0.88	0.14
Q92979	Ribosomal RNA small subunit methyltransferase NEP1	EMG1	27 kDa	0.88	0.14	0.95	0.80	0.92	0.60
F5H7J9	Cluster of Low-density lipoprotein receptor-related protein 6	LRP6	175 kDa	0.88	0.14	1.00	1.00	0.88	0.33
B0QY89	Cluster of Eukaryotic translation initiation factor 3 subunit L	EIF3L	71 kDa	0.88	0.14	0.55	0.07	1.59	0.03
P29692	Cluster of Isoform 3 of Elongation factor 1-delta	EEF1D	29 kDa	0.88	0.14	0.65	0.09	1.35	0.09
Q5F1R6	Isoform 2 of DnaJ homolog subfamily C member 21	DNAJC21	67 kDa	0.88	0.14	1.80	0.08	0.49	0.00
P25786	Cluster of Proteasome subunit alpha type-1	PSMA1	30 kDa	0.88	0.14	0.50	0.00	1.75	0.01
P13674	Isoform 2 of Prolyl 4-hydroxylase subunit alpha-1	P4HA1	61 kDa	0.88	0.14	1.15	0.50	0.76	0.11
Q14696	LDLR chaperone MESD	MESDC2	26 kDa	0.88	0.14	1.00	1.00	0.88	0.33
Q99538	Cluster of Legumain	LGMN	49 kDa	0.88	0.14	1.10	0.50	0.80	0.12

Q9Y2L1	Exosome complex exonuclease RRP44	DIS3	109 kDa	0.88	0.14	0.55	0.07	1.59	0.03
Q13619	Cullin-4A	CUL4A	88 kDa	0.88	0.14	0.75	0.13	1.17	0.28
Q8IWE4	DCN1-like protein 3	DCUN1D3	34 kDa	1.85	0.01	2.85	0.05	0.65	0.01
P41273	Tumor necrosis factor ligand superfamily member 9	TNFSF9	27 kDa	1.33	0.04	1.40	0.00	0.95	0.49
B0QYK1	Ewing sarcoma breakpoint region 1	EWSR1	63 kDa	0.68	0.01	0.70	0.00	0.96	0.64
O14744	Protein arginine N-methyltransferase 5	PRMT5	73 kDa	0.68	0.01	0.80	0.30	0.84	0.26
Q5T2E6	UPF0668 protein C10orf76	C10orf76	79 kDa	0.68	0.01	0.90	0.50	0.75	0.08
O43818	U3 small nucleolar RNA-interacting protein 2	RRP9	52 kDa	0.68	0.01	0.75	0.13	0.90	0.39
Q9HB07	Cluster of UPF0160 protein MYG1, mitochondrial	C12orf10	42 kDa	0.68	0.01	1.15	0.20	0.59	0.00
P07339	Cluster of Cathepsin D	CTSD	45 kDa	0.68	0.01	0.10	0.00	6.75	0.00
Q10567	Cluster of Isoform C of AP-1 complex subunit beta-1	AP1B1	104 kDa	0.68	0.01	0.30	0.00	2.25	0.00
Q9Y224	Cluster of UPF0568 protein C14orf166	C14orf166	28 kDa	1.35	0.04	0.60	0.00	2.25	0.00
O95347	Cluster of Structural maintenance of chromosomes protein 2	SMC2	136 kDa	1.35	0.04	0.90	0.00	1.50	0.02
Q9UKK3	Poly [ADP-ribose] polymerase 4	PARP4	193 kDa	1.35	0.04	1.05	0.80	1.29	0.15
O60341	Cluster of Lysine-specific histone demethylase 1A	KDM1A	93 kDa	0.68	0.01	0.65	0.09	1.04	0.76
P37802	Transgelin-2	TAGLN2	22 kDa	1.00	1.00	0.75	0.13	1.33	0.09
P14735	Insulin-degrading enzyme	IDE	118 kDa	1.00	1.00	0.60	0.16	1.67	0.03
P16930	Fumarylacetoacetase	FAH	46 kDa	1.00	1.00	1.85	0.18	0.54	0.01
H0Y5T9	Bromodomain-containing protein 2 (Fragment)	BRD2	88 kDa	1.00	1.00	1.00	1.00	1.00	1.00
P28482	Cluster of Mitogen-activated protein kinase 1	MAPK1	41 kDa	1.00	1.00	0.55	0.07	1.82	0.02
Q9H444	Charged multivesicular body protein 4b	CHMP4B	25 kDa	1.00	1.00	1.10	0.50	0.91	0.46
O95433	Cluster of Activator of 90 kDa heat shock protein ATPase hc	AHSA1	38 kDa	1.00	1.00	0.65	0.09	1.54	0.03
Q9NRD1	F-box only protein 6	FBXO6	34 kDa	1.00	1.00	1.40	0.16	0.71	0.03
Q7Z304	Cluster of MAM domain-containing protein 2	MAMDC2	78 kDa	1.00	1.00	0.75	0.13	1.33	0.09
H3BQZ7	HCG2044799	HNRNPUL2-B	85 kDa	1.00	1.00	0.75	0.13	1.33	0.09
C9JP52	TBC1 domain family member 5	TBC1D5	91 kDa	1.00	1.00	1.50	0.13	0.67	0.02
Q5R372	Rab GTPase-activating protein 1-like	RABGAP1L	93 kDa	1.00	1.00	1.60	0.11	0.63	0.01
P54819	Cluster of Adenylate kinase 2, mitochondrial	AK2	26 kDa	1.00	1.00	0.85	0.20	1.18	0.25
Q9UJH8	Cluster of Meteorin	METRN	31 kDa	1.00	1.00	1.05	0.50	0.95	0.67
B4DT35	Cluster of Nucleoporin p54	NUP54	51 kDa	1.00	1.00	1.05	0.50	0.95	0.67
C9JMP5	Cluster of SOSS complex subunit B1 (Fragment)	NABP2	16 kDa	1.00	1.00	1.55	0.06	0.65	0.01
Q9NR45	Cluster of Sialic acid synthase	NANS	40 kDa	1.00	1.00	0.80	0.00	1.25	0.07
P47895	Cluster of Aldehyde dehydrogenase family 1 member A3	ALDH1A3	56 kDa	1.00	1.00	0.60	0.00	1.67	0.01
Q5T8U3	Cluster of 60S ribosomal protein L7a (Fragment)	RPL7A	22 kDa	1.00	1.00	0.70	0.00	1.43	0.02
Q9Y6G9	Cytoplasmic dynein 1 light intermediate chain 1	DYNC1LI1	57 kDa	1.00	1.00	0.80	0.00	1.25	0.07
Q15061	WD repeat-containing protein 43	WDR43	75 kDa	1.00	1.00	0.80	0.00	1.25	0.07
Q86W42	THO complex subunit 6 homolog	THOC6	38 kDa	1.30	0.05	1.90	0.20	0.68	0.06

Q96RN5	Mediator of RNA polymerase II transcription subunit 15	MED15	87 kDa	1.30	0.05	1.00	1.00	1.30	0.12
P31937	Cluster of 3-hydroxyisobutyrate dehydrogenase, mitochondri	HIBADH	35 kDa	1.30	0.05	1.35	0.09	0.96	0.74
Q9NQX3	Cluster of Gephyrin	GPHN	80 kDa	1.08	0.39	0.60	0.16	1.79	0.02
E7ESY4	Cluster of Metastasis-associated protein MTA1	MTA1	79 kDa	1.08	0.39	0.65	0.09	1.65	0.02
B7Z3Y2	Prenylcysteine oxidase 1	PCYOX1	48 kDa	1.08	0.39	1.70	0.09	0.63	0.01
H0YIM9	Cluster of Protein CHURC1-FNTB (Fragment)	CHURC1-FNT	10 kDa	1.08	0.39	1.60	0.20	0.67	0.03
Q6MZV4	Protein NOXP20	FAM114A1	40 kDa	1.08	0.39	1.55	0.17	0.69	0.03
P49588	Cluster of Alanine--tRNA ligase, cytoplasmic	AARS	107 kDa	1.08	0.39	0.55	0.07	1.95	0.01
F5H4L0	Cluster of Gamma-tubulin complex component 2	TUBGCP2	106 kDa	1.08	0.39	1.20	0.30	0.90	0.38
O14763	Tumor necrosis factor receptor superfamily member 10B	TNFRSF10B	48 kDa	1.08	0.39	1.30	0.20	0.83	0.15
O15126	Secretory carrier-associated membrane protein 1	SCAMP1	38 kDa	1.08	0.39	1.80	0.08	0.60	0.00
Q14669	Isoform 3 of E3 ubiquitin-protein ligase TRIP12	TRIP12	226 kDa	1.08	0.39	1.05	0.50	1.02	0.84
B4DT77	Annexin	ANXA7	38 kDa	1.08	0.39	0.70	0.00	1.54	0.02
E7ES43	Heat shock 70 kDa protein 4L	HSPA4L	98 kDa	1.08	0.39	0.70	0.00	1.54	0.02
G3V180	Cluster of Dipeptidyl peptidase 3	DPP3	84 kDa	1.08	0.39	0.90	0.00	1.19	0.10
P61970	Nuclear transport factor 2	NUTF2	14 kDa	1.23	0.08	1.10	0.00	1.11	0.24
B4DYH8	Cluster of N-acetylglucosamine-6-sulfatase	GNS	60 kDa	1.23	0.08	0.70	0.20	1.75	0.02
A1A4S6	Rho GTPase-activating protein 10	ARHGAP10	89 kDa	1.23	0.08	3.05	0.11	0.40	0.00
Q9UI42	Carboxypeptidase A4	CPA4	47 kDa	1.23	0.08	0.85	0.20	1.44	0.05
P63151	Cluster of Serine/threonine-protein phosphatase 2A 55 kDa r	PPP2R2A	52 kDa	1.23	0.08	0.95	0.50	1.29	0.11
P09543	2',3'-cyclic-nucleotide 3'-phosphodiesterase	CNP	48 kDa	1.23	0.08	1.00	1.00	1.23	0.08
Q9Y6K5	Cluster of 2'-5'-oligoadenylate synthase 3	OAS3	121 kDa	1.23	0.08	0.90	0.00	1.36	0.03
Q9Y5K8	V-type proton ATPase subunit D	ATP6V1D	28 kDa	1.25	0.06	1.00	1.00	1.25	0.16
Q9UN36	Protein NDRG2	NDRG2	41 kDa	1.25	0.06	2.75	0.09	0.45	0.00
Q8WZA9	Immunity-related GTPase family Q protein	IRGQ	63 kDa	1.25	0.06	2.00	0.06	0.63	0.01
P49642	Cluster of DNA primase small subunit	PRIM1	50 kDa	1.25	0.06	1.30	0.00	0.96	0.60
Q96CB8	Cluster of Integrator complex subunit 12	INTS12	49 kDa	1.25	0.06	2.10	0.00	0.60	0.00
Q56VL3	OCIA domain-containing protein 2	OCIAD2	17 kDa	1.25	0.06	2.20	0.00	0.57	0.00
C9JJ19	28S ribosomal protein S34, mitochondrial	MRPS34	26 kDa	1.38	0.03	2.10	0.11	0.65	0.02
C9JQV0	Cluster of Uncharacterized protein C7orf50 (Fragment)	C7orf50	22 kDa	1.38	0.03	1.70	0.00	0.81	0.04
P49959	Cluster of Isoform 2 of Double-strand break repair protein M	MRE11A	78 kDa	1.63	0.01	1.75	0.04	0.93	0.50
Q9UNF0	Protein kinase C and casein kinase substrate in neurons prote	PACSIN2	56 kDa	0.60	0.00	0.85	0.50	0.71	0.09
Q14103	Cluster of Isoform 3 of Heterogeneous nuclear ribonucleopro	HNRNPD	33 kDa	1.20	0.09	0.80	0.30	1.50	0.04
J3KNF8	Cytochrome b5 type B	CYB5B	17 kDa	1.20	0.09	1.35	0.26	0.89	0.38
P17812	CTP synthase 1	CTPS1	67 kDa	1.20	0.09	0.65	0.09	1.85	0.01
C9JJP5	Protein TFG (Fragment)	TFG	20 kDa	0.60	0.00	0.85	0.20	0.71	0.02
Q15853	Upstream stimulatory factor 2	USF2	37 kDa	0.60	0.00	1.80	0.08	0.33	0.00

J3KN32	REST corepressor 1	RCOR1	53 kDa	1.20	0.09	1.05	0.50	1.14	0.30
E9PFN5	Glutathione S-transferase kappa 1	GSTK1	22 kDa	1.20	0.09	1.65	0.05	0.73	0.02
Q5T4S7	Cluster of Isoform 2 of E3 ubiquitin-protein ligase UBR4	UBR4	576 kDa	1.20	0.09	0.50	0.00	2.40	0.00
P45973	Chromobox protein homolog 5	CBX5	22 kDa	0.60	0.00	0.80	0.00	0.75	0.02
Q93052	Lipoma-preferred partner	LPP	66 kDa	0.60	0.00	1.50	0.00	0.40	0.00
O75116	Rho-associated protein kinase 2	ROCK2	161 kDa	0.60	0.00	0.40	0.11	1.50	0.08
P05783	Cluster of Keratin, type I cytoskeletal 18	KRT18	48 kDa	0.60	0.00	0.35	0.05	1.71	0.02
Q9BV38	WD repeat-containing protein 18	WDR18	47 kDa	0.60	0.00	1.10	0.00	0.55	0.00
Q15738	Sterol-4-alpha-carboxylate 3-dehydrogenase, decarboxylatin	NSDHL	42 kDa	0.60	0.00	1.30	0.00	0.46	0.00
A3KFL2	Exosome complex component RRP4	EXOSC2	32 kDa	0.93	0.32	1.15	0.20	0.80	0.09
Q9NVH2	Integrator complex subunit 7	INTS7	107 kDa	0.93	0.32	1.25	0.13	0.74	0.03
J3KQ83	ATP synthase-coupling factor 6, mitochondrial	ATP5J	13 kDa	0.93	0.32	1.55	0.06	0.60	0.00
F5H667	Aspartyl/asparaginyl beta-hydroxylase	ASPH	83 kDa	0.93	0.32	0.70	0.00	1.32	0.04
B4DKD1	Protein tweety homolog 2	TTYH2	56 kDa	0.93	0.32	1.80	0.00	0.51	0.00
Q01105	Cluster of Protein SET	SET	33 kDa	0.93	0.32	0.60	0.16	1.54	0.04
P63244	Cluster of Guanine nucleotide-binding protein subunit beta-2	GNB2L1	35 kDa	0.95	0.50	0.55	0.07	1.73	0.02
P56385	ATP synthase subunit e, mitochondrial	ATP5I	8 kDa	0.95	0.50	1.20	0.30	0.79	0.09
P05026	Sodium/potassium-transporting ATPase subunit beta-1	ATP1B1	35 kDa	0.95	0.50	1.15	0.50	0.83	0.21
Q8NCW5	NAD(P)H-hydrate epimerase	APOA1BP	32 kDa	0.95	0.50	1.00	1.00	0.95	0.69
P52848	Cluster of Bifunctional heparan sulfate N-deacetylase/N-sulf	NDST1	101 kDa	0.95	0.50	1.10	0.00	0.86	0.10
P60866	40S ribosomal protein S20	RPS20	13 kDa	0.95	0.50	1.10	0.00	0.86	0.10
O43633	Charged multivesicular body protein 2a	CHMP2A	25 kDa	0.95	0.50	1.00	1.00	0.95	0.50
Q15828	Cystatin-M	CST6	17 kDa	0.95	0.50	1.30	0.37	0.73	0.09
P00491	Cluster of Purine nucleoside phosphorylase	PNP	32 kDa	0.95	0.50	0.70	0.20	1.36	0.09
E9PCA1	T-complex protein 1 subunit epsilon	CCT5	57 kDa	0.95	0.50	0.45	0.06	2.11	0.01
H0YG22	DNA-binding protein A (Fragment)	YBX3	19 kDa	0.95	0.50	1.55	0.17	0.61	0.01
P62979	Cluster of Ubiquitin-40S ribosomal protein S27a	RPS27A	18 kDa	0.95	0.50	0.55	0.07	1.73	0.02
P50851	Cluster of Lipopolysaccharide-responsive and beige-like anc	LRBA	319 kDa	0.95	0.50	0.55	0.07	1.73	0.02
O75436	Cluster of Vacuolar protein sorting-associated protein 26A	VPS26A	38 kDa	0.95	0.50	0.55	0.07	1.73	0.02
Q9BV19	Uncharacterized protein C1orf50	C1orf50	22 kDa	0.95	0.50	2.20	0.11	0.43	0.00
Q15404	Cluster of Ras suppressor protein 1	RSU1	32 kDa	0.95	0.50	0.65	0.09	1.46	0.04
P42766	60S ribosomal protein L35	RPL35	15 kDa	0.95	0.50	0.85	0.20	1.12	0.38
Q9NQ88	Fructose-2,6-bisphosphatase TIGAR	TIGAR	30 kDa	0.95	0.50	0.95	0.50	1.00	1.00
A6NDG6	Phosphoglycolate phosphatase	PGP	34 kDa	0.95	0.50	1.05	0.50	0.90	0.38
F2Z2Y4	Pyridoxal kinase	PDXK	31 kDa	0.95	0.50	1.15	0.20	0.83	0.12
Q9H446	RWD domain-containing protein 1	RWDD1	28 kDa	0.95	0.50	1.35	0.09	0.70	0.02
P07602	Isoform Sap-mu-9 of Proactivator polypeptide	PSAP	58 kDa	0.95	0.50	0.10	0.00	9.50	0.00

Q9Y4L1	Cluster of Hypoxia up-regulated protein 1	HYOU1	111 kDa	0.95	0.50	0.40	0.00	2.38	0.00
O00468	Cluster of Agrin	AGRN	215 kDa	0.95	0.50	0.40	0.00	2.38	0.00
E7EU23	Cluster of Rab GDP dissociation inhibitor beta	GDI2	51 kDa	0.95	0.50	0.40	0.00	2.38	0.00
Q9P265	Cluster of Disco-interacting protein 2 homolog B	DIP2B	171 kDa	0.95	0.50	0.70	0.00	1.36	0.03
H3BUQ2	Cluster of 2-oxoglutarate and iron-dependent oxygenase dom	OGFOD1	58 kDa	0.95	0.50	0.90	0.00	1.06	0.50
Q8WWI5	Choline transporter-like protein 1	SLC44A1	73 kDa	0.95	0.50	1.10	0.00	0.86	0.10
B4DW92	Nuclear receptor 2C2-associated protein	NR2C2AP	19 kDa	0.95	0.50	2.90	0.00	0.33	0.00
P35250	Cluster of Replication factor C subunit 2	RFC2	39 kDa	1.53	0.01	1.25	0.13	1.22	0.16
J3KPM9	Signal transducer and activator of transcription 1-alpha/beta	STAT1	83 kDa	1.60	0.01	0.90	0.00	1.78	0.01
C9J180	Ubiquitin-conjugating enzyme E2 E2 (Fragment)	UBE2E2	18 kDa	4.53	0.00	3.70	0.05	1.22	0.16
P32320	Cytidine deaminase	CDA	16 kDa	1.28	0.05	1.55	0.17	0.82	0.16
Q15311	RalA-binding protein 1	RALBP1	76 kDa	1.28	0.05	0.95	0.50	1.34	0.07
E7ETZ4	Basic leucine zipper and W2 domain-containing protein 2 (F	BZW2	47 kDa	1.28	0.05	0.55	0.07	2.32	0.01
Q01085	Cluster of Isoform 2 of Nucleolysin TIAR	TIAL1	43 kDa	0.75	0.02	0.60	0.16	1.25	0.20
D6REB5	Alpha-L-iduronidase	IDUA	58 kDa	0.75	0.02	1.20	0.50	0.63	0.04
Q6FI81	Anamorsin	CIAPIN1	34 kDa	0.75	0.02	0.80	0.00	0.94	0.39
Q0PNE2	Elongator complex protein 6	ELP6	30 kDa	1.13	0.19	1.30	0.20	0.87	0.24
O95084	Serine protease 23	PRSS23	43 kDa	1.13	0.19	2.00	0.06	0.56	0.00
Q9BXS5	AP-1 complex subunit mu-1	AP1M1	49 kDa	1.13	0.19	0.70	0.00	1.61	0.01
P00367	Cluster of Glutamate dehydrogenase 1, mitochondrial	GLUD1	61 kDa	1.13	0.19	0.70	0.00	1.61	0.01
P02768	Cluster of Serum albumin	ALB	69 kDa	0.75	0.02	0.45	0.06	1.67	0.02
E9PEI9	Lanosterol synthase	LSS	82 kDa	0.38	0.00	1.10	0.50	0.34	0.00
F5GY80	Complement component C8 beta chain	C8B	60 kDa	1.13	0.19	1.15	0.66	0.98	0.90
Q13232	Nucleoside diphosphate kinase 3	NME3	19 kDa	1.13	0.19	0.75	0.34	1.50	0.06
O43837	Isocitrate dehydrogenase [NAD] subunit beta, mitochondrial	IDH3B	42 kDa	1.13	0.19	3.00	0.13	0.38	0.00
Q14574	Isoform 3B of Desmocollin-3	DSC3	93 kDa	1.13	0.19	1.65	0.14	0.68	0.02
Q9UBC2	Isoform 2 of Epidermal growth factor receptor substrate 15-l	EPS15L1	100 kDa	1.13	0.19	0.75	0.13	1.50	0.03
Q9ULX6	A-kinase anchor protein 8-like	AKAP8L	72 kDa	1.13	0.19	1.05	0.50	1.07	0.56
J3KQV0	Cyclin-dependent kinase inhibitor 1	CDKN1A	22 kDa	1.13	0.19	1.45	0.07	0.78	0.05
G8JLD5	Cluster of Dynamin-1-like protein	DNM1L	80 kDa	1.13	0.19	0.60	0.00	1.88	0.01
O76094	Cluster of Signal recognition particle 72 kDa protein	SRP72	75 kDa	1.13	0.19	0.70	0.00	1.61	0.01
H0YLU8	Cluster of Low-density lipoprotein receptor	LDLR	95 kDa	1.13	0.19	0.80	0.00	1.41	0.02
Q9P258	Protein RCC2	RCC2	56 kDa	0.75	0.02	0.40	0.11	1.88	0.02
O75368	SH3 domain-binding glutamic acid-rich-like protein	SH3BGRL	13 kDa	0.75	0.02	1.35	0.26	0.56	0.01
P46013	Antigen KI-67	MKI67	359 kDa	0.75	0.02	0.75	0.13	1.00	1.00
Q13421	Cluster of Isoform 4 of Mesothelin	MSLN	68 kDa	0.75	0.02	0.85	0.20	0.88	0.28
Q9NZT2	Opioid growth factor receptor	OGFR	73 kDa	0.75	0.02	1.75	0.04	0.43	0.00

J3KN36	Cluster of Nodal modulator 3	NOMO3	139 kDa	0.75	0.02	0.60	0.00	1.25	0.06
Q9NPF2	Carbohydrate sulfotransferase 11	CHST11	42 kDa	0.75	0.02	1.25	0.13	0.60	0.00
Q9UM47	Neurogenic locus notch homolog protein 3	NOTCH3	244 kDa	0.73	0.01	0.85	0.20	0.85	0.18
J3KR54	Isovaleryl Coenzyme A dehydrogenase, isoform CRA_a	IVD	47 kDa	0.73	0.01	1.55	0.06	0.47	0.00
J3KNT0	Cluster of Fascin	FSCN1	52 kDa	0.73	0.01	0.40	0.00	1.81	0.01
P0C0S5	Cluster of Histone H2A.Z	H2AFZ	14 kDa	0.73	0.01	0.40	0.00	1.81	0.01
Q9Y265	Cluster of RuvB-like 1	RUVBL1	50 kDa	0.73	0.01	0.50	0.00	1.45	0.02
Q9HB40	Retinoid-inducible serine carboxypeptidase	SCPEP1	51 kDa	0.73	0.01	0.80	0.00	0.91	0.22
Q9UI26	Importin-11	IPO11	113 kDa	0.73	0.01	0.80	0.00	0.91	0.22
E9PB90	Hexokinase-2	HK2	99 kDa	1.45	0.02	0.95	0.50	1.53	0.03
Q15904	Cluster of V-type proton ATPase subunit S1	ATP6AP1	52 kDa	0.73	0.01	0.90	0.00	0.81	0.04
E9PK91	Bcl-2-associated transcription factor 1	BCLAF1	100 kDa	0.73	0.01	1.00	1.00	0.73	0.01
Q8WUW1	Protein BRICK1	BRK1	9 kDa	0.73	0.01	1.70	0.26	0.43	0.18
Q16204	Coiled-coil domain-containing protein 6	CCDC6	53 kDa	0.73	0.01	0.60	0.16	1.21	0.26
A2A274	Aconitase 2, mitochondrial	ACO2	88 kDa	0.73	0.01	0.95	0.50	0.76	0.04
P07093	Cluster of Isoform 2 of Glia-derived nexin	SERPINE2	44 kDa	0.73	0.01	0.50	0.00	1.45	0.02
J3KPM7	NADH dehydrogenase [ubiquinone] iron-sulfur protein 2, mi	NDUFS2	52 kDa	1.58	0.01	1.70	0.00	0.93	0.31
A6NMQ1	DNA polymerase	POLA1	166 kDa	1.40	0.02	1.15	0.20	1.22	0.15
Q8IWJ2	Cluster of GRIP and coiled-coil domain-containing protein 2	GCC2	196 kDa	1.40	0.02	1.40	0.30	1.00	1.00
P50613	Cluster of Cyclin-dependent kinase 7	CDK7	39 kDa	0.98	0.72	1.10	0.50	0.89	0.33
Q7KZ85	Transcription elongation factor SPT6	SUPT6H	199 kDa	0.98	0.72	0.65	0.09	1.50	0.03
Q15042	Rab3 GTPase-activating protein catalytic subunit	RAB3GAP1	111 kDa	0.98	0.72	1.05	0.50	0.93	0.49
P28070	Proteasome subunit beta type-4	PSMB4	29 kDa	0.98	0.72	0.50	0.13	1.95	0.01
E9PI88	GDP-mannose 4,6 dehydratase	GMDS	39 kDa	0.98	0.72	1.10	0.70	0.89	0.46
F5H6P0	Protein-glutamine gamma-glutamyltransferase 2	TGM2	70 kDa	0.98	0.72	1.25	0.34	0.78	0.11
A6NN98	Myotubularin related protein 2, isoform CRA_a	MTMR2	66 kDa	1.33	0.03	2.40	0.05	0.55	0.00
E7ETH0	Complement factor I light chain	CFI	67 kDa	1.33	0.03	1.00	1.00	1.33	0.03
Q8TD16	Protein bicaudal D homolog 2	BICD2	94 kDa	1.10	0.25	0.95	0.80	1.16	0.35
P11387	Cluster of DNA topoisomerase 1	TOP1	91 kDa	1.10	0.25	0.55	0.07	2.00	0.01
Q7Z4V5	Cluster of Hepatoma-derived growth factor-related protein 2	HDGFRP2	74 kDa	1.10	0.25	0.60	0.16	1.83	0.02
Q9BRG1	Vacuolar protein-sorting-associated protein 25	VPS25	21 kDa	1.10	0.25	0.95	0.80	1.16	0.35
Q14847	LIM and SH3 domain protein 1	LASP1	30 kDa	1.10	0.25	0.70	0.20	1.57	0.03
O75694	Nuclear pore complex protein Nup155	NUP155	155 kDa	1.10	0.25	0.90	0.50	1.22	0.18
Q8TDN6	Ribosome biogenesis protein BRX1 homolog	BRIX1	41 kDa	1.10	0.25	2.35	0.12	0.47	0.00
P25490	Transcriptional repressor protein YY1	YY1	45 kDa	1.10	0.25	1.40	0.16	0.79	0.07
B5MC59	Cluster of Replication protein A 14 kDa subunit	RPA3	9 kDa	1.10	0.25	0.75	0.13	1.47	0.03
F5H7J5	Regulatory-associated protein of mTOR	RPTOR	132 kDa	1.10	0.25	1.50	0.13	0.73	0.03

O43143	Putative pre-mRNA-splicing factor ATP-dependent RNA hel	DHX15	91 kDa	1.10	0.25	0.75	0.13	1.47	0.03
P61020	Cluster of Ras-related protein Rab-5B	RAB5B	24 kDa	1.10	0.25	1.80	0.08	0.61	0.00
Q9HAU5	Regulator of nonsense transcripts 2	UPF2	148 kDa	1.10	0.25	0.95	0.50	1.16	0.25
P40939	Trifunctional enzyme subunit alpha, mitochondrial	HADHA	83 kDa	1.10	0.25	1.05	0.50	1.05	0.67
Q14258	E3 ubiquitin/ISG15 ligase TRIM25	TRIM25	71 kDa	1.10	0.25	0.60	0.00	1.83	0.01
P20020	Cluster of Plasma membrane calcium-transporting ATPase 1	ATP2B1	139 kDa	1.10	0.25	0.90	0.00	1.22	0.07
Q9C0D3	Protein zyg-11 homolog B	ZYG11B	84 kDa	1.10	0.25	2.00	0.00	0.55	0.00
Q8NEZ5	F-box only protein 22	FBXO22	45 kDa	1.10	0.25	0.90	0.00	1.22	0.07
Q7Z3E2	Cluster of Uncharacterized protein C10orf118	CCDC186	104 kDa	1.48	0.02	1.65	0.14	0.89	0.36
Q9BTU6	Cluster of Phosphatidylinositol 4-kinase type 2-alpha	PI4K2A	54 kDa	1.35	0.03	1.40	0.16	0.96	0.75
Q96F86	Cluster of Enhancer of mRNA-decapping protein 3	EDC3	56 kDa	0.90	0.18	1.60	0.20	0.56	0.01
Q15046	Cluster of Lysine--tRNA ligase	KARS	68 kDa	0.90	0.18	0.55	0.07	1.64	0.02
Q9Y376	Calcium-binding protein 39	CAB39	40 kDa	0.90	0.18	0.75	0.13	1.20	0.18
P01266	Thyroglobulin	TG	305 kDa	0.90	0.18	1.25	0.13	0.72	0.02
Q9NPJ6	Mediator of RNA polymerase II transcription subunit 4	MED4	30 kDa	0.90	0.18	1.45	0.07	0.62	0.00
Q13435	Cluster of Splicing factor 3B subunit 2	SF3B2	100 kDa	0.90	0.18	0.50	0.00	1.80	0.01
Q69YN2	CWF19-like protein 1	CWF19L1	61 kDa	0.45	0.00	0.80	0.00	0.56	0.00
Q9UMF0	Intercellular adhesion molecule 5	ICAM5	97 kDa	0.90	0.18	1.00	1.00	0.90	0.18
P30530	Tyrosine-protein kinase receptor UFO	AXL	98 kDa	0.90	0.18	1.20	0.00	0.75	0.01
J3KQT7	Cluster of Inositol monophosphatase 1	IMPA1	37 kDa	0.90	0.18	0.70	0.20	1.29	0.13
P62314	Small nuclear ribonucleoprotein Sm D1	SNRPD1	13 kDa	0.45	0.00	0.70	0.20	0.64	0.03
P62906	60S ribosomal protein L10a	RPL10A	25 kDa	0.90	0.18	0.80	0.30	1.13	0.40
P38606	V-type proton ATPase catalytic subunit A	ATP6V1A	68 kDa	0.90	0.18	0.80	0.30	1.13	0.40
Q14694	Cluster of Isoform 2 of Ubiquitin carboxyl-terminal hydrolas	USP10	93 kDa	0.90	0.18	0.65	0.09	1.38	0.05
Q6P158	Putative ATP-dependent RNA helicase DHX57	DHX57	156 kDa	0.45	0.00	2.70	0.07	0.17	0.05
P05141	Cluster of ADP/ATP translocase 2	SLC25A5	33 kDa	0.90	0.18	0.75	0.13	1.20	0.18
P22694	Cluster of cAMP-dependent protein kinase catalytic subunit	PRKACB	41 kDa	0.90	0.18	0.75	0.13	1.20	0.18
G3V0J0	Cluster of Fragile X mental retardation 1, isoform CRA_e	FMR1	67 kDa	0.90	0.18	0.95	0.50	0.95	0.62
O95400	CD2 antigen cytoplasmic tail-binding protein 2	CD2BP2	38 kDa	0.90	0.18	1.05	0.50	0.86	0.18
Q8NFW8	N-acylneuraminate cytidylyltransferase	CMAS	48 kDa	0.90	0.18	1.00	1.00	0.90	0.18
H0YGL6	Cluster of Ras-related protein Rab-6A (Fragment)	RAB6A	23 kDa	1.35	0.03	0.90	0.00	1.50	0.01
O15294	UDP-N-acetylglucosamine--peptide N-acetylglucosaminyltra	OGT	117 kDa	1.35	0.03	1.20	0.30	1.13	0.36
O75717	WD repeat and HMG-box DNA-binding protein 1	WDHD1	126 kDa	1.35	0.03	0.85	0.20	1.59	0.02
B4DJL6	UPF0464 protein C15orf44	VWA9	61 kDa	1.35	0.03	1.55	0.06	0.87	0.21
Q8WW22	DnaJ homolog subfamily A member 4	DNAJA4	45 kDa	1.18	0.10	1.50	0.34	0.78	0.21
Q08378	Golgin subfamily A member 3	GOLGA3	167 kDa	1.18	0.10	0.75	0.13	1.57	0.02
Q9UKX7	Isoform 2 of Nuclear pore complex protein Nup50	NUP50	47 kDa	1.18	0.10	0.85	0.20	1.38	0.05

O15498	Synaptobrevin homolog YKT6	YKT6	22 kDa	1.18	0.10	1.05	0.50	1.12	0.34
B9EGP5	MON2 protein	MON2	190 kDa	1.18	0.10	1.25	0.13	0.94	0.56
B5MC72	Endoplasmic reticulum lectin 1	ERLEC1	52 kDa	1.18	0.10	1.75	0.04	0.67	0.01
C9JG97	Angio-associated migratory cell protein	AAMP	45 kDa	1.18	0.10	0.90	0.00	1.31	0.04
P78356	Cluster of Phosphatidylinositol 5-phosphate 4-kinase type-2	PIP4K2B	47 kDa	1.18	0.10	1.10	0.00	1.07	0.39
Q9Y3C8	Ubiquitin-fold modifier-conjugating enzyme 1	UFC1	19 kDa	1.65	0.01	1.50	0.13	1.10	0.42
P55884	Eukaryotic translation initiation factor 3 subunit B	EIF3B	92 kDa	1.30	0.03	0.35	0.05	3.71	0.00
P78318	Immunoglobulin-binding protein 1	IGBP1	39 kDa	1.60	0.01	1.20	0.00	1.33	0.03
P62308	Cluster of Small nuclear ribonucleoprotein G	SNRPG	8 kDa	1.38	0.02	0.70	0.00	1.96	0.00
E7EN22	Ubiquitin carboxyl-terminal hydrolase	USP19	151 kDa	0.78	0.02	1.30	0.37	0.60	0.02
P08727	Cluster of Keratin, type I cytoskeletal 19	KRT19	44 kDa	0.78	0.02	0.35	0.05	2.21	0.01
O60512	Beta-1,4-galactosyltransferase 3	B4GALT3	44 kDa	0.78	0.02	1.05	0.80	0.74	0.08
Q9Y5L0	Cluster of Transportin-3	TNPO3	104 kDa	0.78	0.02	0.80	0.30	0.97	0.80
P28072	Proteasome subunit beta type-6	PSMB6	25 kDa	0.78	0.02	0.65	0.09	1.19	0.18
Q9NV70	Isoform 2 of Exocyst complex component 1	EXOC1	100 kDa	0.78	0.02	1.50	0.13	0.52	0.00
P20962	Parathymosin	PTMS	12 kDa	0.78	0.02	1.05	0.50	0.74	0.02
Q15654	Cluster of Thyroid receptor-interacting protein 6	TRIP6	50 kDa	0.78	0.02	1.25	0.13	0.62	0.00
Q01995	Cluster of Transgelin	TAGLN	23 kDa	0.78	0.02	1.45	0.07	0.53	0.00
B4DGU4	Cluster of Catenin beta-1	CTNNB1	85 kDa	0.78	0.02	0.40	0.00	1.94	0.00
P13797	Cluster of Plastin-3	PLS3	71 kDa	0.78	0.02	0.50	0.00	1.55	0.01
P21589	Cluster of 5'-nucleotidase	NT5E	63 kDa	0.78	0.02	0.60	0.00	1.29	0.04
Q96H79	Zinc finger CCCH-type antiviral protein 1-like	ZC3HAV1L	33 kDa	0.78	0.02	1.85	0.25	0.42	0.19
P50454	Cluster of Serpin H1	SERPINH1	46 kDa	0.78	0.02	0.45	0.06	1.72	0.01
B4DRL9	Cluster of Rab proteins geranylgeranyltransferase componen	CHM	57 kDa	0.78	0.02	1.05	0.50	0.74	0.02
Q7Z7M9	Polypeptide N-acetylgalactosaminyltransferase 5	GALNT5	106 kDa	0.78	0.02	1.15	0.20	0.67	0.01
Q99590	Protein SCAF11	SCAF11	165 kDa	0.78	0.02	1.55	0.06	0.50	0.00
Q9P2X0	Dolichol-phosphate mannosyltransferase subunit 3	DPM3	10 kDa	0.78	0.02	1.85	0.04	0.42	0.00
H7C2Q3	26S proteasome non-ATPase regulatory subunit 2 (Fragment	PSMD2	16 kDa	0.78	0.02	0.90	0.00	0.86	0.08
Q16610	Cluster of Extracellular matrix protein 1	ECM1	61 kDa	0.78	0.02	0.80	0.00	0.97	0.64
Q9C0H2	Protein tweety homolog 3	TTYH3	58 kDa	0.78	0.02	1.00	1.00	0.78	0.02
E9PC67	ATP-binding cassette sub-family F member 1	ABCF1	60 kDa	0.78	0.02	1.30	0.00	0.60	0.00
E9PB02	Ribonuclease P protein subunit p30	RPP30	36 kDa	1.55	0.01	1.05	0.50	1.48	0.03
F5H7N9	Cluster of Lactadherin	MFGE8	42 kDa	1.55	0.01	0.85	0.50	1.82	0.01
O14818	Cluster of Proteasome subunit alpha type-7	PSMA7	28 kDa	0.78	0.02	0.60	0.16	1.29	0.14
Q9NVT9	Armadillo repeat-containing protein 1	ARMC1	31 kDa	0.78	0.02	1.15	0.20	0.67	0.01
Q92896	Cluster of Isoform 3 of Golgi apparatus protein 1	GLG1	136 kDa	0.78	0.02	0.30	0.00	2.58	0.00
Q9H9S4	Calcium-binding protein 39-like	CAB39L	39 kDa	1.68	0.01	1.00	1.00	1.68	0.02

F5GZX4	DNA-directed RNA polymerase	POLR1B	132 kDa	1.05	0.50	1.45	0.32	0.72	0.09
O43399	Cluster of Tumor protein D54	TPD52L2	22 kDa	1.05	0.50	0.75	0.13	1.40	0.04
H0YAV1	Ribonucleoside-diphosphate reductase subunit M2 B (Fragm	RRM2B	47 kDa	1.05	0.50	0.95	0.50	1.11	0.38
P13284	Gamma-interferon-inducible lysosomal thiol reductase	IFI30	28 kDa	1.05	0.50	1.05	0.50	1.00	1.00
E7EN89	Cluster of Toll interacting protein, isoform CRA_b	TOLLIP	27 kDa	1.05	0.50	1.15	0.20	0.91	0.38
P49458	Cluster of Signal recognition particle 9 kDa protein	SRP9	10 kDa	1.05	0.50	0.70	0.00	1.50	0.01
Q9UIG0	Tyrosine-protein kinase BAZ1B	BAZ1B	171 kDa	1.05	0.50	1.10	0.00	0.95	0.50
P51149	Cluster of Ras-related protein Rab-7a	RAB7A	23 kDa	1.05	0.50	0.55	0.07	1.91	0.01
B1AK87	Cluster of Capping protein (Actin filament) muscle Z-line, b	CAPZB	29 kDa	1.05	0.50	0.30	0.00	3.50	0.00
O14792	Heparan sulfate glucosamine 3-O-sulfotransferase 1	HS3ST1	36 kDa	1.05	0.50	0.85	0.50	1.24	0.21
Q8TEM1	Nuclear pore membrane glycoprotein 210	NUP210	205 kDa	1.05	0.50	2.05	0.09	0.51	0.00
Q96T76	Cluster of MMS19 nucleotide excision repair protein homol	MMS19	113 kDa	1.05	0.50	0.95	0.50	1.11	0.38
P51884	Lumican	LUM	38 kDa	1.05	0.50	1.05	0.50	1.00	1.00
C9J2Y9	DNA-directed RNA polymerase	POLR2B	133 kDa	1.05	0.50	0.90	0.00	1.17	0.10
P16278	Cluster of Beta-galactosidase	GLB1	76 kDa	1.05	0.50	0.80	0.00	1.31	0.03
Q13557	Cluster of Isoform Delta 12 of Calcium/calmodulin-depende	CAMK2D	54 kDa	1.05	0.50	0.80	0.00	1.31	0.03
H0Y2W2	Cluster of ATPase family AAA domain-containing protein 3	ATAD3A	64 kDa	1.05	0.50	1.20	0.00	0.88	0.10
D6RBN5	OCIA domain-containing protein 1	OCIAD1	24 kDa	1.05	0.50	1.30	0.00	0.81	0.03
P06732	Creatine kinase M-type	CKM	43 kDa	1.05	0.50	1.45	0.20	0.72	0.04
B4DJ85	Glomulin	GLMN	67 kDa	1.05	0.50	1.00	1.00	1.05	0.69
Q9BRP1	Programmed cell death protein 2-like	PDCD2L	39 kDa	1.05	0.50	1.20	0.30	0.88	0.26
Q68EM7	Isoform 5 of Rho GTPase-activating protein 17	ARHGAP17	94 kDa	1.05	0.50	1.20	0.30	0.88	0.26
Q9C0I1	Myotubularin-related protein 12	MTMR12	86 kDa	1.05	0.50	1.30	0.20	0.81	0.09
Q9UHR4	Brain-specific angiogenesis inhibitor 1-associated protein 2-l	BAIAP2L1	57 kDa	1.03	0.72	0.80	0.50	1.28	0.22
P20700	Lamin-B1	LMNB1	66 kDa	1.03	0.72	0.50	0.13	2.05	0.01
F5GZA8	SH3 domain-binding protein 1	SH3BP1	55 kDa	1.03	0.72	1.00	1.00	1.03	0.84
Q00688	Peptidyl-prolyl cis-trans isomerase FKBP3	FKBP3	25 kDa	1.03	0.72	0.65	0.09	1.58	0.02
P84090	Enhancer of rudimentary homolog	ERH	12 kDa	1.03	0.72	0.85	0.20	1.21	0.15
F5GZK1	Cluster of Exostosin-like 2	EXTL2	36 kDa	1.03	0.72	0.85	0.20	1.21	0.15
P61513	Cluster of 60S ribosomal protein L37a	RPL37A	10 kDa	1.03	0.72	1.10	0.00	0.93	0.32
Q8WUM0	Nuclear pore complex protein Nup133	NUP133	129 kDa	1.03	0.72	1.00	1.00	1.03	0.72
P46821	Microtubule-associated protein 1B	MAP1B	271 kDa	1.03	0.72	1.40	0.16	0.73	0.03
Q9BXP5	Serrate RNA effector molecule homolog	SRRT	101 kDa	1.03	0.72	0.60	0.00	1.71	0.01
P19388	DNA-directed RNA polymerases I, II, and III subunit RPAB	POLR2E	25 kDa	1.03	0.72	1.20	0.00	0.85	0.07
H0YE28	Protein C11orf31 (Fragment)	C11orf31	10 kDa	1.23	0.06	1.95	0.03	0.63	0.00
Q96JB5	Isoform 3 of CDK5 regulatory subunit-associated protein 3	CDK5RAP3	31 kDa	1.23	0.06	1.30	0.00	0.94	0.39
Q8N163	Cluster of DBIRD complex subunit KIAA1967	KIAA1967	103 kDa	1.23	0.06	0.60	0.00	2.04	0.00

G3V529	ATP-dependent RNA helicase DDX24	DDX24	91 kDa	1.23	0.06	1.20	0.00	1.02	0.76
P53597	Cluster of Succinyl-CoA ligase [ADP/GDP-forming] subunit	SUCLG1	36 kDa	1.50	0.01	1.15	0.50	1.30	0.10
Q92499	ATP-dependent RNA helicase DDX1	DDX1	82 kDa	1.50	0.01	0.50	0.00	3.00	0.00
Q9H788	Cluster of SH2 domain-containing protein 4A	SH2D4A	53 kDa	1.50	0.01	1.30	0.00	1.15	0.12
Q9NR09	Baculoviral IAP repeat-containing protein 6	BIRC6	530 kDa	1.43	0.02	1.20	0.00	1.19	0.08
Q5XPI4	Cluster of E3 ubiquitin-protein ligase RNF123	RNF123	149 kDa	1.43	0.02	1.15	0.50	1.24	0.16
J3KNX7	NADH dehydrogenase (Ubiquinone) flavoprotein 3, 10kDa,	NDUFV3	51 kDa	1.43	0.02	1.70	0.00	0.84	0.05
Q9Y2S0	DNA-directed RNA polymerases I and III subunit RPAC2	POLR1D	15 kDa	1.73	0.01	1.30	0.20	1.33	0.06
C9JKL2	MLN64 N-terminal domain homolog	STARD3NL	25 kDa	1.45	0.01	3.05	0.02	0.48	0.00
Q13283	Ras GTPase-activating protein-binding protein 1	G3BP1	52 kDa	1.20	0.07	0.95	0.80	1.26	0.15
Q15208	Cluster of Serine/threonine-protein kinase 38	STK38	54 kDa	1.20	0.07	1.75	0.13	0.69	0.02
P30533	Alpha-2-macroglobulin receptor-associated protein	LRPAP1	41 kDa	1.20	0.07	1.05	0.50	1.14	0.25
Q9NWX6	Probable tRNA(His) guanylyltransferase	THG1L	35 kDa	1.20	0.07	1.15	0.20	1.04	0.67
F5GX23	26S proteasome non-ATPase regulatory subunit 9	PSMD9	20 kDa	1.20	0.07	1.20	0.50	1.00	1.00
P48506	Glutamate--cysteine ligase catalytic subunit	GCLC	73 kDa	1.20	0.07	0.95	0.50	1.26	0.09
Q13867	Cluster of Bleomycin hydrolase	BLMH	53 kDa	1.20	0.07	0.60	0.00	2.00	0.00
Q96GQ7	Probable ATP-dependent RNA helicase DDX27	DDX27	90 kDa	1.20	0.07	1.10	0.50	1.09	0.46
P37235	Cluster of Hippocalcin-like protein 1	HPCAL1	22 kDa	1.20	0.07	0.75	0.13	1.60	0.02
Q8NBJ5	Procollagen galactosyltransferase 1	COLGALT1	72 kDa	0.85	0.06	0.65	0.26	1.31	0.17
D6REX5	Selenoprotein P (Fragment)	SEPP1	35 kDa	0.85	0.06	1.40	0.30	0.61	0.02
P57740	Cluster of Nuclear pore complex protein Nup107	NUP107	106 kDa	0.85	0.06	0.90	0.50	0.94	0.63
Q8TEB1	Cluster of DDB1- and CUL4-associated factor 11	DCAF11	62 kDa	0.85	0.06	1.45	0.20	0.59	0.01
Q15388	Mitochondrial import receptor subunit TOM20 homolog	TOMM20	16 kDa	0.85	0.06	1.20	0.30	0.71	0.02
Q15417	Cluster of Calponin-3	CNN3	36 kDa	0.85	0.06	1.15	0.20	0.74	0.02
O15446	Isoform 2 of DNA-directed RNA polymerase I subunit RPA3	CD3EAP	55 kDa	0.85	0.06	1.15	0.20	0.74	0.02
P49411	Cluster of Elongation factor Tu, mitochondrial	TUFM	50 kDa	0.85	0.06	0.80	0.00	1.06	0.39
P05154	Plasma serine protease inhibitor	SERPINA5	46 kDa	0.85	0.06	1.10	0.00	0.77	0.02
O95218	Zinc finger Ran-binding domain-containing protein 2	ZRANB2	37 kDa	1.28	0.04	0.85	0.20	1.50	0.02
Q9BTL3	RNMT-activating mini protein	FAM103A1	14 kDa	1.28	0.04	1.15	0.20	1.11	0.34
Q9BVJ7	Dual specificity protein phosphatase 23	DUSP23	17 kDa	1.28	0.04	1.25	0.13	1.02	0.84
Q9H4G4	Golgi-associated plant pathogenesis-related protein 1	GLIPR2	17 kDa	1.28	0.04	1.35	0.09	0.94	0.56
Q9NUW8	Cluster of Tyrosyl-DNA phosphodiesterase 1	TDP1	68 kDa	1.28	0.04	1.50	0.00	0.85	0.06
F5H3L5	Protocadherin-1	PCDH1	113 kDa	0.85	0.06	1.25	0.34	0.68	0.03
Q09028	Cluster of Histone-binding protein RBBP4	RBBP4	48 kDa	0.85	0.06	0.75	0.13	1.13	0.28
Q08257	Quinone oxidoreductase	CRYZ	35 kDa	0.85	0.06	0.85	0.20	1.00	1.00
P36955	Cluster of Pigment epithelium-derived factor	SERPINF1	46 kDa	1.28	0.04	0.85	0.20	1.50	0.02
Q9BRT6	Protein LLP homolog	LLPH	15 kDa	1.28	0.04	2.95	0.05	0.43	0.00

Q8WVY7	Ubiquitin-like domain-containing CTD phosphatase 1	UBLCP1	37 kDa	0.85	0.06	0.80	0.30	1.06	0.63
P48723	Heat shock 70 kDa protein 13	HSPA13	52 kDa	0.85	0.06	0.90	0.50	0.94	0.63
P52597	Heterogeneous nuclear ribonucleoprotein F	HNRNPF	46 kDa	0.85	0.06	0.65	0.09	1.31	0.07
Q06124	Cluster of Tyrosine-protein phosphatase non-receptor type 1	PTPN11	68 kDa	0.85	0.06	0.65	0.09	1.31	0.07
Q7Z6Z7	Cluster of E3 ubiquitin-protein ligase HUWE1	HUWE1	482 kDa	0.85	0.06	0.65	0.09	1.31	0.07
I3L1I0	Glyoxalase domain-containing protein 4 (Fragment)	GLOD4	13 kDa	0.85	0.06	3.80	0.05	0.22	0.00
Q969T9	Cluster of WW domain-binding protein 2	WBP2	28 kDa	0.85	0.06	1.05	0.50	0.81	0.07
Q6YP21	Kynurenine--oxoglutarate transaminase 3	CCBL2	51 kDa	0.85	0.06	1.15	0.20	0.74	0.02
P20827	Ephrin-A1	EFNA1	24 kDa	0.85	0.06	1.10	0.00	0.77	0.02
P49327	Fatty acid synthase	FASN	273 kDa	0.43	0.00	0.20	0.00	2.13	0.00
P15531	Cluster of Nucleoside diphosphate kinase A	NME1	17 kDa	0.43	0.00	0.30	0.00	1.42	0.02
P11413	Cluster of Isoform 3 of Glucose-6-phosphate 1-dehydrogena	G6PD	62 kDa	1.08	0.32	0.60	0.16	1.79	0.01
Q9UMX5	Neudesin	NENF	19 kDa	1.08	0.32	1.55	0.17	0.69	0.02
P62753	Cluster of 40S ribosomal protein S6	RPS6	29 kDa	1.08	0.32	0.75	0.13	1.43	0.03
Q96AG4	Leucine-rich repeat-containing protein 59	LRRC59	35 kDa	1.08	0.32	0.60	0.16	1.79	0.01
O75663	TIP41-like protein	TIPRL	31 kDa	1.08	0.32	1.00	1.00	1.08	0.54
P29317	Cluster of Ephrin type-A receptor 2	EPHA2	108 kDa	1.08	0.32	0.55	0.07	1.95	0.01
E9PD53	Structural maintenance of chromosomes protein	SMC4	144 kDa	1.08	0.32	1.00	1.00	1.08	0.54
B4DIS3	Dpy-30-like protein, isoform CRA_b	LOC84661	14 kDa	1.08	0.32	1.20	0.30	0.90	0.33
Q13618	Cluster of Cullin-3	CUL3	89 kDa	1.08	0.32	0.65	0.09	1.65	0.01
Q96B45	UPF0693 protein C10orf32	C10orf32	12 kDa	1.08	0.32	1.60	0.00	0.67	0.00
O15431	High affinity copper uptake protein 1	SLC31A1	21 kDa	1.08	0.32	1.70	0.00	0.63	0.00
Q9NPQ8	Cluster of Isoform 2 of Synembryn-A	RIC8A	59 kDa	1.40	0.02	1.55	0.27	0.90	0.49
D6RGG3	Cluster of Collagen alpha-1(XII) chain	COL12A1	333 kDa	0.70	0.01	0.35	0.05	2.00	0.01
P08582	Cluster of Melanotransferrin	MFI2	80 kDa	0.70	0.01	1.05	0.50	0.67	0.01
P02765	Alpha-2-HS-glycoprotein	AHSG	39 kDa	0.70	0.01	0.70	0.00	1.00	1.00
Q9BUI4	DNA-directed RNA polymerase III subunit RPC3	POLR3C	61 kDa	0.70	0.01	1.10	0.00	0.64	0.00
B4DQJ8	6-phosphogluconate dehydrogenase, decarboxylating	PGD	52 kDa	0.70	0.01	0.35	0.05	2.00	0.01
Q00403	Transcription initiation factor IIB	GTF2B	35 kDa	0.70	0.01	1.00	1.00	0.70	0.03
Q96IJ6	Mannose-1-phosphate guanyltransferase alpha	GMPPA	46 kDa	0.70	0.01	1.20	0.30	0.58	0.00
O95619	YEATS domain-containing protein 4	YEATS4	27 kDa	0.70	0.01	1.95	0.10	0.36	0.00
Q07666	Cluster of KH domain-containing, RNA-binding, signal trans	KHDRBS1	48 kDa	0.70	0.01	0.85	0.20	0.82	0.09
A2A2V4	Cluster of Vascular endothelial growth factor	VEGF	19 kDa	0.70	0.01	0.95	0.50	0.74	0.02
F5H4E4	Ketosamine-3-kinase	FN3KRP	29 kDa	0.70	0.01	1.15	0.20	0.61	0.00
O75955	Flotillin-1	FLOT1	47 kDa	0.70	0.01	1.85	0.04	0.38	0.00
Q13043	Cluster of Serine/threonine-protein kinase 4	STK4	56 kDa	0.70	0.01	2.05	0.03	0.34	0.00
O95793	Cluster of Double-stranded RNA-binding protein Staufen ho	STAU1	63 kDa	0.70	0.01	0.80	0.00	0.88	0.09

Q9NVM9	Cluster of Protein asunder homolog	Asun	80 kDa	0.70	0.01	0.90	0.00	0.78	0.02
Q99832	Cluster of T-complex protein 1 subunit eta	CCT7	59 kDa	0.70	0.01	0.30	0.00	2.33	0.00
P36952	Cluster of Serpin B5	SERPINB5	42 kDa	0.70	0.01	0.80	0.00	0.88	0.09
A8MV37	Protein SEC13 homolog	SEC13	34 kDa	0.70	0.01	0.90	0.00	0.78	0.02
H0YCJ8	Blood group Rh(CE) polypeptide (Fragment)	RHCE	8 kDa	1.78	0.00	1.50	0.34	1.18	0.32
B5MCF9	Cluster of Pescadillo homolog	PES1	66 kDa	2.05	0.00	0.85	0.20	2.41	0.00
Q9UL46	Proteasome activator complex subunit 2	PSME2	27 kDa	0.83	0.04	0.70	0.20	1.18	0.26
F5H293	Seizure 6-like protein 2	SEZ6L2	94 kDa	0.83	0.04	1.50	0.13	0.55	0.00
O75150	Cluster of E3 ubiquitin-protein ligase BRE1B	RNF40	114 kDa	0.83	0.04	0.90	0.00	0.92	0.22
Q9NX24	H/ACA ribonucleoprotein complex subunit 2	NHP2	17 kDa	0.83	0.04	1.30	0.00	0.63	0.00
O00231	Cluster of 26S proteasome non-ATPase regulatory subunit 1	PSMD11	47 kDa	0.83	0.04	0.35	0.05	2.36	0.00
P48444	Cluster of Coatomer subunit delta	ARCN1	57 kDa	0.83	0.04	0.35	0.05	2.36	0.00
Q6YHK3	CD109 antigen	CD109	162 kDa	0.83	0.04	0.45	0.06	1.83	0.01
Q9UQ80	Cluster of Proliferation-associated protein 2G4	PA2G4	44 kDa	0.83	0.04	0.45	0.06	1.83	0.01
H3BLZ8	Cluster of Probable ATP-dependent RNA helicase DDX17	DDX17	80 kDa	0.83	0.04	0.65	0.09	1.27	0.09
Q9NTX5	Cluster of Ethylmalonyl-CoA decarboxylase	ECHDC1	34 kDa	0.83	0.04	0.75	0.13	1.10	0.39
P13693	Cluster of Translationally-controlled tumor protein	TPT1	20 kDa	0.83	0.04	0.75	0.13	1.10	0.39
G8JLK7	Nucleolar protein 6	NOL6	122 kDa	0.83	0.04	1.60	0.11	0.52	0.00
Q9NQG5	Regulation of nuclear pre-mRNA domain-containing protein	RPRD1B	37 kDa	0.83	0.04	0.85	0.20	0.97	0.76
Q86UD1	Out at first protein homolog	OAF	31 kDa	0.83	0.04	0.95	0.50	0.87	0.18
P04844	Cluster of Isoform 2 of Dolichyl-diphosphooligosaccharide--	RPN2	68 kDa	0.83	0.04	1.05	0.50	0.79	0.04
Q14562	ATP-dependent RNA helicase DHX8	DHX8	139 kDa	0.83	0.04	1.15	0.20	0.72	0.01
O14787	Transportin-2	TNPO2	101 kDa	0.83	0.04	1.15	0.20	0.72	0.01
O14949	Cytochrome b-c1 complex subunit 8	UQCRQ	10 kDa	0.83	0.04	1.15	0.20	0.72	0.01
A2A2V1	Major prion protein (Fragment)	PRNP	27 kDa	0.83	0.04	1.25	0.13	0.66	0.01
Q8TB72	Cluster of Pumilio homolog 2	PUM2	114 kDa	0.83	0.04	1.25	0.13	0.66	0.01
Q9Y3B2	Exosome complex component CSL4	EXOSC1	21 kDa	0.83	0.04	1.35	0.09	0.61	0.00
C9J2P0	Cluster of Ubiquitin-conjugating enzyme E2 E1 (Fragment)	UBE2E1	16 kDa	0.83	0.04	1.35	0.09	0.61	0.00
Q6VY07	Cluster of Phosphofurin acidic cluster sorting protein 1	PACS1	105 kDa	0.83	0.04	1.45	0.07	0.57	0.00
Q9NXR1	Cluster of Isoform 2 of Nuclear distribution protein nudE ho	NDE1	38 kDa	0.83	0.04	1.55	0.06	0.53	0.00
Q9BWM7	Sideroflexin-3	SFXN3	36 kDa	0.83	0.04	1.75	0.04	0.47	0.00
Q9ULT8	E3 ubiquitin-protein ligase HECTD1	HECTD1	289 kDa	0.83	0.04	0.80	0.00	1.03	0.64
P04083	Annexin A1	ANXA1	39 kDa	0.83	0.04	0.30	0.00	2.75	0.00
Q99988	Growth/differentiation factor 15	GDF15	34 kDa	0.83	0.04	0.30	0.00	2.75	0.00
F5H2S7	Dynactin subunit 2	DCTN2	45 kDa	0.83	0.04	0.60	0.00	1.38	0.02
H0YMB3	Cluster of GMP reductase 2	GMPR2	34 kDa	0.83	0.04	0.70	0.00	1.18	0.08
O94763	Unconventional prefoldin RPB5 interactor 1	URI1	60 kDa	0.83	0.04	1.10	0.00	0.75	0.01

Q06323	Cluster of Proteasome activator complex subunit 1	PSME1	29 kDa	0.83	0.04	0.60	0.16	1.38	0.08
P63279	Cluster of SUMO-conjugating enzyme UBC9	UBE2I	18 kDa	0.83	0.04	0.60	0.16	1.38	0.08
O60884	DnaJ homolog subfamily A member 2	DNAJA2	46 kDa	0.83	0.04	0.70	0.20	1.18	0.26
B4DZH6	Histone deacetylase 6	HDAC6	133 kDa	0.83	0.04	1.45	0.20	0.57	0.01
O43813	Cluster of LanC-like protein 1	LANCL1	45 kDa	0.83	0.04	0.65	0.09	1.27	0.09
Q13485	Mothers against decapentaplegic homolog 4	SMAD4	60 kDa	0.83	0.04	0.95	0.50	0.87	0.18
F8W9X7	Coiled-coil domain-containing protein 93	CCDC93	73 kDa	0.83	0.04	1.05	0.50	0.79	0.04
Q9GZX3	Cluster of Carbohydrate sulfotransferase 6	CHST6	44 kDa	0.83	0.04	1.35	0.09	0.61	0.00
P78406	Cluster of mRNA export factor	RAE1	41 kDa	0.83	0.04	0.70	0.00	1.18	0.08
Q9BQ67	Glutamate-rich WD repeat-containing protein 1	GRWD1	49 kDa	1.00	1.00	1.45	0.32	0.69	0.06
P03950	Angiogenin	ANG	17 kDa	1.00	1.00	1.15	0.50	0.87	0.30
Q9UQ35	Serine/arginine repetitive matrix protein 2	SRRM2	300 kDa	1.00	1.00	1.10	0.50	0.91	0.40
Q8IWV8	Cluster of E3 ubiquitin-protein ligase UBR2	UBR2	201 kDa	1.00	1.00	1.20	0.30	0.83	0.13
Q709C8	Vacuolar protein sorting-associated protein 13C	VPS13C	422 kDa	1.00	1.00	1.05	0.50	0.95	0.62
P15907	Cluster of Beta-galactoside alpha-2,6-sialyltransferase 1	ST6GAL1	47 kDa	1.00	1.00	1.15	0.20	0.87	0.18
P48729	Cluster of Casein kinase I isoform alpha	CSNK1A1	39 kDa	1.00	1.00	1.15	0.20	0.87	0.18
Q9Y217	Myotubularin-related protein 6	MTMR6	72 kDa	1.00	1.00	1.55	0.06	0.65	0.00
G3V5Z7	Cluster of Proteasome subunit alpha type	PSMA6	28 kDa	1.00	1.00	0.50	0.00	2.00	0.00
E7EWS7	Ubiquitin-conjugating enzyme E2 L3	UBE2L3	24 kDa	1.00	1.00	0.70	0.00	1.43	0.01
H3BSW6	Cluster of Cytoplasmic tRNA 2-thiolation protein 2	CTU2	64 kDa	1.00	1.00	1.00	1.00	1.00	1.00
H0Y4Z8	Cluster of Rab-like protein 6 (Fragment)	RABL6	26 kDa	1.00	1.00	1.10	0.00	0.91	0.18
Q75MJ1	ATP-binding cassette sub-family F member 2	ABCF2	72 kDa	1.00	1.00	1.20	0.00	0.83	0.04
Q9Y508	RING finger protein 114	RNF114	26 kDa	1.00	1.00	1.05	0.87	0.95	0.79
Q99436	Proteasome subunit beta type-7	PSMB7	30 kDa	1.00	1.00	0.55	0.07	1.82	0.01
Q8NFZ4	Cluster of Neuroligin-2	NLGN2	91 kDa	1.00	1.00	1.40	0.16	0.71	0.02
P37198	Nuclear pore glycoprotein p62	NUP62	53 kDa	1.00	1.00	1.25	0.13	0.80	0.05
P13726	Tissue factor	F3	33 kDa	1.00	1.00	2.05	0.03	0.49	0.00
P09874	Poly [ADP-ribose] polymerase 1	PARP1	113 kDa	1.00	1.00	0.40	0.00	2.50	0.00
P35270	Sepiapterin reductase	SPR	28 kDa	1.00	1.00	1.10	0.00	0.91	0.18
Q8IWV7	Cluster of E3 ubiquitin-protein ligase UBR1	UBR1	200 kDa	1.60	0.01	1.00	1.00	1.60	0.01
Q9NW13	Cluster of RNA-binding protein 28	RBM28	86 kDa	1.33	0.02	0.80	0.50	1.66	0.03
Q92905	COP9 signalosome complex subunit 5	COPS5	38 kDa	1.33	0.02	0.75	0.13	1.77	0.01
O43240	Kallikrein-10	KLK10	30 kDa	1.33	0.02	0.85	0.20	1.56	0.02
A9UHW6	MIF4G domain-containing protein	MIF4GD	25 kDa	1.33	0.02	2.15	0.03	0.62	0.00
P14866	Heterogeneous nuclear ribonucleoprotein L	HNRNPL	64 kDa	1.33	0.02	0.50	0.00	2.65	0.00
J3KNM0	Cluster of Protein phosphatase 1A	PPM1A	51 kDa	1.15	0.10	1.00	1.00	1.15	0.26
P54725	UV excision repair protein RAD23 homolog A	RAD23A	40 kDa	1.15	0.10	1.25	0.13	0.92	0.38

Q15833	Syntaxin-binding protein 2	STXBP2	66 kDa	1.15	0.10	0.80	0.30	1.44	0.04
E7ESC6	Cluster of Exportin-7	XPO7	124 kDa	1.15	0.10	0.80	0.30	1.44	0.04
Q9H269	Cluster of Vacuolar protein sorting-associated protein 16 hor	VPS16	95 kDa	1.15	0.10	3.25	0.07	0.35	0.00
C9JEJ2	Cluster of Choline-phosphate cytidylyltransferase A	PCYT1A	43 kDa	1.15	0.10	1.15	0.20	1.00	1.00
Q00577	Transcriptional activator protein Pur-alpha	PURA	35 kDa	1.15	0.10	1.15	0.20	1.00	1.00
Q9BY77	Isoform 2 of Polymerase delta-interacting protein 3	POLDIP3	43 kDa	1.15	0.10	1.25	0.13	0.92	0.38
P15151	Poliovirus receptor	PVR	45 kDa	1.15	0.10	1.45	0.07	0.79	0.04
Q5T123	SH3 domain binding glutamic acid-rich protein like 3	SH3BGRL3	9 kDa	1.15	0.10	1.55	0.06	0.74	0.02
H7C5G1	Isoamyl acetate-hydrolyzing esterase 1 homolog (Fragment)	IAH1	25 kDa	1.15	0.10	1.40	0.00	0.82	0.03
P35658	Isoform 5 of Nuclear pore complex protein Nup214	NUP214	215 kDa	1.15	0.10	1.10	0.00	1.05	0.50
Q9UJX6	Isoform 2 of Anaphase-promoting complex subunit 2	ANAPC2	93 kDa	1.15	0.10	1.30	0.00	0.88	0.10
P17706	Tyrosine-protein phosphatase non-receptor type 2	PTPN2	48 kDa	1.15	0.10	1.30	0.00	0.88	0.10
C9JW96	Prohibitin (Fragment)	PHB	27 kDa	1.15	0.10	0.70	0.00	1.64	0.01
Q5T0D2	Cluster of Cytidine monophosphate (UMP-CMP) kinase 1, c	CMPK1	19 kDa	1.15	0.10	1.20	0.00	0.96	0.50
J3QQ67	60S ribosomal protein L18 (Fragment)	RPL18	22 kDa	1.15	0.10	0.70	0.00	1.64	0.01
Q8NEF9	Serum response factor-binding protein 1	SRFBP1	49 kDa	1.15	0.10	1.35	0.26	0.85	0.21
H7C2C7	Cluster of Negative elongation factor A (Fragment)	NELFA	27 kDa	1.15	0.10	3.65	0.01	0.32	0.00
Q5VST3	Cluster of Protein O-linked mannose beta1,2-N-acetylglucos	POMGNT1	85 kDa	1.15	0.10	1.60	0.00	0.72	0.01
Q5TA45	Cluster of Integrator complex subunit 11	CPSF3L	68 kDa	1.53	0.01	1.45	0.07	1.05	0.60
Q9UIC8	Cluster of Leucine carboxyl methyltransferase 1	LCMT1	38 kDa	1.53	0.01	1.05	0.50	1.45	0.02
Q13952	Cluster of Nuclear transcription factor Y subunit gamma	NFYC	50 kDa	1.13	0.14	1.45	0.42	0.78	0.24
O95486	Protein transport protein Sec24A	SEC24A	120 kDa	1.13	0.14	0.80	0.30	1.41	0.04
Q7Z7H5	Isoform 3 of Transmembrane emp24 domain-containing prot	TMED4	21 kDa	1.13	0.14	1.10	0.50	1.02	0.84
P30419	Cluster of Glycylpeptide N-tetradecanoyltransferase 1	NMT1	57 kDa	1.13	0.14	0.75	0.13	1.50	0.02
Q8N441	Fibroblast growth factor receptor-like 1	FGFRL1	55 kDa	1.13	0.14	0.95	0.50	1.18	0.15
Q9UQ53	Cluster of Alpha-1,3-mannosyl-glycoprotein 4-beta-N-acetyl	MGAT4B	63 kDa	1.13	0.14	1.30	0.00	0.87	0.07
B8ZZZ1	AP-1 complex subunit sigma-3	AP1S3	16 kDa	1.55	0.01	1.30	0.00	1.19	0.06
J3KPP7	Beta-arrestin-1	ARRB1	47 kDa	1.88	0.00	0.95	0.50	1.97	0.00
Q5JR95	40S ribosomal protein S8	RPS8	22 kDa	0.88	0.08	0.75	0.34	1.17	0.34
Q96H20	Cluster of Vacuolar-sorting protein SNF8	SNF8	29 kDa	0.88	0.08	0.85	0.50	1.03	0.84
O95336	6-phosphogluconolactonase	PGLS	28 kDa	0.88	0.08	1.25	0.34	0.70	0.03
O00264	Cluster of Membrane-associated progesterone receptor comp	PGRMC1	22 kDa	0.88	0.08	1.25	0.34	0.70	0.03
P46783	Cluster of 40S ribosomal protein S10	RPS10	19 kDa	0.88	0.08	0.90	0.50	0.97	0.80
P62304	Small nuclear ribonucleoprotein E	SNRPE	11 kDa	0.88	0.08	1.45	0.20	0.60	0.01
Q14108	Lysosome membrane protein 2	SCARB2	54 kDa	0.88	0.08	1.65	0.14	0.53	0.00
Q00653	Cluster of Isoform 4 of Nuclear factor NF-kappa-B p100 sub	NFKB2	97 kDa	0.88	0.08	0.55	0.07	1.59	0.01
Q9NP72	Ras-related protein Rab-18	RAB18	23 kDa	0.88	0.08	1.10	0.50	0.80	0.08

P09960	Leukotriene A-4 hydrolase	LTA4H	69 kDa	0.88	0.08	0.55	0.07	1.59	0.01
P19827	Inter-alpha-trypsin inhibitor heavy chain H1	ITIH1	101 kDa	0.88	0.08	1.30	0.20	0.67	0.01
Q96HE7	Cluster of ERO1-like protein alpha	ERO1L	54 kDa	0.88	0.08	0.65	0.09	1.35	0.04
Q9UBU8	Cluster of Mortality factor 4-like protein 1	MORF4L1	41 kDa	0.88	0.08	1.30	0.20	0.67	0.01
F5H6C8	Mitochondrial antiviral-signaling protein	MAVS	40 kDa	0.88	0.08	3.35	0.07	0.26	0.00
B7Z5Z2	Ras-related protein R-Ras2	RRAS2	24 kDa	0.88	0.08	1.40	0.16	0.63	0.01
P51693	Amyloid-like protein 1	APLP1	72 kDa	0.88	0.08	1.40	0.16	0.63	0.01
B8X2Z3	CARMIL2b	RLTPR	148 kDa	0.88	0.08	2.15	0.08	0.41	0.00
E7EWL7	Cluster of Disks large homolog 1	DLG1	88 kDa	0.88	0.08	0.75	0.13	1.17	0.18
J3QT22	Cluster of Protein phosphatase methylesterase 1	PPME1	44 kDa	0.88	0.08	0.75	0.13	1.17	0.18
P53041	Cluster of Serine/threonine-protein phosphatase 5	PPP5C	57 kDa	0.88	0.08	0.75	0.13	1.17	0.18
O75934	Pre-mRNA-splicing factor SPF27	BCAS2	26 kDa	0.88	0.08	0.85	0.20	1.03	0.76
Q9H2M9	Cluster of Rab3 GTPase-activating protein non-catalytic sub	RAB3GAP2	156 kDa	0.88	0.08	0.95	0.50	0.92	0.39
C9JPG0	Cluster of Secernin-1	SCRN1	49 kDa	0.88	0.08	1.05	0.50	0.83	0.09
P47914	60S ribosomal protein L29	RPL29	18 kDa	0.88	0.08	1.05	0.50	0.83	0.09
Q13573	SNW domain-containing protein 1	SNW1	61 kDa	0.88	0.08	1.15	0.20	0.76	0.02
Q6QNY0	Biogenesis of lysosome-related organelles complex 1 subuni	BLOC1S3	21 kDa	0.88	0.08	1.15	0.20	0.76	0.02
Q9UBI6	Guanine nucleotide-binding protein G(I)/G(S)/G(O) subunit	GNG12	8 kDa	0.88	0.08	1.35	0.09	0.65	0.00
Q15043	Zinc transporter ZIP14	SLC39A14	54 kDa	0.88	0.08	1.45	0.07	0.60	0.00
F5GWX5	Cluster of Chromodomain-helicase-DNA-binding protein 4	CHD4	217 kDa	0.88	0.08	0.50	0.00	1.75	0.00
Q02878	60S ribosomal protein L6	RPL6	33 kDa	0.88	0.08	0.60	0.00	1.46	0.01
Q9P016	Thymocyte nuclear protein 1	THYN1	26 kDa	0.88	0.08	1.00	1.00	0.88	0.08
P35659	Cluster of Protein DEK	DEK	43 kDa	0.88	0.08	0.80	0.00	1.09	0.22
E5RJR5	S-phase kinase-associated protein 1	SKP1	19 kDa	0.88	0.08	0.80	0.00	1.09	0.22
H7BY58	Cluster of Protein-L-isoaspartate O-methyltransferase	PCMT1	30 kDa	0.88	0.08	0.80	0.00	1.09	0.22
P61019	Isoform 2 of Ras-related protein Rab-2A	RAB2A	21 kDa	0.88	0.08	1.10	0.00	0.80	0.02
Q96A49	Synapse-associated protein 1	SYAP1	40 kDa	0.88	0.08	1.60	0.00	0.55	0.00
A5YKK6	Cluster of Isoform 4 of CCR4-NOT transcription complex su	CNOT1	174 kDa	0.88	0.08	0.70	0.00	1.25	0.04
P55263	Isoform 3 of Adenosine kinase	ADK	34 kDa	0.88	0.08	0.80	0.00	1.09	0.22
Q13464	Rho-associated protein kinase 1	ROCK1	158 kDa	0.88	0.08	0.80	0.00	1.09	0.22
Q15291	Retinoblastoma-binding protein 5	RBBP5	59 kDa	0.88	0.08	0.80	0.00	1.09	0.22
H7C2W9	60S ribosomal protein L31 (Fragment)	RPL31	13 kDa	0.88	0.08	1.00	1.00	0.88	0.08
Q8NEW0	Zinc transporter 7	SLC30A7	42 kDa	1.38	0.02	2.60	0.12	0.53	0.00
P31930	Cytochrome b-c1 complex subunit 1, mitochondrial	UQCRC1	53 kDa	1.50	0.01	1.10	0.50	1.36	0.04
Q9NWT1	p21-activated protein kinase-interacting protein 1	PAK1IP1	44 kDa	1.30	0.02	0.90	0.00	1.44	0.01
J3QL81	Cluster of Mitochondrial enolase superfamily member 1	ENOSF1	41 kDa	1.30	0.02	1.45	0.20	0.90	0.35
Q9HBU6	Cluster of Ethanolamine kinase 1	ETNK1	51 kDa	1.30	0.02	2.50	0.08	0.52	0.00

O95571	Protein ETHE1, mitochondrial	ETHE1	28 kDa	1.30	0.02	1.10	0.00	1.18	0.07
H3BP13	Trafficking protein particle complex subunit 2-like protein	TRAPPC2L	27 kDa	1.58	0.01	2.30	0.00	0.68	0.00
Q9Y383	Cluster of Putative RNA-binding protein Luc7-like 2	LUC7L2	47 kDa	1.70	0.00	0.55	0.07	3.09	0.00
H0YES5	HBS1-like protein (Fragment)	HBS1L	19 kDa	1.93	0.00	3.75	0.08	0.51	0.00
H3BSA4	Na(+)/H(+) exchange regulatory cofactor NHE-RF2 (Fragme	SLC9A3R2	19 kDa	1.18	0.07	2.10	0.11	0.56	0.00
O43708	Maleylacetoacetate isomerase	GSTZ1	24 kDa	1.18	0.07	1.20	0.30	0.98	0.84
Q9NUM4	Transmembrane protein 106B	TMEM106B	31 kDa	1.18	0.07	1.40	0.16	0.84	0.12
Q9GZN4	Brain-specific serine protease 4	PRSS22	34 kDa	1.18	0.07	1.05	0.50	1.12	0.28
P17301	Cluster of Integrin alpha-2	ITGA2	129 kDa	1.18	0.07	0.70	0.00	1.68	0.00
Q9BS26	Endoplasmic reticulum resident protein 44	ERP44	47 kDa	1.18	0.07	0.70	0.00	1.68	0.00
H7C2W3	Cluster of Protein transport protein Sec31A	SEC31A	131 kDa	0.95	0.39	0.75	0.13	1.27	0.07
P58546	Myotrophin	MTPN	13 kDa	0.95	0.39	0.95	0.50	1.00	1.00
F8VVA7	Cluster of Coatomer subunit zeta-1	COPZ1	22 kDa	0.95	0.39	0.95	0.50	1.00	1.00
Q96SB3	Neurabin-2	PPP1R9B	89 kDa	0.95	0.39	1.65	0.05	0.58	0.00
Q9Y6A4	UPF0468 protein C16orf80	C16orf80	23 kDa	0.95	0.39	1.65	0.05	0.58	0.00
P19823	Inter-alpha-trypsin inhibitor heavy chain H2	ITIH2	106 kDa	0.95	0.39	0.80	0.00	1.19	0.06
P21796	Voltage-dependent anion-selective channel protein 1	VDAC1	31 kDa	0.95	0.39	0.80	0.00	1.19	0.06
P48739	Phosphatidylinositol transfer protein beta isoform	PITPNB	32 kDa	0.95	0.39	0.90	0.00	1.06	0.39
H3BRS6	ADP-dependent glucokinase (Fragment)	ADPGK	22 kDa	1.43	0.01	1.65	0.05	0.86	0.13
Q5JTD0	Tight junction-associated protein 1	TJAP1	62 kDa	1.43	0.01	2.90	0.00	0.49	0.00
F5H2L4	Acyl-coenzyme A thioesterase 13	ACOT13	12 kDa	1.43	0.01	1.20	0.00	1.19	0.06
Q8NFF5	Cluster of Isoform 2 of FAD synthase	FLAD1	54 kDa	0.95	0.39	2.55	0.10	0.37	0.00
P49757	Protein numb homolog	NUMB	71 kDa	0.48	0.00	2.25	0.08	0.21	0.05
E7EWE9	Kinetochore protein Spc24	SPC24	17 kDa	0.95	0.39	1.85	0.04	0.51	0.00
P26599	Cluster of Polypyrimidine tract-binding protein 1	PTBP1	57 kDa	0.95	0.39	0.60	0.00	1.58	0.01
O00487	26S proteasome non-ATPase regulatory subunit 14	PSMD14	35 kDa	0.95	0.39	0.80	0.00	1.19	0.06
Q86VS8	Cluster of Protein Hook homolog 3	HOOK3	83 kDa	0.95	0.39	1.10	0.00	0.86	0.06
Q9H0D6	5'-3' exoribonuclease 2	XRN2	109 kDa	0.95	0.39	0.60	0.00	1.58	0.01
P00750	Cluster of Tissue-type plasminogen activator	PLAT	63 kDa	0.95	0.39	0.70	0.00	1.36	0.02
O75494	Cluster of Serine/arginine-rich splicing factor 10	SRSF10	31 kDa	0.95	0.39	1.15	0.66	0.83	0.31
Q13751	Laminin subunit beta-3	LAMB3	130 kDa	0.95	0.39	0.55	0.07	1.73	0.01
P46063	ATP-dependent DNA helicase Q1	RECQL	73 kDa	0.95	0.39	0.65	0.09	1.46	0.02
P39748	Cluster of Flap endonuclease 1	FEN1	43 kDa	0.95	0.39	0.50	0.13	1.90	0.01
P84022	Cluster of Isoform 2 of Mothers against decapentaplegic hon	SMAD3	43 kDa	0.95	0.39	3.00	0.06	0.32	0.00
Q9Y221	Isoform 2 of 60S ribosome subunit biogenesis protein NIP7 l	NIP7	15 kDa	0.95	0.39	1.10	0.00	0.86	0.06
P06400	Retinoblastoma-associated protein	RB1	106 kDa	1.65	0.00	1.70	0.09	0.97	0.75
G5E9W3	Cleavage and polyadenylation specific factor 3, 73kDa, isofo	CPSF3	73 kDa	1.65	0.00	0.90	0.00	1.83	0.00

P22626	Heterogeneous nuclear ribonucleoproteins A2/B1	HNRNPA2B1	37 kDa	1.10	0.18	0.45	0.06	2.44	0.00
Q658Y4	Cluster of Protein FAM91A1	FAM91A1	94 kDa	1.10	0.18	1.00	1.00	1.10	0.40
Q9Y3B9	RRP15-like protein	RRP15	31 kDa	1.10	0.18	1.20	0.30	0.92	0.40
P53602	Diphosphomevalonate decarboxylase	MVD	43 kDa	1.10	0.18	0.65	0.09	1.69	0.01
Q9BY44	Cluster of Eukaryotic translation initiation factor 2A	EIF2A	65 kDa	1.10	0.18	0.75	0.13	1.47	0.02
P01137	Transforming growth factor beta-1	TGFB1	44 kDa	1.10	0.18	0.85	0.20	1.29	0.05
P19525	Interferon-induced, double-stranded RNA-activated protein k	EIF2AK2	62 kDa	1.10	0.18	0.85	0.20	1.29	0.05
Q8N335	Glycerol-3-phosphate dehydrogenase 1-like protein	GPD1L	38 kDa	1.10	0.18	1.05	0.50	1.05	0.62
B4E040	Ras-related protein Ral-B	RALB	26 kDa	1.10	0.18	1.75	0.04	0.63	0.00
H3BM91	COMM domain-containing protein 4 (Fragment)	COMMD4	23 kDa	1.10	0.18	1.85	0.04	0.59	0.00
P31751	Cluster of RAC-beta serine/threonine-protein kinase	AKT2	56 kDa	1.10	0.18	1.30	0.00	0.85	0.04
O15230	Laminin subunit alpha-5	LAMA5	400 kDa	0.55	0.00	0.30	0.00	1.83	0.00
Q06210	Cluster of Glucosamine--fructose-6-phosphate aminotransfer	GFPT1	79 kDa	1.10	0.18	0.50	0.00	2.20	0.00
P00492	Hypoxanthine-guanine phosphoribosyltransferase	HPRT1	25 kDa	1.10	0.18	0.70	0.00	1.57	0.01
F5H0L8	SEC23-interacting protein	SEC23IP	90 kDa	0.55	0.00	0.70	0.00	0.79	0.01
I3NI00	Cluster of Basigin	BSG	46 kDa	1.10	0.18	0.80	0.00	1.38	0.01
Q13438	Protein OS-9	OS9	76 kDa	1.10	0.18	0.90	0.00	1.22	0.04
P41567	Eukaryotic translation initiation factor 1	EIF1	13 kDa	1.10	0.18	1.00	1.00	1.10	0.18
C9JMV9	Cluster of Protein ABHD14A-ACY1	ABHD14A-AC	56 kDa	1.10	0.18	1.30	0.00	0.85	0.04
P22670	MHC class II regulatory factor RFX1	RFX1	105 kDa	0.93	0.22	1.40	0.30	0.66	0.03
P34896	Cluster of Serine hydroxymethyltransferase, cytosolic	SHMT1	53 kDa	0.93	0.22	0.85	0.20	1.09	0.39
Q8NHP8	Cluster of Putative phospholipase B-like 2	PLBD2	65 kDa	0.93	0.22	0.95	0.50	0.97	0.76
Q86VN1	Vacuolar protein-sorting-associated protein 36	VPS36	44 kDa	0.93	0.22	1.00	1.00	0.93	0.22
Q8N9N7	Leucine-rich repeat-containing protein 57	LRRC57	27 kDa	0.93	0.22	1.00	1.00	0.93	0.22
P07711	Cluster of Cathepsin L1	CTSL1	38 kDa	0.93	0.22	1.10	0.00	0.84	0.04
F6T1Q0	2',5'-phosphodiesterase 12	PDE12	52 kDa	0.93	0.22	1.00	1.00	0.93	0.22
P35527	Keratin, type I cytoskeletal 9	KRT9	62 kDa	0.93	0.22	1.15	0.66	0.80	0.25
P04181	Ornithine aminotransferase, mitochondrial	OAT	49 kDa	0.93	0.22	1.15	0.50	0.80	0.13
P30084	Enoyl-CoA hydratase, mitochondrial	ECHS1	31 kDa	0.93	0.22	0.90	0.50	1.03	0.80
O60313	Dynamin-like 120 kDa protein, mitochondrial	OPA1	112 kDa	0.93	0.22	1.00	1.00	0.93	0.47
P16035	Metalloproteinase inhibitor 2	TIMP2	24 kDa	0.93	0.22	0.55	0.07	1.68	0.01
Q86TI2	Dipeptidyl peptidase 9	DPP9	98 kDa	0.93	0.22	0.65	0.09	1.42	0.02
F5GX77	tRNA methyltransferase 112 homolog	TRMT112	12 kDa	0.93	0.22	1.30	0.20	0.71	0.02
Q96KR1	Cluster of Zinc finger RNA-binding protein	ZFR	117 kDa	0.93	0.22	0.75	0.13	1.23	0.09
Q9Y2T3	Cluster of Guanine deaminase	GDA	51 kDa	0.93	0.22	0.75	0.13	1.23	0.09
P16615	Cluster of Sarcoplasmic/endoplasmic reticulum calcium ATI	ATP2A2	115 kDa	0.93	0.22	0.75	0.13	1.23	0.09
P19623	Spermidine synthase	SRM	34 kDa	0.93	0.22	1.25	0.13	0.74	0.01

Q9NV88	Cluster of Integrator complex subunit 9	INTS9	74 kDa	0.93	0.22	1.25	0.13	0.74	0.01
O95155	Ubiquitin conjugation factor E4 B	UBE4B	146 kDa	0.93	0.22	1.25	0.13	0.74	0.01
H0YBT8	Cluster of Transforming acidic coiled-coil-containing protei	TACC1	51 kDa	0.93	0.22	1.25	0.13	0.74	0.01
H0YBQ0	Thioredoxin reductase 3 (Fragment)	TXNRD3	76 kDa	0.93	0.22	1.85	0.04	0.50	0.00
P49792	Cluster of E3 SUMO-protein ligase RanBP2	RANBP2	358 kDa	0.93	0.22	0.60	0.00	1.54	0.01
Q5VZE5	N-alpha-acetyltransferase 35, NatC auxiliary subunit	NAA35	84 kDa	0.93	0.22	1.80	0.00	0.51	0.00
Q9P0M6	Core histone macro-H2A.2	H2AFY2	40 kDa	0.93	0.22	1.00	1.00	0.93	0.22
Q00765	Receptor expression-enhancing protein 5	REEP5	21 kDa	0.93	0.22	1.10	0.00	0.84	0.04
Q9Y5L4	Mitochondrial import inner membrane translocase subunit T	TIMM13	11 kDa	0.93	0.22	1.20	0.00	0.77	0.01
G5EA58	Cluster of Hydroxymethylbilane synthase, isoform CRA_d	HMBS	35 kDa	0.93	0.22	1.20	0.00	0.77	0.01
G5EA42	Cluster of Tropomodulin 2 (Neuronal), isoform CRA_a	TMOD2	34 kDa	0.93	0.22	4.40	0.00	0.21	0.00
F5H1H6	Cluster of Cytospin-A	SPECC1L	120 kDa	0.93	0.22	1.10	0.00	0.84	0.04
E9PKG1	Cluster of Protein arginine N-methyltransferase 1	PRMT1	38 kDa	0.93	0.22	0.40	0.00	2.31	0.00
Q06481	Cluster of Amyloid-like protein 2	APLP2	87 kDa	0.93	0.22	0.50	0.00	1.85	0.00
P33993	DNA replication licensing factor MCM7	MCM7	81 kDa	0.93	0.22	0.80	0.00	1.16	0.08
E9PL17	Polyribonucleotide 5'-hydroxyl-kinase Clp1	CLP1	49 kDa	0.93	0.22	1.40	0.00	0.66	0.00
Q9H9T3	Elongator complex protein 3	ELP3	62 kDa	0.93	0.22	1.50	0.00	0.62	0.00
Q9Y3C1	Cluster of Nucleolar protein 16	NOP16	21 kDa	0.93	0.22	1.50	0.00	0.62	0.00
E9PDI4	Ladinin-1	LAD1	59 kDa	0.93	0.22	1.70	0.00	0.54	0.00
Q9ULJ6	Cluster of Zinc finger MIZ domain-containing protein 1	ZMIZ1	115 kDa	1.25	0.03	2.25	0.13	0.56	0.01
Q9UN86	Isoform B of Ras GTPase-activating protein-binding protein	G3BP2	51 kDa	1.25	0.03	0.85	0.20	1.47	0.02
Q9Y305	Acyl-coenzyme A thioesterase 9, mitochondrial	ACOT9	50 kDa	1.25	0.03	2.10	0.06	0.60	0.00
P07099	Cluster of Epoxide hydrolase 1	EPHX1	53 kDa	1.25	0.03	1.15	0.20	1.09	0.38
Q9Y244	Proteasome maturation protein	POMP	16 kDa	1.25	0.03	1.15	0.20	1.09	0.38
Q6ZRH9	Uncharacterized protein FLJ46347		54 kDa	1.25	0.03	1.25	0.13	1.00	1.00
P08574	Cytochrome c1, heme protein, mitochondrial	CYC1	35 kDa	1.25	0.03	1.65	0.05	0.76	0.02
Q9UKZ1	UPF0760 protein C2orf29	CNOT11	55 kDa	1.25	0.03	1.85	0.04	0.68	0.00
H3BLY1	Cluster of Contactin-associated protein-like 3B (Fragment)	CNTNAP3B	129 kDa	1.25	0.03	2.05	0.03	0.61	0.00
Q9HD42	Charged multivesicular body protein 1a	CHMP1A	22 kDa	1.25	0.03	1.00	1.00	1.25	0.03
Q8WUF5	RelA-associated inhibitor	PPP1R13L	89 kDa	1.25	0.03	1.50	0.00	0.83	0.03
O75131	Copine-3	CPNE3	60 kDa	1.25	0.03	0.50	0.13	2.50	0.00
Q14739	Lamin-B receptor	LBR	71 kDa	1.25	0.03	1.55	0.27	0.81	0.17
P19876	Cluster of C-X-C motif chemokine 3	CXCL3	11 kDa	1.25	0.03	0.95	0.50	1.32	0.04
O60885	Cluster of Bromodomain-containing protein 4	BRD4	152 kDa	1.25	0.03	1.25	0.13	1.00	1.00
A7MAP0	Cluster of Coronin	CORO1C	54 kDa	1.25	0.03	0.60	0.00	2.08	0.00
H0YED9	Wilms tumor protein (Fragment)	WT1	20 kDa	1.25	0.03	1.00	1.00	1.25	0.03
Q6DKK2	Tetratricopeptide repeat protein 19, mitochondrial	TTC19	42 kDa	1.23	0.04	1.10	0.50	1.11	0.33

O75844	CAAX prenyl protease 1 homolog	ZMPSTE24	55 kDa	1.23	0.04	1.15	0.20	1.07	0.49
J3KNI9	TBC1 domain family member 15	TBC1D15	79 kDa	1.23	0.04	1.35	0.09	0.91	0.28
Q9UN37	Cluster of Vacuolar protein sorting-associated protein 4A	VPS4A	49 kDa	1.23	0.04	1.30	0.00	0.94	0.32
P54577	Tyrosine--tRNA ligase, cytoplasmic	YARS	59 kDa	0.80	0.02	0.60	0.16	1.33	0.08
Q13275	Cluster of Semaphorin-3F	SEMA3F	88 kDa	0.80	0.02	0.90	0.50	0.89	0.31
Q13425	Cluster of Beta-2-syntrophin	SNTB2	58 kDa	0.80	0.02	0.90	0.50	0.89	0.31
B1Q2N1	Cluster of ATP-dependent RNA helicase DDX39A	DDX39	30 kDa	0.80	0.02	0.45	0.06	1.78	0.01
P27816	Cluster of Isoform 6 of Microtubule-associated protein 4	MAP4	120 kDa	0.80	0.02	0.45	0.06	1.78	0.01
A8MUM1	Cluster of Protein TSSC1	TSSC1	46 kDa	0.80	0.02	1.80	0.16	0.44	0.00
O15240	Neurosecretory protein VGF	VGF	67 kDa	0.80	0.02	0.65	0.09	1.23	0.09
Q9NQT5	Exosome complex component RRP40	EXOSC3	30 kDa	0.80	0.02	1.30	0.20	0.62	0.00
B7WP27	Pre-mRNA-splicing factor CWC22 homolog	CWC22	106 kDa	0.80	0.02	1.50	0.13	0.53	0.00
E5RJD8	Cluster of Tubulin-specific chaperone A	TBCA	14 kDa	0.80	0.02	0.90	0.00	0.89	0.09
P15529	Membrane cofactor protein	CD46	44 kDa	0.80	0.02	1.10	0.00	0.73	0.01
Q13951	Core-binding factor subunit beta	CBFB	22 kDa	0.80	0.02	1.15	0.50	0.70	0.03
H3BPJ9	Cluster of NADH dehydrogenase [ubiquinone] 1 beta subcor	NDUFB10	19 kDa	0.80	0.02	1.25	0.34	0.64	0.02
Q9P2J5	Leucine--tRNA ligase, cytoplasmic	LARS	134 kDa	0.80	0.02	0.45	0.06	1.78	0.01
Q96I24	Far upstream element-binding protein 3	FUBP3	62 kDa	0.80	0.02	1.00	1.00	0.80	0.08
Q96DA6	Mitochondrial import inner membrane translocase subunit T	DNAJC19	12 kDa	0.80	0.02	1.55	0.17	0.52	0.00
Q8NC51	Isoform 2 of Plasminogen activator inhibitor 1 RNA-binding	SERBP1	44 kDa	0.80	0.02	0.55	0.07	1.45	0.02
J3KR78	Queuine tRNA-ribosyltransferase subunit QTRTD1	QTRTD1	48 kDa	0.80	0.02	1.75	0.13	0.46	0.00
Q92485	Cluster of Acid sphingomyelinase-like phosphodiesterase 3b	SMPDL3B	51 kDa	0.80	0.02	1.20	0.30	0.67	0.01
H0Y360	Cluster of AMP deaminase 2 (Fragment)	AMPD2	99 kDa	0.80	0.02	0.65	0.09	1.23	0.09
Q04760	Lactoylglutathione lyase	GLO1	21 kDa	0.80	0.02	1.30	0.20	0.62	0.00
Q96IZ0	PRKC apoptosis WT1 regulator protein	PAWR	37 kDa	0.80	0.02	1.40	0.16	0.57	0.00
B4DLN1	Cluster of Mitochondrial dicarboxylate carrier	SLC25A10	48 kDa	0.80	0.02	0.75	0.13	1.07	0.51
P36404	ADP-ribosylation factor-like protein 2	ARL2	21 kDa	0.80	0.02	1.50	0.13	0.53	0.00
O60220	Mitochondrial import inner membrane translocase subunit T	TIMM8A	11 kDa	0.80	0.02	1.05	0.50	0.76	0.02
F5H1Y4	Golgi-associated PDZ and coiled-coil motif-containing prote	GOPC	51 kDa	0.80	0.02	1.15	0.20	0.70	0.01
Q9H993	UPF0364 protein C6orf211	C6orf211	51 kDa	0.80	0.02	2.70	0.04	0.30	0.00
Q9BYN0	Sulfiredoxin-1	SRXN1	14 kDa	0.80	0.02	1.45	0.07	0.55	0.00
H0Y626	Uncharacterized protein		108 kDa	0.80	0.02	1.45	0.07	0.55	0.00
O75449	Cluster of Katanin p60 ATPase-containing subunit A1	KATNA1	56 kDa	0.80	0.02	1.75	0.04	0.46	0.00
Q49AR2	UPF0489 protein C5orf22	C5orf22	50 kDa	0.80	0.02	1.85	0.04	0.43	0.00
C9JWR9	3-phosphoinositide-dependent protein kinase 1	PDPK1	61 kDa	0.80	0.02	1.95	0.03	0.41	0.00
Q9BQ52	Cluster of Zinc phosphodiesterase ELAC protein 2	ELAC2	92 kDa	0.80	0.02	0.90	0.00	0.89	0.09
Q96FK6	WD repeat-containing protein 89	WDR89	43 kDa	0.80	0.02	1.10	0.00	0.73	0.01

Q8IYB8	ATP-dependent RNA helicase SUPV3L1, mitochondrial	SUPV3L1	88 kDa	0.80	0.02	1.70	0.00	0.47	0.00
Q92466	DNA damage-binding protein 2	DDB2	48 kDa	0.80	0.02	0.80	0.00	1.00	1.00
Q9Y2S2	Lambda-crystallin homolog	CRYL1	35 kDa	1.68	0.00	1.05	0.50	1.60	0.01
O15397	Cluster of Importin-8	IPO8	120 kDa	1.48	0.01	0.95	0.50	1.55	0.01
Q9H814	Phosphorylated adapter RNA export protein	PHAX	44 kDa	1.48	0.01	1.15	0.20	1.28	0.05
P10619	Lysosomal protective protein	CTSA	54 kDa	1.48	0.01	5.65	0.01	0.26	0.00
O43688	Isoform 2 of Lipid phosphate phosphohydrolase 2	PPAP2C	34 kDa	2.85	0.00	2.05	0.09	1.39	0.03
Q9H583	HEAT repeat-containing protein 1	HEATR1	242 kDa	1.40	0.01	1.40	0.16	1.00	1.00
D6RJA0	Histone-lysine N-methyltransferase SETD7	SETD7	40 kDa	1.40	0.01	1.05	0.50	1.33	0.03
Q8TEQ6	Gem-associated protein 5	GEMIN5	169 kDa	1.40	0.01	0.60	0.00	2.33	0.00
P62249	40S ribosomal protein S16	RPS16	16 kDa	1.73	0.00	0.70	0.00	2.46	0.00
Q99575	Ribonucleases P/MRP protein subunit POP1	POP1	115 kDa	0.98	0.64	0.75	0.34	1.30	0.13
P61160	Actin-related protein 2	ACTR2	45 kDa	0.98	0.64	0.70	0.20	1.39	0.04
Q6P1J9	Parafibromin	CDC73	61 kDa	0.98	0.64	1.45	0.20	0.67	0.02
Q9NWY4	UPF0609 protein C4orf27	C4orf27	39 kDa	0.98	0.64	1.60	0.11	0.61	0.00
O15511	Actin-related protein 2/3 complex subunit 5	ARPC5	16 kDa	0.98	0.64	0.85	0.20	1.15	0.18
Q96CN7	Isochorismatase domain-containing protein 1	ISOC1	32 kDa	0.98	0.64	0.95	0.50	1.03	0.76
B4DTE8	Phospholipid scramblase 1	PLSCR1	27 kDa	0.98	0.64	0.95	0.50	1.03	0.76
P46776	Cluster of 60S ribosomal protein L27a	RPL27A	17 kDa	0.98	0.64	1.05	0.50	0.93	0.39
P26373	60S ribosomal protein L13	RPL13	24 kDa	0.98	0.64	1.15	0.20	0.85	0.09
F8W785	Golgi integral membrane protein 4	GOLIM4	79 kDa	0.98	0.64	3.05	0.02	0.32	0.00
P49368	Cluster of T-complex protein 1 subunit gamma	CCT3	61 kDa	0.98	0.64	0.30	0.00	3.25	0.00
P67809	Cluster of Nuclease-sensitive element-binding protein 1	YBX1	36 kDa	0.98	0.64	0.60	0.00	1.63	0.00
Q9Y446	Plakophilin-3	PKP3	87 kDa	0.98	0.64	1.00	1.00	0.98	0.64
Q9HB71	Calcyclin-binding protein	CACYBP	26 kDa	0.98	0.64	0.60	0.00	1.63	0.00
Q9NRW7	Cluster of Vacuolar protein sorting-associated protein 45	VPS45	65 kDa	0.98	0.64	0.80	0.00	1.22	0.04
O60869	Endothelial differentiation-related factor 1	EDF1	16 kDa	0.98	0.64	0.90	0.00	1.08	0.22
O95602	DNA-directed RNA polymerase I subunit RPA1	POLR1A	195 kDa	0.98	0.64	0.90	0.00	1.08	0.22
Q9NYC9	Dynein heavy chain 9, axonemal	DNAH9	512 kDa	0.98	0.64	1.30	0.00	0.75	0.01
E7ESJ7	Protein FAM114A2	FAM114A2	48 kDa	0.98	0.64	1.40	0.00	0.70	0.00
Q8TCS8	Cluster of Polyribonucleotide nucleotidyltransferase 1, mitoc	PNPT1	86 kDa	0.98	0.64	0.85	0.50	1.15	0.34
E9PDI2	Adenylyl cyclase-associated protein	CAP2	50 kDa	0.98	0.64	1.25	0.34	0.78	0.08
I3L430	Cluster of Heme oxygenase 2	HMOX2	33 kDa	0.98	0.64	1.10	0.50	0.89	0.26
O60841	Eukaryotic translation initiation factor 5B	EIF5B	139 kDa	0.98	0.64	0.55	0.07	1.77	0.01
Q9NR30	Cluster of Nucleolar RNA helicase 2	DDX21	87 kDa	0.98	0.64	0.55	0.07	1.77	0.01
Q9Y287	Integral membrane protein 2B	ITM2B	30 kDa	0.98	0.64	1.10	0.50	0.89	0.26
Q10471	Cluster of Polypeptide N-acetylgalactosaminyltransferase 2	GALNT2	65 kDa	0.98	0.64	0.75	0.13	1.30	0.04

Q96I25	Splicing factor 45	RBM17	45 kDa	0.98	0.64	1.25	0.13	0.78	0.02
Q96FJ0	AMSH-like protease	STAMBPL1	50 kDa	0.98	0.64	2.25	0.03	0.43	0.00
O95817	BAG family molecular chaperone regulator 3	BAG3	62 kDa	0.98	0.64	0.90	0.00	1.08	0.22
P24752	Cluster of Acetyl-CoA acetyltransferase, mitochondrial	ACAT1	45 kDa	0.98	0.64	0.80	0.00	1.22	0.04
Q14232	Cluster of Translation initiation factor eIF-2B subunit alpha	EIF2B1	34 kDa	0.98	0.64	0.60	0.00	1.63	0.00
A6NDW3	Cluster of Lipolysis-stimulated lipoprotein receptor	LSR	64 kDa	0.98	0.64	0.60	0.00	1.63	0.00
Q8NBF2	NHL repeat-containing protein 2	NHLRC2	79 kDa	0.98	0.64	0.80	0.00	1.22	0.04
Q8NG11	Tetraspanin-14	TSPAN14	31 kDa	0.98	0.64	0.80	0.00	1.22	0.04
Q15435	Cluster of Protein phosphatase 1 regulatory subunit 7	PPP1R7	42 kDa	0.98	0.64	0.90	0.00	1.08	0.22
Q6NZI2	Polymerase I and transcript release factor	PTRF	43 kDa	0.98	0.64	0.90	0.00	1.08	0.22
H7BYF2	Nucleoporin p58/p45 (Fragment)	NUPL1	53 kDa	0.98	0.64	1.00	1.00	0.98	0.64
Q15942	Cluster of Zyxin	ZYX	61 kDa	0.98	0.64	0.75	0.13	1.30	0.04
E9PCK7	RRP12-like protein	RRP12	137 kDa	0.98	0.64	1.15	0.20	0.85	0.09
E9PDL6	Protein NDRG1	NDRG1	35 kDa	0.98	0.64	1.15	0.20	0.85	0.09
Q96NB3	Zinc finger protein 830	ZNF830	42 kDa	0.98	0.64	1.75	0.04	0.56	0.00
Q9NYL9	Cluster of Tropomodulin-3	TMOD3	40 kDa	0.98	0.64	0.80	0.00	1.22	0.04
Q9UKK9	Cluster of ADP-sugar pyrophosphatase	NUDT5	24 kDa	0.98	0.64	0.80	0.00	1.22	0.04
B4DQI4	Abhydrolase domain-containing protein 14B	ABHD14B	20 kDa	0.98	0.64	1.20	0.00	0.81	0.02
Q9UBS3	DnaJ homolog subfamily B member 9	DNAJB9	26 kDa	0.98	0.64	1.90	0.00	0.51	0.00
Q5TEC6	Cluster of Histone H3	HIST2H3PS2	15 kDa	1.20	0.04	0.50	0.00	2.40	0.00
P50502	Cluster of Hsc70-interacting protein	ST13	41 kDa	1.20	0.04	0.40	0.11	3.00	0.00
Q3ZCQ8	Isoform 2 of Mitochondrial import inner membrane transloca	TIMM50	50 kDa	1.20	0.04	2.10	0.06	0.57	0.00
Q9Y6W5	Wiskott-Aldrich syndrome protein family member 2	WASF2	54 kDa	1.20	0.04	1.15	0.20	1.04	0.62
P62857	40S ribosomal protein S28	RPS28	8 kDa	1.20	0.04	1.10	0.00	1.09	0.18
Q16539	Mitogen-activated protein kinase 14	MAPK14	41 kDa	1.20	0.04	1.40	0.00	0.86	0.04
O75143	Cluster of Isoform 3 of Autophagy-related protein 13	ATG13	44 kDa	1.79	0.00	2.16	0.05	0.83	0.15
P24666	Low molecular weight phosphotyrosine protein phosphatase	ACP1	18 kDa	1.35	0.01	2.15	0.08	0.63	0.00
O95628	Cluster of Isoform 6 of CCR4-NOT transcription complex su	CNOT4	85 kDa	1.35	0.01	1.65	0.14	0.82	0.09
Q8N684	Cluster of Isoform 3 of Cleavage and polyadenylation specif	CPSF7	56 kDa	1.35	0.01	1.15	0.20	1.17	0.12
E5RJP2	Cluster of Vinexin (Fragment)	SORBS3	20 kDa	1.35	0.01	1.30	0.00	1.04	0.50
Q9UBW8	COP9 signalosome complex subunit 7a	COPS7A	30 kDa	1.35	0.01	0.80	0.00	1.69	0.00
Q70IA6	MOB kinase activator 2	MOB2	27 kDa	1.35	0.01	1.30	0.00	1.04	0.50
Q92626	Cluster of Peroxidasin homolog	PXDN	165 kDa	1.05	0.39	0.70	0.20	1.50	0.02
Q86X76	Cluster of Nitrilase homolog 1	NIT1	36 kDa	1.05	0.39	1.05	0.80	1.00	1.00
O14976	Cyclin-G-associated kinase	GAK	143 kDa	1.05	0.39	1.45	0.07	0.72	0.01
Q9Y3C6	Peptidyl-prolyl cis-trans isomerase-like 1	PPIL1	18 kDa	1.05	0.39	1.00	1.00	1.05	0.39
A8MX29	Kinesin light chain 2	KLC2	37 kDa	1.05	0.39	1.35	0.26	0.78	0.06

J3KMX1	Cluster of Nuclear pore complex protein Nup88 (Fragment)	NUP88	79 kDa	1.05	0.39	1.30	0.20	0.81	0.06
Q9NQT4	Exosome complex component RRP46	EXOSC5	25 kDa	1.05	0.39	0.85	0.20	1.24	0.07
Q9H2P0	Activity-dependent neuroprotector homeobox protein	ADNP	124 kDa	1.05	0.39	0.95	0.50	1.11	0.28
H3BV69	Cluster of Cytochrome c oxidase subunit 5A, mitochondrial	COX5A	12 kDa	1.05	0.39	1.05	0.50	1.00	1.00
P08651	Cluster of Nuclear factor 1 C-type	NFIC	56 kDa	1.05	0.39	1.05	0.50	1.00	1.00
Q5JVF3	Isoform 4 of PCI domain-containing protein 2	PCID2	52 kDa	1.58	0.00	1.00	1.00	1.58	0.00
O00471	Exocyst complex component 5	EXOC5	82 kDa	1.05	0.39	1.35	0.26	0.78	0.06
P45974	Ubiquitin carboxyl-terminal hydrolase 5	USP5	96 kDa	1.05	0.39	0.55	0.07	1.91	0.00
Q9UJJ9	N-acetylglucosamine-1-phosphotransferase subunit gamma	GNPTG	34 kDa	1.05	0.39	0.85	0.20	1.24	0.07
E7EP65 [21]	Cluster of Abl interactor 2 (Fragment)	ABI2	53 kDa	1.05	0.39	0.95	0.50	1.11	0.28
Q15276	Rab GTPase-binding effector protein 1	RABEP1	99 kDa	1.05	0.39	1.25	0.13	0.84	0.07
Q5MNZ6	Cluster of WD repeat domain phosphoinositide-interacting p	WDR45B	38 kDa	1.05	0.39	1.00	1.00	1.05	0.39
Q15785	Mitochondrial import receptor subunit TOM34	TOMM34	35 kDa	0.53	0.00	1.20	0.00	0.44	0.00
E9PGP2	Coagulation factor XIa heavy chain	F11	64 kDa	1.58	0.00	1.95	0.10	0.81	0.06
P19838	Cluster of Nuclear factor NF-kappa-B p105 subunit	NFKB1	105 kDa	1.33	0.01	1.05	0.50	1.26	0.05
D6RHI9	Cluster of Ribonuclease T2 (Fragment)	RNASET2	29 kDa	1.33	0.01	1.25	0.13	1.06	0.49
J3KR35	Coiled-coil domain containing 12, isoform CRA_a	CCDC12	21 kDa	1.33	0.01	3.70	0.02	0.36	0.00
Q8N7H5	RNA polymerase II-associated factor 1 homolog	PAF1	60 kDa	1.50	0.01	0.75	0.13	2.00	0.00
Q6P2E9	Enhancer of mRNA-decapping protein 4	EDC4	152 kDa	1.50	0.01	1.00	1.00	1.50	0.01
Q13444	Cluster of Isoform 6 of Disintegrin and metalloproteinase do	ADAM15	89 kDa	1.03	0.64	1.00	1.00	1.03	0.87
Q96KP4	Cluster of Cytosolic non-specific dipeptidase	CNDP2	53 kDa	1.03	0.64	0.60	0.16	1.71	0.01
Q9H1Y0	Autophagy protein 5	ATG5	32 kDa	1.03	0.64	0.95	0.80	1.08	0.56
J3KMX4	Ragulator complex protein LAMTOR3	LAMTOR3	13 kDa	1.03	0.64	1.65	0.23	0.62	0.02
Q12974	Cluster of Protein tyrosine phosphatase type IVA 2	PTP4A2	19 kDa	1.03	0.64	1.65	0.14	0.62	0.01
Q9H936	Cluster of Mitochondrial glutamate carrier 1	SLC25A22	34 kDa	1.03	0.64	1.10	0.50	0.93	0.47
P78527	DNA-dependent protein kinase catalytic subunit	PRKDC	469 kDa	1.03	0.64	0.55	0.07	1.86	0.00
F5H8D7	DNA repair protein XRCC1	XRCC1	66 kDa	1.03	0.64	1.20	0.30	0.85	0.14
P30086	Phosphatidylethanolamine-binding protein 1	PEBP1	21 kDa	1.03	0.64	0.65	0.09	1.58	0.01
E7ETB3	Cluster of Aspartyl aminopeptidase	DNPEP	55 kDa	1.03	0.64	0.65	0.09	1.58	0.01
Q9BQC3	Diphthamide biosynthesis protein 2	DPH2	52 kDa	1.03	0.64	2.15	0.08	0.48	0.00
Q15424	Cluster of Scaffold attachment factor B1	SAFB	103 kDa	1.03	0.64	0.75	0.13	1.37	0.02
F5H6J9	Activating signal cointegrator 1 complex subunit 2	ASCC2	77 kDa	1.03	0.64	2.25	0.08	0.46	0.00
Q9NTM9	Copper homeostasis protein cutC homolog	CUTC	29 kDa	1.03	0.64	0.85	0.20	1.21	0.09
Q9NP79	Vacuolar protein sorting-associated protein VTA1 homolog	VTA1	34 kDa	1.03	0.64	0.85	0.20	1.21	0.09
P50281	Matrix metalloproteinase-14	MMP14	66 kDa	1.03	0.64	1.05	0.50	0.98	0.76
B7ZKQ9	SCARB1 protein	SCARB1	54 kDa	1.03	0.64	1.05	0.50	0.98	0.76
Q13442	28 kDa heat- and acid-stable phosphoprotein	PDAP1	21 kDa	1.03	0.64	1.25	0.13	0.82	0.04

P14222	Perforin-1	PRF1	61 kDa	1.03	0.64	1.45	0.07	0.71	0.01
Q9NZV1	Cysteine-rich motor neuron 1 protein	CRIM1	114 kDa	1.03	0.64	0.60	0.00	1.71	0.00
Q07065	Cytoskeleton-associated protein 4	CKAP4	66 kDa	1.03	0.64	0.80	0.00	1.28	0.02
P55001	Microfibrillar-associated protein 2	MFAP2	21 kDa	1.03	0.64	1.10	0.00	0.93	0.22
Q15637	Cluster of Isoform 3 of Splicing factor 1	SF1	67 kDa	1.03	0.64	0.80	0.00	1.28	0.02
Q9NZB2	Cluster of Isoform F of Constitutive coactivator of PPAR-ga	FAM120A	125 kDa	1.03	0.64	0.90	0.00	1.14	0.08
Q9ULF5	Zinc transporter ZIP10	SLC39A10	94 kDa	1.03	0.64	0.90	0.00	1.14	0.08
Q92541	Cluster of RNA polymerase-associated protein RTF1 homolo	RTF1	80 kDa	1.03	0.64	1.00	1.00	1.03	0.64
E5RJA0	Acyl-protein thioesterase 1	LYPLA1	15 kDa	1.03	0.64	1.10	0.00	0.93	0.22
Q9UBV8	Peflin	PEF1	30 kDa	1.03	0.64	1.40	0.00	0.73	0.00
P42574	Cluster of Caspase-3	CASP3	32 kDa	1.03	0.64	1.40	0.00	0.73	0.00
F6SBX2	Isoleucine--tRNA ligase, mitochondrial	IARS2	106 kDa	1.03	0.64	0.90	0.00	1.14	0.08
O43681	ATPase ASNA1	ASNA1	39 kDa	1.03	0.64	1.00	1.00	1.03	0.64
P49441	Inositol polyphosphate 1-phosphatase	INPP1	44 kDa	1.03	0.64	1.60	0.00	0.64	0.00
O43395	Cluster of U4/U6 small nuclear ribonucleoprotein Prp3	PRPF3	78 kDa	1.03	0.64	1.00	1.00	1.03	0.64
H3BTH8	Hyaluronan and proteoglycan link protein 3	HAPLN3	48 kDa	1.03	0.64	1.20	0.00	0.85	0.04
Q9NP77	Cluster of RNA polymerase II subunit A C-terminal domain	SSU72	23 kDa	1.03	0.64	1.60	0.00	0.64	0.00
H3BM74	NEDD8 ultimate buster 1	NUB1	73 kDa	1.03	0.64	1.80	0.00	0.57	0.00
P42704	Cluster of Leucine-rich PPR motif-containing protein, mitocl	LRPPRC	158 kDa	1.03	0.64	0.75	0.34	1.37	0.08
P13984	General transcription factor IIF subunit 2	GTF2F2	28 kDa	1.03	0.64	1.00	1.00	1.03	0.80
P61221	ATP-binding cassette sub-family E member 1	ABCE1	67 kDa	1.03	0.64	0.65	0.09	1.58	0.01
P04040	Catalase	CAT	60 kDa	1.03	0.64	0.85	0.20	1.21	0.09
F5H897	Cluster of Heat shock protein 75 kDa, mitochondrial	TRAP1	74 kDa	1.03	0.64	0.60	0.00	1.71	0.00
O60678	Protein arginine N-methyltransferase 3	PRMT3	60 kDa	1.03	0.64	0.90	0.00	1.14	0.08
F8VY86	Tumor suppressor p53-binding protein 1	TP53BP1	214 kDa	1.03	0.64	1.30	0.00	0.79	0.01
Q9P287	Cluster of BRCA2 and CDKN1A-interacting protein	BCCIP	36 kDa	1.63	0.00	0.65	0.09	2.50	0.00
O75390	Cluster of Citrate synthase, mitochondrial	CS	52 kDa	1.38	0.01	0.65	0.09	2.12	0.00
P62829	Cluster of 60S ribosomal protein L23	RPL23	15 kDa	0.90	0.09	0.85	0.50	1.06	0.67
O43353	Cluster of Receptor-interacting serine/threonine-protein kina	RIPK2	61 kDa	0.90	0.09	1.70	0.18	0.53	0.00
P04080	Cystatin-B	CSTB	11 kDa	0.90	0.09	0.90	0.50	1.00	1.00
Q9BRX5	DNA replication complex GINS protein PSF3	GINS3	25 kDa	0.90	0.09	0.95	0.50	0.95	0.51
A6NJ11	Ubiquitin fusion degradation protein 1 homolog	UFD1L	33 kDa	0.90	0.09	0.95	0.50	0.95	0.51
B7Z3P1	Tubulin-specific chaperone E	TBCE	65 kDa	0.90	0.09	0.95	0.50	0.95	0.51
J3KNF4	Cluster of Copper chaperone for superoxide dismutase	CCS	27 kDa	0.90	0.09	1.05	0.50	0.86	0.09
Q86V48	Cluster of Leucine zipper protein 1	LUZP1	120 kDa	0.90	0.09	1.05	0.50	0.86	0.09
H0Y7W6	Cluster of Formin-binding protein 1 (Fragment)	FNBP1	66 kDa	0.90	0.09	1.05	0.50	0.86	0.09
P51116	Fragile X mental retardation syndrome-related protein 2	FXR2	74 kDa	0.90	0.09	1.15	0.20	0.78	0.02

Q92542	Nicastrin	NCSTN	78 kDa	0.90	0.09	1.15	0.20	0.78	0.02
Q13112	Chromatin assembly factor 1 subunit B	CHAF1B	61 kDa	0.90	0.09	1.25	0.13	0.72	0.01
G5E934	Cluster of Cancer susceptibility candidate 4, isoform CRA_b	CASC4	49 kDa	0.90	0.09	1.45	0.07	0.62	0.00
Q9C0B0	RING finger protein unkempt homolog	UNK	88 kDa	0.90	0.09	1.75	0.04	0.51	0.00
J3QS36	Cluster of L-xylulose reductase (Fragment)	DCXR	24 kDa	0.90	0.09	0.90	0.00	1.00	1.00
Q9H773	Cluster of dCTP pyrophosphatase 1	DCTPP1	19 kDa	0.90	0.09	1.00	1.00	0.90	0.09
Q86SQ4	Isoform 3 of G-protein coupled receptor 126	GPR126	140 kDa	0.90	0.09	0.70	0.00	1.29	0.02
B0QZ18	Cluster of Copine I	CPNE1	60 kDa	0.90	0.09	0.80	0.00	1.13	0.09
P39019	40S ribosomal protein S19	RPS19	16 kDa	0.90	0.09	0.80	0.00	1.13	0.09
Q9UBB6	Cluster of Isoform 3 of Neurochondrin	NCDN	79 kDa	0.90	0.09	1.00	1.00	0.90	0.09
Q03135	Caveolin-1	CAV1	20 kDa	0.90	0.09	1.10	0.00	0.82	0.02
Q86W92	Cluster of Isoform 2 of Liprin-beta-1	PPFIBP1	113 kDa	0.90	0.09	1.65	0.31	0.55	0.27
Q9H2C0	Gigaxonin	GAN	68 kDa	0.90	0.09	0.95	0.80	0.95	0.67
Q5QPH3	Cluster of Acetyl-coenzyme A synthetase, cytoplasmic	ACSS2	80 kDa	0.90	0.09	1.05	0.80	0.86	0.25
O95429	BAG family molecular chaperone regulator 4	BAG4	50 kDa	0.90	0.09	5.05	0.10	0.18	0.10
Q14978	Isoform Beta of Nucleolar and coiled-body phosphoprotein 1	NOLC1	75 kDa	0.90	0.09	0.45	0.06	2.00	0.00
Q14974	Cluster of Importin subunit beta-1	KPNB1	97 kDa	0.90	0.09	0.45	0.06	2.00	0.00
P51572	B-cell receptor-associated protein 31	BCAP31	28 kDa	0.90	0.09	0.90	0.50	1.00	1.00
Q8IYS1	Peptidase M20 domain-containing protein 2	PM20D2	48 kDa	0.90	0.09	1.00	1.00	0.90	0.31
Q69YN4	Protein virilizer homolog	KIAA1429	202 kDa	0.90	0.09	1.00	1.00	0.90	0.31
P30050	60S ribosomal protein L12	RPL12	18 kDa	0.90	0.09	0.55	0.07	1.64	0.01
Q5TFE4	Cluster of 5'-nucleotidase domain-containing protein 1	NT5DC1	52 kDa	0.90	0.09	1.10	0.50	0.82	0.08
A8MZ87	Cluster of Kinesin light chain 2	KLC2	57 kDa	0.90	0.09	0.55	0.07	1.64	0.01
E9PGF9	Protein O-GlcNAcase	MGEA5	97 kDa	0.90	0.09	0.65	0.09	1.38	0.02
P47755	F-actin-capping protein subunit alpha-2	CAPZA2	33 kDa	0.90	0.09	0.75	0.13	1.20	0.09
Q9C0C9	Ubiquitin-conjugating enzyme E2 O	UBE2O	141 kDa	0.90	0.09	0.75	0.13	1.20	0.09
J3KTE4	Ribosomal protein L19	RPL19	23 kDa	0.90	0.09	0.85	0.20	1.06	0.51
P52735	Isoform 3 of Guanine nucleotide exchange factor VAV2	VAV2	97 kDa	0.90	0.09	0.85	0.20	1.06	0.51
Q08170	Cluster of Serine/arginine-rich splicing factor 4	SRSF4	57 kDa	0.90	0.09	0.85	0.20	1.06	0.51
O60216	Double-strand-break repair protein rad21 homolog	RAD21	72 kDa	0.90	0.09	0.85	0.20	1.06	0.51
Q96FZ7	Cluster of Charged multivesicular body protein 6	CHMP6	23 kDa	0.90	0.09	1.70	0.09	0.53	0.00
P04066	Tissue alpha-L-fucosidase	FUCA1	54 kDa	0.90	0.09	0.95	0.50	0.95	0.51
Q9UKM7	Cluster of Endoplasmic reticulum mannosyl-oligosaccharide	MAN1B1	80 kDa	0.90	0.09	1.05	0.50	0.86	0.09
Q9Y696	Chloride intracellular channel protein 4	CLIC4	29 kDa	0.90	0.09	1.05	0.50	0.86	0.09
P13995	Cluster of Bifunctional methylenetetrahydrofolate dehydroge	MTHFD2	38 kDa	0.90	0.09	1.15	0.20	0.78	0.02
Q5R372	Cluster of Isoform 8 of Rab GTPase-activating protein 1-like	RABGAP1L	43 kDa	0.90	0.09	1.15	0.20	0.78	0.02
P83436	Conserved oligomeric Golgi complex subunit 7	COG7	86 kDa	0.90	0.09	1.15	0.20	0.78	0.02

D6RFG8	Deoxycytidine kinase	DCK	37 kDa	0.90	0.09	1.25	0.13	0.72	0.01
Q96CT7	Coiled-coil domain-containing protein 124	CCDC124	26 kDa	0.90	0.09	1.35	0.09	0.67	0.00
Q4G0F5	Vacuolar protein sorting-associated protein 26B	VPS26B	39 kDa	0.90	0.09	1.45	0.07	0.62	0.00
P55196	Cluster of Isoform 2 of Afadin	MLLT4	206 kDa	0.90	0.09	1.55	0.06	0.58	0.00
F5GX65	Armadillo repeat-containing protein 10	ARMC10	25 kDa	0.90	0.09	1.65	0.05	0.55	0.00
H0YJ66	Dehydrogenase/reductase SDR family member 7 (Fragment)	DHRS7	45 kDa	0.90	0.09	1.75	0.04	0.51	0.00
A2RUC4	Cluster of tRNA wybutosine-synthesizing protein 5	TYW5	37 kDa	0.90	0.09	1.85	0.04	0.49	0.00
P05386	60S acidic ribosomal protein P1	RPLP1	12 kDa	0.90	0.09	0.70	0.00	1.29	0.02
E7EP00	Protein transport protein Sec24C	SEC24C	107 kDa	0.90	0.09	0.60	0.00	1.50	0.01
Q12792	Isoform 4 of Twinfilin-1	TWF1	29 kDa	0.90	0.09	0.80	0.00	1.13	0.09
Q8N0X4	Citrate lyase subunit beta-like protein, mitochondrial	CLYBL	37 kDa	0.90	0.09	1.90	0.00	0.47	0.00
G3V1S2	Cullin-2	CUL2	89 kDa	0.90	0.09	0.60	0.00	1.50	0.01
Q13547	Cluster of Histone deacetylase 1	HDAC1	55 kDa	0.90	0.09	0.80	0.00	1.13	0.09
P62079	Tetraspanin-5	TSPAN5	30 kDa	0.90	0.09	0.90	0.00	1.00	1.00
E9PRZ1	Cluster of Protein SAAL1	SAAL1	54 kDa	0.90	0.09	1.00	1.00	0.90	0.09
Q14195	Isoform LCRMP-4 of Dihydropyrimidinase-related protein 3	DPYSL3	74 kDa	0.90	0.09	1.30	0.00	0.69	0.00
J3KPZ4	Nuclear nucleic acid-binding protein C1D	C1D	20 kDa	0.90	0.09	1.40	0.00	0.64	0.00
Q01432	AMP deaminase 3	AMPD3	89 kDa	0.90	0.09	1.70	0.00	0.53	0.00
Q8NFQ8	Torsin-1A-interacting protein 2	TOR1AIP2	51 kDa	0.90	0.09	2.50	0.00	0.36	0.00
J3KQG6	Cluster of Eukaryotic peptide chain release factor GTP-bindi	GSPT1	69 kDa	0.90	0.09	0.60	0.00	1.50	0.01
P00390	Glutathione reductase, mitochondrial	GSR	56 kDa	0.90	0.09	0.60	0.00	1.50	0.01
O43815	Striatin	STRN	86 kDa	0.90	0.09	1.00	1.00	0.90	0.09
O95292	Vesicle-associated membrane protein-associated protein B/C	VAPB	27 kDa	0.90	0.09	1.00	1.00	0.90	0.09
Q13243	Serine/arginine-rich splicing factor 5	SRSF5	31 kDa	0.90	0.09	1.10	0.00	0.82	0.02
P49589	Cluster of Isoform 3 of Cysteine--tRNA ligase, cytoplasmic	CARS	95 kDa	1.08	0.22	0.25	0.04	4.30	0.00
Q9NYU2	Cluster of UDP-glucose:glycoprotein glucosyltransferase 1	UGGT1	177 kDa	1.08	0.22	0.70	0.20	1.54	0.02
H0YH87	Cluster of Ataxin-2 (Fragment)	ATXN2	98 kDa	1.08	0.22	1.65	0.14	0.65	0.01
Q68E01	Isoform 2 of Integrator complex subunit 3	INTS3	118 kDa	1.08	0.22	1.10	0.50	0.98	0.80
Q3MHD2	Protein LSM12 homolog	LSM12	22 kDa	1.08	0.22	1.20	0.30	0.90	0.26
Q93034	Cluster of Cullin-5	CUL5	91 kDa	1.08	0.22	0.65	0.09	1.65	0.01
Q96S59	Ran-binding protein 9	RANBP9	78 kDa	1.08	0.22	1.40	0.16	0.77	0.03
O75326	Semaphorin-7A	SEMA7A	75 kDa	1.08	0.22	0.75	0.13	1.43	0.01
P33947	ER lumen protein retaining receptor 2	KDELR2	24 kDa	1.08	0.22	1.60	0.11	0.67	0.01
E9PLI6	Probable RNA-binding protein EIF1AD (Fragment)	EIF1AD	13 kDa	1.08	0.22	1.80	0.08	0.60	0.00
Q96QC0	Serine/threonine-protein phosphatase 1 regulatory subunit 1(	PPP1R10	99 kDa	1.08	0.22	0.95	0.50	1.13	0.18
Q9UEW8	Cluster of STE20/SPS1-related proline-alanine-rich protein l	STK39	59 kDa	1.08	0.22	1.05	0.50	1.02	0.76
P06727	Apolipoprotein A-IV	APOA4	45 kDa	1.08	0.22	1.15	0.20	0.93	0.39

J3QQJ0	Cluster of SAP30-binding protein (Fragment)	SAP30BP	36 kDa	1.08	0.22	1.15	0.20	0.93	0.39
O14841	5-oxoprolinase	OPLAH	137 kDa	1.08	0.22	1.15	0.20	0.93	0.39
Q8TBB5	Kelch domain-containing protein 4	KLHDC4	58 kDa	1.08	0.22	1.25	0.13	0.86	0.09
Q9BQL6	Isoform 4 of Fermitin family homolog 1	FERMT1	57 kDa	1.08	0.22	1.45	0.07	0.74	0.01
Q13131	Isoform 2 of 5'-AMP-activated protein kinase catalytic subur	PRKAA1	66 kDa	1.08	0.22	1.55	0.06	0.69	0.00
H3BMS0	RNA-binding protein with serine-rich domain 1	RNPS1	27 kDa	1.08	0.22	1.65	0.05	0.65	0.00
Q9BQP7	Uncharacterized protein C20orf72	MGME1	39 kDa	1.08	0.22	2.15	0.03	0.50	0.00
P09497	Isoform Non-brain of Clathrin light chain B	CLTB	23 kDa	1.08	0.22	0.80	0.00	1.34	0.01
Q8WUA8	Tsukushin	TSKU	38 kDa	1.08	0.22	1.00	1.00	1.08	0.22
Q92538	Golgi-specific brefeldin A-resistance guanine nucleotide exc	GBF1	206 kDa	1.08	0.22	1.30	0.00	0.83	0.02
P46940	Cluster of Ras GTPase-activating-like protein IQGAP1	IQGAP1	189 kDa	1.08	0.22	0.30	0.00	3.58	0.00
Q13347	Eukaryotic translation initiation factor 3 subunit I	EIF3I	37 kDa	1.08	0.22	0.60	0.00	1.79	0.00
Q13177	Cluster of Serine/threonine-protein kinase PAK 2	PAK2	58 kDa	1.08	0.22	0.70	0.00	1.54	0.00
Q13363	Cluster of C-terminal-binding protein 1	CTBP1	48 kDa	1.08	0.22	0.70	0.00	1.54	0.00
Q29RF7	Cluster of Sister chromatid cohesion protein PDS5 homolog	PDS5A	151 kDa	1.08	0.22	0.90	0.00	1.19	0.04
P46976	Cluster of Glycogenin-1	GYG1	39 kDa	1.08	0.22	1.00	1.00	1.08	0.22
Q96EU7	C1GALT1-specific chaperone 1	C1GALT1C1	36 kDa	1.08	0.22	1.10	0.00	0.98	0.64
Q06033	Inter-alpha-trypsin inhibitor heavy chain H3	ITIH3	100 kDa	1.08	0.22	1.30	0.37	0.83	0.19
P28331	Isoform 2 of NADH-ubiquinone oxidoreductase 75 kDa subu	NDUFS1	81 kDa	1.08	0.22	0.90	0.50	1.19	0.14
Q9H6R7	WD repeat-containing protein C2orf44	C2orf44	79 kDa	1.08	0.22	2.20	0.11	0.49	0.00
P60953	Cluster of Cell division control protein 42 homolog	CDC42	21 kDa	1.08	0.22	0.65	0.09	1.65	0.01
Q16222	UDP-N-acetylhexosamine pyrophosphorylase	UAP1	59 kDa	1.08	0.22	0.85	0.20	1.26	0.04
P46939	Utrophin	UTRN	394 kDa	1.08	0.22	0.85	0.20	1.26	0.04
Q8N5I2	Arrestin domain-containing protein 1	ARRDC1	46 kDa	1.08	0.22	0.95	0.50	1.13	0.18
O94910	Latrophilin-1	LPHN1	163 kDa	1.08	0.22	1.25	0.13	0.86	0.09
P52272	Heterogeneous nuclear ribonucleoprotein M	HNRNPM	78 kDa	1.08	0.22	0.60	0.00	1.79	0.00
B7ZKJ8	Cluster of ITIH4 protein	ITIH4	104 kDa	1.08	0.22	0.80	0.00	1.34	0.01
A8MV58	Cluster of Drebrin	DBN1	76 kDa	1.08	0.22	0.80	0.00	1.34	0.01
P12110	Cluster of Collagen alpha-2(VI) chain	COL6A2	109 kDa	1.08	0.22	0.70	0.00	1.54	0.00
A8MUW5	Protein FAM98B	FAM98B	46 kDa	1.08	0.22	1.00	1.00	1.08	0.22
Q92614	Unconventional myosin-XVIIIa	MYO18A	233 kDa	1.45	0.01	0.90	0.00	1.61	0.00
Q13418	Cluster of Integrin-linked protein kinase	ILK	51 kDa	1.45	0.01	1.40	0.00	1.04	0.50
Q86UA1	Pre-mRNA-processing factor 39	PRPF39	78 kDa	1.45	0.01	1.40	0.16	1.04	0.69
Q14376	Cluster of UDP-glucose 4-epimerase	GALE	38 kDa	1.45	0.01	1.55	0.06	0.94	0.38
Q16831	Cluster of Uridine phosphorylase 1	UPP1	34 kDa	1.45	0.01	0.90	0.00	1.61	0.00
Q5SGD2	Protein phosphatase 1L	PPM1L	41 kDa	0.65	0.00	1.40	0.30	0.46	0.16
P49207	60S ribosomal protein L34	RPL34	13 kDa	0.65	0.00	0.80	0.30	0.81	0.12

P07686	Cluster of Beta-hexosaminidase subunit beta	HEXB	63 kDa	0.65	0.00	0.55	0.07	1.18	0.13
P30740	Cluster of Leukocyte elastase inhibitor	SERPINB1	43 kDa	0.65	0.00	0.65	0.09	1.00	1.00
C9J0D9	Complement receptor type 2	CR2	110 kDa	0.65	0.00	0.85	0.20	0.76	0.02
J3KP43	Ribonucleoside-diphosphate reductase subunit M2	RRM2	51 kDa	0.65	0.00	0.95	0.50	0.68	0.00
E7EQY1	Cluster of Protein FAM136A	FAM136A	27 kDa	0.65	0.00	1.45	0.07	0.45	0.00
Q6UVK1	Chondroitin sulfate proteoglycan 4	CSPG4	251 kDa	0.65	0.00	0.60	0.00	1.08	0.18
Q5VWZ2	Lysophospholipase-like protein 1	LYPLAL1	26 kDa	1.30	0.01	1.20	0.00	1.08	0.18
O75044	Cluster of SLIT-ROBO Rho GTPase-activating protein 2	SRGAP2	121 kDa	1.30	0.01	1.40	0.00	0.93	0.18
P46736	Isoform 1 of Lys-63-specific deubiquitinase BRCC36	BRCC3	33 kDa	0.65	0.00	1.05	0.50	0.62	0.00
P55011	Solute carrier family 12 member 2	SLC12A2	131 kDa	0.65	0.00	1.90	0.00	0.34	0.00
Q15847	Adipose most abundant gene transcript 2 protein	ADIRF	8 kDa	1.30	0.01	1.15	0.20	1.13	0.18
E9PEQ6	Peptidyl-prolyl cis-trans isomerase	PPIE	28 kDa	1.30	0.01	1.55	0.06	0.84	0.05
P06132	Cluster of Uroporphyrinogen decarboxylase	UROD	41 kDa	1.30	0.01	0.90	0.00	1.44	0.01
Q9NZ08	Endoplasmic reticulum aminopeptidase 1	ERAP1	107 kDa	1.30	0.01	1.00	1.00	1.30	0.01
Q6PCB0	von Willebrand factor A domain-containing protein 1	VWA1	47 kDa	1.60	0.00	1.30	0.00	1.23	0.02
Q9Y639	Cluster of Neuroplastin	NPTN	44 kDa	1.60	0.00	2.20	0.25	0.73	0.44
Q9UNS2	Cluster of Isoform 2 of COP9 signalosome complex subunit	COPS3	46 kDa	1.60	0.00	0.70	0.00	2.29	0.00
P06280	Alpha-galactosidase A	GLA	49 kDa	1.60	0.00	0.95	0.50	1.68	0.00
Q9H2G2	Cluster of Isoform 2 of STE20-like serine/threonine-protein	SLK	139 kDa	1.43	0.01	0.80	0.00	1.78	0.00
O60518	Ran-binding protein 6	RANBP6	125 kDa	1.43	0.01	0.95	0.50	1.50	0.01
P02649	Cluster of Apolipoprotein E	APOE	36 kDa	1.43	0.01	1.35	0.09	1.06	0.49
Q96T51	Cluster of RUN and FYVE domain-containing protein 1	RUFY1	80 kDa	1.43	0.01	0.90	0.00	1.58	0.00
G3XAK1	Cluster of Hepatocyte growth factor-like protein beta chain	MST1	82 kDa	1.43	0.01	1.20	0.00	1.19	0.04
O60911	Cathepsin L2	CTSV	37 kDa	2.18	0.00	1.15	0.66	1.89	0.01
P02452	Collagen alpha-1(I) chain	COL1A1	139 kDa	1.15	0.06	1.00	1.00	1.15	0.20
P49750	Isoform 4 of YLP motif-containing protein 1	YLPM1	242 kDa	1.15	0.06	1.20	0.30	0.96	0.63
P00505	Aspartate aminotransferase, mitochondrial	GOT2	48 kDa	0.58	0.00	0.65	0.09	0.88	0.20
Q9UBE0	Cluster of SUMO-activating enzyme subunit 1	SAE1	38 kDa	1.15	0.06	0.75	0.13	1.53	0.01
Q99519	Sialidase-1	NEU1	45 kDa	1.15	0.06	0.95	0.50	1.21	0.07
Q6NUK1	Calcium-binding mitochondrial carrier protein SCaMC-1	SLC25A24	53 kDa	1.15	0.06	1.85	0.04	0.62	0.00
Q9NWU2	Glucose-induced degradation protein 8 homolog	GID8	27 kDa	0.58	0.00	1.85	0.04	0.31	0.00
P46779	Cluster of 60S ribosomal protein L28	RPL28	16 kDa	1.15	0.06	0.90	0.00	1.28	0.02
O75607	Nucleoplasmin-3	NPM3	19 kDa	1.15	0.06	1.00	1.00	1.15	0.06
J3KNX3	Cluster of 6-phosphofructokinase	PFKM	93 kDa	1.15	0.06	0.80	0.00	1.44	0.01
E9PL57	Cluster of Protein NEDD8-MDP1 (Fragment)	NEDD8-MDP1	20 kDa	1.15	0.06	0.90	0.00	1.28	0.02
E9PHK0	Tetranectin	CLEC3B	18 kDa	1.73	0.00	0.85	0.20	2.03	0.00
P78417	Glutathione S-transferase omega-1	GSTO1	28 kDa	1.15	0.06	0.55	0.07	2.09	0.00

D3YTF8	Cluster of Thioredoxin reductase 2, mitochondrial	TXNRD2	54 kDa	1.15	0.06	1.95	0.10	0.59	0.00
Q86X55	Histone-arginine methyltransferase CARM1	CARM1	66 kDa	1.15	0.06	0.75	0.13	1.53	0.01
P60228	Cluster of Eukaryotic translation initiation factor 3 subunit E	EIF3E	52 kDa	1.15	0.06	6.75	0.02	0.17	0.00
B4DH53	Microtubule-associated protein 1S	MAP1S	110 kDa	1.15	0.06	1.10	0.00	1.05	0.39
P14678	Cluster of Isoform SM-B1 of Small nuclear ribonucleoprotei	SNRPB	30 kDa	1.15	0.06	0.70	0.00	1.64	0.00
P49821	NADH dehydrogenase [ubiquinone] flavoprotein 1, mitocho	NDUFV1	51 kDa	1.90	0.00	1.30	0.20	1.46	0.01
P33992	Cluster of DNA replication licensing factor MCM5	MCM5	82 kDa	1.48	0.00	0.60	0.16	2.46	0.00
J3KR72	Cluster of Transcription initiation factor TFIID subunit 6	TAF6	79 kDa	1.48	0.00	1.80	0.08	0.82	0.04
Q9H9B4	Cluster of Sideroflexin-1	SFXN1	36 kDa	1.48	0.00	1.00	1.00	1.48	0.00
Q9BQA1	Methylosome protein 50	WDR77	37 kDa	1.48	0.00	0.80	0.00	1.84	0.00
Q86SQ0	Cluster of Pleckstrin homology-like domain family B membe	PHLDB2	142 kDa	1.13	0.08	1.00	1.00	1.13	0.43
Q9H2P9	Diphthine synthase	DPH5	32 kDa	1.18	0.09	2.95	0.07	0.40	0.18
Q9UHD1	Cysteine and histidine-rich domain-containing protein 1	CHORDC1	37 kDa	1.13	0.08	0.45	0.06	2.50	0.00
P53999	Activated RNA polymerase II transcriptional coactivator p15	SUB1	14 kDa	1.13	0.08	0.90	0.50	1.25	0.08
Q7L985	Cluster of Leucine-rich repeat and immunoglobulin-like dom	LINGO2	68 kDa	1.13	0.08	1.80	0.16	0.63	0.01
J3QR68	Haptoglobin beta chain (Fragment)	HP	45 kDa	1.13	0.08	1.45	0.20	0.78	0.05
Q7Z4H8	Cluster of KDEL motif-containing protein 2	KDELC2	59 kDa	1.13	0.08	1.65	0.14	0.68	0.01
O15357	Phosphatidylinositol 3,4,5-trisphosphate 5-phosphatase 2	INPPL1	139 kDa	1.13	0.08	2.20	0.11	0.51	0.00
Q14320	Cluster of Protein FAM50A	FAM50A	40 kDa	1.13	0.08	1.20	0.30	0.94	0.47
O00203	Cluster of AP-3 complex subunit beta-1	AP3B1	121 kDa	1.13	0.08	0.65	0.09	1.73	0.00
P02794	Cluster of Ferritin heavy chain	FTH1	21 kDa	1.13	0.08	1.30	0.20	0.87	0.14
Q9H3U1	Isoform 2 of Protein unc-45 homolog A	UNC45A	102 kDa	1.13	0.08	0.75	0.13	1.50	0.01
E7EQ69	Cluster of N-alpha-acetyltransferase 50	NAA50	19 kDa	1.13	0.08	0.75	0.13	1.50	0.01
O43252	Bifunctional 3'-phosphoadenosine 5'-phosphosulfate synthas	PAPSS1	71 kDa	1.13	0.08	0.85	0.20	1.32	0.02
B4DKB2	Endothelin-converting enzyme 1	ECE1	84 kDa	1.13	0.08	1.80	0.08	0.63	0.00
Q15334	Lethal(2) giant larvae protein homolog 1	LLGL1	115 kDa	1.13	0.08	2.00	0.06	0.56	0.00
Q92522	Histone H1x	H1FX	22 kDa	1.13	0.08	1.05	0.50	1.07	0.39
J3KQ42	Tetraspanin-4	TSPAN4	28 kDa	1.13	0.08	1.05	0.50	1.07	0.39
Q9NT62	Ubiquitin-like-conjugating enzyme ATG3	ATG3	36 kDa	1.13	0.08	1.05	0.50	1.07	0.39
Q8NBN3	Transmembrane protein 87A	TMEM87A	63 kDa	1.13	0.08	2.20	0.05	0.51	0.00
P05546	Heparin cofactor 2	SERPIND1	57 kDa	1.13	0.08	1.15	0.20	0.98	0.76
E7EPD0	Cluster of Target of Myb protein 1	TOM1	50 kDa	1.13	0.08	2.70	0.04	0.42	0.00
Q9BRS2	Serine/threonine-protein kinase RIO1	RIOK1	66 kDa	1.13	0.08	2.45	0.02	0.46	0.00
Q9H9G7	Cluster of Protein argonaute-3	EIF2C3	97 kDa	1.13	0.08	0.80	0.00	1.41	0.01
Q9H0R4	Haloacid dehalogenase-like hydrolase domain-containing pro	HDHD2	29 kDa	1.13	0.08	1.20	0.00	0.94	0.22
B4DQU7	Cluster of Caspase-7 subunit p20	CASP7	32 kDa	1.13	0.08	1.20	0.00	0.94	0.22
P36873	Cluster of Isoform Gamma-2 of Serine/threonine-protein pho	PPP1CC	39 kDa	1.13	0.08	0.60	0.00	1.88	0.00

P11717	Cation-independent mannose-6-phosphate receptor	IGF2R	274 kDa	1.13	0.08	0.40	0.00	2.81	0.00
O43175	D-3-phosphoglycerate dehydrogenase	PHGDH	57 kDa	1.13	0.08	0.50	0.00	2.25	0.00
Q6ZMU5	Tripartite motif-containing protein 72	TRIM72	53 kDa	1.13	0.08	0.60	0.00	1.88	0.00
Q96T37	Cluster of Isoform 3 of Putative RNA-binding protein 15	RBM15	106 kDa	1.78	0.00	1.85	0.11	0.96	0.63
Q9Y5Z4	Heme-binding protein 2	HEBP2	23 kDa	1.78	0.00	1.70	0.18	1.04	0.68
P35573	Glycogen debranching enzyme	AGL	175 kDa	1.55	0.00	0.65	0.09	2.38	0.00
C9JA08	60S ribosomal export protein NMD3	NMD3	60 kDa	1.55	0.00	1.05	0.50	1.48	0.01
Q9Y3E7	Cluster of Isoform 4 of Charged multivesicular body protein	CHMP3	21 kDa	1.55	0.00	1.25	0.13	1.24	0.04
Q99543	DnaJ homolog subfamily C member 2	DNAJC2	72 kDa	1.70	0.00	1.10	0.00	1.55	0.00
Q86UY0	TXNDC5 protein	TXNDC5	40 kDa	1.53	0.00	0.65	0.09	2.35	0.00
C9JK10	Cluster of Integrin alpha-6 (Fragment)	ITGA6	122 kDa	1.40	0.01	0.65	0.09	2.15	0.00
Q96P16	Regulation of nuclear pre-mRNA domain-containing protein	RPRD1A	36 kDa	1.00	1.00	1.65	0.31	0.61	0.31
Q9BTV6	WD repeat-containing protein 85	DPH7	51 kDa	1.00	1.00	0.95	0.80	1.05	0.67
Q9H074	Polyadenylate-binding protein-interacting protein 1	PAIP1	54 kDa	1.00	1.00	0.70	0.20	1.43	0.03
P23560	Brain-derived neurotrophic factor	BDNF	28 kDa	1.00	1.00	1.55	0.17	0.65	0.01
P63241	Cluster of Isoform 2 of Eukaryotic translation initiation facto	EIF5A	20 kDa	1.00	1.00	0.65	0.09	1.54	0.01
O14929	Cluster of Histone acetyltransferase type B catalytic subunit	HAT1	50 kDa	1.00	1.00	0.65	0.09	1.54	0.01
Q08J23	tRNA (cytosine(34)-C(5))-methyltransferase	NSUN2	86 kDa	1.00	1.00	0.75	0.13	1.33	0.02
P05362	Intercellular adhesion molecule 1	ICAM1	58 kDa	1.00	1.00	0.75	0.13	1.33	0.02
P31949	Protein S100-A11	S100A11	12 kDa	1.00	1.00	0.75	0.13	1.33	0.02
Q15428	Splicing factor 3A subunit 2	SF3A2	49 kDa	1.00	1.00	0.75	0.13	1.33	0.02
P04843	Dolichyl-diphosphooligosaccharide--protein glycosyltransfer	RPN1	69 kDa	1.00	1.00	0.85	0.20	1.18	0.09
Q9NQR4	Cluster of Omega-amidase NIT2	NIT2	31 kDa	1.00	1.00	0.85	0.20	1.18	0.09
P06746	Cluster of DNA polymerase beta	POLB	38 kDa	1.00	1.00	1.15	0.20	0.87	0.09
Q9Y2P8	RNA 3'-terminal phosphate cyclase-like protein	RCL1	41 kDa	1.00	1.00	1.25	0.13	0.80	0.02
P56381	ATP synthase subunit epsilon, mitochondrial	ATP5E	6 kDa	1.00	1.00	1.55	0.06	0.65	0.00
Q92575	UBX domain-containing protein 4	UBXN4	57 kDa	1.00	1.00	1.85	0.04	0.54	0.00
P00533	Cluster of Epidermal growth factor receptor	EGFR	134 kDa	1.00	1.00	1.00	1.00	1.00	1.00
P07951	Cluster of Isoform 3 of Tropomyosin beta chain	TPM2	29 kDa	1.00	1.00	0.30	0.00	3.33	0.00
P49915	GMP synthase [glutamine-hydrolyzing]	GMPS	77 kDa	1.00	1.00	0.50	0.00	2.00	0.00
Q92859	Neogenin	NEO1	160 kDa	1.00	1.00	0.50	0.00	2.00	0.00
F5H737	Adenosylhomocysteinase	AHCY	45 kDa	1.00	1.00	0.50	0.00	2.00	0.00
P61289	Proteasome activator complex subunit 3	PSME3	30 kDa	1.00	1.00	0.60	0.00	1.67	0.00
O60664	Cluster of Perilipin-3	PLIN3	47 kDa	1.00	1.00	0.70	0.00	1.43	0.01
O96019	Cluster of Actin-like protein 6A	ACTL6A	47 kDa	1.00	1.00	0.80	0.00	1.25	0.02
H3BLU7	Cluster of Aflatoxin B1 aldehyde reductase member 2 (Fragi	AKR7A2	35 kDa	1.00	1.00	0.90	0.00	1.11	0.09
P54802	Alpha-N-acetylglucosaminidase	NAGLU	82 kDa	1.00	1.00	1.10	0.00	0.91	0.09

Q7Z739	Cluster of YTH domain family protein 3	YTHDF3	64 kDa	1.00	1.00	1.10	0.00	0.91	0.09
Q9ULV0	Cluster of Unconventional myosin-Vb	MYO5B	214 kDa	1.00	1.00	1.20	0.00	0.83	0.02
Q9HBD1	Cluster of RING finger and CCCH-type zinc finger domain-c	RC3H2	132 kDa	1.00	1.00	1.30	0.00	0.77	0.01
Q14692	Ribosome biogenesis protein BMS1 homolog	BMS1	146 kDa	1.00	1.00	1.40	0.00	0.71	0.00
Q9UDY4	DnaJ homolog subfamily B member 4	DNAJB4	38 kDa	1.00	1.00	1.70	0.00	0.59	0.00
E7EM64	Cluster of COP9 signalosome complex subunit 6	COPS6	36 kDa	1.00	1.00	0.70	0.00	1.43	0.01
Q07954	Prolow-density lipoprotein receptor-related protein 1	LRP1	505 kDa	1.00	1.00	1.50	0.00	0.67	0.00
E7ENU4	Double-stranded RNA-specific adenosine deaminase	ADAR	141 kDa	1.00	1.00	0.60	0.16	1.67	0.01
E9PGZ0	Cold shock domain-containing protein E1	CSDE1	91 kDa	1.00	1.00	0.90	0.50	1.11	0.31
P56537	Cluster of Eukaryotic translation initiation factor 6	EIF6	27 kDa	1.00	1.00	0.65	0.09	1.54	0.01
E9PL10	Transcription factor BTF3 homolog 4	BTF3L4	16 kDa	1.00	1.00	1.30	0.20	0.77	0.03
P36405	ADP-ribosylation factor-like protein 3	ARL3	20 kDa	1.00	1.00	1.40	0.16	0.71	0.01
Q9UNW1	Cluster of Multiple inositol polyphosphate phosphatase 1	MINPP1	55 kDa	1.00	1.00	0.95	0.50	1.05	0.51
P53007	Tricarboxylate transport protein, mitochondrial	SLC25A1	34 kDa	1.00	1.00	1.05	0.50	0.95	0.51
P53582	Methionine aminopeptidase 1	METAP1	43 kDa	1.00	1.00	1.05	0.50	0.95	0.51
Q15397	Pumilio domain-containing protein KIAA0020	KIAA0020	74 kDa	1.00	1.00	1.05	0.50	0.95	0.51
Q9H078	Cluster of Caseinolytic peptidase B protein homolog	CLPB	79 kDa	1.00	1.00	1.15	0.20	0.87	0.09
H3BPE7	RNA-binding protein FUS	FUS	53 kDa	1.00	1.00	0.70	0.00	1.43	0.01
Q9BT09	Protein canopy homolog 3	CNPY3	31 kDa	1.00	1.00	1.00	1.00	1.00	1.00
Q9NUQ3	Gamma-taxilin	TXLNG	61 kDa	1.00	1.00	1.00	1.00	1.00	1.00
Q9NY12	H/ACA ribonucleoprotein complex subunit 1	GAR1	22 kDa	1.00	1.00	1.00	1.00	1.00	1.00
B1B0M1	Cluster of GRIP1-associated protein 1	GRIPAP1	91 kDa	1.00	1.00	1.00	1.00	1.00	1.00
P05161	Ubiquitin-like protein ISG15	ISG15	18 kDa	1.18	0.04	0.90	0.50	1.31	0.04
Q9Y5H3	Cluster of Protocadherin gamma-A10	PCDHGA10	101 kDa	1.18	0.04	1.70	0.09	0.69	0.01
P48047	Cluster of ATP synthase subunit O, mitochondrial	ATP5O	23 kDa	1.18	0.04	0.80	0.00	1.47	0.00
P62491	Cluster of Ras-related protein Rab-11A	RAB11A	24 kDa	1.18	0.04	0.80	0.00	1.47	0.00
O14646	Cluster of Chromodomain-helicase-DNA-binding protein 1	CHD1	197 kDa	1.18	0.04	1.00	1.00	1.18	0.04
Q14139	Ubiquitin conjugation factor E4 A	UBE4A	123 kDa	1.18	0.04	0.65	0.26	1.81	0.01
Q9Y657	Spindlin-1	SPIN1	30 kDa	1.18	0.04	1.30	0.20	0.90	0.26
Q15714	TSC22 domain family protein 1	TSC22D1	110 kDa	1.18	0.04	2.15	0.08	0.55	0.00
Q96ST2	Protein IWS1 homolog	IWS1	92 kDa	1.18	0.04	1.15	0.20	1.02	0.76
C9JBI3	Phosphoserine phosphatase (Fragment)	PSPH	21 kDa	1.18	0.04	1.45	0.07	0.81	0.02
Q9NZN4	Cluster of EH domain-containing protein 2	EHD2	61 kDa	1.18	0.04	2.35	0.02	0.50	0.00
Q9P2K2	Thioredoxin domain-containing protein 16	TXNDC16	94 kDa	1.18	0.04	1.50	0.00	0.78	0.01
H3BRL3	Ubiquitin domain-containing protein UBFD1	UBFD1	33 kDa	1.18	0.04	0.80	0.00	1.47	0.00
P50895	Basal cell adhesion molecule	BCAM	67 kDa	1.18	0.04	0.90	0.50	1.31	0.04
Q9Y6W3	Calpain-7	CAPN7	93 kDa	1.18	0.04	1.80	0.08	0.65	0.00

P53367	Isoform A of Arfaptin-1	ARFIP1	39 kDa	1.18	0.04	0.95	0.50	1.24	0.04
Q12955	Ankyrin-3	ANK3	480 kDa	1.18	0.04	1.15	0.20	1.02	0.76
H0Y9D8	Inorganic pyrophosphatase 2, mitochondrial (Fragment)	PPA2	26 kDa	1.18	0.04	1.15	0.20	1.02	0.76
O95786	Probable ATP-dependent RNA helicase DDX58	DDX58	107 kDa	1.18	0.04	1.25	0.13	0.94	0.39
Q9BW85	Coiled-coil domain-containing protein 94	CCDC94	37 kDa	1.18	0.04	1.25	0.13	0.94	0.39
O60826	Coiled-coil domain-containing protein 22	CCDC22	71 kDa	1.18	0.04	1.35	0.09	0.87	0.09
Q5R3F8	Protein phosphatase 1 regulatory subunit 29	ELFN2	90 kDa	1.18	0.04	3.75	0.01	0.31	0.00
Q15233	Non-POU domain-containing octamer-binding protein	NONO	54 kDa	1.18	0.04	0.80	0.00	1.47	0.00
H0Y612	Cluster of E3 ubiquitin-protein ligase TRIM33 (Fragment)	TRIM33	99 kDa	1.18	0.04	1.10	0.00	1.07	0.22
E9PBL8	Conserved oligomeric Golgi complex subunit 1 (Fragment)	COG1	107 kDa	1.18	0.04	1.40	0.00	0.84	0.02
Q9H4A6	Golgi phosphoprotein 3	GOLPH3	34 kDa	1.18	0.04	1.40	0.00	0.84	0.02
P08240	Signal recognition particle receptor subunit alpha	SRPR	70 kDa	1.18	0.04	1.80	0.00	0.65	0.00
F8W930	Insulin-like growth factor 2 mRNA-binding protein 2	IGF2BP2	67 kDa	1.18	0.04	1.10	0.00	1.07	0.22
Q9UMS4	Cluster of Pre-mRNA-processing factor 19	PRPF19	55 kDa	0.63	0.00	0.75	0.13	0.83	0.06
Q9P0L0	Vesicle-associated membrane protein-associated protein A	VAPA	28 kDa	1.25	0.02	0.75	0.13	1.67	0.00
F5H025	Cluster of Neural cell adhesion molecule L1	L1CAM	140 kDa	0.63	0.00	0.95	0.50	0.66	0.00
A4D1W8	Ependymin related protein 1 (Zebrafish), isoform CRA_b	EPDR1	38 kDa	1.25	0.02	2.10	0.06	0.60	0.00
Q02790	Cluster of Peptidyl-prolyl cis-trans isomerase FKBP4	FKBP4	52 kDa	0.63	0.00	0.35	0.05	1.79	0.00
Q9BT78	COP9 signalosome complex subunit 4	COPS4	46 kDa	0.63	0.00	0.55	0.07	1.14	0.20
B4DTU4	Cluster of DNA ligase	LIG1	98 kDa	0.63	0.00	1.05	0.50	0.60	0.00
P07996	Cluster of Thrombospondin-1	THBS1	129 kDa	0.63	0.00	0.40	0.00	1.56	0.00
Q86VX2	Isoform 2 of COMM domain-containing protein 7	COMMD7	22 kDa	1.23	0.02	1.40	0.00	0.88	0.04
Q06546	GA-binding protein alpha chain	GABPA	51 kDa	1.23	0.02	1.35	0.09	0.91	0.18
P21964	Catechol O-methyltransferase	COMT	30 kDa	1.23	0.02	1.85	0.04	0.66	0.00
Q15057	Cluster of Arf-GAP with coiled-coil, ANK repeat and PH do	ACAP2	88 kDa	1.23	0.02	0.70	0.20	1.75	0.01
B4DME2	Protein phosphatase 1 regulatory subunit 12C	PPP1R12C	77 kDa	1.23	0.02	2.30	0.10	0.53	0.00
Q14114	Cluster of Low-density lipoprotein receptor-related protein 8	LRP8	106 kDa	1.23	0.02	1.20	0.30	1.02	0.80
H3BSH7	Cirhin (Fragment)	CIRH1A	78 kDa	1.23	0.02	1.35	0.09	0.91	0.18
O60921	Cluster of Checkpoint protein HUS1	HUS1	32 kDa	1.23	0.02	2.65	0.02	0.46	0.00
P08123	Collagen alpha-2(I) chain	COL1A2	129 kDa	1.23	0.02	2.95	0.02	0.42	0.00
Q9Y5X3	Sorting nexin-5	SNX5	47 kDa	1.23	0.02	0.90	0.00	1.36	0.01
Q00341	Cluster of Vigilin	HDLBP	141 kDa	0.75	0.00	0.85	0.50	0.88	0.38
P46087	Putative ribosomal RNA methyltransferase NOP2	NOP2	89 kDa	0.75	0.00	0.70	0.20	1.07	0.54
P30049	ATP synthase subunit delta, mitochondrial	ATP5D	17 kDa	0.75	0.00	1.05	0.80	0.71	0.04
P55060	Exportin-2	CSE1L	110 kDa	0.75	0.00	0.45	0.06	1.67	0.00
Q9BYT8	Neurolysin, mitochondrial	NLN	81 kDa	1.50	0.00	1.00	1.00	1.50	0.01
P29966	Myristoylated alanine-rich C-kinase substrate	MARCKS	32 kDa	0.75	0.00	1.00	1.00	0.75	0.03

Q68CR9	Cluster of Aspartate--tRNA ligase, cytoplasmic	DKFZp781B11	46 kDa	0.75	0.00	0.55	0.07	1.36	0.02
Q96GD0	Pyridoxal phosphate phosphatase	PDXP	32 kDa	0.75	0.00	1.20	0.30	0.63	0.00
O60264	SWI/SNF-related matrix-associated actin-dependent regulatc	SMARCA5	122 kDa	0.75	0.00	0.75	0.13	1.00	1.00
P13861	Cluster of cAMP-dependent protein kinase type II-alpha regu	PRKAR2A	46 kDa	0.75	0.00	0.85	0.20	0.88	0.13
P21281	Cluster of V-type proton ATPase subunit B, brain isoform	ATP6V1B2	57 kDa	0.75	0.00	0.85	0.20	0.88	0.13
Q96SI9	Spermatid perinuclear RNA-binding protein	STRBP	74 kDa	0.75	0.00	0.85	0.20	0.88	0.13
Q9H5X1	MIP18 family protein FAM96A	FAM96A	18 kDa	0.75	0.00	2.00	0.06	0.38	0.00
P83731	60S ribosomal protein L24	RPL24	18 kDa	0.75	0.00	1.05	0.50	0.71	0.00
Q9H497	Cluster of Torsin-3A	TOR3A	46 kDa	0.75	0.00	1.15	0.20	0.65	0.00
Q9H307	Pinin	PNN	82 kDa	0.75	0.00	1.15	0.20	0.65	0.00
Q14746	Cluster of Conserved oligomeric Golgi complex subunit 2	COG2	83 kDa	0.75	0.00	1.25	0.13	0.60	0.00
Q9HD47	Cluster of Ran guanine nucleotide release factor	RANGRF	20 kDa	0.75	0.00	1.65	0.05	0.45	0.00
Q9Y5Y6	Suppressor of tumorigenicity 14 protein	ST14	95 kDa	0.75	0.00	0.80	0.00	0.94	0.18
B4DP17	Cluster of Cellular nucleic acid-binding protein	CNBP	18 kDa	0.75	0.00	0.80	0.00	0.94	0.18
F8W1H5	Ras-related protein Rab-5C	RAB5C	27 kDa	0.75	0.00	0.80	0.00	0.94	0.18
Q9BUH6	Uncharacterized protein C9orf142	C9orf142	22 kDa	0.75	0.00	0.90	0.00	0.83	0.01
Q96I15	Cluster of Selenocysteine lyase	SCLY	48 kDa	0.75	0.00	1.10	0.00	0.68	0.00
P46937	Yorkie homolog	YAP1	54 kDa	0.75	0.00	1.50	0.00	0.50	0.00
Q9ULH1	Arf-GAP with SH3 domain, ANK repeat and PH domain-cor	ASAP1	126 kDa	0.75	0.00	1.60	0.00	0.47	0.00
Q14CX7	Cluster of N-alpha-acetyltransferase 25, NatB auxiliary subu	NAA25	112 kDa	0.75	0.00	0.50	0.00	1.50	0.00
Q9BWD1	Cluster of Acetyl-CoA acetyltransferase, cytosolic	ACAT2	41 kDa	0.75	0.00	0.50	0.00	1.50	0.00
Q8NBJ4	Cluster of Golgi membrane protein 1	GOLM1	45 kDa	0.75	0.00	0.70	0.00	1.07	0.18
Q9Y263	Cluster of Phospholipase A-2-activating protein	PLAA	87 kDa	0.75	0.00	0.70	0.00	1.07	0.18
Q92888	Cluster of Rho guanine nucleotide exchange factor 1	ARHGEF1	102 kDa	0.75	0.00	0.80	0.00	0.94	0.18
Q01650	Large neutral amino acids transporter small subunit 1	SLC7A5	55 kDa	1.50	0.00	0.90	0.00	1.67	0.00
Q13443	Disintegrin and metalloproteinase domain-containing proteir	ADAM9	91 kDa	0.75	0.00	0.90	0.00	0.83	0.01
Q15643	Cluster of Thyroid receptor-interacting protein 11	TRIP11	228 kDa	0.75	0.00	0.90	0.00	0.83	0.01
Q9UJW0	Cluster of Dynactin subunit 4	DCTN4	52 kDa	0.75	0.00	0.90	0.00	0.83	0.01
Q9UPT5	Exocyst complex component 7	EXOC7	83 kDa	0.75	0.00	0.90	0.00	0.83	0.01
Q969Q0	Cluster of 60S ribosomal protein L36a-like	RPL36AL	12 kDa	1.50	0.00	1.00	1.00	1.50	0.00
Q99805	Transmembrane 9 superfamily member 2	TM9SF2	76 kDa	0.75	0.00	1.00	1.00	0.75	0.00
Q96AY3	Cluster of Peptidyl-prolyl cis-trans isomerase FKBP10	FKBP10	64 kDa	0.75	0.00	1.10	0.00	0.68	0.00
G3V141	Collagen triple helix repeat containing 1, isoform CRA_c	CTHRC1	25 kDa	0.75	0.00	1.20	0.00	0.63	0.00
Q6P3X3	Tetratricopeptide repeat protein 27	TTC27	97 kDa	0.75	0.00	1.30	0.00	0.58	0.00
Q9Y2S7	Polymerase delta-interacting protein 2	POLDIP2	42 kDa	0.75	0.00	1.90	0.00	0.39	0.00
H0YDW2	Cluster of Liprin-alpha-1 (Fragment)	PPFIA1	42 kDa	1.28	0.01	0.85	0.20	1.50	0.01
O75683	Surfeit locus protein 6	SURF6	41 kDa	1.28	0.01	1.65	0.05	0.77	0.01

H3BTI0	Cysteine-rich secretory protein LCCL domain-containing 2	CRISPLD2	56 kDa	1.28	0.01	1.85	0.04	0.69	0.00
B2RAK2	MHC class I polypeptide-related sequence B	MICB	43 kDa	1.28	0.01	1.95	0.03	0.65	0.00
Q99816	Tumor susceptibility gene 101 protein	TSG101	44 kDa	1.28	0.01	1.00	1.00	1.28	0.01
P40926	Malate dehydrogenase, mitochondrial	MDH2	36 kDa	1.28	0.01	0.60	0.16	2.13	0.00
Q92889	DNA repair endonuclease XPF	ERCC4	104 kDa	1.28	0.01	2.45	0.02	0.52	0.00
F5H5N1	Cluster of NADH dehydrogenase [ubiquinone] iron-sulfur pr	NDUFS7	20 kDa	1.28	0.01	1.80	0.08	0.71	0.01
Q8NFH4	Nucleoporin Nup37	NUP37	37 kDa	1.28	0.01	0.95	0.50	1.34	0.01
Q9BSG5	Isoform 2 of Retbindin	RTBDN	28 kDa	1.90	0.00	1.35	0.09	1.41	0.01
P50453	Serpin B9	SERPINB9	42 kDa	1.10	0.09	0.85	0.50	1.29	0.09
B4DR61	Cluster of Protein transport protein Sec61 subunit alpha isof	SEC61A1	53 kDa	1.10	0.09	1.00	1.00	1.10	0.31
P31431	Cluster of Syndecan-4	SDC4	22 kDa	1.10	0.09	0.55	0.07	2.00	0.00
Q8NEJ9	Cluster of Neuroguidin	NGDN	36 kDa	1.10	0.09	1.75	0.13	0.63	0.00
P07737	Profilin-1	PFN1	15 kDa	1.10	0.09	0.65	0.09	1.69	0.00
Q96BP3	Peptidylprolyl isomerase domain and WD repeat-containing	PPWD1	74 kDa	1.10	0.09	0.65	0.09	1.69	0.00
Q04917	Cluster of 14-3-3 protein eta	YWHAH	28 kDa	1.10	0.09	0.75	0.13	1.47	0.01
O75462	Cytokine receptor-like factor 1	CRLF1	46 kDa	1.10	0.09	0.75	0.13	1.47	0.01
Q86TU7	Cluster of Histone-lysine N-methyltransferase setd3	SETD3	67 kDa	1.10	0.09	0.85	0.20	1.29	0.02
P49840	Cluster of Glycogen synthase kinase-3 alpha	GSK3A	51 kDa	1.10	0.09	0.85	0.20	1.29	0.02
Q9NVU7	Protein SDA1 homolog	SDAD1	80 kDa	1.10	0.09	1.80	0.08	0.61	0.00
Q6WCQ1	Cluster of Isoform 2 of Myosin phosphatase Rho-interacting	MPRIP	118 kDa	1.10	0.09	1.05	0.50	1.05	0.51
I3L1G1	Cluster of TOM1-like protein 1 (Fragment)	TOM1L1	28 kDa	1.10	0.09	1.05	0.50	1.05	0.51
E5RHG8	Transcription elongation factor B polypeptide 1 (Fragment)	TCEB1	10 kDa	1.10	0.09	1.15	0.20	0.96	0.51
H7BYW6	Platelet-derived growth factor subunit A (Fragment)	PDGFA	23 kDa	1.10	0.09	1.35	0.09	0.81	0.02
Q6I9Y2	THO complex subunit 7 homolog	THOC7	24 kDa	1.10	0.09	1.55	0.06	0.71	0.00
P61218	Cluster of DNA-directed RNA polymerases I, II, and III subu	POLR2F	14 kDa	1.10	0.09	1.75	0.04	0.63	0.00
Q99614	Tetratricopeptide repeat protein 1	TTC1	34 kDa	1.10	0.09	0.90	0.00	1.22	0.02
Q9H2J4	Phosducin-like protein 3	PDCL3	28 kDa	1.10	0.09	0.90	0.00	1.22	0.02
P50452	Cluster of Serpin B8	SERPINB8	43 kDa	1.10	0.09	1.10	0.00	1.00	1.00
Q68BL7	Cluster of Olfactomedin-like protein 2A	OLFML2A	73 kDa	1.10	0.09	1.30	0.00	0.85	0.02
Q00610	Cluster of Clathrin heavy chain 1	CLTC	192 kDa	1.10	0.09	5.80	0.00	0.19	0.00
D6RER5	Cluster of Septin-11	SEPT11	50 kDa	1.10	0.09	0.50	0.00	2.20	0.00
P36551	Coproporphyrinogen-III oxidase, mitochondrial	CPOX	50 kDa	1.10	0.09	0.60	0.00	1.83	0.00
O15145	Actin-related protein 2/3 complex subunit 3	ARPC3	21 kDa	1.10	0.09	0.90	0.00	1.22	0.02
P24534	Elongation factor 1-beta	EEF1B2	25 kDa	1.10	0.09	0.35	0.05	3.14	0.00
Q9NVE7	Pantothenate kinase 4	PANK4	86 kDa	1.10	0.09	1.75	0.13	0.63	0.00
H3BRM0	Cluster of Phosphomannomutase 2	PMM2	18 kDa	1.10	0.09	0.75	0.13	1.47	0.01
F5H7V7	NADH dehydrogenase [ubiquinone] 1 alpha subcomplex sub	NDUFA8	15 kDa	1.10	0.09	1.65	0.05	0.67	0.00

Q96TC7	Cluster of Regulator of microtubule dynamics protein 3	RMDN3	52 kDa	1.10	0.09	2.05	0.03	0.54	0.00
B1AH89	Tubulin tyrosine ligase-like family, member 12	TTLL12	74 kDa	1.10	0.09	0.70	0.00	1.57	0.00
P00441	Cluster of Superoxide dismutase [Cu-Zn]	SOD1	16 kDa	1.10	0.09	0.80	0.00	1.38	0.01
Q9BUT1	Cluster of 3-hydroxybutyrate dehydrogenase type 2	BDH2	27 kDa	1.10	0.09	2.10	0.00	0.52	0.00
Q96I99	Isoform 2 of Succinyl-CoA ligase [GDP-forming] subunit be	SUCLG2	48 kDa	1.35	0.01	1.75	0.04	0.77	0.01
P01008	Antithrombin-III	SERPINC1	53 kDa	1.35	0.01	0.70	0.00	1.93	0.00
B4DVE7	Cluster of Annexin	ANXA11	51 kDa	1.35	0.01	0.60	0.00	2.25	0.00
A3KN83	Protein strawberry notch homolog 1	SBNO1	154 kDa	0.68	0.00	1.90	0.14	0.36	0.10
H0Y6E7	Cluster of RNA-binding motif protein, X chromosome (Frag	RBMX	32 kDa	0.68	0.00	1.30	0.20	0.52	0.00
Q96FJ2	Cluster of Dynein light chain 2, cytoplasmic	DYNLL2	10 kDa	1.35	0.01	0.95	0.50	1.42	0.01
Q08722	Isoform OA3-312 of Leukocyte surface antigen CD47	CD47	34 kDa	0.68	0.00	1.35	0.09	0.50	0.00
Q9H7P6	Cluster of Multivesicular body subunit 12B	MVB12B	36 kDa	0.68	0.00	1.65	0.05	0.41	0.00
O60568	Procollagen-lysine,2-oxoglutarate 5-dioxygenase 3	PLOD3	85 kDa	0.68	0.00	0.30	0.00	2.25	0.00
E9PCD7	Epididymis-specific alpha-mannosidase	MAN2B2	108 kDa	0.68	0.00	1.00	1.00	0.68	0.00
Q9H081	Protein MIS12 homolog	MIS12	24 kDa	0.68	0.00	1.60	0.00	0.42	0.00
P09429	Cluster of High mobility group protein B1	HMGB1	25 kDa	0.68	0.00	0.25	0.04	2.70	0.00
G3V3F1	Neural cell expressed, developmentally down-regulated 1, is	NEDD1	73 kDa	1.35	0.01	1.40	0.16	0.96	0.63
Q5TCM7	Protein tyrosine phosphatase type IVA 1	PTP4A1	17 kDa	1.35	0.01	1.15	0.20	1.17	0.07
P07357	Complement component C8 alpha chain	C8A	65 kDa	0.68	0.00	1.40	0.00	0.48	0.00
P12830	Cluster of Cadherin-1	CDH1	97 kDa	1.33	0.01	0.65	0.09	2.04	0.00
P51946	Cluster of Cyclin-H	CCNH	38 kDa	1.33	0.01	2.10	0.11	0.63	0.01
Q9UIV1	CCR4-NOT transcription complex subunit 7	CNOT7	33 kDa	1.33	0.01	3.00	0.06	0.44	0.00
Q8IYB7	Cluster of DIS3-like exonuclease 2	DIS3L2	99 kDa	1.33	0.01	0.85	0.20	1.56	0.00
Q9Y6D9	Cluster of Mitotic spindle assembly checkpoint protein MAI	MAD1L1	83 kDa	1.33	0.01	1.15	0.20	1.15	0.09
F8VSZ4	Cluster of Plexin-A1	PLXNA1	209 kDa	1.33	0.01	1.25	0.13	1.06	0.39
Q9H008	Phospholysine phosphohistidine inorganic pyrophosphate ph	LHPP	29 kDa	1.33	0.01	1.35	0.09	0.98	0.76
Q96M27	Cluster of Protein PRRC1	PRRC1	47 kDa	1.33	0.01	1.10	0.00	1.20	0.02
Q8IWB7	WD repeat and FYVE domain-containing protein 1	WDFY1	46 kDa	1.33	0.01	1.90	0.00	0.70	0.00
Q9H939	Proline-serine-threonine phosphatase-interacting protein 2	PSTPIP2	39 kDa	1.60	0.00	1.05	0.50	1.52	0.00
P41271	Isoform 2 of Neuroblastoma suppressor of tumorigenicity 1	NBL1	23 kDa	1.60	0.00	1.35	0.09	1.19	0.05
E7EPK6	40S ribosomal protein S24	RPS24	32 kDa	1.60	0.00	1.10	0.00	1.45	0.00
B4DTG2	Cluster of Elongation factor 1-gamma	EEF1G	56 kDa	2.95	0.00	0.35	0.05	8.43	0.00
B3KW71	Cluster of Dihydropteridine reductase	QDPR	22 kDa	1.38	0.00	1.00	1.00	1.38	0.02
O75493	Carbonic anhydrase-related protein 11	CA11	36 kDa	1.38	0.00	1.95	0.16	0.71	0.03
P50238	Cysteine-rich protein 1	CRIP1	9 kDa	1.38	0.00	1.25	0.13	1.10	0.18
Q92854	Cluster of Semaphorin-4D	SEMA4D	96 kDa	1.38	0.00	1.10	0.00	1.25	0.01
D6W5A2	Metastasis associated 1 family, member 3, isoform CRA_a	MTA3	61 kDa	1.38	0.00	1.70	0.00	0.81	0.01

Q86Y82	Syntaxin-12	STX12	32 kDa	0.73	0.00	1.10	0.50	0.66	0.01
Q8NC96	Cluster of Adaptin ear-binding coat-associated protein 1	NECAP1	30 kDa	0.73	0.00	1.30	0.20	0.56	0.00
P21291	Cluster of Cysteine and glycine-rich protein 1	CSRP1	21 kDa	0.73	0.00	0.75	0.13	0.97	0.63
Q96SU4	Isoform 7 of Oxysterol-binding protein-related protein 9	OSBPL9	81 kDa	0.73	0.00	2.45	0.07	0.30	0.05
P62191	26S protease regulatory subunit 4	PSMC1	49 kDa	0.73	0.00	0.85	0.20	0.85	0.06
Q13643	Four and a half LIM domains protein 3	FHL3	31 kDa	0.73	0.00	1.15	0.20	0.63	0.00
Q9UBG0	C-type mannose receptor 2	MRC2	167 kDa	0.73	0.00	1.35	0.09	0.54	0.00
P43686	26S protease regulatory subunit 6B	PSMC4	47 kDa	0.73	0.00	0.70	0.00	1.04	0.39
O94874	E3 UFM1-protein ligase 1	UFL1	90 kDa	0.73	0.00	0.80	0.00	0.91	0.06
Q53FA7	Quinone oxidoreductase PIG3	TP53I3	36 kDa	0.73	0.00	0.90	0.00	0.81	0.01
Q92817	Envoplakin	EVPL	232 kDa	0.73	0.00	0.90	0.00	0.81	0.01
P08107	Cluster of Heat shock 70 kDa protein 1A/1B	HSPA1A	70 kDa	0.73	0.00	0.25	0.04	2.90	0.00
P30040	Cluster of Endoplasmic reticulum resident protein 29	ERP29	29 kDa	0.73	0.00	1.00	1.00	0.73	0.40
P22314	Cluster of Ubiquitin-like modifier-activating enzyme 1	UBA1	118 kDa	0.73	0.00	0.35	0.05	2.07	0.00
P37837	Transaldolase	TALDO1	38 kDa	0.73	0.00	0.45	0.06	1.61	0.00
P43034	Cluster of Platelet-activating factor acetylhydrolase IB subur	PAFAH1B1	47 kDa	0.73	0.00	0.55	0.07	1.32	0.02
P27708	Cluster of CAD protein	CAD	243 kDa	0.73	0.00	0.65	0.09	1.12	0.20
Q05086	Ubiquitin-protein ligase E3A	UBE3A	101 kDa	0.73	0.00	0.75	0.13	0.97	0.63
P62280	40S ribosomal protein S11	RPS11	18 kDa	0.73	0.00	0.85	0.20	0.85	0.06
Q9NZZ3	Charged multivesicular body protein 5	CHMP5	25 kDa	0.73	0.00	0.85	0.20	0.85	0.06
Q14651	Plastin-1	PLS1	70 kDa	0.73	0.00	0.85	0.20	0.85	0.06
Q6P988	Protein notum homolog	NOTUM	56 kDa	0.73	0.00	1.15	0.20	0.63	0.00
Q9Y6A5	Transforming acidic coiled-coil-containing protein 3	TACC3	90 kDa	0.73	0.00	1.15	0.20	0.63	0.00
E7EPA7	Transketolase	TKT	69 kDa	0.73	0.00	0.20	0.00	3.63	0.00
D6RAK8	Cluster of Vitamin D-binding protein	GC	55 kDa	0.73	0.00	0.60	0.00	1.21	0.02
P07225	Cluster of Vitamin K-dependent protein S	PROS1	75 kDa	0.73	0.00	0.80	0.00	0.91	0.06
B4DP11	Prostaglandin E synthase 3	PTGES3	16 kDa	0.73	0.00	1.00	1.00	0.73	0.00
Q96SZ5	2-aminoethanethiol dioxygenase	ADO	30 kDa	1.83	0.00	1.15	0.20	1.59	0.00
Q5VIR6	Cluster of Isoform 4 of Vacuolar protein sorting-associated p	VPS53	94 kDa	1.20	0.02	2.20	0.20	0.55	0.24
O95352	Cluster of Ubiquitin-like modifier-activating enzyme ATG7	ATG7	78 kDa	1.20	0.02	0.85	0.20	1.41	0.01
O60476	Mannosyl-oligosaccharide 1,2-alpha-mannosidase IB	MAN1A2	73 kDa	1.20	0.02	0.95	0.50	1.26	0.02
P24347	Cluster of Stromelysin-3	MMP11	55 kDa	1.20	0.02	1.05	0.50	1.14	0.09
J3KRC4	Cluster of 5'(3')-deoxyribonucleotidase, cytosolic type	NT5C	20 kDa	1.20	0.02	1.35	0.09	0.89	0.09
P01024	Complement C3	C3	187 kDa	1.20	0.02	0.40	0.00	3.00	0.00
Q9Y2Q5	Ragulator complex protein LAMTOR2	LAMTOR2	14 kDa	1.14	0.09	2.19	0.01	0.52	0.00
P61201	COP9 signalosome complex subunit 2	COPS2	52 kDa	1.20	0.02	0.70	0.00	1.71	0.00
O75843	Cluster of AP-1 complex subunit gamma-like 2	AP1G2	87 kDa	1.20	0.02	2.10	0.00	0.57	0.00

O14732	Inositol monophosphatase 2	IMPA2	31 kDa	1.20	0.02	3.00	0.00	0.40	0.00
A6NFM0	Cluster of DNA-directed RNA polymerase II subunit RPB11	POLR2J2	17 kDa	1.20	0.02	2.10	0.06	0.57	0.00
Q12841	Cluster of Follistatin-related protein 1	FSTL1	35 kDa	1.20	0.02	1.10	0.50	1.09	0.31
C9JRZ6	Coiled-coil-helix-coiled-coil-helix domain-containing protei	CHCHD3	27 kDa	1.20	0.02	1.40	0.16	0.86	0.08
P21912	Succinate dehydrogenase [ubiquinone] iron-sulfur subunit, m	SDHB	32 kDa	1.20	0.02	1.05	0.50	1.14	0.09
O00401	Neural Wiskott-Aldrich syndrome protein	WASL	55 kDa	1.20	0.02	1.35	0.09	0.89	0.09
B7Z3D5	Cluster of Myotubularin related protein 1, isoform CRA_a	MTMR1	65 kDa	1.20	0.02	1.65	0.05	0.73	0.00
E9PK59	N-terminal kinase-like protein	SCYL1	86 kDa	1.20	0.02	1.30	0.00	0.92	0.09
O14974	Cluster of Isoform 4 of Protein phosphatase 1 regulatory sub	PPP1R12A	109 kDa	1.20	0.02	0.90	0.50	1.33	0.03
G8JLD3	Cluster of ELKS/Rab6-interacting/CAST family member 1	ERC1	125 kDa	0.85	0.01	0.60	0.16	1.42	0.03
P25788	Proteasome subunit alpha type-3	PSMA3	28 kDa	0.85	0.01	0.45	0.06	1.89	0.00
O43405	Cluster of Cochlin	COCH	59 kDa	0.85	0.01	0.90	0.50	0.94	0.54
O95633	Follistatin-related protein 3	FSTL3	28 kDa	0.85	0.01	1.00	1.00	0.85	0.12
P08621	U1 small nuclear ribonucleoprotein 70 kDa	SNRNP70	52 kDa	0.85	0.01	0.55	0.07	1.55	0.00
O75531	Barrier-to-autointegration factor	BANF1	10 kDa	0.85	0.01	0.75	0.13	1.13	0.13
Q13510	Isoform 2 of Acid ceramidase	ASAH1	47 kDa	0.85	0.01	0.75	0.13	1.13	0.13
P12955	Xaa-Pro dipeptidase	PEPD	55 kDa	0.85	0.01	0.85	0.20	1.00	1.00
P14550	Alcohol dehydrogenase [NADP(+)]	AKR1A1	37 kDa	0.85	0.01	0.85	0.20	1.00	1.00
P61962	DDB1- and CUL4-associated factor 7	DCAF7	39 kDa	0.85	0.01	1.05	0.50	0.81	0.02
P11171	Protein 4.1	EPB41	97 kDa	0.85	0.01	1.15	0.20	0.74	0.00
Q5T2T1	MAGUK p55 subfamily member 7	MPP7	66 kDa	0.85	0.01	1.15	0.20	0.74	0.00
Q12769	Cluster of Nuclear pore complex protein Nup160	NUP160	162 kDa	0.85	0.01	1.55	0.06	0.55	0.00
Q92974	Rho guanine nucleotide exchange factor 2	ARHGEF2	112 kDa	0.85	0.01	1.55	0.06	0.55	0.00
P29372	Isoform 2 of DNA-3-methyladenine glycosylase	MPG	32 kDa	0.85	0.01	1.65	0.05	0.52	0.00
G3V4K3	Spermatogenesis-defective protein 39 homolog	VIPAS39	60 kDa	0.85	0.01	1.95	0.03	0.44	0.00
J3QRS3	Cluster of Myosin regulatory light chain 12A	MYL12A	20 kDa	0.85	0.01	0.60	0.00	1.42	0.00
P12259	Coagulation factor V	F5	252 kDa	0.85	0.01	0.70	0.00	1.21	0.01
Q15007	Pre-mRNA-splicing regulator WTAP	WTAP	44 kDa	0.85	0.01	0.80	0.00	1.06	0.18
Q8WWY3	Cluster of U4/U6 small nuclear ribonucleoprotein Prp31	PRPF31	55 kDa	0.85	0.01	1.00	1.00	0.85	0.01
P19387	DNA-directed RNA polymerase II subunit RPB3	POLR2C	31 kDa	0.85	0.01	3.40	0.00	0.25	0.00
Q01813	Cluster of 6-phosphofructokinase type C	PFKP	86 kDa	0.85	0.01	0.50	0.00	1.70	0.00
Q01970	1-phosphatidylinositol 4,5-bisphosphate phosphodiesterase b	PLCB3	139 kDa	0.85	0.01	0.50	0.00	1.70	0.00
P61326	Cluster of Protein mago nashi homolog	MAGOH	17 kDa	0.85	0.01	0.80	0.00	1.06	0.18
Q02241	Cluster of Kinesin-like protein KIF23	KIF23	110 kDa	0.85	0.01	0.80	0.00	1.06	0.18
P53609	Geranylgeranyl transferase type-1 subunit beta	PGGT1B	42 kDa	0.85	0.01	1.00	1.00	0.85	0.01
Q15257	Cluster of Serine/threonine-protein phosphatase 2A activator	PPP2R4	41 kDa	0.85	0.01	1.00	1.00	0.85	0.01
Q16763	Ubiquitin-conjugating enzyme E2 S	UBE2S	24 kDa	0.85	0.01	1.00	1.00	0.85	0.01

Q5RKV6	Exosome complex component MTR3	EXOSC6	28 kDa	0.85	0.01	1.00	1.00	0.85	0.01
O43795	Unconventional myosin-Ib	MYO1B	132 kDa	0.85	0.01	1.10	0.00	0.77	0.00
Q9H6Y2	WD repeat-containing protein 55	WDR55	42 kDa	0.85	0.01	1.20	0.00	0.71	0.00
Q9NRF8	CTP synthase 2	CTPS2	66 kDa	0.85	0.01	1.20	0.00	0.71	0.00
Q9UBL6	Copine-7	CPNE7	70 kDa	0.85	0.01	1.20	0.00	0.71	0.00
P50225	Cluster of Sulfotransferase 1A1	SULT1A1	34 kDa	0.85	0.01	1.50	0.00	0.57	0.00
O95104	Splicing factor, arginine/serine-rich 15	SCAF4	126 kDa	0.85	0.01	2.10	0.00	0.40	0.00
P02786	Cluster of Transferrin receptor protein 1	TFRC	85 kDa	0.85	0.01	0.50	0.13	1.70	0.01
Q09666	Cluster of Neuroblast differentiation-associated protein AHN	AHNAK	629 kDa	0.85	0.01	0.50	0.13	1.70	0.01
P61158	Cluster of Actin-related protein 3	ACTR3	47 kDa	0.85	0.01	0.55	0.07	1.55	0.00
P61011	Signal recognition particle 54 kDa protein	SRP54	56 kDa	0.85	0.01	0.65	0.09	1.31	0.02
E9PCI9	Farnesyl pyrophosphate synthase	FDPS	41 kDa	0.85	0.01	0.75	0.13	1.13	0.13
E9PJF4	Cluster of Methylosome subunit pICln	CLNS1A	20 kDa	0.85	0.01	0.85	0.20	1.00	1.00
Q9UBL3	Cluster of Set1/Ash2 histone methyltransferase complex sub	ASH2L	69 kDa	0.85	0.01	0.95	0.50	0.89	0.13
Q9UBQ7	Cluster of Glyoxylate reductase/hydroxypyruvate reductase	GRHPR	36 kDa	0.85	0.01	0.95	0.50	0.89	0.13
Q9UDT6	Cluster of CAP-Gly domain-containing linker protein 2	CLIP2	116 kDa	0.85	0.01	0.95	0.50	0.89	0.13
P36969	Phospholipid hydroperoxide glutathione peroxidase, mitocho	GPX4	22 kDa	0.85	0.01	1.05	0.50	0.81	0.02
H0Y400	Cluster of Spliceosome RNA helicase DDX39B (Fragment)	DDX39B	22 kDa	0.85	0.01	1.05	0.50	0.81	0.02
E9PNU4	Cluster of Syntaxin-5	STX5	28 kDa	0.85	0.01	1.05	0.50	0.81	0.02
Q06330	Isoform 7 of Recombining binding protein suppressor of hai	RBPJ	54 kDa	0.85	0.01	1.15	0.20	0.74	0.00
G3XAN8	Mitochondrial import inner membrane translocase subunit T	TIMM8B	11 kDa	0.85	0.01	1.25	0.13	0.68	0.00
Q14160	Cluster of Isoform 3 of Protein scribble homolog	SCRIB	178 kDa	0.85	0.01	1.25	0.13	0.68	0.00
P10301	Ras-related protein R-Ras	RRAS	23 kDa	0.85	0.01	1.35	0.09	0.63	0.00
Q14264	HERV-R_7q21.2 provirus ancestral Env polyprotein	ERV3-1	68 kDa	0.85	0.01	1.35	0.09	0.63	0.00
E5RFU2	Bifunctional epoxide hydrolase 2	EPHX2	59 kDa	0.85	0.01	1.35	0.09	0.63	0.00
O94813	Slit homolog 2 protein	SLIT2	170 kDa	0.85	0.01	1.75	0.04	0.49	0.00
Q9UBT2	SUMO-activating enzyme subunit 2	UBA2	71 kDa	0.85	0.01	0.50	0.00	1.70	0.00
P09525	Cluster of Annexin A4	ANXA4	36 kDa	0.85	0.01	0.60	0.00	1.42	0.00
Q99497	Protein DJ-1	PARK7	20 kDa	0.85	0.01	0.70	0.00	1.21	0.01
P47813	Cluster of Eukaryotic translation initiation factor 1A, X-chro	EIF1AX	16 kDa	0.85	0.01	0.80	0.00	1.06	0.18
O15344	Midline-1	MID1	75 kDa	0.85	0.01	1.30	0.00	0.65	0.00
Q6PD62	RNA polymerase-associated protein CTR9 homolog	CTR9	134 kDa	0.89	0.13	0.84	0.20	1.06	0.18
Q6IN85	Cluster of Serine/threonine-protein phosphatase 4 regulatory	SMEK1	95 kDa	0.85	0.01	0.80	0.00	1.06	0.18
P29373	Cellular retinoic acid-binding protein 2	CRABP2	16 kDa	0.85	0.01	0.90	0.00	0.94	0.18
O00566	U3 small nucleolar ribonucleoprotein protein MPP10	MPHOSPH10	79 kDa	0.85	0.01	1.00	1.00	0.85	0.01
Q7Z6M1	Cluster of Rab9 effector protein with kelch motifs	RABEPK	41 kDa	0.85	0.01	1.10	0.00	0.77	0.00
Q10713	Cluster of Mitochondrial-processing peptidase subunit alpha	PMPCA	58 kDa	0.85	0.01	1.20	0.00	0.71	0.00

Q9H1B7	Cluster of Interferon regulatory factor 2-binding protein-like	IRF2BPL	83 kDa	1.43	0.00	1.25	0.13	1.14	0.09
P40763	Signal transducer and activator of transcription 3	STAT3	88 kDa	1.43	0.00	0.95	0.50	1.50	0.00
E1P660	Uncharacterized protein	ZNF207	53 kDa	1.43	0.00	1.05	0.50	1.36	0.01
Q9Y2V7	Cluster of Conserved oligomeric Golgi complex subunit 6	COG6	73 kDa	1.43	0.00	1.05	0.50	1.36	0.01
Q8N9R8	Cluster of Protein SCAI	SCAI	70 kDa	1.43	0.00	1.45	0.07	0.98	0.76
Q9NVJ2	Cluster of ADP-ribosylation factor-like protein 8B	ARL8B	22 kDa	1.43	0.00	1.10	0.00	1.30	0.01
Q13907	Cluster of Isopentenyl-diphosphate Delta-isomerase 1	IDI1	26 kDa	1.43	0.00	1.20	0.00	1.19	0.02
Q9BY76	Angiopoietin-related protein 4	ANGPTL4	45 kDa	1.48	0.00	1.10	0.50	1.34	0.02
Q9NQY0	Bridging integrator 3	BIN3	30 kDa	1.48	0.00	1.30	0.20	1.13	0.14
Q15031	Probable leucine--tRNA ligase, mitochondrial	LARS2	102 kDa	1.48	0.00	3.25	0.04	0.45	0.00
A6NE09	Protein RPSAP58	RPSAP58	33 kDa	1.48	0.00	6.95	0.03	0.21	0.00
Q14997	Proteasome activator complex subunit 4	PSME4	211 kDa	1.48	0.00	0.90	0.00	1.64	0.00
P04632	Cluster of Calpain small subunit 1	CAPNS1	28 kDa	0.78	0.00	0.50	0.13	1.55	0.02
Q5TA95	Tetratricopeptide repeat domain 4	TTC4	46 kDa	0.78	0.00	1.10	0.70	0.70	0.35
Q32P28	Cluster of Prolyl 3-hydroxylase 1	LEPRE1	83 kDa	0.78	0.00	0.90	0.50	0.86	0.16
P09661	U2 small nuclear ribonucleoprotein A'	SNRPA1	28 kDa	0.78	0.00	0.90	0.50	0.86	0.16
Q16512	Cluster of Serine/threonine-protein kinase N1	PKN1	104 kDa	0.78	0.00	0.90	0.50	0.86	0.16
Q8N392	Rho GTPase-activating protein 18	ARHGAP18	75 kDa	0.78	0.00	0.55	0.07	1.41	0.01
Q9UK22	F-box only protein 2	FBXO2	33 kDa	0.78	0.00	0.65	0.09	1.19	0.06
P15104	Glutamine synthetase	GLUL	42 kDa	0.78	0.00	0.75	0.13	1.03	0.63
O95816	BAG family molecular chaperone regulator 2	BAG2	24 kDa	0.78	0.00	0.85	0.20	0.91	0.20
G5E928	Lysosomal alpha-mannosidase D peptide	MAN2B1	114 kDa	0.78	0.00	0.95	0.50	0.82	0.02
E7EWM9	Extracellular matrix protein FRAS1	FRAS1	79 kDa	0.78	0.00	0.95	0.50	0.82	0.02
Q99622	Protein C10	C12orf57	13 kDa	0.78	0.00	3.05	0.05	0.25	0.04
O15160	Cluster of DNA-directed RNA polymerases I and III subunit	POLR1C	39 kDa	0.78	0.00	1.05	0.50	0.74	0.00
P11177	Pyruvate dehydrogenase E1 component subunit beta, mitoch	PDHB	39 kDa	0.78	0.00	1.15	0.20	0.67	0.00
Q9H267	Vacuolar protein sorting-associated protein 33B	VPS33B	71 kDa	0.78	0.00	1.15	0.20	0.67	0.00
A6NJZ9	Nucleolar complex protein 3 homolog	NOC3L	93 kDa	0.78	0.00	1.15	0.20	0.67	0.00
Q9UKZ9	Procollagen C-endopeptidase enhancer 2	PCOLCE2	46 kDa	0.78	0.00	1.25	0.13	0.62	0.00
Q96ES7	SAGA-associated factor 29 homolog	CCDC101	33 kDa	0.78	0.00	1.25	0.13	0.62	0.00
B4E0G4	PHD finger protein 6	PHF6	38 kDa	0.78	0.00	1.35	0.09	0.57	0.00
O15084	Serine/threonine-protein phosphatase 6 regulatory ankyrin re	ANKRD28	113 kDa	0.78	0.00	1.95	0.03	0.40	0.00
O60244	Mediator of RNA polymerase II transcription subunit 14	MED14	161 kDa	0.78	0.00	1.40	0.00	0.55	0.00
Q13740	Isoform 2 of CD166 antigen	ALCAM	64 kDa	0.78	0.00	0.60	0.00	1.29	0.01
Q16787	Laminin subunit alpha-3	LAMA3	367 kDa	0.78	0.00	0.60	0.00	1.29	0.01
Q92572	AP-3 complex subunit sigma-1	AP3S1	22 kDa	0.78	0.00	1.10	0.00	0.70	0.00
Q96AX1	Vacuolar protein sorting-associated protein 33A	VPS33A	68 kDa	0.78	0.00	1.10	0.00	0.70	0.00

P10809	Cluster of 60 kDa heat shock protein, mitochondrial	HSPD1	61 kDa	0.78	0.00	0.35	0.05	2.21	0.00
Q9BVM2	Cluster of Protein DPCD	DPCD	23 kDa	0.78	0.00	0.95	0.50	0.82	0.02
P62269	40S ribosomal protein S18	RPS18	18 kDa	0.78	0.00	0.70	0.00	1.11	0.06
B5MD17	Chromobox protein homolog 1 (Fragment)	CBX1	19 kDa	0.78	0.00	1.00	1.00	0.78	0.00
Q96GA7	Cluster of Serine dehydratase-like	SDSL	35 kDa	0.78	0.00	1.10	0.00	0.70	0.00
Q9H1A4	Cluster of Anaphase-promoting complex subunit 1	ANAPC1	217 kDa	0.78	0.00	1.20	0.00	0.65	0.00
Q99828	Calcium and integrin-binding protein 1	CIB1	22 kDa	1.30	0.01	2.35	0.07	0.55	0.00
H3BQH0	Cluster of Calponin-2 (Fragment)	CNN2	23 kDa	1.30	0.01	1.05	0.50	1.24	0.02
Q8N9V3	WD repeat, SAM and U-box domain-containing protein 1	WDSUB1	53 kDa	1.30	0.01	2.90	0.10	0.45	0.11
P23921	Ribonucleoside-diphosphate reductase large subunit	RRM1	90 kDa	1.30	0.01	0.75	0.13	1.73	0.00
Q4G0J3	La-related protein 7	LARP7	67 kDa	1.30	0.01	0.95	0.50	1.37	0.01
P54886	Delta-1-pyrroline-5-carboxylate synthase	ALDH18A1	87 kDa	1.30	0.01	0.95	0.50	1.37	0.01
Q5TBA9	Protein furry homolog	FRY	339 kDa	1.30	0.01	1.35	0.09	0.96	0.51
Q9NQ55	Cluster of Suppressor of SWI4 1 homolog	PPAN	53 kDa	1.30	0.01	1.30	0.00	1.00	1.00
B7WPD3	Transcription elongation factor B (SIII), polypeptide 2 (18kD	TCEB2	18 kDa	1.30	0.01	1.00	1.00	1.30	0.03
P05114	Cluster of Non-histone chromosomal protein HMG-14	HMGN1	11 kDa	1.30	0.01	1.60	0.11	0.81	0.03
Q8IV08	Phospholipase D3	PLD3	55 kDa	1.30	0.01	1.15	0.20	1.13	0.09
E9PQK6	Apoptosis inhibitor 5	API5	14 kDa	1.30	0.01	1.15	0.20	1.13	0.09
Q9Y2A9	UDP-GlcNAc:betaGal beta-1,3-N-acetylglucosaminyltransfe	B3GNT3	43 kDa	1.30	0.01	1.10	0.00	1.18	0.02
Q9NV06	DDB1- and CUL4-associated factor 13	DCAF13	51 kDa	1.53	0.00	1.15	0.20	1.33	0.01
E7EUF1	Cluster of Ectonucleotide pyrophosphatase/phosphodiesteras	ENPP2	102 kDa	1.58	0.00	1.80	0.08	0.88	0.08
Q9BUP3	Oxidoreductase HTATIP2	HTATIP2	27 kDa	1.58	0.00	1.35	0.09	1.17	0.04
Q12905	Interleukin enhancer-binding factor 2	ILF2	43 kDa	1.58	0.00	0.60	0.00	2.63	0.00
P10768	Cluster of S-formylglutathione hydrolase	ESD	31 kDa	0.95	0.18	1.15	0.66	0.83	0.57
P06744	Glucose-6-phosphate isomerase	GPI	63 kDa	0.95	0.18	0.25	0.04	3.80	0.00
Q92783	Cluster of Signal transducing adapter molecule 1	STAM	59 kDa	0.95	0.18	0.85	0.50	1.12	0.38
A0MZ66	Cluster of Shootin-1	KIAA1598	72 kDa	0.95	0.18	0.60	0.16	1.58	0.01
A8MW61	Cluster of Pleiotropic regulator 1	PLRG1	57 kDa	0.95	0.18	1.30	0.37	0.73	0.33
Q13813	Cluster of Spectrin alpha chain, non-erythrocytic 1	SPTAN1	285 kDa	0.95	0.18	0.35	0.05	2.71	0.00
H3BLV8	Vacuolar protein sorting-associated protein 29 (Fragment)	VPS29	22 kDa	0.95	0.18	0.70	0.20	1.36	0.03
Q9BRJ7	Cluster of Protein syndesmos	NUDT16L1	23 kDa	0.95	0.18	1.05	0.80	0.90	0.38
P48147	Prolyl endopeptidase	PREP	81 kDa	0.95	0.18	0.80	0.30	1.19	0.12
Q24JP5	Cluster of Transmembrane protein 132A	TMEM132A	110 kDa	0.95	0.18	0.80	0.30	1.19	0.12
Q8WUA2	Peptidyl-prolyl cis-trans isomerase-like 4	PPIL4	57 kDa	0.95	0.18	1.60	0.20	0.59	0.18
E9PGR1	Heparanase 8 kDa subunit	HPSE	55 kDa	0.95	0.18	1.25	0.34	0.76	0.04
H3BM09	Cluster of HLA class I histocompatibility antigen, A-68 alph	HLA-A	34 kDa	0.95	0.18	0.45	0.06	2.11	0.00
Q53H82	Beta-lactamase-like protein 2	LACTB2	33 kDa	0.95	0.18	1.45	0.20	0.66	0.01

O14828	Secretory carrier-associated membrane protein 3	SCAMP3	38 kDa	0.95	0.18	1.00	1.00	0.95	0.54
P62258	Cluster of 14-3-3 protein epsilon	YWHAE	29 kDa	0.95	0.18	0.55	0.07	1.73	0.00
O15355	Protein phosphatase 1G	PPM1G	59 kDa	0.95	0.18	0.55	0.07	1.73	0.00
Q15024	Exosome complex component RRP42	EXOSC7	32 kDa	0.95	0.18	1.10	0.50	0.86	0.12
P35221	Cluster of Catenin alpha-1	CTNNA1	100 kDa	0.95	0.18	0.55	0.07	1.73	0.00
Q9Y2T2	Cluster of AP-3 complex subunit mu-1	AP3M1	47 kDa	0.95	0.18	1.10	0.50	0.86	0.12
B5MD23	Tetraspanin-9	TSPAN9	30 kDa	0.95	0.18	1.20	0.30	0.79	0.03
Q8IX12	Cluster of Cell division cycle and apoptosis regulator protein	CCAR1	133 kDa	0.95	0.18	1.20	0.30	0.79	0.03
P22059	Cluster of Oxysterol-binding protein 1	OSBP	89 kDa	0.95	0.18	0.65	0.09	1.46	0.00
Q03405	Urokinase plasminogen activator surface receptor	PLAUR	37 kDa	0.95	0.18	0.65	0.09	1.46	0.00
Q9BQ61	Uncharacterized protein C19orf43	C19orf43	18 kDa	0.95	0.18	1.30	0.20	0.73	0.01
Q12996	Cleavage stimulation factor subunit 3	CSTF3	83 kDa	0.95	0.18	0.75	0.13	1.27	0.02
Q92520	Cluster of Protein FAM3C	FAM3C	25 kDa	0.95	0.18	0.75	0.13	1.27	0.02
Q93008	Cluster of Probable ubiquitin carboxyl-terminal hydrolase FA	USP9X	292 kDa	0.95	0.18	0.75	0.13	1.27	0.02
P61088	Cluster of Ubiquitin-conjugating enzyme E2 N	UBE2N	17 kDa	0.95	0.18	0.75	0.13	1.27	0.02
B7Z3X4	Cluster of Glucosamine-6-phosphate isomerase 1	GNPDA1	24 kDa	0.95	0.18	0.75	0.13	1.27	0.02
A8MTH6	Sorcin	SRI	20 kDa	0.95	0.18	0.75	0.13	1.27	0.02
O15551	Cluster of Claudin-3	CLDN3	23 kDa	0.95	0.18	0.75	0.13	1.27	0.02
Q13362	Isoform 4 of Serine/threonine-protein phosphatase 2A 56 kD	PPP2R5C	63 kDa	0.95	0.18	1.60	0.11	0.59	0.00
P04114	Apolipoprotein B-100	APOB	516 kDa	0.95	0.18	0.85	0.20	1.12	0.13
O60343	Cluster of TBC1 domain family member 4	TBC1D4	147 kDa	0.95	0.18	0.85	0.20	1.12	0.13
B4DM74	60S ribosomal protein L18a	RPL18A	18 kDa	0.95	0.18	0.85	0.20	1.12	0.13
I3L3Q4	Glyoxalase domain-containing protein 4 (Fragment)	GLOD4	26 kDa	0.95	0.18	0.85	0.20	1.12	0.13
O00743	Serine/threonine-protein phosphatase 6 catalytic subunit	PPP6C	35 kDa	0.95	0.18	0.85	0.20	1.12	0.13
O76021	Ribosomal L1 domain-containing protein 1	RSL1D1	55 kDa	0.95	0.18	0.85	0.20	1.12	0.13
O94992	Cluster of Protein HEXIM1	HEXIM1	41 kDa	0.95	0.18	0.85	0.20	1.12	0.13
O95453	Cluster of Poly(A)-specific ribonuclease PARN	PARN	73 kDa	0.95	0.18	0.85	0.20	1.12	0.13
P09455	Retinol-binding protein 1	RBP1	16 kDa	0.95	0.18	0.85	0.20	1.12	0.13
E9PQE8	Cluster of Trafficking protein particle complex subunit 4	TRAPPC4	19 kDa	0.95	0.18	1.70	0.09	0.56	0.00
P22695	Cytochrome b-c1 complex subunit 2, mitochondrial	UQCRC2	48 kDa	0.95	0.18	0.95	0.50	1.00	1.00
A4D0Z6	Inosine-5'-monophosphate dehydrogenase C	IMPDH1	64 kDa	0.95	0.18	0.95	0.50	1.00	1.00
O43852	Cluster of Calumenin	CALU	37 kDa	0.95	0.18	0.95	0.50	1.00	1.00
P54709	Cluster of Sodium/potassium-transporting ATPase subunit b	ATP1B3	32 kDa	0.95	0.18	0.95	0.50	1.00	1.00
P53634	Cluster of Dipeptidyl peptidase 1	CTSC	52 kDa	0.95	0.18	0.95	0.50	1.00	1.00
P63173	60S ribosomal protein L38	RPL38	8 kDa	0.95	0.18	0.95	0.50	1.00	1.00
H3BNK9	Protein FAM192A (Fragment)	FAM192A	16 kDa	0.95	0.18	1.90	0.07	0.50	0.00
Q8TBA6	Golgin subfamily A member 5	GOLGA5	83 kDa	0.95	0.18	1.05	0.50	0.90	0.13

Q96CV9	Cluster of Optineurin	OPTN	66 kDa	0.95	0.18	1.05	0.50	0.90	0.13
P28370	Probable global transcription activator SNF2L1	SMARCA1	123 kDa	0.95	0.18	1.15	0.20	0.83	0.02
Q9BPU6	Dihydropyrimidinase-related protein 5	DPYSL5	61 kDa	0.95	0.18	1.15	0.20	0.83	0.02
F8WAF2	Cluster of CCR4-NOT transcription complex subunit 10	CNOT10	89 kDa	0.95	0.18	1.15	0.20	0.83	0.02
G3XAP6	Cartilage oligomeric matrix protein	COMP	80 kDa	0.95	0.18	1.15	0.20	0.83	0.02
P12644	Bone morphogenetic protein 4	BMP4	47 kDa	0.95	0.18	1.25	0.13	0.76	0.00
Q8IUR7	Cluster of Isoform 2 of Armadillo repeat-containing protein 8	ARMC8	74 kDa	0.95	0.18	1.25	0.13	0.76	0.00
Q99523	Sortilin	SORT1	92 kDa	0.95	0.18	1.25	0.13	0.76	0.00
Q05209	Tyrosine-protein phosphatase non-receptor type 12	PTPN12	88 kDa	0.95	0.18	1.35	0.09	0.70	0.00
O00442	RNA 3'-terminal phosphate cyclase	RTCA	39 kDa	0.95	0.18	1.45	0.07	0.66	0.00
P03973	Antileukoproteinase	SLPI	14 kDa	0.95	0.18	1.45	0.07	0.66	0.00
C9JNV2	Cluster of Protein BUD31 homolog	BUD31	14 kDa	0.95	0.18	1.45	0.07	0.66	0.00
E7ER20	Cluster of Proteasome inhibitor PI31 subunit	PSMF1	22 kDa	0.95	0.18	1.55	0.06	0.61	0.00
Q7Z7E8	Ubiquitin-conjugating enzyme E2 Q1	UBE2Q1	46 kDa	0.95	0.18	1.65	0.05	0.58	0.00
E9PJM3	Cluster of F-box only protein 3	FBXO3	49 kDa	0.95	0.18	1.85	0.04	0.51	0.00
P49756	Cluster of RNA-binding protein 25	RBM25	100 kDa	0.95	0.18	0.80	0.00	1.19	0.01
P29353	Cluster of SHC-transforming protein 1	SHC1	63 kDa	0.95	0.18	0.90	0.00	1.06	0.18
Q9NVD7	Cluster of Alpha-parvin	PARVA	42 kDa	0.95	0.18	1.00	1.00	0.95	0.18
B4DMQ3	Cluster of Nuclear pore complex protein Nup85	NUP85	70 kDa	0.95	0.18	1.00	1.00	0.95	0.18
Q9Y237	Cluster of Isoform 2 of Peptidyl-prolyl cis-trans isomerase N	PIN4	17 kDa	0.95	0.18	1.40	0.00	0.68	0.00
Q9Y371	Endophilin-B1	SH3GLB1	41 kDa	0.95	0.18	1.40	0.00	0.68	0.00
P98160	Cluster of Basement membrane-specific heparan sulfate prot	HSPG2	469 kDa	0.95	0.18	0.30	0.00	3.17	0.00
F8W8J4	Myoferlin	MYOF	235 kDa	0.95	0.18	0.50	0.00	1.90	0.00
O75533	Splicing factor 3B subunit 1	SF3B1	146 kDa	0.95	0.18	0.50	0.00	1.90	0.00
P16403	Cluster of Histone H1.2	HIST1H1C	21 kDa	0.95	0.18	0.50	0.00	1.90	0.00
P27348	Cluster of 14-3-3 protein theta	YWHAQ	28 kDa	0.95	0.18	0.50	0.00	1.90	0.00
F5HFY4	Cluster of Nucleosome assembly protein 1-like 4	NAP1L4b	44 kDa	0.95	0.18	0.60	0.00	1.58	0.00
G5E9U1	Cdc42-interacting protein 4	TRIP10	68 kDa	0.95	0.18	0.60	0.00	1.58	0.00
Q15020	Cluster of Squamous cell carcinoma antigen recognized by T	SART3	110 kDa	0.95	0.18	0.60	0.00	1.58	0.00
Q3LXA3	Cluster of Bifunctional ATP-dependent dihydroxyacetone ki	DAK	59 kDa	0.95	0.18	0.60	0.00	1.58	0.00
O00469	Isoform 2 of Procollagen-lysine,2-oxoglutarate 5-dioxygenas	PLOD2	87 kDa	0.95	0.18	0.70	0.00	1.36	0.00
H0Y5F5	Cluster of Polyadenylate-binding protein 4 (Fragment)	PABPC4	60 kDa	0.95	0.18	0.70	0.00	1.36	0.00
P00734	Prothrombin	F2	70 kDa	0.95	0.18	0.70	0.00	1.36	0.00
P15927	Isoform 3 of Replication protein A 32 kDa subunit	RPA2	39 kDa	0.95	0.18	0.70	0.00	1.36	0.00
P21283	Cluster of V-type proton ATPase subunit C 1	ATP6V1C1	44 kDa	0.95	0.18	0.80	0.00	1.19	0.01
Q8WXF1	Paraspeckle component 1	PSPC1	59 kDa	0.95	0.18	0.80	0.00	1.19	0.01
Q9GZT8	NIF3-like protein 1	NIF3L1	42 kDa	0.95	0.18	0.80	0.00	1.19	0.01

P62318	Cluster of Small nuclear ribonucleoprotein Sm D3	SNRPD3	14 kDa	0.95	0.18	0.80	0.00	1.19	0.01
B7Z6D9	Cluster of Rho guanine nucleotide exchange factor 7	ARHGEF7	20 kDa	0.95	0.18	0.80	0.00	1.19	0.01
Q9NWH9	SAFB-like transcription modulator	SLTM	117 kDa	0.95	0.18	0.90	0.00	1.06	0.18
Q6P9A2	Putative polypeptide N-acetylgalactosaminyltransferase-like	GALNT18	70 kDa	0.95	0.18	0.90	0.00	1.06	0.18
Q8IUF8	Cluster of Isoform 4 of MYC-induced nuclear antigen	MINA	53 kDa	0.95	0.18	1.00	1.00	0.95	0.18
J3KPP4	Cisplatin resistance-associated overexpressed protein, isofor	LUC7L3	58 kDa	0.95	0.18	1.00	1.00	0.95	0.18
O00159	Cluster of Isoform 3 of Unconventional myosin-Ic	MYO1C	120 kDa	0.95	0.18	1.00	1.00	0.95	0.18
O00622	Protein CYR61	CYR61	42 kDa	0.95	0.18	1.00	1.00	0.95	0.18
Q9H223	EH domain-containing protein 4	EHD4	61 kDa	0.95	0.18	1.10	0.00	0.86	0.01
Q9HCP0	Cluster of Casein kinase I isoform gamma-1	CSNK1G1	49 kDa	0.95	0.18	1.10	0.00	0.86	0.01
Q9NVX2	Notchless protein homolog 1	NLE1	53 kDa	0.95	0.18	1.10	0.00	0.86	0.01
Q9NWT6	Hypoxia-inducible factor 1-alpha inhibitor	HIF1AN	40 kDa	0.95	0.18	1.10	0.00	0.86	0.01
C9JC60	Cluster of Nicotinate phosphoribosyltransferase	NAPRT1	60 kDa	0.95	0.18	1.10	0.00	0.86	0.01
P52298	Cluster of Isoform 2 of Nuclear cap-binding protein subunit :	NCBP2	16 kDa	0.95	0.18	1.10	0.00	0.86	0.01
P10643	Complement component C7	C7	94 kDa	0.95	0.18	1.10	0.00	0.86	0.01
O95983	Isoform 2 of Methyl-CpG-binding domain protein 3	MBD3	29 kDa	0.95	0.18	1.20	0.00	0.79	0.00
P06493	Cyclin-dependent kinase 1	CDK1	34 kDa	0.95	0.18	1.20	0.00	0.79	0.00
Q7Z7M0	Multiple epidermal growth factor-like domains protein 8	MEGF8	303 kDa	0.95	0.18	1.20	0.00	0.79	0.00
Q86U44	N6-adenosine-methyltransferase 70 kDa subunit	METTL3	64 kDa	0.95	0.18	1.20	0.00	0.79	0.00
Q6P275	Cluster of Cohesin subunit SA-1	STAG1	140 kDa	0.95	0.18	1.30	0.00	0.73	0.00
Q96EE3	Nucleoporin SEH1	SEH1L	40 kDa	0.95	0.18	1.30	0.00	0.73	0.00
P00742	Coagulation factor X	F10	55 kDa	0.95	0.18	1.40	0.00	0.68	0.00
P18583	Cluster of Isoform A of Protein SON	SON	232 kDa	0.95	0.18	1.50	0.00	0.63	0.00
H0YH01	A-kinase anchor protein 7 isoform gamma (Fragment)	AKAP7	6 kDa	0.95	0.18	1.50	0.00	0.63	0.00
Q8TAA9	Vang-like protein 1	VANGL1	60 kDa	0.95	0.18	1.70	0.00	0.56	0.00
O95999	B-cell lymphoma/leukemia 10	BCL10	26 kDa	0.95	0.18	1.80	0.00	0.53	0.00
Q8NFU3	Thiosulfate sulfurtransferase/rhodanese-like domain-contain	TSTD1	13 kDa	0.95	0.18	2.00	0.00	0.48	0.00
Q9UM54	Cluster of Isoform 6 of Unconventional myosin-VI	MYO6	149 kDa	0.83	0.01	0.50	0.13	1.65	0.01
Q96P11	Cluster of Isoform 2 of Putative methyltransferase NSUN5	NSUN5	50 kDa	0.83	0.01	0.85	0.50	0.97	0.90
Q15262	Receptor-type tyrosine-protein phosphatase kappa	PTPRK	162 kDa	0.83	0.01	0.60	0.16	1.38	0.04
Q7L014	Cluster of Probable ATP-dependent RNA helicase DDX46	DDX46	117 kDa	0.83	0.01	0.70	0.20	1.18	0.16
P05455	Lupus La protein	SSB	47 kDa	0.83	0.01	0.35	0.05	2.36	0.00
P25685	DnaJ homolog subfamily B member 1	DNAJB1	38 kDa	0.83	0.01	0.70	0.20	1.18	0.16
Q13405	Cluster of 39S ribosomal protein L49, mitochondrial	MRPL49	19 kDa	0.83	0.01	3.20	0.11	0.26	0.11
O95163	Cluster of Elongator complex protein 1	IKBKAP	150 kDa	0.83	0.01	1.35	0.26	0.61	0.17
G5E955	HCG27698, isoform CRA_c	DDX47	45 kDa	0.83	0.01	1.55	0.17	0.53	0.12
E7EPC6	Cluster of CD44 antigen	CD44	79 kDa	0.83	0.01	0.65	0.09	1.27	0.02

P49257	Protein ERGIC-53	LMAN1	58 kDa	0.83	0.01	1.30	0.20	0.63	0.00
P50750	Isoform 2 of Cyclin-dependent kinase 9	CDK9	53 kDa	0.83	0.01	1.40	0.16	0.59	0.00
E9PFR3	Cluster of Serine/threonine-protein phosphatase 2A 56 kDa r	PPP2R5D	69 kDa	0.83	0.01	0.75	0.13	1.10	0.20
O43324	Cluster of Eukaryotic translation elongation factor 1 epsilon-	EEF1E1	20 kDa	0.83	0.01	0.75	0.13	1.10	0.20
Q9H6T3	RNA polymerase II-associated protein 3	RPAP3	76 kDa	0.83	0.01	0.95	0.50	0.87	0.06
Q9P2I0	Cluster of Cleavage and polyadenylation specificity factor su	CPSF2	88 kDa	0.83	0.01	0.95	0.50	0.87	0.06
P18077	60S ribosomal protein L35a	RPL35A	13 kDa	0.83	0.01	0.95	0.50	0.87	0.06
D6RFW1	Small glutamine-rich tetratricopeptide repeat-containing prot	SGTB	22 kDa	0.83	0.01	0.95	0.50	0.87	0.06
D3YTA9	Cluster of Calcineurin subunit B type 1	PPP3R1	21 kDa	0.83	0.01	1.05	0.50	0.79	0.01
E9PGM4	1,4-alpha-glucan-branching enzyme	GBE1	76 kDa	0.83	0.01	1.05	0.50	0.79	0.01
F5H2A4	High mobility group protein HMGI-C	HMGA2	13 kDa	0.83	0.01	1.05	0.50	0.79	0.01
F5H6B8	Splicing factor, suppressor of white-apricot homolog	SFSWAP	110 kDa	0.83	0.01	1.05	0.50	0.79	0.01
Q6GMV2	SET and MYND domain-containing protein 5	SMYD5	47 kDa	0.83	0.01	1.05	0.50	0.79	0.01
Q9BW91	Isoform 2 of ADP-ribose pyrophosphatase, mitochondrial	NUDT9	34 kDa	0.83	0.01	1.15	0.20	0.72	0.00
Q7L2J0	7SK snRNA methylphosphate capping enzyme	MEPCE	74 kDa	0.83	0.01	1.15	0.20	0.72	0.00
Q8IXL6	Cluster of Extracellular serine/threonine protein kinase Fam2	FAM20C	66 kDa	0.83	0.01	1.15	0.20	0.72	0.00
Q8N8R5	UPF0565 protein C2orf69	C2orf69	43 kDa	0.83	0.01	1.15	0.20	0.72	0.00
Q96QU8	Exportin-6	XPO6	129 kDa	0.83	0.01	1.65	0.05	0.50	0.00
Q86UN2	Reticulon-4 receptor-like 1	RTN4RL1	49 kDa	0.83	0.01	1.75	0.04	0.47	0.00
P13497	Cluster of Bone morphogenetic protein 1	BMP1	111 kDa	0.83	0.01	0.60	0.00	1.38	0.00
O95749	Cluster of Geranylgeranyl pyrophosphate synthase	GGPS1	35 kDa	0.83	0.01	1.00	1.00	0.83	0.01
P24539	ATP synthase subunit b, mitochondrial	ATP5F1	29 kDa	0.83	0.01	1.00	1.00	0.83	0.01
Q12972	Nuclear inhibitor of protein phosphatase 1	PPP1R8	38 kDa	0.83	0.01	1.10	0.00	0.75	0.00
Q5T200	Cluster of Zinc finger CCCH domain-containing protein 13	ZC3H13	197 kDa	0.83	0.01	1.10	0.00	0.75	0.00
Q15075	Early endosome antigen 1	EEA1	162 kDa	0.83	0.01	0.50	0.00	1.65	0.00
Q96FQ6	Protein S100-A16	S100A16	12 kDa	0.83	0.01	0.60	0.00	1.38	0.00
H0YN26	Cluster of Acidic leucine-rich nuclear phosphoprotein 32 fan	ANP32A	20 kDa	0.83	0.01	0.70	0.00	1.18	0.02
O00567	Cluster of Nucleolar protein 56	NOP56	66 kDa	0.83	0.01	0.70	0.00	1.18	0.02
O76061	Cluster of Stanniocalcin-2	STC2	33 kDa	0.83	0.01	0.70	0.00	1.18	0.02
Q13033	Striatin-3	STRN3	87 kDa	0.83	0.01	0.90	0.00	0.92	0.06
Q04323	Isoform 2 of UBX domain-containing protein 1	UBXN1	35 kDa	0.83	0.01	1.00	1.00	0.83	0.01
Q86UN3	Reticulon-4 receptor-like 2	RTN4RL2	46 kDa	0.83	0.01	1.10	0.00	0.75	0.00
Q8IV38	Ankyrin repeat and MYND domain-containing protein 2	ANKMY2	49 kDa	0.83	0.01	1.10	0.00	0.75	0.00
Q8IXB1	DnaJ homolog subfamily C member 10	DNAJC10	91 kDa	0.83	0.01	1.10	0.00	0.75	0.00
Q6P597	Kinesin light chain 3	KLC3	55 kDa	0.83	0.01	1.20	0.00	0.69	0.00
O15212	Prefoldin subunit 6	PFDN6	15 kDa	0.83	0.01	1.20	0.63	0.69	0.43
Q8IUL8	Cartilage intermediate layer protein 2	CILP2	126 kDa	0.83	0.01	0.50	0.13	1.65	0.01

P43487	Cluster of Ran-specific GTPase-activating protein	RANBP1	23 kDa	0.83	0.01	0.60	0.16	1.38	0.04
O14617	AP-3 complex subunit delta-1	AP3D1	130 kDa	0.83	0.01	0.70	0.20	1.18	0.16
P36871	Phosphoglucomutase-1	PGM1	61 kDa	0.83	0.01	0.55	0.07	1.50	0.00
C9JYQ9	60S ribosomal protein L22-like 1	RPL22L1	14 kDa	0.83	0.01	1.10	0.50	0.75	0.02
P55036	Cluster of 26S proteasome non-ATPase regulatory subunit 4	PSMD4	41 kDa	0.83	0.01	0.75	0.13	1.10	0.20
Q6P4I2	WD repeat-containing protein 73	WDR73	42 kDa	0.83	0.01	1.50	0.13	0.55	0.00
P38432	Coilin	COIL	63 kDa	0.83	0.01	1.15	0.20	0.72	0.00
P21399	Cytoplasmic aconitate hydratase	ACO1	98 kDa	0.83	0.01	0.70	0.00	1.18	0.02
B7Z5W1	Junctional adhesion molecule A	F11R	33 kDa	0.83	0.01	0.90	0.00	0.92	0.06
Q96ST3	Paired amphipathic helix protein Sin3a	SIN3A	145 kDa	0.83	0.01	1.00	1.00	0.83	0.01
Q9C0C4	Semaphorin-4C	SEMA4C	93 kDa	0.83	0.01	1.00	1.00	0.83	0.01
Q9UH62	Armadillo repeat-containing X-linked protein 3	ARMCX3	43 kDa	0.83	0.01	1.30	0.00	0.63	0.00
B1AUU8	Cluster of Epidermal growth factor receptor pathway substra	EPS15	84 kDa	0.83	0.01	1.60	0.00	0.52	0.00
Q9H1K1	Iron-sulfur cluster assembly enzyme ISCU, mitochondrial	ISCU	18 kDa	2.15	0.00	3.40	0.03	0.63	0.00
A8CTX8	Intersectin 1 long form variant 2	ITSN1	195 kDa	1.63	0.00	1.50	0.00	1.08	0.08
P56377	AP-1 complex subunit sigma-2	AP1S2	19 kDa	1.40	0.00	2.25	0.17	0.62	0.24
Q9BW61	DET1- and DDB1-associated protein 1	DDA1	12 kDa	1.40	0.00	2.30	0.14	0.61	0.20
Q96BH1	E3 ubiquitin-protein ligase RNF25	RNF25	51 kDa	1.40	0.00	2.40	0.09	0.58	0.00
P15880	Cluster of 40S ribosomal protein S2	RPS2	31 kDa	1.40	0.00	0.75	0.13	1.87	0.00
P10646	Cluster of Tissue factor pathway inhibitor	TFPI	35 kDa	1.40	0.00	0.95	0.50	1.47	0.00
Q9UKG1	DCC-interacting protein 13-alpha	APPL1	80 kDa	1.40	0.00	1.15	0.20	1.22	0.02
O75348	V-type proton ATPase subunit G 1	ATP6V1G1	14 kDa	1.40	0.00	1.55	0.06	0.90	0.09
I3L3S0	Trafficking protein particle complex subunit 1 (Fragment)	TRAPPC1	11 kDa	1.40	0.00	2.25	0.03	0.62	0.00
O15400	Syntaxin-7	STX7	30 kDa	1.40	0.00	1.60	0.00	0.88	0.02
P62312	U6 snRNA-associated Sm-like protein LSm6	LSM6	9 kDa	1.40	0.00	0.90	0.00	1.56	0.00
Q8IZ73	RNA pseudouridylate synthase domain-containing protein 2	RPUSD2	61 kDa	1.40	0.00	1.70	0.00	0.82	0.01
Q9UHJ6	Sedoheptulokinase	SHPK	51 kDa	1.40	0.00	0.95	0.50	1.47	0.00
Q96HW7	Cluster of Integrator complex subunit 4	INTS4	108 kDa	1.68	0.00	1.25	0.13	1.34	0.01
Q01518	Cluster of Adenylyl cyclase-associated protein 1	CAP1	52 kDa	1.68	0.00	0.40	0.00	4.19	0.00
P78536	Disintegrin and metalloproteinase domain-containing proteir	ADAM17	93 kDa	0.88	0.02	1.10	0.70	0.80	0.46
O14561	Cluster of Acyl carrier protein, mitochondrial	NDUFAB1	17 kDa	0.88	0.02	1.40	0.30	0.63	0.23
Q9Y5B9	FACT complex subunit SPT16	SUPT16H	120 kDa	0.88	0.02	0.35	0.05	2.50	0.00
Q92688	Acidic leucine-rich nuclear phosphoprotein 32 family membe	ANP32B	29 kDa	0.88	0.02	0.80	0.30	1.09	0.36
J3KNP2	Cluster of Transducin beta-like protein 3 (Fragment)	TBL3	77 kDa	0.88	0.02	1.25	0.34	0.70	0.23
P22102	Cluster of Trifunctional purine biosynthetic protein adenosir	GART	108 kDa	0.88	0.02	0.45	0.06	1.94	0.00
O60524	Nuclear export mediator factor NEMF	NEMF	123 kDa	0.88	0.02	1.00	1.00	0.88	0.16
Q9P2B2	Prostaglandin F2 receptor negative regulator	PTGFRN	99 kDa	0.88	0.02	0.55	0.07	1.59	0.00

Q9BUQ8	Cluster of Probable ATP-dependent RNA helicase DDX23	DDX23	96 kDa	0.88	0.02	0.65	0.09	1.35	0.01
J3QQX2	Cluster of Rho GDP-dissociation inhibitor 1	ARHGDIA	26 kDa	0.88	0.02	0.65	0.09	1.35	0.01
Q2M2I8	Isoform 2 of AP2-associated protein kinase 1	AAK1	94 kDa	0.88	0.02	1.40	0.16	0.63	0.00
Q92785	Zinc finger protein ubi-d4	DPF2	44 kDa	0.88	0.02	1.40	0.16	0.63	0.00
P13489	Cluster of Ribonuclease inhibitor	RNH1	50 kDa	0.88	0.02	0.75	0.13	1.17	0.06
Q7L3B6	Hsp90 co-chaperone Cdc37-like 1	CDC37L1	39 kDa	0.88	0.02	1.50	0.13	0.58	0.00
Q9UNN5	Cluster of FAS-associated factor 1	FAF1	74 kDa	0.88	0.02	0.75	0.13	1.17	0.06
Q9BVG4	Cluster of UPF0368 protein Cxorf26	CXorf26	26 kDa	0.88	0.02	0.95	0.50	0.92	0.20
Q9BZF9	Uveal autoantigen with coiled-coil domains and ankyrin repe	UACA	163 kDa	0.88	0.02	0.95	0.50	0.92	0.20
Q7Z417	Nuclear fragile X mental retardation-interacting protein 2	NUFIP2	76 kDa	0.88	0.02	2.00	0.06	0.44	0.00
P61457	Pterin-4-alpha-carbinolamine dehydratase	PCBD1	12 kDa	0.88	0.02	1.05	0.50	0.83	0.02
C9J0J7	Cluster of Profilin-2	PFN2	10 kDa	0.88	0.02	1.05	0.50	0.83	0.02
Q9NXR7	Cluster of Isoform 1 of BRCA1-A complex subunit BRE	BRE	47 kDa	0.88	0.02	1.05	0.50	0.83	0.02
Q52LW3	Rho GTPase-activating protein 29	ARHGAP29	142 kDa	0.88	0.02	1.15	0.20	0.76	0.00
Q9UNE7	Cluster of E3 ubiquitin-protein ligase CHIP	STUB1	35 kDa	0.88	0.02	1.25	0.13	0.70	0.00
Q9Y4W6	AFG3-like protein 2	AFG3L2	89 kDa	0.88	0.02	1.25	0.13	0.70	0.00
Q13501	Sequestosome-1	SQSTM1	48 kDa	0.88	0.02	1.35	0.09	0.65	0.00
Q99574	Neuroserpin	SERPINI1	46 kDa	0.88	0.02	1.35	0.09	0.65	0.00
Q92882	Osteoclast-stimulating factor 1	OSTF1	24 kDa	0.88	0.02	1.45	0.07	0.60	0.00
Q04837	Single-stranded DNA-binding protein, mitochondrial	SSBP1	17 kDa	0.88	0.02	1.45	0.07	0.60	0.00
Q86X83	COMM domain-containing protein 2	COMMD2	23 kDa	0.88	0.02	1.55	0.06	0.56	0.00
P07108	Cluster of Isoform 4 of Acyl-CoA-binding protein	DBI	14 kDa	0.88	0.02	1.65	0.05	0.53	0.00
F5GWN1	Protein SSXT	SS18	4 kDa	0.88	0.02	2.15	0.03	0.41	0.00
P68104	Elongation factor 1-alpha 1	EEF1A1	50 kDa	0.88	0.02	0.40	0.00	2.19	0.00
P43490	Nicotinamide phosphoribosyltransferase	NAMPT	56 kDa	0.88	0.02	0.50	0.00	1.75	0.00
P07954	Fumarate hydratase, mitochondrial	FH	55 kDa	0.88	0.02	0.60	0.00	1.46	0.00
Q8TAQ2	Cluster of SWI/SNF complex subunit SMARCC2	SMARCC2	133 kDa	0.88	0.02	0.70	0.00	1.25	0.01
Q13085	Cluster of Acetyl-CoA carboxylase 1	ACACA	266 kDa	0.88	0.02	0.80	0.00	1.09	0.06
B0UX83	Cluster of HLA-B associated transcript 3	BAT3	119 kDa	0.88	0.02	0.90	0.00	0.97	0.39
B0UZ83	Cluster of Complement C4-A alpha chain	C4A	193 kDa	0.88	0.02	0.90	0.00	0.97	0.39
O43290	U4/U6.U5 tri-snRNP-associated protein 1	SART1	90 kDa	0.88	0.02	0.90	0.00	0.97	0.39
O75054	Immunoglobulin superfamily member 3	IGSF3	135 kDa	0.88	0.02	0.90	0.00	0.97	0.39
Q9UJX3	Cluster of Anaphase-promoting complex subunit 7	ANAPC7	67 kDa	0.88	0.02	1.20	0.00	0.73	0.00
Q9BPX3	Cluster of Condensin complex subunit 3	NCAPG	114 kDa	0.88	0.02	1.30	0.00	0.67	0.00
Q14181	DNA polymerase alpha subunit B	POLA2	66 kDa	0.88	0.02	2.20	0.00	0.40	0.00
Q96C19	Cluster of EF-hand domain-containing protein D2	EFHD2	27 kDa	0.88	0.02	0.70	0.00	1.25	0.01
Q9BUJ2	Heterogeneous nuclear ribonucleoprotein U-like protein 1	HNRNPUL1	96 kDa	0.88	0.02	0.70	0.00	1.25	0.01

G8JLH6	CD9 antigen (Fragment)	CD9	25 kDa	0.88	0.02	0.70	0.00	1.25	0.01
P48556	26S proteasome non-ATPase regulatory subunit 8	PSMD8	40 kDa	0.88	0.02	0.80	0.00	1.09	0.06
P31942	Heterogeneous nuclear ribonucleoprotein H3	HNRNPH3	37 kDa	0.88	0.02	1.00	1.00	0.88	0.02
P43121	Cluster of Cell surface glycoprotein MUC18	MCAM	72 kDa	0.88	0.02	1.00	1.00	0.88	0.02
P46459	Cluster of Vesicle-fusing ATPase	NSF	83 kDa	0.88	0.02	1.00	1.00	0.88	0.02
J3KMY7	Cluster of SRA stem-loop-interacting RNA-binding protein,	SLIRP	12 kDa	0.88	0.02	1.10	0.00	0.80	0.00
J3KNW0	Cluster of FCH domain only protein 2	FCHO2	89 kDa	0.88	0.02	1.10	0.00	0.80	0.00
A0FGR8	Cluster of Isoform 2 of Extended synaptotagmin-2	ESYT2	99 kDa	0.88	0.02	1.50	0.00	0.58	0.00
Q9BX68	Histidine triad nucleotide-binding protein 2, mitochondrial	HINT2	17 kDa	0.88	0.02	1.60	0.00	0.55	0.00
P12429	Annexin A3	ANXA3	36 kDa	1.05	0.18	0.35	0.05	3.00	0.00
Q9Y5P4	Isoform 3 of Collagen type IV alpha-3-binding protein	COL4A3BP	84 kDa	1.05	0.18	1.05	0.80	1.00	1.00
J3KQB0	THUMP domain-containing protein 1	THUMPD1	49 kDa	1.05	0.18	0.80	0.30	1.31	0.03
P18031	Tyrosine-protein phosphatase non-receptor type 1	PTPN1	50 kDa	1.05	0.18	0.90	0.50	1.17	0.12
O15042	Cluster of U2 snRNP-associated SURP motif-containing pro	U2SURP	118 kDa	1.05	0.18	1.00	1.00	1.05	0.54
O15427	Cluster of Monocarboxylate transporter 4	SLC16A3	49 kDa	1.05	0.18	1.00	1.00	1.05	0.54
E7ESX1	Chordin	CHRD	102 kDa	1.05	0.18	1.00	1.00	1.05	0.54
P61006	Cluster of Ras-related protein Rab-8A	RAB8A	24 kDa	1.05	0.18	0.55	0.07	1.91	0.00
Q9NS15	Cluster of Latent-transforming growth factor beta-binding pr	LTBP3	139 kDa	1.05	0.18	0.55	0.07	1.91	0.00
H7C525	Cluster of Coiled-coil domain-containing protein 58 (Fragme	CCDC58	16 kDa	1.05	0.18	1.10	0.50	0.95	0.54
Q9UPY8	Microtubule-associated protein RP/EB family member 3	MAPRE3	32 kDa	1.05	0.18	2.85	0.09	0.37	0.08
Q8IV36	Cluster of Protein hid-1 homolog	C17orf28	89 kDa	1.05	0.18	1.20	0.30	0.88	0.12
Q9NTK5	Isoform 3 of Obg-like ATPase 1	OLA1	31 kDa	1.05	0.18	0.65	0.09	1.62	0.00
E9PFW3	AP-2 complex subunit mu	AP2M1	52 kDa	1.05	0.18	0.65	0.09	1.62	0.00
F8WBX1	Rho GTPase-activating protein 27	ARHGAP27	95 kDa	1.05	0.18	1.50	0.13	0.70	0.00
J3KPV4	Cluster of DNA replication licensing factor MCM4	MCM4	95 kDa	1.05	0.18	0.75	0.13	1.40	0.00
E7EUG6	Cluster of General transcription factor IIF subunit 1	GTF2F1	48 kDa	1.05	0.18	0.85	0.20	1.24	0.02
Q8WUJ3	Cluster of Protein KIAA1199	CEMIP	153 kDa	1.05	0.18	0.85	0.20	1.24	0.02
Q92747	Actin-related protein 2/3 complex subunit 1A	ARPC1A	42 kDa	1.05	0.18	0.85	0.20	1.24	0.02
Q9H832	Cluster of Ubiquitin-conjugating enzyme E2 Z	UBE2Z	38 kDa	1.05	0.18	0.85	0.20	1.24	0.02
Q9HCY8	Protein S100-A14	S100A14	12 kDa	1.05	0.18	0.85	0.20	1.24	0.02
Q9ULC4	Cluster of Malignant T-cell-amplified sequence 1	MCTS1	21 kDa	1.05	0.18	0.85	0.20	1.24	0.02
P53990	Cluster of Isoform 2 of IST1 homolog	IST1	37 kDa	1.05	0.18	0.85	0.20	1.24	0.02
F8W7C6	Cluster of 60S ribosomal protein L10	RPL10	19 kDa	1.05	0.18	0.85	0.20	1.24	0.02
P98175	RNA-binding protein 10	RBM10	104 kDa	1.05	0.18	1.05	0.50	1.00	1.00
Q14691	DNA replication complex GINS protein PSF1	GINS1	23 kDa	1.05	0.18	1.05	0.50	1.00	1.00
E9PCJ7	Cluster of Putative E3 ubiquitin-protein ligase UBR7	UBR7	40 kDa	1.05	0.18	1.15	0.20	0.91	0.13
Q92793	Cluster of CREB-binding protein	CREBBP	265 kDa	1.05	0.18	1.15	0.20	0.91	0.13

B5MCP9	40S ribosomal protein S7	RPS7	21 kDa	1.05	0.18	1.15	0.20	0.91	0.13
Q9Y6I3	Isoform 2 of Epsin-1	EPN1	69 kDa	1.05	0.18	1.15	0.20	0.91	0.13
Q14498	RNA-binding protein 39	RBM39	59 kDa	1.05	0.18	1.25	0.13	0.84	0.02
Q6P9B6	Cluster of TLD domain-containing protein KIAA1609	TLDC1	51 kDa	1.05	0.18	1.25	0.13	0.84	0.02
P11279	Lysosome-associated membrane glycoprotein 1	LAMP1	45 kDa	1.05	0.18	1.25	0.13	0.84	0.02
Q9H9K5	HERV-MER_4q12 provirus ancestral Env polyprotein	ERVMER34-1	64 kDa	1.05	0.18	1.35	0.09	0.78	0.00
Q96G46	tRNA-dihydrouridine(47) synthase [NAD(P)(+)]-like	DUS3L	73 kDa	1.05	0.18	1.35	0.09	0.78	0.00
F5GXG0	E3 ubiquitin-protein ligase UHRF1	UHRF1	90 kDa	1.05	0.18	1.45	0.07	0.72	0.00
O43286	Beta-1,4-galactosyltransferase 5	B4GALT5	45 kDa	1.05	0.18	1.55	0.06	0.68	0.00
O43505	N-acetyllactosaminide beta-1,3-N-acetylglucosaminyltransfe	B3GNT1	47 kDa	1.05	0.18	1.65	0.05	0.64	0.00
E9PMG1	Cluster of RalBP1-associated Eps domain-containing protein	REPS1	80 kDa	1.05	0.18	2.05	0.03	0.51	0.00
Q16706	Cluster of Alpha-mannosidase 2	MAN2A1	131 kDa	1.05	0.18	0.60	0.00	1.75	0.00
F8VPV9	Cluster of ATP synthase subunit beta	ATP5B	55 kDa	1.05	0.18	0.70	0.00	1.50	0.00
O15067	Cluster of Phosphoribosylformylglycinamidine synthase	PFAS	145 kDa	1.05	0.18	0.70	0.00	1.50	0.00
H0YJ34	Fermitin family homolog 2 (Fragment)	FERMT2	73 kDa	1.05	0.18	0.80	0.00	1.31	0.00
J3KNL3	Cluster of Chitinase domain-containing protein 1	CHID1	48 kDa	1.05	0.18	0.80	0.00	1.31	0.00
E7EX17	Cluster of Uncharacterized protein	EIF4B	70 kDa	1.05	0.18	0.90	0.00	1.17	0.01
F8WCF6	Cluster of Actin-related protein 2/3 complex subunit 4	ARPC4	21 kDa	1.05	0.18	0.90	0.00	1.17	0.01
P13073	Cytochrome c oxidase subunit 4 isoform 1, mitochondrial	COX4I1	20 kDa	1.05	0.18	1.00	1.00	1.05	0.18
P36543	Cluster of Isoform 2 of V-type proton ATPase subunit E 1	ATP6V1E1	24 kDa	1.05	0.18	1.00	1.00	1.05	0.18
P53985	Monocarboxylate transporter 1	SLC16A1	54 kDa	1.05	0.18	1.10	0.00	0.95	0.18
Q13630	GDP-L-fucose synthase	TSTA3	36 kDa	1.05	0.18	1.10	0.00	0.95	0.18
Q0VDF9	Cluster of Heat shock 70 kDa protein 14	HSPA14	55 kDa	1.05	0.18	1.30	0.00	0.81	0.00
P52788	Cluster of Spermine synthase	SMS	41 kDa	1.11	0.13	0.74	0.13	1.50	0.00
Q9UGI8	Cluster of Isoform 2 of Testin	TES	47 kDa	1.05	0.18	0.50	0.00	2.10	0.00
P52907	F-actin-capping protein subunit alpha-1	CAPZA1	33 kDa	1.05	0.18	0.60	0.00	1.75	0.00
Q92616	Translational activator GCN1	GCN1L1	293 kDa	1.05	0.18	0.70	0.00	1.50	0.00
Q9NPH2	Inositol-3-phosphate synthase 1	ISYNA1	61 kDa	1.05	0.18	0.70	0.00	1.50	0.00
Q9UHV9	Prefoldin subunit 2	PFDN2	17 kDa	1.05	0.18	0.80	0.00	1.31	0.00
Q9Y333	U6 snRNA-associated Sm-like protein LSm2	LSM2	11 kDa	1.05	0.18	0.80	0.00	1.31	0.00
Q5VW32	BRO1 domain-containing protein BROX	BROX	46 kDa	1.05	0.18	0.90	0.00	1.17	0.01
Q8IUI8	Cytokine receptor-like factor 3	CRLF3	50 kDa	1.05	0.18	0.90	0.00	1.17	0.01
P49006	MARCKS-related protein	MARCKSL1	20 kDa	1.05	0.18	1.00	1.00	1.05	0.18
P53004	Biliverdin reductase A	BLVRA	33 kDa	1.05	0.18	1.00	1.00	1.05	0.18
Q12860	Contactin-1	CNTN1	113 kDa	1.05	0.18	1.00	1.00	1.05	0.18
P22681	Cluster of E3 ubiquitin-protein ligase CBL	CBL	100 kDa	1.05	0.18	1.10	0.00	0.95	0.18
Q96K76	Isoform 2 of Ubiquitin carboxyl-terminal hydrolase 47	USP47	147 kDa	1.05	0.18	1.10	0.00	0.95	0.18

B8ZZN6	Small ubiquitin-related modifier 1	SUMO1	17 kDa	1.05	0.18	1.20	0.00	0.88	0.01
C9JP16	Cartilage-associated protein	CRTAP	41 kDa	1.05	0.18	1.20	0.00	0.88	0.01
E9PB93	DNA-directed RNA polymerase II subunit RPB4	POLR2D	12 kDa	1.05	0.18	1.20	0.00	0.88	0.01
Q13111	Cluster of Chromatin assembly factor 1 subunit A	CHAF1A	107 kDa	1.05	0.18	1.50	0.00	0.70	0.00
B7Z7B0	Cluster of Cleavage and polyadenylation-specificity factor su	CPSF4	22 kDa	1.05	0.18	1.50	0.00	0.70	0.00
Q15172	Serine/threonine-protein phosphatase 2A 56 kDa regulatory s	PPP2R5A	56 kDa	1.50	0.00	1.40	0.16	1.07	0.31
Q9Y6X9	MORC family CW-type zinc finger protein 2	MORC2	118 kDa	1.50	0.00	1.65	0.05	0.91	0.09
J3QRZ5	Ubiquitin carboxyl-terminal hydrolase	USP14	52 kDa	1.50	0.00	0.70	0.00	2.14	0.00
P18084	Integrin beta-5	ITGB5	88 kDa	1.50	0.00	0.90	0.00	1.67	0.00
Q01581	Cluster of Hydroxymethylglutaryl-CoA synthase, cytoplasmi	HMGCS1	57 kDa	0.93	0.06	0.50	0.13	1.85	0.00
Q9Y230	RuvB-like 2	RUVBL2	51 kDa	0.93	0.06	0.60	0.16	1.54	0.01
P07814	Bifunctional glutamate/proline--tRNA ligase	EPRS	171 kDa	0.97	0.63	0.47	0.02	2.06	0.00
J3QS62	CDK5 regulatory subunit-associated protein 3 (Fragment)	CDK5RAP3	23 kDa	0.93	0.06	1.55	0.17	0.60	0.14
Q9Y3B4	Pre-mRNA branch site protein p14	SF3B14	15 kDa	0.93	0.06	1.10	0.50	0.84	0.07
Q9H944	Cluster of Mediator of RNA polymerase II transcription subu	MED20	23 kDa	0.93	0.06	1.30	0.20	0.71	0.01
E9PHK9	Cluster of Treacle protein	TCOF1	156 kDa	0.93	0.06	0.75	0.13	1.23	0.02
Q9Y2H0	Cluster of Isoform 2 of Disks large-associated protein 4	DLGAP4	108 kDa	0.93	0.06	1.60	0.11	0.58	0.00
C9JFV8	Deoxyguanosine kinase, mitochondrial	DGUOK	30 kDa	0.93	0.06	1.80	0.08	0.51	0.00
B7ZKK9	Cluster of PPP2R5E protein	PPP2R5E	54 kDa	0.93	0.06	1.80	0.08	0.51	0.00
P30405	Peptidyl-prolyl cis-trans isomerase F, mitochondrial	PPIF	22 kDa	0.93	0.06	0.95	0.50	0.97	0.63
P48637	Cluster of Glutathione synthetase	GSS	52 kDa	0.93	0.06	0.95	0.50	0.97	0.63
C9JV86	CD63 antigen	CD63	25 kDa	0.93	0.06	1.05	0.50	0.88	0.06
Q96HR9	Receptor expression-enhancing protein 6	REEP6	21 kDa	0.93	0.06	1.05	0.50	0.88	0.06
Q96JB2	Conserved oligomeric Golgi complex subunit 3	COG3	94 kDa	0.93	0.06	1.05	0.50	0.88	0.06
Q96FX7	Cluster of tRNA (adenine(58)-N(1))-methyltransferase cataly	TRMT61A	31 kDa	0.93	0.06	1.35	0.09	0.69	0.00
Q92804	TATA-binding protein-associated factor 2N	TAF15	62 kDa	0.93	0.06	1.45	0.07	0.64	0.00
F8VW96	Cysteine and glycine-rich protein 2	CSRP2	27 kDa	0.93	0.06	1.45	0.07	0.64	0.00
Q99747	Gamma-soluble NSF attachment protein	NAPG	35 kDa	0.93	0.06	1.65	0.05	0.56	0.00
F5H098	Malate dehydrogenase, cytoplasmic	MDH1	39 kDa	0.93	0.06	0.50	0.00	1.85	0.00
Q9NUJ1	Abhydrolase domain-containing protein 10, mitochondrial	ABHD10	34 kDa	0.93	0.06	0.70	0.00	1.32	0.00
P61964	WD repeat-containing protein 5	WDR5	37 kDa	0.93	0.06	0.80	0.00	1.16	0.02
P67775	Cluster of Serine/threonine-protein phosphatase 2A catalytic	PPP2CA	36 kDa	0.93	0.06	0.80	0.00	1.16	0.02
D3YTB1	60S ribosomal protein L32 (Fragment)	RPL32	16 kDa	0.93	0.06	1.00	1.00	0.93	0.06
D6RAR4	Hepatocyte growth factor activator short chain	HGFAC	71 kDa	0.93	0.06	1.00	1.00	0.93	0.06
P51452	Cluster of Dual specificity protein phosphatase 3	DUSP3	20 kDa	0.93	0.06	1.10	0.00	0.84	0.01
Q5T1M5	FK506-binding protein 15	FKBP15	134 kDa	0.93	0.06	1.20	0.00	0.77	0.00
Q676U5	Isoform 2 of Autophagy-related protein 16-1	ATG16L1	66 kDa	0.93	0.06	1.20	0.00	0.77	0.00

O95297	Myelin protein zero-like protein 1	MPZL1	29 kDa	0.93	0.06	1.30	0.00	0.71	0.00
E9PRZ9	Protein C11orf58 (Fragment)	C11orf58	10 kDa	0.93	0.06	1.60	0.00	0.58	0.00
E7EVY3	Cluster of Calpastatin	CAST	75 kDa	0.97	0.63	0.63	0.09	1.54	0.00
P24592	Insulin-like growth factor-binding protein 6	IGFBP6	25 kDa	0.93	0.06	0.60	0.00	1.54	0.00
Q6UXN9	WD repeat-containing protein 82	WDR82	35 kDa	0.93	0.06	0.70	0.00	1.32	0.00
Q9BTE3	Cluster of Mini-chromosome maintenance complex-binding	MCMBP	73 kDa	0.93	0.06	0.70	0.00	1.32	0.00
Q9UBB4	Cluster of Ataxin-10	ATXN10	53 kDa	0.93	0.06	0.70	0.00	1.32	0.00
P25205	Cluster of DNA replication licensing factor MCM3	MCM3	91 kDa	0.93	0.06	0.80	0.00	1.16	0.02
Q96G03	Cluster of Phosphoglucomutase-2	PGM2	68 kDa	0.93	0.06	0.90	0.00	1.03	0.39
Q96MX6	Cluster of WD repeat-containing protein 92	WDR92	40 kDa	0.93	0.06	0.90	0.00	1.03	0.39
O14802	DNA-directed RNA polymerase III subunit RPC1	POLR3A	156 kDa	0.93	0.06	1.00	1.00	0.93	0.06
O14908	PDZ domain-containing protein GIPC1	GIPC1	36 kDa	0.93	0.06	1.00	1.00	0.93	0.06
P42677	40S ribosomal protein S27	RPS27	9 kDa	0.93	0.06	1.10	0.00	0.84	0.01
P29034	Cluster of Protein S100-A2	S100A2	11 kDa	0.93	0.06	1.10	0.00	0.84	0.01
Q99986	Cluster of Serine/threonine-protein kinase VRK1	VRK1	45 kDa	0.93	0.06	1.10	0.00	0.84	0.01
B4DDT7	5'-AMP-activated protein kinase subunit gamma-1	PRKAG1	34 kDa	0.93	0.06	1.10	0.00	0.84	0.01
B4DI46	Testican-3	SPOCK3	36 kDa	0.93	0.06	1.10	0.00	0.84	0.01
Q8TED0	Cluster of U3 small nucleolar RNA-associated protein 15 ho	UTP15	58 kDa	0.93	0.06	1.20	0.00	0.77	0.00
Q8WVC0	RNA polymerase-associated protein LEO1	LEO1	75 kDa	0.93	0.06	1.20	0.00	0.77	0.00
Q14165	Cluster of Malectin	MLEC	32 kDa	0.93	0.06	1.40	0.00	0.66	0.00
Q9H9A6	Leucine-rich repeat-containing protein 40	LRRC40	68 kDa	0.93	0.06	1.80	0.00	0.51	0.00
Q6XZF7	Dynamin-binding protein	DNMBP	177 kDa	0.93	0.06	1.80	0.30	0.51	0.27
Q8WU90	Zinc finger CCCH domain-containing protein 15	ZC3H15	49 kDa	0.93	0.06	0.85	0.50	1.09	0.70
O00178	GTP-binding protein 1	GTPBP1	72 kDa	0.93	0.06	3.05	0.11	0.30	0.10
P00568	Cluster of Adenylate kinase isoenzyme 1	AK1	22 kDa	0.93	0.06	0.75	0.13	1.23	0.02
P00918	Cluster of Carbonic anhydrase 2	CA2	29 kDa	0.93	0.06	0.75	0.13	1.23	0.02
G3V1P3	Loss of heterozygosity 12 chromosomal region 1 protein	LOH12CR1	20 kDa	0.93	0.06	1.35	0.09	0.69	0.00
Q9NZJ9	Cluster of Diphosphoinositol polyphosphate phosphohydrola	NUDT4	20 kDa	0.93	0.06	1.55	0.06	0.60	0.00
Q969V3	Nicalin	NCLN	63 kDa	0.93	0.06	1.75	0.04	0.53	0.00
P18669	Cluster of Phosphoglycerate mutase 1	PGAM1	29 kDa	0.93	0.06	0.50	0.00	1.85	0.00
P26196	Probable ATP-dependent RNA helicase DDX6	DDX6	54 kDa	0.93	0.06	0.60	0.00	1.54	0.00
A6NM98	Cluster of ARD1 homolog A, N-acetyltransferase (S. cerevis	NAA10	25 kDa	0.93	0.06	0.80	0.00	1.16	0.02
P36507	Dual specificity mitogen-activated protein kinase kinase 2	MAP2K2	44 kDa	0.93	0.06	1.10	0.00	0.84	0.01
Q8WW12	PEST proteolytic signal-containing nuclear protein	PCNP	19 kDa	0.93	0.06	1.10	0.00	0.84	0.01
B5MC40	Transcriptional repressor p66-alpha	GATAD2A	70 kDa	0.93	0.06	1.30	0.00	0.71	0.00
Q8N108	Cluster of Mesoderm induction early response protein 1	MIER1	58 kDa	0.93	0.06	1.50	0.00	0.62	0.00
P29728	2'-5'-oligoadenylate synthase 2	OAS2	82 kDa	0.93	0.06	1.50	0.00	0.62	0.00

D6RF48	Syntaxin-18	STX18	35 kDa	0.93	0.06	1.60	0.00	0.58	0.00
Q9UBB5	Methyl-CpG-binding domain protein 2	MBD2	43 kDa	0.98	0.39	1.75	0.20	0.56	0.20
P05198	Cluster of Eukaryotic translation initiation factor 2 subunit 1	EIF2S1	36 kDa	0.98	0.39	0.65	0.09	1.50	0.00
P18124	Cluster of 60S ribosomal protein L7	RPL7	29 kDa	0.98	0.39	0.65	0.09	1.50	0.00
P69905	Hemoglobin subunit alpha	HBA1	15 kDa	0.98	0.39	0.75	0.13	1.30	0.01
Q96CW5	Gamma-tubulin complex component 3	TUBGCP3	104 kDa	0.98	0.39	1.60	0.11	0.61	0.00
P19784	Casein kinase II subunit alpha'	CSNK2A2	41 kDa	0.98	0.39	0.95	0.50	1.03	0.63
O43719	HIV Tat-specific factor 1	HTATSF1	86 kDa	0.98	0.39	0.95	0.50	1.03	0.63
Q9Y5A9	YTH domain family protein 2	YTHDF2	62 kDa	0.98	0.39	0.95	0.50	1.03	0.63
O14944	Proepiregulin	EREG	19 kDa	0.98	0.39	0.95	0.50	1.03	0.63
Q9UKS6	Protein kinase C and casein kinase substrate in neurons prote	PACSIN3	48 kDa	0.98	0.39	1.05	0.50	0.93	0.20
P62244	Cluster of 40S ribosomal protein S15a	RPS15A	15 kDa	0.98	0.39	1.05	0.50	0.93	0.20
O95750	Fibroblast growth factor 19	FGF19	24 kDa	0.98	0.39	1.15	0.20	0.85	0.02
Q9NSI2	Protein FAM207A	FAM207A	25 kDa	0.98	0.39	1.35	0.09	0.72	0.00
O94832	Unconventional myosin-Id	MYO1D	116 kDa	0.98	0.39	1.35	0.09	0.72	0.00
Q9H3T3	Semaphorin-6B	SEMA6B	95 kDa	0.98	0.39	1.35	0.09	0.72	0.00
Q96B54	Zinc finger protein 428	ZNF428	20 kDa	0.98	0.39	1.45	0.07	0.67	0.00
Q15363	Transmembrane emp24 domain-containing protein 2	TMED2	23 kDa	0.98	0.39	1.55	0.06	0.63	0.00
Q86Y38	Xylosyltransferase 1	XYLT1	108 kDa	0.98	0.39	1.55	0.06	0.63	0.00
O00425	Insulin-like growth factor 2 mRNA-binding protein 3	IGF2BP3	64 kDa	0.98	0.39	1.75	0.04	0.56	0.00
Q96IY1	Cluster of Kinetochore-associated protein NSL1 homolog	NSL1	32 kDa	0.98	0.39	1.75	0.04	0.56	0.00
O00560	Syntenin-1	SDCBP	32 kDa	0.98	0.39	0.70	0.00	1.39	0.00
Q14764	Major vault protein	MVP	99 kDa	0.98	0.39	0.70	0.00	1.39	0.00
Q96DI7	U5 small nuclear ribonucleoprotein 40 kDa protein	SNRNP40	39 kDa	0.98	0.39	0.80	0.00	1.22	0.01
Q8NI36	Cluster of WD repeat-containing protein 36	WDR36	105 kDa	0.98	0.39	1.10	0.00	0.89	0.02
Q9NRN7	L-aminoadipate-semialdehyde dehydrogenase-phosphopante	AASDHPPT	36 kDa	0.98	0.39	1.60	0.00	0.61	0.00
H3BVG0	Cluster of Nuclear pore complex protein Nup93	NUP93	100 kDa	0.93	0.20	0.67	0.09	1.39	0.00
O43776	Asparagine--tRNA ligase, cytoplasmic	NARS	63 kDa	0.98	0.39	0.40	0.00	2.44	0.00
P53621	Coatomer subunit alpha	COPA	138 kDa	0.98	0.39	0.40	0.00	2.44	0.00
F5H2A7	Prefoldin subunit 3	VBP1	22 kDa	0.98	0.39	0.60	0.00	1.63	0.00
O75146	Huntingtin-interacting protein 1-related protein	HIP1R	119 kDa	0.98	0.39	0.80	0.00	1.22	0.01
O95456	Cluster of Proteasome assembly chaperone 1	PSMG1	33 kDa	0.98	0.39	0.80	0.00	1.22	0.01
P09496	Cluster of Clathrin light chain A	CLTA	27 kDa	0.98	0.39	0.80	0.00	1.22	0.01
P10155	Cluster of 60 kDa SS-A/Ro ribonucleoprotein	TROVE2	61 kDa	0.98	0.39	0.80	0.00	1.22	0.01
Q9HAB8	Phosphopantothenate--cysteine ligase	PPCS	34 kDa	0.98	0.39	0.90	0.00	1.08	0.06
Q9NR28	Cluster of Diablo homolog, mitochondrial	DIABLO	27 kDa	0.98	0.39	0.90	0.00	1.08	0.06
Q9BRT9	DNA replication complex GINS protein SLD5	GINS4	26 kDa	0.98	0.39	1.10	0.00	0.89	0.02

O75439	Cluster of Mitochondrial-processing peptidase subunit beta	PMPCB	54 kDa	0.98	0.39	1.20	0.00	0.81	0.00
Q8NEB9	Phosphatidylinositol 3-kinase catalytic subunit type 3	PIK3C3	102 kDa	1.95	0.00	1.40	0.00	1.39	0.00
Q9BYG3	MKI67 FHA domain-interacting nucleolar phosphoprotein	NIFK	34 kDa	0.98	0.39	2.20	0.00	0.44	0.00
P30043	Flavin reductase (NADPH)	BLVRB	22 kDa	1.15	0.01	1.35	0.26	0.85	0.12
Q01780	Cluster of Exosome component 10	EXOSC10	101 kDa	1.15	0.01	1.10	0.50	1.05	0.54
B4DRY3	MOB kinase activator 1B	MOB1B	25 kDa	1.15	0.01	1.40	0.16	0.82	0.03
B7Z9I3	Cluster of Renin receptor	ATP6AP2	36 kDa	1.15	0.01	0.75	0.13	1.53	0.00
Q99615	DnaJ homolog subfamily C member 7	DNAJC7	56 kDa	1.15	0.01	0.70	0.00	1.64	0.00
Q9C0B1	Cluster of Alpha-ketoglutarate-dependent dioxygenase FTO	FTO	58 kDa	1.15	0.01	0.70	0.00	1.64	0.00
O94776	Metastasis-associated protein MTA2	MTA2	75 kDa	1.15	0.01	0.80	0.00	1.44	0.00
F5GXP4	ADP-ribosylation factor-like protein 6-interacting protein 1	ARL6IP1	20 kDa	1.15	0.01	1.20	0.00	0.96	0.18
P12109	Collagen alpha-1(VI) chain	COL6A1	109 kDa	1.15	0.01	0.25	0.04	4.60	0.00
P07203	Glutathione peroxidase 1	GPX1	22 kDa	1.15	0.01	1.20	0.30	0.96	0.54
P49773	Cluster of Histidine triad nucleotide-binding protein 1	HINT1	14 kDa	1.15	0.01	0.85	0.20	1.35	0.00
Q9GZP4	PITH domain-containing protein 1	PITHD1	24 kDa	1.15	0.01	0.95	0.50	1.21	0.02
P23919	Cluster of Thymidylate kinase	DTYMK	24 kDa	1.15	0.01	1.05	0.50	1.10	0.13
P32321	Deoxycytidylate deaminase	DCTD	20 kDa	1.15	0.01	1.25	0.13	0.92	0.13
Q06265	Cluster of Exosome complex component RRP45	EXOSC9	49 kDa	1.15	0.01	0.80	0.00	1.44	0.00
Q08209	Cluster of Isoform 2 of Serine/threonine-protein phosphatase	PPP3CA	58 kDa	1.15	0.01	0.80	0.00	1.44	0.00
F5GYN4	Cluster of Ubiquitin thioesterase OTUB1	OTUB1	28 kDa	1.15	0.01	1.10	0.00	1.05	0.18
P24821	Isoform 6 of Tenascin	TNC	171 kDa	1.15	0.01	2.00	0.00	0.58	0.00
A8MU44	Protein Hook homolog 1	HOOK1	80 kDa	1.10	0.13	1.24	0.13	0.88	0.01
P17931	Cluster of Galectin-3	LGALS3	26 kDa	1.15	0.01	0.70	0.00	1.64	0.00
Q8WVM8	Cluster of Sec1 family domain-containing protein 1	SCFD1	72 kDa	1.15	0.01	0.80	0.00	1.44	0.00
G5E975	SWI/SNF related, matrix associated, actin dependent regulat	SMARCB1	45 kDa	1.15	0.01	1.00	1.00	1.15	0.01
J3KQG9	UPF0696 protein C11orf68	C11orf68	32 kDa	1.15	0.01	1.00	1.00	1.15	0.01
H0YHG0	Uncharacterized protein (Fragment)		59 kDa	1.15	0.01	1.10	0.00	1.05	0.18
Q58WW2	Isoform 3 of DDB1- and CUL4-associated factor 6	DCAF6	107 kDa	1.15	0.01	1.40	0.00	0.82	0.00
B5MCQ5	Protein disulfide-isomerase A6	PDIA6	53 kDa	1.15	0.01	0.70	0.20	1.64	0.00
P17174	Cluster of Aspartate aminotransferase, cytoplasmic	GOT1	46 kDa	1.15	0.01	0.55	0.07	2.09	0.00
P08579	U2 small nuclear ribonucleoprotein B"	SNRPB2	25 kDa	1.15	0.01	0.85	0.20	1.35	0.00
P62699	Protein yippee-like 5	YPEL5	14 kDa	1.15	0.01	3.60	0.05	0.32	0.05
P15514	Amphiregulin	AREG	28 kDa	1.15	0.01	0.95	0.50	1.21	0.02
P60033	Cluster of CD81 antigen	CD81	26 kDa	1.15	0.01	0.60	0.00	1.92	0.00
E9PDC5	Receptor-type tyrosine-protein phosphatase S	PTPRS	217 kDa	1.15	0.01	0.60	0.00	1.92	0.00
O43765	Small glutamine-rich tetratricopeptide repeat-containing prot	SGTA	34 kDa	1.15	0.01	0.80	0.00	1.44	0.00
P49755	Transmembrane emp24 domain-containing protein 10	TMED10	25 kDa	1.15	0.01	0.90	0.00	1.28	0.00

C9JME2	FERM, RhoGEF and pleckstrin domain-containing protein 1	FARP1	122 kDa	1.15	0.01	1.30	0.00	0.88	0.01
P61927	60S ribosomal protein L37	RPL37	11 kDa	1.15	0.01	1.30	0.00	0.88	0.01
Q5LJB0	Cluster of Ubiquitin carboxyl-terminal hydrolase L5 (Fragm	UCHL5	29 kDa	1.03	0.39	0.65	0.26	1.58	0.23
P54289	Voltage-dependent calcium channel subunit alpha-2/delta-1	CACNA2D1	125 kDa	1.03	0.39	1.55	0.36	0.66	0.37
Q99496	E3 ubiquitin-protein ligase RING2	RNF2	38 kDa	1.03	0.39	1.45	0.32	0.71	0.34
E5RJF8	Centrin-3	CETN3	22 kDa	1.03	0.39	1.30	0.37	0.79	0.40
O60749	Cluster of Sorting nexin-2	SNX2	58 kDa	1.03	0.39	0.80	0.30	1.28	0.04
Q9UQE7	Structural maintenance of chromosomes protein 3	SMC3	142 kDa	1.03	0.39	0.65	0.09	1.58	0.00
O14579	Coatomer subunit epsilon	COPE	34 kDa	1.03	0.39	0.65	0.09	1.58	0.00
Q9Y3B3	Cluster of Transmembrane emp24 domain-containing protei	TMED7	25 kDa	1.03	0.39	1.30	0.20	0.79	0.02
E5RFP0	NudC domain-containing protein 2	NUDCD2	15 kDa	1.03	0.39	1.40	0.16	0.73	0.01
J3KRD0	Archaemetzincin-2 (Fragment)	AMZ2	28 kDa	1.03	0.39	1.60	0.11	0.64	0.00
F6QR24	Nuclear pore complex protein Nup153	NUP153	157 kDa	1.03	0.39	1.70	0.09	0.60	0.00
Q9Y5S2	Cluster of Serine/threonine-protein kinase MRCK beta	CDC42BPB	194 kDa	1.03	0.39	0.85	0.20	1.21	0.02
Q9UL25	Ras-related protein Rab-21	RAB21	24 kDa	1.03	0.39	0.95	0.50	1.08	0.20
C9JQD4	Cluster of Peptidyl-prolyl cis-trans isomerase (Fragment)	PPIH	16 kDa	1.03	0.39	0.95	0.50	1.08	0.20
D3DQV9	Cluster of Eukaryotic translation initiation factor 4 gamma 2	EIF4G2	102 kDa	1.03	0.39	0.95	0.50	1.08	0.20
Q96C36	Cluster of Pyrroline-5-carboxylate reductase 2	PYCR2	34 kDa	1.03	0.39	1.05	0.50	0.98	0.63
O43291	Kunitz-type protease inhibitor 2	SPINT2	28 kDa	1.03	0.39	1.05	0.50	0.98	0.63
O75175	Cluster of CCR4-NOT transcription complex subunit 3	CNOT3	82 kDa	1.03	0.39	1.05	0.50	0.98	0.63
O43760	Synaptogyrin-2	SYNGR2	25 kDa	1.03	0.39	1.15	0.20	0.89	0.06
Q12929	Cluster of Epidermal growth factor receptor kinase substrate	EPS8	92 kDa	1.03	0.39	1.15	0.20	0.89	0.06
Q03169	Cluster of Tumor necrosis factor alpha-induced protein 2	TNFAIP2	73 kDa	1.03	0.39	1.25	0.13	0.82	0.01
E7ETV8	Importin-5 (Fragment)	IPO5	14 kDa	1.03	0.39	1.25	0.13	0.82	0.01
P82094	TATA element modulatory factor	TMF1	123 kDa	1.03	0.39	1.35	0.09	0.76	0.00
Q9NWS0	PIH1 domain-containing protein 1	PIH1D1	32 kDa	1.03	0.39	1.35	0.09	0.76	0.00
B8ZZY2	Arf-GAP domain and FG repeat-containing protein 1	AGFG1	56 kDa	1.03	0.39	1.45	0.07	0.71	0.00
P17655	Cluster of Calpain-2 catalytic subunit	CAPN2	80 kDa	1.03	0.39	0.30	0.00	3.42	0.00
O75083	Cluster of WD repeat-containing protein 1	WDR1	66 kDa	1.03	0.39	0.40	0.00	2.56	0.00
O00499	Cluster of Isoform IIB of Myc box-dependent-interacting pro	BIN1	56 kDa	1.03	0.39	0.80	0.00	1.28	0.00
O75937	DnaJ homolog subfamily C member 8	DNAJC8	30 kDa	1.03	0.39	0.80	0.00	1.28	0.00
Q9NX58	Cell growth-regulating nucleolar protein	LYAR	44 kDa	1.03	0.39	0.80	0.00	1.28	0.00
Q13895	Bystin	BYSL	50 kDa	1.03	0.39	1.10	0.00	0.93	0.06
Q7L266	Cluster of L-asparaginase	ASRGL1	32 kDa	1.03	0.39	1.10	0.00	0.93	0.06
O95372	Acyl-protein thioesterase 2	LYPLA2	25 kDa	1.03	0.39	1.20	0.00	0.85	0.01
Q6UW63	KDEL motif-containing protein 1	KDELC1	58 kDa	1.03	0.39	1.50	0.00	0.68	0.00
P09234	Cluster of U1 small nuclear ribonucleoprotein C	SNRPC	17 kDa	1.03	0.39	2.00	0.00	0.51	0.00

Q9NZL9	Cluster of Methionine adenosyltransferase 2 subunit beta	MAT2B	38 kDa	1.03	0.39	0.70	0.00	1.46	0.00
Q9Y295	Cluster of Developmentally-regulated GTP-binding protein 1	DRG1	41 kDa	1.03	0.39	0.70	0.00	1.46	0.00
O00571	Cluster of ATP-dependent RNA helicase DDX3X	DDX3X	73 kDa	1.03	0.39	0.80	0.00	1.28	0.00
O60925	Prefoldin subunit 1	PFDN1	14 kDa	1.03	0.39	0.80	0.00	1.28	0.00
Q5VZK9	Leucine-rich repeat-containing protein 16A	LRRC16A	152 kDa	1.03	0.39	1.00	1.00	1.03	0.39
Q96PU5	Cluster of Isoform 6 of E3 ubiquitin-protein ligase NEDD4-l	NEDD4L	109 kDa	1.03	0.39	1.10	0.00	0.93	0.06
J3QK89	Calcium homeostasis endoplasmic reticulum protein	CHERP	105 kDa	1.03	0.39	1.20	0.00	0.85	0.01
J3QTA2	BAG family molecular chaperone regulator 1	BAG1	39 kDa	1.03	0.39	1.20	0.00	0.85	0.01
P45985	Dual specificity mitogen-activated protein kinase kinase 4	MAP2K4	44 kDa	1.03	0.39	1.80	0.00	0.57	0.00
O00299	Chloride intracellular channel protein 1	CLIC1	27 kDa	1.08	0.06	0.45	0.06	2.39	0.00
C9J2K4	V-type proton ATPase subunit F	ATP6V1F	16 kDa	1.08	0.06	1.00	1.00	1.08	0.36
P46060	Cluster of Ran GTPase-activating protein 1	RANGAP1	64 kDa	1.08	0.06	0.65	0.09	1.65	0.00
O60671	Cell cycle checkpoint protein RAD1	RAD1	32 kDa	1.08	0.06	1.60	0.11	0.67	0.00
Q9NRL2	Bromodomain adjacent to zinc finger domain protein 1A	BAZ1A	179 kDa	1.08	0.06	4.25	0.05	0.25	0.05
Q8TEX9	Cluster of Isoform 2 of Importin-4	IPO4	119 kDa	1.08	0.06	0.85	0.20	1.26	0.01
Q96DG6	Carboxymethylenebutenolidase homolog	CMBL	28 kDa	1.08	0.06	0.95	0.50	1.13	0.06
Q15648	Mediator of RNA polymerase II transcription subunit 1	MED1	168 kDa	1.08	0.06	2.00	0.06	0.54	0.00
B1AJY7	26S proteasome non-ATPase regulatory subunit 10	PSMD10	21 kDa	1.08	0.06	1.05	0.50	1.02	0.63
O00151	PDZ and LIM domain protein 1	PDLIM1	36 kDa	1.08	0.06	1.05	0.50	1.02	0.63
O15479	Melanoma-associated antigen B2	MAGEB2	35 kDa	1.08	0.06	1.05	0.50	1.02	0.63
B7ZKS7	Cluster of USP48 protein	USP48	121 kDa	1.08	0.06	1.15	0.20	0.93	0.20
P81605	Dermcidin	DCD	11 kDa	1.08	0.06	1.25	0.13	0.86	0.02
Q9H3G5	Cluster of Probable serine carboxypeptidase CPVL	CPVL	54 kDa	1.08	0.06	0.70	0.00	1.54	0.00
Q9HAV7	GrpE protein homolog 1, mitochondrial	GRPEL1	24 kDa	1.08	0.06	0.70	0.00	1.54	0.00
E9PEP6	Cluster of Neuropilin-1	NRP1	101 kDa	1.08	0.06	0.70	0.00	1.54	0.00
B4E1Z4	Cluster of Complement factor B Ba fragment	CFB	141 kDa	1.08	0.06	0.80	0.00	1.34	0.00
P09958	Cluster of Furin	FURIN	87 kDa	1.19	0.01	1.11	0.00	1.08	0.06
P53365	Cluster of Arfaptin-2	ARFIP2	38 kDa	1.08	0.06	1.10	0.00	0.98	0.39
P53611	Geranylgeranyl transferase type-2 subunit beta	RABGGTB	37 kDa	1.08	0.06	1.10	0.00	0.98	0.39
Q12805	Cluster of EGF-containing fibulin-like extracellular matrix p	EFEMP1	55 kDa	1.08	0.06	1.10	0.00	0.98	0.39
Q15181	Inorganic pyrophosphatase	PPA1	33 kDa	1.08	0.06	0.65	0.09	1.65	0.00
D6R9T0	Cluster of Heterogeneous nuclear ribonucleoprotein H, N-ter	HNRNPH1	18 kDa	1.08	0.06	0.65	0.09	1.65	0.00
P50552	Vasodilator-stimulated phosphoprotein	VASP	40 kDa	1.08	0.06	0.85	0.20	1.26	0.01
F5GYQ8	Acyl-coenzyme A oxidase	ACOX1	70 kDa	1.08	0.06	1.15	0.20	0.93	0.20
Q9C0C2	182 kDa tankyrase-1-binding protein	TNKS1BP1	182 kDa	1.08	0.06	1.35	0.09	0.80	0.00
H7C4F1	Cluster of Arf-GAP with GTPase, ANK repeat and PH doma	AGAP3	21 kDa	1.08	0.06	1.45	0.07	0.74	0.00
Q9GZL7	Ribosome biogenesis protein WDR12	WDR12	48 kDa	1.08	0.06	0.80	0.00	1.34	0.00

A8MZB2	N-acetyltransferase 5	NAA20	19 kDa	1.08	0.06	0.90	0.00	1.19	0.01
B4DL14	ATP synthase gamma chain	ATP5C1	28 kDa	1.08	0.06	0.90	0.00	1.19	0.01
O00273	DNA fragmentation factor subunit alpha	DFFA	37 kDa	1.08	0.06	1.00	1.00	1.08	0.06
O60508	Pre-mRNA-processing factor 17	CDC40	66 kDa	1.08	0.06	1.10	0.00	0.98	0.39
Q9C0J8	Cluster of pre-mRNA 3' end processing protein WDR33	WDR33	146 kDa	1.08	0.06	1.20	0.00	0.90	0.02
Q92954	Cluster of Proteoglycan 4	PRG4	151 kDa	1.08	0.06	1.30	0.00	0.83	0.00
F8WBS8	26S proteasome non-ATPase regulatory subunit 2	PSMD2	7 kDa	1.08	0.06	1.70	0.00	0.63	0.00
O75521	Enoyl-CoA delta isomerase 2, mitochondrial	ECI2	44 kDa	1.08	0.06	2.00	0.00	0.54	0.00
P80303	Cluster of Nucleobindin-2	NUCB2	50 kDa	1.25	0.00	0.55	0.07	2.27	0.00
P43243	Cluster of Matrin-3	MATR3	95 kDa	1.25	0.00	0.65	0.09	1.92	0.00
Q5JPT4	SH3 domain-containing kinase-binding protein 1	SH3KBP1	47 kDa	1.25	0.00	0.75	0.13	1.67	0.00
O00462	Beta-mannosidase	MANBA	101 kDa	1.25	0.00	0.85	0.20	1.47	0.00
P15121	Aldose reductase	AKR1B1	36 kDa	1.25	0.00	1.05	0.50	1.19	0.02
Q9UEY8	Isoform 1 of Gamma-adducin	ADD3	76 kDa	1.25	0.00	1.15	0.20	1.09	0.13
C9JJV1	Protein TSSC4 (Fragment)	TSSC4	22 kDa	1.25	0.00	2.40	0.05	0.52	0.00
O60243	Heparan-sulfate 6-O-sulfotransferase 1	HS6ST1	48 kDa	1.25	0.00	1.25	0.13	1.00	1.00
Q8NC60	Nitric oxide-associated protein 1	NOA1	78 kDa	1.25	0.00	2.65	0.02	0.47	0.00
E9PDY5	Cluster of Heparan-sulfate 6-O-sulfotransferase 2	HS6ST2	73 kDa	1.25	0.00	0.80	0.00	1.56	0.00
Q13595	Isoform Short of Transformer-2 protein homolog alpha	TRA2A	13 kDa	1.25	0.00	1.30	0.00	0.96	0.18
E9PEZ3	Cluster of Protein diaphanous homolog 1	DIAPH1	141 kDa	1.25	0.00	0.50	0.00	2.50	0.00
Q00059	Cluster of Transcription factor A, mitochondrial	TFAM	29 kDa	1.25	0.00	1.10	0.00	1.14	0.01
B4DFL2	Isocitrate dehydrogenase [NADP]	IDH2	45 kDa	1.25	0.00	1.20	0.00	1.04	0.18
C9JPM4	ADP-ribosylation factor 4 (Fragment)	ARF4	15 kDa	1.25	0.00	0.75	0.13	1.67	0.00
P12532	Cluster of Creatine kinase U-type, mitochondrial	CKMT1A	47 kDa	1.25	0.00	0.95	0.50	1.32	0.00
Q92692	Isoform Alpha of Poliovirus receptor-related protein 2	PVRL2	51 kDa	1.25	0.00	1.15	0.20	1.09	0.13
O60437	Periplakin	PPL	205 kDa	1.13	0.02	0.85	0.50	1.32	0.31
O75400	Pre-mRNA-processing factor 40 homolog A	PRPF40A	109 kDa	1.13	0.02	0.90	0.50	1.25	0.04
Q15008	Cluster of 26S proteasome non-ATPase regulatory subunit 6	PSMD6	46 kDa	1.13	0.02	0.55	0.07	2.05	0.00
Q5VTR2	E3 ubiquitin-protein ligase BRE1A	RNF20	114 kDa	1.13	0.02	1.10	0.50	1.02	0.75
O96008	Mitochondrial import receptor subunit TOM40 homolog	TOMM40	38 kDa	1.13	0.02	1.10	0.50	1.02	0.75
P49137	Cluster of MAP kinase-activated protein kinase 2	MAPKAPK2	46 kDa	1.13	0.02	1.20	0.30	0.94	0.36
Q99584	Protein S100-A13	S100A13	11 kDa	1.13	0.02	0.85	0.20	1.32	0.00
P22087	rRNA 2'-O-methyltransferase fibrillarin	FBL	34 kDa	1.13	0.02	0.85	0.20	1.32	0.00
A8MX94	Glutathione S-transferase P	GSTP1	19 kDa	1.13	0.02	0.85	0.20	1.32	0.00
Q96BW5	Phosphotriesterase-related protein	PTER	39 kDa	1.13	0.02	0.95	0.50	1.18	0.02
P46531	Neurogenic locus notch homolog protein 1	NOTCH1	272 kDa	1.13	0.02	1.05	0.50	1.07	0.20
P51511	Matrix metalloproteinase-15	MMP15	76 kDa	1.13	0.02	1.25	0.13	0.90	0.06

Q9GZX9	Twisted gastrulation protein homolog 1	TWSG1	25 kDa	1.13	0.02	1.45	0.07	0.78	0.00
Q03111	Protein ENL	MLLT1	62 kDa	1.13	0.02	1.45	0.07	0.78	0.00
Q8N1Q1	Carbonic anhydrase 13	CA13	29 kDa	1.13	0.02	1.55	0.06	0.73	0.00
O43396	Cluster of Thioredoxin-like protein 1	TXNL1	32 kDa	1.13	0.02	0.70	0.00	1.61	0.00
Q6NXG1	Cluster of Epithelial splicing regulatory protein 1	ESRP1	76 kDa	1.13	0.02	0.90	0.00	1.25	0.00
O60645	Exocyst complex component 3	EXOC3	87 kDa	1.13	0.02	1.80	0.00	0.63	0.00
Q9BSB4	Autophagy-related protein 101	ATG101	25 kDa	1.13	0.02	2.60	0.00	0.43	0.00
Q14008	Cluster of Cytoskeleton-associated protein 5	CKAP5	226 kDa	1.13	0.02	0.50	0.00	2.25	0.00
P25787	Proteasome subunit alpha type-2	PSMA2	26 kDa	1.13	0.02	0.70	0.00	1.61	0.00
H7C0V4	Reticulon-4 receptor (Fragment)	RTN4R	53 kDa	1.13	0.02	1.10	0.00	1.02	0.39
G3XAE6	Extracellular sulfatase Sulf-2	SULF2	98 kDa	1.13	0.02	1.50	0.00	0.75	0.00
Q32P41	Cluster of tRNA (guanine(37)-N1)-methyltransferase	TRMT5	58 kDa	1.13	0.02	1.50	0.00	0.75	0.00
P23469	Cluster of Receptor-type tyrosine-protein phosphatase epsilo	PTPRE	81 kDa	1.35	0.00	1.35	0.09	1.00	1.00
Q9NRB3	Carbohydrate sulfotransferase 12	CHST12	48 kDa	1.35	0.00	3.50	0.03	0.39	0.00
Q9BTY2	Plasma alpha-L-fucosidase	FUCA2	54 kDa	1.35	0.00	0.90	0.00	1.50	0.00
Q9NY97	UDP-GlcNAc:betaGal beta-1,3-N-acetylglucosaminyltransfe	B3GNT2	46 kDa	1.35	0.00	0.75	0.13	1.80	0.00
F8W031	Uncharacterized protein (Fragment)		29 kDa	1.35	0.00	1.25	0.13	1.08	0.13
Q9HAT2	Sialate O-acetylesterase	SIAE	58 kDa	1.35	0.00	1.20	0.00	1.13	0.01
Q9P2E9	Cluster of Ribosome-binding protein 1	RRBP1	152 kDa	1.18	0.01	0.55	0.07	2.14	0.00
P31040	Succinate dehydrogenase [ubiquinone] flavoprotein subunit,	SDHA	73 kDa	1.18	0.01	0.95	0.50	1.24	0.01
O94760	Cluster of N(G),N(G)-dimethylarginine dimethylaminohydrc	DDAH1	31 kDa	1.18	0.01	1.25	0.13	0.94	0.20
Q96QK1	Cluster of Vacuolar protein sorting-associated protein 35	VPS35	92 kDa	1.18	0.01	0.50	0.00	2.35	0.00
E7ES19	Cluster of Thrombospondin-4	THBS4	96 kDa	1.18	0.01	1.10	0.00	1.07	0.06
O75223	Cluster of Gamma-glutamylcyclotransferase	GGCT	21 kDa	1.18	0.01	0.90	0.50	1.31	0.02
E7EWZ6	Integrin alpha-V light chain	ITGAV	111 kDa	1.18	0.01	1.00	1.00	1.18	0.07
F8W6G1	Nuclear receptor-binding protein	NRBP1	61 kDa	1.18	0.01	1.45	0.07	0.81	0.00
B4DJA5	Ras-related protein Rab-5A	RAB5A	22 kDa	1.18	0.01	1.45	0.07	0.81	0.00
O75554	WW domain-binding protein 4	WBP4	43 kDa	1.18	0.01	1.55	0.06	0.76	0.00
P01009	Alpha-1-antitrypsin	SERPINA1	47 kDa	1.18	0.01	1.55	0.06	0.76	0.00
O75153	Cluster of Clustered mitochondria protein homolog	KIAA0664	147 kDa	1.18	0.01	0.80	0.00	1.47	0.00
Q00839	Cluster of Heterogeneous nuclear ribonucleoprotein U	HNRNPU	91 kDa	1.18	0.01	0.70	0.00	1.68	0.00
P39060	Collagen alpha-1(XVIII) chain	COL18A1	178 kDa	1.18	0.01	0.90	0.00	1.31	0.00
Q9UDY2	Isoform C1 of Tight junction protein ZO-2	TJP2	131 kDa	1.18	0.01	1.00	1.00	1.18	0.01
Q9UBK9	Cluster of Protein UXT	UXT	18 kDa	1.23	0.00	1.40	0.30	0.88	0.54
Q9H7B2	Ribosome production factor 2 homolog	RPF2	36 kDa	1.23	0.00	1.70	0.09	0.72	0.00
Q9H4E7	Cluster of Differentially expressed in FDCP 6 homolog	DEF6	74 kDa	1.23	0.00	1.55	0.06	0.79	0.00
H3BPF6	Cluster of Prefoldin subunit 5 (Fragment)	PFDN5	17 kDa	1.23	0.00	0.80	0.00	1.53	0.00

Q9UKY7	Protein CDV3 homolog	CDV3	27 kDa	1.23	0.00	1.90	0.07	0.64	0.00
Q9BXR0	Cluster of Queuine tRNA-ribosyltransferase	QTRT1	44 kDa	1.23	0.00	1.15	0.20	1.07	0.20
E9PED3	Ankycorbin	RAI14	107 kDa	1.23	0.00	1.25	0.13	0.98	0.63
F5GXF7	Cluster of Zinc finger protein 185	ZNF185	77 kDa	1.23	0.00	1.25	0.13	0.98	0.63
O75752	UDP-GalNAc:beta-1,3-N-acetylgalactosaminyltransferase 1	B3GALNT1	40 kDa	1.23	0.00	1.85	0.04	0.66	0.00
Q2NL82	Pre-rRNA-processing protein TSR1 homolog	TSR1	92 kDa	1.23	0.00	2.25	0.03	0.54	0.00
O60231	Putative pre-mRNA-splicing factor ATP-dependent RNA hel	DHX16	119 kDa	1.23	0.00	1.40	0.00	0.88	0.01
Q9P273	Teneurin-3	TENM3	301 kDa	1.23	0.00	1.00	1.00	1.23	0.00
Q96HS1	Cluster of Serine/threonine-protein phosphatase PGAM5, mi	PGAM5	32 kDa	1.23	0.00	1.10	0.00	1.11	0.02
B7Z4K6	Deoxyribonuclease-2-alpha	DNASE2	34 kDa	1.23	0.00	1.30	0.00	0.94	0.06
B7Z2R2	Cytochrome b-c1 complex subunit 7	UQCRB	19 kDa	1.23	0.00	1.30	0.00	0.94	0.06
Q8IZ83	Cluster of Aldehyde dehydrogenase family 16 member A1	ALDH16A1	85 kDa	1.45	0.00	0.65	0.09	2.23	0.00
Q8NCN5	Pyruvate dehydrogenase phosphatase regulatory subunit, mit	PDPR	99 kDa	1.45	0.00	1.20	0.00	1.21	0.00
E9PBB0	PERQ amino acid-rich with GYF domain-containing protein	GIGYF2	149 kDa	1.38	0.00	3.62	0.02	0.38	0.11
Q9NX55	Cluster of Huntingtin-interacting protein K	HYPK	15 kDa	1.45	0.00	2.05	0.03	0.71	0.00
Q5TH30	NDRG family member 3	NDRG3	43 kDa	1.45	0.00	1.20	0.00	1.21	0.00
O95373	Importin-7	IPO7	120 kDa	1.45	0.00	0.60	0.00	2.42	0.00
Q12962	Transcription initiation factor TFIID subunit 10	TAF10	22 kDa	1.45	0.00	1.90	0.00	0.76	0.00
Q5VYB9	Cluster of Low density lipoprotein receptor-related protein 1	LRP11	35 kDa	1.28	0.00	1.05	0.80	1.21	0.37
O75695	Protein XRP2	RP2	40 kDa	1.28	0.00	1.60	0.20	0.80	0.35
P15407	Fos-related antigen 1	FOSL1	29 kDa	1.28	0.00	1.90	0.07	0.67	0.00
Q53H96	Pyrroline-5-carboxylate reductase 3	PYCRL	29 kDa	1.28	0.00	1.25	0.13	1.02	0.63
Q5R2V8	C2orf4 protein	MEMO1	34 kDa	1.28	0.00	1.30	0.00	0.98	0.39
P31153	Cluster of S-adenosylmethionine synthase isoform type-2	MAT2A	44 kDa	1.28	0.00	0.60	0.00	2.13	0.00
F5H241	Glucosylceramidase	GBA	48 kDa	1.28	0.00	0.90	0.50	1.42	0.01
F5H6I0	Beta-2-microglobulin form pI 5.3	B2M	14 kDa	1.28	0.00	0.95	0.50	1.34	0.00
Q9NYB0	Cluster of Telomeric repeat-binding factor 2-interacting prot	TERF2IP	44 kDa	1.28	0.00	1.15	0.20	1.11	0.06
O75976	Carboxypeptidase D	CPD	153 kDa	1.28	0.00	0.90	0.00	1.42	0.00
P61353	60S ribosomal protein L27	RPL27	16 kDa	1.33	0.00	0.60	0.16	2.21	0.00
Q15006	ER membrane protein complex subunit 2	EMC2	35 kDa	1.33	0.00	2.75	0.09	0.48	0.11
Q13093	Platelet-activating factor acetylhydrolase	PLA2G7	50 kDa	1.33	0.00	1.50	0.13	0.88	0.07
Q9Y5K6	CD2-associated protein	CD2AP	71 kDa	1.33	0.00	1.15	0.20	1.15	0.02
B5MBX2	Transcobalamin-2	TCN2	47 kDa	1.33	0.00	1.25	0.13	1.06	0.20
O96005	Cleft lip and palate transmembrane protein 1	CLPTM1	76 kDa	1.33	0.00	2.65	0.02	0.50	0.00
E9PCI3	Asparagine synthetase	ASNS	62 kDa	1.33	0.00	0.40	0.00	3.31	0.00
P04792	Cluster of Heat shock protein beta-1	HSPB1	23 kDa	1.33	0.00	0.70	0.00	1.89	0.00
Q7L775	EPM2A-interacting protein 1	EPM2AIP1	70 kDa	1.33	0.00	1.55	0.06	0.85	0.01

Q7RTV0	PHD finger-like domain-containing protein 5A	PHF5A	12 kDa	1.33	0.00	0.90	0.00	1.47	0.00
Q9NX46	Poly(ADP-ribose) glycohydrolase ARH3	ADPRHL2	39 kDa	1.55	0.00	1.70	0.00	0.91	0.01
Q969E8	Pre-rRNA-processing protein TSR2 homolog	TSR2	21 kDa	1.38	0.00	2.50	0.08	0.55	0.11
Q9NXH8	Torsin-4A	TOR4A	47 kDa	1.38	0.00	1.10	0.50	1.25	0.02
P78345	Ribonuclease P protein subunit p38	RPP38	32 kDa	1.38	0.00	1.10	0.50	1.25	0.02
B4E2N0	Protein LZIC	LZIC	24 kDa	1.38	0.00	1.40	0.16	0.98	0.75
P18754	Cluster of Isoform 2 of Regulator of chromosome condensati	RCC1	48 kDa	1.38	0.00	0.75	0.13	1.83	0.00
E7EM93	Peroxisomal NADH pyrophosphatase NUDT12	NUDT12	50 kDa	1.38	0.00	1.05	0.50	1.31	0.00
Q9HA77	Cluster of Probable cysteine--tRNA ligase, mitochondrial	CARS2	62 kDa	1.38	0.00	1.25	0.13	1.10	0.06
H7BYY3	Mediator of RNA polymerase II transcription subunit 23	MED23	157 kDa	1.38	0.00	1.20	0.00	1.15	0.01
Q9UHD2	Serine/threonine-protein kinase TBK1	TBK1	84 kDa	1.43	0.00	1.30	0.20	1.10	0.16
Q5H9R7	Cluster of Isoform 5 of Serine/threonine-protein phosphatase	PPP6R3	98 kDa	1.43	0.00	0.85	0.20	1.68	0.00
O43237	Cluster of Cytoplasmic dynein 1 light intermediate chain 2	DYNC1LI2	54 kDa	1.43	0.00	0.95	0.50	1.50	0.00
O95881	Thioredoxin domain-containing protein 12	TXNDC12	19 kDa	1.43	0.00	1.25	0.13	1.14	0.02
Q9Y697	Cluster of Isoform Cytoplasmic of Cysteine desulfurase, mit	NFS1	44 kDa	1.43	0.00	2.75	0.02	0.52	0.00
O15031	Plexin-B2	PLXNB2	205 kDa	1.65	0.00	0.75	0.13	2.20	0.00
P13716	Isoform 2 of Delta-aminolevulinic acid dehydratase	ALAD	39 kDa	1.48	0.00	1.30	0.20	1.13	0.07
J3KQ34	COP9 signalosome complex subunit 7b	COPS7B	26 kDa	1.48	0.00	0.80	0.30	1.84	0.00
Q9Y3C7	Mediator of RNA polymerase II transcription subunit 31	MED31	16 kDa	1.67	0.00	2.24	0.01	0.74	0.00
E7EMK3	Flotillin-2	FLOT2	53 kDa	1.53	0.00	1.30	0.00	1.17	0.00
O95376	E3 ubiquitin-protein ligase ARIH2	ARIH2	58 kDa	1.58	0.00	1.40	0.30	1.13	0.54
Q96T23	Remodeling and spacing factor 1	RSF1	164 kDa	1.63	0.00	1.60	0.00	1.02	0.39
P07902	Cluster of Galactose-1-phosphate uridylyltransferase	GALT	43 kDa	1.68	0.00	2.50	0.04	0.67	0.00
P04075	Cluster of Fructose-bisphosphate aldolase A	ALDOA	39 kDa	1.68	0.00	0.20	0.00	8.38	0.00
Q86VP6	Cluster of Cullin-associated NEDD8-dissociated protein 1	CAND1	136 kDa	1.83	0.00	0.30	0.00	6.08	0.00
O00232	26S proteasome non-ATPase regulatory subunit 12	PSMD12	53 kDa	1.83	0.00	0.60	0.00	3.04	0.00
E9PGT6	COP9 signalosome complex subunit 8	COPS8	19 kDa	0.90	0.00	1.00	1.00	0.90	0.70
Q96SB4	Cluster of Isoform 1 of SRSF protein kinase 1	SRPK1	92 kDa	0.70	0.00	0.85	0.50	0.82	0.50
Q99460	Cluster of 26S proteasome non-ATPase regulatory subunit 1	PSMD1	106 kDa	0.90	0.00	0.60	0.16	1.50	0.20
P63104	Cluster of 14-3-3 protein zeta/delta	YWHAZ	28 kDa	1.00	1.00	0.35	0.05	2.86	0.05
F5H4C2	Ran-binding protein 3	RANBP3	47 kDa	1.00	1.00	0.70	0.20	1.43	0.20
Q8NCL4	Cluster of Polypeptide N-acetylgalactosaminyltransferase 6	GALNT6	71 kDa	0.80	0.00	1.05	0.80	0.76	0.34
G5E9W7	28S ribosomal protein S22, mitochondrial	MRPS22	37 kDa	0.90	0.00	2.05	0.15	0.44	0.14
F5GY50	Prostaglandin reductase 1	PTGR1	33 kDa	0.90	0.00	0.90	0.50	1.00	1.00
O43823	A-kinase anchor protein 8	AKAP8	76 kDa	0.90	0.00	1.00	1.00	0.90	0.50
O94907	Cluster of Dickkopf-related protein 1	DKK1	29 kDa	0.80	0.00	0.55	0.07	1.45	0.13
B4DZI8	Cluster of Coatomer protein complex, subunit beta 2 (Beta p	COPB2	99 kDa	0.80	0.00	0.55	0.07	1.45	0.13

Q6P2Q9	Cluster of Pre-mRNA-processing-splicing factor 8	PRPF8	274 kDa	0.70	0.00	0.55	0.07	1.27	0.20
P50914	60S ribosomal protein L14	RPL14	23 kDa	1.30	0.00	0.55	0.07	2.36	0.04
Q96P70	Importin-9	IPO9	116 kDa	1.00	1.00	0.55	0.07	1.82	0.07
Q9BRK5	Cluster of 45 kDa calcium-binding protein	SDF4	42 kDa	1.00	1.00	0.55	0.07	1.82	0.07
Q8N8S7	Protein enabled homolog	ENAH	67 kDa	0.90	0.00	1.20	0.30	0.75	0.20
Q8NDZ4	Deleted in autism protein 1	C3orf58	49 kDa	0.80	0.00	1.20	0.30	0.67	0.16
Q96LD4	Tripartite motif-containing protein 47	TRIM47	70 kDa	0.60	0.00	1.20	0.30	0.50	0.11
B7Z815	Ubiquitin carboxyl-terminal hydrolase	USP7	126 kDa	1.00	1.00	0.65	0.09	1.54	0.09
Q9NRR5	Cluster of Ubiquilin-4	UBQLN4	64 kDa	1.10	0.00	0.65	0.09	1.69	0.07
O43684	Mitotic checkpoint protein BUB3	BUB3	37 kDa	1.00	1.00	0.65	0.09	1.54	0.09
O76003	Glutaredoxin-3	GLRX3	37 kDa	1.00	1.00	0.65	0.09	1.54	0.09
P23193	Cluster of Transcription elongation factor A protein 1	TCEA1	34 kDa	1.00	1.00	0.65	0.09	1.54	0.09
P0DJI8	Cluster of Serum amyloid A-1 protein	SAA1	14 kDa	1.00	1.00	1.30	0.20	0.77	0.20
Q8NBL1	Protein O-glucosyltransferase 1	POGLUT1	46 kDa	1.30	0.00	1.30	0.20	1.00	1.00
Q96E11	Cluster of Ribosome-recycling factor, mitochondrial	MRRF	29 kDa	1.20	0.00	1.40	0.16	0.86	0.30
Q96QG7	Myotubularin-related protein 9	MTMR9	63 kDa	0.70	0.00	1.40	0.16	0.50	0.09
P09622	Cluster of Dihydrolipoyl dehydrogenase, mitochondrial	DLD	54 kDa	0.90	0.00	0.75	0.13	1.20	0.20
Q9UKD2	mRNA turnover protein 4 homolog	MRTO4	28 kDa	0.70	0.00	0.75	0.13	0.93	0.50
J3KNW4	Four and a half LIM domains protein 2	FHL2	44 kDa	1.10	0.00	0.75	0.13	1.47	0.09
Q92797	Symplekin	SYMPK	141 kDa	0.90	0.00	0.75	0.13	1.20	0.20
G8JLA8	Cluster of Transforming growth factor-beta-induced protein	TGFBI	75 kDa	1.00	1.00	1.60	0.11	0.63	0.11
Q66K14	TBC1 domain family member 9B	TBC1D9B	141 kDa	0.70	0.00	1.60	0.11	0.44	0.07
Q2TAY7	Cluster of WD40 repeat-containing protein SMU1	SMU1	58 kDa	1.60	0.00	0.85	0.20	1.88	0.04
B4DGP8	Cluster of Calnexin	CANX	72 kDa	1.00	1.00	0.85	0.20	1.18	0.20
F8W8Q7	Golgin subfamily A member 4	GOLGA4	262 kDa	1.40	0.00	0.85	0.20	1.65	0.06
Q641Q3	Meteorin-like protein	METRNL	34 kDa	0.80	0.00	0.85	0.20	0.94	0.50
Q8N3U4	Isoform 2 of Cohesin subunit SA-2	STAG2	146 kDa	0.80	0.00	0.85	0.20	0.94	0.50
Q9HCU4	Cadherin EGF LAG seven-pass G-type receptor 2	CELSR2	317 kDa	0.80	0.00	1.70	0.09	0.47	0.07
P13807	Cluster of Glycogen [starch] synthase, muscle	GYS1	84 kDa	1.20	0.00	0.95	0.50	1.26	0.13
H7C0P6	Cluster of Mitogen-activated protein kinase kinase kinase ki	MAP4K4	121 kDa	1.00	1.00	0.95	0.50	1.05	0.50
Q13287	N-myc-interactor	NMI	35 kDa	0.90	0.00	0.95	0.50	0.95	0.50
E7EP23	Cluster of Elongator complex protein 2	ELP2	100 kDa	0.70	0.00	0.95	0.50	0.74	0.13
E9PRJ3	Cluster of CD151 antigen (Fragment)	CD151	20 kDa	0.90	0.00	0.95	0.50	0.95	0.50
Q6DKI1	60S ribosomal protein L7-like 1	RPL7L1	29 kDa	1.00	1.00	2.00	0.06	0.50	0.06
E9PCR4	Anaphase-promoting complex subunit 4	ANAPC4	92 kDa	0.90	0.00	2.00	0.06	0.45	0.06
P48059	Cluster of Isoform 5 of LIM and senescent cell antigen-like-c	LIMS1	42 kDa	1.10	0.00	1.05	0.50	1.05	0.50
P54920	Alpha-soluble NSF attachment protein	NAPA	33 kDa	1.10	0.00	1.05	0.50	1.05	0.50

Q9UKN8	General transcription factor 3C polypeptide 4	GTF3C4	92 kDa	1.00	1.00	1.05	0.50	0.95	0.50
B7ZKM8	Protein transport protein Sec24B	SEC24B	140 kDa	0.90	0.00	1.05	0.50	0.86	0.20
H0Y3M3	Cluster of Echinoderm microtubule-associated protein-like 3	EML3	98 kDa	0.80	0.00	1.05	0.50	0.76	0.13
P48200	Iron-responsive element-binding protein 2	IREB2	105 kDa	0.50	0.00	1.05	0.50	0.48	0.06
P62834	Cluster of Ras-related protein Rap-1A	RAP1A	21 kDa	0.40	0.00	1.05	0.50	0.38	0.05
Q07960	Rho GTPase-activating protein 1	ARHGAP1	50 kDa	0.30	0.00	1.05	0.50	0.29	0.04
F5H740	Voltage-dependent anion-selective channel protein 3	VDAC3	31 kDa	1.10	0.00	1.05	0.50	1.05	0.50
O76071	Probable cytosolic iron-sulfur protein assembly protein CIA(	CIAO1	38 kDa	1.00	1.00	1.05	0.50	0.95	0.50
O94906	Pre-mRNA-processing factor 6	PRPF6	107 kDa	1.00	1.00	1.05	0.50	0.95	0.50
Q96QR8	Cluster of Transcriptional activator protein Pur-beta	PURB	33 kDa	0.90	0.00	1.05	0.50	0.86	0.20
Q9H6F5	Coiled-coil domain-containing protein 86	CCDC86	40 kDa	0.90	0.00	1.05	0.50	0.86	0.20
Q9NVP1	Cluster of ATP-dependent RNA helicase DDX18	DDX18	75 kDa	1.60	0.00	1.15	0.20	1.39	0.07
Q04206	Cluster of Transcription factor p65	RELA	60 kDa	0.90	0.00	1.15	0.20	0.78	0.13
Q14676	Cluster of Mediator of DNA damage checkpoint protein 1	MDC1	227 kDa	0.90	0.00	1.15	0.20	0.78	0.13
Q15269	Cluster of Periodic tryptophan protein 2 homolog	PWP2	102 kDa	0.90	0.00	1.15	0.20	0.78	0.13
Q9H0A0	Cluster of N-acetyltransferase 10	NAT10	116 kDa	0.80	0.00	1.15	0.20	0.70	0.09
Q96N67	Cluster of Dedicator of cytokinesis protein 7	DOCK7	243 kDa	1.10	0.00	1.15	0.20	0.96	0.50
P49711	Cluster of Transcriptional repressor CTCF	CTCF	83 kDa	1.00	1.00	1.15	0.20	0.87	0.20
Q9H9E3	Cluster of Conserved oligomeric Golgi complex subunit 4	COG4	89 kDa	0.90	0.00	1.25	0.13	0.72	0.09
Q9UK59	Lariat debranching enzyme	DBR1	62 kDa	0.90	0.00	1.25	0.13	0.72	0.09
Q86SZ2	Trafficking protein particle complex subunit 6B	TRAPPC6B	18 kDa	0.90	0.00	1.25	0.13	0.72	0.09
C9JAB2	Cluster of Serine/arginine-rich-splicing factor 7	SRSF7	27 kDa	0.90	0.00	1.25	0.13	0.72	0.09
O00625	Pirin	PIR	32 kDa	0.80	0.00	1.25	0.13	0.64	0.07
O43909	Exostosin-like 3	EXTL3	105 kDa	0.80	0.00	1.25	0.13	0.64	0.07
O75792	Ribonuclease H2 subunit A	RNASEH2A	33 kDa	1.10	0.00	1.35	0.09	0.81	0.13
Q96QZ7	Membrane-associated guanylate kinase, WW and PDZ doma	MAGI1	165 kDa	0.90	0.00	1.35	0.09	0.67	0.07
O75947	ATP synthase subunit d, mitochondrial	ATP5H	18 kDa	0.90	0.00	1.35	0.09	0.67	0.07
Q8N5K1	CDGSH iron-sulfur domain-containing protein 2	CISD2	15 kDa	0.80	0.00	1.35	0.09	0.59	0.06
O43665	Regulator of G-protein signaling 10	RGS10	20 kDa	0.80	0.00	1.35	0.09	0.59	0.06
Q07812	Apoptosis regulator BAX	BAX	21 kDa	1.10	0.00	1.35	0.09	0.81	0.13
Q7Z7A3	Cytoplasmic tRNA 2-thiolation protein 1	CTU1	36 kDa	1.10	0.00	1.35	0.09	0.81	0.13
Q9NXV6	CDKN2A-interacting protein	CDKN2AIP	61 kDa	0.60	0.00	1.35	0.09	0.44	0.04
O00116	Alkyldihydroxyacetonephosphate synthase, peroxisomal	AGPS	73 kDa	0.90	0.00	1.45	0.07	0.62	0.06
G3V1U8	CDK-activating kinase assembly factor MAT1	MNAT1	31 kDa	0.80	0.00	1.45	0.07	0.55	0.05
Q16762	Thiosulfate sulfurtransferase	TST	33 kDa	0.80	0.00	1.55	0.06	0.52	0.04
P41240	Tyrosine-protein kinase CSK	CSK	51 kDa	1.10	0.00	1.65	0.05	0.67	0.06
Q5NDL2	EGF domain-specific O-linked N-acetylglucosamine transfer	EOGT	62 kDa	0.90	0.00	1.65	0.05	0.55	0.04

E5RK00	Dynactin subunit 6	DCTN6	18 kDa	1.20	0.00	1.65	0.05	0.73	0.07
Q8WXI9	Transcriptional repressor p66-beta	GATAD2B	65 kDa	0.90	0.00	1.75	0.04	0.51	0.04
Q9NQZ2	Something about silencing protein 10	UTP3	55 kDa	1.10	0.00	1.75	0.04	0.63	0.05
H7BZW1	Cluster of Uncharacterized protein (Fragment)		34 kDa	1.20	0.00	2.05	0.03	0.59	0.04
D6RHG7	Serine incorporator 5	SERINC5	51 kDa	0.95	0.50	1.16	0.20	0.82	0.00
P14618	Cluster of Isoform 3 of Pyruvate kinase isozymes M1/M2	PKM	56 kDa	0.10	0.00	0.10	0.00	1.00	1.00
P11586	Cluster of C-1-tetrahydrofolate synthase, cytoplasmic	MTHFD1	102 kDa	1.10	0.00	0.50	0.00	2.20	0.00
Q14118	Dystroglycan	DAG1	97 kDa	0.80	0.00	0.50	0.00	1.60	0.00
Q9UBS4	Cluster of DnaJ homolog subfamily B member 11	DNAJB11	41 kDa	0.80	0.00	0.50	0.00	1.60	0.00
E7EP74	Cluster of Golgin subfamily B member 1	GOLGB1	377 kDa	0.50	0.00	0.50	0.00	1.00	1.00
P26447	Protein S100-A4	S100A4	12 kDa	1.20	0.00	0.60	0.00	2.00	0.00
Q9Y3I0	tRNA-splicing ligase RtcB homolog	RTCB	55 kDa	1.10	0.00	0.60	0.00	1.83	0.00
E9PLM6	Midkine	MDK	17 kDa	1.10	0.00	0.60	0.00	1.83	0.00
H0Y7A7	Calmodulin (Fragment)	CALM2	21 kDa	1.10	0.00	0.60	0.00	1.83	0.00
P49189	4-trimethylaminobutyraldehyde dehydrogenase	ALDH9A1	54 kDa	1.10	0.00	0.60	0.00	1.83	0.00
Q14683	Structural maintenance of chromosomes protein 1A	SMC1A	143 kDa	0.90	0.00	0.60	0.00	1.50	0.00
Q14444	Cluster of Caprin-1	CAPRIN1	78 kDa	0.90	0.00	0.60	0.00	1.50	0.00
Q5SSJ5	Cluster of Heterochromatin protein 1-binding protein 3	HP1BP3	61 kDa	0.90	0.00	0.60	0.00	1.50	0.00
B4DVY1	Eukaryotic translation initiation factor 3 subunit D	EIF3D	58 kDa	0.90	0.00	0.60	0.00	1.50	0.00
Q9UNH7	Cluster of Sorting nexin-6	SNX6	47 kDa	0.80	0.00	0.60	0.00	1.33	0.00
O14776	Cluster of Transcription elongation regulator 1	TCERG1	124 kDa	0.80	0.00	0.60	0.00	1.33	0.00
O15143	Actin-related protein 2/3 complex subunit 1B	ARPC1B	41 kDa	0.80	0.00	0.60	0.00	1.33	0.00
P31947	14-3-3 protein sigma	SFN	28 kDa	0.80	0.00	0.60	0.00	1.33	0.00
P62750	Cluster of 60S ribosomal protein L23a	RPL23A	18 kDa	0.70	0.00	0.60	0.00	1.17	0.00
P54727	Cluster of UV excision repair protein RAD23 homolog B	RAD23B	43 kDa	1.20	0.00	0.70	0.00	1.71	0.00
P26006	Cluster of Integrin alpha-3	ITGA3	117 kDa	1.10	0.00	0.70	0.00	1.57	0.00
Q9UHB9	Signal recognition particle 68 kDa protein	SRP68	71 kDa	0.90	0.00	0.70	0.00	1.29	0.00
Q9Y4E8	Cluster of Ubiquitin carboxyl-terminal hydrolase 15	USP15	112 kDa	0.90	0.00	0.70	0.00	1.29	0.00
C9JFE4	Cluster of COP9 signalosome complex subunit 1	GPS1	53 kDa	0.90	0.00	0.70	0.00	1.29	0.00
E9PHY5	Cluster of Band 4.1-like protein 2	EPB41L2	104 kDa	0.90	0.00	0.70	0.00	1.29	0.00
H0Y930	Extracellular matrix protein FRAS1 (Fragment)	FRAS1	250 kDa	0.90	0.00	0.70	0.00	1.29	0.00
H0YLA4	Sorbitol dehydrogenase	SORD	36 kDa	0.90	0.00	0.70	0.00	1.29	0.00
Q07021	Complement component 1 Q subcomponent-binding protein,	C1QBP	31 kDa	0.90	0.00	0.70	0.00	1.29	0.00
P01034	Cystatin-C	CST3	16 kDa	0.80	0.00	0.70	0.00	1.14	0.00
P13521	Secretogranin-2	SCG2	71 kDa	0.80	0.00	0.70	0.00	1.14	0.00
Q9Y2Z0	Suppressor of G2 allele of SKP1 homolog	SUGT1	41 kDa	1.30	0.00	0.80	0.00	1.63	0.00
P36915	Guanine nucleotide-binding protein-like 1	GNL1	69 kDa	0.90	0.00	0.80	0.00	1.13	0.00

P31689	DnaJ homolog subfamily A member 1	DNAJA1	45 kDa	0.70	0.00	0.80	0.00	0.88	0.00
P62851	40S ribosomal protein S25	RPS25	14 kDa	1.30	0.00	0.90	0.00	1.44	0.00
P17480	Cluster of Nucleolar transcription factor 1	UBTF	89 kDa	1.20	0.00	0.90	0.00	1.33	0.00
Q99961	Cluster of Endophilin-A2	SH3GL1	41 kDa	1.00	1.00	0.90	0.00	1.11	0.00
Q9BWJ5	Splicing factor 3B subunit 5	SF3B5	10 kDa	1.00	1.00	0.90	0.00	1.11	0.00
Q9BZK7	Cluster of F-box-like/WD repeat-containing protein TBL1XI	TBL1XR1	56 kDa	0.90	0.00	0.90	0.00	1.00	1.00
Q9H1E3	Nuclear ubiquitous casein and cyclin-dependent kinase subst	NUCKS1	27 kDa	0.90	0.00	0.90	0.00	1.00	1.00
Q9ULE6	Paladin	PALD1	97 kDa	0.90	0.00	0.90	0.00	1.00	1.00
Q9Y2W1	Thyroid hormone receptor-associated protein 3	THRAP3	109 kDa	0.90	0.00	0.90	0.00	1.00	1.00
Q13404	Ubiquitin-conjugating enzyme E2 variant 1	UBE2V1	16 kDa	0.80	0.00	0.90	0.00	0.89	0.00
Q9UBF2	Coatomer subunit gamma-2	COPG2	98 kDa	1.20	0.00	1.00	1.00	1.20	0.00
Q7Z2Z2	Elongation factor Tu GTP-binding domain-containing protei	EFTUD1	125 kDa	1.00	1.00	1.00	1.00	1.00	1.00
P11166	Solute carrier family 2, facilitated glucose transporter memb	SLC2A1	54 kDa	0.90	0.00	1.00	1.00	0.90	0.00
P11172	Cluster of Uridine 5'-monophosphate synthase	UMPS	52 kDa	0.90	0.00	1.00	1.00	0.90	0.00
P17858	Cluster of 6-phosphofructokinase, liver type	PFKL	85 kDa	0.90	0.00	1.00	1.00	0.90	0.00
P19174	1-phosphatidylinositol 4,5-bisphosphate phosphodiesterase g	PLCG1	149 kDa	0.90	0.00	1.00	1.00	0.90	0.00
P48507	Glutamate--cysteine ligase regulatory subunit	GCLM	31 kDa	0.80	0.00	1.00	1.00	0.80	0.00
Q9H3K6	Cluster of BolA-like protein 2	BOLA2	10 kDa	0.80	0.00	1.00	1.00	0.80	0.00
Q5TDH0	Cluster of Protein DDI1 homolog 2	DDI2	45 kDa	0.80	0.00	1.00	1.00	0.80	0.00
B4E2W0	Trifunctional enzyme subunit beta, mitochondrial	HADHB	49 kDa	0.70	0.00	1.00	1.00	0.70	0.00
F5H2U2	Cluster of Serine/threonine-protein kinase PRP4 homolog	PRPF4B	115 kDa	0.50	0.00	1.00	1.00	0.50	0.00
P21583	Kit ligand	KITLG	31 kDa	1.20	0.00	1.10	0.00	1.09	0.00
B7Z1E5	Cell differentiation protein RCD1 homolog	RQCD1	37 kDa	0.90	0.00	1.10	0.00	0.82	0.00
C9JGI3	Thymidine phosphorylase (Fragment)	TYMP	46 kDa	0.90	0.00	1.10	0.00	0.82	0.00
P00387	Cluster of Isoform 3 of NADH-cytochrome b5 reductase 3	CYB5R3	38 kDa	1.20	0.00	1.20	0.00	1.00	1.00
P45984	Cluster of Isoform Beta-1 of Mitogen-activated protein kinas	MAPK9	44 kDa	1.10	0.00	1.20	0.00	0.92	0.00
F8W8S3	Cluster of Transducin-like enhancer protein 3	TLE3	83 kDa	1.10	0.00	1.20	0.00	0.92	0.00
Q9BRA2	Cluster of Thioredoxin domain-containing protein 17	TXNDC17	14 kDa	0.90	0.00	1.20	0.00	0.75	0.00
Q9H3N1	Thioredoxin-related transmembrane protein 1	TMX1	32 kDa	0.90	0.00	1.20	0.00	0.75	0.00
Q9H410	Kinetochore-associated protein DSN1 homolog	DSN1	40 kDa	0.90	0.00	1.20	0.00	0.75	0.00
Q7LGC8	Carbohydrate sulfotransferase 3	CHST3	55 kDa	0.80	0.00	1.20	0.00	0.67	0.00
Q6ZMI0	Cluster of Protein phosphatase 1 regulatory subunit 21	PPP1R21	88 kDa	0.80	0.00	1.20	0.00	0.67	0.00
P34096	Ribonuclease 4	RNASE4	17 kDa	0.70	0.00	1.20	0.00	0.58	0.00
C9J2Q2	ATP synthase mitochondrial F1 complex assembly factor 2 (	ATPAF2	23 kDa	1.10	0.00	1.30	0.00	0.85	0.00
Q99714	3-hydroxyacyl-CoA dehydrogenase type-2	HSD17B10	27 kDa	0.90	0.00	1.30	0.00	0.69	0.00
P48745	Protein NOV homolog	NOV	39 kDa	0.80	0.00	1.30	0.00	0.62	0.00
Q15386	Ubiquitin-protein ligase E3C	UBE3C	124 kDa	0.80	0.00	1.30	0.00	0.62	0.00

Q8IWZ8	SURP and G-patch domain-containing protein 1	SUGP1	72 kDa	0.60	0.00	1.30	0.00	0.46	0.00
J3KR58	Selenide, water dikinase 2	SEPHS2	51 kDa	1.40	0.00	1.40	0.00	1.00	1.00
Q14690	Protein RRP5 homolog	PDCD11	209 kDa	1.00	1.00	1.40	0.00	0.71	0.00
B3KQV7	Equilibrative nucleoside transporter 1	SLC29A1	59 kDa	0.80	0.00	1.50	0.00	0.53	0.00
Q9NX70	Mediator of RNA polymerase II transcription subunit 29	MED29	21 kDa	0.50	0.00	1.50	0.00	0.33	0.00
Q5JTV8	Torsin-1A-interacting protein 1	TOR1AIP1	66 kDa	0.90	0.00	1.80	0.00	0.50	0.00
B4DP36	Calcium-binding and coiled-coil domain-containing protein [illegible]	CALCOCO2	55 kDa	0.70	0.00	2.00	0.00	0.35	0.00
E9PC90	Cluster of G2/mitotic-specific cyclin-B1 (Fragment)	CCNB1	45 kDa	0.60	0.00	2.00	0.00	0.30	0.00
G8JLI5	WD repeat domain phosphoinositide-interacting protein 4	WDR45	38 kDa	1.00	1.00	2.30	0.00	0.43	0.00

Supplementary Table S3. The differntially expressed proteins between MT and KO cells.

Accession Number	Protein subgroup name	Gene symbol	Molecular Weight	FC (MT/WT)	P-value (MT/WT)
F8VPG7	Phosphatidylinositol-binding clathrin assembly protein	PICALM	70 kDa	0.50	0.01
J3KQQ3	Zinc finger and BTB domain-containing protein 7B	ZBTB7B	62 kDa	0.43	0.00
E9PB30	Anoctamin	ANO6	104 kDa	4.38	0.02
P11142	Cluster of Heat shock cognate 71 kDa protein	HSPA8	71 kDa	0.60	0.02
Q504X1	ARHGAP12 protein	ARHGAP12	96 kDa	0.60	0.02
Q05639	Elongation factor 1-alpha 2	EEF1A2	50 kDa	0.63	0.03
Q15365	Cluster of Poly(rC)-binding protein 1	PCBP1	37 kDa	0.45	0.00
P53618	Coatomer subunit beta	COPB1	107 kDa	0.50	0.01
E9PDM8	Cluster of Protein transport protein Sec24D	SEC24D	75 kDa	2.15	0.03
Q9NQZ5	StAR-related lipid transfer protein 7, mitochondrial	STARD7	43 kDa	4.08	0.01
P25398	40S ribosomal protein S12	RPS12	15 kDa	1.73	0.05
P06748	Cluster of Nucleophosmin	NPM1	33 kDa	1.73	0.04
P51784	Ubiquitin carboxyl-terminal hydrolase 11	USP11	110 kDa	1.80	0.03
P26639	Cluster of Threonine--tRNA ligase, cytoplasmic	TARS	83 kDa	0.53	0.00
A6NGP5	Cluster of Hematological and neurological-expressed 1-	HN1L	19 kDa	1.95	0.03
Q6IBS0	Cluster of Twinfilin-2	TWF2	40 kDa	0.55	0.01
P55809	Cluster of Succinyl-CoA:3-ketoacid coenzyme A transferase 1, mitochondrial	OXCT1	56 kDa	0.55	0.01
Q02818	Cluster of Nucleobindin-1	NUCB1	54 kDa	0.50	0.00
H7C463	Cluster of Mitochondrial inner membrane protein	IMMT	68 kDa	0.50	0.00
G5E9Y2	Cluster of X-prolyl aminopeptidase (Aminopeptidase P) 1, soluble, isoform CRA_b	XPNPEP1	75 kDa	2.63	0.01
Q6ZMP0	Thrombospondin type-1 domain-containing protein 4	THSD4	112 kDa	1.78	0.03
Q92945	Far upstream element-binding protein 2	KHSRP	73 kDa	2.20	0.01
Q9HCD5	Nuclear receptor coactivator 5	NCOA5	66 kDa	2.18	0.01
Q9BZH6	WD repeat-containing protein 11	WDR11	137 kDa	1.78	0.02
Q9Y248	DNA replication complex GINS protein PSF2	GINS2	21 kDa	0.48	0.00
P04004	Vitronectin	VTN	54 kDa	0.63	0.01
P49720	Proteasome subunit beta type-3	PSMB3	23 kDa	0.63	0.01
P17987	Cluster of T-complex protein 1 subunit alpha	TCP1	60 kDa	0.63	0.01
Q16254	Transcription factor E2F4	E2F4	44 kDa	1.60	0.03
G3XAH6	Cluster of Poly(A) polymerase alpha	PAPOLA	81 kDa	1.73	0.02
Q9NW82	WD repeat-containing protein 70	WDR70	73 kDa	1.95	0.01
P13667	Cluster of Protein disulfide-isomerase A4	PDIA4	73 kDa	0.53	0.00
Q16531	Cluster of DNA damage-binding protein 1	DDB1	127 kDa	0.53	0.00
B4E1K7	Cluster of Stomatin-like protein 2	STOML2	33 kDa	0.53	0.00
P19404	NADH dehydrogenase [ubiquinone] flavoprotein 2,	NDUFV2	27 kDa	0.53	0.00
Q7L2H7	Eukaryotic translation initiation factor 3 subunit M	EIF3M	43 kDa	0.53	0.00
Q8N1G2	Cap-specific mRNA (nucleoside-2'-O-)-	FTSJD2	95 kDa	3.75	0.00
Q92636	Protein FAN	NSMAF	104 kDa	1.70	0.02
E7ETA6	Pericentriolar material 1 protein	PCM1	228 kDa	2.35	0.01
G3V0F8	Cluster of Protein tyrosine phosphatase, non-receptor type 6, isoform CRA_e	PTPN6	70 kDa	1.83	0.01
P61586	Cluster of Transforming protein RhoA	RHOA	22 kDa	1.60	0.02
Q9Y3D6	Mitochondrial fission 1 protein	FIS1	17 kDa	0.58	0.00
Q5VTL8	Pre-mRNA-splicing factor 38B	PRPF38B	64 kDa	0.58	0.00
O75822	Cluster of Eukaryotic translation initiation factor 3	EIF3J	29 kDa	0.35	0.00
P25789	Cluster of Proteasome subunit alpha type-4	PSMA4	29 kDa	0.50	0.00
P20618	Proteasome subunit beta type-1	PSMB1	26 kDa	0.50	0.00
P49366	Deoxyhypusine synthase	DHPS	41 kDa	0.50	0.00
Q7Z4H3	HD domain-containing protein 2	HDDC2	23 kDa	1.73	0.01
P82932	28S ribosomal protein S6, mitochondrial	MRPS6	14 kDa	2.30	0.01
H0YGR4	Oligoribonuclease, mitochondrial (Fragment)	REXO2	22 kDa	1.63	0.02

P51665	Cluster of 26S proteasome non-ATPase regulatory	PSMD7	37 kDa	1.63	0.02
E9PH42	Receptor-type tyrosine-protein phosphatase U	PTPRU	162 kDa	0.63	0.00
P23246	Splicing factor, proline- and glutamine-rich	SFPQ	76 kDa	0.63	0.00
Q8TF42	Ubiquitin-associated and SH3 domain-containing protein	UBASH3B	73 kDa	0.63	0.00
O14964	Hepatocyte growth factor-regulated tyrosine kinase	HGS	86 kDa	0.63	0.00
P46100	Transcriptional regulator ATRX	ATRX	283 kDa	0.63	0.00
P07900	Cluster of Heat shock protein HSP 90-alpha	HSP90AA1	85 kDa	1.60	0.02
A8MZF9	Cluster of Developmentally-regulated GTP-binding	DRG2	38 kDa	1.83	0.01
Q9H4L7	Cluster of SWI/SNF-related matrix-associated actin-dependent regulator of chromatin subfamily A	SMARCAD1	117 kDa	3.15	0.00
Q9NX08	COMM domain-containing protein 8	COMMD8	21 kDa	2.05	0.01
Q8IWE4	DCN1-like protein 3	DCUN1D3	34 kDa	1.85	0.01
P49959	Cluster of Isoform 2 of Double-strand break repair	MRE11A	78 kDa	1.63	0.01
Q9UNF0	Protein kinase C and casein kinase substrate in neurons	PACSIN2	56 kDa	0.60	0.00
C9JJP5	Protein TFG (Fragment)	TFG	20 kDa	0.60	0.00
Q15853	Upstream stimulatory factor 2	USF2	37 kDa	0.60	0.00
P45973	Chromobox protein homolog 5	CBX5	22 kDa	0.60	0.00
Q93052	Lipoma-preferred partner	LPP	66 kDa	0.60	0.00
O75116	Rho-associated protein kinase 2	ROCK2	161 kDa	0.60	0.00
P05783	Cluster of Keratin, type I cytoskeletal 18	KRT18	48 kDa	0.60	0.00
Q9BV38	WD repeat-containing protein 18	WDR18	47 kDa	0.60	0.00
Q15738	Sterol-4-alpha-carboxylate 3-dehydrogenase,	NSDHL	42 kDa	0.60	0.00
J3KPM9	Signal transducer and activator of transcription 1-	STAT1	83 kDa	1.60	0.01
C9J180	Ubiquitin-conjugating enzyme E2 E2 (Fragment)	UBE2E2	18 kDa	4.53	0.00
E9PEI9	Lanosterol synthase	LSS	82 kDa	0.38	0.00
Q69YN2	CWF19-like protein 1	CWF19L1	61 kDa	0.45	0.00
P62314	Small nuclear ribonucleoprotein Sm D1	SNRPD1	13 kDa	0.45	0.00
Q6P158	Putative ATP-dependent RNA helicase DHX57	DHX57	156 kDa	0.45	0.00
Q9Y3C8	Ubiquitin-fold modifier-conjugating enzyme 1	UFC1	19 kDa	1.65	0.01
P78318	Immunoglobulin-binding protein 1	IGBP1	39 kDa	1.60	0.01
Q9H9S4	Calcium-binding protein 39-like	CAB39L	39 kDa	1.68	0.01
Q9Y2S0	DNA-directed RNA polymerases I and III subunit	POLR1D	15 kDa	1.73	0.01
P49327	Fatty acid synthase	FASN	273 kDa	0.43	0.00
P15531	Cluster of Nucleoside diphosphate kinase A	NME1	17 kDa	0.43	0.00
H0YCJ8	Blood group Rh(CE) polypeptide (Fragment)	RHCE	8 kDa	1.78	0.00
B5MCF9	Cluster of Pescadillo homolog	PES1	66 kDa	2.05	0.00
Q8IWV7	Cluster of E3 ubiquitin-protein ligase UBR1	UBR1	200 kDa	1.60	0.01
J3KPP7	Beta-arrestin-1	ARRB1	47 kDa	1.88	0.00
Q9Y383	Cluster of Putative RNA-binding protein Luc7-like 2	LUC7L2	47 kDa	1.70	0.00
H0YES5	HBS1-like protein (Fragment)	HBS1L	19 kDa	1.93	0.00
P49757	Protein numb homolog	NUMB	71 kDa	0.48	0.00
P06400	Retinoblastoma-associated protein	RB1	106 kDa	1.65	0.00
G5E9W3	Cleavage and polyadenylation specific factor 3, 73kDa,	CPSF3	73 kDa	1.65	0.00
O15230	Laminin subunit alpha-5	LAMA5	400 kDa	0.55	0.00
F5H0L8	SEC23-interacting protein	SEC23IP	90 kDa	0.55	0.00
Q9Y2S2	Lambda-crystallin homolog	CRYL1	35 kDa	1.68	0.00
O43688	Isoform 2 of Lipid phosphate phosphohydrolase 2	PPAP2C	34 kDa	2.85	0.00
P62249	40S ribosomal protein S16	RPS16	16 kDa	1.73	0.00
O75143	Cluster of Isoform 3 of Autophagy-related protein 13	ATG13	44 kDa	1.79	0.00
Q15785	Mitochondrial import receptor subunit TOM34	TOMM34	35 kDa	0.53	0.00
Q9P287	Cluster of BRCA2 and CDKN1A-interacting protein	BCCIP	36 kDa	1.63	0.00
Q6PCB0	von Willebrand factor A domain-containing protein 1	VWA1	47 kDa	1.60	0.00
Q9Y639	Cluster of Neuroplastin	NPTN	44 kDa	1.60	0.00
Q9UNS2	Cluster of Isoform 2 of COP9 signalosome complex	COPS3	46 kDa	1.60	0.00
P06280	Alpha-galactosidase A	GLA	49 kDa	1.60	0.00
O60911	Cathepsin L2	CTSL2	37 kDa	2.18	0.00

P00505	Aspartate aminotransferase, mitochondrial	GOT2	48 kDa	0.58	0.00
Q9NWU2	Glucose-induced degradation protein 8 homolog	GID8	27 kDa	0.58	0.00
E9PHK0	Tetranectin	CLEC3B	18 kDa	1.73	0.00
P49821	NADH dehydrogenase [ubiquinone] flavoprotein 1,	NDUFV1	51 kDa	1.90	0.00
Q96T37	Cluster of Isoform 3 of Putative RNA-binding protein 15	RBM15	106 kDa	1.78	0.00
Q9Y5Z4	Heme-binding protein 2	HEBP2	23 kDa	1.78	0.00
Q99543	DnaJ homolog subfamily C member 2	DNAJC2	72 kDa	1.70	0.00
Q9UMS4	Cluster of Pre-mRNA-processing factor 19	PRPF19	55 kDa	0.63	0.00
F5H025	Cluster of Neural cell adhesion molecule L1	L1CAM	140 kDa	0.63	0.00
Q02790	Cluster of Peptidyl-prolyl cis-trans isomerase FKBP4	FKBP4	52 kDa	0.63	0.00
Q9BT78	COP9 signalosome complex subunit 4	COPS4	46 kDa	0.63	0.00
B4DTU4	Cluster of DNA ligase	LIG1	98 kDa	0.63	0.00
P07996	Cluster of Thrombospondin-1	THBS1	129 kDa	0.63	0.00
Q9BSG5	Isoform 2 of Retbindin	RTBDN	28 kDa	1.90	0.00
Q9H939	Proline-serine-threonine phosphatase-interacting protein	PSTPIP2	39 kDa	1.60	0.00
P41271	Isoform 2 of Neuroblastoma suppressor of	NBL1	23 kDa	1.60	0.00
E7EPK6	40S ribosomal protein S24	RPS24	32 kDa	1.60	0.00
B4DTG2	Cluster of Elongation factor 1-gamma	EEF1G	56 kDa	2.95	0.00
Q96SZ5	2-aminoethanethiol dioxygenase	ADO	30 kDa	1.83	0.00
Q9H1K1	Iron-sulfur cluster assembly enzyme ISCU,	ISCU	18 kDa	2.15	0.00
A8CTX8	Intersectin 1 long form variant 2	ITSN1	195 kDa	1.63	0.00
Q96HW7	Cluster of Integrator complex subunit 4	INTS4	108 kDa	1.68	0.00
Q01518	Cluster of Adenylyl cyclase-associated protein 1	CAP1	52 kDa	1.68	0.00
Q8NEB9	Phosphatidylinositol 3-kinase catalytic subunit type 3	PIK3C3	102 kDa	1.95	0.00
O15031	Plexin-B2	PLXNB2	205 kDa	1.65	0.00
Q9Y3C7	Mediator of RNA polymerase II transcription subunit 31	MED31	16 kDa	1.67	0.00
Q96T23	Remodeling and spacing factor 1	RSF1	164 kDa	1.63	0.00
P07902	Cluster of Galactose-1-phosphate uridylyltransferase	GALT	43 kDa	1.68	0.00
P04075	Cluster of Fructose-bisphosphate aldolase A	ALDOA	39 kDa	1.68	0.00
Q86VP6	Cluster of Cullin-associated NEDD8-dissociated protein	CAND1	136 kDa	1.83	0.00
O00232	26S proteasome non-ATPase regulatory subunit 12	PSMD12	53 kDa	1.83	0.00
Q96LD4	Tripartite motif-containing protein 47	TRIM47	70 kDa	0.60	0.00
Q2TAY7	Cluster of WD40 repeat-containing protein SMU1	SMU1	58 kDa	1.60	0.00
P48200	Iron-responsive element-binding protein 2	IREB2	105 kDa	0.50	0.00
P62834	Cluster of Ras-related protein Rap-1A	RAP1A	21 kDa	0.40	0.00
Q07960	Rho GTPase-activating protein 1	ARHGAP1	50 kDa	0.30	0.00
Q9NVP1	Cluster of ATP-dependent RNA helicase DDX18	DDX18	75 kDa	1.60	0.00
Q9NXV6	CDKN2A-interacting protein	CDKN2AIP	61 kDa	0.60	0.00
P14618	Cluster of Isoform 3 of Pyruvate kinase isozymes	PKM	56 kDa	0.10	0.00
E7EP74	Cluster of Golgin subfamily B member 1	GOLGB1	377 kDa	0.50	0.00
F5H2U2	Cluster of Serine/threonine-protein kinase PRP4	PRPF4B	115 kDa	0.50	0.00
Q8IWZ8	SURP and G-patch domain-containing protein 1	SUGP1	72 kDa	0.60	0.00
Q9NX70	Mediator of RNA polymerase II transcription subunit 29	MED29	21 kDa	0.50	0.00
E9PC90	Cluster of G2/mitotic-specific cyclin-B1 (Fragment)	CCNB1	45 kDa	0.60	0.00

Supplementary Table S4. The differentially expressed proteins between KO and WT cells.

Accession Number	Protein subgroup name	Gene symbol	Molecular Weight	FC (KO/WT)	P-value (KO/WT)
O94985	Isoform 2 of Calsyntenin-1	CLSTN1	109 kDa	0.30	0.00
F5GWY5	Podocalyxin	PODXL	59 kDa	1.70	0.00
J3KQQ3	Zinc finger and BTB domain-containing protein 7B	ZBTB7B	62 kDa	3.15	0.01
G3XAD8	Cluster of Stress-induced-phosphoprotein 1	STIP1	68 kDa	0.30	0.00
E9PB30	Anoctamin	ANO6	104 kDa	3.40	0.03
B5ME19	Eukaryotic translation initiation factor 3 subunit C	EIF3CL	105 kDa	0.40	0.00
Q86XP3	ATP-dependent RNA helicase DDX42	DDX42	103 kDa	0.50	0.00
F5H8J2	Cluster of Protein disulfide-isomerase	P4HB	51 kDa	0.25	0.04
Q08945	Cluster of FACT complex subunit SSRP1	SSRP1	81 kDa	0.35	0.05
P53396	Cluster of ATP-citrate synthase	ACLY	121 kDa	0.30	0.00
P28799	Granulins	GRN	64 kDa	0.50	0.00
P11142	Cluster of Heat shock cognate 71 kDa protein	HSPA8	71 kDa	0.25	0.04
P42285	Cluster of Superkiller viralicidic activity 2-like 2	SKIV2L2	118 kDa	0.60	0.00
P53618	Coatomer subunit beta	COPB1	107 kDa	0.40	0.00
P07355	Cluster of Isoform 2 of Annexin A2	ANXA2	40 kDa	0.20	0.00
Q16851	Cluster of UTP--glucose-1-phosphate uridylyltransferase	UGP2	57 kDa	0.60	0.00
O95834	Isoform 2 of Echinoderm microtubule-associated protein-like 2	EML2	86 kDa	0.60	0.00
P38919	Cluster of Eukaryotic initiation factor 4A-III	EIF4A3	47 kDa	0.30	0.00
E9PFM1	Cluster of Eukaryotic translation initiation factor 4 gamma 1	EIF4G1	176 kDa	0.50	0.00
Q9NQZ5	StAR-related lipid transfer protein 7, mitochondrial	STARD7	43 kDa	2.00	0.00
P26583	Cluster of High mobility group protein B2	HMGB2	24 kDa	0.40	0.00
P11047	Laminin subunit gamma-1	LAMC1	178 kDa	0.25	0.04
P10586	Cluster of Receptor-type tyrosine-protein phosphatase F	PTPRF	213 kDa	0.30	0.00
Q13217	DnaJ homolog subfamily C member 3	DNAJC3	58 kDa	0.60	0.00
F8VXB4	Cluster of Keratin, type II cytoskeletal 8	KRT8	57 kDa	0.20	0.00
B4DRN8	Cluster of Probable palmitoyltransferase ZDHHC20	ZDHHC20	34 kDa	2.30	0.05
P06396	Cluster of Isoform 2 of Gelsolin	GSN	81 kDa	0.35	0.05
P00488	Coagulation factor XIII A chain	F13A1	83 kDa	1.65	0.05
Q16181	Cluster of Septin-7	SEPT7	51 kDa	0.60	0.00
P07195	Cluster of L-lactate dehydrogenase B chain	LDHB	37 kDa	4.90	0.03
P06748	Cluster of Nucleophosmin	NPM1	33 kDa	0.35	0.05
Q9Y678	Cluster of Coatomer subunit gamma-1	COPG1	98 kDa	0.60	0.00
P26639	Cluster of Threonine--tRNA ligase, cytoplasmic	TARS	83 kDa	0.30	0.00
G5EA52	Cluster of Protein disulfide isomerase family A, member 3, isoform CRA_b	PDIA3	55 kDa	0.25	0.04
A6NGV6	Aryl hydrocarbon receptor nuclear translocator	ARNT	46 kDa	1.65	0.05
Q14980	Cluster of Isoform 2 of Nuclear mitotic apparatus protein 1	NUMA1	237 kDa	0.30	0.00
Q02818	Cluster of Nucleobindin-1	NUCB1	54 kDa	0.40	0.00
P12270	Cluster of Nucleoprotein TPR	TPR	267 kDa	0.30	0.00
Q8NBP7	Cluster of Proprotein convertase subtilisin/kexin type 9	PCSK9	74 kDa	0.35	0.05
Q14204	Cytoplasmic dynein 1 heavy chain 1	DYNC1H1	532 kDa	0.30	0.00
P22234	Cluster of Multifunctional protein ADE2	PAICS	47 kDa	0.35	0.05
Q9Y5X2	Sorting nexin-8	SNX8	53 kDa	2.25	0.03
P23528	Cofilin-1	CFL1	19 kDa	0.50	0.00
P68363	Cluster of Tubulin alpha-1B chain	TUBA1B	50 kDa	0.30	0.00
Q92973	Transportin-1	TNPO1	102 kDa	0.60	0.00
Q5T6F2	Cluster of Ubiquitin-associated protein 2	UBAP2	117 kDa	1.65	0.05
Q9HCE5	Methyltransferase-like protein 14	METTL14	52 kDa	1.65	0.05
O75643	U5 small nuclear ribonucleoprotein 200 kDa helicase	SNRNP200	245 kDa	0.30	0.00
O00186	Syntaxin-binding protein 3	STXBP3	68 kDa	3.40	0.03
P13639	Elongation factor 2	EEF2	95 kDa	0.25	0.04

O95707	Ribonuclease P protein subunit p29	POP4	25 kDa	1.85	0.04
P62333	Cluster of 26S protease regulatory subunit 10B	PSMC6	44 kDa	0.60	0.00
O43805	Sjoegren syndrome nuclear autoantigen 1	SSNA1	14 kDa	2.05	0.03
P32119	Cluster of Peroxiredoxin-2	PRDX2	22 kDa	0.40	0.00
Q9UBQ5	Eukaryotic translation initiation factor 3 subunit K	EIF3K	25 kDa	0.60	0.00
Q92945	Far upstream element-binding protein 2	KHSRP	73 kDa	0.50	0.00
P12081	Cluster of Histidine--tRNA ligase, cytoplasmic	HARS	57 kDa	0.25	0.04
Q9HA65	TBC1 domain family member 17	TBC1D17	73 kDa	2.15	0.03
O75874	Isocitrate dehydrogenase [NADP] cytoplasmic	IDH1	47 kDa	0.50	0.00
P14625	Cluster of Endoplasmin	HSP90B1	92 kDa	0.30	0.00
P78371	T-complex protein 1 subunit beta	CCT2	57 kDa	0.30	0.00
P40227	Cluster of T-complex protein 1 subunit zeta	CCT6A	58 kDa	0.40	0.00
Q9Y5B8	Nucleoside diphosphate kinase 7	NME7	42 kDa	1.65	0.05
Q2L7G6	Heterogeneous nuclear ribonucleoprotein R	HNRNPR	67 kDa	0.50	0.00
Q8IZH2	5'-3' exoribonuclease 1	XRN1	194 kDa	1.70	0.00
Q9H5N1	Rab GTPase-binding effector protein 2	RABEP2	64 kDa	1.65	0.05
P09382	Galectin-1	LGALS1	15 kDa	0.30	0.00
P08195	Cluster of Isoform 2 of 4F2 cell-surface antigen heavy chain	SLC3A2	58 kDa	0.50	0.00
Q16254	Transcription factor E2F4	E2F4	44 kDa	4.10	0.04
H0Y8C6	Importin-5 (Fragment)	IPO5	124 kDa	0.50	0.00
P52564	Cluster of Dual specificity mitogen-activated protein kinase kinase 6	MAP2K6	37 kDa	2.90	0.03
B4DII8	Cluster of Gamma-secretase C-terminal fragment 57	APP	85 kDa	0.40	0.00
P07858	Cathepsin B	CTSB	38 kDa	0.50	0.00
G8JLQ3	Biogenesis of lysosome-related organelles complex 1 subunit 1	BLOC1S1	9 kDa	1.60	0.00
P34932	Heat shock 70 kDa protein 4	HSPA4	94 kDa	0.35	0.05
Q9Y490	Cluster of Talin-1	TLN1	270 kDa	0.35	0.05
P38646	Cluster of Stress-70 protein, mitochondrial	HSPA9	74 kDa	0.35	0.05
B4E1F0	Plasma protease C1 inhibitor	SERPING1	56 kDa	1.75	0.04
F8VQZ7	Cluster of Methionine aminopeptidase	METAP2	53 kDa	0.60	0.00
P11216	Cluster of Glycogen phosphorylase, brain form	PYGB	97 kDa	0.20	0.00
P00558	Cluster of Phosphoglycerate kinase 1	PGK1	45 kDa	0.30	0.00
Q14697	Cluster of Neutral alpha-glucosidase AB	GANAB	107 kDa	0.30	0.00
O60271	Cluster of C-Jun-amino-terminal kinase-interacting protein 4	SPAG9	146 kDa	0.60	0.00
Q8N3E9	1-phosphatidylinositol 4,5-bisphosphate phosphodiesterase delta-3	PLCD3	89 kDa	3.55	0.04
P21333	Cluster of Filamin-A	FLNA	281 kDa	0.30	0.00
Q92900	Regulator of nonsense transcripts 1	UPF1	124 kDa	0.60	0.00
H3BQQ4	FAD-linked sulfhydryl oxidase ALR	GFER	15 kDa	1.70	0.00
J3QQZ1	Sodium channel protein type 4 subunit alpha	SCN4A	208 kDa	4.05	0.03
P46777	60S ribosomal protein L5	RPL5	34 kDa	0.50	0.00
Q13509	Cluster of Tubulin beta-3 chain	TUBB3	50 kDa	0.35	0.05
Q99879	Cluster of Histone H2B type 1-M	HIST1H2BM	14 kDa	0.25	0.04
P13667	Cluster of Protein disulfide-isomerase A4	PDIA4	73 kDa	0.25	0.04
Q16531	Cluster of DNA damage-binding protein 1	DDB1	127 kDa	0.35	0.05
Q7L2H7	Eukaryotic translation initiation factor 3 subunit M	EIF3M	43 kDa	0.10	0.00
Q13263	Transcription intermediary factor 1-beta	TRIM28	89 kDa	0.60	0.00
Q96G25	Mediator of RNA polymerase II transcription subunit 8	MED8	29 kDa	1.65	0.05
Q06830	Peroxiredoxin-1	PRDX1	22 kDa	0.35	0.05
I3L3I4	Cluster of Actin, cytoplasmic 2	ACTG1	42 kDa	0.20	0.00
H3BRY3	Coronin	CORO1A	43 kDa	1.70	0.00
Q92598	Cluster of Heat shock protein 105 kDa	HSPH1	97 kDa	0.30	0.00
Q92820	Gamma-glutamyl hydrolase	GGH	36 kDa	0.60	0.00

H0YFI1	Ragulator complex protein LAMTOR1 (Fragment)	LAMTOR1	9 kDa	2.20	0.00
E9PIL6	Cluster of Lysine-specific demethylase 2A	KDM2A	82 kDa	2.05	0.03
B4DRF9	Cyclic AMP-dependent transcription factor ATF-1	ATF1	15 kDa	2.05	0.03
Q06203	Amidophosphoribosyltransferase	PPAT	57 kDa	0.60	0.00
P29144	Cluster of Tripeptidyl-peptidase 2	TPP2	138 kDa	0.60	0.00
B1AMW1	CD58 antigen, (Lymphocyte function-associated antigen 3), isoform CRA_c	CD58	27 kDa	2.20	0.00
Q92636	Protein FAN	NSMAF	104 kDa	2.40	0.05
Q7L576	Cluster of Cytoplasmic FMR1-interacting protein 1	CYFIP1	145 kDa	0.60	0.00
Q9BXJ9	Cluster of N-alpha-acetyltransferase 15, NatA auxiliary subunit	NAA15	101 kDa	0.35	0.05
P30153	Cluster of Serine/threonine-protein phosphatase 2A 65 kDa regulatory subunit A alpha isoform	PPP2R1A	65 kDa	0.40	0.00
O60487	Myelin protein zero-like protein 2	MPZL2	24 kDa	1.80	0.00
Q9Y303	Isoform 3 of Putative N-acetylglucosamine-6-phosphate deacetylase	AMDHD2	64 kDa	1.75	0.04
Q14517	Cluster of Protocadherin Fat 1	FAT1	506 kDa	0.30	0.00
P28066	Cluster of Proteasome subunit alpha type-5	PSMA5	26 kDa	0.40	0.00
E7ETA6	Pericentriolar material 1 protein	PCM1	228 kDa	1.65	0.05
Q9NSD9	Phenylalanine--tRNA ligase beta subunit	FARSB	66 kDa	0.50	0.00
O43242	Cluster of 26S proteasome non-ATPase regulatory subunit 3	PSMD3	61 kDa	0.60	0.00
Q10570	Cleavage and polyadenylation specificity factor subunit 1	CPSF1	161 kDa	0.60	0.00
O60493	Sorting nexin-3	SNX3	19 kDa	2.20	0.00
O60506	Cluster of Isoform 3 of Heterogeneous nuclear ribonucleoprotein Q	SYNCRIP	63 kDa	0.25	0.04
D6RDX8	Tetraspanin-17	TSPAN17	34 kDa	1.65	0.05
P06213	Insulin receptor	INSR	156 kDa	2.05	0.03
Q15542	Transcription initiation factor TFIID subunit 5	TAF5	87 kDa	1.80	0.00
H7C5I8	Cluster of Down syndrome critical region protein 3 (Fragment)	DSCR3	9 kDa	3.85	0.03
Q9BXS4	Cluster of Transmembrane protein 59	TMEM59	36 kDa	1.65	0.05
P57735	Ras-related protein Rab-25	RAB25	23 kDa	2.15	0.03
Q9H1D9	DNA-directed RNA polymerase III subunit RPC6	POLR3F	36 kDa	1.75	0.04
Q9UHW5	Isoform 2 of GPN-loop GTPase 3	GPN3	34 kDa	1.70	0.00
P82932	28S ribosomal protein S6, mitochondrial	MRPS6	14 kDa	2.45	0.02
Q9BQI0	Allograft inflammatory factor 1-like	AIF1L	17 kDa	1.85	0.04
P46100	Transcriptional regulator ATRX	ATRX	283 kDa	2.55	0.02
J3KP07	Cluster of Growth arrest-specific protein 6	GAS6	80 kDa	0.60	0.00
P07900	Cluster of Heat shock protein HSP 90-alpha	HSP90AA1	85 kDa	8.25	0.05
O75063	Glycosaminoglycan xylosylkinase	FAM20B	46 kDa	1.80	0.00
P49591	Serine--tRNA ligase, cytoplasmic	SARS	59 kDa	0.60	0.00
P05556	Cluster of Integrin beta-1	ITGB1	88 kDa	0.50	0.00
Q01082	Cluster of Spectrin beta chain, non-erythrocytic 1	SPTBN1	275 kDa	0.30	0.00
P62701	Cluster of 40S ribosomal protein S4, X isoform	RPS4X	30 kDa	0.60	0.00
P01023	Cluster of Alpha-2-macroglobulin	A2M	163 kDa	0.35	0.05
B3KS98	Cluster of Eukaryotic translation initiation factor 3 subunit H	EIF3H	42 kDa	0.35	0.05
P08758	Cluster of Annexin A5	ANXA5	36 kDa	0.60	0.00
P52888	Thimet oligopeptidase	THOP1	79 kDa	0.60	0.00
Q14563	Semaphorin-3A	SEMA3A	89 kDa	0.60	0.00
B4DQH9	Ran-binding protein 10	RANBP10	65 kDa	2.65	0.02
P14314	Glucosidase 2 subunit beta	PRKCSH	59 kDa	0.50	0.00
P35579	Cluster of Myosin-9	MYH9	227 kDa	0.25	0.04
B3KPE7	Bcl-2-like protein 13	BCL2L13	35 kDa	1.85	0.04

B4DR87	Cluster of Procollagen-lysine,2-oxoglutarate 5-dioxygenase 1	PLOD1	88 kDa	0.30	0.00
P25786	Cluster of Proteasome subunit alpha type-1	PSMA1	30 kDa	0.50	0.00
P07339	Cluster of Cathepsin D	CTSD	45 kDa	0.10	0.00
Q10567	Cluster of Isoform C of AP-1 complex subunit beta-1	AP1B1	104 kDa	0.30	0.00
Q9Y224	Cluster of UPF0568 protein C14orf166	C14orf166	28 kDa	0.60	0.00
P47895	Cluster of Aldehyde dehydrogenase family 1 member A3	ALDH1A3	56 kDa	0.60	0.00
Q96CB8	Cluster of Integrator complex subunit 12	INTS12	49 kDa	2.10	0.00
Q56VL3	OCIA domain-containing protein 2	OCIAD2	17 kDa	2.20	0.00
C9JQV0	Cluster of Uncharacterized protein C7orf50 (Fragment)	C7orf50	22 kDa	1.70	0.00
P49959	Cluster of Isoform 2 of Double-strand break repair protein MRE11A	MRE11A	78 kDa	1.75	0.04
E9PFN5	Glutathione S-transferase kappa 1	GSTK1	22 kDa	1.65	0.05
Q5T4S7	Cluster of Isoform 2 of E3 ubiquitin-protein ligase UBR4	UBR4	576 kDa	0.50	0.00
P05783	Cluster of Keratin, type I cytoskeletal 18	KRT18	48 kDa	0.35	0.05
B4DKD1	Protein tweety homolog 2	TTYH2	56 kDa	1.80	0.00
P07602	Isoform Sap-mu-9 of Proactivator polypeptide	PSAP	58 kDa	0.10	0.00
Q9Y4L1	Cluster of Hypoxia up-regulated protein 1	HYOU1	111 kDa	0.40	0.00
O00468	Cluster of Agrin	AGRN	215 kDa	0.40	0.00
E7EU23	Cluster of Rab GDP dissociation inhibitor beta	GDI2	51 kDa	0.40	0.00
B4DW92	Nuclear receptor 2C2-associated protein	NR2C2AP	19 kDa	2.90	0.00
C9J180	Ubiquitin-conjugating enzyme E2 E2 (Fragment)	UBE2E2	18 kDa	3.70	0.05
G8JLD5	Cluster of Dynamin-1-like protein	DNM1L	80 kDa	0.60	0.00
Q9NZT2	Opioid growth factor receptor	OGFR	73 kDa	1.75	0.04
J3KN36	Cluster of Nodal modulator 3	NOMO3	139 kDa	0.60	0.00
J3KNT0	Cluster of Fascin	FSCN1	52 kDa	0.40	0.00
P0C0S5	Cluster of Histone H2A.Z	H2AFZ	14 kDa	0.40	0.00
Q9Y265	Cluster of RuvB-like 1	RUVBL1	50 kDa	0.50	0.00
P07093	Cluster of Isoform 2 of Glia-derived nexin	SERPINE2	44 kDa	0.50	0.00
J3KPM7	NADH dehydrogenase [ubiquinone] iron-sulfur protein 2, mitochondrial	NDUFS2	52 kDa	1.70	0.00
A6NN98	Myotubularin related protein 2, isoform CRA_a	MTMR2	66 kDa	2.40	0.05
Q14258	E3 ubiquitin/ISG15 ligase TRIM25	TRIM25	71 kDa	0.60	0.00
Q9C0D3	Protein zyg-11 homolog B	ZYG11B	84 kDa	2.00	0.00
Q13435	Cluster of Splicing factor 3B subunit 2	SF3B2	100 kDa	0.50	0.00
B5MC72	Endoplasmic reticulum lectin 1	ERLEC1	52 kDa	1.75	0.04
P55884	Eukaryotic translation initiation factor 3 subunit B	EIF3B	92 kDa	0.35	0.05
P08727	Cluster of Keratin, type I cytoskeletal 19	KRT19	44 kDa	0.35	0.05
B4DGU4	Cluster of Catenin beta-1	CTNNB1	85 kDa	0.40	0.00
P13797	Cluster of Plastin-3	PLS3	71 kDa	0.50	0.00
P21589	Cluster of 5'-nucleotidase	NT5E	63 kDa	0.60	0.00
Q9P2X0	Dolichol-phosphate mannosyltransferase subunit 3	DPM3	10 kDa	1.85	0.04
Q92896	Cluster of Isoform 3 of Golgi apparatus protein 1	GLG1	136 kDa	0.30	0.00
B1AK87	Cluster of Capping protein (Actin filament) muscle Z-line, beta	CAPZB	29 kDa	0.30	0.00
Q9BXP5	Serrate RNA effector molecule homolog	SRRT	101 kDa	0.60	0.00
H0YE28	Protein C11orf31 (Fragment)	C11orf31	10 kDa	1.95	0.03
Q8N163	Cluster of DBIRD complex subunit KIAA1967	KIAA1967	103 kDa	0.60	0.00
Q92499	ATP-dependent RNA helicase DDX1	DDX1	82 kDa	0.50	0.00
J3KNX7	NADH dehydrogenase (Ubiquinone) flavoprotein 3, 10kDa, isoform CRA_d	NDUFV3	51 kDa	1.70	0.00
C9JKL2	MLN64 N-terminal domain homolog	STARD3NL	25 kDa	3.05	0.02
Q13867	Cluster of Bleomycin hydrolase	BLMH	53 kDa	0.60	0.00
Q9BRT6	Protein LLP homolog	LLPH	15 kDa	2.95	0.05
I3L1I0	Glyoxalase domain-containing protein 4 (Fragment)	GLOD4	13 kDa	3.80	0.05
P49327	Fatty acid synthase	FASN	273 kDa	0.20	0.00

P15531	Cluster of Nucleoside diphosphate kinase A	NME1	17 kDa	0.30	0.00
Q96B45	UPF0693 protein C10orf32	C10orf32	12 kDa	1.60	0.00
O15431	High affinity copper uptake protein 1	SLC31A1	21 kDa	1.70	0.00
D6RGG3	Cluster of Collagen alpha-1(XII) chain	COL12A1	333 kDa	0.35	0.05
B4DQJ8	6-phosphogluconate dehydrogenase, decarboxylating	PGD	52 kDa	0.35	0.05
O75955	Flotillin-1	FLOT1	47 kDa	1.85	0.04
Q13043	Cluster of Serine/threonine-protein kinase 4	STK4	56 kDa	2.05	0.03
Q99832	Cluster of T-complex protein 1 subunit eta	CCT7	59 kDa	0.30	0.00
O00231	Cluster of 26S proteasome non-ATPase regulatory subunit 11	PSMD11	47 kDa	0.35	0.05
P48444	Cluster of Coatomer subunit delta	ARCN1	57 kDa	0.35	0.05
Q9BWM7	Sideroflexin-3	SFXN3	36 kDa	1.75	0.04
P04083	Annexin A1	ANXA1	39 kDa	0.30	0.00
Q99988	Growth/differentiation factor 15	GDF15	34 kDa	0.30	0.00
F5H2S7	Dynactin subunit 2	DCTN2	45 kDa	0.60	0.00
G3V5Z7	Cluster of Proteasome subunit alpha type	PSMA6	28 kDa	0.50	0.00
P13726	Tissue factor	F3	33 kDa	2.05	0.03
P09874	Poly [ADP-ribose] polymerase 1	PARP1	113 kDa	0.40	0.00
A9UHW6	MIF4G domain-containing protein	MIF4GD	25 kDa	2.15	0.03
P14866	Heterogeneous nuclear ribonucleoprotein L	HNRNPL	64 kDa	0.50	0.00
H7C2C7	Cluster of Negative elongation factor A (Fragment)	WHSC2	27 kDa	3.65	0.01
Q5VST3	Cluster of Protein O-linked mannose beta1,2-N-acetylglucosaminyltransferase	POMGNT1	85 kDa	1.60	0.00
F5GWX5	Cluster of Chromodomain-helicase-DNA-binding protein 4	CHD4	217 kDa	0.50	0.00
Q02878	60S ribosomal protein L6	RPL6	33 kDa	0.60	0.00
Q96A49	Synapse-associated protein 1	SYAP1	40 kDa	1.60	0.00
H3BP13	Trafficking protein particle complex subunit 2-like protein	TRAPPC2L	27 kDa	2.30	0.00
Q96SB3	Neurabin-2	PPP1R9B	89 kDa	1.65	0.05
Q9Y6A4	UPF0468 protein C16orf80	C16orf80	23 kDa	1.65	0.05
H3BRS6	ADP-dependent glucokinase (Fragment)	ADPGK	22 kDa	1.65	0.05
Q5JTD0	Tight junction-associated protein 1	TJAP1	62 kDa	2.90	0.00
E7EWE9	Kinetochore protein Spc24	SPC24	17 kDa	1.85	0.04
P26599	Cluster of Polypyrimidine tract-binding protein 1	PTBP1	57 kDa	0.60	0.00
Q9H0D6	5'-3' exoribonuclease 2	XRN2	109 kDa	0.60	0.00
B4E040	Ras-related protein Ral-B	RALB	26 kDa	1.75	0.04
H3BM91	COMM domain-containing protein 4 (Fragment)	COMMD4	23 kDa	1.85	0.04
O15230	Laminin subunit alpha-5	LAMA5	400 kDa	0.30	0.00
Q06210	Cluster of Glucosamine--fructose-6-phosphate aminotransferase [isomerizing] 1	GFPT1	79 kDa	0.50	0.00
H0YBQ0	Thioredoxin reductase 3 (Fragment)	TXNRD3	76 kDa	1.85	0.04
P49792	Cluster of E3 SUMO-protein ligase RanBP2	RANBP2	358 kDa	0.60	0.00
Q5VZE5	N-alpha-acetyltransferase 35, NatC auxiliary subunit	NAA35	84 kDa	1.80	0.00
G5EA42	Cluster of Tropomodulin 2 (Neuronal), isoform CRA_a	TMOD2	34 kDa	4.40	0.00
E9PKG1	Cluster of Protein arginine N-methyltransferase 1	PRMT1	38 kDa	0.40	0.00
Q06481	Cluster of Amyloid-like protein 2	APLP2	87 kDa	0.50	0.00
E9PDI4	Ladinin-1	LAD1	59 kDa	1.70	0.00
P08574	Cytochrome c1, heme protein, mitochondrial	CYC1	35 kDa	1.65	0.05
Q9UKZ1	UPF0760 protein C2orf29	C2orf29	55 kDa	1.85	0.04
H3BLY1	Cluster of Contactin-associated protein-like 3B (Fragment)	CNTNAP3B	129 kDa	2.05	0.03
A7MAP0	Cluster of Coronin	CORO1C	54 kDa	0.60	0.00
Q9H993	UPF0364 protein C6orf211	C6orf211	51 kDa	2.70	0.04
O75449	Cluster of Katanin p60 ATPase-containing subunit A1	KATNA1	56 kDa	1.75	0.04
Q49AR2	UPF0489 protein C5orf22	C5orf22	50 kDa	1.85	0.04
C9JWR9	3-phosphoinositide-dependent protein kinase 1	PDPK1	61 kDa	1.95	0.03
Q8IYB8	ATP-dependent RNA helicase SUPV3L1, mitochondrial	SUPV3L1	88 kDa	1.70	0.00
P10619	Lysosomal protective protein	CTSA	54 kDa	5.65	0.01

Q8TEQ6	Gem-associated protein 5	GEMIN5	169 kDa	0.60	0.00
F8W785	Golgi integral membrane protein 4	GOLIM4	79 kDa	3.05	0.02
P49368	Cluster of T-complex protein 1 subunit gamma	CCT3	61 kDa	0.30	0.00
P67809	Cluster of Nuclease-sensitive element-binding protein 1	YBX1	36 kDa	0.60	0.00
Q9HB71	Calcyclin-binding protein	CACYBP	26 kDa	0.60	0.00
Q96FJ0	AMSH-like protease	STAMBPL1	50 kDa	2.25	0.03
Q14232	Cluster of Translation initiation factor eIF-2B subunit	EIF2B1	34 kDa	0.60	0.00
A6NDW3	Cluster of Lipolysis-stimulated lipoprotein receptor	LSR	64 kDa	0.60	0.00
Q96NB3	Zinc finger protein 830	ZNF830	42 kDa	1.75	0.04
Q9UBS3	DnaJ homolog subfamily B member 9	DNAJB9	26 kDa	1.90	0.00
Q5TEC6	Cluster of Histone H3	HIST2H3PS2	15 kDa	0.50	0.00
O75143	Cluster of Isoform 3 of Autophagy-related protein 13	ATG13	44 kDa	2.16	0.05
J3KR35	Coiled-coil domain containing 12, isoform CRA_a	CCDC12	21 kDa	3.70	0.02
Q9NZV1	Cysteine-rich motor neuron 1 protein	CRIM1	114 kDa	0.60	0.00
P49441	Inositol polyphosphate 1-phosphatase	INPP1	44 kDa	1.60	0.00
Q9NP77	Cluster of RNA polymerase II subunit A C-terminal domain phosphatase SSU72	SSU72	23 kDa	1.60	0.00
H3BM74	NEDD8 ultimate buster 1	NUB1	73 kDa	1.80	0.00
F5H897	Cluster of Heat shock protein 75 kDa, mitochondrial	TRAP1	74 kDa	0.60	0.00
Q9C0B0	RING finger protein unkempt homolog	UNK	88 kDa	1.75	0.04
F5GX65	Armadillo repeat-containing protein 10	ARMC10	25 kDa	1.65	0.05
H0YJ66	Dehydrogenase/reductase SDR family member 7 (Fragment)	DHRS7	45 kDa	1.75	0.04
A2RUC4	Cluster of tRNA wybutosine-synthesizing protein 5	TYW5	37 kDa	1.85	0.04
E7EP00	Protein transport protein Sec24C	SEC24C	107 kDa	0.60	0.00
Q8N0X4	Citrate lyase subunit beta-like protein, mitochondrial	CLYBL	37 kDa	1.90	0.00
G3V1S2	Cullin-2	CUL2	89 kDa	0.60	0.00
Q01432	AMP deaminase 3	AMPD3	89 kDa	1.70	0.00
Q8NFQ8	Torsin-1A-interacting protein 2	TOR1AIP2	51 kDa	2.50	0.00
J3KQG6	Cluster of Eukaryotic peptide chain release factor GTP-binding subunit ERF3A	GSPT1	69 kDa	0.60	0.00
P00390	Glutathione reductase, mitochondrial	GSR	56 kDa	0.60	0.00
P49589	Cluster of Isoform 3 of Cysteine--tRNA ligase, cytoplasmic	CARS	95 kDa	0.25	0.04
H3BMS0	RNA-binding protein with serine-rich domain 1	RNPS1	27 kDa	1.65	0.05
Q9BQP7	Uncharacterized protein C20orf72	C20orf72	39 kDa	2.15	0.03
P46940	Cluster of Ras GTPase-activating-like protein IQGAP1	IQGAP1	189 kDa	0.30	0.00
Q13347	Eukaryotic translation initiation factor 3 subunit I	EIF3I	37 kDa	0.60	0.00
P52272	Heterogeneous nuclear ribonucleoprotein M	HNRNPM	78 kDa	0.60	0.00
Q6UVK1	Chondroitin sulfate proteoglycan 4	CSPG4	251 kDa	0.60	0.00
P55011	Solute carrier family 12 member 2	SLC12A2	131 kDa	1.90	0.00
Q6NUK1	Calcium-binding mitochondrial carrier protein SCaMC-1	SLC25A24	53 kDa	1.85	0.04
Q9NWU2	Glucose-induced degradation protein 8 homolog	GID8	27 kDa	1.85	0.04
P60228	Cluster of Eukaryotic translation initiation factor 3 subunit E	EIF3E	52 kDa	6.75	0.02
E7EPD0	Cluster of Target of Myb protein 1	TOM1	50 kDa	2.70	0.04
Q9BRS2	Serine/threonine-protein kinase RIO1	RIOK1	66 kDa	2.45	0.02
P36873	Cluster of Isoform Gamma-2 of Serine/threonine-protein phosphatase PP1-gamma catalytic subunit	PPP1CC	39 kDa	0.60	0.00
P11717	Cation-independent mannose-6-phosphate receptor	IGF2R	274 kDa	0.40	0.00
O43175	D-3-phosphoglycerate dehydrogenase	PHGDH	57 kDa	0.50	0.00
Q6ZMU5	Tripartite motif-containing protein 72	TRIM72	53 kDa	0.60	0.00
Q92575	UBX domain-containing protein 4	UBXN4	57 kDa	1.85	0.04
P07951	Cluster of Isoform 3 of Tropomyosin beta chain	TPM2	29 kDa	0.30	0.00
P49915	GMP synthase [glutamine-hydrolyzing]	GMPS	77 kDa	0.50	0.00
Q92859	Neogenin	NEO1	160 kDa	0.50	0.00
F5H737	Adenosylhomocysteinase	AHCY	45 kDa	0.50	0.00

P61289	Proteasome activator complex subunit 3	PSME3	30 kDa	0.60	0.00
Q9UDY4	DnaJ homolog subfamily B member 4	DNAJB4	38 kDa	1.70	0.00
Q9NZN4	Cluster of EH domain-containing protein 2	EHD2	61 kDa	2.35	0.02
Q5R3F8	Protein phosphatase 1 regulatory subunit 29	ELFN2	90 kDa	3.75	0.01
P08240	Signal recognition particle receptor subunit alpha	SRPR	70 kDa	1.80	0.00
Q02790	Cluster of Peptidyl-prolyl cis-trans isomerase FKBP4	FKBP4	52 kDa	0.35	0.05
P07996	Cluster of Thrombospondin-1	THBS1	129 kDa	0.40	0.00
P21964	Catechol O-methyltransferase	COMT	30 kDa	1.85	0.04
O60921	Cluster of Checkpoint protein HUS1	HUS1	32 kDa	2.65	0.02
P08123	Collagen alpha-2(I) chain	COL1A2	129 kDa	2.95	0.02
Q9HD47	Cluster of Ran guanine nucleotide release factor	RANGRF	20 kDa	1.65	0.05
Q9ULH1	Arf-GAP with SH3 domain, ANK repeat and PH domain-containing protein 1	ASAP1	126 kDa	1.60	0.00
Q14CX7	Cluster of N-alpha-acetyltransferase 25, NatB auxiliary subunit	NAA25	112 kDa	0.50	0.00
Q9BWD1	Cluster of Acetyl-CoA acetyltransferase, cytosolic	ACAT2	41 kDa	0.50	0.00
Q9Y2S7	Polymerase delta-interacting protein 2	POLDIP2	42 kDa	1.90	0.00
O75683	Surfeit locus protein 6	SURF6	41 kDa	1.65	0.05
H3BTI0	Cysteine-rich secretory protein LCCL domain-containing 2	CRISPLD2	56 kDa	1.85	0.04
B2RAK2	MHC class I polypeptide-related sequence B	MICB	43 kDa	1.95	0.03
Q92889	DNA repair endonuclease XPF	ERCC4	104 kDa	2.45	0.02
P61218	Cluster of DNA-directed RNA polymerases I, II, and III subunit RPABC2	POLR2F	14 kDa	1.75	0.04
Q00610	Cluster of Clathrin heavy chain 1	CLTC	192 kDa	5.80	0.00
D6RER5	Cluster of Septin-11	SEPT11	50 kDa	0.50	0.00
P36551	Coproporphyrinogen-III oxidase, mitochondrial	CPOX	50 kDa	0.60	0.00
P24534	Elongation factor 1-beta	EEF1B2	25 kDa	0.35	0.05
F5H7V7	NADH dehydrogenase [ubiquinone] 1 alpha subcomplex subunit 8	NDUFA8	15 kDa	1.65	0.05
Q96TC7	Cluster of Regulator of microtubule dynamics protein 3	FAM82A2	52 kDa	2.05	0.03
Q9BUT1	Cluster of 3-hydroxybutyrate dehydrogenase type 2	BDH2	27 kDa	2.10	0.00
Q96I99	Isoform 2 of Succinyl-CoA ligase [GDP-forming] subunit beta, mitochondrial	SUCLG2	48 kDa	1.75	0.04
B4DVE7	Cluster of Annexin	ANXA11	51 kDa	0.60	0.00
Q9H7P6	Cluster of Multivesicular body subunit 12B	FAM125B	36 kDa	1.65	0.05
O60568	Procollagen-lysine,2-oxoglutarate 5-dioxygenase 3	PLOD3	85 kDa	0.30	0.00
Q9H081	Protein MIS12 homolog	MIS12	24 kDa	1.60	0.00
P09429	Cluster of High mobility group protein B1	HMGB1	25 kDa	0.25	0.04
Q8IWB7	WD repeat and FYVE domain-containing protein 1	WDFY1	46 kDa	1.90	0.00
B4DTG2	Cluster of Elongation factor 1-gamma	EEF1G	56 kDa	0.35	0.05
D6W5A2	Metastasis associated 1 family, member 3, isoform CRA_a	MTA3	61 kDa	1.70	0.00
P08107	Cluster of Heat shock 70 kDa protein 1A/1B	HSPA1A	70 kDa	0.25	0.04
P22314	Cluster of Ubiquitin-like modifier-activating enzyme 1	UBA1	118 kDa	0.35	0.05
E7EPA7	Transketolase	TKT	69 kDa	0.20	0.00
D6RAK8	Cluster of Vitamin D-binding protein	GC	55 kDa	0.60	0.00
P01024	Complement C3	C3	187 kDa	0.40	0.00
Q9Y2Q5	Ragulator complex protein LAMTOR2	LAMTOR2	14 kDa	2.19	0.01
O75843	Cluster of AP-1 complex subunit gamma-like 2	AP1G2	87 kDa	2.10	0.00
O14732	Inositol monophosphatase 2	IMPA2	31 kDa	3.00	0.00
B7Z3D5	Cluster of Myotubularin related protein 1, isoform CRA_a	MTMR1	65 kDa	1.65	0.05
P29372	Isoform 2 of DNA-3-methyladenine glycosylase	MPG	32 kDa	1.65	0.05
G3V4K3	Spermatogenesis-defective protein 39 homolog	VIPAS39	60 kDa	1.95	0.03
J3QRS3	Cluster of Myosin regulatory light chain 12A	MYL12A	20 kDa	0.60	0.00
P19387	DNA-directed RNA polymerase II subunit RPB3	POLR2C	31 kDa	3.40	0.00
Q01813	Cluster of 6-phosphofructokinase type C	PFKP	86 kDa	0.50	0.00

Q01970	1-phosphatidylinositol 4,5-bisphosphate phosphodiesterase beta-3	PLCB3	139 kDa	0.50	0.00
O95104	Splicing factor, arginine/serine-rich 15	SCAF4	126 kDa	2.10	0.00
O94813	Slit homolog 2 protein	SLIT2	170 kDa	1.75	0.04
Q9UBT2	SUMO-activating enzyme subunit 2	UBA2	71 kDa	0.50	0.00
P09525	Cluster of Annexin A4	ANXA4	36 kDa	0.60	0.00
Q15031	Probable leucine--tRNA ligase, mitochondrial	LARS2	102 kDa	3.25	0.04
A6NE09	Protein RPSAP58	RPSAP58	33 kDa	6.95	0.03
Q99622	Protein C10	C12orf57	13 kDa	3.05	0.05
O15084	Serine/threonine-protein phosphatase 6 regulatory ankyrin repeat subunit A	ANKRD28	113 kDa	1.95	0.03
Q13740	Isoform 2 of CD166 antigen	ALCAM	64 kDa	0.60	0.00
Q16787	Laminin subunit alpha-3	LAMA3	367 kDa	0.60	0.00
P10809	Cluster of 60 kDa heat shock protein, mitochondrial	HSPD1	61 kDa	0.35	0.05
Q12905	Interleukin enhancer-binding factor 2	ILF2	43 kDa	0.60	0.00
P06744	Glucose-6-phosphate isomerase	GPI	63 kDa	0.25	0.04
Q13813	Cluster of Spectrin alpha chain, non-erythrocytic 1	SPTAN1	285 kDa	0.35	0.05
Q7Z7E8	Ubiquitin-conjugating enzyme E2 Q1	UBE2Q1	46 kDa	1.65	0.05
E9PJM3	Cluster of F-box only protein 3	FBXO3	49 kDa	1.85	0.04
P98160	Cluster of Basement membrane-specific heparan sulfate proteoglycan core protein	HSPG2	469 kDa	0.30	0.00
F8W8J4	Myoferlin	MYOF	235 kDa	0.50	0.00
O75533	Splicing factor 3B subunit 1	SF3B1	146 kDa	0.50	0.00
P16403	Cluster of Histone H1.2	HIST1H1C	21 kDa	0.50	0.00
P27348	Cluster of 14-3-3 protein theta	YWHAQ	28 kDa	0.50	0.00
F5HFY4	Cluster of Nucleosome assembly protein 1-like 4	NAP1L4b	44 kDa	0.60	0.00
G5E9U1	Cdc42-interacting protein 4	TRIP10	68 kDa	0.60	0.00
Q15020	Cluster of Squamous cell carcinoma antigen recognized by T-cells 3	SART3	110 kDa	0.60	0.00
Q3LXA3	Cluster of Bifunctional ATP-dependent dihydroxyacetone kinase/FAD-AMP lyase (cyclizing)	DAK	59 kDa	0.60	0.00
Q8TAA9	Vang-like protein 1	VANGL1	60 kDa	1.70	0.00
O95999	B-cell lymphoma/leukemia 10	BCL10	26 kDa	1.80	0.00
Q8NFU3	Thiosulfate sulfurtransferase/rhodanese-like domain-containing protein 1	TSTD1	13 kDa	2.00	0.00
P05455	Lupus La protein	SSB	47 kDa	0.35	0.05
Q96QU8	Exportin-6	XPO6	129 kDa	1.65	0.05
Q86UN2	Reticulon-4 receptor-like 1	RTN4RL1	49 kDa	1.75	0.04
P13497	Cluster of Bone morphogenetic protein 1	BMP1	111 kDa	0.60	0.00
Q15075	Early endosome antigen 1	EEA1	162 kDa	0.50	0.00
Q96FQ6	Protein S100-A16	S100A16	12 kDa	0.60	0.00
B1AUU8	Cluster of Epidermal growth factor receptor pathway substrate 15	EPS15	84 kDa	1.60	0.00
Q9H1K1	Iron-sulfur cluster assembly enzyme ISCU, mitochondrial	ISCU	18 kDa	3.40	0.03
I3L3S0	Trafficking protein particle complex subunit 1 (Fragment)	TRAPPC1	11 kDa	2.25	0.03
O15400	Syntaxin-7	STX7	30 kDa	1.60	0.00
Q8IZ73	RNA pseudouridylate synthase domain-containing protein 2	RPUSD2	61 kDa	1.70	0.00
Q01518	Cluster of Adenylyl cyclase-associated protein 1	CAP1	52 kDa	0.40	0.00
Q9Y5B9	FACT complex subunit SPT16	SUPT16H	120 kDa	0.35	0.05
P07108	Cluster of Isoform 4 of Acyl-CoA-binding protein	DBI	14 kDa	1.65	0.05
F5GWN1	Protein SSXT	SS18	4 kDa	2.15	0.03
P68104	Elongation factor 1-alpha 1	EEF1A1	50 kDa	0.40	0.00
P43490	Nicotinamide phosphoribosyltransferase	NAMPT	56 kDa	0.50	0.00
P07954	Fumarate hydratase, mitochondrial	FH	55 kDa	0.60	0.00
Q14181	DNA polymerase alpha subunit B	POLA2	66 kDa	2.20	0.00

Q9BX68	Histidine triad nucleotide-binding protein 2, mitochondrial	HINT2	17 kDa	1.60	0.00
P12429	Annexin A3	ANXA3	36 kDa	0.35	0.05
O43505	N-acetyllactosaminide beta-1,3-N-acetylglucosaminyltransferase	B3GNT1	47 kDa	1.65	0.05
E9PMG1	Cluster of RalBP1-associated Eps domain-containing protein 1	REPS1	80 kDa	2.05	0.03
Q16706	Cluster of Alpha-mannosidase 2	MAN2A1	131 kDa	0.60	0.00
Q9UGI8	Cluster of Isoform 2 of Testin	TES	47 kDa	0.50	0.00
P52907	F-actin-capping protein subunit alpha-1	CAPZA1	33 kDa	0.60	0.00
Q9Y6X9	MORC family CW-type zinc finger protein 2	MORC2	118 kDa	1.65	0.05
P07814	Bifunctional glutamate/proline--tRNA ligase	EPRS	171 kDa	0.47	0.02
Q99747	Gamma-soluble NSF attachment protein	NAPG	35 kDa	1.65	0.05
F5H098	Malate dehydrogenase, cytoplasmic	MDH1	39 kDa	0.50	0.00
E9PRZ9	Protein C11orf58 (Fragment)	C11orf58	10 kDa	1.60	0.00
P24592	Insulin-like growth factor-binding protein 6	IGFBP6	25 kDa	0.60	0.00
Q9H9A6	Leucine-rich repeat-containing protein 40	LRRC40	68 kDa	1.80	0.00
Q969V3	Nicalin	NCLN	63 kDa	1.75	0.04
P18669	Cluster of Phosphoglycerate mutase 1	PGAM1	29 kDa	0.50	0.00
P26196	Probable ATP-dependent RNA helicase DDX6	DDX6	54 kDa	0.60	0.00
D6RF48	Syntaxin-18	STX18	35 kDa	1.60	0.00
O00425	Insulin-like growth factor 2 mRNA-binding protein 3	IGF2BP3	64 kDa	1.75	0.04
Q96IY1	Cluster of Kinetochore-associated protein NSL1 homolog	NSL1	32 kDa	1.75	0.04
Q9NRN7	L-aminoadipate-semialdehyde dehydrogenase-phosphopantetheinyl transferase	AASDHPPT	36 kDa	1.60	0.00
O43776	Asparagine--tRNA ligase, cytoplasmic	NARS	63 kDa	0.40	0.00
P53621	Coatomer subunit alpha	COPA	138 kDa	0.40	0.00
F5H2A7	Prefoldin subunit 3	VBP1	22 kDa	0.60	0.00
Q9BYG3	MKI67 FHA domain-interacting nucleolar phosphoprotein	MKI67IP	34 kDa	2.20	0.00
P12109	Collagen alpha-1(VI) chain	COL6A1	109 kDa	0.25	0.04
P24821	Isoform 6 of Tenascin	TNC	171 kDa	2.00	0.00
P62699	Protein yippee-like 5	YPEL5	14 kDa	3.60	0.05
P60033	Cluster of CD81 antigen	CD81	26 kDa	0.60	0.00
E9PDC5	Receptor-type tyrosine-protein phosphatase S	PTPRS	217 kDa	0.60	0.00
P17655	Cluster of Calpain-2 catalytic subunit	CAPN2	80 kDa	0.30	0.00
O75083	Cluster of WD repeat-containing protein 1	WDR1	66 kDa	0.40	0.00
P09234	Cluster of U1 small nuclear ribonucleoprotein C	SNRPC	17 kDa	2.00	0.00
P45985	Dual specificity mitogen-activated protein kinase kinase 4	MAP2K4	44 kDa	1.80	0.00
Q9NRL2	Bromodomain adjacent to zinc finger domain protein 1A	BAZ1A	179 kDa	4.25	0.05
F8WBS8	26S proteasome non-ATPase regulatory subunit 2	PSMD2	7 kDa	1.70	0.00
O75521	Enoyl-CoA delta isomerase 2, mitochondrial	ECI2	44 kDa	2.00	0.00
C9JJV1	Protein TSSC4 (Fragment)	TSSC4	22 kDa	2.40	0.05
Q8NC60	Nitric oxide-associated protein 1	NOA1	78 kDa	2.65	0.02
E9PEZ3	Cluster of Protein diaphanous homolog 1	DIAPH1	141 kDa	0.50	0.00
O60645	Exocyst complex component 3	EXOC3	87 kDa	1.80	0.00
Q9BSB4	Autophagy-related protein 101	ATG101	25 kDa	2.60	0.00
Q14008	Cluster of Cytoskeleton-associated protein 5	CKAP5	226 kDa	0.50	0.00
Q9NRB3	Carbohydrate sulfotransferase 12	CHST12	48 kDa	3.50	0.03
Q96QK1	Cluster of Vacuolar protein sorting-associated protein 35	VPS35	92 kDa	0.50	0.00
O75752	UDP-GalNAc:beta-1,3-N-acetylgalactosaminyltransferase 1	B3GALNT1	40 kDa	1.85	0.04
Q2NL82	Pre-rRNA-processing protein TSR1 homolog	TSR1	92 kDa	2.25	0.03
E9PBB0	PERQ amino acid-rich with GYF domain-containing protein 2	GIGYF2	149 kDa	3.62	0.02
Q9NX55	Cluster of Huntingtin-interacting protein K	HYPK	15 kDa	2.05	0.03
O95373	Importin-7	IPO7	120 kDa	0.60	0.00
Q12962	Transcription initiation factor TFIID subunit 10	TAF10	22 kDa	1.90	0.00

P31153	Cluster of S-adenosylmethionine synthase isoform type-2	MAT2A	44 kDa	0.60	0.00
O96005	Cleft lip and palate transmembrane protein 1	CLPTM1	76 kDa	2.65	0.02
E9PCI3	Asparagine synthetase	ASNS	62 kDa	0.40	0.00
Q9NX46	Poly(ADP-ribose) glycohydrolase ARH3	ADPRHL2	39 kDa	1.70	0.00
Q9Y697	Cluster of Isoform Cytoplasmic of Cysteine desulfurase, mitochondrial	NFS1	44 kDa	2.75	0.02
Q9Y3C7	Mediator of RNA polymerase II transcription subunit 31	MED31	16 kDa	2.24	0.01
Q96T23	Remodeling and spacing factor 1	RSF1	164 kDa	1.60	0.00
P07902	Cluster of Galactose-1-phosphate uridylyltransferase	GALT	43 kDa	2.50	0.04
P04075	Cluster of Fructose-bisphosphate aldolase A	ALDOA	39 kDa	0.20	0.00
Q86VP6	Cluster of Cullin-associated NEDD8-dissociated protein 1	CAND1	136 kDa	0.30	0.00
O00232	26S proteasome non-ATPase regulatory subunit 12	PSMD12	53 kDa	0.60	0.00
P63104	Cluster of 14-3-3 protein zeta/delta	YWHAZ	28 kDa	0.35	0.05
P41240	Tyrosine-protein kinase CSK	CSK	51 kDa	1.65	0.05
Q5NDL2	EGF domain-specific O-linked N-acetylglucosamine transferase	EOGT	62 kDa	1.65	0.05
E5RK00	Dynactin subunit 6	DCTN6	18 kDa	1.65	0.05
Q8WXI9	Transcriptional repressor p66-beta	GATAD2B	65 kDa	1.75	0.04
Q9NQZ2	Something about silencing protein 10	UTP3	55 kDa	1.75	0.04
H7BZW1	Cluster of Uncharacterized protein (Fragment)		34 kDa	2.05	0.03
P14618	Cluster of Isoform 3 of Pyruvate kinase isozymes M1/M2	PKM	56 kDa	0.10	0.00
P11586	Cluster of C-1-tetrahydrofolate synthase, cytoplasmic	MTHFD1	102 kDa	0.50	0.00
Q14118	Dystroglycan	DAG1	97 kDa	0.50	0.00
Q9UBS4	Cluster of DnaJ homolog subfamily B member 11	DNAJB11	41 kDa	0.50	0.00
E7EP74	Cluster of Golgin subfamily B member 1	GOLGB1	377 kDa	0.50	0.00
P26447	Protein S100-A4	S100A4	12 kDa	0.60	0.00
Q9Y3I0	tRNA-splicing ligase RtcB homolog	C22orf28	55 kDa	0.60	0.00
E9PLM6	Midkine	MDK	17 kDa	0.60	0.00
H0Y7A7	Calmodulin (Fragment)	CALM2	21 kDa	0.60	0.00
P49189	4-trimethylaminobutyraldehyde dehydrogenase	ALDH9A1	54 kDa	0.60	0.00
Q14683	Structural maintenance of chromosomes protein 1A	SMC1A	143 kDa	0.60	0.00
Q14444	Cluster of Caprin-1	CAPRIN1	78 kDa	0.60	0.00
Q5SSJ5	Cluster of Heterochromatin protein 1-binding protein 3	HP1BP3	61 kDa	0.60	0.00
B4DVY1	Eukaryotic translation initiation factor 3 subunit D	EIF3D	58 kDa	0.60	0.00
Q9UNH7	Cluster of Sorting nexin-6	SNX6	47 kDa	0.60	0.00
O14776	Cluster of Transcription elongation regulator 1	TCERG1	124 kDa	0.60	0.00
O15143	Actin-related protein 2/3 complex subunit 1B	ARPC1B	41 kDa	0.60	0.00
P31947	14-3-3 protein sigma	SFN	28 kDa	0.60	0.00
P62750	Cluster of 60S ribosomal protein L23a	RPL23A	18 kDa	0.60	0.00
Q5JTV8	Torsin-1A-interacting protein 1	TOR1AIP1	66 kDa	1.80	0.00
B4DP36	Calcium-binding and coiled-coil domain-containing protein 2	CALCOCO2	55 kDa	2.00	0.00
E9PC90	Cluster of G2/mitotic-specific cyclin-B1 (Fragment)	CCNB1	45 kDa	2.00	0.00
G8JLI5	WD repeat domain phosphoinositide-interacting protein 4	WDR45	38 kDa	2.30	0.00
O14964	Hepatocyte growth factor-regulated tyrosine kinase substrate	HGS	86 kDa	0.60	0.05

Supplementary Table S5. The differentially expressed proteins between MT and KO cells.

Accession Number	Protein subgroup name	Gene symbol	Molecular Weight	FC (MT/KO)	P-value (MT/KO)
O94985	Isoform 2 of Calsyntenin-1	CLSTN1	109 kDa	5.08	0.04
J3KQQ3	Zinc finger and BTB domain-containing protein 7B	ZBTB7B	62 kDa	0.13	0.00
G3XAD8	Cluster of Stress-induced-phosphoprotein 1	STIP1	68 kDa	3.50	0.03
B5ME19	Eukaryotic translation initiation factor 3 subunit C	EIF3CL	105 kDa	2.88	0.03
Q86XP3	ATP-dependent RNA helicase DDX42	DDX42	103 kDa	2.35	0.04
O95573	Cluster of Long-chain-fatty-acid--CoA ligase 3	ACSL3	80 kDa	0.29	0.00
Q8TDW7	Protocadherin Fat 3	FAT3	506 kDa	0.35	0.01
Q08945	Cluster of FACT complex subunit SSRP1	SSRP1	81 kDa	3.00	0.05
P53396	Cluster of ATP-citrate synthase	ACLY	121 kDa	3.67	0.02
P28799	Granulins	GRN	64 kDa	2.45	0.03
P07355	Cluster of Isoform 2 of Annexin A2	ANXA2	40 kDa	6.25	0.01
Q8TF74	WAS/WASL-interacting protein family member 2	WIPF2	46 kDa	0.29	0.01
E2QRD5	Protein C15orf38-AP3S2	C15orf38-AP3S2	44 kDa	0.58	0.03
E9PDM8	Cluster of Protein transport protein Sec24D	SEC24D	75 kDa	2.39	0.05
P38919	Cluster of Eukaryotic initiation factor 4A-III	EIF4A3	47 kDa	3.42	0.01
E9PFM1	Cluster of Eukaryotic translation initiation factor 4 gamma	EIF4G1	176 kDa	2.15	0.03
Q9NQZ5	StAR-related lipid transfer protein 7, mitochondrial	STARD7	43 kDa	2.04	0.03
F8VUJ3	Cluster of Protein POC1B-GALNT4	POC1B-GALNT4	66 kDa	0.30	0.00
P08581	Cluster of Hepatocyte growth factor receptor	MET	156 kDa	2.23	0.05
P11047	Laminin subunit gamma-1	LAMC1	178 kDa	2.80	0.03
P10586	Cluster of Receptor-type tyrosine-protein phosphatase F	PTPRF	213 kDa	2.33	0.02
F8VXB4	Cluster of Keratin, type II cytoskeletal 8	KRT8	57 kDa	7.00	0.01
B4DRN8	Cluster of Probable palmitoyltransferase ZDHHC20	ZDHHC20	34 kDa	0.57	0.02
P06396	Cluster of Isoform 2 of Gelsolin	GSN	81 kDa	2.93	0.03
P25398	40S ribosomal protein S12	RPS12	15 kDa	2.46	0.02
P00488	Coagulation factor XIII A chain	F13A1	83 kDa	0.45	0.00
Q9ULC5	Long-chain-fatty-acid--CoA ligase 5	ACSL5	76 kDa	0.58	0.01
Q16181	Cluster of Septin-7	SEPT7	51 kDa	2.33	0.02
P07195	Cluster of L-lactate dehydrogenase B chain	LDHB	37 kDa	0.14	0.00
P06748	Cluster of Nucleophosmin	NPM1	33 kDa	4.93	0.01
P12956	X-ray repair cross-complementing protein 6	XRCC6	70 kDa	0.30	0.00
Q9H4M9	Cluster of EH domain-containing protein 1	EHD1	61 kDa	2.18	0.04
Q9Y678	Cluster of Coatomer subunit gamma-1	COPG1	98 kDa	1.67	0.05
B4DUC8	Cluster of Purine nucleoside phosphorylase A	MTAP	33 kDa	2.21	0.04
P20042	Eukaryotic translation initiation factor 2 subunit 2	EIF2S2	38 kDa	2.29	0.04
P26639	Cluster of Threonine--tRNA ligase, cytoplasmic	TARS	83 kDa	1.75	0.04
G5EA52	Cluster of Protein disulfide isomerase family A, member 3, isoform CRA_b	PDIA3	55 kDa	3.50	0.02
A6NGV6	Aryl hydrocarbon receptor nuclear translocator	ARNT	46 kDa	0.53	0.01
Q13433	Zinc transporter ZIP6	SLC39A6	85 kDa	0.58	0.02
Q14980	Cluster of Isoform 2 of Nuclear mitotic apparatus protein 1	NUMA1	237 kDa	3.75	0.01
J3KPV7	Sulfurtransferase	MPST	35 kDa	0.45	0.01
P12270	Cluster of Nucleoprotein TPR	TPR	267 kDa	2.67	0.01
Q8NBP7	Cluster of Proprotein convertase subtilisin/kexin type 9	PCSK9	74 kDa	2.29	0.03
Q14204	Cytoplasmic dynein 1 heavy chain 1	DYNC1H1	532 kDa	3.25	0.01
Q9H6U6	Isoform 4 of Breast carcinoma-amplified sequence 3	BCAS3	104 kDa	0.57	0.05
P52292	Importin subunit alpha-2	KPNA2	58 kDa	2.89	0.02
P22234	Cluster of Multifunctional protein ADE2	PAICS	47 kDa	2.43	0.03
G5E9Y2	Cluster of X-prolyl aminopeptidase (Aminopeptidase P) 1, soluble, isoform CRA_b	XPNPEP1	75 kDa	4.04	0.01
Q9Y5X2	Sorting nexin-8	SNX8	53 kDa	0.41	0.00

P23528	Cofilin-1	CFL1	19 kDa	1.85	0.03
Q5BKZ1	DBIRD complex subunit ZNF326	ZNF326	66 kDa	0.55	0.01
P68363	Cluster of Tubulin alpha-1B chain	TUBA1B	50 kDa	2.25	0.02
O95295	SNARE-associated protein Snapin	SNAPIN	15 kDa	0.54	0.02
Q14019	Coactosin-like protein	COTL1	16 kDa	2.12	0.04
Q9NVZ3	Adaptin ear-binding coat-associated protein 2	NECAP2	28 kDa	0.50	0.01
P51178	1-phosphatidylinositol 4,5-bisphosphate phosphodiesterase delta-1	PLCD1	86 kDa	0.59	0.03
P82909	28S ribosomal protein S36, mitochondrial	MRPS36	11 kDa	0.56	0.01
O75643	U5 small nuclear ribonucleoprotein 200 kDa helicase	SNRNP200	245 kDa	4.08	0.01
Q05048	Cleavage stimulation factor subunit 1	CSTF1	48 kDa	1.93	0.02
P52294	Cluster of Importin subunit alpha-1	KPNA1	60 kDa	1.92	0.04
O00186	Syntaxin-binding protein 3	STXBP3	68 kDa	0.37	0.00
P13639	Elongation factor 2	EEF2	95 kDa	3.90	0.01
O95707	Ribonuclease P protein subunit p29	POP4	25 kDa	0.53	0.01
F6S928	Cluster of Mitochondrial import receptor subunit TOM5 homolog	TOMM5	10 kDa	0.54	0.03
P62333	Cluster of 26S protease regulatory subunit 10B	PSMC6	44 kDa	1.92	0.02
P60983	Cluster of Glia maturation factor beta	GMFB	17 kDa	0.54	0.01
O43805	Sjoegren syndrome nuclear autoantigen 1	SSNA1	14 kDa	0.35	0.00
P32119	Cluster of Peroxiredoxin-2	PRDX2	22 kDa	1.81	0.02
Q92945	Far upstream element-binding protein 2	KHSRP	73 kDa	4.40	0.00
P23284	Peptidyl-prolyl cis-trans isomerase B	PPIB	24 kDa	2.15	0.03
P12081	Cluster of Histidine--tRNA ligase, cytoplasmic	HARS	57 kDa	3.40	0.01
Q9NZ45	CDGSH iron-sulfur domain-containing protein 1	CISD1	12 kDa	0.63	0.02
Q9HA65	TBC1 domain family member 17	TBC1D17	73 kDa	0.40	0.00
O75874	Isocitrate dehydrogenase [NADP] cytoplasmic	IDH1	47 kDa	1.70	0.03
P14625	Cluster of Endoplasmin	HSP90B1	92 kDa	2.83	0.01
P78371	T-complex protein 1 subunit beta	CCT2	57 kDa	2.83	0.01
P40227	Cluster of T-complex protein 1 subunit zeta	CCT6A	58 kDa	2.13	0.01
P18206	Cluster of Vinculin	VCL	124 kDa	2.13	0.04
Q9C0B5	Palmitoyltransferase ZDHHC5	ZDHHC5	78 kDa	0.50	0.01
Q9HCD5	Nuclear receptor coactivator 5	NCOA5	66 kDa	2.29	0.02
Q9Y617	Phosphoserine aminotransferase	PSAT1	40 kDa	2.00	0.03
P48960	CD97 antigen	CD97	92 kDa	0.45	0.00
Q9Y5B8	Nucleoside diphosphate kinase 7	NME7	42 kDa	0.42	0.00
Q9Y248	DNA replication complex GINS protein PSF2	GINS2	21 kDa	0.45	0.01
Q8IZH2	5'-3' exoribonuclease 1	XRN1	194 kDa	0.56	0.00
Q9HAV4	Exportin-5	XPO5	136 kDa	1.86	0.04
Q9H5N1	Rab GTPase-binding effector protein 2	RABEP2	64 kDa	0.62	0.02
P09382	Galectin-1	LGALS1	15 kDa	2.17	0.01
P52657	Transcription initiation factor IIA subunit 2	GTF2A2	12 kDa	0.62	0.02
J3KMX3	Alpha-fetoprotein	AFP	70 kDa	0.45	0.00
O00483	NADH dehydrogenase [ubiquinone] 1 alpha subcomplex subunit 4	NDUFA4	9 kDa	0.50	0.00
P08195	Cluster of Isoform 2 of 4F2 cell-surface antigen heavy chain	SLC3A2	58 kDa	2.65	0.01
Q99623	Cluster of Prohibitin-2	PHB2	33 kDa	1.91	0.03
Q16254	Transcription factor E2F4	E2F4	44 kDa	0.39	0.00
H0Y8C6	Importin-5 (Fragment)	IPO5	124 kDa	2.20	0.01
P52564	Cluster of Dual specificity mitogen-activated protein kinase kinase 6	MAP2K6	37 kDa	0.52	0.00
B4DII8	Cluster of Gamma-secretase C-terminal fragment 57	APP	85 kDa	2.19	0.01
P07858	Cathepsin B	CTSB	38 kDa	1.75	0.02
G8JLQ3	Biogenesis of lysosome-related organelles complex 1 subunit 1	BLOC1S1	9 kDa	0.55	0.00
P34932	Heat shock 70 kDa protein 4	HSPA4	94 kDa	2.21	0.02

Q9Y490	Cluster of Talin-1	TLN1	270 kDa	2.21	0.02
P38646	Cluster of Stress-70 protein, mitochondrial	HSPA9	74 kDa	2.21	0.02
Q13505	Cluster of Metaxin-1	MTX1	51 kDa	0.53	0.01
Q9UP83	Conserved oligomeric Golgi complex subunit 5	COG5	93 kDa	0.62	0.02
B4E1F0	Plasma protease C1 inhibitor	SERPING1	56 kDa	0.44	0.00
P11216	Cluster of Glycogen phosphorylase, brain form	PYGB	97 kDa	3.88	0.00
P00558	Cluster of Phosphoglycerate kinase 1	PGK1	45 kDa	2.58	0.01
Q14697	Cluster of Neutral alpha-glucosidase AB	GANAB	107 kDa	2.58	0.01
F5H5U7	Cluster of Coiled-coil domain-containing protein 132	CCDC132	108 kDa	0.52	0.00
Q8N3E9	1-phosphatidylinositol 4,5-bisphosphate phosphodiesterase delta-3	PLCD3	89 kDa	0.40	0.00
P21333	Cluster of Filamin-A	FLNA	281 kDa	4.00	0.00
Q92900	Regulator of nonsense transcripts 1	UPF1	124 kDa	1.79	0.02
G3XAH6	Cluster of Poly(A) polymerase alpha	PAPOLA	81 kDa	2.46	0.01
Q9NW82	WD repeat-containing protein 70	WDR70	73 kDa	1.70	0.05
Q4G176	Cluster of Acyl-CoA synthetase family member 3, mitochondrial	ACSF3	64 kDa	0.60	0.04
Q9Y6R7	IgGFc-binding protein	FCGBP	572 kDa	0.61	0.02
J3QQZ1	Sodium channel protein type 4 subunit alpha	SCN4A	208 kDa	0.25	0.00
P46777	60S ribosomal protein L5	RPL5	34 kDa	2.00	0.01
Q13509	Cluster of Tubulin beta-3 chain	TUBB3	50 kDa	2.86	0.01
Q99879	Cluster of Histone H2B type 1-M	HIST1H2BM	14 kDa	4.20	0.01
P13667	Cluster of Protein disulfide-isomerase A4	PDIA4	73 kDa	2.10	0.02
B4E1K7	Cluster of Stomatin-like protein 2	STOML2	33 kDa	0.48	0.00
P19404	NADH dehydrogenase [ubiquinone] flavoprotein 2, mitochondrial	NDUFV2	27 kDa	0.34	0.00
Q7L2H7	Eukaryotic translation initiation factor 3 subunit M	EIF3M	43 kDa	5.25	0.00
P41091	Eukaryotic translation initiation factor 2 subunit 3	EIF2S3	51 kDa	2.11	0.02
Q96G25	Mediator of RNA polymerase II transcription subunit 8	MED8	29 kDa	0.58	0.01
Q06830	Peroxiredoxin-1	PRDX1	22 kDa	2.36	0.02
J3QRV5	Lethal(2) giant larvae protein homolog 2	LLGL2	113 kDa	0.47	0.00
I3L3I4	Cluster of Actin, cytoplasmic 2	ACTG1	42 kDa	4.13	0.00
H3BRY3	Coronin	CORO1A	43 kDa	0.49	0.00
Q92598	Cluster of Heat shock protein 105 kDa	HSPH1	97 kDa	3.00	0.01
J3QT38	Cluster of Exocyst complex component 6B	EXOC6B	79 kDa	0.57	0.01
J3KR97	Cluster of Tubulin-specific chaperone D	TBCD	137 kDa	2.00	0.02
H0YFI1	Ragulator complex protein LAMTOR1 (Fragment)	LAMTOR1	9 kDa	0.59	0.00
E9PIL6	Cluster of Lysine-specific demethylase 2A	KDM2A	82 kDa	0.39	0.00
Q06203	Amidophosphoribosyltransferase	PPAT	57 kDa	1.96	0.01
J3KTL2	Cluster of Serine/arginine-rich-splicing factor 1	SRSF1	28 kDa	2.14	0.02
P09972	Cluster of Fructose-bisphosphate aldolase C	ALDOC	39 kDa	2.17	0.02
O15144	Cluster of Actin-related protein 2/3 complex subunit 2	ARPC2	34 kDa	1.77	0.03
P29144	Cluster of Tripeptidyl-peptidase 2	TPP2	138 kDa	1.63	0.02
Q86UP2	Cluster of Kinectin	KTN1	156 kDa	1.77	0.03
B1AMW1	CD58 antigen, (Lymphocyte function-associated antigen 3), isoform CRA_c	CD58	27 kDa	0.44	0.00
Q8N1G2	Cap-specific mRNA (nucleoside-2'-O-)-methyltransferase 1	FTSJD2	95 kDa	3.00	0.01
Q53GQ0	Estradiol 17-beta-dehydrogenase 12	HSD17B12	34 kDa	0.44	0.00
Q7L576	Cluster of Cytoplasmic FMR1-interacting protein 1	CYFIP1	145 kDa	2.54	0.01
Q9BXJ9	Cluster of N-alpha-acetyltransferase 15, NatA auxiliary subunit	NAA15	101 kDa	2.14	0.02
P30153	Cluster of Serine/threonine-protein phosphatase 2A 65 kDa regulatory subunit A alpha isoform	PPP2R1A	65 kDa	1.88	0.01
Q9UL15	BAG family molecular chaperone regulator 5	BAG5	51 kDa	0.63	0.01
O60487	Myelin protein zero-like protein 2	MPZL2	24 kDa	0.42	0.00

F5H3A1	Cluster of Sodium/potassium-transporting ATPase subunit alpha-1	ATP1A1	113 kDa	1.67	0.04
P53680	AP-2 complex subunit sigma	AP2S1	17 kDa	0.60	0.01
Q86WR0	Coiled-coil domain-containing protein 25	CCDC25	24 kDa	0.60	0.01
Q8IY81	pre-rRNA processing protein FTSJ3	FTSJ3	97 kDa	0.60	0.01
Q9Y303	Isoform 3 of Putative N-acetylglucosamine-6-phosphate deacetylase	AMDHD2	64 kDa	0.43	0.00
Q9NXF7	DDB1- and CUL4-associated factor 16	DCAF16	24 kDa	0.58	0.00
Q14517	Cluster of Protocadherin Fat 1	FAT1	506 kDa	2.50	0.01
P28066	Cluster of Proteasome subunit alpha type-5	PSMA5	26 kDa	1.88	0.01
P02795	Cluster of Metallothionein-2	MT2A	6 kDa	0.63	0.01
Q9UBV2	Protein sel-1 homolog 1	SEL1L	89 kDa	0.60	0.01
Q9NSD9	Phenylalanine--tRNA ligase beta subunit	FARSB	66 kDa	1.75	0.02
Q15047	Histone-lysine N-methyltransferase SETDB1	SETDB1	143 kDa	0.63	0.01
G3V0F8	Cluster of Protein tyrosine phosphatase, non-receptor type 6, isoform CRA_e	PTPN6	70 kDa	1.92	0.02
O43242	Cluster of 26S proteasome non-ATPase regulatory subunit 3	PSMD3	61 kDa	2.46	0.01
P61586	Cluster of Transforming protein RhoA	RHOA	22 kDa	2.13	0.02
E7EQ61	Ubiquitin-like modifier-activating enzyme 5	UBA5	42 kDa	0.57	0.01
Q10570	Cleavage and polyadenylation specificity factor subunit 1	CPSF1	161 kDa	1.71	0.02
O60701	UDP-glucose 6-dehydrogenase	UGDH	55 kDa	1.86	0.02
P26640	Cluster of Valine--tRNA ligase	VARS	140 kDa	2.27	0.01
Q9Y3D6	Mitochondrial fission 1 protein	FIS1	17 kDa	0.44	0.01
O00629	Cluster of Importin subunit alpha-4	KPNA4	58 kDa	1.86	0.01
O75822	Cluster of Eukaryotic translation initiation factor 3 subunit	EIF3J	29 kDa	0.50	0.00
O60493	Sorting nexin-3	SNX3	19 kDa	0.48	0.00
Q6P1K2	Isoform 4 of Polyamine-modulated factor 1	PMF1	19 kDa	0.55	0.00
D6RH31	Nephronectin (Fragment)	NPNT	67 kDa	0.54	0.00
O00170	AH receptor-interacting protein	AIP	38 kDa	1.61	0.02
Q8TD19	Serine/threonine-protein kinase Nek9	NEK9	107 kDa	1.93	0.02
O60506	Cluster of Isoform 3 of Heterogeneous nuclear ribonucleoprotein Q	SYNCRIP	63 kDa	5.80	0.00
Q15063	Periostin	POSTN	93 kDa	0.49	0.01
P49366	Deoxyhypusine synthase	DHPS	41 kDa	0.63	0.01
D6RDX8	Tetraspanin-17	TSPAN17	34 kDa	0.47	0.00
J3KPU0	[Pyruvate dehydrogenase [acetyl-transferring]]-phosphatase 1, mitochondrial	PDP1	64 kDa	0.46	0.01
Q99536	Synaptic vesicle membrane protein VAT-1 homolog	VAT1	42 kDa	1.94	0.01
P06213	Insulin receptor	INSR	156 kDa	0.45	0.00
Q15542	Transcription initiation factor TFIID subunit 5	TAF5	87 kDa	0.51	0.00
P60981	Destrin	DSTN	19 kDa	1.68	0.03
H7C5I8	Cluster of Down syndrome critical region protein 3 (Fragment)	DSCR3	9 kDa	0.24	0.00
Q8IYI6	Exocyst complex component 8	EXOC8	82 kDa	0.60	0.01
Q9BXS4	Cluster of Transmembrane protein 59	TMEM59	36 kDa	0.56	0.00
P57735	Ras-related protein Rab-25	RAB25	23 kDa	0.43	0.00
Q08AM6	Cluster of Protein VAC14 homolog	VAC14	88 kDa	1.96	0.03
Q7L7X3	Cluster of Serine/threonine-protein kinase TAO1	TAOK1	116 kDa	0.49	0.00
A6NCK0	Cluster of NEDD8-activating enzyme E1 regulatory subunit	NAE1	61 kDa	1.97	0.02
Q9NRX5	Serine incorporator 1	SERINC1	50 kDa	0.55	0.02
E9PSI1	Cluster of Transmembrane 9 superfamily member 1	TM9SF1	92 kDa	0.56	0.01
Q14315	Filamin-C	FLNC	291 kDa	2.00	0.02
Q15554	Telomeric repeat-binding factor 2	TERF2	56 kDa	0.51	0.00
Q86U86	Protein polybromo-1	PBRM1	193 kDa	0.53	0.00
P10253	Lysosomal alpha-glucosidase	GAA	105 kDa	1.88	0.02

O00217	NADH dehydrogenase [ubiquinone] iron-sulfur protein 8, mitochondrial	NDUFS8	24 kDa	0.56	0.02
O00391	Cluster of Sulfhydryl oxidase 1	QSOX1	83 kDa	1.95	0.02
H0YGR4	Oligoribonuclease, mitochondrial (Fragment)	REXO2	22 kDa	2.03	0.01
P51665	Cluster of 26S proteasome non-ATPase regulatory subunit 7	PSMD7	37 kDa	1.81	0.01
Q9BQI0	Allograft inflammatory factor 1-like	AIF1L	17 kDa	0.53	0.00
B1AHL2	Cluster of Fibulin 1	FBLN1	78 kDa	2.82	0.01
P49321	Cluster of Nuclear autoantigenic sperm protein	NASP	85 kDa	2.38	0.01
E9PH42	Receptor-type tyrosine-protein phosphatase U	PTPRU	162 kDa	0.57	0.03
Q8TF42	Ubiquitin-associated and SH3 domain-containing protein B	UBASH3B	73 kDa	0.36	0.00
P46100	Transcriptional regulator ATRX	ATRX	283 kDa	0.25	0.00
J3KP07	Cluster of Growth arrest-specific protein 6	GAS6	80 kDa	2.08	0.01
P07900	Cluster of Heat shock protein HSP 90-alpha	HSP90AA1	85 kDa	0.19	0.00
O75369	Cluster of Isoform 8 of Filamin-B	FLNB	282 kDa	2.06	0.02
P23434	Glycine cleavage system H protein, mitochondrial	GCSH	19 kDa	0.40	0.00
O95479	GDH/6PGL endoplasmic bifunctional protein	H6PD	89 kDa	0.41	0.00
Q9BRZ2	E3 ubiquitin-protein ligase TRIM56	TRIM56	81 kDa	0.41	0.00
B9ZVT1	RNA-binding protein 12B	RBM12B	103 kDa	0.62	0.02
P49591	Serine--tRNA ligase, cytoplasmic	SARS	59 kDa	2.00	0.01
O00154	Cluster of Cytosolic acyl coenzyme A thioester hydrolase	ACOT7	42 kDa	1.89	0.02
P05556	Cluster of Integrin beta-1	ITGB1	88 kDa	1.70	0.01
Q01082	Cluster of Spectrin beta chain, non-erythrocytic 1	SPTBN1	275 kDa	2.83	0.00
Q9NQP4	Prefoldin subunit 4	PFDN4	15 kDa	1.70	0.04
Q6GMV3	Putative peptidyl-tRNA hydrolase PTRHD1	PTRHD1	16 kDa	0.53	0.00
Q96F24	Nuclear receptor-binding factor 2	NRBF2	32 kDa	0.50	0.00
P54136	Arginine--tRNA ligase, cytoplasmic	RARS	75 kDa	1.89	0.02
P01023	Cluster of Alpha-2-macroglobulin	A2M	163 kDa	2.43	0.01
B3KS98	Cluster of Eukaryotic translation initiation factor 3 subunit H	EIF3H	42 kDa	3.21	0.00
F8VRQ1	Cluster of Heterogeneous nuclear ribonucleoprotein A1	HNRNPA1	33 kDa	1.73	0.02
P08758	Cluster of Annexin A5	ANXA5	36 kDa	1.88	0.01
Q04721	Cluster of Neurogenic locus notch homolog protein 2	NOTCH2	265 kDa	1.92	0.02
P52888	Thimet oligopeptidase	THOP1	79 kDa	1.92	0.01
Q14563	Semaphorin-3A	SEMA3A	89 kDa	1.92	0.01
B4DQH9	Ran-binding protein 10	RANBP10	65 kDa	0.59	0.00
A6NHG4	D-dopachrome decarboxylase-like protein	DDTL	14 kDa	0.59	0.01
E9PN76	RING finger protein 214	RNF214	58 kDa	0.27	0.00
P14314	Glucosidase 2 subunit beta	PRKCSH	59 kDa	2.05	0.01
O75882	Cluster of Isoform 2 of Attractin	ATRN	141 kDa	1.86	0.02
B1AKJ5	Nardilysin	NRD1	139 kDa	1.91	0.01
Q15758	Neutral amino acid transporter B(0)	SLC1A5	57 kDa	1.68	0.03
O75367	Core histone macro-H2A.1	H2AFY	40 kDa	1.68	0.01
P16870	Cluster of Carboxypeptidase E	CPE	53 kDa	2.19	0.01
P17980	Cluster of 26S protease regulatory subunit 6A	PSMC3	49 kDa	2.04	0.01
P35579	Cluster of Myosin-9	MYH9	227 kDa	3.20	0.00
J3KQJ1	Cluster of Sulfatase-modifying factor 2	SUMF2	36 kDa	0.36	0.00
Q9UKI9	Cluster of Isoform 2 of POU domain, class 2, transcription factor 3	POU2F3	48 kDa	0.55	0.01
B3KPE7	Bcl-2-like protein 13	BCL2L13	35 kDa	0.43	0.00
B4DR87	Cluster of Procollagen-lysine,2-oxoglutarate 5-dioxygenase 1	PLOD1	88 kDa	2.67	0.00
Q9UII2	ATPase inhibitor, mitochondrial	ATPIF1	12 kDa	0.62	0.01
Q5F1R6	Isoform 2 of DnaJ homolog subfamily C member 21	DNAJC21	67 kDa	0.49	0.00
P25786	Cluster of Proteasome subunit alpha type-1	PSMA1	30 kDa	1.75	0.01
Q9HB07	Cluster of UPF0160 protein MYG1, mitochondrial	C12orf10	42 kDa	0.59	0.00

P07339	Cluster of Cathepsin D	CTSD	45 kDa	6.75	0.00
Q10567	Cluster of Isoform C of AP-1 complex subunit beta-1	AP1B1	104 kDa	2.25	0.00
Q9Y224	Cluster of UPF0568 protein C14orf166	C14orf166	28 kDa	2.25	0.00
P14735	Insulin-degrading enzyme	IDE	118 kDa	1.67	0.03
P16930	Fumarylacetoacetase	FAH	46 kDa	0.54	0.01
P28482	Cluster of Mitogen-activated protein kinase 1	MAPK1	41 kDa	1.82	0.02
Q5R372	Rab GTPase-activating protein 1-like	RABGAP1L	93 kDa	0.63	0.01
P47895	Cluster of Aldehyde dehydrogenase family 1 member A3	ALDH1A3	56 kDa	1.67	0.01
Q9NQX3	Cluster of Gephyrin	GPHN	80 kDa	1.79	0.02
E7ESY4	Cluster of Metastasis-associated protein MTA1	MTA1	79 kDa	1.65	0.02
P49588	Cluster of Alanine--tRNA ligase, cytoplasmic	AARS	107 kDa	1.95	0.01
O15126	Secretory carrier-associated membrane protein 1	SCAMP1	38 kDa	0.60	0.00
B4DYH8	Cluster of N-acetylglucosamine-6-sulfatase	GNS	60 kDa	1.75	0.02
A1A4S6	Rho GTPase-activating protein 10	ARHGAP10	89 kDa	0.40	0.00
Q9UN36	Protein NDRG2	NDRG2	41 kDa	0.45	0.00
Q8WZA9	Immunity-related GTPase family Q protein	IRGQ	63 kDa	0.63	0.01
Q96CB8	Cluster of Integrator complex subunit 12	INTS12	49 kDa	0.60	0.00
Q56VL3	OCIA domain-containing protein 2	OCIAD2	17 kDa	0.57	0.00
P17812	CTP synthase 1	CTPS1	67 kDa	1.85	0.01
Q15853	Upstream stimulatory factor 2	USF2	37 kDa	0.33	0.00
Q5T4S7	Cluster of Isoform 2 of E3 ubiquitin-protein ligase UBR4	UBR4	576 kDa	2.40	0.00
Q93052	Lipoma-preferred partner	LPP	66 kDa	0.40	0.00
P05783	Cluster of Keratin, type I cytoskeletal 18	KRT18	48 kDa	1.71	0.02
Q9BV38	WD repeat-containing protein 18	WDR18	47 kDa	0.55	0.00
Q15738	Sterol-4-alpha-carboxylate 3-dehydrogenase, decarboxylating	NSDHL	42 kDa	0.46	0.00
J3KQ83	ATP synthase-coupling factor 6, mitochondrial	ATP5J	13 kDa	0.60	0.00
B4DKD1	Protein tweety homolog 2	TTYH2	56 kDa	0.51	0.00
P63244	Cluster of Guanine nucleotide-binding protein subunit beta-2-like 1	GNB2L1	35 kDa	1.73	0.02
E9PCA1	T-complex protein 1 subunit epsilon	CCT5	57 kDa	2.11	0.01
H0YG22	DNA-binding protein A (Fragment)	CSDA	19 kDa	0.61	0.01
P62979	Cluster of Ubiquitin-40S ribosomal protein S27a	RPS27A	18 kDa	1.73	0.02
P50851	Cluster of Lipopolysaccharide-responsive and beige-like anchor protein	LRBA	319 kDa	1.73	0.02
O75436	Cluster of Vacuolar protein sorting-associated protein 26A	VPS26A	38 kDa	1.73	0.02
Q9BV19	Uncharacterized protein C1orf50	C1orf50	22 kDa	0.43	0.00
P07602	Isoform Sap-mu-9 of Proactivator polypeptide	PSAP	58 kDa	9.50	0.00
Q9Y4L1	Cluster of Hypoxia up-regulated protein 1	HYOU1	111 kDa	2.38	0.00
O00468	Cluster of Agrin	AGRN	215 kDa	2.38	0.00
E7EU23	Cluster of Rab GDP dissociation inhibitor beta	GDI2	51 kDa	2.38	0.00
B4DW92	Nuclear receptor 2C2-associated protein	NR2C2AP	19 kDa	0.33	0.00
J3KPM9	Signal transducer and activator of transcription 1-alpha/beta	STAT1	83 kDa	1.78	0.01
E7ETZ4	Basic leucine zipper and W2 domain-containing protein 2 (Fragment)	BZW2	47 kDa	2.32	0.01
D6REB5	Alpha-L-iduronidase	IDUA	58 kDa	0.63	0.04
O95084	Serine protease 23	PRSS23	43 kDa	0.56	0.00
Q9BXS5	AP-1 complex subunit mu-1	AP1M1	49 kDa	1.61	0.01
P00367	Cluster of Glutamate dehydrogenase 1, mitochondrial	GLUD1	61 kDa	1.61	0.01
P02768	Cluster of Serum albumin	ALB	69 kDa	1.67	0.02
E9PEI9	Lanosterol synthase	LSS	82 kDa	0.34	0.00
O43837	Isocitrate dehydrogenase [NAD] subunit beta,	IDH3B	42 kDa	0.38	0.00
G8JLD5	Cluster of Dynamin-1-like protein	DNM1L	80 kDa	1.88	0.01
O76094	Cluster of Signal recognition particle 72 kDa protein	SRP72	75 kDa	1.61	0.01
Q9P258	Protein RCC2	RCC2	56 kDa	1.88	0.02
O75368	SH3 domain-binding glutamic acid-rich-like protein	SH3BGRL	13 kDa	0.56	0.01

Q9NZT2	Opioid growth factor receptor	OGFR	73 kDa	0.43	0.00
Q9NPF2	Carbohydrate sulfotransferase 11	CHST11	42 kDa	0.60	0.00
J3KR54	Isovaleryl Coenzyme A dehydrogenase, isoform CRA_a	IVD	47 kDa	0.47	0.00
J3KNT0	Cluster of Fascin	FSCN1	52 kDa	1.81	0.01
P0C0S5	Cluster of Histone H2A.Z	H2AFZ	14 kDa	1.81	0.01
P28070	Proteasome subunit beta type-4	PSMB4	29 kDa	1.95	0.01
A6NN98	Myotubularin related protein 2, isoform CRA_a	MTMR2	66 kDa	0.55	0.00
P11387	Cluster of DNA topoisomerase 1	TOP1	91 kDa	2.00	0.01
Q7Z4V5	Cluster of Hepatoma-derived growth factor-related protein	HDGFRP2	74 kDa	1.83	0.02
Q8TDN6	Ribosome biogenesis protein BRX1 homolog	BRIX1	41 kDa	0.47	0.00
P61020	Cluster of Ras-related protein Rab-5B	RAB5B	24 kDa	0.61	0.00
Q14258	E3 ubiquitin/ISG15 ligase TRIM25	TRIM25	71 kDa	1.83	0.01
Q9C0D3	Protein zyg-11 homolog B	ZYG11B	84 kDa	0.55	0.00
Q96F86	Cluster of Enhancer of mRNA-decapping protein 3	EDC3	56 kDa	0.56	0.01
Q15046	Cluster of Lysine--tRNA ligase	KARS	68 kDa	1.64	0.02
Q9NPJ6	Mediator of RNA polymerase II transcription subunit 4	MED4	30 kDa	0.62	0.00
Q13435	Cluster of Splicing factor 3B subunit 2	SF3B2	100 kDa	1.80	0.01
Q69YN2	CWF19-like protein 1	CWF19L1	61 kDa	0.56	0.00
P55884	Eukaryotic translation initiation factor 3 subunit B	EIF3B	92 kDa	3.71	0.00
P62308	Cluster of Small nuclear ribonucleoprotein G	SNRPG	8 kDa	1.96	0.00
E7EN22	Ubiquitin carboxyl-terminal hydrolase	USP19	151 kDa	0.60	0.02
P08727	Cluster of Keratin, type I cytoskeletal 19	KRT19	44 kDa	2.21	0.01
Q9NV70	Isoform 2 of Exocyst complex component 1	EXOC1	100 kDa	0.52	0.00
Q15654	Cluster of Thyroid receptor-interacting protein 6	TRIP6	50 kDa	0.62	0.00
Q01995	Cluster of Transgelin	TAGLN	23 kDa	0.53	0.00
B4DGU4	Cluster of Catenin beta-1	CTNNB1	85 kDa	1.94	0.00
P50454	Cluster of Serpin H1	SERPINH1	46 kDa	1.72	0.01
Q99590	Protein SCAF11	SCAF11	165 kDa	0.50	0.00
Q9P2X0	Dolichol-phosphate mannosyltransferase subunit 3	DPM3	10 kDa	0.42	0.00
E9PC67	ATP-binding cassette sub-family F member 1	ABCF1	60 kDa	0.60	0.00
F5H7N9	Cluster of Lactadherin	MFGE8	42 kDa	1.82	0.01
Q92896	Cluster of Isoform 3 of Golgi apparatus protein 1	GLG1	136 kDa	2.58	0.00
Q9H9S4	Calcium-binding protein 39-like	CAB39L	39 kDa	1.68	0.02
P51149	Cluster of Ras-related protein Rab-7a	RAB7A	23 kDa	1.91	0.01
B1AK87	Cluster of Capping protein (Actin filament) muscle Z-line, beta	CAPZB	29 kDa	3.50	0.00
Q8TEM1	Nuclear pore membrane glycoprotein 210	NUP210	205 kDa	0.51	0.00
P20700	Lamin-B1	LMNB1	66 kDa	2.05	0.01
Q9BXP5	Serrate RNA effector molecule homolog	SRRT	101 kDa	1.71	0.01
H0YE28	Protein C11orf31 (Fragment)	C11orf31	10 kDa	0.63	0.00
Q8N163	Cluster of DBIRD complex subunit KIAA1967	KIAA1967	103 kDa	2.04	0.00
Q92499	ATP-dependent RNA helicase DDX1	DDX1	82 kDa	3.00	0.00
C9JKL2	MLN64 N-terminal domain homolog	STARD3NL	25 kDa	0.48	0.00
Q13867	Cluster of Bleomycin hydrolase	BLMH	53 kDa	2.00	0.00
P37235	Cluster of Hippocalcin-like protein 1	HPCAL1	22 kDa	1.60	0.02
D6REX5	Selenoprotein P (Fragment)	SEPP1	35 kDa	0.61	0.02
Q8TEB1	Cluster of DDB1- and CUL4-associated factor 11	DCAF11	62 kDa	0.59	0.01
Q9BRT6	Protein LLP homolog	LLPH	15 kDa	0.43	0.00
I3L1I0	Glyoxalase domain-containing protein 4 (Fragment)	GLOD4	13 kDa	0.22	0.00
P49327	Fatty acid synthase	FASN	273 kDa	2.13	0.00
P11413	Cluster of Isoform 3 of Glucose-6-phosphate 1-dehydrogenase	G6PD	62 kDa	1.79	0.01
Q96AG4	Leucine-rich repeat-containing protein 59	LRRC59	35 kDa	1.79	0.01
P29317	Cluster of Ephrin type-A receptor 2	EPHA2	108 kDa	1.95	0.01
Q13618	Cluster of Cullin-3	CUL3	89 kDa	1.65	0.01
D6RGG3	Cluster of Collagen alpha-1(XII) chain	COL12A1	333 kDa	2.00	0.01

B4DQJ8	6-phosphogluconate dehydrogenase, decarboxylating	PGD	52 kDa	2.00	0.01
Q96IJ6	Mannose-1-phosphate guanyltransferase alpha	GMPPA	46 kDa	0.58	0.00
O95619	YEATS domain-containing protein 4	YEATS4	27 kDa	0.36	0.00
F5H4E4	Ketosamine-3-kinase	FN3KRP	29 kDa	0.61	0.00
O75955	Flotillin-1	FLOT1	47 kDa	0.38	0.00
Q13043	Cluster of Serine/threonine-protein kinase 4	STK4	56 kDa	0.34	0.00
Q99832	Cluster of T-complex protein 1 subunit eta	CCT7	59 kDa	2.33	0.00
B5MCF9	Cluster of Pescadillo homolog	PES1	66 kDa	2.41	0.00
F5H293	Seizure 6-like protein 2	SEZ6L2	94 kDa	0.55	0.00
O00231	Cluster of 26S proteasome non-ATPase regulatory subunit 11	PSMD11	47 kDa	2.36	0.00
P48444	Cluster of Coatomer subunit delta	ARCN1	57 kDa	2.36	0.00
Q6YHK3	CD109 antigen	CD109	162 kDa	1.83	0.01
Q9UQ80	Cluster of Proliferation-associated protein 2G4	PA2G4	44 kDa	1.83	0.01
G8JLK7	Nucleolar protein 6	NOL6	122 kDa	0.52	0.00
Q9Y3B2	Exosome complex component CSL4	EXOSC1	21 kDa	0.61	0.00
C9J2P0	Cluster of Ubiquitin-conjugating enzyme E2 E1 (Fragment)	UBE2E1	16 kDa	0.61	0.00
Q6VY07	Cluster of Phosphofurin acidic cluster sorting protein 1	PACS1	105 kDa	0.57	0.00
Q9NXR1	Cluster of Isoform 2 of Nuclear distribution protein nudE homolog 1	NDE1	38 kDa	0.53	0.00
Q9BWM7	Sideroflexin-3	SFXN3	36 kDa	0.47	0.00
P04083	Annexin A1	ANXA1	39 kDa	2.75	0.00
Q99988	Growth/differentiation factor 15	GDF15	34 kDa	2.75	0.00
B4DZH6	Histone deacetylase 6	HDAC6	133 kDa	0.57	0.01
Q9GZX3	Cluster of Carbohydrate sulfotransferase 6	CHST6	44 kDa	0.61	0.00
G3V5Z7	Cluster of Proteasome subunit alpha type	PSMA6	28 kDa	2.00	0.00
Q99436	Proteasome subunit beta type-7	PSMB7	30 kDa	1.82	0.01
P13726	Tissue factor	F3	33 kDa	0.49	0.00
P09874	Poly [ADP-ribose] polymerase 1	PARP1	113 kDa	2.50	0.00
Q8IWV7	Cluster of E3 ubiquitin-protein ligase UBR1	UBR1	200 kDa	1.60	0.01
Q9NW13	Cluster of RNA-binding protein 28	RBM28	86 kDa	1.66	0.03
Q92905	COP9 signalosome complex subunit 5	COPS5	38 kDa	1.77	0.01
A9UHW6	MIF4G domain-containing protein	MIF4GD	25 kDa	0.62	0.00
P14866	Heterogeneous nuclear ribonucleoprotein L	HNRNPL	64 kDa	2.65	0.00
Q9H269	Cluster of Vacuolar protein sorting-associated protein 16 homolog	VPS16	95 kDa	0.35	0.00
C9JW96	Prohibitin (Fragment)	PHB	27 kDa	1.64	0.01
J3QQ67	60S ribosomal protein L18 (Fragment)	RPL18	22 kDa	1.64	0.01
H7C2C7	Cluster of Negative elongation factor A (Fragment)	WHSC2	27 kDa	0.32	0.00
J3KPP7	Beta-arrestin-1	ARRB1	47 kDa	1.97	0.00
P62304	Small nuclear ribonucleoprotein E	SNRPE	11 kDa	0.60	0.01
Q14108	Lysosome membrane protein 2	SCARB2	54 kDa	0.53	0.00
F5H6C8	Mitochondrial antiviral-signaling protein	MAVS	40 kDa	0.26	0.00
B7Z5Z2	Ras-related protein R-Ras2	RRAS2	24 kDa	0.63	0.01
P51693	Amyloid-like protein 1	APLP1	72 kDa	0.63	0.01
B8X2Z3	CARMIL2b	RLTPR	148 kDa	0.41	0.00
Q15043	Zinc transporter ZIP14	SLC39A14	54 kDa	0.60	0.00
F5GWX5	Cluster of Chromodomain-helicase-DNA-binding protein 4	CHD4	217 kDa	1.75	0.00
Q96A49	Synapse-associated protein 1	SYAP1	40 kDa	0.55	0.00
Q8NEW0	Zinc transporter 7	SLC30A7	42 kDa	0.53	0.00
Q9HBU6	Cluster of Ethanolamine kinase 1	ETNK1	51 kDa	0.52	0.00
Q9Y383	Cluster of Putative RNA-binding protein Luc7-like 2	LUC7L2	47 kDa	3.09	0.00
H0YES5	HBS1-like protein (Fragment)	HBS1L	19 kDa	0.51	0.00
H3BSA4	Na(+)/H(+) exchange regulatory cofactor NHE-RF2 (Fragment)	SLC9A3R2	19 kDa	0.56	0.00
P17301	Cluster of Integrin alpha-2	ITGA2	129 kDa	1.68	0.00

Q9BS26	Endoplasmic reticulum resident protein 44	ERP44	47 kDa	1.68	0.00
Q96SB3	Neurabin-2	PPP1R9B	89 kDa	0.58	0.00
Q9Y6A4	UPF0468 protein C16orf80	C16orf80	23 kDa	0.58	0.00
Q5JTD0	Tight junction-associated protein 1	TJAP1	62 kDa	0.49	0.00
Q8NFF5	Cluster of Isoform 2 of FAD synthase	FLAD1	54 kDa	0.37	0.00
P49757	Protein numb homolog	NUMB	71 kDa	0.21	0.05
E7EWE9	Kinetochore protein Spc24	SPC24	17 kDa	0.51	0.00
Q13751	Laminin subunit beta-3	LAMB3	130 kDa	1.73	0.01
P39748	Cluster of Flap endonuclease 1	FEN1	43 kDa	1.90	0.01
P84022	Cluster of Isoform 2 of Mothers against decapentaplegic homolog 3	SMAD3	43 kDa	0.32	0.00
G5E9W3	Cleavage and polyadenylation specific factor 3, 73kDa, isoform CRA_b	CPSF3	73 kDa	1.83	0.00
P22626	Heterogeneous nuclear ribonucleoproteins A2/B1	HNRNPA2B1	37 kDa	2.44	0.00
P53602	Diphosphomevalonate decarboxylase	MVD	43 kDa	1.69	0.01
B4E040	Ras-related protein Ral-B	RALB	26 kDa	0.63	0.00
H3BM91	COMM domain-containing protein 4 (Fragment)	COMMD4	23 kDa	0.59	0.00
O15230	Laminin subunit alpha-5	LAMA5	400 kDa	1.83	0.00
Q06210	Cluster of Glucosamine--fructose-6-phosphate aminotransferase [isomerizing] 1	GFPT1	79 kDa	2.20	0.00
P16035	Metalloproteinase inhibitor 2	TIMP2	24 kDa	1.68	0.01
H0YBQ0	Thioredoxin reductase 3 (Fragment)	TXNRD3	76 kDa	0.50	0.00
Q5VZE5	N-alpha-acetyltransferase 35, NatC auxiliary subunit	NAA35	84 kDa	0.51	0.00
G5EA42	Cluster of Tropomodulin 2 (Neuronal), isoform CRA_a	TMOD2	34 kDa	0.21	0.00
E9PKG1	Cluster of Protein arginine N-methyltransferase 1	PRMT1	38 kDa	2.31	0.00
Q06481	Cluster of Amyloid-like protein 2	APLP2	87 kDa	1.85	0.00
Q9H9T3	Elongator complex protein 3	ELP3	62 kDa	0.62	0.00
Q9Y3C1	Cluster of Nucleolar protein 16	NOP16	21 kDa	0.62	0.00
E9PDI4	Ladinin-1	LAD1	59 kDa	0.54	0.00
Q9ULJ6	Cluster of Zinc finger MIZ domain-containing protein 1	ZMIZ1	115 kDa	0.56	0.01
Q9Y305	Acyl-coenzyme A thioesterase 9, mitochondrial	ACOT9	50 kDa	0.60	0.00
H3BLY1	Cluster of Contactin-associated protein-like 3B (Fragment)	CNTNAP3B	129 kDa	0.61	0.00
O75131	Copine-3	CPNE3	60 kDa	2.50	0.00
A7MAP0	Cluster of Coronin	CORO1C	54 kDa	2.08	0.00
B1Q2N1	Cluster of ATP-dependent RNA helicase DDX39A	DDX39	30 kDa	1.78	0.01
P27816	Cluster of Isoform 6 of Microtubule-associated protein 4	MAP4	120 kDa	1.78	0.01
A8MUM1	Cluster of Protein TSSC1	TSSC1	46 kDa	0.44	0.00
Q9NQT5	Exosome complex component RRP40	EXOSC3	30 kDa	0.62	0.00
B7WP27	Pre-mRNA-splicing factor CWC22 homolog	CWC22	106 kDa	0.53	0.00
Q9P2J5	Leucine--tRNA ligase, cytoplasmic	LARS	134 kDa	1.78	0.01
Q96DA6	Mitochondrial import inner membrane translocase subunit TIM14	DNAJC19	12 kDa	0.52	0.00
J3KR78	Queuine tRNA-ribosyltransferase subunit QTRTD1	QTRTD1	48 kDa	0.46	0.00
Q04760	Lactoylglutathione lyase	GLO1	21 kDa	0.62	0.00
Q96IZ0	PRKC apoptosis WT1 regulator protein	PAWR	37 kDa	0.57	0.00
P36404	ADP-ribosylation factor-like protein 2	ARL2	21 kDa	0.53	0.00
Q9H993	UPF0364 protein C6orf211	C6orf211	51 kDa	0.30	0.00
Q9BYN0	Sulfiredoxin-1	SRXN1	14 kDa	0.55	0.00
H0Y626	Uncharacterized protein		108 kDa	0.55	0.00
O75449	Cluster of Katanin p60 ATPase-containing subunit A1	KATNA1	56 kDa	0.46	0.00
Q49AR2	UPF0489 protein C5orf22	C5orf22	50 kDa	0.43	0.00
C9JWR9	3-phosphoinositide-dependent protein kinase 1	PDPK1	61 kDa	0.41	0.00
Q8IYB8	ATP-dependent RNA helicase SUPV3L1, mitochondrial	SUPV3L1	88 kDa	0.47	0.00
P10619	Lysosomal protective protein	CTSA	54 kDa	0.26	0.00
Q8TEQ6	Gem-associated protein 5	GEMIN5	169 kDa	2.33	0.00

P62249	40S ribosomal protein S16	RPS16	16 kDa	2.46	0.00
Q9NWY4	UPF0609 protein C4orf27	C4orf27	39 kDa	0.61	0.00
F8W785	Golgi integral membrane protein 4	GOLIM4	79 kDa	0.32	0.00
P49368	Cluster of T-complex protein 1 subunit gamma	CCT3	61 kDa	3.25	0.00
P67809	Cluster of Nuclease-sensitive element-binding protein 1	YBX1	36 kDa	1.63	0.00
Q9HB71	Calcyclin-binding protein	CACYBP	26 kDa	1.63	0.00
O60841	Eukaryotic translation initiation factor 5B	EIF5B	139 kDa	1.77	0.01
Q9NR30	Cluster of Nucleolar RNA helicase 2	DDX21	87 kDa	1.77	0.01
Q96FJ0	AMSH-like protease	STAMBPL1	50 kDa	0.43	0.00
Q14232	Cluster of Translation initiation factor eIF-2B subunit alpha	EIF2B1	34 kDa	1.63	0.00
A6NDW3	Cluster of Lipolysis-stimulated lipoprotein receptor	LSR	64 kDa	1.63	0.00
Q96NB3	Zinc finger protein 830	ZNF830	42 kDa	0.56	0.00
Q9UBS3	DnaJ homolog subfamily B member 9	DNAJB9	26 kDa	0.51	0.00
Q5TEC6	Cluster of Histone H3	HIST2H3PS2	15 kDa	2.40	0.00
P50502	Cluster of Hsc70-interacting protein	ST13	41 kDa	3.00	0.00
Q3ZCQ8	Isoform 2 of Mitochondrial import inner membrane translocase subunit TIM50	TIMM50	50 kDa	0.57	0.00
P24666	Low molecular weight phosphotyrosine protein phosphatase	ACP1	18 kDa	0.63	0.00
Q9UBW8	COP9 signalosome complex subunit 7a	COPS7A	30 kDa	1.69	0.00
P45974	Ubiquitin carboxyl-terminal hydrolase 5	USP5	96 kDa	1.91	0.00
Q15785	Mitochondrial import receptor subunit TOM34	TOMM34	35 kDa	0.44	0.00
J3KR35	Coiled-coil domain containing 12, isoform CRA_a	CCDC12	21 kDa	0.36	0.00
Q8N7H5	RNA polymerase II-associated factor 1 homolog	PAF1	60 kDa	2.00	0.00
Q96KP4	Cluster of Cytosolic non-specific dipeptidase	CNDP2	53 kDa	1.71	0.01
J3KMX4	Ragulator complex protein LAMTOR3	LAMTOR3	13 kDa	0.62	0.02
Q12974	Cluster of Protein tyrosine phosphatase type IVA 2	PTP4A2	19 kDa	0.62	0.01
P78527	DNA-dependent protein kinase catalytic subunit	PRKDC	469 kDa	1.86	0.00
Q9BQC3	Diphthamide biosynthesis protein 2	DPH2	52 kDa	0.48	0.00
F5H6J9	Activating signal cointegrator 1 complex subunit 2	ASCC2	77 kDa	0.46	0.00
Q9NZV1	Cysteine-rich motor neuron 1 protein	CRIM1	114 kDa	1.71	0.00
H3BM74	NEDD8 ultimate buster 1	NUB1	73 kDa	0.57	0.00
F5H897	Cluster of Heat shock protein 75 kDa, mitochondrial	TRAP1	74 kDa	1.71	0.00
Q9P287	Cluster of BRCA2 and CDKN1A-interacting protein	BCCIP	36 kDa	2.50	0.00
O75390	Cluster of Citrate synthase, mitochondrial	CS	52 kDa	2.12	0.00
O43353	Cluster of Receptor-interacting serine/threonine-protein kinase 2	RIPK2	61 kDa	0.53	0.00
G5E934	Cluster of Cancer susceptibility candidate 4, isoform CRA_b	CASC4	49 kDa	0.62	0.00
Q9C0B0	RING finger protein unkempt homolog	UNK	88 kDa	0.51	0.00
Q14978	Isoform Beta of Nucleolar and coiled-body phosphoprotein 1	NOLC1	75 kDa	2.00	0.00
Q14974	Cluster of Importin subunit beta-1	KPNB1	97 kDa	2.00	0.00
P30050	60S ribosomal protein L12	RPL12	18 kDa	1.64	0.01
A8MZ87	Cluster of Kinesin light chain 2	KLC2	57 kDa	1.64	0.01
Q96FZ7	Cluster of Charged multivesicular body protein 6	CHMP6	23 kDa	0.53	0.00
Q4G0F5	Vacuolar protein sorting-associated protein 26B	VPS26B	39 kDa	0.62	0.00
P55196	Cluster of Isoform 2 of Afadin	MLLT4	206 kDa	0.58	0.00
F5GX65	Armadillo repeat-containing protein 10	ARMC10	25 kDa	0.55	0.00
H0YJ66	Dehydrogenase/reductase SDR family member 7 (Fragment)	DHRS7	45 kDa	0.51	0.00
A2RUC4	Cluster of tRNA wybutosine-synthesizing protein 5	TYW5	37 kDa	0.49	0.00
Q8N0X4	Citrate lyase subunit beta-like protein, mitochondrial	CLYBL	37 kDa	0.47	0.00
Q01432	AMP deaminase 3	AMPD3	89 kDa	0.53	0.00
Q8NFQ8	Torsin-1A-interacting protein 2	TOR1AIP2	51 kDa	0.36	0.00
P49589	Cluster of Isoform 3 of Cysteine--tRNA ligase, cytoplasmic	CARS	95 kDa	4.30	0.00
Q93034	Cluster of Cullin-5	CUL5	91 kDa	1.65	0.01

E9PLI6	Probable RNA-binding protein EIF1AD (Fragment)	EIF1AD	13 kDa	0.60	0.00
Q9BQP7	Uncharacterized protein C20orf72	C20orf72	39 kDa	0.50	0.00
P46940	Cluster of Ras GTPase-activating-like protein IQGAP1	IQGAP1	189 kDa	3.58	0.00
Q13347	Eukaryotic translation initiation factor 3 subunit I	EIF3I	37 kDa	1.79	0.00
Q9H6R7	WD repeat-containing protein C2orf44	C2orf44	79 kDa	0.49	0.00
P60953	Cluster of Cell division control protein 42 homolog	CDC42	21 kDa	1.65	0.01
P52272	Heterogeneous nuclear ribonucleoprotein M	HNRNPM	78 kDa	1.79	0.00
Q92614	Unconventional myosin-XVIIIa	MYO18A	233 kDa	1.61	0.00
Q16831	Cluster of Uridine phosphorylase 1	UPP1	34 kDa	1.61	0.00
E7EQY1	Cluster of Protein FAM136A	FAM136A	27 kDa	0.45	0.00
P46736	Isoform 1 of Lys-63-specific deubiquitinase BRCC36	BRCC3	33 kDa	0.62	0.00
P55011	Solute carrier family 12 member 2	SLC12A2	131 kDa	0.34	0.00
Q9UNS2	Cluster of Isoform 2 of COP9 signalosome complex subunit 3	COPS3	46 kDa	2.29	0.00
P06280	Alpha-galactosidase A	GLA	49 kDa	1.68	0.00
Q9H2G2	Cluster of Isoform 2 of STE20-like serine/threonine-protein kinase	SLK	139 kDa	1.78	0.00
O60911	Cathepsin L2	CTSL2	37 kDa	1.89	0.01
Q6NUK1	Calcium-binding mitochondrial carrier protein SCaMC-1	SLC25A24	53 kDa	0.62	0.00
Q9NWU2	Glucose-induced degradation protein 8 homolog	GID8	27 kDa	0.31	0.00
E9PHK0	Tetranectin	CLEC3B	18 kDa	2.03	0.00
P78417	Glutathione S-transferase omega-1	GSTO1	28 kDa	2.09	0.00
D3YTF8	Cluster of Thioredoxin reductase 2, mitochondrial	TXNRD2	54 kDa	0.59	0.00
P60228	Cluster of Eukaryotic translation initiation factor 3 subunit	EIF3E	52 kDa	0.17	0.00
P14678	Cluster of Isoform SM-B1 of Small nuclear ribonucleoprotein-associated proteins B and B'	SNRPB	30 kDa	1.64	0.00
P33992	Cluster of DNA replication licensing factor MCM5	MCM5	82 kDa	2.46	0.00
Q9BQA1	Methylosome protein 50	WDR77	37 kDa	1.84	0.00
Q9UHD1	Cysteine and histidine-rich domain-containing protein 1	CHORDC1	37 kDa	2.50	0.00
Q7L985	Cluster of Leucine-rich repeat and immunoglobulin-like domain-containing nogo receptor-interacting protein 2	LINGO2	68 kDa	0.63	0.01
O15357	Phosphatidylinositol 3,4,5-trisphosphate 5-phosphatase 2	INPPL1	139 kDa	0.51	0.00
O00203	Cluster of AP-3 complex subunit beta-1	AP3B1	121 kDa	1.73	0.00
B4DKB2	Endothelin-converting enzyme 1	ECE1	84 kDa	0.63	0.00
Q15334	Lethal(2) giant larvae protein homolog 1	LLGL1	115 kDa	0.56	0.00
Q8NBN3	Transmembrane protein 87A	TMEM87A	63 kDa	0.51	0.00
E7EPD0	Cluster of Target of Myb protein 1	TOM1	50 kDa	0.42	0.00
Q9BRS2	Serine/threonine-protein kinase RIO1	RIOK1	66 kDa	0.46	0.00
P36873	Cluster of Isoform Gamma-2 of Serine/threonine-protein phosphatase PP1-gamma catalytic subunit	PPP1CC	39 kDa	1.88	0.00
P11717	Cation-independent mannose-6-phosphate receptor	IGF2R	274 kDa	2.81	0.00
O43175	D-3-phosphoglycerate dehydrogenase	PHGDH	57 kDa	2.25	0.00
Q6ZMU5	Tripartite motif-containing protein 72	TRIM72	53 kDa	1.88	0.00
P35573	Glycogen debranching enzyme	AGL	175 kDa	2.38	0.00
Q86UY0	TXNDC5 protein	TXNDC5	40 kDa	2.35	0.00
C9JK10	Cluster of Integrin alpha-6 (Fragment)	ITGA6	122 kDa	2.15	0.00
Q92575	UBX domain-containing protein 4	UBXN4	57 kDa	0.54	0.00
P07951	Cluster of Isoform 3 of Tropomyosin beta chain	TPM2	29 kDa	3.33	0.00
P49915	GMP synthase [glutamine-hydrolyzing]	GMPS	77 kDa	2.00	0.00
Q92859	Neogenin	NEO1	160 kDa	2.00	0.00
F5H737	Adenosylhomocysteinase	AHCY	45 kDa	2.00	0.00
P61289	Proteasome activator complex subunit 3	PSME3	30 kDa	1.67	0.00
Q9UDY4	DnaJ homolog subfamily B member 4	DNAJB4	38 kDa	0.59	0.00
E7ENU4	Double-stranded RNA-specific adenosine deaminase	ADAR	141 kDa	1.67	0.01
Q14139	Ubiquitin conjugation factor E4 A	UBE4A	123 kDa	1.81	0.01
Q15714	TSC22 domain family protein 1	TSC22D1	110 kDa	0.55	0.00

Q9NZN4	Cluster of EH domain-containing protein 2	EHD2	61 kDa	0.50	0.00
Q5R3F8	Protein phosphatase 1 regulatory subunit 29	ELFN2	90 kDa	0.31	0.00
Q9P0L0	Vesicle-associated membrane protein-associated protein A	VAPA	28 kDa	1.67	0.00
A4D1W8	Ependymin related protein 1 (Zebrafish), isoform CRA_b	UCC1	38 kDa	0.60	0.00
Q02790	Cluster of Peptidyl-prolyl cis-trans isomerase FKBP4	FKBP4	52 kDa	1.79	0.00
B4DTU4	Cluster of DNA ligase	LIG1	98 kDa	0.60	0.00
Q15057	Cluster of Arf-GAP with coiled-coil, ANK repeat and PH domain-containing protein 2	ACAP2	88 kDa	1.75	0.01
B4DME2	Protein phosphatase 1 regulatory subunit 12C	PPP1R12C	77 kDa	0.53	0.00
O60921	Cluster of Checkpoint protein HUS1	HUS1	32 kDa	0.46	0.00
P08123	Collagen alpha-2(I) chain	COL1A2	129 kDa	0.42	0.00
P55060	Exportin-2	CSE1L	110 kDa	1.67	0.00
Q96GD0	Pyridoxal phosphate phosphatase	PDXP	32 kDa	0.63	0.00
Q9H5X1	MIP18 family protein FAM96A	FAM96A	18 kDa	0.38	0.00
Q14746	Cluster of Conserved oligomeric Golgi complex subunit 2	COG2	83 kDa	0.60	0.00
Q9HD47	Cluster of Ran guanine nucleotide release factor	RANGRF	20 kDa	0.45	0.00
P46937	Yorkie homolog	YAP1	54 kDa	0.50	0.00
Q9ULH1	Arf-GAP with SH3 domain, ANK repeat and PH domain-containing protein 1	ASAP1	126 kDa	0.47	0.00
Q01650	Large neutral amino acids transporter small subunit 1	SLC7A5	55 kDa	1.67	0.00
G3V141	Collagen triple helix repeat containing 1, isoform CRA_c	CTHRC1	25 kDa	0.63	0.00
Q6P3X3	Tetratricopeptide repeat protein 27	TTC27	97 kDa	0.58	0.00
Q9Y2S7	Polymerase delta-interacting protein 2	POLDIP2	42 kDa	0.39	0.00
P40926	Malate dehydrogenase, mitochondrial	MDH2	36 kDa	2.13	0.00
Q92889	DNA repair endonuclease XPF	ERCC4	104 kDa	0.52	0.00
P31431	Cluster of Syndecan-4	SDC4	22 kDa	2.00	0.00
Q8NEJ9	Cluster of Neuroguidin	NGDN	36 kDa	0.63	0.00
P07737	Profilin-1	PFN1	15 kDa	1.69	0.00
Q96BP3	Peptidylprolyl isomerase domain and WD repeat-containing protein 1	PPWD1	74 kDa	1.69	0.00
Q9NVU7	Protein SDA1 homolog	SDAD1	80 kDa	0.61	0.00
P61218	Cluster of DNA-directed RNA polymerases I, II, and III subunit RPABC2	POLR2F	14 kDa	0.63	0.00
Q00610	Cluster of Clathrin heavy chain 1	CLTC	192 kDa	0.19	0.00
D6RER5	Cluster of Septin-11	SEPT11	50 kDa	2.20	0.00
P36551	Coproporphyrinogen-III oxidase, mitochondrial	CPOX	50 kDa	1.83	0.00
P24534	Elongation factor 1-beta	EEF1B2	25 kDa	3.14	0.00
Q9NVE7	Pantothenate kinase 4	PANK4	86 kDa	0.63	0.00
Q96TC7	Cluster of Regulator of microtubule dynamics protein 3	FAM82A2	52 kDa	0.54	0.00
Q9BUT1	Cluster of 3-hydroxybutyrate dehydrogenase type 2	BDH2	27 kDa	0.52	0.00
P01008	Antithrombin-III	SERPINC1	53 kDa	1.93	0.00
B4DVE7	Cluster of Annexin	ANXA11	51 kDa	2.25	0.00
H0Y6E7	Cluster of RNA-binding motif protein, X chromosome (Fragment)	RBMX	32 kDa	0.52	0.00
Q08722	Isoform OA3-312 of Leukocyte surface antigen CD47	CD47	34 kDa	0.50	0.00
Q9H7P6	Cluster of Multivesicular body subunit 12B	FAM125B	36 kDa	0.41	0.00
O60568	Procollagen-lysine,2-oxoglutarate 5-dioxygenase 3	PLOD3	85 kDa	2.25	0.00
Q9H081	Protein MIS12 homolog	MIS12	24 kDa	0.42	0.00
P09429	Cluster of High mobility group protein B1	HMGB1	25 kDa	2.70	0.00
P07357	Complement component C8 alpha chain	C8A	65 kDa	0.48	0.00
P12830	Cluster of Cadherin-1	CDH1	97 kDa	2.04	0.00
Q9UIV1	CCR4-NOT transcription complex subunit 7	CNOT7	33 kDa	0.44	0.00
B4DTG2	Cluster of Elongation factor 1-gamma	EEF1G	56 kDa	8.43	0.00
Q8NC96	Cluster of Adaptin ear-binding coat-associated protein 1	NECAP1	30 kDa	0.56	0.00
Q96SU4	Isoform 7 of Oxysterol-binding protein-related protein 9	OSBPL9	81 kDa	0.30	0.05
Q9UBG0	C-type mannose receptor 2	MRC2	167 kDa	0.54	0.00

P08107	Cluster of Heat shock 70 kDa protein 1A/1B	HSPA1A	70 kDa	2.90	0.00
P22314	Cluster of Ubiquitin-like modifier-activating enzyme 1	UBA1	118 kDa	2.07	0.00
P37837	Transaldolase	TALDO1	38 kDa	1.61	0.00
E7EPA7	Transketolase	TKT	69 kDa	3.63	0.00
P01024	Complement C3	C3	187 kDa	3.00	0.00
Q9Y2Q5	Ragulator complex protein LAMTOR2	LAMTOR2	14 kDa	0.52	0.00
P61201	COP9 signalosome complex subunit 2	COPS2	52 kDa	1.71	0.00
O75843	Cluster of AP-1 complex subunit gamma-like 2	AP1G2	87 kDa	0.57	0.00
O14732	Inositol monophosphatase 2	IMPA2	31 kDa	0.40	0.00
A6NFM0	Cluster of DNA-directed RNA polymerase II subunit RPB11-b1	POLR2J2	17 kDa	0.57	0.00
P25788	Proteasome subunit alpha type-3	PSMA3	28 kDa	1.89	0.00
Q12769	Cluster of Nuclear pore complex protein Nup160	NUP160	162 kDa	0.55	0.00
Q92974	Rho guanine nucleotide exchange factor 2	ARHGEF2	112 kDa	0.55	0.00
P29372	Isoform 2 of DNA-3-methyladenine glycosylase	MPG	32 kDa	0.52	0.00
G3V4K3	Spermatogenesis-defective protein 39 homolog	VIPAS39	60 kDa	0.44	0.00
P19387	DNA-directed RNA polymerase II subunit RPB3	POLR2C	31 kDa	0.25	0.00
Q01813	Cluster of 6-phosphofructokinase type C	PFKP	86 kDa	1.70	0.00
Q01970	1-phosphatidylinositol 4,5-bisphosphate phosphodiesterase beta-3	PLCB3	139 kDa	1.70	0.00
P50225	Cluster of Sulfotransferase 1A1	SULT1A1	34 kDa	0.57	0.00
O95104	Splicing factor, arginine/serine-rich 15	SCAF4	126 kDa	0.40	0.00
P02786	Cluster of Transferrin receptor protein 1	TFRC	85 kDa	1.70	0.01
Q09666	Cluster of Neuroblast differentiation-associated protein AHNAK	AHNAK	629 kDa	1.70	0.01
P10301	Ras-related protein R-Ras	RRAS	23 kDa	0.63	0.00
Q14264	HERV-R_7q21.2 provirus ancestral Env polyprotein	ERV3-1	68 kDa	0.63	0.00
E5RFU2	Bifunctional epoxide hydrolase 2	EPHX2	59 kDa	0.63	0.00
O94813	Slit homolog 2 protein	SLIT2	170 kDa	0.49	0.00
Q9UBT2	SUMO-activating enzyme subunit 2	UBA2	71 kDa	1.70	0.00
Q15031	Probable leucine--tRNA ligase, mitochondrial	LARS2	102 kDa	0.45	0.00
A6NE09	Protein RPSAP58	RPSAP58	33 kDa	0.21	0.00
Q14997	Proteasome activator complex subunit 4	PSME4	211 kDa	1.64	0.00
Q99622	Protein C10	C12orf57	13 kDa	0.25	0.04
Q9UKZ9	Procollagen C-endopeptidase enhancer 2	PCOLCE2	46 kDa	0.62	0.00
Q96ES7	SAGA-associated factor 29 homolog	CCDC101	33 kDa	0.62	0.00
B4E0G4	PHD finger protein 6	PHF6	38 kDa	0.57	0.00
O15084	Serine/threonine-protein phosphatase 6 regulatory ankyrin repeat subunit A	ANKRD28	113 kDa	0.40	0.00
O60244	Mediator of RNA polymerase II transcription subunit 14	MED14	161 kDa	0.55	0.00
P10809	Cluster of 60 kDa heat shock protein, mitochondrial	HSPD1	61 kDa	2.21	0.00
Q99828	Calcium and integrin-binding protein 1	CIB1	22 kDa	0.55	0.00
P23921	Ribonucleoside-diphosphate reductase large subunit	RRM1	90 kDa	1.73	0.00
Q12905	Interleukin enhancer-binding factor 2	ILF2	43 kDa	2.63	0.00
P06744	Glucose-6-phosphate isomerase	GPI	63 kDa	3.80	0.00
Q13813	Cluster of Spectrin alpha chain, non-erythrocytic 1	SPTAN1	285 kDa	2.71	0.00
H3BM09	Cluster of HLA class I histocompatibility antigen, A-68 alpha chain (Fragment)	HLA-A	34 kDa	2.11	0.00
P62258	Cluster of 14-3-3 protein epsilon	YWHAE	29 kDa	1.73	0.00
O15355	Protein phosphatase 1G	PPM1G	59 kDa	1.73	0.00
P35221	Cluster of Catenin alpha-1	CTNNA1	100 kDa	1.73	0.00
Q13362	Isoform 4 of Serine/threonine-protein phosphatase 2A 56 kDa regulatory subunit gamma isoform	PPP2R5C	63 kDa	0.59	0.00
E9PQE8	Cluster of Trafficking protein particle complex subunit 4	TRAPPC4	19 kDa	0.56	0.00
H3BNK9	Protein FAM192A (Fragment)	FAM192A	16 kDa	0.50	0.00
E7ER20	Cluster of Proteasome inhibitor PI31 subunit	PSMF1	22 kDa	0.61	0.00

Q7Z7E8	Ubiquitin-conjugating enzyme E2 Q1	UBE2Q1	46 kDa	0.58	0.00
E9PJM3	Cluster of F-box only protein 3	FBXO3	49 kDa	0.51	0.00
P98160	Cluster of Basement membrane-specific heparan sulfate proteoglycan core protein	HSPG2	469 kDa	3.17	0.00
F8W8J4	Myoferlin	MYOF	235 kDa	1.90	0.00
O75533	Splicing factor 3B subunit 1	SF3B1	146 kDa	1.90	0.00
P16403	Cluster of Histone H1.2	HIST1H1C	21 kDa	1.90	0.00
P27348	Cluster of 14-3-3 protein theta	YWHAQ	28 kDa	1.90	0.00
Q8TAA9	Vang-like protein 1	VANGL1	60 kDa	0.56	0.00
O95999	B-cell lymphoma/leukemia 10	BCL10	26 kDa	0.53	0.00
Q8NFU3	Thiosulfate sulfurtransferase/rhodanese-like domain-containing protein 1	TSTD1	13 kDa	0.48	0.00
Q9UM54	Cluster of Isoform 6 of Unconventional myosin-VI	MYO6	149 kDa	1.65	0.01
P05455	Lupus La protein	SSB	47 kDa	2.36	0.00
P50750	Isoform 2 of Cyclin-dependent kinase 9	CDK9	53 kDa	0.59	0.00
Q96QU8	Exportin-6	XPO6	129 kDa	0.50	0.00
Q86UN2	Reticulon-4 receptor-like 1	RTN4RL1	49 kDa	0.47	0.00
Q15075	Early endosome antigen 1	EEA1	162 kDa	1.65	0.00
Q8IUL8	Cartilage intermediate layer protein 2	CILP2	126 kDa	1.65	0.01
Q6P4I2	WD repeat-containing protein 73	WDR73	42 kDa	0.55	0.00
B1AUU8	Cluster of Epidermal growth factor receptor pathway substrate 15	EPS15	84 kDa	0.52	0.00
Q96BH1	E3 ubiquitin-protein ligase RNF25	RNF25	51 kDa	0.58	0.00
P15880	Cluster of 40S ribosomal protein S2	RPS2	31 kDa	1.87	0.00
I3L3S0	Trafficking protein particle complex subunit 1 (Fragment)	TRAPPC1	11 kDa	0.62	0.00
Q01518	Cluster of Adenylyl cyclase-associated protein 1	CAP1	52 kDa	4.19	0.00
Q9Y5B9	FACT complex subunit SPT16	SUPT16H	120 kDa	2.50	0.00
P22102	Cluster of Trifunctional purine biosynthetic protein adenosine-3	GART	108 kDa	1.94	0.00
Q2M2I8	Isoform 2 of AP2-associated protein kinase 1	AAK1	94 kDa	0.63	0.00
Q92785	Zinc finger protein ubi-d4	DPF2	44 kDa	0.63	0.00
Q7L3B6	Hsp90 co-chaperone Cdc37-like 1	CDC37L1	39 kDa	0.58	0.00
Q7Z417	Nuclear fragile X mental retardation-interacting protein 2	NUFIP2	76 kDa	0.44	0.00
Q92882	Osteoclast-stimulating factor 1	OSTF1	24 kDa	0.60	0.00
Q04837	Single-stranded DNA-binding protein, mitochondrial	SSBP1	17 kDa	0.60	0.00
Q86X83	COMM domain-containing protein 2	COMMD2	23 kDa	0.56	0.00
P07108	Cluster of Isoform 4 of Acyl-CoA-binding protein	DBI	14 kDa	0.53	0.00
F5GWN1	Protein SSXT	SS18	4 kDa	0.41	0.00
P68104	Elongation factor 1-alpha 1	EEF1A1	50 kDa	2.19	0.00
P43490	Nicotinamide phosphoribosyltransferase	NAMPT	56 kDa	1.75	0.00
Q14181	DNA polymerase alpha subunit B	POLA2	66 kDa	0.40	0.00
A0FGR8	Cluster of Isoform 2 of Extended synaptotagmin-2	ESYT2	99 kDa	0.58	0.00
Q9BX68	Histidine triad nucleotide-binding protein 2, mitochondrial	HINT2	17 kDa	0.55	0.00
P12429	Annexin A3	ANXA3	36 kDa	3.00	0.00
P61006	Cluster of Ras-related protein Rab-8A	RAB8A	24 kDa	1.91	0.00
Q9NS15	Cluster of Latent-transforming growth factor beta-binding protein 3	LTBP3	139 kDa	1.91	0.00
Q9NTK5	Isoform 3 of Obg-like ATPase 1	OLA1	31 kDa	1.62	0.00
E9PFW3	AP-2 complex subunit mu	AP2M1	52 kDa	1.62	0.00
E9PMG1	Cluster of RalBP1-associated Eps domain-containing protein 1	REPS1	80 kDa	0.51	0.00
Q16706	Cluster of Alpha-mannosidase 2	MAN2A1	131 kDa	1.75	0.00
Q9UGI8	Cluster of Isoform 2 of Testin	TES	47 kDa	2.10	0.00
P52907	F-actin-capping protein subunit alpha-1	CAPZA1	33 kDa	1.75	0.00
J3QRZ5	Ubiquitin carboxyl-terminal hydrolase	USP14	52 kDa	2.14	0.00
P18084	Integrin beta-5	ITGB5	88 kDa	1.67	0.00

Q01581	Cluster of Hydroxymethylglutaryl-CoA synthase, cytoplasmic	HMGCS1	57 kDa	1.85	0.00
P07814	Bifunctional glutamate/proline--tRNA ligase	EPRS	171 kDa	2.06	0.00
Q9Y2H0	Cluster of Isoform 2 of Disks large-associated protein 4	DLGAP4	108 kDa	0.58	0.00
C9JFV8	Deoxyguanosine kinase, mitochondrial	DGUOK	30 kDa	0.51	0.00
B7ZKK9	Cluster of PPP2R5E protein	PPP2R5E	54 kDa	0.51	0.00
Q99747	Gamma-soluble NSF attachment protein	NAPG	35 kDa	0.56	0.00
F5H098	Malate dehydrogenase, cytoplasmic	MDH1	39 kDa	1.85	0.00
E9PRZ9	Protein C11orf58 (Fragment)	C11orf58	10 kDa	0.58	0.00
Q9H9A6	Leucine-rich repeat-containing protein 40	LRRC40	68 kDa	0.51	0.00
Q9NZJ9	Cluster of Diphosphoinositol polyphosphate phosphohydrolase 2	NUDT4	20 kDa	0.60	0.00
Q969V3	Nicalin	NCLN	63 kDa	0.53	0.00
P18669	Cluster of Phosphoglycerate mutase 1	PGAM1	29 kDa	1.85	0.00
Q8N108	Cluster of Mesoderm induction early response protein 1	MIER1	58 kDa	0.62	0.00
P29728	2'-5'-oligoadenylate synthase 2	OAS2	82 kDa	0.62	0.00
D6RF48	Syntaxin-18	STX18	35 kDa	0.58	0.00
Q96CW5	Gamma-tubulin complex component 3	TUBGCP3	104 kDa	0.61	0.00
Q15363	Transmembrane emp24 domain-containing protein 2	TMED2	23 kDa	0.63	0.00
Q86Y38	Xylosyltransferase 1	XYLT1	108 kDa	0.63	0.00
O00425	Insulin-like growth factor 2 mRNA-binding protein 3	IGF2BP3	64 kDa	0.56	0.00
Q96IY1	Cluster of Kinetochore-associated protein NSL1 homolog	NSL1	32 kDa	0.56	0.00
Q9NRN7	L-aminoadipate-semialdehyde dehydrogenase-phosphopantetheinyl transferase	AASDHPPT	36 kDa	0.61	0.00
O43776	Asparagine--tRNA ligase, cytoplasmic	NARS	63 kDa	2.44	0.00
P53621	Coatomer subunit alpha	COPA	138 kDa	2.44	0.00
F5H2A7	Prefoldin subunit 3	VBP1	22 kDa	1.63	0.00
Q9BYG3	MKI67 FHA domain-interacting nucleolar phosphoprotein	MKI67IP	34 kDa	0.44	0.00
Q99615	DnaJ homolog subfamily C member 7	DNAJC7	56 kDa	1.64	0.00
Q9C0B1	Cluster of Alpha-ketoglutarate-dependent dioxygenase FTO	FTO	58 kDa	1.64	0.00
P12109	Collagen alpha-1(VI) chain	COL6A1	109 kDa	4.60	0.00
P24821	Isoform 6 of Tenascin	TNC	171 kDa	0.58	0.00
P17931	Cluster of Galectin-3	LGALS3	26 kDa	1.64	0.00
B5MCQ5	Protein disulfide-isomerase A6	PDIA6	53 kDa	1.64	0.00
P17174	Cluster of Aspartate aminotransferase, cytoplasmic	GOT1	46 kDa	2.09	0.00
P62699	Protein yippee-like 5	YPEL5	14 kDa	0.32	0.05
P60033	Cluster of CD81 antigen	CD81	26 kDa	1.92	0.00
E9PDC5	Receptor-type tyrosine-protein phosphatase S	PTPRS	217 kDa	1.92	0.00
F6QR24	Nuclear pore complex protein Nup153	NUP153	157 kDa	0.60	0.00
P17655	Cluster of Calpain-2 catalytic subunit	CAPN2	80 kDa	3.42	0.00
O75083	Cluster of WD repeat-containing protein 1	WDR1	66 kDa	2.56	0.00
P09234	Cluster of U1 small nuclear ribonucleoprotein C	SNRPC	17 kDa	0.51	0.00
P45985	Dual specificity mitogen-activated protein kinase kinase 4	MAP2K4	44 kDa	0.57	0.00
O00299	Chloride intracellular channel protein 1	CLIC1	27 kDa	2.39	0.00
P46060	Cluster of Ran GTPase-activating protein 1	RANGAP1	64 kDa	1.65	0.00
Q9NRL2	Bromodomain adjacent to zinc finger domain protein 1A	BAZ1A	179 kDa	0.25	0.05
Q15648	Mediator of RNA polymerase II transcription subunit 1	MED1	168 kDa	0.54	0.00
Q15181	Inorganic pyrophosphatase	PPA1	33 kDa	1.65	0.00
D6R9T0	Cluster of Heterogeneous nuclear ribonucleoprotein H, N-terminally processed (Fragment)	HNRNPH1	18 kDa	1.65	0.00
O75521	Enoyl-CoA delta isomerase 2, mitochondrial	ECI2	44 kDa	0.54	0.00
P80303	Cluster of Nucleobindin-2	NUCB2	50 kDa	2.27	0.00
P43243	Cluster of Matrin-3	MATR3	95 kDa	1.92	0.00
Q5JPT4	SH3 domain-containing kinase-binding protein 1	SH3KBP1	47 kDa	1.67	0.00
C9JJV1	Protein TSSC4 (Fragment)	TSSC4	22 kDa	0.52	0.00
Q8NC60	Nitric oxide-associated protein 1	NOA1	78 kDa	0.47	0.00

E9PEZ3	Cluster of Protein diaphanous homolog 1	DIAPH1	141 kDa	2.50	0.00
C9JPM4	ADP-ribosylation factor 4 (Fragment)	ARF4	15 kDa	1.67	0.00
Q15008	Cluster of 26S proteasome non-ATPase regulatory subunit 6	PSMD6	46 kDa	2.05	0.00
O43396	Cluster of Thioredoxin-like protein 1	TXNL1	32 kDa	1.61	0.00
O60645	Exocyst complex component 3	EXOC3	87 kDa	0.63	0.00
Q9BSB4	Autophagy-related protein 101	ATG101	25 kDa	0.43	0.00
Q14008	Cluster of Cytoskeleton-associated protein 5	CKAP5	226 kDa	2.25	0.00
P25787	Proteasome subunit alpha type-2	PSMA2	26 kDa	1.61	0.00
Q9NRB3	Carbohydrate sulfotransferase 12	CHST12	48 kDa	0.39	0.00
Q9NY97	UDP-GlcNAc:betaGal beta-1,3-N-acetylglucosaminyltransferase 2	B3GNT2	46 kDa	1.80	0.00
Q9P2E9	Cluster of Ribosome-binding protein 1	RRBP1	152 kDa	2.14	0.00
Q96QK1	Cluster of Vacuolar protein sorting-associated protein 35	VPS35	92 kDa	2.35	0.00
Q00839	Cluster of Heterogeneous nuclear ribonucleoprotein U	HNRNPU	91 kDa	1.68	0.00
Q2NL82	Pre-rRNA-processing protein TSR1 homolog	TSR1	92 kDa	0.54	0.00
Q8IZ83	Cluster of Aldehyde dehydrogenase family 16 member A1	ALDH16A1	85 kDa	2.23	0.00
O95373	Importin-7	IPO7	120 kDa	2.42	0.00
P31153	Cluster of S-adenosylmethionine synthase isoform type-2	MAT2A	44 kDa	2.13	0.00
P61353	60S ribosomal protein L27	RPL27	16 kDa	2.21	0.00
O96005	Cleft lip and palate transmembrane protein 1	CLPTM1	76 kDa	0.50	0.00
E9PCI3	Asparagine synthetase	ASNS	62 kDa	3.31	0.00
P04792	Cluster of Heat shock protein beta-1	HSPB1	23 kDa	1.89	0.00
P18754	Cluster of Isoform 2 of Regulator of chromosome condensation	RCC1	48 kDa	1.83	0.00
Q5H9R7	Cluster of Isoform 5 of Serine/threonine-protein phosphatase 6 regulatory subunit 3	PPP6R3	98 kDa	1.68	0.00
Q9Y697	Cluster of Isoform Cytoplasmic of Cysteine desulfurase, mitochondrial	NFS1	44 kDa	0.52	0.00
O15031	Plexin-B2	PLXNB2	205 kDa	2.20	0.00
J3KQ34	COP9 signalosome complex subunit 7b	COPS7B	26 kDa	1.84	0.00
P04075	Cluster of Fructose-bisphosphate aldolase A	ALDOA	39 kDa	8.38	0.00
Q86VP6	Cluster of Cullin-associated NEDD8-dissociated protein 1	CAND1	136 kDa	6.08	0.00
O00232	26S proteasome non-ATPase regulatory subunit 12	PSMD12	53 kDa	3.04	0.00
P63104	Cluster of 14-3-3 protein zeta/delta	YWHAZ	28 kDa	2.86	0.05
P50914	60S ribosomal protein L14	RPL14	23 kDa	2.36	0.04
Q2TAY7	Cluster of WD40 repeat-containing protein SMU1	SMU1	58 kDa	1.88	0.04
P62834	Cluster of Ras-related protein Rap-1A	RAP1A	21 kDa	0.38	0.05
Q07960	Rho GTPase-activating protein 1	ARHGAP1	50 kDa	0.29	0.04
Q9NXV6	CDKN2A-interacting protein	CDKN2AIP	61 kDa	0.44	0.04
G3V1U8	CDK-activating kinase assembly factor MAT1	MNAT1	31 kDa	0.55	0.05
Q16762	Thiosulfate sulfurtransferase	TST	33 kDa	0.52	0.04
Q5NDL2	EGF domain-specific O-linked N-acetylglucosamine transferase	EOGT	62 kDa	0.55	0.04
Q8WXI9	Transcriptional repressor p66-beta	GATAD2B	65 kDa	0.51	0.04
Q9NQZ2	Something about silencing protein 10	UTP3	55 kDa	0.63	0.05
H7BZW1	Cluster of Uncharacterized protein (Fragment)		34 kDa	0.59	0.04
P11586	Cluster of C-1-tetrahydrofolate synthase, cytoplasmic	MTHFD1	102 kDa	2.20	0.00
Q14118	Dystroglycan	DAG1	97 kDa	1.60	0.00
Q9UBS4	Cluster of DnaJ homolog subfamily B member 11	DNAJB11	41 kDa	1.60	0.00
P26447	Protein S100-A4	S100A4	12 kDa	2.00	0.00
Q9Y3I0	tRNA-splicing ligase RtcB homolog	C22orf28	55 kDa	1.83	0.00
E9PLM6	Midkine	MDK	17 kDa	1.83	0.00
H0Y7A7	Calmodulin (Fragment)	CALM2	21 kDa	1.83	0.00
P49189	4-trimethylaminobutyraldehyde dehydrogenase	ALDH9A1	54 kDa	1.83	0.00
P54727	Cluster of UV excision repair protein RAD23 homolog B	RAD23B	43 kDa	1.71	0.00

Q9Y2Z0	Suppressor of G2 allele of SKP1 homolog	SUGT1	41 kDa	1.63	0.00
F5H2U2	Cluster of Serine/threonine-protein kinase PRP4 homolog	PRPF4B	115 kDa	0.50	0.00
P34096	Ribonuclease 4	RNASE4	17 kDa	0.58	0.00
P48745	Protein NOV homolog	NOV	39 kDa	0.62	0.00
Q15386	Ubiquitin-protein ligase E3C	UBE3C	124 kDa	0.62	0.00
Q8IWZ8	SURP and G-patch domain-containing protein 1	SUGP1	72 kDa	0.46	0.00
B3KQV7	Equilibrative nucleoside transporter 1	SLC29A1	59 kDa	0.53	0.00
Q9NX70	Mediator of RNA polymerase II transcription subunit 29	MED29	21 kDa	0.33	0.00
Q5JTV8	Torsin-1A-interacting protein 1	TOR1AIP1	66 kDa	0.50	0.00
B4DP36	Calcium-binding and coiled-coil domain-containing protein	CALCOCO2	55 kDa	0.35	0.00
E9PC90	Cluster of G2/mitotic-specific cyclin-B1 (Fragment)	CCNB1	45 kDa	0.30	0.00
G8JLI5	WD repeat domain phosphoinositide-interacting protein 4	WDR45	38 kDa	0.43	0.00

Supplementary Table S6. The shared differentially expressed proteins between the HCT116-*TP53*(R273H) (MT) vs. HCT116-*TP53* (WT), and HCT116-*TP53*$^{(-/-)}$ (KO) vs. HCT116-*TP53* (WT) groups.

Accession Number	Protein subgroup name	Gene symbol	Molecular Weight (kDa)	MT/WT		KO/WT	
				Fold Change	*P*-value	Fold Change	*P*-value
P14618	Cluster of Isoform 3 of Pyruvate kinase isozymes M1/M2	PKM	56	0.10	0.0000	0.10	0.0000
J3KQQ3	Zinc finger and BTB domain-containing protein 7B	ZBTB7B	62	0.43	0.0046	3.15	0.0046
P49327	Fatty acid synthase	FASN	273	0.43	0.0002	0.20	0.0002
P15531	Cluster of Nucleoside diphosphate kinase A	NME1	17	0.43	0.0002	0.30	0.0002
P53618	Coatomer subunit beta	COPB1	107	0.50	0.0058	0.40	0.0058
Q02818	Cluster of Nucleobindin-1	NUCB1	54	0.50	0.0032	0.40	0.0032
E7EP74	Cluster of Golgin subfamily B member 1	GOLGB1	377	0.50	0.0000	0.50	0.0000
Q7L2H7	Eukaryotic translation initiation factor 3 subunit M	EIF3M	43	0.53	0.0022	0.10	0.0022
P13667	Cluster of Protein disulfide-isomerase A4	PDIA4	73	0.53	0.0022	0.25	0.0022
P26639	Cluster of Threonine--tRNA ligase, cytoplasmic	TARS	83	0.53	0.0048	0.30	0.0048
Q16531	Cluster of DNA damage-binding protein 1	DDB1	127	0.53	0.0022	0.35	0.0022
O15230	Laminin subunit alpha-5	LAMA5	400	0.55	0.0006	0.30	0.0006
P11142	Cluster of Heat shock cognate 71 kDa protein	HSPA8	71	0.60	0.0220	0.25	0.0220
P05783	Cluster of Keratin, type I cytoskeletal 18	KRT18	48	0.60	0.0023	0.35	0.0023
Q02790	Cluster of Peptidyl-prolyl cis-trans isomerase FKBP4	FKBP4	52	0.63	0.0006	0.35	0.0006
P07996	Cluster of Thrombospondin-1	THBS1	129	0.63	0.0006	0.40	0.0006
O14964	Hepatocyte growth factor-regulated tyrosine kinase substrate	HGS	86	0.63	0.0043	0.60	0.0043
Q9NWU2	Glucose-induced degradation protein 8 homolog	GID8	27	0.58	0.0004	1.85	0.0004
E9PC90	Cluster of G2/mitotic-specific cyclin-B1 (Fragment)	CCNB1	45	0.60	0.0000	2.00	0.0000
P46100	Transcriptional regulator ATRX	ATRX	283	0.63	0.0043	2.55	0.0043
P04075	Cluster of Fructose-bisphosphate aldolase A	ALDOA	39	1.68	0.0001	0.20	0.0001
Q01518	Cluster of Adenylyl cyclase-associated protein 1	CAP1	52	1.68	0.0008	0.40	0.0008

P06748	Cluster of Nucleophosmin	NPM1	33	1.73	0.0446	0.35	0.0446
Q86VP6	Cluster of Cullin-associated NEDD8-dissociated protein 1	CAND1	136	1.83	0.0001	0.30	0.0001
B4DTG2	Cluster of Elongation factor 1-gamma	EEF1G	56	2.95	0.0003	0.35	0.0003
O00232	26S proteasome non-ATPase regulatory subunit 12	PSMD12	53	1.83	0.0001	0.60	0.0001
Q92945	Far upstream element-binding protein 2	KHSRP	73	2.20	0.0133	0.50	0.0133
P07900	Cluster of Heat shock protein HSP 90-alpha	HSP90AA1	85	1.60	0.0163	8.25	0.0163
Q16254	Transcription factor E2F4	E2F4	44	1.60	0.0321	4.10	0.0321
Q96T23	Remodeling and spacing factor 1	RSF1	164	1.63	0.0001	1.60	0.0001
P49959	Cluster of Isoform 2 of Double-strand break repair protein MRE11A	MRE11A	78	1.63	0.0110	1.75	0.0110
Q9Y3C7	Mediator of RNA polymerase II transcription subunit 31	MED31	16	1.67	0.0002	2.24	0.0002
P07902	Cluster of Galactose-1-phosphate uridylyltransferase	GALT	43	1.68	0.0001	2.50	0.0001
Q92636	Protein FAN	NSMAF	104	1.70	0.0176	2.40	0.0176
O75143	Cluster of Isoform 3 of Autophagy-related protein 13	ATG13	44	1.79	0.0040	2.16	0.0040
Q9H1K1	Iron-sulfur cluster assembly enzyme ISCU, mitochondrial	ISCU	18	2.15	0.0004	3.40	0.0004
P82932	28S ribosomal protein S6, mitochondrial	MRPS6	14	2.30	0.0053	2.45	0.0053
E7ETA6	Pericentriolar material 1 protein	PCM1	228	2.35	0.0068	1.65	0.0068
Q9NQZ5	StAR-related lipid transfer protein 7, mitochondrial	STARD7	43	4.08	0.0109	2.00	0.0109
E9PB30	Anoctamin	ANO6	104	4.38	0.0201	3.40	0.0201
C9J180	Ubiquitin-conjugating enzyme E2 E2 (Fragment)	UBE2E2	18	4.53	0.0014	3.70	0.0014
